# Supplementary material for: Regioselective fluorination of allenes enabled by I(I)/I(III) catalysis
Source: Nat Commun. 2024 Jul 9;15:5770. doi: 10.1038/s41467-024-50227-x (PMC11233658; doi:10.1038/s41467-024-50227-x)
Supplement: Supplementary file 1 — Supplementary Information [file 41467_2024_50227_MOESM1_ESM.pdf]

Supplementary Information

Regioselective Fluorination of Allenes Enabled by I(I)/I(III) Catalysis

Zi-Xuan Wang,[1] Yameng Xu,[1] Ryan Gilmour[1]*

[1] Institute for Organic Chemistry, University of Münster, Corrensstraße 36, 48149 Münster (Germany)

*E-mail: ryan.gilmour@uni-muenster.de

Table of Contents

1. Supplementary Methods 3
1.1 General information 3
1.2 Preparation and characterisation of allenes 5
1.3 Preparation and characterisation of catalysts 31
1.4 Preparation and characterisation of propargylic fluorides 32
1.5 Synthetic applications 54
1.5.1 Product derivatisation 54
1.5.2 Sequential reaction 58
1.5.3 Preliminary validation of enantioselective catalysis 60
1.6 General catalytic functionalisation of allenes 62
1.7 NMR-Spectra of key compounds 65
2. Supplementary References 174

1. Supplementary Methods

1.1 General information

All commercially available reagents were purchased as reagent grade from *Sigma Aldrich*, *Merck*, *Alfa Aesar*, *TCI*, *Fluorochem* or *abcr* and were used without further purification unless otherwise stated. Solvents for extractions or chromatographic purifications were bought as technical grade and distilled on a rotary evaporator prior to use. All reactions with HF were run in Teflon® vials. For analytical thin layer chromatography, glass plates coated with SiO_2-60 F254 were used from *Merck*. They were visualized with UV-light (254 nm) or with $KMnO_4$ or CAM solution. Column chromatography was performed using silica gel (40-63 µm, *VWR Chemicals*). For preparative thin layer chromatography, glass plates coated with SiO_2-60 F254 and 2 mm thickness were used from *Merck.* The obtained products are often volatile and care must be taken in the isolation. The NMR measurements were performed on a *Bruker AV300*, *AV400*, *Agilent DD2 500* or an *Agilent DD2 600* by the NMR service department of the Organisch-Chemisches Institut, University of Münster. The chemical shifts were referenced to the residual solvent peak as the internal standard (7.26 ppm for $CDCl_3$, 2.50 ppm for DMSO-d_6 for ^{1}H-NMR, 77.16 ppm for $CDCl_3$, 39.52 ppm for DMSO-d_6 for ^{13}C-NMR). The multiplicity is abbreviated as follows: s (singlet), d (doublet), t (triplet), q (quartet), p (pentet), h (heptet), m (multiplet) and br (broad). The given assignments are supported by additional 1D and 2D NMR experiments. The melting points were determined on a *Büchi* B-545 melting point apparatus with open glass capillaries. The IR measurements were performed on a *Perkin-Elmer 100 FT-IR* spectrometer and the intensities of the bands are assigned as follows: w (weak), m (medium), s (strong). High resolution mass spectrometry was performed by the MS service of the Organisch-Chemisches Institut, University of Münster on a Bruker Daltonics MicroTof (HRMS-ESI), a Triplequad TSQ 7000 (MS-EI), Triplequad Quattro Micro GC (GC-EI-MS), a Qp5050 Single Quad (GC-EI-MS) or a LTQ Orbitap LTQ XL (HRMS-APCI).

Preparation of various amine·HF mixtures

Amine·HF sources:

NEt_3·3HF; Supplier: abcr; (MW: 161.21g/mol, ρ = 0.990 g/mL)

Olah's Reagent (70wt% HF: Py·9.23HF); Supplier: Sigma Aldrich; (MW: 263.79 g/mol, ρ = 1.1 g/mL)

Procedure for calculating compositions of amine·HF mixtures:

The amine·HF mixtures based on NEt_3·3HF and Olah's reagent reagent were prepared following the procedure previously described by this group.[1]

1:4.5 (0.5 mL): 0.34 mL NEt_3·3HF and 0.16 mL Olah's reagent

1:5.0 (0.5 mL): 0.29 mL NEt_3·3HF and 0.21 mL Olah's reagent

1:5.5 (0.5 mL): 0.25 mL NEt_3·3HF and 0.25 mL Olah's reagent

1:6.0 (0.5 mL): 0.21 mL NEt_3·3HF and 0.29 mL Olah's reagent

1.2 Preparation and characterisation of allenes

General Procedure A

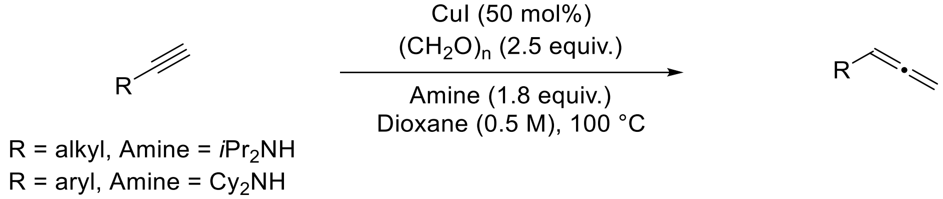

According to a modified literature procedure,[2] to a pressure tube was added CuI (50 mol%), $(CH_2O)_n$ (2.5 equiv.), dioxane (0.50 M), alkyne (1.0 equiv.), and amine (1.8 equiv.) sequentially under Ar atmosphere. The reaction mixture was heated to 100 °C and stirred 24 h. After completion of the reaction, diethyl ether was added, and the reaction mixture was filtrated through a small pad of silica gel to remove insoluble solid. The filtrate was then concentrated under reduced pressure and the residue was purified by column chromatography (SiO_2, specified combination of solvents).

Pentadeca-1,2-diene (S1)

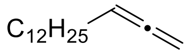

Compound **S1** was prepared according to General Procedure **A** using 1-tetradecyne (777.4 mg, 4.00 mmol, 1.0 eq.). The crude residue was purified by column chromatography (*n*-pentane) to yield the title compound as a colorless oil (660.8 mg, 3.17 mmol, 79%).

R*f* = 0.95 (*n*-pentane).

^{1}H NMR (400 MHz, $CDCl_3$) δ 5.17 – 5.03 (m, 1H), 4.64 (dt, *J* = 6.6, 3.2 Hz, 2H), 2.07 – 1.92 (m, 2H), 1.46 – 1.36 (m, 2H), 1.35 – 1.17 (m, 18H), 0.88 (t, *J* = 6.7 Hz, 3H).

GC-EI-MS: (*m/z*) requires: [($C_{15}H_{28}$)] = 208.2191, (*m/z*) found: [($C_{15}H_{28}$)] = 208.2184.

Analytical data is in agreement with literature values.[3]

Buta-2,3-dien-1-ylcyclohexane (S2)

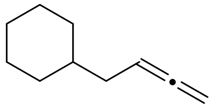

Compound **S2** was prepared according to General Procedure **A** using prop-2-ynylcyclohexane (488.8 mg, 4.00 mmol, 1.0 eq.). The crude residue was purified by column chromatography (*n*-pentane) to yield the title compound as a colorless oil (266.1 mg, 1.95 mmol, 49%).

R*f* = 0.90 (*n*-pentane).

^{1}H NMR (400 MHz, $CDCl_3$) δ 5.05 (p, *J* = 7.1 Hz, 1H), 4.62 (dt, *J* = 6.8, 2.8 Hz, 2H), 1.90 (tt, *J* =

7.1, 2.9 Hz, 2H), 1.79 – 1.60 (m, 5H), 1.39 – 1.08 (m, 4H), 0.98 – 0.84 (m, 2H).

GC-EI-MS: (*m/z*) requires: $[(C_{10}H_{16})]$ = 136.1252, (*m/z*) found: $[(C_{10}H_{16})]$ = 136.1246.

Analytical data is in agreement with literature values.[2]

Dodeca-10,11-dien-1-ol (S6)

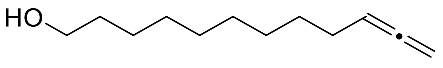

Compound **S6** was prepared according to General Procedure **A** using undec-10-yn-1-ol (1.01 g, 6.00 mmol, 1.0 eq.). The crude residue was purified by column chromatography (*n*-pentane: Et_2O 2:1) to yield the title compound as a colorless oil (730.1 mg, 4.00 mmol, 67%).

$\mathbf{R}_f$ = 0.40 (*n*-pentane:Et_2O 2:1).

^{1}H NMR (400 MHz, $CDCl_3$) δ 5.09 (p, *J* = 6.8 Hz, 1H), 4.64 (dt, *J* = 6.5, 3.2 Hz, 2H), 3.64 (q, *J* = 6.1 Hz, 2H), 2.05 – 1.92 (m, 2H), 1.62 – 1.50 (m, 2H), 1.45 – 1.28 (m, 12H), 1.23 (t, *J* = 5.1 Hz, 1H).

ESI-MS: (*m/z*) requires: $[(C_{12}H_{22}OAg)^+]$ = 289.0716, (*m/z*) found: $[(C_{12}H_{22}OAg)^+]$ = 289.0717.

Analytical data is in agreement with literature values.[4]

12-Bromododeca-1,2-diene (S3)

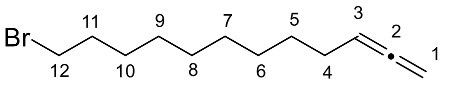

Under argon atmosphere, to a solution of dodeca-10,11-dien-1-ol (**S6**) (182.3 mg, 1.00 mmol, 1.0 eq.) and CBr_4 (364.8 mg, 1.10 mmol, 1.1 eq.) in dry THF (2 mL) was added PPh_3 (288.5 mg, 1.10 mmol, 1.1 eq.) at 0 °C. The reaction mixture was allowed to warm to room temperature and stir for 16 h. After removal of the solvent, the residue was directly purified by column chromatography (*n*-pentane) to yield the title compound as a colorless oil (162.4 mg, 0.66 mmol, 66%).

$\mathbf{R}_f$ = 0.80 (*n*-pentane).

^{1}H NMR (599 MHz, $CDCl_3$) δ 5.09 (p, $^3J_{HH}$ = 6.7 Hz, $^4J_{HH}$ = 6.7 Hz, 1H, H-C3, 1H), 4.65 (dt, $^4J_{HH}$ = 6.6 Hz, $^5J_{HH}$ = 3.2 Hz, 2H, H-C1), 3.41 (t, $^3J_{HH}$ = 6.9 Hz, 2H, H-C12), 2.05 – 1.94 (m, 2H, H-C4), 1.90 – 1.80 (m, 2H, H-C11), 1.49 – 1.36 (m, 4H, H-C5, H-C10), 1.35 – 1.25 (m, 8H, H-C6~C9).

^{13}C NMR (151 MHz, $CDCl_3$) δ 208.65 (C2), 90.21 (C3), 74.69 (C1), 34.18 (C12), 32.99 (C11), 29.52 (*C*H_2), 29.45 (*C*H_2), 29.24 (*C*H_2), 29.17 (C5), 28.89 (*C*H_2), 28.40 (C4), 28.32 (C10).

GC-EI-MS: (*m/z*) requires: $[(C_{12}H_{21}{}^{79}Br)]$ = 244.0827, (*m/z*) found: $[(C_{12}H_{21}{}^{79}Br)]$ = 244.0822.

FT-IR ($\tilde{\nu}$ = cm^{-1}): 2927 (s), 2852 (s), 1957 (w), 1457 (m), 1440 (m), 1262 (w), 837 (s), 723 (m), 648 (m).

4-((Dodeca-10,11-dien-1-yloxy)methyl)-1,2-difluorobenzene (S4)

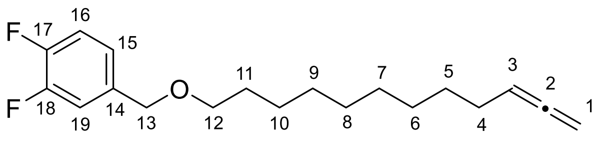

Under argon atmosphere, to a flame-dried Schlenk flask were added NaH (160.0 mg, 60% in mineral oil, 4.00 mmol, 2.0 eq.) and THF (4 mL). The mixture was cooled to 0 °C. Dodeca-10,11-dien-1-ol (**S6**) (364.6 mg, 2.00 mmol, dissolved in 1 mL THF, 1.0 eq.) was added dropwise. The reaction mixture was then stirred for 80 minutes. Tetrabutylammonium iodide (36.9 mg, 0.10 mmol, 5 mol%) and α-bromo-3,4-difluorotoluene (455.4 mg, 2.20 mmol, 1.1 eq.) were added. The cooling bath was removed and the reaction mixture was heated to 40 °C and stirred for 20 h. The reaction was cooled to room temperature. A saturated aqueous solution of NH_4Cl was added to quench the reaction. The aqueous layer was then extracted with diethyl ether and the combined organic layer was dried over anhydrous Na_2SO_4, filtered, and concentrated in vacuo. The residue was purified by column chromatography (*n*-pentane:Et_2O 60:1) to yield the title compound as a colorless oil (577.6 mg, 1.87 mmol, 94%).

$\mathbf{R_{\mathit{f}}}$ = 0.30 (*n*-pentane:Et_2O 60:1).

^{1}H NMR (599 MHz, $CDCl_3$) δ 7.20 – 7.08 (m, 2H, H-C16, H-C19), 7.06 – 7.00 (m, 1H, H-C15), 5.09 (p, $^{3}J_{HH}$ = 6.8 Hz, $^{4}J_{HH}$ = 6.8 Hz, 1H, H-C3), 4.64 (dt, $^{4}J_{HH}$ = 6.6 Hz, $^{5}J_{HH}$ = 3.2 Hz, 2H, H-C1), 4.43 (s, 2H, H-C13), 3.46 (t, $^{3}J_{HH}$ = 6.6 Hz, 2H, H-C12), 2.03 – 1.95 (m, 2H, H-C4), 1.65 – 1.58 (m, 2H, H-C11), 1.44 – 1.34 (m, 4H, H-C5, H-C10), 1.33 – 1.25 (m, 8H, H-C6~C9).

^{13}C NMR (151 MHz, $CDCl_3$) δ 208.65 (C2), 150.49 (dd, $^{1}J_{CF}$ = 248.6 Hz, $^{2}J_{CF}$ = 12.6 Hz, *C*F), 149.84 (dd, $^{1}J_{CF}$ = 247.1 Hz, $^{2}J_{CF}$ = 12.6 Hz, *C*F), 136.01 (dd, $^{3}J_{CF}$ = 5.2 Hz, $^{4}J_{CF}$ = 3.8 Hz, C14), 123.38 (dd, $^{3}J_{CF}$ = 6.3 Hz, $^{4}J_{CF}$ = 3.7 Hz, C15), 117.16 (d, $^{2}J_{CF}$ = 17.3 Hz, C19), 116.52 (d, $^{2}J_{CF}$ = 17.5 Hz, C16), 90.23 (C3), 74.66 (C1), 71.75 (d, $^{4}J_{CF}$ = 1.4 Hz, C13), 70.90 (C12), 29.85 (C11), 29.66 (*C*H_2), 29.57 (*C*H_2), 29.51 (*C*H_2), 29.26 (*C*H_2), 29.20 (*C*H_2), 28.41 (C4), 26.30 (C10).

^{19}F NMR (564 MHz, $CDCl_3$) δ -138.09 (dddd, $^{3}J_{FF}$ = 21.1 Hz, $^{3}J_{HF}$ = 11.1 Hz, $^{4}J_{HF}$ = 7.9 Hz, $^{5}J_{HF}$ = 1.4 Hz, 1F, F-C18), -139.87 – -140.05 (m, 1F, F-C17).

^{19}F{^{1}H} NMR (564 MHz, $CDCl_3$) δ -138.09 (d, $^{3}J_{FF}$ = 21.1 Hz, 1F, F-C18), -139.96 (d, $^{3}J_{FF}$ = 21.1 Hz, 1F, F-C17).

GC-EI-MS: (*m/z*) requires: [($C_{19}H_{25}OF_2$)] = 307.1873 [M-H], (*m/z*) found: [($C_{19}H_{25}OF_2$)] = 307.1867.

FT-IR ($\tilde{\nu}$ = cm^{-1}): 2927 (s), 2852 (m), 1957 (w), 1612 (w), 1521 (s), 1434 (m), 1360 (w), 1285 (s), 1211 (w), 1142 (m), 1102 (s), 843 (s), 815 (s), 774 (m), 746 (m).

Buta-2,3-dien-1-ylbenzene (S5)

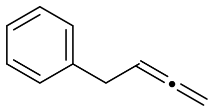

Compound **S5** was prepared according to General Procedure **A** using propargyl benzene (464.6 mg, 4.00 mmol, 1.0 eq.). The crude residue was purified by column chromatography (*n*-pentane) to yield the title compound as a colorless oil (335.1 mg, 2.57 mmol, 64%).

$\mathbf{R_f}$ = 0.80 (*n*-pentane).

^{1}H NMR (400 MHz, $CDCl_3$) δ 7.36 – 7.27 (m, 2H), 7.25 – 7.17 (m, 3H), 5.28 (p, *J* = 7.0 Hz, 1H), 4.72 (dt, *J* = 6.7, 2.9 Hz, 2H), 3.36 (dt, *J* = 7.4, 2.9 Hz, 2H).

GC-EI-MS: (*m/z*) requires: [($C_{10}H_{10}$)] = 130.0783, (*m/z*) found: [($C_{10}H_{10}$)] = 130.0776.

Analytical data is in agreement with literature values.[3]

Dodeca-10,11-dien-1-yl 4-methylbenzenesulfonate (S7)

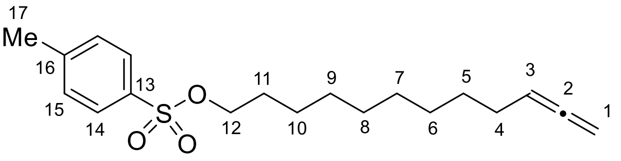

Under argon atmosphere, to a flame-dried Schlenk flask was added dodeca-10,11-dien-1-ol (**S6**) (350.0 mg, 1.92 mmol, 1.0 eq.) and DCM (3 mL). The mixture was cooled to 0 °C. DMAP (30.5 mg, 0.25 mmol, 13 mol%), NEt_3 (0.5 mL) and TsCl (576.8 mg, 3.03 mmol, 1.6 eq.) were added successively. The reaction mixture was warmed to room temperature and stirred for 24 h. After removal of the solvent, the residue was directly purified by column chromatography (*n*-pentane:Et_2O 10:1) to yield the title compound as a colorless oil (264.6 mg, 0.79 mmol, 41%).

$\mathbf{R_f}$ = 0.40 (*n*-pentane:Et_2O 10:1).

^{1}H NMR (500 MHz, $CDCl_3$) δ 7.81 – 7.76 (m, 2H, H-C14), 7.37 – 7.31 (m, 2H, H-C15), 5.08 (p, $^3J_{HH}$ = 6.8 Hz, $^4J_{HH}$ = 6.8 Hz, 1H, H-C3, 1H), 4.64 (dt, $^4J_{HH}$ = 6.6 Hz, $^5J_{HH}$ = 3.2 Hz, 2H, H-C1), 4.02 (t, $^3J_{HH}$ = 6.5 Hz, 2H, H-C12), 2.45 (s, 3H, H-C17), 2.02 – 1.94 (m, 2H, H-C4), 1.63 1.69 – 1.57 (m, 2H, H-C11), 1.45 – 1.34 (m, 2H, H-C5), 1.32 – 1.25 (m, 4H, H-C6, H-C10), 1.25 – 1.17 (m, 6H, H-C7~C9).

^{13}C NMR (126 MHz, $CDCl_3$) δ 208.64 (C2), 144.74 (C16), 133.45 (C13), 129.93 (C15), 128.03 (C14), 90.19 (C3), 74.69 (C1), 70.83 (C12), 29.45 (*C*H_2), 29.39 (*C*H_2), 29.21 (*C*H_2), 29.13 (*C*H_2), 29.03 (*C*H_2), 28.96 (C11), 28.37 (C4), 25.46 (*C*H_2), 21.77 (C17).

ESI-MS: (*m/z*) requires: [$(C_{19}H_{28}O_3SNa)^+$] = 359.1651, (*m/z*) found: [$(C_{19}H_{28}O_3SNa)^+$] = 359.1653.

FT-IR ($\tilde{\nu}$ = cm^{-1}): 2927 (m), 2852 (w), 1457 (w), 1360 (s), 1176 (s), 1096 (w), 952 (m), 837 (m), 815 (m), 665 (m).

2-(Dodeca-10,11-dien-1-yl)isoindoline-1,3-dione (S8)

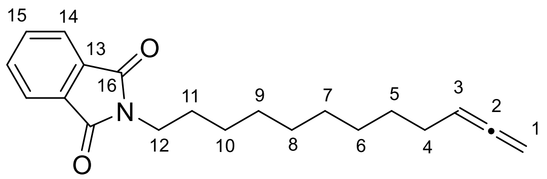

Under argon atmosphere, to a flame-dried round bottom flask equipped with a magnetic stir bar was added phthalimide (565.7 mg, 3.84 mmol, 2.1 equiv), dodeca-10,11-dien-1-ol (**S6**) (336.3 mg, 1.85 mmol, 1.0 eq.), triphenylphosphine (972.3 mg, 3.71 mmol, 2.0 equiv) and Et_2O (5 mL). The reaction solution was cooled to 0 °C and diethylazodicarboxylate (1.5 mL, 40% in PhMe) was added slowly. The reaction was gradually warmed to room temperature and stirred overnight. After removal of the solvent, the residue was directly purified by column chromatography (*n*-pentane:Et_2O 9:1) to yield the title compound as a pink oil (436.4 mg, 1.40 mmol, 76%).

R_f = 0.30 (*n*-pentane:Et_2O 9:1).

^{1}H NMR (500 MHz, $CDCl_3$) δ 7.83 (dd, $^3J_{HH}$ = 5.4 Hz, $^4J_{HH}$ = 3.0 Hz, 2H, H-C14), 7.74 – 7.65 (m, 2H, H-C15), 5.07 (p, $^3J_{HH}$ = 6.8 Hz, $^4J_{HH}$ = 6.8 Hz, 1H, H-C3, 1H), 4.63 (dt, $^4J_{HH}$ = 6.6 Hz, $^5J_{HH}$ = 3.2 Hz, 2H, H-C1), 3.71 – 3.62 (m, 2H, H-C12), 2.04 – 1.92 (m, 2H, H-C4), 1.72 – 1.61 (m, 2H, H-C11), 1.38 (p, $^3J_{HH}$ = 6.6 Hz, 2H, H-C5), 1.34 – 1.30 (m, 4H, H-C6, H-C10), 1.29 – 1.24 (m, 6H, H-C7~C9).

^{13}C NMR (126 MHz, $CDCl_3$) δ 208.63 (C2), 168.60 (C16), 133.96 (C15), 132.35 (C13), 123.28 (C14), 90.22 (C3), 74.65 (C1), 38.21 (C12), 29.55 (*C*H_2), 29.46 (*C*H_2), 29.29 (*C*H_2), 29.23 (*C*H_2), 29.15 (*C*H_2), 28.73 (C11), 28.39 (C4), 26.99 (C10).

ESI-MS: (*m/z*) requires: [$(C_{20}H_{25}O_2NNa)^+$] = 334.1777, (*m/z*) found: [$(C_{20}H_{25}O_2NNa)^+$] = 334.1778.

FT-IR ($\tilde{\nu}$ = cm^{-1}): 2927 (m), 2852 (w), 1773 (w), 1710 (s), 1463 (w), 1440 (m), 1394 (m), 1366 (m), 1188 (w), 1067 (m), 1033 (w), 906 (s), 843 (m), 717 (s), 648 (m).

Dodeca-10,11-dien-1-yl nicotinate (S9)

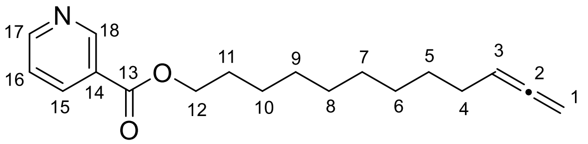

To a solution of nicotinic acid (135.4 mg, 1.10 mmol, 1.1 eq.) in DCM (2 mL) were added 1-ethyl-3-(3-(dimethylamino)propyl)-carbodiimide hydrochloride (EDCI) (287.6 mg, 1.50 mmol, 1.5 eq.), *N*,*N*-dimethylaminopyridine (12.2 mg, 0.10 mmol, 10 mol %) and then dodeca-10,11-dien-1-ol (**S6**) (182.3 mg, 1.00 mmol, 1.0 eq.). The reaction mixture was stirred at room temperature for 12 h. DCM was added. The mixture was washed with brine and dried over Na_2SO_4. After removal of the solvent, the residue was purified by column chromatography (*n*-pentane:Et_2O 2.5:1-2:1) to yield the title compound as a pale yellow oil (236.1 mg, 0.82 mmol, 82%).

$\mathbf{R}_{\boldsymbol{f}}$ = 0.35 (*n*-pentane:Et_2O 2.5:1).

^{1}H NMR (500 MHz, $CDCl_3$) δ 9.23 (d, $^4J_{HH}$ = 2.2 Hz, 1H, H-C18), 8.77 (dd, $^3J_{HH}$ = 4.8 Hz, $^4J_{HH}$ = 1.8 Hz, 1H, H-C17), 8.29 (dt, $^3J_{HH}$ = 7.9 Hz, $^4J_{HH}$ = 2.0 Hz, 1H, H-C15), 7.39 (dd, $^3J_{HH}$ = 7.9 Hz, 4.8 Hz, 1H, H-16), 5.08 (p, $^3J_{HH}$ = 6.8 Hz, $^4J_{HH}$ = 6.8 Hz, 1H, H-C3), 4.64 (dt, $^4J_{HH}$ = 6.6 Hz, $^5J_{HH}$ = 3.2 Hz, 2H, H-C1), 4.35 (t, $^3J_{HH}$ = 6.7 Hz, 2H, H-C12), 2.03 – 1.94 (m, 2H, H-C4), 1.82 – 1.73 (m, 2H, H-C11), 1.48 – 1.38 (m, 4H, H-C5, H-C10), 1.38 – 1.26 (m, 8H, H-C6~C9).

^{13}C NMR (126 MHz, $CDCl_3$) δ 208.64 (C2), 165.50 (C13), 153.46 (C17), 151.06 (C18), 137.16 (C15), 126.53 (C14), 123.41 (C16), 90.21 (C3), 74.68 (C1), 65.75 (C12), 29.59 (CH$_2$), 29.47 (CH$_2$), 29.37 (C9), 29.23 (C5), 29.17 (CH$_2$), 28.79 (C11), 28.39 (C4), 26.12 (C10).

ESI-MS: (*m/z*) requires: [$(C_{18}H_{25}O_2NNa)^+$] = 310.1777, (*m/z*) found: [$(C_{18}H_{25}O_2NNa)^+$] = 310.1778.

FT-IR ($\tilde{\nu}$ = cm^{-1}): 2927 (m), 2852 (w), 1957 (w), 1721 (s), 1589 (w), 1463 (w), 1423 (w), 1279 (s), 1113 (s), 1021 (m), 843 (m), 740 (s), 700 (m).

Dodeca-10,11-dien-1-yl 2-methylthiazole-4-carboxylate (S10)

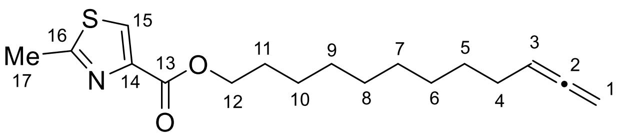

To a solution of 2-methylthiazole-4-carboxylic acid (157.5 mg, 1.10 mmol, 1.1 eq.) in DCM (2 mL) were added 1-ethyl-3-(3-(dimethylamino)propyl)-carbodiimide hydrochloride (EDCI) (287.6 mg, 1.50 mmol, 1.5 eq.), *N*,*N*-dimethylaminopyridine (12.2 mg, 0.10 mmol, 10 mol %) and then dodeca-10,11-dien-1-ol (**S6**) (182.3 mg, 1.00 mmol, 1.0 eq.). The reaction mixture was stirred at room temperature for 12 h. DCM was added. The mixture was washed with brine and dried over Na_2SO_4. After removal of the

solvent, the residue was purified by column chromatography (*n*-pentane:Et_2O 2.5:1-2:1) to yield the title compound as a white wax (229.8 mg, 0.75 mmol, 75%).

R*f* = 0.30 (*n*-pentane:Et_2O 2.5:1).

^{1}H NMR (500 MHz, $CDCl_3$) δ 8.01 (s, 1H, H-C15), 5.08 (p, $^3J_{HH}$ = 6.8 Hz, $^4J_{HH}$ = 6.8 Hz, 1H, H-C3), 4.64 (dt, $^4J_{HH}$ = 6.6 Hz, $^5J_{HH}$ = 3.2 Hz, 2H, H-C1), 4.34 (t, $^3J_{HH}$ = 6.9 Hz, 2H, H-C12), 2.76 (s, 3H, H-C17), 2.03 – 1.93 (m, 2H, H-C4), 1.82 – 1.70 (m, 2H, H-C11), 1.44 – 1.36 (m, 4H, H-C5, H-C10), 1.34 – 1.27 (m, 8H, H-C6~C9).

^{13}C NMR (126 MHz, $CDCl_3$) δ 208.63 (C2), 166.88 (C16), 161.59 (C13), 147.06 (C14), 127.26 (C15), 90.21 (C3), 74.67 (C1), 65.68 (C12), 29.57 (*C*H_2), 29.46 (*C*H_2), 29.37 (C9), 29.23 (C5), 29.16 (*C*H_2), 28.85 (C11), 28.38 (C4), 26.01 (C10), 19.51 (C17).

ESI-MS: (*m/z*) requires: [$(C_{17}H_{25}O_2NSNa)^+$] = 330.1498, (*m/z*) found: [$(C_{17}H_{25}O_2NSNa)^+$] = 330.1493.

FT-IR ($\tilde{\nu}$ = cm^{-1}): 2927 (m), 2852 (m), 1957 (w), 1716 (s), 1486 (m), 1320 (m), 1205 (s), 1170 (s), 1096 (m), 964 (w), 912 (w), 843 (m), 751 (s), 728 (s), 619 (w).

Dodeca-10,11-dien-1-yl cyclopropanecarboxylate (S11)

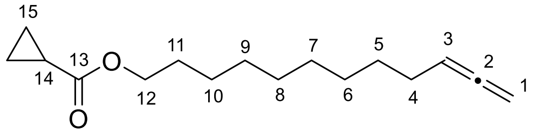

To a solution of cyclopropylcarboxylic acid (129.1 mg, 1.50 mmol, 1.5 eq.) in DCM (2 mL) were added 1-ethyl-3-(3-(dimethylamino)propyl)-carbodiimide hydrochloride (EDCI) (287.6 mg, 1.50 mmol, 1.5 eq.), *N*,*N*-dimethylaminopyridine (12.2 mg, 0.10 mmol, 10 mol %) and then dodeca-10,11-dien-1-ol (**S6**) (182.3 mg, 1.00 mmol, 1.0 eq.). The reaction mixture was stirred at room temperature for 12 h. DCM was added. The mixture was washed with brine and dried over Na_2SO_4. After removal of the solvent, the residue was purified by column chromatography (*n*-pentane:Et_2O 40:1-30:1) to yield the title compound as a colorless oil (209.9 mg, 0.84 mmol, 84%).

R*f* = 0.35 (*n*-pentane:Et_2O 30:1).

^{1}H NMR (599 MHz, $CDCl_3$) δ 5.09 (p, $^3J_{HH}$ = 6.8 Hz, $^4J_{HH}$ = 6.8 Hz, 1H, H-C3), 4.64 (dt, $^4J_{HH}$ = 6.6 Hz, $^5J_{HH}$ = 3.2 Hz, 2H, H-C1), 4.06 (t, $^3J_{HH}$ = 6.8 Hz, 2H, H-C12), 2.04 – 1.95 (m, 2H, H-C4), 1.65 – 1.56 (m, 3H, H-C14, H-C11), 1.43 – 1.37 (m, 2H, H-C5), 1.37 – 1.26 (m, 10H, H-C6~C10), 1.00 – 0.96 (m, 2H, H-C15), 0.86 – 0.82 (m, 2H, H-C15).

^{13}C NMR (151 MHz, $CDCl_3$) δ 208.65 (C2), 175.13 (C13), 90.22 (C3), 74.67 (C1), 64.77 (C12), 29.60 (*C*H_2), 29.47 (*C*H_2), 29.37 (*C*H_2), 29.25 (C5), 29.18 (C6), 28.83 (C11), 28.40 (C4), 26.05 (C10), 13.06 (C14), 8.42 (C15).
ESI-MS: (*m/z*) requires: [$(C_{16}H_{26}O_2Na)^+$] = 273.1825, (*m/z*) found: [$(C_{16}H_{26}O_2Na)^+$] = 273.1824.
FT-IR ($\tilde{\nu}$ = cm^{-1}): 2927 (m), 2852 (m), 1727 (s), 1457 (w), 1400 (m), 1371 (m), 1268 (w), 1170 (s), 1073 (m), 1033 (w), 843 (m), 746 (w),

Propa-1,2-dien-1-ylcyclohexane (S12)

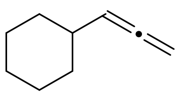

This compound is commercially available.

(8R,9S,13S,14S)-13-Methyl-17-oxo-7,8,9,11,12,13,14,15,16,17-decahydro-6H-cyclopenta[a]phenanthren-3-yl dodeca-10,11-dienoate (S13)

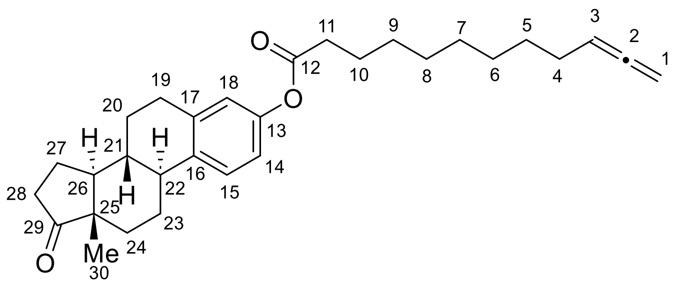

To a solution of undec-10-ynoic acid (364.5 mg, 2.00 mmol, 1.0 eq.) in DCM (4 mL) were added 1-ethyl-3-(3-(dimethylamino)propyl)-carbodiimide hydrochloride (EDCI) (575.1 mg, 3.00 mmol, 1.5 eq.), *N*,*N*-dimethylaminopyridine (24.4 mg, 0.20 mmol, 10 mol %) and then estrone (594.8 mg, 2.20 mmol, 1.1 eq.). The reaction mixture was stirred at room temperature for 12 h. DCM was added. The mixture was washed with brine and dried over Na_2SO_4. After removal of the solvent, the residue was filtrated through a small pad of silica gel (pentane:Et_2O = 2:1). The solvent was removed under reduced pressure with the aid of a rotary evaporator to give the crude (8R,9S,13S,14S)-13-methyl-17-oxo-7,8,9,11,12,13,14,15,16,17-decahydro-6H-cyclopenta[a]phenanthren-3-yl undec-10-ynoate, which was used without any further purification for the next step.
Compound **S13** was prepared according to General Procedure **A** using crude (8R,9S,13S,14S)-13-methyl-17-oxo-7,8,9,11,12,13,14,15,16,17-decahydro-6H-cyclopenta[a]phenanthren-3-yl undec-10-ynoate (408.5 mg, 0.94 mmol, 1.0 eq.). The crude residue was purified by column chromatography (*n*-pentane: EA 7.5:1-6:1) to yield the title compound as a white solid (282.3 mg, 0.63 mmol, 67%).
R*f* = 0.40 (*n*-pentane:EA 6:1).

^{1}H NMR (599 MHz, $CDCl_3$) δ 7.28 (d, $^3J_{HH}$ = 8.6 Hz, 1H, H-C15), 6.84 (dd, $^3J_{HH}$ = 8.5 Hz, $^4J_{HH}$ = 2.6 Hz, 1H, H-C14), 6.80 (d, $^4J_{HH}$ = 2.5 Hz, 1H, H-C18), 5.09 (p, $^3J_{HH}$ = 6.8 Hz, $^4J_{HH}$ = 6.8 Hz, 1H, H-C3), 4.65 (dt, $^4J_{HH}$ = 6.6 Hz, $^5J_{HH}$ = 3.2 Hz, 2H, H-C1), 2.95 – 2.88 (m, 2H, H-C19), 2.55 – 2.47 (m, 3H, H-C11, H-C28), 2.43 – 2.38 (m, 1H, H-C23), 2.29 (td, $^3J_{HH}$ = 10.8, 4.2 Hz, 1H, H-C22), 2.19 – 2.11 (m, 1H, H-C28), 2.08 – 1.94 (m, 5H, H-C4, H-C20, H-C24, H-C27), 1.74 (p, $^3J_{HH}$ = 7.5 Hz, 2H, H-C10), 1.67 – 1.59 (m, 2H, H-C21, H-C27), 1.58 – 1.44 (m, 4H, H-C20, H-C23, H-C24, H-C26), 1.44 – 1.38 (m, 4H, H-C5, H-C9), 1.37 – 1.30 (m, 6H, H-C6~C8), 0.91 (s, 3H, H-C30).

^{13}C NMR (151 MHz, $CDCl_3$) δ 220.86 (C29), 208.64 (C2), 172.73 (C12), 148.79 (C13), 138.09 (C16), 137.39 (C17), 126.50 (C15), 121.72 (C18), 118.90 (C14), 90.19 (C3), 74.69 (C1), 50.59 (C26), 48.08 (C25), 44.30 (C22), 38.16 (C21), 35.99 (C28), 34.55 (C11), 31.70 (C24), 29.53 (C19), 29.35 (CH$_2$), 29.33 (CH$_2$), 29.21 (CH$_2$), 29.12 (CH$_2$), 28.37 (C4), 26.49 (C20), 25.90 (C23), 25.11 (C10), 21.73 (C27), 13.97 (C30).

ESI-MS: (*m/z*) requires: [$(C_{30}H_{40}O_3Na)^+$] = 471.2870, (*m/z*) found: [$(C_{30}H_{40}O_3Na)^+$] = 471.2866.

FT-IR ($\tilde{\nu}$ = cm^{-1}): 2927 (m), 2858 (w), 1951 (w), 1739 (s), 1492 (w), 1457 (w), 1371 (w), 1222 (m), 1153 (m), 1084 (m), 1056 (w), 1010 (w), 906 (m), 843 (m), 728 (s), 648 (w).

Melting Point: 54-55.4 ˚C.

Dodeca-10,11-dien-1-yl 2-(4-isobutylphenyl)propanoate (S14)

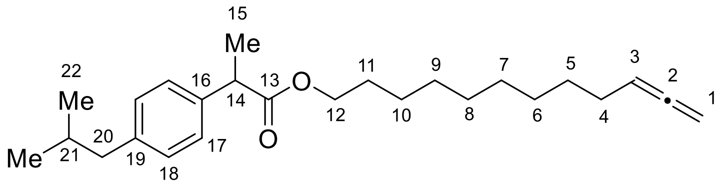

To a solution of ibuprofen (309.4 mg, 1.50 mmol, 1.5 eq.) in DCM (2 mL) were added 1-ethyl-3-(3-(dimethylamino)propyl)-carbodiimide hydrochloride (EDCI) (287.6 mg, 1.50 mmol, 1.5 eq.), *N*,*N*-dimethylaminopyridine (12.2 mg, 0.10 mmol, 10 mol %) and then dodeca-10,11-dien-1-ol (**S6**) (182.3 mg, 1.00 mmol, 1.0 eq.). The reaction mixture was stirred at room temperature for 12 h. DCM was added. The mixture was washed with brine and dried over Na_2SO_4. After removal of the solvent, the residue was purified by column chromatography (*n*-pentane:Et_2O 40:1) to yield the title compound as a colorless oil (307.6 mg, 0.83 mmol, 83%).

R*f* = 0.29 (*n*-pentane:Et_2O 40:1).

^{1}H NMR (599 MHz, $CDCl_3$) δ 7.22 – 7.18 (m, 2H, H-C18), 7.10 – 7.07 (m, 2H, H-C17), 5.09 (p, $^3J_{HH}$ = 6.8 Hz, $^4J_{HH}$ = 6.8 Hz, 1H, H-C3), 4.65 (dt, $^4J_{HH}$ = 6.6 Hz, $^5J_{HH}$ = 3.2 Hz, 2H, H-C1), 4.05

(td, $^3J_{HH}$ = 6.7 Hz, $^5J_{HH}$ = 0.9 Hz, 2H, H-C12), 3.68 (q, $^3J_{HH}$ = 7.2 Hz, 1H, H-C14), 2.44 (d, $^3J_{HH}$ = 7.2 Hz, 2H, H-C20), 2.03 – 1.95 (m, 2H, H-C4), 1.89 – 1.80 (m, 1H, H-C21), 1.60 – 1.53 (m, 2H, H-C11), 1.48 (d, $^3J_{HH}$ = 7.2 Hz, 3H, H-C15), 1.43 – 1.36 (m, 2H, H-C5), 1.34 – 1.27 (m, 2H, H-C6), 1.27 – 1.18 (m, 8H, H-C7~C10), 0.90 (d, $^3J_{HH}$ = 6.6 Hz, 6H, H-C22).

^{13}C NMR (151 MHz, $CDCl_3$) δ 208.65 (C2), 174.97 (C13), 140.56 (C19), 138.07 (C16), 129.40 (C18), 127.30 (C17), 90.22 (C3), 74.68 (C1), 64.91 (C12), 45.37 (C14), 45.19 (C20), 30.33 (C21), 29.57 (*C*H_2), 29.46 (*C*H_2), 29.28 (*C*H_2), 29.26 (C5), 29.19 (C6), 28.68 (C11), 28.41 (C4), 25.91 (C10), 22.53 (C22), 18.61 (C15).

ESI-MS: (*m/z*) requires: [$(C_{25}H_{38}O_2Na)^+$] = 393.2764, (*m/z*) found: [$(C_{25}H_{38}O_2Na)^+$] = 393.2761.

FT-IR ($\tilde{\nu}$ = cm^{-1}): 2927 (s), 2858 (m), 1733 (s), 1509 (w), 1457 (m), 1383 (w), 1331 (w), 1205 (m), 1165 (s), 1073 (m), 912 (w), 843 (s), 757 (m), 734 (s).

Dodeca-10,11-dien-1-yl (E)-3-(4-bromophenyl)acrylate (S15)

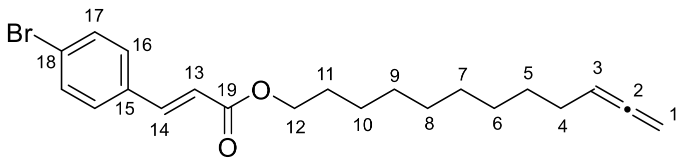

To a solution of 4-bromocinnamic acid (374.6 mg, 1.65 mmol, 1.1 eq.) in DCM (3 mL) were added 1-ethyl-3-(3-(dimethylamino)propyl)-carbodiimide hydrochloride (EDCI) (431.3 mg, 2.25 mmol, 1.5 eq.), *N*,*N*-dimethylaminopyridine (18.3 mg, 0.15 mmol, 10 mol%) and then dodeca-10,11-dien-1-ol (**S6**) (273.5 mg, 1.50 mmol, 1.0 eq.). The reaction mixture was stirred at room temperature for 12 h. DCM was added. The mixture was washed with brine and dried over Na_2SO_4. After removal of the solvent, the residue was purified by column chromatography (*n*-pentane:Et_2O 20:1) to yield the title compound as a white solid (319.6 mg, 0.82 mmol, 54%).

R_f = 0.35 (*n*-pentane:Et_2O 20:1).

^{1}H NMR (400 MHz, $CDCl_3$) δ 7.61 (d, $^3J_{HH}$ = 16.0 Hz, 1H, H-C13), 7.52 (d, $^3J_{HH}$ = 8.4 Hz, 2H, H-C16), 7.39 (d, $^3J_{HH}$ = 8.4 Hz, 2H, H-C17), 6.42 (d, $^3J_{HH}$ = 16.0 Hz, 1H, H-C14), 5.09 (p, $^3J_{HH}$ = 6.8 Hz, $^4J_{HH}$ = 6.8 Hz, 1H, H-C3), 4.64 (dt, $^4J_{HH}$ = 6.5 Hz, $^5J_{HH}$ = 3.2 Hz, 2H, H-C1), 4.20 (t, $^3J_{HH}$ = 6.7 Hz, 2H, H-C12), 2.02 – 1.96 (m, 2H, H-C4), 1.69 (p, $^3J_{HH}$ = 6.9 Hz, 2H, H-C11), 1.39 (p, $^3J_{HH}$ = 7.4 Hz, 4H, H-C5, H-C10), 1.36 – 1.25 (m, 8H, H-C6~C9).

^{13}C NMR (151 MHz, $CDCl_3$) δ 208.64 (C2), 166.96 (C19), 143.30 (C13), 133.56 (C15), 132.27 (C16), 129.57 (C17), 124.59 (C18), 119.17 (C14), 90.22 (C3), 74.68 (C1), 65.01 (C12), 29.60 (*C*H_2), 29.48 (*C*H_2), 29.38 (C9), 29.25 (C5), 29.18 (*C*H_2), 28.85 (C11), 28.40 (C4), 26.10 (C10).

ESI-MS: (*m/z*) requires: $[(C_{21}H_{27}O_2{}^{79}BrNa)^+]$ = 413.1087, (*m/z*) found: $[(C_{21}H_{27}O_2{}^{79}BrNa)^+]$ = 413.1087.

FT-IR ($\tilde{\nu}$ = cm^{-1}): 2921 (m), 2852 (w), 1704 (m), 1635 (m), 1486 (w), 1463 (w), 1400 (w), 1308 (m), 1165 (s), 1067 (m), 1004 (s), 815 (s), 723 (m).

Melting Point: 39-40 ˚C.

1-(Propa-1,2-dien-1-yl)-4-(trifluoromethyl)benzene (S16)

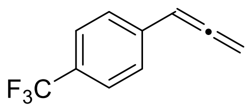

Compound **S16** was prepared according to General Procedure **A** using 4-trifluoromethylphenylacetylene (680.5 mg, 4.00 mmol, 1.0 eq.). The crude residue was purified by column chromatography (*n*-pentane) to yield the title compound as a colorless oil (183.4 mg, 1.00 mmol, 25%).

$\mathbf{R}_f$ = 0.85 (*n*-pentane).

^{1}**H NMR** (400 MHz, $CDCl_3$) δ 7.55 (d, *J* = 8.1 Hz, 2H), 7.39 (d, *J* = 8.0 Hz, 2H), 6.19 (t, *J* = 6.8 Hz, 1H), 5.21 (d, *J* = 6.8 Hz, 2H).

^{19}**F{^{1}H} NMR** (377 MHz, $CDCl_3$) δ -62.44 (s, 3F).

GC-EI-MS: (*m/z*) requires: $[(C_{10}H_7F_3)]$ = 184.0500, (*m/z*) found: $[(C_{10}H_7F_3)]$ = 184.0492.

Analytical data is in agreement with literature values.[5]

1-(Propa-1,2-dien-1-yl)-3,5-bis(trifluoromethyl)benzene (S17)

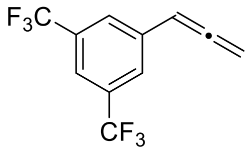

Compound **S17** was prepared according to General Procedure **A** using 3,5-bis(trifluoromethyl)phenylacetylene (952.5 mg, 4.00 mmol, 1.0 eq.). The crude residue was purified by column chromatography (*n*-pentane) to yield the title compound as a white wax (156.1 mg, 0.62 mmol, 15%).

$\mathbf{R}_f$ = 0.85 (*n*-pentane).

^{1}**H NMR** (599 MHz, $CDCl_3$) δ 7.73 – 7.65 (m, 3H), 6.24 (t, *J* = 6.8 Hz, 1H), 5.31 (d, *J* = 6.8 Hz, 2H).

^{19}**F{^{1}H} NMR** (564 MHz, $CDCl_3$) δ -63.07 (s, 6F).

GC-EI-MS: (*m/z*) requires: $[(C_{11}H_6F_6)]$ = 252.0374, (*m/z*) found: $[(C_{11}H_6F_6)]$ = 252.0369.

Analytical data is in agreement with literature values.[6]

Methyl 4-(propa-1,2-dien-1-yl)benzoate (S18)

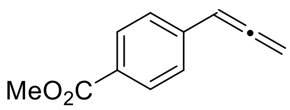

Compound **S18** was prepared according to General Procedure **A** using 4-ethynylbenzoic acid methyl ester (1.28 g, 8.00 mmol, 1.0 eq.). The crude residue was purified by column chromatography (*n*-pentane: Et_2O 10:1) to yield the title compound as a pale yellow solid (730.7 mg, 4.19 mmol, 52%).

$\mathbf{R_f}$ = 0.39 (*n*-pentane: Et_2O 10:1).

^{1}H NMR (400 MHz, $CDCl_3$) δ 8.04 – 7.93 (m, 2H), 7.40 – 7.32 (m, 2H), 6.20 (t, *J* = 6.8 Hz, 1H), 5.21 (d, *J* = 6.8 Hz, 2H), 3.91 (s, 3H).

GC-EI-MS: (*m/z*) requires: [($C_{11}H_{10}O_2$)] = 174.0681, (*m/z*) found: [($C_{11}H_{10}O_2$)] = 174.0674.

Analytical data is in agreement with literature values.[7]

Morpholino(4-(propa-1,2-dien-1-yl)phenyl)methanone (S19)

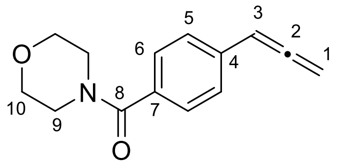

A mixture of methyl 4-(propa-1,2-dien-1-yl)benzoate (**S18**) (358.4 mg, 2.06 mmol, 1.0 eq.) and LiOH (295.6 mg, 12.34 mmol, 6.0 eq.) in THF (6 mL) and H_2O (6 mL) was vigorously stirred at rt for 8 h. After the reaction was quenched with saturated aqueous NH_4Cl, the water layer was acidified with concentrated HCl (pH = 3) and extracted with DCM. The organic layer was dried with Na_2SO_4, concentrated and the crude 4-(propa-1,2-dien-1-yl)benzoic acid was directly used in next step without further purification.

To a solution of crude 4-(propa-1,2-dien-1-yl)benzoic acid (160.2 mg, 1.00 mmol, 1.0 equiv.) in DCM (2 mL) was added 1-ethyl-3-(3-(dimethylamino)propyl)-carbodiimide hydrochloride (EDCI) (287.6 mg, 1.50 mmol, 1.5 eq.), *N*,*N*-dimethylaminopyridine (12.2 mg, 0.10 mmol, 10 mol%) and then morpholine (130.7 mg, 1.50 mmol, 1.5 eq.). The reaction mixture was stirred at room temperature for 12 h, before DCM was added. The mixture was washed with brine and dried over Na_2SO_4. After removal of the solvent, the residue was purified by column chromatography (*n*-pentane:EA 1:1.5) to yield the title compound as a pale yellow oil (141.9 mg, 0.62 mmol, 62%).

$\mathbf{R_f}$ = 0.30 (*n*-pentane:EA 1:1.5).

^{1}H NMR (400 MHz, $CDCl_3$) δ 7.37 – 7.30 (m, 4H, H-C5, H-C6), 6.17 (t, $^4J_{HH}$ = 6.8 Hz, 1H, H-C3), 5.18 (d, $^4J_{HH}$ = 6.7 Hz, 2H, H-C1), 3.79 – 3.54 (m, 8H, H-C9, H-C10).

^{13}C NMR (151 MHz, $CDCl_3$) δ 210.37 (C2), 170.41 (C8), 136.11 (C4), 133.66 (C7), 127.79(C6), 126.83 (C5), 93.58 (C3), 79.32 (C1), 67.03 (C9, C10).

ESI-MS: (*m/z*) requires: $[(C_{14}H_{15}NO_2Na)^+]$ = 252.0995, (*m/z*) found: $[(C_{14}H_{15}NO_2Na)^+]$ = 252.0993.

FT-IR ($\tilde{\nu}$ = cm^{-1}): 2973 (w), 2881 (w), 1618 (m), 1434 (m), 1279 (m), 1113 (m), 1084 (s), 1044 (s), 878 (s), 843 (w).

3-Ethynylphenyl trifluoromethanesulfonate (Int 1)

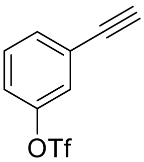

To a flame dried Schlenk flask was added 3-hydroxy phenylacetylene (590.7 mg, 5.0 mmol, 1.0 eq.) and pyridine (5 ml) under argon. The solution was cooled to 0 °C and triflic anhydride (0.93 ml, 5.5 mmol, 1.1 eq.) was added dropwise. The mixture was allowed to slowly warm to ambient temperature and was stirred for 16 h. The reaction mixture was diluted with diethyl ether and was washed with aqueous saturated solution of CuSO4 (3 x 50 ml) and water (1 x 50 ml). The organic phase was dried over Na_2SO_4 and the solvent was removed. The residue was purified by column chromatography (pentane) and the product (1.09 g, 4.36 mmol, 87%) was obtained as a colorless oil.

$\mathbf{R}_{\boldsymbol{f}}$ = 0.20 (*n*-pentane).

^{1}H NMR (400 MHz, $CDCl_3$) δ 7.51 (dt, *J* = 7.7, 1.3 Hz, 1H), 7.45 – 7.38 (m, 2H), 7.30 – 7.26 (m, 1H), 3.18 (s, 1H).

^{19}F{^{1}H} NMR (377 MHz, $CDCl_3$) δ -72.78 (s, 3F).

ESI-MS: (*m/z*) requires: $[(C_9H_5O_3F_3SNa)^+]$ = 272.9804, (*m/z*) found: $[(C_9H_5O_3F_3SNa)^+]$ = 272.9808.

Analytical data is in agreement with literature values.[8]

4-((Dodeca-10,11-dien-1-yloxy)methyl)-1,2-difluorobenzene (S20)

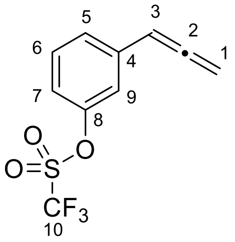

Compound **S20** was prepared according to General Procedure **A** using 3-ethynylphenyl trifluoromethanesulfonate (**Int 1**) (875.7 mg, 3.50 mmol, 1.0 eq.). The crude residue was purified by column chromatography (*n*-pentane: Et_2O 1:0-30:1) to yield the title compound as a colorless oil (122.0 mg, 0.46 mmol, 13%).

$\mathbf{R}_{\boldsymbol{f}}$ = 0.40 (*n*-pentane:Et_2O 30:1).

^{1}H NMR (599 MHz, $CDCl_3$) δ 7.37 (t, $^3J_{HH}$ = 8.0 Hz, 1H, H-C6), 7.30 (d, $^3J_{HH}$ = 7.8 Hz, 1H, H-C5), 7.19 (t, $^4J_{HH}$ = 2.1 Hz, 1H, H-C9), 7.09 (dd, $^3J_{HH}$ = 8.2 Hz, $^4J_{HH}$ = 2.5 Hz, 1H, H-C7), 6.16 (t, $^4J_{HH}$ = 6.8 Hz, 1H, H-C3), 5.23 (d, $^4J_{HH}$ = 6.8 Hz, 2H, H-C1).

^{13}**C NMR** (151 MHz, $CDCl_3$) δ 210.26 (C2), 150.15 (C8), 137.32 (C4), 130.40 (C6), 126.68 (C5), 119.51 (C7), 119.30 (C9), 118.88 (app. q, $^{1}J_{CF}$ = 321.1 Hz, C10), 93.03 (C3), 79.93 (C1).

^{19}**F NMR** (564 MHz, $CDCl_3$) δ -72.95 (s, 3F, F-C10).

^{19}**F{^{1}H} NMR** (564 MHz, $CDCl_3$) δ -72.95 (s, 3F, F-C10).

GC-EI-MS: (*m/z*) requires: [($C_{10}H_7F_3O_3S$)] = 264.0068, (*m/z*) found: [($C_{10}H_7F_3O_3S$)] = 264.0062.

FT-IR ($\tilde{\nu}$ = cm^{-1}): 1945 (w), 1612 (w), 1578 (w), 1486 (w), 1417 (m), 1245 (m), 1205 (s), 1136 (s), 1119 (s), 947 (m), 924 (m), 883 (m), 832 (s), 786 (m), 734 (w), 688 (m), 660 (w).

1-(Methylsulfonyl)-4-(propa-1,2-dien-1-yl)benzene (S21)

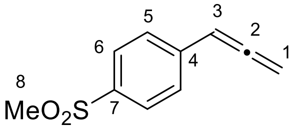

Compound **S21** was prepared according to General Procedure **A** using 1-ethynyl-4-(methylsulfonyl)benzene (682.0 mg, 3.78 mmol, 1.0 eq.). The crude residue was purified by column chromatography (*n*-pentane:EA 10:1) to yield the title compound as a light yellow solid (209.1 mg, 1.08 mmol, 29%).

R_f = 0.20 (*n*-pentane:EA 10:1).

^{1}**H NMR** (599 MHz, $CDCl_3$) δ 7.88 – 7.84 (m, 2H, H-C6), 7.48 – 7.44 (m, 2H, H-C5), 6.21 (t, $^{4}J_{HH}$ = 6.7 Hz, 1H, H-C3), 5.25 (d, $^{4}J_{HH}$ = 6.7 Hz, 2H, H-C1), 3.04 (s, 3H, H-C8).

^{13}**C NMR** (151 MHz, $CDCl_3$) δ 211.06 (C2), 140.40 (C4), 138.60 (C7), 127.88 (C6), 127.43 (C5), 93.25 (C3), 79.81 (C1), 44.72 (C8).

ESI-MS: (*m/z*) requires: [($C_{10}H_{10}O_2SNa$)$^+$] = 217.0294, (*m/z*) found: [($C_{10}H_{10}O_2SNa$)$^+$] = 217.0293.

FT-IR ($\tilde{\nu}$ = cm^{-1}): 1940 (w), 1595 (w), 1308 (m), 1268 (m), 1147 (s), 1090 (w), 958 (m), 906 (m), 860 (m), 728 (s).

Melting Point: 111-112 ˚C.

(1R,2S,5R)-2-Isopropyl-5-methylcyclohexyl 4-(propa-1,2-dien-1-yl)benzoate (S22)

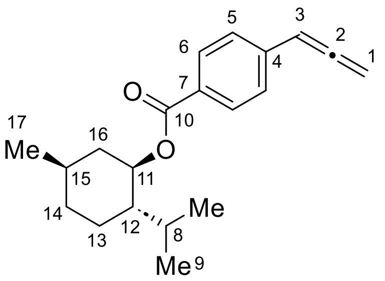

A mixture of methyl 4-(propa-1,2-dien-1-yl)benzoate (**S18**) (358.4 mg, 2.06 mmol, 1.0 eq.) and LiOH (295.6 mg, 12.34 mmol, 6.0 eq.) in THF (6 mL) and H_2O (6 mL) was vigorously stirred at rt for 8 h. After the reaction was quenched with saturated aqueous NH_4Cl, the water layer was acidified with concentrated HCl (pH = 3) and extracted with DCM. The organic layer was dried with Na_2SO_4, concentrated and the crude 4-(propa-1,2-dien-1-yl)benzoic acid was directly used in next step without further purification.

To a solution of crude 4-(propa-1,2-dien-1-yl)benzoic acid (160.2 mg, 1.00 mmol, 1.0 eq.) in DCM (5 mL) was added 1-ethyl-3-(3-(dimethylamino)propyl)-carbodiimide hydrochloride (EDCI) (287.6 mg, 1.50 mmol, 1.5 eq.), *N*,*N*-dimethylaminopyridine (12.2 mg, 0.10 mmol, 10 mol %) and then *L*-menthol (187.5 mg, 1.20 mmol, 1.2 eq.). The reaction mixture was stirred at room temperature for 12 h, before DCM was added. The mixture was washed with brine and dried over Na_2SO_4. After removal of the solvent, the residue was purified by column chromatography (*n*-pentane:Et_2O 40:1) to yield the title compound as a colorless oil (92.5 mg, 0.31 mmol, 31%).

R*f* = 0.30 (*n*-pentane:Et_2O 40:1).

^{1}H NMR (500 MHz, $CDCl_3$) δ 7.97 (d, $^3J_{HH}$ = 8.3 Hz, 2H, H-C5), 7.34 (d, $^3J_{HH}$ = 8.3 Hz, 2H, H-C6), 6.20 (t, $^4J_{HH}$ = 6.8 Hz, 1H, H-C3), 5.20 (d, $^4J_{HH}$ = 6.8 Hz, 2H, H-C1), 4.92 (td, $^3J_{HH}$ = 10.9, 4.4 Hz, 1H, H-C11), 2.18 – 2.06 (m, 1H, H-C16), 2.01 – 1.90 (m, 1H, H-C15), 1.78 – 1.68 (m, 2H, H-C13, H-C14), 1.58 – 1.52 (m, 2H, H-C8, H-C12), 1.19 – 1.04 (m, 2H, H-C13, H-C16), 0.96 – 0.88 (m, 7H, H-C9, H-C14), 0.79 (d, $^3J_{HH}$ = 7.0 Hz, 3H, H-C17).

^{13}C NMR (126 MHz, $CDCl_3$) δ 210.78 (C2), 166.06 (C10), 138.94 (C4), 130.06 (C6), 129.32 (C7), 126.61 (C5), 93.80 (C3), 79.31 (C1), 74.90 (C11), 47.45 (C12), 41.15 (C16), 34.49 (C14), 31.60 (C8), 26.69 (C15), 23.85 (C13), 22.20 (C9), 20.90 (C9), 16.72 (C17).

ESI-MS: (*m/z*) requires: [$(C_{20}H_{26}O_2Na)^+$] = 321.1825, (*m/z*) found: [$(C_{20}H_{26}O_2Na)^+$] = 321.1824.

FT-IR ($\tilde{\nu}$ = cm^{-1}): 2973 (m), 2881 (w), 1377 (w), 1084 (m), 1044 (s), 878 (m).

Methyl 4-(3-hydroxyprop-1-yn-1-yl)benzoate (Int 2)

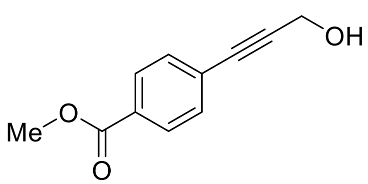

To a solution of methyl 4-iodobenzoate (3.93 g, 15.00 mmol, 1.0 eq.) in trimethylamine (37.5 mL) was added $Pd(PPh_3)_2Cl_2$ (105.3 mg, 0.15 mmol, 1 mol%), CuI (57.1 mg, 0.30 mmol, 2 mol%) and propargyl alcohol (1.01 g, 18.00 mmol, 1.2 eq.) under argon. The resulting mixture was stirred at ambient temperature for 12 h. Then, the reaction mixture was treated with a saturated $NaHCO_3$ solution and extracted with ethyl acetate (3 times). The combined organic layers were washed with a saturated NaCl solution, dried over Na_2SO_4 and concentrated under reduced pressure. The residue was purified by column chromatography (*n*-pentane:Et_2O 1:1.2-1:1.25) to yield the title compound as a brown solid (2.35 g, 12.35 mmol, 82%).

R*f* = 0.40 (*n*-pentane:Et_2O 1:1.25).

^{1}H NMR (400 MHz, $CDCl_3$) δ 8.05 – 7.93 (m, 2H), 7.57 – 7.43 (m, 2H), 4.52 (d, *J* = 6.2 Hz, 2H), 3.92 (s, 3H), 1.69 (t, *J* = 6.3 Hz, 1H).

GC-EI-MS: (*m/z*) requires: [($C_{11}H_{10}O_3$)] = 190.0630, (*m/z*) found: [($C_{11}H_{10}O_3$)] = 190.0624. Analytical data is in agreement with literature values.[9]

Methyl 4-(3-bromoprop-1-yn-1-yl)benzoate (Int 3)

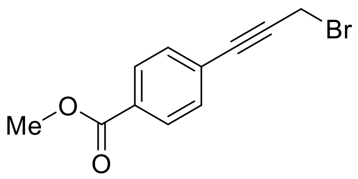

To a solution of methyl 4-(3-hydroxyprop-1-yn-1-yl)benzoate (**Int 2**) (1.90 g, 10.00 mmol, 1.0 eq.) in Et_2O (20 mL) at 0 °C was added PBr_3 (1.35 g, 5.00 mmol, 50 mol%). The solution was warmed to room temperature and stirred for 16 hours. The reaction was cooled to 0 °C. Water and 5% aq. K_2CO_3 were added. The organic phase was isolated, washed with brine, dried over Na_2SO_4 and concentrated in vacuo. The crude residue was purified by column chromatography (*n*-pentane) to yield the title compound as a white solid (2.10 g, 8.29 mmol, 83%).

$\mathbf{R}_f$ = 0.35 (*n*-pentane:Et_2O 10:1).

^{1}H NMR (400 MHz, $CDCl_3$) δ 8.04 – 7.92 (m, 2H), 7.56 – 7.45 (m, 2H), 4.16 (s, 2H), 3.92 (s, 3H).

ESI-MS: (*m/z*) requires: [($C_{11}H_{10}O_2{}^{79}Br)^+$] = 252.9859, (*m/z*) found: [($C_{11}H_{10}O_2{}^{79}Br)^+$] = 252.9859. Analytical data is in agreement with literature values.[9]

Methyl 4-(1-chlorobuta-2,3-dien-2-yl)benzoate (S23)

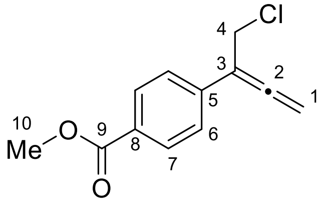

Under argon atmosphere, formaldehyde (37% aqueous solution, 0.62 mL, 8.25 mmol, 1.1 eq.) was added to a vigorously stirred solution of methyl 4-(3-bromoprop-1-yn-1-yl)benzoate (**Int 3**) (1.90 g, 7.50 mmol, 1.0 eq.) in THF/water (1:1, 30 mL). Indium powder (1.29 g, 11.25 mmol, 1.5 eq.) was added quickly, and vigorous stirring was continued for 12 h. The mixture was extracted with DCM. The combined organic layers were washed with brine, dried (Na_2SO_4), and evaporated. The residue was filtrated through a small pad of silica gel (*n*-pentane:Et_2O = 1:1.25). The solvent was removed under reduced pressure with the aid of a rotary evaporator to give the crude methyl 4-(1-hydroxybuta-2,3-dien-2-yl)benzoate, which was used without any further purification for the next step.

The crude methyl 4-(1-hydroxybuta-2,3-dien-2-yl)benzoate (550.9 mg, 2.70 mmol, 1.0 eq.) and PPh_3 (779.0 mg, 2.97 mmol, 1.1 eq.) were dissolved in dry DCM (0.2 M) under the argon. Then, NCS (396.6 mg, 2.97 mmol, 1.1 eq.) was added to the mixture slowly at 0 °C. The reaction mixture was allowed to warmed to room temperature and stir overnight. The mixture was quenched by the addition of H_2O and extracted with DCM. The combined organic layers were dried over Na_2SO_4,

filtered, and concentrated. The residue was purified by column chromatography (*n*-pentane:Et_2O 10:1) to yield the title compound as a white solid (536.4 mg, 2.41 mmol, 89%).

R*f* = 0.40 (*n*-pentane:Et_2O 10:1).

^{1}H NMR (500 MHz, $CDCl_3$) δ 8.02 (d, $^3J_{HH}$ = 8.7 Hz, 2H, H-C7), 7.53 (d, $^3J_{HH}$ = 8.8 Hz, 2H, H-C6), 5.32 (t, $^5J_{HH}$ = 1.7 Hz, 2H, H-C1), 4.54 (t, $^5J_{HH}$ = 1.6 Hz, 2H, H-C4), 3.92 (s, 3H, H-C10).

^{13}C NMR (126 MHz, $CDCl_3$) δ 210.51 (C2), 166.92 (C9), 138.01 (C5), 130.00 (C7), 129.02 (C8), 126.17 (C6), 103.01 (C3), 80.07 (C1), 52.25 (C10), 43.82 (C4).

ESI-MS: (*m/z*) requires: $[(C_{12}H_{11}O_2ClNa)^+]$ = 245.0340, (*m/z*) found: $[(C_{12}H_{11}O_2ClNa)^+]$ = 245.0339.

FT-IR ($\tilde{\nu}$ = cm^{-1}): 1922 (w), 1704 (s), 1607 (w), 1434 (m), 1268 (s), 1182 (m), 1107 (s), 1010 (w), 964 (w), 872 (m), 855 (m), 774 (m), 734 (m), 694 (s), 631 (m).

Melting Point: 71-72 ˚C.

3-(4-(Trifluoromethyl)phenyl)prop-2-yn-1-ol (Int 4)

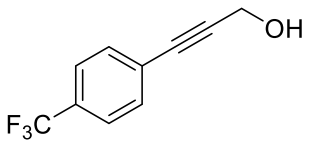

To a solution of 4-iodobenzotrifluoride (4.08 g, 15.00 mmol, 1.0 eq.) in trimethylamine (37.5 mL) was added $Pd(PPh_3)_2Cl_2$ (105.3 mg, 0.15 mmol, 1 mol%), CuI (57.1 mg, 0.30 mmol, 2 mol%) and propargyl alcohol (1.01 g, 18.00 mmol, 1.2 eq.) under argon. The resulting mixture was stirred at ambient temperature for 12 h. Then, the reaction mixture was treated with a saturated $NaHCO_3$ solution and extracted with ethyl acetate (3 times). The combined organic layers were washed with a saturated NaCl solution, dried over Na_2SO_4 and concentrated under reduced pressure. The residue was purified by column chromatography (*n*-pentane:Et_2O 1.5:1-1:1) to yield the title compound as a brown solid (961.3 mg, 4.80 mmol, 32%).

R*f* = 0.40 (*n*-pentane:Et_2O 1:1).

^{1}H NMR (400 MHz, $CDCl_3$) δ 7.67 – 7.47 (m, 4H), 4.52 (d, *J* = 5.7 Hz, 2H), 1.68 (t, *J* = 6.1 Hz, 1H).

^{19}F{^{1}H} NMR (377 MHz, $CDCl_3$) δ -62.90 (s, 3F).

GC-EI-MS: (*m/z*) requires: $[(C_{10}H_7F_3O)]$ = 200.0449, (*m/z*) found: $[(C_{10}H_7F_3O)]$ = 200.0442.

Analytical data is in agreement with literature values.[10]

1-(3-Bromoprop-1-yn-1-yl)-4-(trifluoromethyl)benzene (Int 5)

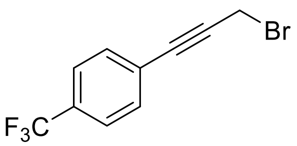

To a solution of 3-(4-(Trifluoromethyl)phenyl)prop-2-yn-1-ol (**Int 4**) (800.6 mg, 4.00 mmol, 1.0 eq.) in Et_2O (8 mL) at 0 °C was added PBr_3 (541.4 mg, 2.00 mmol, 50 mol%). The solution was warmed to room temperature and stirred for 16 hours. The reaction was cooled to 0 °C. Water and 5% aq. K_2CO_3 were added. The organic phase was isolated, washed with brine, dried over Na_2SO_4 and concentrated in vacuo. The crude residue was purified by column chromatography (*n*-pentane) to yield the title compound as a colorless oil (869.8 mg, 3.31 mmol, 83%).

$\mathbf{R}_f$ = 0.50 (*n*-pentane).

^{1}H NMR (400 MHz, $CDCl_3$) δ 7.67 – 7.45 (m, 4H), 4.15 (s, 2H).

^{19}F{^{1}H} NMR (377 MHz, $CDCl_3$) δ -62.94 (s, 3F).

GC-EI-MS: (*m/z*) requires: [($C_{10}H_6F_3{}^{79}Br$)] = 261.9605, (*m/z*) found: [($C_{10}H_6F_3{}^{79}Br$)] = 261.9608.

Analytical data is in agreement with literature values.[11]

1-(1-Chlorobuta-2,3-dien-2-yl)-4-(trifluoromethyl)benzene (S24)

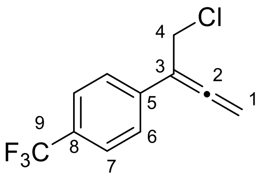

Under argon atmosphere, formaldehyde (37% aqueous solution, 0.17 mL, 2.20 mmol, 1.1 eq.) was added to a vigorously stirred solution of 1-(3-bromoprop-1-yn-1-yl)-4-(trifluoromethyl)benzene (**Int 5**) (526.1 mg, 2.00 mmol, 1.0 eq.) in THF/water (1:1, 8 mL). Indium powder (344.5 mg, 3.00 mmol, 1.5 eq.) was added quickly, and vigorous stirring was continued for 12 h. The mixture was extracted with DCM. The combined organic layers were washed with brine, dried (Na_2SO_4), and evaporated. The residue was filtrated through a small pad of silica gel (*n*-pentane:Et_2O = 2.5:1). The solvent was removed under reduced pressure with the aid of a rotary evaporator to give the crude 2-(4-(trifluoromethyl)phenyl)buta-2,3-dien-1-ol, which was used without any further purification for the next step.

To a solution of crude 2-(4-(trifluoromethyl)phenyl)buta-2,3-dien-1-ol (225.8 mg, 1.05 mmol, 1.0 eq.) in MeCN (0.6 mL) at 0 °C was added PPh_3 (359.5 mg, 1.37 mmol, 1.3 eq.) and CCl_4 (0.5 mL). The solution was warmed to room temperature and stirred for 2 hours. The reaction mixture was directly purified by column chromatography (*n*-pentane) to yield the title compound as a pale yellow oil (72.3 mg, 0.31 mmol, 30%).

$\mathbf{R}_f$ = 0.50 (*n*-pentane).

^{1}H NMR (500 MHz, $CDCl_3$) δ 7.65 – 7.54 (m, 4H, H-C6, H-C7), 5.33 (s, 2H, H-C1), 4.54 (t, $^{5}J_{HH}$ = 1.6 Hz, 2H, H-C4).

^{13}C NMR (126 MHz, $CDCl_3$) δ 210.29 (C2), 137.05 (C5), 129.45 (q, $^{2}J_{CF}$ = 32.6 Hz, C8), 126.54 (C6), 125.67 (q, $^{3}J_{CF}$ = 3.9 Hz, C7), 124.27 (q, $^{1}J_{CF}$ = 272.2 Hz, C9), 102.65 (C3), 80.17 (C1), 43.80 (C4).

^{19}F NMR (470 MHz, $CDCl_3$) δ -62.59 (s, 3F, F-C9).

^{19}F{^{1}H} NMR (470 MHz, $CDCl_3$) δ -62.59 (s, 3F, F-C9).

GC-EI-MS: (*m/z*) requires: [($C_{11}H_8F_3Cl$)] = 232.0267, (*m/z*) found: [($C_{11}H_8F_3Cl$)] = 232.0262.

FT-IR ($\tilde{\nu}$ = cm^{-1}): 1940 (w), 1618 (w), 1325 (s), 1262 (w), 1165 (m), 1113 (s), 1067 (s), 1015 (m), 843 (s), 717 (m), 625 (m).

2-Benzylbuta-2,3-dien-1-ol (Int 6)

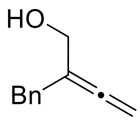

To a pressure tube were added benzyl bromide (4.70 g, 27.5 mmol, 1.1 eq.) and ethyl (triphenylphosphoranylidene)acetate (8.71 g, 25.00 mmol, 1.0 eq.) in $CHCl_3$ (75.0 mL) under Ar atmosphere. The reaction mixture was heated to 65 °C, until all of the triphenylphosphorane had disappeared (monitored using TLC). The solvent was evaporated under reduced pressure. Then triethylamine (5.56 g, 55.0 mmol, 2.2 eq.) and DCM (100 mL) were added to the resulting phosphonium salt. After 1 h, AcCl (2.16 g, 27.5 mmol, 1.1 eq.) was added at 0 ºC and then the reaction mixture was allowed to warm up to room temperature and stirred for another 12 h. After completion of the reaction, the reaction mixture was filtered to remove insoluble solid. The filtrate was then concentrated under reduced pressure and the residue was filtrated through a small pad of silica gel (*n*-pentane:Et_2O 25:1). The solvent was removed under reduced pressure with the aid of a rotary evaporator to give the crude product ethyl 2-benzylbuta-2,3-dienoate, which was used without any further purification for the next step.

A dry three-necked flask was charged with the crude ethyl 2-benzylbuta-2,3-dienoate (1.72 g, 8.50 mmol, 1.0 eq.) in dry toluene (22 mL). DIBAL-H (1.2 M solution in toluene,14.9 mL, 2.1 eq.) was added dropwise at -78 °C under Ar atmosphere. When the addition was completed, the mixture was stirred at -78 °C for 4 h. 10 mL of methanol was added to quench the reaction at 0 °C. Then, brine was added, and the mixture was stirred for 5 min, filtered, and separated. The aqueous layer was extracted with diethyl ether. The combined ether layer was dried over anhydrous sodium sulfate.

Evaporation and purification by column chromatography (*n*-pentane:Et_2O 2.5:1) to yield the title compound as a pale yellow oil (607.3 mg, 3.79 mmol, 45%).

R*f* = 0.40 (*n*-pentane:Et_2O 2.5:1).

^{1}H NMR (400 MHz, $CDCl_3$) δ 7.32 – 7.27 (m, 2H), 7.25 – 7.18 (m, 3H), 4.87 (p, *J* = 2.8 Hz, 2H), 4.04 (t, *J* = 3.0 Hz, 2H), 3.39 (t, *J* = 2.6 Hz, 2H).

GC-EI-MS: (*m/z*) requires: [($C_{11}H_{12}O$)] = 160.0888, (*m/z*) found: [($C_{11}H_{12}O$)] = 160.0883.

Analytical data is in agreement with literature values.[12]

(2-(Chloromethyl)buta-2,3-dien-1-yl)benzene (S25)

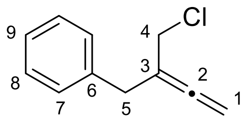

To a solution of 2-Benzylbuta-2,3-dien-1-ol (**Int 6**) (320.4 mg, 2.00 mmol, 1.0 eq.) in MeCN (0.3 mL) at 0 °C was added PPh_3 (682.0 mg, 2.60 mmol, 1.3 eq.) and CCl_4 (0.3 mL). The solution was warmed to room temperature and stirred for 2 hours. The reaction mixture was directly purified by column chromatography (*n*-pentane) to yield the title compound as a colorless oil (299.7 mg, 1.68 mmol, 84%).

R*f* = 0.55 (*n*-pentane).

^{1}H NMR (400 MHz, $CDCl_3$) δ 7.35 – 7.29 (m, 2H, H-C7), 7.28 – 7.21 (m, 3H, H-C8, H-C9), 4.90 – 4.79 (m, 2H, H-C1), 4.03 (t, $^5J_{HH}$ = 1.8 Hz, 2H, H-C4), 3.49 (t, $^5J_{HH}$ = 2.6 Hz, 2H, H-C5).

^{13}C NMR (151 MHz, $CDCl_3$) δ 207.73 (C2), 138.44 (C6), 129.19 (C8), 128.57 (C7), 126.73 (C9), 100.73 (C3), 76.61 (C1), 45.94 (C4), 36.24 (C5).

GC-EI-MS: (*m/z*) requires: [($C_{11}H_{11}Cl$)] = 178.0549, (*m/z*) found: [($C_{11}H_{11}Cl$)] = 178.0543.

FT-IR ($\tilde{\nu}$ = cm^{-1}): 1957 (m), 1601 (w), 1492 (w), 1452 (w), 1434 (m), 1245 (m), 1073 (w), 1027 (w), 906 (w), 855 (s), 786 (m), 728 (s), 694 (s).

2-Vinylidenetetradecan-1-ol (Int 7)

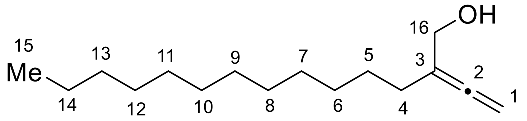

To a pressure tube were added 1-dodecylbromide (6.85 g, 27.5 mmol, 1.1 eq.) and ethyl (triphenylphosphoranylidene)acetate (8.71 g, 25.00 mmol, 1.0 eq.) in $CHCl_3$ (75.0 mL) under Ar atmosphere. The reaction mixture was heated to 65 °C, until all of the triphenylphosphorane had disappeared (monitored using TLC). The solvent was evaporated under reduced pressure. Then triethylamine (5.56 g, 55.0 mmol, 2.2 eq.) and DCM (100 mL) were added to the resulting phosphonium salt. After 1 h, AcCl (2.16 g, 27.5 mmol, 1.1 eq.) was added at 0 ºC and then the reaction mixture was allowed to warm up to room temperature and

stirred for another 12 h. After completion of the reaction, the reaction mixture was filtered to remove insoluble solid. The filtrate was then concentrated under reduced pressure and the residue was filtrated through a small pad of silica gel (*n*-pentane:Et_2O 40:1). The solvent was removed under reduced pressure with the aid of a rotary evaporator to give the crude product ethyl 2-vinylidenetetradecanoate, which was used without any further purification for the next step.

A dry three-necked flask was charged with the crude ethyl 2-vinylidenetetradecanoate (1.40 g, 5.00 mmol, 1.0 eq.) in dry toluene (13 mL). DIBAL-H (1.2 M solution in toluene, 8.8 mL, 2.1 eq.) was added dropwise at -78 °C under Ar atmosphere. When the addition was completed, the mixture was stirred at -78 °C for 4 h. 5 mL of methanol was added to quench the reaction at 0 °C. Then, brine was added, and the mixture was stirred for 5 min, filtered, and separated. The aqueous layer was extracted with diethyl ether. The combined ether layer was dried over anhydrous sodium sulfate. Evaporation and purification by column chromatography (*n*-pentane:Et_2O 4:1) to yield the title compound as a colorless oil (642.8 mg, 2.70 mmol, 54%).

R_f = 0.35 (*n*-pentane:Et_2O 4:1).

^{1}H NMR (500 MHz, $CDCl_3$) δ 4.87 (p, $^5J_{HH}$ = 3.2 Hz, 2H, H-C1), 4.09 – 4.00 (m, 2H, H-C16), 1.99 (tt, $^3J_{HH}$ = 7.0 Hz, $^5J_{HH}$ = 3.2 Hz, 2H, H-C4), 1.51 – 1.39 (m, 3H, H-C5, OH), 1.34 – 1.23 (m, 18H, H-C6~C14), 0.88 (t, $^3J_{HH}$ = 6.9 Hz, 3H, H-C15).

^{13}C NMR (126 MHz, $CDCl_3$) δ 204.39 (C2), 104.91 (C3), 78.63 (C1), 63.01 (C16), 32.07 (*C*H_2), 29.82 (*C*H_2), 29.80 (*C*H_2), 29.77 (*C*H_2), 29.60 (*C*H_2), 29.51 (*C*H_2), 29.48 (*C*H_2), 28.97 (C4), 27.67 (C5), 22.84 (*C*H_2), 14.26 (C15).

ESI-MS: (*m/z*) requires: [$(C_{16}H_{30}ONa)^+$] = 261.2189, (*m/z*) found: [$(C_9H_5O_3F_3SNa)^+$] = 261.2188.

FT-IR ($\tilde{\nu}$ = cm^{-1}): 2921 (s), 2852 (s), 1957 (w), 1463 (m), 1371 (w), 1010 (m), 912 (w), 843 (m), 757 (m), 734 (m), 642 (w).

3-(Chloromethyl)pentadeca-1,2-diene (S26)

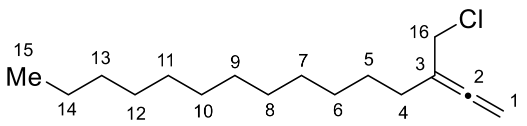

To a solution of 2-vinylidenetetradecan-1-ol (**Int 7**) (642.8 mg, 2.70 mmol, 1.0 eq.) in MeCN (0.4 mL) at 0 °C was added PPh_3 (920.6 mg, 3.51 mmol, 1.3 eq.) and CCl_4 (0.4 mL). The solution was warmed to room temperature and stirred for 2 hours. The reaction mixture was directly purified by column chromatography (*n*-pentane) to yield the title compound as a colorless oil (601.2 mg, 2.34 mmol, 87%).

R_f = 0.90 (*n*-pentane).

^{1}H NMR (500 MHz, $CDCl_3$) δ 4.82 (tt, $^{5}J_{HH}$ = 3.5, 1.8 Hz, 2H, H-C1), 4.10 (t, $^{5}J_{HH}$ = 1.8 Hz, 2H, H-C16), 2.14 – 2.06 (m, 2H, H-C4), 1.49 – 1.40 (m, 2H, H-C5), 1.35 – 1.23 (m, 18H, H-C6~C14), 0.88 (t, $^{3}J_{HH}$ = 6.9 Hz, 3H, H-C15).

^{13}C NMR (126 MHz, $CDCl_3$) δ 207.16 (C2), 101.32 (C3), 76.89 (C1), 47.26 (C16), 32.08 (*C*H_2), 29.82 (*C*H_2), 29.80 (*C*H_2), 29.76 (*C*H_2), 29.59 (*C*H_2), 29.51 (*C*H_2), 29.37 (*C*H_2), 29.22 (C4), 27.34 (C5), 22.84 (*C*H_2), 14.27 (C15).

GC-EI-MS: (*m/z*) requires: [($C_{16}H_{29}$)] = 221.2264 [M-H], (*m/z*) found: [($C_{16}H_{29}$)] = 221.2265.

FT-IR ($\tilde{\nu}$ = cm^{-1}): 2921 (s), 2852 (m), 1957 (w), 1463 (w), 1251 (w), 849 (m), 711 (s), 625 (w).

3-(Fluoromethyl)pentadeca-1,2-diene (S27)

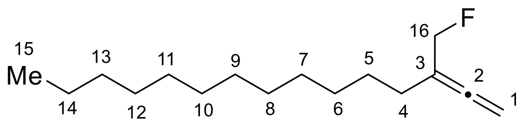

To a solution of 2-vinylidenetetradecan-1-ol (**Int 7**) (238.4 mg, 1.00 mmol, 1.0 eq.) in dry DCM (4 mL) under argon at -78 °C was added DAST (209.5 mg, 1.30 mmol, 1.3 eq.) dropwise. Then, the solution was warmed to room temperature and stirred overnight. The reaction was quenched carefully by addition of saturated $NaHCO_3$ aqueous solution dropwise, until no bubbles were observed. The organic phase was separated, and the aqueous phase was extracted with DCM. The combined organic layers were washed with brine, dried over Na_2SO_4, and concentrated under reduced pressure. The residue was purified by column chromatography (*n*-pentane) to yield the title compound as a colorless oil (61.5 mg, 0.26 mmol, 26%).

R_f = 0.75 (*n*-pentane).

^{1}H NMR (500 MHz, $CDCl_3$) δ 4.90 – 4.77 (m, 4H, H-C1, H-C16), 2.11 – 2.02 (m, 2H, H-C4), 1.51 – 1.41 (m, 2H, H-C5), 1.34 – 1.24 (m, 18H, H-C6~C14), 0.88 (t, $^{3}J_{HH}$ = 6.9 Hz, 3H, H-C15).

^{13}C NMR (126 MHz, $CDCl_3$) δ 207.49 (d, $^{3}J_{CF}$ = 10.4 Hz, C2), 100.41 (d, $^{2}J_{CF}$ = 18.8 Hz, C3), 84.34 (d, $^{1}J_{CF}$ = 169.6 Hz, C16), 76.56 (d, $^{4}J_{CF}$ = 3.5 Hz, C1), 32.08 (*C*H_2), 29.83 (*C*H_2), 29.80 (*C*H_2), 29.77 (*C*H_2), 29.59 (*C*H_2), 29.51 (*C*H_2), 29.41 (*C*H_2), 28.73 (C4), 27.55 (C5), 22.85 (*C*H_2), 14.26 (C15).

^{19}F NMR (470 MHz, $CDCl_3$) δ -211.86 (tt, $^{2}J_{HF}$ = 48.4 Hz, $^{4}J_{HF}$ = 11.1 Hz, 1F, F-C16).

^{19}F{^{1}H} NMR (470 MHz, $CDCl_3$) δ -211.86 (s, 1F, F-C16).

GC-EI-MS: (*m/z*) requires: [($C_{16}H_{29}F$)] = 240.2253, (*m/z*) found: [($C_{16}H_{29}F$)] = 240.2246.

FT-IR ($\tilde{\nu}$ = cm^{-1}): 2921 (s), 2852 (m), 1463 (w), 1371 (w), 981 (s), 849 (m), 723 (w).

3-(Ethoxymethyl)pentadeca-1,2-diene (S28)

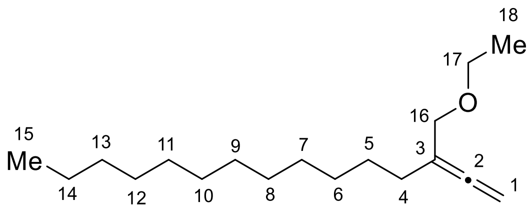

2-Vinylidenetetradecan-1-ol (**Int 7**) (268.1 mg, 1.13 mmol, 1.0 eq.) was dissolved in anhydrous DMF (2.3 mL). NaH (54.0 mg, 60% in mineral oil, 1.35 mmol, 1.2 eq.) was slowly added to the solution at 0 °C. The resulting mixture was stirred at 0 °C for 1.5 h before slow addition of ethyl iodide (263.2 mg, 1.69 mmol, 1.5 eq.). The reaction was slowly warmed to room temperature over 30 min and then stirred for additional 2 h before being quenched by aq. NH_4Cl and diluted with water. The product was extracted by ether. The combined organic layer was then washed with water and brine, and dried over sodium sulfate. The organic layer was filtered and concentrated under reduced pressure. The residue was purified by column chromatography (*n*-pentane) to yield the title compound as a colorless oil (202.9 mg, 0.76 mmol, 68%).

$\mathbf{R}_{\boldsymbol{f}}$ = 0.45 (*n*-pentane:Et_2O 50:1).

^{1}H NMR (500 MHz, $CDCl_3$) δ 4.72 (tt, $^5J_{HH}$ = 3.3, 2.2 Hz, 2H, H-C1), 3.96 (t, $^5J_{HH}$ = 2.2 Hz, 2H, H-C16), 3.47 (q, $^3J_{HH}$ = 7.0 Hz, 2H, H-C17), 2.05 – 1.96 (m, 2H, H-C4), 1.50 – 1.40 (m, 2H, H-C5), 1.35 – 1.23 (m, 18H, H- C6~C14), 1.20 (t, $^3J_{HH}$ = 7.0 Hz, 3H, H-C18), 0.88 (t, $^3J_{HH}$ = 6.9 Hz, 3H, H-C5).

^{13}C NMR (126 MHz, $CDCl_3$) δ 206.68 (C2), 101.02 (C3), 75.63 (C1), 71.67 (C16), 65.26 (C17), 32.08 (*C*H_2), 29.83 (*C*H_2), 29.81 (*C*H_2), 29.80 (*C*H_2), 29.79 (*C*H_2), 29.64 (*C*H_2), 29.51 (*C*H_2), 29.10 (C4), 27.58 (C5), 22.84 (*C*H_2), 15.27 (C18), 14.26 (C15).

ESI-MS: (*m/z*) requires: [$(C_{18}H_{34}ONa)^+$] = 289.2502, (*m/z*) found: [$(C_{18}H_{34}ONa)^+$] = 289.2503.

FT-IR ($\tilde{\nu}$ = cm^{-1}): 2921 (s), 2852 (m), 1957 (w), 1463 (w), 1096 (s), 843 (m), 757 (w), 734 (w).

N,4-dimethyl-N-(2-vinylidenetetradecyl)benzenesulfonamide (S29)

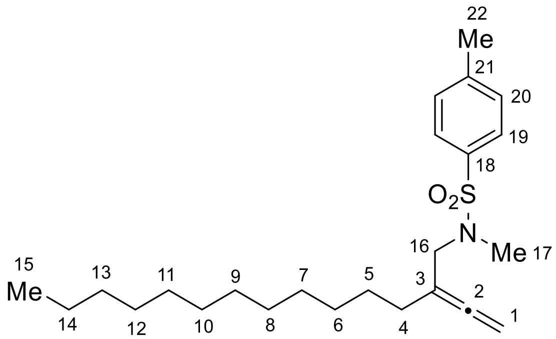

To a solution of 2-vinylidenetetradecan-1-ol (**Int 7**) (238.4 mg, 1.00 mmol, 1.0 eq.), N-methyl-p-toluenesulfonamide (203.8 mg, 1.10 mmol, 1.1 eq.), and triphenylphosphine (288.5 mg, 1.10 mmol, 1.1 eq.) in THF (3 mL) was added diisopropyl azodicarboxylate (222.4 mg, 1.10 mmol, 1.1 eq.) dropwise at 0 °C under argon. The reaction mixture was warmed to rt and stirred for 16 h. Then the reaction mixture was concentrated and purified by

column chromatography (n-pentane:Et_2O 15:1-12:1) to yield the title compound as a white solid (194.2 mg, 0.48 mmol, 48%).

R_f = 0.30 (n-pentane:Et_2O 15:1).

^{1}H NMR (500 MHz, $CDCl_3$) δ 7.66 (d, $^3J_{HH}$ = 8.3 Hz, 2H, H-C19), 7.31 (d, $^3J_{HH}$ = 8.1 Hz, 2H, H-C20), 4.71 – 4.63 (m, 2H, H-C1), 3.58 – 3.50 (m, 2H, H-C16), 2.64 (s, 3H, H-C17), 2.43 (s, 3H, H-C22), 1.99 (tt, $^3J_{HH}$ = 7.1 Hz, $^5J_{HH}$ = 3.3 Hz, 2H, H-C4), 1.48 – 1.39 (m, 2H, H-C5), 1.33 – 1.22 (m, 18H, H- C6~C14), 0.88 (t, $^3J_{HH}$ = 6.9 Hz, 3H, H-C15).

^{13}C NMR (126 MHz, $CDCl_3$) δ 207.43 (C2), 143.43 (C18), 134.55 (C21), 129.77 (C20), 127.62 (C19), 99.07 (C3), 76.23 (C1), 53.18 (C16), 34.09 (C17), 32.08 (CH_2), 29.84 (CH_2), 29.81 (CH_2), 29.80 (CH_2), 29.62 (CH_2), 29.51 (CH_2), 29.43 (CH_2), 28.98 (C4), 27.40 (C5), 22.84 (CH_2), 21.64 (C22), 14.27 (C15).

ESI-MS: (m/z) requires: [$(C_{24}H_{39}O_2NSNa)^+$] = 428.2594, (m/z) found: [$(C_{24}H_{39}O_2NSNa)^+$] = 428.2594.

FT-IR ($\tilde{\nu}$ = cm^{-1}): 2927 (m), 2852 (w), 1457 (w), 1343 (m), 1159 (s), 1090 (w), 981 (w), 912 (s), 849 (w), 815 (w), 751 (s), 734 (s), 654 (s).

Melting Point: 34.5-35.5 ˚C.

4-Vinylidenehexadecane (S30)

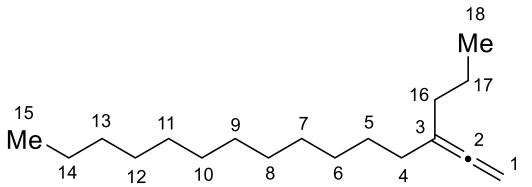

TsCl (686.3 mg, 3.60 mmol, 1.2 eq.) was added to a solution of pentadec-2-yn-1-ol (674.2 mg, 3.00 mmol, 1.0 eq.) in Et_2O (8 mL) under argon. The reaction mixture was cooled to 0 °C, then KOH (300 mg/mmol substrate) was added in small portions. The solution was allowed to warm to room temperature and stir for 30 min. The reaction mixture was poured on ice. The aqueous phase was extracted with Et_2O. The combined organic phases were washed with brine, dried over Na_2SO_4 and concentrated. The crude pentadec-2-yn-1-yl 4-methylbenzenesulfonate was directly used in next step without further purification.

A mixture of CuBr (43.0 mg, 0.30 mmol, 10 mol%), THF (6 mL) and the crude pentadec-2-yn-1-yl 4-methylbenzenesulfonate was cooled to 0 °C. n-Propylmagnesium chloride (2 M in Et_2O, 1.9 mL, 1.25 eq.) was added dropwise. The reaction mixture was allowed to warm to room temperature and stirred for 2 h. It was quenched by addition of a saturated aqueous solution of NH_4Cl followed by extraction with Et_2O. The combined organic phases were washed with brine, dried over Na_2SO_4

and concentrated. The residue was purified by column chromatography (*n*-pentane) to yield the title compound as a colorless oil (422.3 mg, 1.69 mmol, 56%).

R_f = 0.90 (*n*-pentane).

^{1}H NMR (599 MHz, $CDCl_3$) δ 4.63 (p, $^5J_{HH}$ = 3.2 Hz, 2H, H-C1), 1.96 – 1.87 (m, 4H, H-C4, H-C16), 1.50 – 1.38 (m, 4H, H-C5, H-C17), 1.32 – 1.24 (m, 18H, H-C6~C14), 0.92 (t, $^3J_{HH}$ = 7.4 Hz, 3H, H-C18), 0.88 (t, $^3J_{HH}$ = 7.0 Hz, 3H, H-C15).

^{13}C NMR (151 MHz, $CDCl_3$) δ 205.93 (C2), 103.29 (C3), 75.23 (C1), 34.44 (CH_2), 32.28 (CH_2), 32.09 (CH_2), 29.84 (CH_2), 29.83 (CH_2), 29.81 (CH_2), 29.68 (CH_2), 29.54 (CH_2), 29.52 (CH_2), 27.72 (CH_2), 22.85 (CH_2), 20.93 (CH_2), 14.27 (C15), 14.02 (C18).

GC-EI-MS: (*m/z*) requires: [($C_{18}H_{34}$)] = 250.2661, (*m/z*) found: [($C_{18}H_{34}$)] = 250.2657.

FT-IR ($\tilde{\nu}$ = cm^{-1}): 2921 (s), 2852 (s), 1957 (w), 1457 (m), 1377 (w), 843 (s), 723 (w).

(1S,2R,5S)-2-Isopropyl-5-methylcyclohexyl 4-(1-chlorobuta-2,3-dien-2-yl)benzoate (S31)

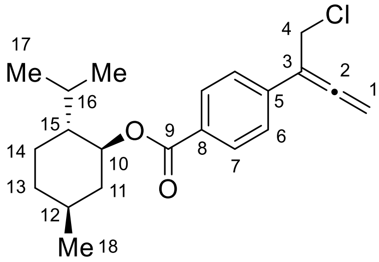

To a solution of 4-iodobenzoic acid (4.96 g, 20.00 mmol, 1.0 eq.) in DCM (40 mL) was added 1-ethyl-3-(3-(dimethylamino)propyl)-carbodiimide hydrochloride (EDCI) (5.75 g, 30.00 mmol, 1.5 eq.), *N*,*N*-dimethylaminopyridine (244.3 mg, 2.00 mmol, 10 mol %) and then *D*-menthol (5.75 g, 24.00 mmol, 1.2 eq.). The reaction mixture was stirred at room temperature for 12 h, before DCM was added. The mixture was washed with brine and dried over Na_2SO_4. After removal of the solvent, the residue was filtrated through a small pad of silica gel (*n*-pentane:Et_2O = 50:1). The solvent was removed under reduced pressure with the aid of a rotary evaporator to give the crude (1S,2R,5S)-2-isopropyl-5-methylcyclohexyl 4-iodobenzoate, which was used without any further purification for the next step.

To a solution of the crude (1S,2R,5S)-2-isopropyl-5-methylcyclohexyl 4-iodobenzoate (5.79 g, 15.00 mmol, 1.0 eq.) in trimethylamine (37.5 mL) was added $Pd(PPh_3)_2Cl_2$ (105.3 mg, 0.15 mmol, 1 mol%), CuI (57.1 mg, 0.30 mmol, 2 mol%) and propargyl alcohol (1.01 g, 18.00 mmol, 1.2 eq.) under argon. The resulting mixture was stirred at ambient temperature for 12 h. Then, the reaction mixture was treated with a saturated $NaHCO_3$ solution and extracted with ethyl acetate (3 times). The combined organic layers were washed with a saturated NaCl solution, dried over Na_2SO_4 and concentrated under reduced pressure. The residue was filtrated through a small pad of silica gel (*n*-pentane:EA = 3:1). The solvent was removed under reduced pressure with the aid of a rotary

evaporator to give the crude (1S,2R,5S)-2-isopropyl-5-methylcyclohexyl 4-(3-hydroxyprop-1-yn-1-yl)benzoate, which was used without any further purification for the next step.

To a solution of the crude (1S,2R,5S)-2-isopropyl-5-methylcyclohexyl 4-(3-hydroxyprop-1-yn-1-yl)benzoate (3.14 g, 10.00 mmol, 1.0 eq.) in Et_2O (20 mL) at 0 °C was added PBr_3 (1.35 g, 5.00 mmol, 50 mol%). The solution was warmed to room temperature and stirred for 16 hours. The reaction was cooled to 0 °C. Water and 5% aq. K_2CO_3 were added. The organic phase was isolated, washed with brine, dried over Na_2SO_4 and concentrated in vacuo. The residue was filtrated through a small pad of silica gel (*n*-pentane: Et_2O = 40:1). The solvent was removed under reduced pressure with the aid of a rotary evaporator to give the crude (1S,2R,5S)-2-isopropyl-5-methylcyclohexyl 4-(3-bromoprop-1-yn-1-yl)benzoate, which was used without any further purification for the next step.

Under argon atmosphere, formaldehyde (37% aqueous solution, 0.50 mL, 6.60 mmol, 1.1 eq.) was added to a vigorously stirred solution of the crude (1S,2R,5S)-2-isopropyl-5-methylcyclohexyl 4-(3-bromoprop-1-yn-1-yl)benzoate (2.26 g, 6.00 mmol, 1.0 eq.) in THF/water (1:1, 24 mL). Indium powder (1.03 g, 9.00 mmol, 1.5 eq.) was added quickly, and vigorous stirring was continued for 12 h. The mixture was extracted with DCM. The combined organic layers were washed with brine, dried (Na_2SO_4), and evaporated. The residue was filtrated through a small pad of silica gel (*n*-pentane:Et_2O = 2:1). The solvent was removed under reduced pressure with the aid of a rotary evaporator to give the crude (1S,2R,5S)-2-isopropyl-5-methylcyclohexyl 4-(1-hydroxybuta-2,3-dien-2-yl)benzoate, which was used without any further purification for the next step.

The crude (1S,2R,5S)-2-isopropyl-5-methylcyclohexyl 4-(1-hydroxybuta-2,3-dien-2-yl)benzoate (656.9 mg, 2.00 mmol, 1.0 eq.) and PPh_3 (577.0 mg, 2.20 mmol, 1.1 eq.) were dissolved in dry DCM (0.2 M) under the argon. Then, NCS (293.8 mg, 2.20 mmol, 1.1 eq.) was added to the mixture slowly at 0 °C. The reaction mixture was allowed to warmed to room temperature and stir overnight. The mixture was quenched by the addition of H_2O and extracted with DCM. The combined organic layers were dried over Na_2SO_4, filtered, and concentrated. The residue was purified by column chromatography (*n*-pentane:Et_2O 40:1) to yield the title compound as a colorless oil (607.5 mg, 1.75 mmol, 88%).

$\mathbf{R_{\mathit{f}}}$ = 0.30 (*n*-pentane:Et_2O 40:1).

^{1}H NMR (599 MHz, $CDCl_3$) δ 8.05 – 8.01 (m, 2H, H-C7), 7.58 – 7.47 (m, 2H, H-C6), 5.31 (t, $^5J_{HH}$ = 1.7 Hz, 2H, H-C1), 4.93 (td, $^3J_{HH}$ = 10.9, 4.4 Hz, 1H, H-C10), 4.54 (t, $^5J_{HH}$ = 1.6 Hz, 2H, H-C4), 2.16 – 2.10 (m, 1H, H-C11), 2.00 – 1.92 (m, 1H, H-C12), 1.77 – 1.70 (m, 2H, H-C13, H-C14),

1.60 – 1.53 (m, 2H, H-C15, H-C16), 1.18 – 1.06 (m, 2H, H-C11, H-C14), 0.95 – 0.89 (m, 7H, H-C13, H-C17), 0.79 (d, $^{3}J_{HH}$ = 6.9 Hz, 3H, H-C18).

^{13}C NMR (151 MHz, $CDCl_3$) δ 210.45 (C2), 165.91 (C9), 137.78 (C8), 129.98 (C7), 129.76 (C5), 126.13 (C6), 103.05 (C3), 79.98 (C1), 75.02 (C10), 47.44 (C15), 43.86 (C4), 41.12 (C11), 34.48 (C13), 31.60 (C16), 26.69 (C12), 23.84 (C14), 22.19 (C17), 20.89 (C17'), 16.71 (C18).

ESI-MS: (*m/z*) requires: $[(C_{21}H_{27}ClNaO_2)^+]$ = 369.1592, (*m/z*) found: $[(C_{21}H_{27}ClNaO_2)^+]$ = 369.1591.

FT-IR ($\tilde{\nu}$ = cm^{-1}): 2955 (m), 2359 (m), 1710 (s), 1607 (m), 1452 (w), 1274 (s), 1182 (m), 1113 (m), 855 (m), 774 (w), 700 (w).

1.3 Preparation and characterisation of catalysts

Dimethyl 2,2'-((2-iodo-1,3-phenylene)bis(oxy))(2*R*,2'*R*)-bis(3-methylbutanoate)

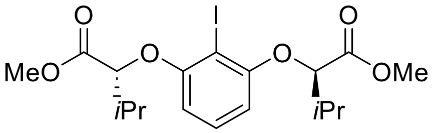

This compound was prepared according to a previous report from our research group.[13]

(1'R)-2'-(((2R)-2-(2-Iodo-3-(((2R)-3-methyl-1-oxo-1-((2'-((2,4,6-trimethylbenzoyl)oxy)-[1,1'-binaphthalen]-2-yl)oxy)butan-2-yl)oxy)phenoxy)-3-methylbutanoyl)oxy)-[1,1'-binaphthalen]-2-yl 2,4,6-trimethylbenzoate

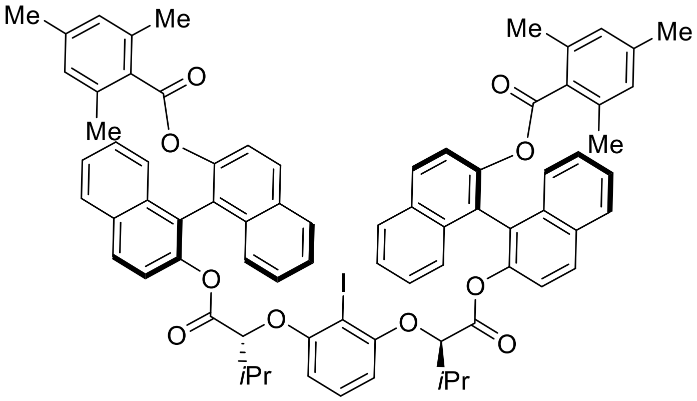

This compound was prepared according to a previous report from our research group.[13]

1.4 Preparation and characterisation of propargylic fluorides

General Procedure B for regioselective fluorination of allenes

Unless otherwise stated, a Teflon® vial was equipped with a 1 cm stirring bar followed by the addition of allene (0.1 mmol, 1.0 eq.), 1-iodo-2,4-dimethylbenzene (7 mg, 0.03 mmol, 30 mol%) and DCM (0.25 mL). The stated amine:HF mixture was added (0.25 mL) via syringe. After stirring for 1 min, Selectfluor® (71 mg, 0.2 mmol, 2.0 eq.) was added in one portion. The reaction vessel was then sealed with a Teflon® screw cap. After stirring (350 rpm) at ambient temperature for 24 h, the reaction mixture was poured into 100 mL of a saturated solution of $NaHCO_3$ (CAUTION, generation of CO_2!). The Teflon® vial was rinsed with DCM and dropped into another flask of saturated aqueous solution of $NaHCO_3$ to guarantee the removal of excess HF. The organics were extracted with DCM (3x 30 mL), the combined organic layers were dried over Na_2SO_4, filtered and the solvent was carefully removed under reduced pressure. An internal standard (ethyl fluoroacetate) was added to the crude residue. The NMR yield and regioselectivity ratio (branched:linear) were analysed by ^{19}F NMR spectroscopy against the internal standard. The NMR sample was recombined with the crude residue and purification by column chromatography or preparative thin layer chromatography yielded the desired product.

3-Fluoropentadec-1-yne (1)

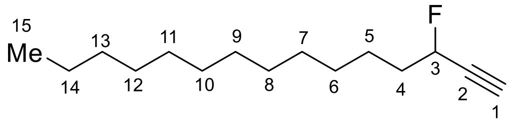

Compound **1** was prepared according to the General Procedure **B** with an amine:HF ratio of 1:5.0 using pentadeca-1,2-diene (**S1**) (20.8 mg, 0.1 mmol, 1.0 eq.). After workup, the crude mixture was analysed by ^{19}F NMR (b:l > 20:1). Purification by column chromatography (*n*-pentane) yielded the title compound **1** as a colorless oil (17.7 mg, 0.078 mmol, 78%).

$\mathbf{R}_{\boldsymbol{f}}$ = 0.65 (*n*-pentane).

^{1}H NMR (500 MHz, $CDCl_3$) δ 5.19 – 4.99 (m, 1H, H-C3), 2.65 (dd, $^4J_{HF}$ = 5.6 Hz, $^4J_{HH}$ = 2.1 Hz, 1H, H-C1), 1.94 – 1.75 (m, 2H, H-C4), 1.52 – 1.42 (m, 2H, H-C5), 1.33 – 1.25 (m, 18H, H-C6~C14), 0.88 (t, $^3J_{HH}$ = 7.0 Hz, 3H, H-C15).

^{13}C NMR (126 MHz, $CDCl_3$) δ 82.62 (d, $^1J_{CF}$ = 167.2 Hz, C3), 80.68 (d, $^2J_{CF}$ = 26.1 Hz, C2), 76.23 (d, $^3J_{CF}$ = 10.6 Hz, C1), 35.95 (d, $^2J_{CF}$ = 22.3 Hz, C4), 32.07 (CH$_2$), 29.81 (CH$_2$), 29.79 (CH$_2$), 29.77 (CH$_2$), 29.66 (CH$_2$), 29.58 (CH$_2$), 29.50 (CH$_2$), 29.23 (CH$_2$), 24.50 (d, $^3J_{CF}$ = 4.3 Hz, C5), 22.84 (CH$_2$), 14.26 (C15).

^{19}F NMR (470 MHz, $CDCl_3$) δ -175.03 (dddd, $^2J_{HF}$ = 48.2 Hz, $^3J_{HF}$ = 21.5, 19.4 Hz, $^4J_{HF}$ = 5.6 Hz, 1F, F-C3).
^{19}F{^{1}H} NMR (377 MHz, $CDCl_3$) δ -175.03 (s, 1F, F-C3).
APCI-MS: (*m/z*) requires: [($C_{15}H_{25}$)] = 205.1962 [M-H_2F], (*m/z*) found: [($C_{15}H_{25}$)] = 205.1961.
FT-IR ($\tilde{\nu}$ = cm^{-1}): 2921 (s), 2852 (s), 1457 (m), 1130 (m), 1033 (m), 797 (w), 751 (s), 637 (m).

(2-Fluorobut-3-yn-1-yl)cyclohexane (2)

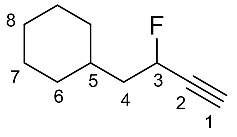

Compound **2** was prepared according to the General Procedure **B** with an amine:HF ratio of 1:4.5 using buta-2,3-dien-1-ylcyclohexane (**S2**) (27.2 mg, 0.2 mmol, 1.0 eq.). After workup, the crude mixture was analysed by ^{19}F NMR (b:l = 9:1). Purification by column chromatography (*n*-pentane) yielded the title compound **2** as a colorless oil (14.1 mg, 0.091 mmol, 46%).
R*f* = 0.60 (*n*-pentane).
^{1}H NMR (400 MHz, $CDCl_3$) δ 5.30 – 5.05 (m, 1H, H-C3), 2.66 (dd, $^4J_{HF}$ = 5.6 Hz, $^5J_{HH}$ = 2.1 Hz, 1H, H-C1), 1.93 – 1.76 (m, 2H, H-C4, H-C6), 1.75 – 1.62 (m, 5H, H-C4, H-C6, H-C7, H-C8), 1.61 – 1.53 (m, 1H, H-C5), 1.34 – 1.12 (m, 3H, H-C7, H-C8), 1.04 – 0.89 (m, 2H, H-C6).
^{13}C NMR (151 MHz, $CDCl_3$) δ 81.17 (d, $^1J_{CF}$ = 166.7 Hz, C3), 80.98 (d, $^2J_{CF}$ = 25.7 Hz, C2), 76.22 (d, $^3J_{CF}$ = 10.5 Hz, C1), 43.48 (d, $^2J_{CF}$ = 21.7 Hz, C4), 33.92 (d, $^3J_{CF}$ = 3.5 Hz, C5), 33.47 (C6), 32.95 (C6), 26.48 (C8), 26.26 (C7), 26.17 (C7).
^{19}F NMR (564 MHz, $CDCl_3$) δ -174.89 (dddd, $^2J_{HF}$ = 48.6 Hz, $^3J_{HF}$ = 26.8, 16.3 Hz, $^4J_{HF}$ = 5.6 Hz, 1F, F-C3).
^{19}F{^{1}H} NMR (377 MHz, $CDCl_3$) δ -174.89 (s, 1F, F-C3).
GC-EI-MS: (*m/z*) requires: [($C_{10}H_{14}F$)] = 153.1074 [M-H], (*m/z*) found: [($C_{10}H_{14}F$)] = 153.1074.
FT-IR ($\tilde{\nu}$ = cm^{-1}): 2973 (m), 2887 (w), 1377 (w), 1084 (s), 1044 (s), 878 (m).

12-Bromo-3-fluorododec-1-yne (3)

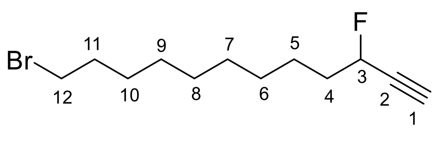

Compound **3** was prepared according to the General Procedure **B** with an amine:HF ratio of 1:5.0 using 12-bromododeca-1,2-diene (**S3**) (24.5 mg, 0.1 mmol, 1.0 eq.). After workup, the crude mixture was analysed by ^{19}F NMR (b:l > 20:1). Purification by column chromatography (*n*-pentane) yielded the title compound **3** as a colorless oil (15.9 mg, 0.060 mmol, 60%).
R*f* = 0.30 (*n*-pentane:DCM 20:1).

^{1}H NMR (500 MHz, $CDCl_3$) δ 5.19 – 5.00 (m, 1H, H-C3), 3.41 (t, $^{3}J_{HH}$ = 6.9 Hz, 2H, H-C12), 2.66 (dd, $^{4}J_{HF}$ = 5.6 Hz, $^{5}J_{HH}$ = 2.1 Hz, 1H, H-C1), 1.94 – 1.75 (m, 4H, H-C4, H-C11), 1.53 – 1.39 (m, 4H, H-C5, H-C10), 1.37 – 1.27 (m, 8H, H-C6~C9).

^{13}C NMR (126 MHz, $CDCl_3$) δ 82.59 (d, $^{1}J_{CF}$ = 167.3 Hz, C3), 80.63 (d, $^{2}J_{CF}$ = 25.8 Hz, C2), 76.28 (d, $^{3}J_{CF}$ = 10.5 Hz, C1), 35.90 (d, $^{2}J_{CF}$ = 22.4 Hz, C4), 34.14 (C12), 32.95 (C11), 29.43 (CH_2), 29.40 (CH_2), 29.15 (CH_2), 28.84 (CH_2), 28.28 (C10), 24.45 (d, $^{3}J_{CF}$ = 4.2 Hz, C5).

^{19}F NMR (470 MHz, $CDCl_3$) δ -175.05 (dddd, $^{2}J_{HF}$ = 48.2 Hz, $^{3}J_{HF}$ = 21.5, 19.6 Hz, $^{4}J_{HF}$ = 5.6 Hz, 1F, F-C3).

^{19}F{^{1}H} NMR (377 MHz, $CDCl_3$) δ -175.05 (s, 1F, F-C3).

GC-EI-MS: (*m/z*) requires: [($C_5H_{10}{}^{79}Br$)] = 150.9940 [M-$C_7H_{10}F$], (*m/z*) found: [($C_5H_{10}{}^{79}Br$)] = 150.9940.

FT-IR ($\tilde{\nu}$ = cm^{-1}): 3300 (w), 2927 (s), 2858 (m), 1463 (m), 1343 (w), 1256 (w), 1056 (m), 987 (s), 723 (m), 671 (s), 642 (s).

1,2-Difluoro-4-(((10-fluorododec-11-yn-1-yl)oxy)methyl)benzene (4)

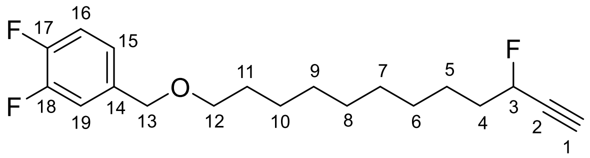

Compound **4** was prepared according to the General Procedure **B** with an amine:HF ratio of 1:5.0 using 4-((dodeca-10,11-dien-1-yloxy)methyl)-1,2-difluorobenzene (**S4**) (61.7 mg, 0.2 mmol, 1.0 eq.). After workup, the crude mixture was analysed by ^{19}F NMR (b:l > 20:1). Purification by column chromatography (*n*-pentane:Et_2O 30:1) yielded the title compound **4** as a colorless oil (50.6 mg, 0.155 mmol, 77%).

R_f = 0.30 (*n*-pentane:Et_2O 30:1).

^{1}H NMR (400 MHz, $CDCl_3$) δ 7.22 – 7.07 (m, 2H, H-C16, H-C19), 7.06 – 7.00 (m, 1H, H-C15), 5.21 – 4.95 (m, 1H, H-C3), 4.43 (s, 2H, H-C13), 3.46 (t, $^{3}J_{HH}$ = 6.6 Hz, 2H, H-C12), 2.66 (dd, $^{4}J_{HF}$ = 5.5 Hz, $^{5}J_{HH}$ = 2.1 Hz, 1H, H-C1), 1.96 – 1.74 (m, 2H, H-C4), 1.67 – 1.55 (m, 2H, H-C11), 1.54 – 1.42 (m, 2H, H-C5), 1.41 – 1.22 (m, 10H, H-C6~C10).

^{13}C NMR (126 MHz, $CDCl_3$) δ 150.51 (dd, $^{1}J_{CF}$ = 248.4 Hz, $^{2}J_{CF}$ = 12.4 Hz, *C*F), 149.83 (dd, $^{1}J_{CF}$ = 246.9 Hz, $^{2}J_{CF}$ = 12.4 Hz, *C*F), 136.00 (dd, $^{3}J_{CF}$ = 5.4 Hz, $^{4}J_{CF}$ = 4.0 Hz, C14), 123.38 (dd, $^{3}J_{CF}$ = 6.3 Hz, $^{4}J_{CF}$ = 3.7 Hz, C15), 117.16 (d, $^{2}J_{CF}$ = 17.2 Hz, C19), 116.51 (d, $^{2}J_{CF}$ = 17.3 Hz, C16), 82.60 (d, $^{1}J_{CF}$ = 167.3 Hz, C3), 80.65 (d, $^{2}J_{CF}$ = 26.1 Hz, C2), 76.26 (d, $^{3}J_{CF}$ = 10.5 Hz, C1), 71.75

(d, $^{4}J_{CF}$ = 1.4 Hz, C13), 70.87 (C12), 35.91 (d, $^{2}J_{CF}$ = 22.1 Hz, C4), 29.83 (C11), 29.54 (CH$_2$), 29.53 (CH$_2$), 29.48 (CH$_2$), 29.18 (CH$_2$), 26.29 (CH$_2$), 24.46 (d, $^{3}J_{CF}$ = 4.2 Hz, C5).

^{19}F NMR (470 MHz, $CDCl_3$) δ -138.08 (dddd, $^{3}J_{FF}$ = 21.1 Hz, $^{3}J_{HF}$ = 11.1 Hz, $^{4}J_{HF}$ = 7.9 Hz, $^{5}J_{HF}$ = 1.4 Hz, 1F, F-C18), -139.85 – -140.03 (m, 1F, F-C17), -175.03 (dddd, $^{2}J_{HF}$ = 48.2 Hz, $^{3}J_{HF}$ = 21.5, 19.6 Hz, $^{4}J_{HF}$ = 5.6 Hz, 1F, F-C3).

^{19}F{^{1}H} NMR (377 MHz, $CDCl_3$) δ -138.08 (d, $^{3}J_{FF}$ = 21.2 Hz, 1F, F-C18), -139.95 (d, $^{3}J_{FF}$ = 21.2 Hz, 1F, F-C17), -175.03 (s, 1F, F-C3).

ESI-MS: (*m/z*) requires: [$(C_{19}H_{25}OF_3Na)^+$] = 349.1750, (*m/z*) found: [$(C_{19}H_{25}OF_3Na)^+$] = 349.1748.

FT-IR ($\tilde{\nu}$ = cm^{-1}): 3306 (w), 2927 (m), 2858 (m), 1612 (w), 1521 (s), 1434 (m), 1360 (w), 1285 (s), 1211 (w), 1142 (m), 1102 (s), 987 (m), 872 (w), 815 (m), 774 (m), 734 (m), 677 (m), 637 (m).

(2-Fluorobut-3-yn-1-yl)benzene (5)

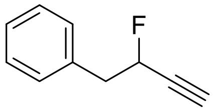

Compound **5** was prepared according to the General Procedure **B** with an amine:HF ratio of 1:5.0 using buta-2,3-dien-1-ylbenzene (**S5**) (26.0 mg, 0.2 mmol, 1.0 eq.). After workup, the crude mixture was analysed by ^{19}F NMR (71% branched product and 6% linear product, b:l = 12:1). Purification by column chromatography (*n*-pentane:Et_2O 60:1) yielded the title compound **5** and the linear propargylic fluoride as an inseparable mixture (colorless oil, 20.6 mg, 0.139 mmol, 70%, b:l = 11:1).

R$_f$ = 0.25 (*n*-pentane:Et_2O 60:1).

^{1}H NMR (500 MHz, $CDCl_3$) δ 7.36 – 7.32 (m, 2H), 7.31 – 7.27 (m, 3H), 5.40 – 5.13 (m, 1H), 3.28 – 3.07 (m, 2H), 2.69 (dd, J = 5.5, 2.1 Hz, 1H).

^{19}F{^{1}H} NMR (470 MHz, $CDCl_3$) δ -173.05 (s, 1F).

GC-EI-MS: (*m/z*) requires: [$(C_{10}H_9F)$] = 148.0688, (*m/z*) found: [$(C_{10}H_9F)$] = 148.0682.

Analytical data is in agreement with literature values.[14]

10-Fluorododec-11-yn-1-ol (6)

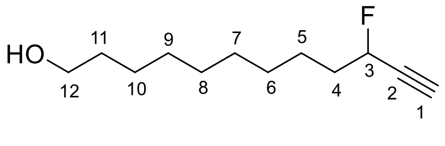

Compound **6** was prepared according to the General Procedure **B** with an amine:HF ratio of 1:5.0 using dodeca-10,11-dien-1-ol (**S6**) (36.5 mg, 0.2 mmol, 1.0 eq.). After workup, the crude mixture was analysed by ^{19}F NMR (b:l > 20:1). Purification by column chromatography (*n*-pentane:Et_2O 1:1) yielded the title compound **6** as a colorless oil (27.0 mg, 0.135 mmol, 67%).

R$_f$ = 0.35 (*n*-pentane:Et_2O 1:1).

^{1}H NMR (400 MHz, $CDCl_3$) δ 5.21 – 4.95 (m, 1H, H-C3), 3.64 (t, $^{3}J_{HH}$ = 6.6 Hz, 2H, H-C12), 2.66 (dd, $^{4}J_{HF}$ = 5.6 Hz, $^{5}J_{HH}$ = 2.1 Hz, 1H, H-C1), 1.96 – 1.72 (m, 2H, H-C4), 1.65 – 1.52 (m, 3H, H-C11, OH), 1.51 – 1.42 (m, 2H, H-C5), 1.37 – 1.26 (m, 10H, H-C6~C10).

^{13}C NMR (126 MHz, $CDCl_3$) δ 82.60 (d, $^{1}J_{CF}$ = 167.3 Hz, C3), 80.64 (d, $^{2}J_{CF}$ = 25.9 Hz, C2), 76.27 (d, $^{3}J_{CF}$ = 10.5 Hz, C1), 63.20 (C12), 35.90 (d, $^{2}J_{CF}$ = 22.0 Hz, C4), 32.92 (C11), 29.56 (CH_2), 29.50 (CH_2), 29.47 (CH_2), 29.18 (C6), 25.85 (CH_2), 24.46 (d, $^{3}J_{CF}$ = 4.1 Hz, C5).

^{19}F NMR (470 MHz, $CDCl_3$) δ -175.02 (dddd, $^{2}J_{HF}$ = 48.3 Hz, $^{3}J_{HF}$ = 21.5, 19.5 Hz, $^{4}J_{HF}$ = 5.6 Hz, 1F, F-C3).

^{19}F{^{1}H} NMR (377 MHz, $CDCl_3$) δ -175.02 (s, 1F, F-C3).

ESI-MS: (*m/z*) requires: [($C_{12}H_{21}OFNa)^+$] = 223.1469, (*m/z*) found: [($C_{12}H_{21}OFNa)^+$] = 223.1468.

FT-IR ($\tilde{\nu}$ = cm^{-1}): 3306 (w), 2927 (s), 2858 (m), 1463 (w), 1343 (w), 1050 (s), 987 (s), 637 (m).

10-Fluorododec-11-yn-1-yl 4-methylbenzenesulfonate (7)

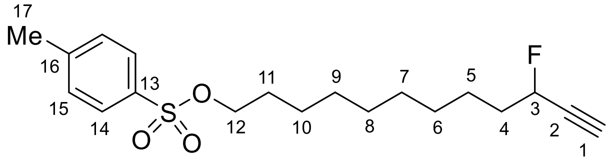

Compound **7** was prepared according to the General Procedure **B** with an amine:HF ratio of 1:5.0 using dodeca-10,11-dien-1-yl 4-methylbenzenesulfonate (**S7**) (49.6 mg, 0.15 mmol, 1.0 eq.). After workup, the crude mixture was analysed by ^{19}F NMR (b:l > 20:1). Purification by column chromatography (*n*-pentane:EA 10:1) yielded the title compound **7** as a colorless oil (36.4 mg, 0.103 mmol, 70%).

R$_f$ = 0.50 (*n*-pentane:EA 10:1).

^{1}H NMR (500 MHz, $CDCl_3$) δ 7.82 – 7.76 (m, 2H, H-C14), 7.40 – 7.30 (m, 2H, H-C15), 5.16 – 5.00 (m, 1H, H-C3), 4.02 (t, $^{3}J_{HH}$ = 6.5 Hz, 2H, H-C12), 2.66 (dd, $^{4}J_{HF}$ = 5.6 Hz, $^{5}J_{HH}$ = 2.1 Hz, 1H, H-C1), 2.45 (s, 3H, H-C17), 1.96 – 1.70 (m, 2H, H-C4), 1.68 – 1.59 (m, 2H, H-C11), 1.53 – 1.40 (m, 2H, H-C5), 1.33 – 1.21 (m, 10H, H-C6~C10).

^{13}C NMR (126 MHz, $CDCl_3$) δ 144.75 (C16), 133.43 (C13), 129.93 (C15), 128.02 (C14), 82.57 (d, $^{1}J_{CF}$ = 167.2 Hz, C3), 80.61 (d, $^{2}J_{CF}$ = 26.0 Hz, C2), 76.30 (d, $^{3}J_{CF}$ = 10.5 Hz, C1), 70.79 (C12), 35.87 (d, $^{2}J_{CF}$ = 22.1 Hz, C4), 29.36 (CH_2), 29.33 (CH_2), 29.11 (CH_2), 28.99 (CH_2), 28.95 (C11), 25.44 (C10), 24.42 (d, $^{3}J_{CF}$ = 4.2 Hz, C5), 21.76 (C17).

^{19}F NMR (470 MHz, $CDCl_3$) δ -175.06 (dddd, $^{2}J_{HF}$ = 48.2 Hz, $^{3}J_{HF}$ = 21.4, 19.7 Hz, $^{4}J_{HF}$ = 5.6 Hz, 1F, F-C3).

^{19}F{^{1}H} NMR (470 MHz, $CDCl_3$) δ -175.06 (s, 1F, F-C3).

ESI-MS: (*m/z*) requires: [$(C_{19}H_{27}O_3FSNa)^+$] = 377.1557, (*m/z*) found: [$(C_{19}H_{27}O_3FSNa)^+$] = 377.1555.

FT-IR ($\tilde{\nu}$ = cm^{-1}): 2932 (w), 1675 (s), 1389 (w), 1360 (w), 1262 (m), 1176 (m), 1090 (m), 952 (w), 734 (s), 705 (s), 660 (m).

2-(10-Fluorododec-11-yn-1-yl)isoindoline-1,3-dione (8)

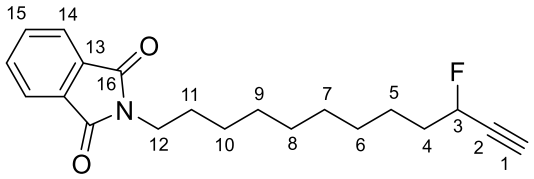

Compound **8** was prepared according to the General Procedure **B** with an amine:HF ratio of 1:5.0 using 2-(dodeca-10,11-dien-1-yl)isoindoline-1,3-dione (**S8**) (66.0 mg, 0.21 mmol, 1.0 eq.). After workup, the crude mixture was analysed by ^{19}F NMR (b:l > 20:1). Purification by column chromatography (*n*-pentane:EA 10:1) yielded the title compound **8** as a light yellow oil (38.1 mg, 0.116 mmol, 55%).

R_f = 0.43 (*n*-pentane:EA 10:1).

^{1}H NMR (400 MHz, $CDCl_3$) δ 7.84 (dd, $^3J_{HH}$ = 5.4 Hz, $^4J_{HH}$ = 3.1 Hz, 2H, H-C14), 7.70 (dd, $^3J_{HH}$ = 5.5 Hz, $^4J_{HH}$ = 3.0 Hz, 2H, H-C15), 5.19 – 4.94 (m, 1H, H-C3), 3.72 – 3.63 (m, 2H, H-C12), 2.65 (dd, $^4J_{HF}$ = 5.5 Hz, $^5J_{HH}$ = 2.1 Hz, 1H, H-C1), 1.94 – 1.74 (m, 2H, H-C4), 1.71 – 1.61 (m, 2H, H-C11), 1.52 – 1.40 (m, 2H, H-C5), 1.37 – 1.25 (m, 10H, H-C6~C10).

^{13}C NMR (151 MHz, $CDCl_3$) δ 168.60 (C16), 133.97 (C15), 132.34 (C13), 123.29 (C14), 82.59 (d, $^1J_{CF}$ = 167.1 Hz, C3), 80.65 (d, $^2J_{CF}$ = 26.0 Hz, C2), 76.27 (d, $^3J_{CF}$ = 10.5 Hz, C1), 38.18 (C12), 35.89 (d, $^2J_{CF}$ = 22.1 Hz, C4), 29.43 (CH_2), 29.23 (CH_2), 29.13 (CH_2), 28.71 (C11), 26.95 (C10), 24.43 (d, $^3J_{CF}$ = 4.1 Hz, C5).

^{19}F NMR (564 MHz, $CDCl_3$) δ -175.00 (dddd, $^2J_{HF}$ = 48.3 Hz, $^3J_{HF}$ = 21.4, 19.5 Hz, $^4J_{HF}$ = 5.6 Hz, 1F, F-C3).

^{19}F{^{1}H} NMR (564 MHz, $CDCl_3$) δ -175.00 (s, 1F, F-C3).

ESI-MS: (*m/z*) requires: [$(C_{20}H_{24}O_2FNNa)^+$] = 352.1683, (*m/z*) found: [$(C_{20}H_{24}O_2FNNa)^+$] = 352.1683.

FT-IR ($\tilde{\nu}$ = cm^{-1}): 2927 (m), 2858 (w), 1773 (w), 1710 (s), 1463 (w), 1440 (w), 1394 (s), 1366 (m), 1268 (w), 1188 (w), 1061 (m), 987 (m), 717 (s).

10-Fluorododec-11-yn-1-yl nicotinate (9)

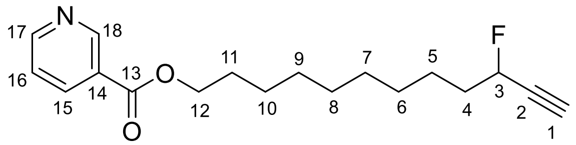

Compound **9** was prepared according to the General Procedure **B** with an amine:HF ratio of 1:5.0 using dodeca-10,11-dien-1-yl nicotinate (**S9**) (28.7 mg, 0.1 mmol, 1.0 eq.). After workup, the crude mixture was analysed by ^{19}F NMR (b:l > 20:1). Purification by column chromatography (*n*-pentane:Et_2O 1.5:1) yielded the title compound **9** as a pale yellow oil (25.1 mg, 0.082 mmol, 82%).

R*f* = 0.35 (*n*-pentane:Et_2O 1.5:1).

^{1}H NMR (400 MHz, $CDCl_3$) δ 9.22 (d, $^{4}J_{HH}$ = 1.3 Hz, 1H, H-C18), 8.77 (dd, $^{3}J_{HH}$ = 4.8 Hz, $^{4}J_{HH}$ = 1.8 Hz, 1H, H-C17), 8.29 (dt, $^{3}J_{HH}$ = 8.0 Hz, $^{4}J_{HH}$ = 2.0 Hz, 1H, H-C15), 7.39 (ddd, $^{3}J_{HH}$ = 7.9, 4.9 Hz, $^{5}J_{HH}$ = 0.9 Hz, 1H, H-C16), 5.17 – 4.96 (m, 1H, H-C3), 4.35 (t, $^{3}J_{HH}$ = 6.7 Hz, 2H, H-C12), 2.66 (dd, $^{4}J_{HF}$ = 5.6 Hz, $^{5}J_{HH}$ = 2.1 Hz, 1H, H-C1), 1.94 – 1.71 (m, 4H, H-C4, H-C11), 1.53 – 1.40 (m, 4H, H-C5, H-C10), 1.38 – 1.28 (m, 8H, H-C6~C9).

^{13}C NMR (126 MHz, $CDCl_3$) δ 165.48 (C13), 153.46 (C17), 151.03 (C18), 137.15 (C15), 126.50 (C14), 123.40 (C16), 82.57 (d, $^{1}J_{CF}$ = 167.1 Hz, C3), 80.62 (d, $^{2}J_{CF}$ = 26.0 Hz, C2), 76.28 (d, $^{3}J_{CF}$ = 10.6 Hz, C1), 65.70 (C12), 35.88 (d, $^{2}J_{CF}$ = 22.1 Hz, C4), 29.46 ($\mathit{C}H_2$), 29.44 ($\mathit{C}H_2$), 29.31 ($\mathit{C}H_2$), 29.15 ($\mathit{C}H_2$), 28.76 (C11), 26.09 (C10), 24.44 (d, $^{3}J_{CF}$ = 4.2 Hz, C5).

^{19}F NMR (470 MHz, $CDCl_3$) δ -175.05 (dddd, $^{2}J_{HF}$ = 48.3 Hz, $^{3}J_{HF}$ = 21.5, 19.6 Hz, $^{4}J_{HF}$ = 5.6 Hz, 1F, F-C3).

^{19}F{^{1}H} NMR (377 MHz, $CDCl_3$) δ -175.05 (s, 1F, F-C3).

ESI-MS: (*m/z*) requires: [$(C_{18}H_{24}O_2FNNa)^+$] = 328.1683, (*m/z*) found: [$(C_{18}H_{24}O_2FNNa)^+$] = 328.1683.

FT-IR ($\tilde{\nu}$ = cm^{-1}): 2927 (m), 2858 (w), 1721 (s), 1589 (w), 1463 (w), 1423 (w), 1279 (s), 1113 (s), 1021 (m), 987 (w), 740 (s), 700 (m).

10-Fluorododec-11-yn-1-yl 2-methylthiazole-4-carboxylate (10)

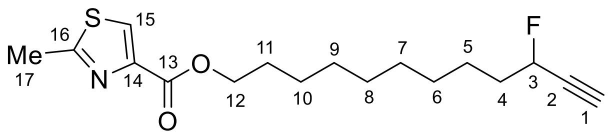

Compound **10** was prepared according to the General Procedure **B** with an amine:HF ratio of 1:5.0 using dodeca-10,11-dien-1-yl 2-methylthiazole-4-carboxylate (**S10**) (30.8 mg, 0.1 mmol, 1.0 eq.). After workup, the crude mixture was analysed by ^{19}F NMR (b:l > 20:1). Purification by column chromatography (*n*-pentane:Et_2O 1.5:1) yielded the title compound **10** as a colorless oil (25.0 mg, 0.077 mmol, 77%).

$\mathbf{R_f}$ = 0.30 (*n*-pentane:Et_2O 1.5:1).

^{1}H NMR (400 MHz, $CDCl_3$) δ 8.01 (s, 1H, H-15), 5.18 – 4.97 (m, 1H, H-C3), 4.34 (t, $^{3}J_{HH}$ = 6.9 Hz, 2H, H-C12), 2.76 (s, 3H, H-C17), 2.65 (dd, $^{4}J_{HF}$ = 5.5 Hz, $^{5}J_{HH}$ = 2.1 Hz, 1H, H-C1), 1.93 – 1.71 (m, 4H, H-C4, H-C11), 1.51 – 1.23 (m, 12H, H-C5~C10).

^{13}C NMR (126 MHz, $CDCl_3$) δ 166.89 (C16), 161.59 (C13), 147.04 (C14), 127.27 (C15), 82.58 (d, $^{1}J_{CF}$ = 167.3 Hz, C3), 80.62 (d, $^{2}J_{CF}$ = 26.2 Hz, C2), 76.28 (d, $^{3}J_{CF}$ = 10.6 Hz, C1), 65.63 (C12), 35.88 (d, $^{2}J_{CF}$ = 22.0 Hz, C4), 29.44 (CH_2), 29.43 (CH_2), 29.32 (CH_2), 29.14 (CH_2), 28.83 (C11), 25.98 (C10), 24.44 (d, $^{3}J_{CF}$ = 4.3 Hz, C5), 19.51 (C17).

^{19}F NMR (470 MHz, $CDCl_3$) δ -175.02 (dddd, $^{2}J_{HF}$ = 48.3 Hz, $^{3}J_{HF}$ = 21.5, 19.6 Hz, $^{4}J_{HF}$ = 5.5 Hz, 1F, F-C3).

^{19}F{^{1}H} NMR (377 MHz, $CDCl_3$) δ -175.02 (s, 1F, F-C3).

ESI-MS: (*m/z*) requires: [$(C_{17}H_{24}O_2FNSNa)^+$] = 348.1404, (*m/z*) found: [$(C_{17}H_{24}O_2FNSNa)^+$] = 348.1403.

FT-IR ($\tilde{\nu}$ = cm^{-1}): 3288 (w), 3093 (w), 2927 (m), 2852 (w), 1716 (s), 1469 (m), 1325 (m), 1216 (s), 1176 (s), 1107 (s), 1010 (m), 970 (s), 901 (w), 855 (w), 757 (m), 705 (m), 665 (m), 625 (w).

10-Fluorododec-11-yn-1-yl cyclopropanecarboxylate (11)

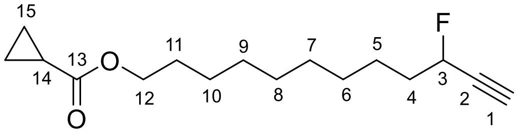

Compound **11** was prepared according to the General Procedure **B** with an amine:HF ratio of 1:5.0 using dodeca-10,11-dien-1-yl cyclopropanecarboxylate (**S11**) (25.0 mg, 0.1 mmol, 1.0 eq.). After workup, the crude mixture was analysed by ^{19}F NMR (b:l > 20:1). Purification by column chromatography (*n*-pentane:Et_2O 30:1) yielded the title compound **11** as a colorless oil (18.7 mg, 0.070 mmol, 70%).

$\mathbf{R_f}$ = 0.30 (*n*-pentane:Et_2O 30:1).

^{1}H NMR (400 MHz, $CDCl_3$) δ 5.21 – 4.97 (m, 1H, H-C3), 4.06 (t, $^{3}J_{HH}$ = 6.7 Hz, 2H, H-C12), 2.66 (dd, $^{4}J_{HF}$ = 5.6 Hz, $^{5}J_{HH}$ = 2.1 Hz, 1H, H-C1), 1.97 – 1.72 (m, 2H, H-C4), 1.68 – 1.55 (m, 3H, H-C11, H-C14), 1.53 – 1.42 (m, 2H, H-C5), 1.38 – 1.28 (m, 10H, H-C6~C10), 1.01 – 0.95 (m, 2H, H-C15), 0.86 – 0.81 (m, 2H, H-C15).

^{13}C NMR (151 MHz, $CDCl_3$) δ 175.12 (C13), 82.59 (d, $^{1}J_{CF}$ = 167.3 Hz, C3), 80.64 (d, $^{2}J_{CF}$ = 26.0 Hz, C2), 76.27 (d, $^{3}J_{CF}$ = 10.4 Hz, C1), 64.73 (C12), 35.90 (d, $^{2}J_{CF}$ = 22.2 Hz, C4), 29.47 (CH_2), 29.45 (CH_2), 29.33 (CH_2), 29.16 (CH_2), 28.81 (C11), 26.02 (CH_2), 24.46 (d, $^{3}J_{CF}$ = 4.1 Hz, C5), 13.05 (C14), 8.42 (C15).

^{19}F NMR (564 MHz, $CDCl_3$) δ -175.06 (dddd, $^2J_{HF}$ = 48.2 Hz, $^3J_{HF}$ = 21.5, 19.5 Hz, $^4J_{HF}$ = 5.6 Hz, 1F, F-C3).

^{19}F{^{1}H} NMR (377 MHz, $CDCl_3$) δ -175.06 (s, 1F, F-C3).

ESI-MS: (*m/z*) requires: [$(C_{16}H_{25}O_2FNa)^+$] = 291.1731, (*m/z*) found: [$(C_{16}H_{25}O_2FNa)^+$] = 291.1731.

FT-IR ($\tilde{\nu}$ = cm^{-1}): 2927 (s), 2858 (m), 1721 (s), 1457 (w), 1406 (m), 1371 (m), 1268 (w), 1176 (s), 1073 (m), 1033 (m), 992 (m), 901 (w), 746 (w), 671 (m).

(2-Fluorobut-3-yn-1-yl)benzene (12)

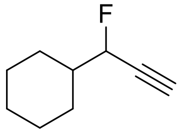

Compound **12** was prepared according to the General Procedure **B** with an amine:HF ratio of 1:5.0 using propa-1,2-dien-1-ylcyclohexane (**S12**) (24.4 mg, 0.2 mmol, 1.0 eq.). After workup, the crude mixture was analysed by ^{19}F NMR (54% branched product and 5% linear product, b:l = 11:1). Purification by column chromatography (*n*-pentane) yielded the title compound **12** as a colorless volatile liquid (6.5 mg, 0.046 mmol, 23%).

R*f* = 0.60 (*n*-pentane).

^{1}H NMR (400 MHz, $CDCl_3$) δ 4.94 – 4.78 (m, 1H), 2.66 (dd, *J* = 5.7, 2.1 Hz, 1H), 1.95 – 1.64 (m, 6H), 1.30 – 1.05 (m, 5H).

^{19}F{^{1}H} NMR (377 MHz, $CDCl_3$) δ -180.77 (s, 1F).

GC-EI-MS: (*m/z*) requires: [$(C_9H_{12}F)$] = 139.0918 [M-H], (*m/z*) found: [$(C_9H_{12}F)$] = 139.0919.

Analytical data is in agreement with literature values.[14]

(8R,9S,13S,14S)-13-Methyl-17-oxo-7,8,9,11,12,13,14,15,16,17-decahydro-6H-cyclopenta[a]phenanthren-3-yl 10-fluorododec-11-ynoate (13)

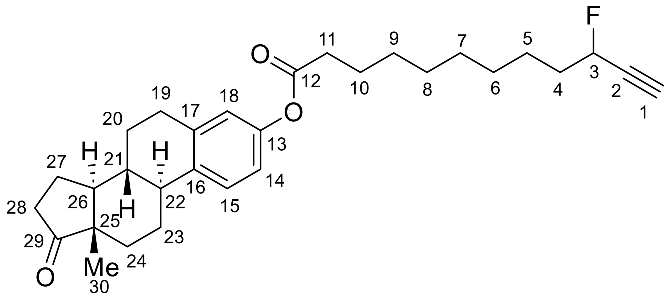

Compound **13** was prepared according to the General Procedure **B** with an amine:HF ratio of 1:5.0 using (8R,9S,13S,14S)-13-Methyl-17-oxo-7,8,9,11,12,13,14,15,16,17-decahydro-6H-cyclopenta[a]phenanthren-3-yl dodeca-10,11-dienoate (**S13**) (25.0 mg, 0.1 mmol, 1.0 eq.). After workup, the crude mixture was analysed by ^{19}F NMR (b:l > 20:1). Purification by column chromatography (*n*-pentane:EA 6:1) yielded the title compound **13** as a white wax (24.8 mg, 0.053 mmol, 53%).

R*f* = 0.30 (*n*-pentane:EA 6:1).

^{1}H NMR (599 MHz, $CDCl_3$) δ 7.28 (d, $^{3}J_{HH}$ = 8.4 Hz, 1H, H-C15), 6.84 (dd, $^{3}J_{HH}$ = 8.5 Hz, $^{4}J_{HH}$ = 2.5 Hz, 1H, H-C14), 6.80 (d, $^{4}J_{HH}$ = 2.5 Hz, 1H, H-C18), 5.14 – 5.02 (m, 1H, H-C3), 2.94 – 2.87 (m, 2H, H-C19), 2.66 (dd, $^{4}J_{HF}$ = 5.6 Hz, $^{5}J_{HH}$ = 2.1 Hz, 1H, H-C1), 2.56 – 2.47 (m, 3H, H-C11, H-C28), 2.43 – 2.37 (m, 1H, H-C23), 2.28 (td, $^{3}J_{HH}$ = 11.0, 3.9 Hz, 1H, H-C22), 2.19 – 2.10 (m, 1H, H-C28), 2.09 – 1.99 (m, 2H, H-C20, H-C27), 1.98 – 1.94 (m, 1H, H-C24), 1.93 – 1.79 (m, 2H, H-C4), 1.74 (p, $^{3}J_{HH}$ = 7.5 Hz, 2H, H-C10), 1.67 – 1.59 (m, 2H, H-C21, H-C27), 1.57 – 1.43 (m, 6H, H-C5, H-C20, H-C23, H-C24, H-C26), 1.42 – 1.30 (m, 8H, H-C6~C9), 0.91 (s, 3H, H-C30).
^{13}C NMR (151 MHz, $CDCl_3$) δ 220.89 (C29), 172.70 (C12), 148.77 (C13), 138.10 (C16), 137.41 (C17), 126.50 (C15), 121.71 (C18), 118.89 (C14), 82.57 (d, $^{1}J_{CF}$ = 167.4 Hz, C3), 80.61 (d, $^{2}J_{CF}$ = 26.2 Hz, C2), 76.30 (d, $^{3}J_{CF}$ = 10.6 Hz, C1), 50.58 (C26), 48.08 (C25), 44.29 (C22), 38.15 (C21), 35.99 (C28), 35.88 (d, $^{2}J_{CF}$ = 22.0 Hz, C4), 34.52 (C11), 31.69 (C24), 29.53 (C19), 29.34 (CH_2), 29.21 (CH_2), 29.17 (CH_2), 29.12 (CH_2), 26.48 (C20), 25.89 (C23), 25.07 (C10), 24.43 (d, $^{3}J_{CF}$ = 4.3 Hz, C5), 21.72 (C27), 13.97 (C30).
^{19}F NMR (564 MHz, $CDCl_3$) δ -175.04 (dddd, $^{2}J_{HF}$ = 48.1 Hz, $^{3}J_{HF}$ = 21.3, 19.5 Hz, $^{4}J_{HF}$ = 5.6 Hz, 1F, F-C3).
^{19}F{^{1}H} NMR (377 MHz, $CDCl_3$) δ -175.04 (s, 1F, F-C3).
ESI-MS: (*m/z*) requires: $[(C_{30}H_{39}O_3FNa)^+]$ = 489.2775, (*m/z*) found: $[(C_{30}H_{39}O_3FNa)^+]$ = 489.2780.
FT-IR ($\tilde{\nu}$ = cm^{-1}): 3214 (w), 2927 (m), 2858 (m), 1756 (s), 1733 (s), 1492 (w), 1469 (w), 1411 (w), 1377 (w), 1228 (m), 1136 (s), 1056 (m), 1010 (s), 883 (m), 820 (m), 723 (m).

10-Fluorododec-11-yn-1-yl 2-(4-isobutylphenyl)propanoate (14)

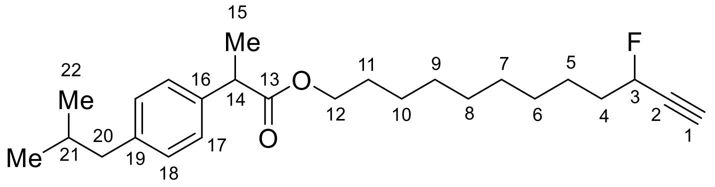

Compound **14** was prepared according to the General Procedure **B** with an amine:HF ratio of 1:5.0 using dodeca-10,11-dien-1-yl-2-(4-isobutylphenyl)propanoate (**S14**) (37.1 mg, 0.1 mmol, 1.0 eq.). After workup, the crude mixture was analysed by ^{19}F NMR (b:l > 20:1). Purification by column chromatography (*n*-pentane:Et_2O 30:1) yielded the title compound **14** as a colorless oil (26.4 mg, 0.068 mmol, 68%).
R_f = 0.35 (*n*-pentane:Et_2O 30:1).
^{1}H NMR (400 MHz, $CDCl_3$) δ 7.22 – 7.17 (m, 2H, H-C18), 7.12 – 7.05 (m, 2H, H-C17), 5.18 – 4.94 (m, 1H, H-C3), 4.05 (t, $^{3}J_{HH}$ = 6.7 Hz, 2H, H-C12), 3.68 (q, $^{3}J_{HH}$ = 7.1 Hz, 1H, H-C14), 2.66 (dd, $^{4}J_{HF}$ = 5.6 Hz, $^{5}J_{HH}$ = 2.1 Hz, 1H, H-C1), 2.45 (d, $^{3}J_{HH}$ = 7.2 Hz, 2H, H-C20), 1.95 – 1.74 (m,

3H, H-C4, H-C21), 1.62 – 1.53 (m, 2H, H-C11), 1.51 – 1.42 (m, 5H, H-C5, H-C15), 1.33 – 1.22 (m, 10H, H-C6~C10), 0.90 (d, J = 6.7 Hz, 6H, H-C22).

^{13}**C NMR** (151 MHz, $CDCl_3$) δ 174.95 (C13), 140.56 (C19), 138.06 (C16), 129.39 (C18), 127.29 (C17), 82.57 (d, $^1J_{CF}$ = 167.5 Hz, C3), 80.64 (d, $^2J_{CF}$ = 25.9 Hz, C2), 76.27 (d, $^3J_{CF}$ = 10.5 Hz, C1), 64.86 (C12), 45.35 (C14), 45.18 (C20), 35.91 (d, $^2J_{CF}$ = 22.2 Hz, C4), 30.32 (C21), 29.45 (CH_2), 29.43 (CH_2), 29.23 (CH_2), 29.17 (CH_2), 28.66 (C11), 25.88 (CH_2), 24.46 (d, $^3J_{CF}$ = 4.1 Hz, C5), 22.52 (C22), 18.61 (C15).

^{19}**F NMR** (564 MHz, $CDCl_3$) δ -175.04 (dddd, $^2J_{HF}$ = 48.2 Hz, $^3J_{HF}$ = 21.4, 19.5 Hz, $^4J_{HF}$ = 5.6 Hz, 1F, F-C3).

^{19}**F{^{1}H} NMR** (377 MHz, $CDCl_3$) δ -175.04 (s, 1F, F-C3).

ESI-MS: (*m/z*) requires: [$(C_{25}H_{37}O_2FNa)^+$] = 411.2670, (*m/z*) found: [$(C_{25}H_{37}O_2FNa)^+$] = 411.2667.

FT-IR ($\tilde{\nu}$ = cm^{-1}): 2927 (s), 2858 (m), 1733 (s), 1515 (w), 1463 (m), 1377 (w), 1331 (m), 1205 (m), 1165 (s), 1067 (m), 992 (m), 912 (m), 849 (w), 757 (s), 734 (s), 671 (m), 637 (m).

10-Fluorododec-11-yn-1-yl (E)-3-(4-bromophenyl)acrylate (15)

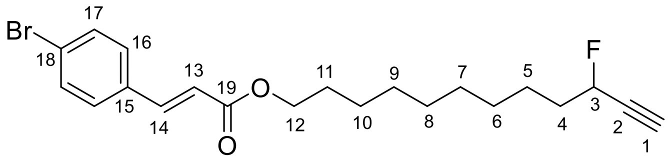

Compound **15** was prepared according to the General Procedure **B** with an amine:HF ratio of 1:5.0 using dodeca-10,11-dien-1-yl (E)-3-(4-bromophenyl)acrylate (**S15**) (39.1 mg, 0.1 mmol, 1.0 eq.). After workup, the crude mixture was analysed by ^{19}F NMR (b:l > 20:1). Purification by column chromatography (*n*-pentane:Et_2O 15:1) yielded the title compound **15** as a colorless oil (31.8 mg, 0.078 mmol, 78%).

$\mathbf{R_f}$ = 0.30 (*n*-pentane:Et_2O 15:1).

^{1}**H NMR** (599 MHz, $CDCl_3$) δ 7.60 (d, $^3J_{HH}$ = 16.0 Hz, 1H, H-C13), 7.54 – 7.48 (m, 2H, H-C16), 7.43 – 7.34 (m, 2H, H-C17), 6.42 (d, $^3J_{HH}$ = 16.0 Hz, 1H, H-C14), 5.16 – 5.01 (m, 1H, H-C3), 4.19 (t, $^3J_{HH}$ = 6.7 Hz, 2H, H-C12), 2.66 (dd, $^4J_{HF}$ = 5.6 Hz, $^5J_{HH}$ = 2.1 Hz, 1H, H-C1), 1.94 – 1.76 (m, 2H, H-C4), 1.73 – 1.66 (m, 2H, H-C11), 1.54 – 1.44 (m, 2H, H-C5), 1.42 – 1.37 (m, 2H, H-C10), 1.36 – 1.29 (m, 8H, H-C6~C9).

^{13}**C NMR** (151 MHz, $CDCl_3$) δ 166.95 (C19), 143.31 (C13), 133.54 (C15), 132.27 (C16), 129.56 (C17), 124.60 (C18), 119.14 (C14), 82.58 (d, $^1J_{CF}$ = 167.4 Hz, C3), 80.63 (d, $^2J_{CF}$ = 26.1 Hz, C2), 76.28 (d, $^3J_{CF}$ = 10.6 Hz, C1), 64.96 (C12), 35.90 (d, $^2J_{CF}$ = 22.1 Hz, C4), 29.48 (CH_2), 29.45 (CH_2), 29.34 (CH_2), 29.16 (CH_2), 28.83 (C11), 26.07 (C10), 24.45 (d, $^3J_{CF}$ = 4.1 Hz, C5).

^{19}F NMR (564 MHz, $CDCl_3$) δ -175.05 (dddd, $^{2}J_{HF}$ = 48.2 Hz, $^{3}J_{HF}$ = 21.5, 19.5 Hz, $^{4}J_{HF}$ = 5.6 Hz, 1F, F-C3).

^{19}F{^{1}H} NMR (564 MHz, $CDCl_3$) δ -175.05 (s, 1F, F-C3).

ESI-MS: (*m/z*) requires: [($C_{21}H_{26}O_2F^{79}BrNa)^+$] = 431.0992, (*m/z*) found: [($C_{21}H_{26}O_2F^{79}BrNa)^+$] = 431.0996.

FT-IR ($\tilde{\nu}$ = cm^{-1}): 3237 (w), 2921 (m), 2852 (m), 1704 (s), 1635 (m), 1584 (w), 1486 (m), 1400 (w), 1314 (s), 1268 (m), 1170 (s), 1067 (s), 1004 (m), 981 (s), 820 (s), 694 (m).

1-(1-Fluoroprop-2-yn-1-yl)-4-(trifluoromethyl)benzene (16)

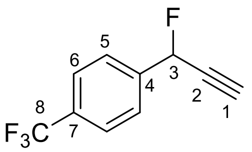

Compound 1**6** was prepared according to the General Procedure **B** with an amine:HF ratio of 1:4.5 using 1-(propa-1,2-dien-1-yl)-4-(trifluoromethyl)benzene (**S16**) (36.8 mg, 0.2 mmol, 1.0 eq.). The solvent was changed to DCE. After workup, the crude mixture was analysed by ^{19}F NMR (65% branched product and 6% linear product, b:l = 11:1). Purification by column chromatography (*n*-pentane) yielded the title compound **16** and the linear propargylic fluoride as an inseparable mixture (colorless volatile liquid, 12.1 mg, 0.060 mmol, 30%, b:l = 9:1).

R*f* = 0.35 (*n*-pentane).

^{1}H NMR (400 MHz, $CDCl_3$) δ 7.73 – 7.63 (m, 4H, H-C5, H-C6), 6.20 – 6.03 (m, 1H, H-C3), 2.92 (dd, $^{4}J_{HF}$ = 5.6 Hz, $^{5}J_{HH}$ = 2.2 Hz, 1H, H-C1).

^{13}C NMR (126 MHz, $CDCl_3$) δ 140.08 (app. C4), 127.36 (d, $^{3}J_{CF}$ = 4.8 Hz, C5), 125.91 (q, $^{3}J_{CF}$ = 3.9 Hz, C6), 82.08 (d, $^{1}J_{CF}$ = 170.4 Hz, C3), 79.24 (d, $^{3}J_{CF}$ = 10.1 Hz, C1), 78.75 (d, $^{2}J_{CF}$ = 27.4 Hz, C2).

^{19}F NMR (470 MHz, $CDCl_3$) δ -62.83 (s, 3F, F-C8), -163.00 (dd, $^{3}J_{HF}$ = 47.9 Hz, $^{4}J_{HF}$ = 5.6 Hz, 1F, F-C3).

^{19}F{^{1}H} NMR (470 MHz, $CDCl_3$) δ -62.83 (d, $^{7}J_{FF}$ = 1.9 Hz, 3F, F-C8), -163.00 (q, $^{7}J_{FF}$ = 1.9 Hz, 1F, F-C3).

GC-EI-MS: (*m/z*) requires: [($C_{10}H_6F_4$)] = 202.0406, (*m/z*) found: [($C_{10}H_6F_4$)] = 202.0400.

FT-IR ($\tilde{\nu}$ = cm^{-1}): 2973 (m), 2364 (w), 1067 (s).

1-(1-Fluoroprop-2-yn-1-yl)-3,5-bis(trifluoromethyl)benzene (17)

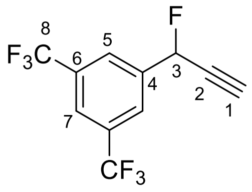

Compound 17 was prepared according to the General Procedure **B** with an amine:HF ratio of 1:5.5 using 1-(propa-1,2-dien-1-yl)-3,5-bis(trifluoromethyl)benzene (**S17**) (25.2 mg, 0.1 mmol, 1.0 eq.). The solvent was changed to DCE. After workup, the crude mixture was analysed by ^{19}F NMR (47% branched product and 7% linear product, b:l = 7:1). Purification by column chromatography (*n*-pentane) yielded the title compound **17** and the linear propargylic fluoride as an inseparable mixture (colorless volatile liquid, 12.8 mg, 0.047 mmol, 47%, b:l = 6:1).

$\mathbf{R_f}$ = 0.55 (*n*-pentane).

^{1}H NMR (400 MHz, $CDCl_3$) δ 8.00 (s, 2H, H-C5), 7.94 (s, 1H, H-C7), 6.37 – 6.01 (m, 1H, H-C3), 2.99 (dd, $^4J_{HF}$ = 5.7 Hz, $^5J_{HH}$ = 2.2 Hz, 1H, H-C1).

^{13}C NMR (126 MHz, $CDCl_3$) δ 138.85 (d, $^2J_{CF}$ = 22.1 Hz, C4), 132.48 (q, $^2J_{CF}$ = 33.9 Hz, C6), 127.21 (m, C5), 123.62 (m, C7), 123.12 (q, $^1J_{CF}$ = 273.1 Hz, C8), 81.33 (d, $^1J_{CF}$ = 172.5 Hz, C3), 80.20 (d, $^3J_{CF}$ = 10.1 Hz, C1), 77.84 (d, $^2J_{CF}$ = 27.1 Hz, C2).

^{19}F NMR (470 MHz, $CDCl_3$) δ -62.99 (s, 6F, F-C8), -164.10 (dd, $^3J_{HF}$ = 47.5 Hz, $^4J_{HF}$ = 5.8 Hz, 1F, F-C3).

^{19}F{^{1}H} NMR (377 MHz, $CDCl_3$) δ -62.99 (s, 6F, F-C8), -164.10 (s, 1F, F-C3).

GC-EI-MS: (*m/z*) requires: [($C_{11}H_5F_7$)] = 270.0279, (*m/z*) found: [($C_{11}H_5F_7$)] = 270.0275.

FT-IR ($\tilde{\nu}$ = cm^{-1}): 2978 (m), 2359 (m), 1406 (m), 1383 (m), 1228 (w), 1067 (s), 901 (w), 723 (w).

Methyl 4-(1-fluoroprop-2-yn-1-yl)benzoate (18)

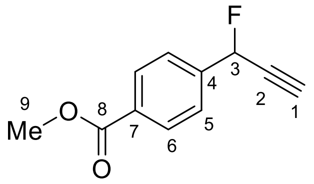

Compound **18** was prepared according to the General Procedure **B** with an amine:HF ratio of 1:5.0 using methyl 4-(propa-1,2-dien-1-yl)benzoate (**S18**) (34.8 mg, 0.2 mmol, 1.0 eq.). The solvent was changed to DCE. After workup, the crude mixture was analysed by ^{19}F NMR (b:l = 13:1). Purification by column chromatography (*n*-pentane:Et_2O 10:1) yielded the title compound **18** as a colorless oil (20.2 mg, 0.105 mmol, 53%).

$\mathbf{R_f}$ = 0.30 (*n*-pentane:Et_2O 10:1).

^{1}H NMR (400 MHz, $CDCl_3$) δ 8.12 – 8.06 (m, 2H, H-C6), 7.64 – 7.59 (m, 2H, H-C5), 6.20 – 6.02 (m, 1H, H-C3), 3.93 (s, 3H, H-C9), 2.91 (dd, $^4J_{HF}$ = 5.6 Hz, $^5J_{HH}$ = 2.2 Hz, 1H, H-C1).

^{13}**C NMR** (151 MHz, $CDCl_3$) δ 166.59 (d, $^{6}J_{CF}$ = 1.1 Hz, C8), 140.86 (d, $^{2}J_{CF}$ = 21.0 Hz, C4), 131.35 (d, $^{5}J_{CF}$ = 2.5 Hz, C7), 130.15 (d, $^{4}J_{CF}$ = 0.9 Hz, C6), 126.96 (d, $^{3}J_{CF}$ = 4.7 Hz, C5), 82.26 (d, $^{1}J_{CF}$ = 170.0 Hz, C3), 79.10 (d, $^{3}J_{CF}$ = 10.1 Hz, C1), 78.89 (d, $^{2}J_{CF}$ = 27.7 Hz, C2), 52.43 (C9).
^{19}**F NMR** (564 MHz, $CDCl_3$) δ -162.75 (ddt, $^{3}J_{HF}$ = 47.7 Hz, $^{4}J_{HF}$ = 5.7, 1.4 Hz, 1F, F-C3).
^{19}**F{^{1}H} NMR** (564 MHz, $CDCl_3$) δ -162.75 (s, 1F, F-C3).
GC-EI-MS: (*m/z*) requires: [($C_{11}H_9FO_2$)] = 192.0587, (*m/z*) found: [($C_{11}H_9FO_2$)] = 192.0582.
FT-IR ($\tilde{\nu}$ = cm^{-1}): 3237 (w), 2955 (w), 2123 (w), 1716 (s), 1612 (w), 1434 (m), 1411 (w), 1274 (s), 1193 (m), 1107 (s), 1015 (m), 952 (m), 866 (m), 832 (m), 809 (m), 751 (m), 700 (m).

(4-(1-Fluoroprop-2-yn-1-yl)phenyl)(morpholino)methanone (19)

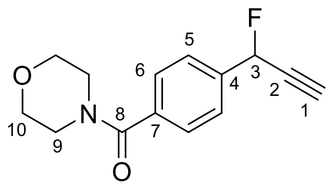

Compound **19** was prepared according to the General Procedure **B** with an amine:HF ratio of 1:5.0 using morpholino(4-(propa-1,2-dien-1-yl)phenyl)methanone (**S19**) (22.9 mg, 0.1 mmol, 1.0 eq.). The solvent was changed to DCE. After workup, the crude mixture was analysed by ^{19}F NMR (50% branched product and 3% linear product, b:l = 17:1). Purification by column chromatography (*n*-pentane:EA 1:1.5-1:2) yielded the title compound **19** and the linear propargylic fluoride as an inseparable mixture (colorless oil, 13.1 mg, 0.053 mmol, 53%, b:l = 21:1).
R*f* = 0.30 (*n*-pentane:EA 1:1.5).
^{1}**H NMR** (400 MHz, $CDCl_3$) δ 7.64 – 7.57 (m, 2H, H-C5), 7.49 – 7.43 (m, 2H, H-C6), 6.16 – 5.98 (m, 1H, H-C3), 3.79 – 3.41 (m, 8H, H-C9, H-C10), 2.91 (dd, $^{4}J_{HF}$ = 5.6 Hz, $^{5}J_{HH}$ = 2.2 Hz, 1H, H-C1).
^{13}**C NMR** (151 MHz, $CDCl_3$) δ 169.76 (C8), 137.94 (d, $^{2}J_{CF}$ = 21.2 Hz, C4), 136.74 (d, $^{5}J_{CF}$ = 2.9 Hz, C7), 127.65 (d, $^{4}J_{CF}$ = 1.3 Hz, C6), 127.42 (d, $^{3}J_{CF}$ = 4.4 Hz, C5), 82.32 (d, $^{1}J_{CF}$ = 169.5 Hz, C3), 79.01 (d, $^{3}J_{CF}$ = 10.0 Hz, C1), 78.97 (d, $^{2}J_{CF}$ = 28.0 Hz, C2), 66.99 (C9, C10).
^{19}**F NMR** (564 MHz, $CDCl_3$) δ -160.39 – -160.55 (m, 1F, F-C3).
^{19}**F{^{1}H} NMR** (377 MHz, $CDCl_3$) δ -160.46 (s, 1F, F-C3).
ESI-MS: (*m/z*) requires: [($C_{14}H_{14}O_2NFNa)^+$] = 270.0901, (*m/z*) found: [($C_{14}H_{14}O_2NFNa)^+$] = 270.0899.
FT-IR ($\tilde{\nu}$ = cm^{-1}): 2858 (w), 1630 (s), 1429 (s), 1274 (s), 1256 (m), 1153 (w), 1113 (s), 1067 (m), 1010 (s), 941 (m), 895 (w), 843 (m), 763 (m).

3-(1-Fluoroprop-2-yn-1-yl)phenyl trifluoromethanesulfonate (20)

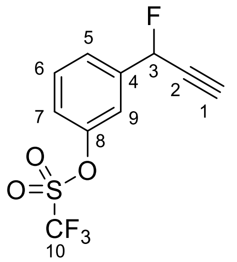

Compound **20** was prepared according to the General Procedure **B** with an amine:HF ratio of 1:5.0 using methyl 4-((dodeca-10,11-dien-1-yloxy)methyl)-1,2-difluorobenzene (**S20**) (26.4 mg, 0.1 mmol, 1.0 eq.). The solvent was changed to DCE. After workup, the crude mixture was analysed by ^{19}F NMR (b:l = 9:1). Purification by column chromatography (*n*-pentane:Et_2O 50:1) yielded the title compound **20** as a colorless oil (21.2 mg, 0.075 mmol, 75%).

R*f* = 0.30 (*n*-pentane:Et_2O 50:1).

^{1}H NMR (400 MHz, $CDCl_3$) δ 7.61 – 7.56 (m, 1H, H-C5), 7.53 (t, $^3J_{HH}$ = 7.9 Hz, 1H, H-C6), 7.50 – 7.46 (m, 1H, H-C9), 7.40 – 7.29 (m, 1H, H-C7), 6.26 – 5.96 (m, 1H, H-C3), 2.94 (dd, $^4J_{HF}$ = 5.7 Hz, $^5J_{HH}$ = 2.2 Hz, 1H, H-C1).

^{13}C NMR (126 MHz, $CDCl_3$) δ 149.75 (d, $^4J_{CF}$ = 1.0 Hz, C8), 139.19 (d, $^2J_{CF}$ = 21.9 Hz, C4), 130.83 (d, $^4J_{CF}$ = 1.0 Hz, C6), 126.98 (d, $^3J_{CF}$ = 4.6 Hz, C5), 122.57 (d, $^5J_{CF}$ = 2.5 Hz, C7), 120.11 (d, $^3J_{CF}$ = 4.9 Hz, C9), 118.89 (q, $^1J_{CF}$ = 320.9 Hz, C10), 81.62 (d, $^1J_{CF}$ = 171.1 Hz, C3), 79.47 (d, $^3J_{CF}$ = 10.0 Hz, C1), 78.36 (d, $^2J_{CF}$ = 27.5 Hz, C2).

^{19}F NMR (470 MHz, $CDCl_3$) δ -72.80 (s, 3F, F-C10), -162.31 (ddt, $^3J_{HF}$ = 47.7 Hz, $^4J_{HF}$ = 5.8, 1.3 Hz, 1F, F-C3).

^{19}F{^{1}H} NMR (377 MHz, $CDCl_3$) δ -72.80 (s, 3F, F-C10), -162.31 (s, 1F, F-C3).

GC-EI-MS: (*m/z*) requires: [($C_{10}H_6F_4O_3S$)] = 281.9974, (*m/z*) found: [($C_{10}H_6F_4O_3S$)] = 281.9968.

FT-IR ($\tilde{\nu}$ = cm^{-1}): 3300 (w), 1589 (w), 1486 (w), 1423 (s), 1205 (s), 1136 (s), 1010 (m), 975 (m), 889 (s), 832 (s), 797 (m), 746 (m), 688 (m), 654 (m).

1-(1-Fluoroprop-2-yn-1-yl)-4-(methylsulfonyl)benzene (21)

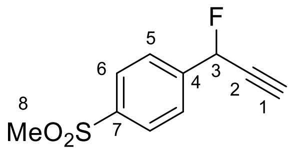

Compound **21** was prepared according to the General Procedure **B** with an amine:HF ratio of 1:5.0 using 1-(methylsulfonyl)-4-(propa-1,2-dien-1-yl)benzene (**S21**) (19.8 mg, 0.1 mmol, 1.0 eq.). After workup, the crude mixture was analysed by ^{19}F NMR (b:l = 14:1). Purification by column chromatography (*n*-pentane:EA 4:1) yielded the title compound **21** as a colorless oil (11.4 mg, 0.054 mmol, 54%).

R*f* = 0.23 (*n*-pentane:EA 10:3).

^{1}H NMR (500 MHz, $CDCl_3$) δ 8.07 – 7.97 (m, 2H, H-C6), 7.81 – 7.71 (m, 2H, H-C5), 6.23 – 6.04 (m, 1H, H-C3), 3.07 (s, 3H, H-C8), 2.94 (dd, $^4J_{HF}$ = 5.7 Hz, $^5J_{HH}$ = 2.2 Hz, 1H, H-C1).

^{13}C NMR (126 MHz, $CDCl_3$) δ 142.09 (d, $^2J_{CF}$ = 21.3 Hz, C4), 141.73 (d, $^5J_{CF}$ = 2.4 Hz, C7), 128.09 (C6), 127.82 (d, $^3J_{CF}$ = 5.1 Hz, C5), 81.78 (d, $^1J_{CF}$ = 171.6 Hz, C3), 79.64 (d, $^3J_{CF}$ = 10.2 Hz, C1), 78.41 (d, $^2J_{CF}$ = 27.5 Hz, C2), 44.63 (C8).

^{19}F NMR (470 MHz, $CDCl_3$) δ -164.86 (ddt, $^3J_{HF}$ = 47.4 Hz, $^4J_{HF}$ = 5.7, 1.2 Hz, 1F, F-C3).

^{19}F{^{1}H} NMR (470 MHz, $CDCl_3$) δ -164.86 (s, 1F, F-C3).

ESI-MS: (*m/z*) requires: [($C_{10}H_9O_2FSNa$)$^+$] = 235.0199, (*m/z*) found: [($C_{10}H_9O_2FSNa$)$^+$] = 235.0199.

FT-IR ($\tilde{\nu}$ = cm^{-1}): 3294 (w), 2927 (w), 2852 (w), 1406 (w), 1302 (s), 1193 (w), 1147 (s), 1090 (m), 1010 (m), 952 (s), 849 (w), 763 (s), 734 (m), 688 (m).

(1R,2S,5R)-2-Isopropyl-5-methylcyclohexyl 4-(1-fluoroprop-2-yn-1-yl)benzoate (22)

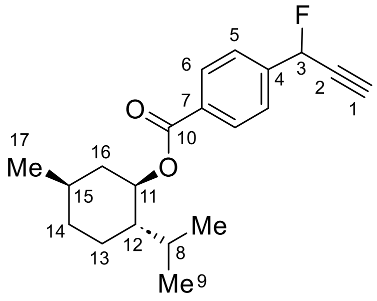

Compound **22** was prepared according to the General Procedure **B** with an amine:HF ratio of 1:5.0 using (1R,2S,5R)-2-isopropyl-5-methylcyclohexyl 4-(propa-1,2-dien-1-yl)benzoate (**S22**) (29.8 mg, 0.1 mmol, 1.0 eq.). The solvent was changed to DCE. After workup, the crude mixture was analysed by ^{19}F NMR (b:l = 12:1). Purification by column chromatography (*n*-pentane:Et_2O 30:1) yielded the title compound **22** as a colorless oil (18.4 mg, 0.058 mmol, 58%).

Two sets of peaks were observed in ^{13}C and ^{19}F NMR spectra due to diastereoisomers.

R_f = 0.35 (*n*-pentane:Et_2O 30:1).

^{1}H NMR (400 MHz, $CDCl_3$) δ 8.18 – 7.97 (m, 2H, H-C6), 7.69 – 7.54 (m, 2H, H-C5), 6.28 – 6.00 (m, 1H, H-C3), 4.94 (d, $^3J_{HH}$ = 10.9, 4.4 Hz, 1H, H-C11), 2.90 (dd, $^4J_{HF}$ = 5.6 Hz, $^5J_{HH}$ = 2.2 Hz, 1H, H-C1), 2.16 – 2.08 (m, 1H, H-C16), 2.00 – 1.88 (m, 1H, H-C15), 1.78 – 1.69 (m, 2H, H-C13, H-C14), 1.61 – 1.54 (m, 2H, H-C8, H-C12), 1.19 – 1.06 (m, 2H, H-C13, H-C16), 0.99 – 0.88 (m, 7H, H-C9, H-C14), 0.79 (d, $^3J_{HH}$ = 6.9 Hz, 3H, H-C17).

^{13}C NMR (151 MHz, $CDCl_3$) δ 165.59 (C10), 140.69 (d, $^2J_{CF}$ = 21.0 Hz, C4), 140.67 (d, $^2J_{CF}$ = 21.0 Hz, C4'), 132.10 (d, $^5J_{CF}$ = 2.5 Hz, C7), 132.08 (d, $^5J_{CF}$ = 2.5 Hz, C7'), 130.14 (C6), 126.94 (d, $^3J_{CF}$ = 4.6 Hz, C5), 126.89 (d, $^3J_{CF}$ = 4.6 Hz, C5'), 82.31 (d, $^1J_{CF}$ = 170.0 Hz, C3), 82.30 (d, $^1J_{CF}$ = 170.0 Hz, C3'), 79.03 (d, $^3J_{CF}$ = 10.1 Hz, C1), 79.03 (d, $^3J_{CF}$ = 10.1 Hz, C1'), 78.98 (d, $^2J_{CF}$ = 27.8 Hz, C2), 75.34 (C11), 47.42 (C12), 41.09 (C16), 34.45 (C14), 31.60 (C8), 26.71 (C15), 23.81 (C13), 22.18 (C9), 20.90 (C9), 16.69 (C17).

^{19}F NMR (377 MHz, $CDCl_3$) δ -162.35 (ddt, $^3J_{HF}$ = 47.6 Hz, $^4J_{HF}$ = 5.7, 1.4 Hz, 1F, F-C3), -162.68 (ddt, $^3J_{HF}$ = 47.6 Hz, $^4J_{HF}$ = 5.7, 1.4 Hz, 1F, F-C3').
^{19}F{^{1}H} NMR (377 MHz, $CDCl_3$) δ -162.35 (s, 1F, F-C3), -162.68 (s, 1F, F-C3').
ESI-MS: (*m/z*) requires: [$(C_{20}H_{25}O_2FNa)^+$] = 339.1731, (*m/z*) found: [$(C_{20}H_{25}O_2FNa)^+$] = 339.1730.
FT-IR ($\tilde{\nu}$ = cm^{-1}): 2955 (m), 2869 (w), 1710 (s), 1457 (w), 1411 (w), 1371 (w), 1268 (s), 1176 (m), 1107 (s), 1015 (m), 958 (m), 860 (w), 769 (w), 705 (w).

Methyl 4-(1-chloro-2-fluorobut-3-yn-2-yl)benzoate (23)

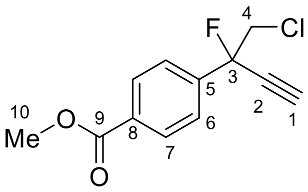

Compound **23** was prepared according to the General Procedure **B** with an amine:HF ratio of 1:5.0 using methyl 4-(1-chlorobuta-2,3-dien-2-yl)benzoate (**S23**) (22.3 mg, 0.1 mmol, 1.0 eq.). The crude product was purified by column chromatography (*n*-pentane:Et_2O 8:1) to yield the title compound as a colorless oil (18.7 mg, 0.078 mmol, 78%).
R_f = 0.30 (*n*-pentane:Et_2O 8:1).
^{1}H NMR (400 MHz, $CDCl_3$) δ 8.09 (d, $^3J_{HH}$ = 7.9 Hz, 2H, H-C7), 7.64 (d, $^3J_{HH}$ = 8.2 Hz, 2H, H-C6), 3.94 (s, 3H, H-C10), 3.90 (d, $^3J_{HF}$ = 16.4 Hz, 2H), 2.98 (d, $^4J_{HF}$ = 5.3 Hz, 1H, H-C1).
^{13}C NMR (151 MHz, $CDCl_3$) δ 166.49 (C9), 141.97 (d, $^2J_{CF}$ = 23.5 Hz, C5), 131.34 (d, $^5J_{CF}$ = 1.5 Hz, C8), 129.94 (C7), 125.70 (d, $^3J_{CF}$ = 6.5 Hz, C6), 90.73 (d, $^1J_{CF}$ = 180.7 Hz, C3), 79.11 (d, $^2J_{CF}$ = 28.9 Hz, C2), 79.04 (d, $^3J_{CF}$ = 9.0 Hz, C1), 52.47 (C10), 50.86 (d, $^2J_{CF}$ = 31.1 Hz, C4).
^{19}F NMR (564 MHz, $CDCl_3$) δ -144.38 (td, $^3J_{HF}$ = 16.5 Hz, $^4J_{HF}$ = 5.4 Hz, 1F, F-C3).
^{19}F{^{1}H} NMR (564 MHz, $CDCl_3$) δ -144.38 (s, 1F, F-C3).
GC-EI-MS: (*m/z*) requires: [$(C_{12}H_{10}FClO_2)$] = 240.0353, (*m/z*) found: [$(C_{12}H_{10}FClO_2)$] = 240.0349.
FT-IR ($\tilde{\nu}$ = cm^{-1}): 3283 (m), 2955 (w), 1710 (s), 1612 (w), 1578 (w), 1434 (m), 1411 (m), 1268 (s), 1188 (m), 1113 (s), 1067 (s), 992 (m), 964 (m), 866 (m), 826 (w), 763 (s), 728 (m), 700 (s), 660 (s), 614 (m).

1-(1-Chloro-2-fluorobut-3-yn-2-yl)-4-(trifluoromethyl)benzene (24)

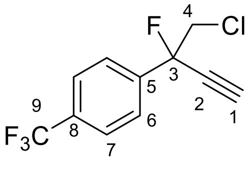

Compound **24** was prepared according to the General Procedure **B** with an amine:HF ratio of 1:5.5 using 1-(1-chlorobuta-2,3-dien-2-yl)-4-(trifluoromethyl)benzene (**S24**) (23.3 mg, 0.1 mmol, 1.0 eq.). The crude product was purified by column chromatography (*n*-pentane:Et_2O 80:1) to

yield the title compound as a colorless oil (17.3 mg, 0.069 mmol, 69%).

R*f* = 0.35 (*n*-pentane:Et_2O 80:1).

^{1}H NMR (500 MHz, $CDCl_3$) δ 7.77 – 7.63 (m, 4H, H-C6, H-C7), 3.94 – 3.87 (m, 2H, H-C4), 2.99 (d, $^4J_{HF}$ = 5.4 Hz, 1H, H-C1).

^{13}C NMR (126 MHz, $CDCl_3$) δ 141.20 (d, $^2J_{CF}$ = 23.9 Hz, C5), 131.81 (app. q, $^2J_{CF}$ = 32.6 Hz, C8), 126.16 (d, $^3J_{CF}$ = 6.6 Hz, C6), 125.72 (q, $^3J_{CF}$ = 3.7 Hz, C7), 123.90 (q, $^1J_{CF}$ = 272.4 Hz, C9), 90.53 (d, $^1J_{CF}$ = 180.7 Hz, C3), 79.17 (d, $^3J_{CF}$ = 9.5 Hz, C1), 79.02 (d, $^2J_{CF}$ = 29.5 Hz, C2), 50.80 (d, $^2J_{CF}$ = 31.3 Hz, C4).

^{19}F NMR (470 MHz, $CDCl_3$) δ -62.84 (s, 3F, F-C9), -144.35 (td, $^3J_{HF}$ = 16.3 Hz, $^4J_{HF}$ = 5.4 Hz, 1F, F-C3).

^{19}F{^{1}H} NMR (377 MHz, $CDCl_3$) δ -62.84 (s, 3F, F-C9), -144.34 (s, 1F, F-C3).

GC-EI-MS: (*m/z*) requires: [($C_{11}H_7ClF_4$)] = 250.0172, (*m/z*) found: [($C_{11}H_7ClF_4$)] = 250.0164.

FT-IR ($\tilde{\nu}$ = cm^{-1}): 3300 (w), 2967 (w), 1618 (w), 1411 (m), 1325 (s), 1165 (s), 1124 (s), 1067 (s), 1021 (m), 998 (m), 843 (s), 763 (m), 688 (m), 665 (s), 625 (m).

(2-(Chloromethyl)-2-fluorobut-3-yn-1-yl)benzene (25)

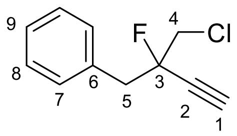

Compound **25** was prepared according to the General Procedure **B** with an amine:HF ratio of 1:6.0 using (2-(chloromethyl)buta-2,3-dien-1-yl)benzene (**S25**) (17.9 mg, 0.1 mmol, 1.0 eq.) over 48 h. The crude product was purified by column chromatography (*n*-pentane:Et_2O 70:1) to yield the title compound as a colorless oil (9.8 mg, 0.050 mmol, 50%).

R*f* = 0.30 (*n*-pentane:Et_2O 70:1).

^{1}H NMR (500 MHz, $CDCl_3$) δ 7.37 – 7.30 (m, 5H, H-C7~C9), 3.73 – 3.58 (m, 2H, H-C4), 3.35 – 3.23 (m, 2H, H-C5), 2.76 (d, $^4J_{HF}$ = 5.1 Hz, 1H, H-C1).

^{13}C NMR (126 MHz, $CDCl_3$) δ 133.68 (d, $^3J_{CF}$ = 4.6 Hz, C6), 130.75 (d, $^4J_{CF}$ = 1.0 Hz, C7), 128.50 (C8), 127.68 (C9), 90.37 (d, $^1J_{CF}$ = 178.6 Hz, C3), 79.68 (d, $^2J_{CF}$ = 28.5 Hz, C2), 78.29 (d, $^3J_{CF}$ = 9.0 Hz, C1), 47.68 (d, $^2J_{CF}$ = 29.8 Hz, C4), 42.92 (d, $^2J_{CF}$ = 24.1 Hz, C5).

^{19}F NMR (470 MHz, $CDCl_3$) δ -143.35 (dtdd, $^3J_{HF}$ = 18.6, 17.5, 12.3 Hz, $^4J_{HF}$ = 5.1 Hz, 1F, F-C3).

^{19}F{^{1}H} NMR (377 MHz, $CDCl_3$) δ -143.33 (s, 1F, F-C3).

GC-EI-MS: (*m/z*) requires: [($C_{11}H_{10}FCl$)] = 196.0455, (*m/z*) found: [($C_{11}H_{10}FCl$)] = 196.0450.

FT-IR ($\tilde{\nu}$ = cm^{-1}): 3294 (w), 1498 (w), 1452 (w), 1222 (m), 1079 (m), 1027 (s), 855 (m), 763 (m), 694 (s), 660 (s).

3-(Chloromethyl)-3-fluoropentadec-1-yne (26)

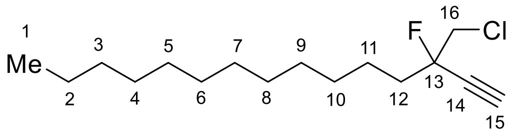

Compound **26** was prepared according to the General Procedure **B** with an amine:HF ratio of 1:5.5 using 3-(chloromethyl)pentadeca-1,2-diene (**S26**) (25.7 mg, 0.1 mmol, 1.0 eq.) over 48 h. The crude product was purified by column chromatography (*n*-pentane) to yield the title compound as a colorless oil (20.8 mg, 0.076 mmol, 76%).

R*f* = 0.50 (*n*-pentane).

^{1}H NMR (599 MHz, $CDCl_3$) δ 3.84 – 3.61 (m, 2H, H-C16), 2.72 (d, $^{4}J_{HF}$ = 5.0 Hz, 1H, H-15), 2.04 – 1.77 (m, 2H, H-C12), 1.57 – 1.50 (m, 2H, H-C11), 1.36 – 1.25 (m, 18H, H-C2~C10), 0.88 (t, $^{3}J_{HH}$ = 7.0 Hz, 3H, H-C1).

^{13}C NMR (151 MHz, $CDCl_3$) δ 90.89 (d, $^{1}J_{CF}$ = 176.2 Hz, C13), 79.95 (d, $^{2}J_{CF}$ = 29.2 Hz, C14), 77.01 (d, $^{3}J_{CF}$ = 9.0 Hz, C15), 48.85 (d, $^{2}J_{CF}$ = 29.5 Hz, C16), 37.20 (d, $^{2}J_{CF}$ = 23.4 Hz, C12), 32.07 (CH_2), 29.80 (CH_2), 29.78 (CH_2), 29.76 (CH_2), 29.64 (CH_2), 29.52 (CH_2), 29.50 (CH_2), 23.76 (d, $^{3}J_{CF}$ = 4.2 Hz, C11), 22.84 (CH_2), 14.26 (C1).

^{19}F NMR (564 MHz, $CDCl_3$) δ -144.95 – -145.16 (m, 1F, F-C13).

^{19}F{^{1}H} NMR (564 MHz, $CDCl_3$) δ -145.06 (s, 1F, F-C13).

FT-IR ($\tilde{\nu}$ = cm^{-1}): 2921 (s), 2852 (s), 1463 (m), 1377 (w), 1061 (m), 929 (w), 866 (w), 723 (m), 648 (m).

Analysis by mass spectrometry was inconclusive.

3-Fluoro-3-(fluoromethyl)pentadec-1-yne (27)

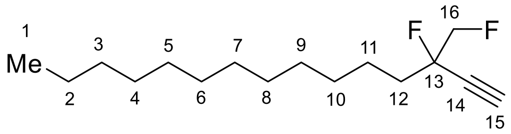

Compound **27** was prepared according to the General Procedure **B** with an amine:HF ratio of 1:5.5 using 3-(fluoromethyl)pentadeca-1,2-diene (**S27**) (24.0 mg, 0.1 mmol, 1.0 eq.). The crude product was purified by column chromatography (*n*-pentane) to yield the title compound as a colorless oil (17.1 mg, 0.066 mmol, 66%).

R*f* = 0.30 (*n*-pentane).

^{1}H NMR (500 MHz, $CDCl_3$) δ 4.60 – 4.33 (m, 2H, H-C16), 2.70 (d, $^{4}J_{HF}$ = 5.0 Hz, 1H, H-C15), 2.02 – 1.72 (m, 2H, H-C12), 1.58 – 1.52 (m, 2H, H-C11), 1.36 – 1.25 (m, 18H, H-C2~C10), 0.88 (t, $^{3}J_{HH}$ = 6.9 Hz, 3H, H-C1).

^{13}C NMR (126 MHz, $CDCl_3$) δ 90.76 (dd, $^{1}J_{CF}$ = 174.6 Hz, $^{2}J_{CF}$ = 21.1 Hz, C13), 85.06 (dd, $^{1}J_{CF}$ = 183.9 Hz, $^{2}J_{CF}$ = 27.2 Hz, C16), 78.71 (dd, $^{2}J_{CF}$ = 29.0 Hz, $^{3}J_{CF}$ = 7.9 Hz, C14), 77.54 (dd, $^{3}J_{CF}$

= 9.1 Hz, $^4J_{CF}$ = 1.4 Hz, C15), 35.24 (dd, $^2J_{CF}$ = 23.7 Hz, $^3J_{CF}$ = 2.9 Hz, C12), 32.07 (CH_2), 29.80 (CH_2), 29.79 (CH_2), 29.76 (CH_2), 29.65 (CH_2), 29.59 (CH_2), 29.53 (CH_2), 29.50 (CH_2), 23.68 (d, $^3J_{CF}$ = 4.4 Hz, C11), 22.84 (CH_2), 14.26 (C1).

^{19}F NMR (470 MHz, $CDCl_3$) δ -151.45 – -151.86 (m, 1F, F-C13), -226.34 (td, $^2J_{HF}$ = 47.4 Hz, $^3J_{FF}$ = 14.9 Hz, 1F, F-C16).

^{19}F{^{1}H} NMR (377 MHz, $CDCl_3$) δ -151.66 (d, $^3J_{FF}$ = 14.9 Hz, 1F, F-C13), -226.33 (d, $^3J_{FF}$ = 14.9 Hz, 1F, F-C16).

GC-EI-MS: (*m/z*) requires: [($C_{15}H_{26}F$)] = 225.2013 [M-CH_2F], (*m/z*) found: [($C_{15}H_{26}F$)] = 225.2013.

FT-IR ($\tilde{\nu}$ = cm^{-1}): 3306 (w), 2921 (s), 2852 (s), 1463 (m), 1377 (w), 901 (m), 671 (m), 648 (s), 608 (w).

3-(Chloromethyl)-3-fluoropentadec-1-yne (28)

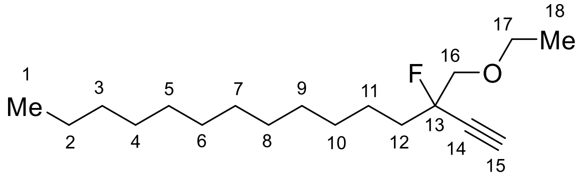

Compound **28** was prepared according to the General Procedure **B** with an amine:HF ratio of 1:4.5 using 3-(ethoxymethyl)pentadeca-1,2-diene (**S28**) (26.7 mg, 0.1 mmol, 1.0 eq.). The crude product was purified by column chromatography (*n*-pentane:Et_2O 40:1) to yield the title compound as a colorless oil (17.4 mg, 0.061 mmol, 61%).

R_f = 0.35 (*n*-pentane:Et_2O 40:1).

^{1}H NMR (500 MHz, $CDCl_3$) δ 3.76 – 3.55 (m, 4H, H-C16, H-C17), 2.65 (d, $^4J_{HF}$ = 5.1 Hz, 1H, H-C15), 2.02 – 1.69 (m, 2H, H-C12), 1.55 – 1.47 (m, 2H, H-C11), 1.30 – 1.24 (m, 18H, H-C2~C10), 1.22 (t, $^3J_{HH}$ = 7.0 Hz, 3H, H-C18), 0.88 (t, $^3J_{HH}$ = 7.0 Hz, 3H, H-C1).

^{13}C NMR (126 MHz, $CDCl_3$) δ 92.42 (d, $^1J_{CF}$ = 172.3 Hz, C13), 80.96 (d, $^2J_{CF}$ = 29.1 Hz, C14), 76.20 (d, $^3J_{CF}$ = 9.2 Hz, C15), 74.76 (d, $^2J_{CF}$ = 25.4 Hz, C16), 67.73 (C17), 36.31 (d, $^2J_{CF}$ = 23.8 Hz, C12), 32.07 (CH_2), 29.81 (CH_2), 29.79 (CH_2), 29.78 (CH_2), 29.68 (CH_2), 29.59 (CH_2), 29.50 (CH_2), 23.83 (d, $^3J_{CF}$ = 4.3 Hz, C11), 22.84 (CH_2), 15.22 (C18), 14.26 (C1).

^{19}F NMR (470 MHz, $CDCl_3$) δ -148.39 – -148.70 (m, 1F, F-C13).

^{19}F{^{1}H} NMR (470 MHz, $CDCl_3$) δ -148.53 (s, 1F, F-C13).

ESI-MS: (*m/z*) requires: [($C_{18}H_{33}OFNa$)$^+$] = 307.2408, (*m/z*) found: [($C_{18}H_{33}OFNa$)$^+$] = 307.2407.

FT-IR ($\tilde{\nu}$ = cm^{-1}): 2921 (s), 2852 (m), 1463 (m), 1377 (w), 1119 (s), 895 (w), 723 (w), 665 (m), 637 (m).

N-(2-ethynyl-2-fluorotetradecyl)-N,4-dimethylbenzenesulfonamide (29)

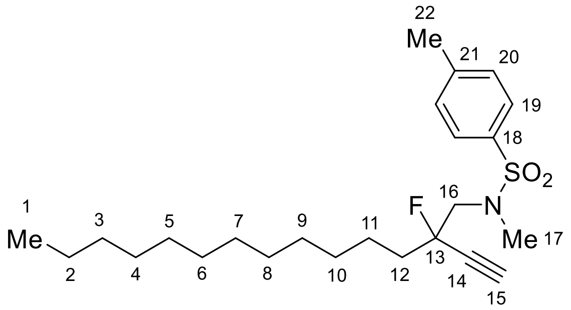

Compound **29** was prepared according to the General Procedure **B** with an amine:HF ratio of 1:5.0 using N,4-dimethyl-N-(2-vinylidenetetradecyl)benzenesulfonamide (**S29**) (40.6 mg, 0.1 mmol, 1.0 eq.). The crude product was purified by column chromatography (*n*-pentane:Et_2O 10:1) to yield the title compound as a colorless oil (23.2 mg, 0.055 mmol, 55%).

$\mathbf{R_f}$ = 0.30 (*n*-pentane:Et_2O 10:1).

^{1}H NMR (500 MHz, $CDCl_3$) δ 7.67 (d, $^3J_{HH}$ = 8.3 Hz, 2H, H-C19), 7.33 (d, $^3J_{HH}$ = 8.2 Hz, 2H, H-C20), 3.54 (dd, $^3J_{HF}$ = 23.8 Hz, $^2J_{HH}$ = 14.6 Hz, 1H, H-C16), 3.28 (dd, $^3J_{HF}$ = 16.2 Hz, $^2J_{HH}$ = 14.6 Hz, 1H, H-C16), 2.85 (s, 3H, H-C17), 2.71 (d, $^4J_{HF}$ = 5.2 Hz, 1H, H-C15), 2.43 (s, 3H, H-C22), 2.06 – 1.93 (m, 1H, H-C12), 1.91 – 1.76 (m, 1H, H-C12), 1.67 – 1.51 (m, 2H, H-C11), 1.35 – 1.24 (m, 18H, H-C2~C10), 0.88 (t, $^3J_{HH}$ = 6.9 Hz, 3H, H-C1).

^{13}C NMR (126 MHz, $CDCl_3$) δ 143.74 (C18), 134.83 (C21), 129.92 (C20), 127.57 (C19), 93.44 (d, $^1J_{CF}$ = 173.5 Hz, C13), 80.58 (d, $^2J_{CF}$ = 29.3 Hz, C14), 77.36 (d, $^3J_{CF}$ = 9.5 Hz, C15), 57.33 (d, $^2J_{CF}$ = 24.4 Hz, C16), 37.24 (d, $^4J_{CF}$ = 4.7 Hz, C17), 36.84 (d, $^2J_{CF}$ = 23.9 Hz, C12), 32.07 (*C*H_2), 29.82 (*C*H_2), 29.79 (*C*H_2), 29.78 (*C*H_2), 29.71 (*C*H_2), 29.64 (*C*H_2), 29.63 (*C*H_2), 29.50 (*C*H_2), 23.81 (d, $^3J_{CF}$ = 4.0 Hz, C11), 22.84 (*C*H_2), 21.65 (C22), 14.26 (C1).

^{19}F NMR (376 MHz, $CDCl_3$) δ -143.57 (tddd, $^3J_{HF}$ = 22.9, 16.2, 11.2 Hz, $^4J_{HF}$ = 5.2 Hz, 1F, F-C13).

^{19}F{^{1}H} NMR (376 MHz, $CDCl_3$) δ -143.56 (s, 1F, F-C13).

ESI-MS: (*m/z*) requires: [$(C_{24}H_{38}O_2FNSNa)^+$] = 446.2499, (*m/z*) found: [$(C_{24}H_{38}O_2FNSNa)^+$] = 446.2498.

FT-IR ($\tilde{\nu}$ = cm^{-1}): 2921 (m), 2852 (m), 1457 (w), 1343 (m), 1159 (s), 1090 (m), 964 (m), 815 (w), 746 (s), 654 (s).

4-Ethynyl-4-fluorohexadecane (30)

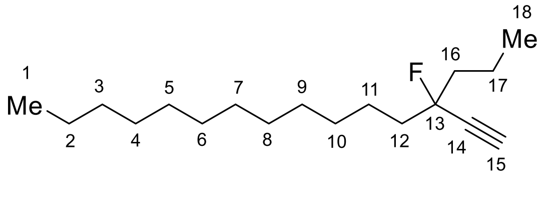

Compound **30** was prepared according to the General Procedure **B** with an amine:HF ratio of 1:5.5 using 4-vinylidenehexadecane (**S30**) (25.1 mg, 0.1 mmol, 1.0 eq.). The crude product was purified by column

chromatography (*n*-pentane) to yield the title compound as a colorless oil (10.8 mg, 0.04 mmol, 40%).

R*f* = 0.50 (*n*-pentane).

^{1}H NMR (400 MHz, $CDCl_3$) δ 2.61 (d, $^4J_{HF}$ = 5.0 Hz, 1H, H-C15), 1.87 – 1.65 (m, 4H, H-C12, H-C16), 1.62 – 1.48 (m, 4H, H-C11, H-C17), 1.32 – 1.24 (m, 18H, H-C2~C10), 0.96 (t, $^3J_{HH}$ = 7.3 Hz, 3H, H-C18), 0.88 (t, $^3J_{HH}$ = 6.8 Hz, 3H, H-C1).

^{13}C NMR (151 MHz, $CDCl_3$) δ 93.04 (d, $^1J_{CF}$ = 167.9 Hz, C13), 82.84 (d, $^2J_{CF}$ = 30.0 Hz, C14), 75.17 (d, $^3J_{CF}$ = 9.1 Hz, C15), 42.34 (d, $^2J_{CF}$ = 24.2 Hz, C16), 40.25 (d, $^2J_{CF}$ = 24.1 Hz, C12), 32.08 (CH_2), 29.82 (CH_2), 29.79 (CH_2), 29.72 (CH_2), 29.71 (CH_2), 29.63 (CH_2), 29.51 (CH_2), 24.00 (d, $^3J_{CF}$ = 4.0 Hz, C11), 22.84 (CH_2), 17.41 (d, $^3J_{CF}$ = 4.3 Hz, C17), 14.27 (C1), 14.23 (C18).

^{19}F NMR (376 MHz, $CDCl_3$) δ -142.37 (ttd, $^3J_{HF}$ = 24.0, 14.6 Hz, $^4J_{HF}$ = 5.1 Hz, 1F, F-C13).

^{19}F{^{1}H} NMR (376 MHz, $CDCl_3$) δ -142.37 (s, 1F, F-C13).

GC-EI-MS: (*m/z*) requires: [($C_{18}H_{33}F$)] = 268.2566, (*m/z*) found: [($C_{18}H_{33}F$)] = 268.2561.

FT-IR ($\tilde{\nu}$ = cm^{-1}): 2921 (s), 2852 (s), 1463 (m), 1377 (w), 1061 (m), 970 (m), 929 (w), 855 (w), 723 (w), 665 (m), 631 (m).

(1S,2R,5S)-2-Isopropyl-5-methylcyclohexyl 4-((S)-1-chloro-2-fluorobut-3-yn-2-yl)benzoate (31)

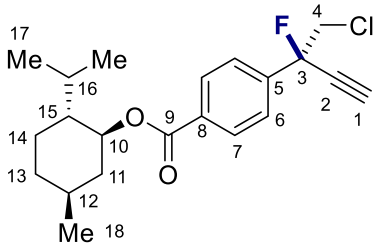

Compound **31** was prepared according to the General Procedure **B** with an amine:HF ratio of 1:5.0 using (1S,2R,5S)-2-isopropyl-5-methylcyclohexyl 4-(1-chlorobuta-2,3-dien-2-yl)benzoate (**S31**) (34.7 mg, 0.1 mmol, 1.0 eq.). The crude product was purified by column chromatography (*n*-pentane:Et_2O 30:1) to yield the title compound as a colorless oil (26.8 mg, 0.073 mmol, 73%).

Two sets of peaks were observed in ^{19}F NMR spectra due to diastereoisomers.

R*f* = 0.30 (*n*-pentane:Et_2O 30:1).

^{1}H NMR (599 MHz, $CDCl_3$) δ 8.20 – 8.04 (m, 2H, H-C7), 7.71 – 7.52 (m, 2H, H-C6), 4.94 (td, $^3J_{HH}$ = 10.9, 4.4 Hz, 1H, H-C10), 3.90 (dd, $^3J_{HF}$ = 16.5 Hz, $^5J_{HH}$ = 1.0 Hz, 2H, H-C4), 2.97 (d, $^4J_{HF}$ = 5.3 Hz, 1H, H-C1), 2.18 – 2.10 (m, 1H, H-C11), 1.98 – 1.90 (m, 1H, H-C12), 1.80 – 1.68 (m, 2H, H-C13, H-C14), 1.60 – 1.53 (m, 2H, H-C15, H-C16), 1.20 – 1.05 (m, 2H, H-C11, H-C14), 0.99 – 0.87 (m, 7H, H-C13, H-C17), 0.79 (d, $^3J_{HH}$ = 7.0 Hz, 3H, H-C18).

^{13}C NMR (151 MHz, $CDCl_3$) δ 165.48 (C9), 141.76 (d, $^2J_{CF}$ = 23.3 Hz, C5), 132.05 (C8), 129.91 (C7), 125.62 (d, $^3J_{CF}$ = 6.2 Hz, C6), 90.74 (d, $^1J_{CF}$ = 180.5 Hz, C3), 79.18 (d, $^2J_{CF}$ = 28.9 Hz, C2), 78.97 (d, $^3J_{CF}$ = 8.9 Hz, C1), 75.37 (C10), 50.86 (d, $^2J_{CF}$ = 23.3 Hz, C4), 47.41 (C15), 41.08 (C11), 34.44 (C13), 31.60 (C16), 26.68 (C12), 23.77 (C14), 22.18 (C17), 20.90 (C17'), 16.66 (C18).

^{19}F NMR (564 MHz, $CDCl_3$) δ -144.32 – -144.52 (m, 1F, F-C3).

^{19}F{^{1}H} NMR (564 MHz, $CDCl_3$) δ -144.41 (s, 1F, F-C3), -144.42 (s, 1F, F-C3').

ESI-MS: (*m/z*) requires: [($C_{21}H_{26}ClFNaO_2$)$^+$] = 387.1498, (*m/z*) found: [($C_{21}H_{26}ClFNaO_2$)$^+$] = 387.1498.

FT-IR ($\tilde{\nu}$ = cm^{-1}): 2955 (m), 2359 (s), 1716 (s), 1406 (w), 1274 (s), 1182 (w), 1113 (m), 866 (w), 700 (w), 671 (w).

1.5 Synthetic applications

1.5.1 Product derivatisation

Methyl 4-(1-chloro-2-fluoro-4-phenylbut-3-yn-2-yl)benzoate (32)

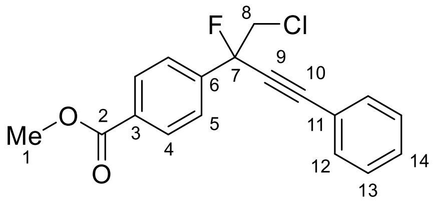

Methyl 4-(1-chloro-2-fluorobut-3-yn-2-yl)benzoate **(23)** (24.1 mg, 0.10 mmol, 1.0 eq.), $Pd(PPh_3)_4$ (11.6 mg, 0.01 mmol, 10 mol%), and CuI (1.9 mg, 0.01 mmol, 10 mol%) were placed in a Schlenk flask. The flask was evacuated and refilled with Ar. NEt_3 (0.3 mL), and iodobenzene (40.8 mg, 0.20 mmol, 2.0 eq.) were successively added into the flask. The reaction was stirred at 60 °C for 12 h. The solvent was removed on a rotary evaporator. Further purification by column chromatography (*n*-pentane:Et_2O 8:1) afforded the title compound as a white wax (22.8 mg, 0.072 mmol, 72%).

R_f = 0.30 (*n*-pentane:Et_2O 8:1).

^{1}H NMR (599 MHz, $CDCl_3$) δ 8.14 – 8.09 (m, 2H, H-C4), 7.74 – 7.68 (m, 2H, H-C5), 7.56 – 7.51 (m, 2H, H-C12), 7.43 – 7.34 (m, 3H, H-C13, H-C14), 4.02 – 3.97 (m, 2H, H-C8), 3.94 (s, 3H, H-C1).

^{13}C NMR (151 MHz, $CDCl_3$) δ 166.57 (C2), 142.74 (d, $^2J_{CF}$ = 24.0 Hz, C6), 132.25 (d, $^5J_{CF}$ = 2.6 Hz, C12), 131.17 (d, $^5J_{CF}$ = 1.4 Hz, C3), 129.94 (C4), 129.77 (C14), 128.60 (C13), 125.83 (d, $^3J_{CF}$ = 6.4 Hz, C5), 121.06 (d, $^3J_{CF}$ = 3.5 Hz, C11), 91.39 (d, $^1J_{CF}$ = 180.0 Hz, C7), 90.64 (d, $^3J_{CF}$ = 8.9 Hz, C10), 84.20 (d, $^2J_{CF}$ = 28.3 Hz, C9), 52.44 (C1), 51.22 (d, $^2J_{CF}$ = 31.9 Hz, C8).

^{19}F NMR (564 MHz, $CDCl_3$) δ -141.62 (t, $^3J_{HF}$ = 16.4 Hz, 1F, F-C7).

^{19}F{^{1}H} NMR (564 MHz, $CDCl_3$) δ -141.62 (s, 1F, F-C7).

ESI-MS: (*m/z*) requires: [($C_{18}H_{14}O_2FClNa$)$^+$] = 339.0559, (*m/z*) found: [($C_{18}H_{14}O_2FClNa$)$^+$] = 339.0559.

FT-IR ($\tilde{\nu}$ = cm^{-1}): 2955 (w), 1721 (s), 1434 (w), 1406 (w), 1279 (s), 1113 (m), 751 (m), 694 (m).

Methyl 4-(1-chloro-2-fluoro-4-phenylbut-3-yn-2-yl)benzoate (33)

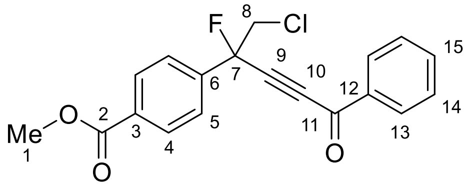

Methyl 4-(1-chloro-2-fluorobut-3-yn-2-yl)benzoate **(23)** (24.1 mg, 0.10 mmol, 1.0 eq.), Pd(PPh_3)$_4$ (11.6 mg, 0.01 mmol, 10 mol%), and CuI (2.9 mg, 0.015 mmol, 15 mol%) were placed in a Schlenk flask. The flask was evacuated and refilled with Ar. NEt_3 (0.4 mL), and benzoyl chloride (42.2 mg, 0.30 mmol, 3.0 eq.) were successively added into the flask. The reaction was stirred at 30 °C for 12 h. The solvent was removed on a rotary evaporator. Further purification by column chromatography (*n*-pentane:Et_2O 4:1) afforded the title compound as a pale yellow oil (22.8 mg, 0.072 mmol, 72%).

R*f* = 0.39 (*n*-pentane:Et_2O 3:1).

^{1}H NMR (500 MHz, $CDCl_3$) δ 8.20 – 8.09 (m, 4H, H-C4, H-C13), 7.72 – 7.68 (m, 2H, H-C5), 7.68 – 7.64 (m, 2H, H-C15), 7.56 – 7.50 (m, 2H, H-C14), 4.05 – 3.99 (m, 2H, H-C8), 3.95 (s, 3H, H-C1).

^{13}C NMR (126 MHz, $CDCl_3$) δ 176.76 (d, $^4J_{CF}$ = 2.3 Hz, C11), 166.33 (C2), 140.92 (d, $^2J_{CF}$ = 23.5 Hz, C6), 136.15 (C12), 135.03 (C15), 131.79 (C3), 130.23 (C4), 129.89 (C13), 129.03 (C14), 125.64 (d, $^3J_{CF}$ = 6.4 Hz, C5), 91.10 (d, $^1J_{CF}$ = 183.5 Hz, C7), 86.57 (d, $^2J_{CF}$ = 28.9 Hz, C9), 86.39 (d, $^3J_{CF}$ = 8.5 Hz, C10), 52.54 (C1), 50.51 (d, $^2J_{CF}$ = 31.0 Hz, C8).

^{19}F NMR (470 MHz, $CDCl_3$) δ -145.02 (dd, $^3J_{HF}$ = 18.1, 15.2 Hz, 1F, F-C7).

^{19}F{^{1}H} NMR (470 MHz, $CDCl_3$) δ -145.02 (s, 1F, F-C7).

ESI-MS: (*m/z*) requires: [($C_{19}H_{14}O_3FClNa$)$^+$] = 367.0508, (*m/z*) found: [($C_{19}H_{14}O_3FClNa$)$^+$] = 367.0508.

FT-IR ($\tilde{\nu}$ = cm^{-1}): 2973 (m), 2887 (m), 1377 (m), 1274 (w), 1084 (s), 1044 (s).

3-Fluoropentadecane (34)

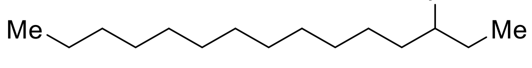

3-Fluoropentadec-1-yne (**1**) (22.6 mg, 0.10 mmol, 1.0 eq.) and palladium on carbon (10% w/w. 10.6 mg, 0.01 mmol, 10 mol%) were placed in a Schlenk flask. The flask was evacuated and refilled with hydrogen through a balloon. After addition of 1.0 mL methanol, the mixture was stirred at room

temperature for 24 h under balloon pressure of hydrogen. The reaction mixture was filtered through celite and washed with Et_2O. After removal of solvent, the residue was purified by column chromatography (*n*-pentane) to yield the title compound as a colorless oil (22.3 mg, 0.097 mmol, 97%).

R*f* = 0.65 (*n*-pentane).

^{1}H NMR (500 MHz, $CDCl_3$) δ 4.52 – 4.25 (m, 1H), 1.69 – 1.42 (m, 4H), 1.35 – 1.22 (m, 20H), 0.96 (t, *J* = 7.4 Hz, 3H), 0.90 – 0.87 (m, 3H).

^{19}F NMR (470 MHz, $CDCl_3$) δ -180.98 – -181.42 (m, 1F).

GC-EI-MS: (*m/z*) requires: [($C_{15}H_{30}$)] = 210.2342 [M-HF], (*m/z*) found: [($C_{15}H_{30}$)] = 210.2341.

Analytical data is in agreement with literature values.[15]

3-Fluoropentadec-1-ene (35)

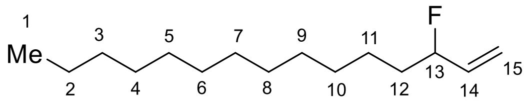

3-Fluoropentadec-1-yne (**1**) (22.6 mg, 0.10 mmol, 1.0 eq.) and lindlar catalyst (4.5 mg, 20 wt%) were placed in a Schlenk flask. The flask was evacuated and refilled with hydrogen through a balloon. After addition of 0.6 mL pyridine and quinoline (12.9 mg, 0.10 mmol, 1.0 eq.), the mixture was stirred at room temperature for 30 min under balloon pressure of hydrogen. The reaction mixture was filtered through celite and washed with Et_2O. After removal of solvent, the residue was purified by column chromatography (*n*-pentane) to yield the title compound as a colorless oil (22.4 mg, 0.098 mmol, 98%).

R*f* = 0.65 (*n*-pentane).

^{1}H NMR (500 MHz, $CDCl_3$) δ 5.88 (dddd, $^3J_{HF}$ = 14.0 Hz, $^3J_{HH}$ = 17.3, 10.6, 6.1 Hz, 1H, H-C14), 5.30 (ddt, $^3J_{HH}$ = 17.2, $^4J_{HF}$ = 3.6 Hz, $^2J_{HH}$ = 1.4 Hz, $^4J_{HH}$ = 1.4 Hz, 1H, H-C15), 5.21 (dt, $^3J_{HH}$ = 10.6, $^2J_{HH}$ =1.3 Hz, $^4J_{HH}$ =1.3 Hz, 1H, H-C15), 4.96 – 4.76 (m, 1H, H-C13), 1.78 – 1.54 (m, 2H, H-C12), 1.49 – 1.25 (m, 20H, H-C2~C11), 0.88 (t, $^3J_{HH}$ = 6.9 Hz, 3H, H-C1).

^{13}C NMR (126 MHz, $CDCl_3$) δ 136.99 (d, $^2J_{CF}$ = 19.7 Hz, C14), 116.85 (d, $^3J_{CF}$ = 12.0 Hz, C15), 93.91 (d, $^1J_{CF}$ = 166.8 Hz, C13), 35.39 (d, $^2J_{CF}$ = 22.0 Hz, C12), 32.08 (CH_2), 29.82 (CH_2), 29.80 (CH_2), 29.71 (CH_2), 29.66 (CH_2), 29.55 (CH_2), 29.51 (CH_2), 24.82 (d, $^3J_{CF}$ = 4.7 Hz, C11), 22.85 (CH_2), 14.27 (C1).

^{19}F NMR (470 MHz, $CDCl_3$) δ -176.50 – -177.02 (m, 1F, F-C13).

^{19}F{^{1}H} NMR (470 MHz, $CDCl_3$) δ -176.76 (s, 1F, F-C13).

GC-EI-MS: (*m/z*) requires: [($C_{15}H_{28}$)] = 208.2186 [M-HF], (*m/z*) found: [($C_{15}H_{28}$)] = 208.2186.

FT-IR ($\tilde{\nu}$ = cm^{-1}): 2921 (s), 2852 (s), 1463 (w), 981 (m), 929 (m), 723 (w).

3-Fluoropentadecan-2-one (36)

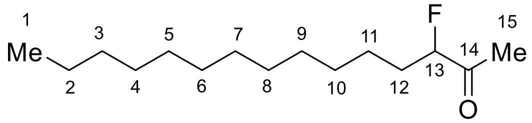

3-Fluoropentadec-1-yne (**1**) (22.6 mg, 0.10 mmol, 1.0 eq.) and $AgSbF_6$ (10.3 mg, 0.03 mmol, 30 mol%) were placed in a Schlenk flask. The flask was evacuated and refilled with Ar. After addition of 0.5 mL methanol and 0.05 mL water, the reaction was stirred at 80 °C for 24 h. The solvent was removed on a rotary evaporator. Further purification by column chromatography (*n*-pentane:Et_2O 60:1) afforded the title compound as a pale yellow oil (13.8 mg, 0.056 mmol, 56%)..

R*f* = 0.30 (*n*-pentane:Et_2O 60:1).

^{1}H NMR (500 MHz, $CDCl_3$) δ 4.82 – 4.59 (m, 1H, H-C13), 2.25 (d, $^4J_{HF}$ = 4.8 Hz, 3H, H-C15), 1.88 – 1.68 (m, 2H, H-C12), 1.47 – 1.40 (m, 2H, H-C11), 1.36 – 1.22 (m, 18H, H-C2~C10), 0.88 (t, $^3J_{HH}$ = 6.9 Hz, 3H, H-C1).

^{13}C NMR (126 MHz, $CDCl_3$) δ 208.63 (d, $^2J_{CF}$ = 25.9 Hz, C14), 96.16 (d, $^1J_{CF}$ = 183.9 Hz, C13), 32.06, 32.00 (d, $^2J_{CF}$ = 20.8 Hz, C12), 29.79 (CH_2), 29.78 (CH_2), 29.74 (CH_2), 29.64 (CH_2), 29.49 (CH_2), 29.48 (CH_2), 29.31 (CH_2), 26.00 (C15), 24.60 (d, $^3J_{CF}$ = 3.0 Hz, C11), 22.84 (CH_2), 14.26 (C1).

^{19}F NMR (470 MHz, $CDCl_3$) δ -189.12 – -189.89 (m, 1F, F-C13).

^{19}F{^{1}H} NMR (470 MHz, $CDCl_3$) δ -189.46 (s, 1F, F-C13).

GC-EI-MS: (*m/z*) requires: [($C_{15}H_{29}FO$)] = 244.2202, (*m/z*) found: [($C_{15}H_{29}FO$)] = 244.2197.

FT-IR ($\tilde{\nu}$ = cm^{-1}): 2921 (s), 2852 (s), 1727 (s), 1463 (m), 1360 (m), 1234 (w), 1079 (m), 981 (w), 723 (w).

N-(4-((4-(1-fluorotridecyl)-1H-1,2,3-triazol-1-yl)sulfonyl)phenyl)acetamide (37)

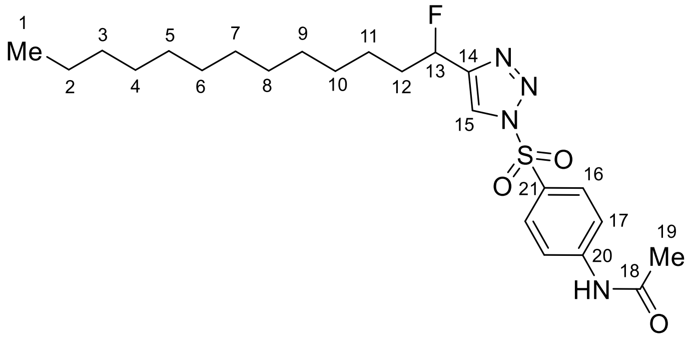

Copper thiophene-2-carboxylate (1.9 mg, 0.01 mmol, 10 mol%) was added to a stirred solution of 3-fluoropentadec-1-yne (**1**) (22.6 mg, 0.10 mmol, 1.0 eq.) in toluene (0.5 mL). The reaction mixture was cooled in an ice-water bath. Subsequently, 4-

acetamidobenzenesulfonyl azide (28.8 mg, 0.12 mmol, 1.2 eq.) was added slowly. The reaction mixture was allowed to warm to room temperature and stir for 12 h. After removal of the solvent, the residue was directly purified by column chromatography (*n*-pentane:EA 1.5:1-1:1) to yield the title compound as a white solid (41.8 mg, 0.090 mmol, 90%).

R*f* = 0.25 (*n*-pentane:EA 1.5:1).

^{1}H NMR (500 MHz, $CDCl_3$) δ 8.26 (s, 1H, NH), 8.18 – 8.14 (m, 1H, H-C15), 8.01 – 7.95 (m, 2H, H-C17), 7.77 – 7.72 (m, 2H, H-C16), 5.70 – 5.54 (m, 1H, H-C13), 2.22 (s, 3H, H-C19), 2.12 – 1.90 (m, 2H, H-C12), 1.54 – 1.37 (m, 2H, H-C11), 1.35 – 1.22 (m, 18H, H-C2~C10), 0.87 (t, $^3J_{HH}$ = 6.9 Hz, 3H, H-C1).

^{13}C NMR (126 MHz, $CDCl_3$) δ 169.36 (C18), 147.38 (d, $^2J_{CF}$ = 25.1 Hz, C14), 145.39 (C20), 130.46 (C17), 129.20 (C21), 121.64 (d, $^3J_{CF}$ = 4.3 Hz, C15), 119.74 (C16), 87.27 (d, $^1J_{CF}$ = 168.6 Hz, C13), 35.02 (d, $^2J_{CF}$ = 22.1 Hz, C12), 32.03 (CH_2), 29.77 (CH_2), 29.75 (CH_2), 29.72 (CH_2), 29.63 (CH_2), 29.51 (CH_2), 29.46 (CH_2), 29.28 (CH_2), 24.86 (C19), 24.84 (d, $^3J_{CF}$ = 3.9 Hz, C11), 22.80 (CH_2), 14.24 (C1).

^{19}F NMR (470 MHz, $CDCl_3$) δ -176.30 (ddd, $^2J_{HF}$ = 47.8 Hz, $^3J_{HF}$ = 29.7, 18.0 Hz, 1F, F-C13).

^{19}F{^{1}H} NMR (470 MHz, $CDCl_3$) δ -176.30 (s, 1F, F-C13).

ESI-MS: (*m/z*) requires: [$(C_{23}H_{35}O_3FN_4SNa)^+$] = 489.2306, (*m/z*) found: [$(C_{23}H_{35}O_3FN_4SNa)^+$] = 489.2306.

FT-IR ($\tilde{\nu}$ = cm^{-1}): 2921 (m), 2852 (w), 1698 (m), 1589 (m), 1526 (m), 1394 (s), 1320 (w), 1193 (m), 1176 (s), 1090 (w), 1021 (m), 998 (m), 832 (w), 728 (m), 648 (m), 619 (s).

Melting Point: 80.5-81.5 ˚C.

1.5.2 Sequential reaction

4-(Chloromethyl)-4-fluorohexadeca-1,2-diene (38)

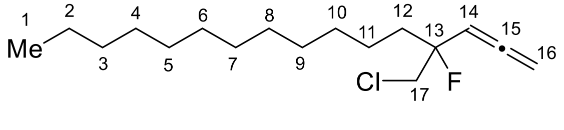

Compound **38** was prepared according to General Procedure **A** using 3-(chloromethyl)-3-fluoropentadec-1-yne (**26**) (328.1 mg, 1.19 mmol, 1.0 eq.). The crude residue was purified by column chromatography (*n*-pentane) to yield the title compound as a colorless oil (191.9 mg, 0.66 mmol, 56%).

R*f* = 0.65 (*n*-pentane).

^{1}H NMR (500 MHz, $CDCl_3$) δ 5.35 (dt, $^{3}J_{HF}$ = 11.5 Hz, $^{4}J_{HH}$ = 6.8 Hz, 1H, H-C14), 4.98 (t, $^{4}J_{HH}$ = 6.8 Hz, 2H, H-C16), 3.75 – 3.57 (m, 2H, H-C17), 1.96 – 1.72 (m, 2H, H-C12), 1.44 – 1.36 (m, 2H, H-C11), 1.32 – 1.24 (m, 18H, H-C2~C10), 0.88 (t, $^{3}J_{HH}$ = 6.9 Hz, 3H, H-C1).
^{13}C NMR (126 MHz, $CDCl_3$) δ 207.64 (d, $^{3}J_{CF}$ = 10.2 Hz, C15), 94.73 (d, $^{1}J_{CF}$ = 177.9 Hz, C13), 92.45 (d, $^{2}J_{CF}$ = 27.9 Hz, C14), 78.99 (d, $^{4}J_{CF}$ = 1.9 Hz, C16), 48.73 (d, $^{2}J_{CF}$ = 29.9 Hz, C17), 36.07 (d, $^{2}J_{CF}$ = 22.7 Hz, C12), 32.07 (CH_2), 29.81 (CH_2), 29.79 (CH_2), 29.77 (CH_2), 29.67 (CH_2), 29.58 (CH_2), 29.50 (CH_2), 23.28 (d, $^{3}J_{CF}$ = 5.2 Hz, C11), 22.84 (CH_2), 14.26 (C1).
^{19}F NMR (470 MHz, $CDCl_3$) δ -146.80 – -147.27 (m, 1F, F-C13).
^{19}F{^{1}H} NMR (470 MHz, $CDCl_3$) δ -147.06 (s, 1F, F-C13).
GC-EI-MS: (*m/z*) requires: [($C_{17}H_{30}FCl$)] = 288.2020, (*m/z*) found: [($C_{17}H_{30}FCl$)] = 288.2015.
FT-IR ($\tilde{\nu}$ = cm^{-1}): 2921 (s), 2852 (s), 1957 (w), 1463 (m), 1010 (w), 849 (s), 797 (m), 751 (m), 642 (w).

(*Z*)-4-(chloromethyl)-1,2,4-trifluorohexadec-2-ene (39)

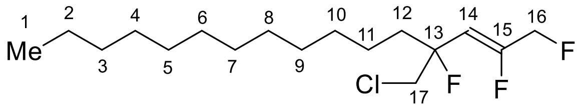

Compound **39** was prepared according to the General Procedure **B** with an amine:HF ratio of 1:5.5 using 3-(chloromethyl)pentadeca-1,2-diene (**38**) (28.9 mg, 0.1 mmol, 1.0 eq.). The crude product was purified by column chromatography (*n*-pentane) to yield the title compound as a colorless oil (11.3 mg, 0.035 mmol, 35%).

R*f* = 0.35 (*n*-pentane).

^{1}H NMR (400 MHz, $CDCl_3$) δ 5.21 (ddd, $^{3}J_{HF}$ = 38.0, 17.1 Hz, $^{4}J_{HF}$ = 3.0 Hz, 1H, H-C14), 5.35 (dd, $^{2}J_{HF}$ = 47.1 Hz, $^{3}J_{HF}$ = 14.9 Hz, 2H, H-C16), 3.88 – 3.65 (m, 2H, H-C17), 2.00 – 1.82 (m, 2H, H-C12), 1.49 – 1.37 (m, 2H, H-C11), 1.35 – 1.20 (m, 18H, H-C2~C10), 0.88 (t, $^{3}J_{HH}$ = 6.8 Hz, 3H, H-C1).

^{13}C NMR (126 MHz, $CDCl_3$) δ 154.32 (app. C15), 111.14 (dt, $^{2}J_{CF}$ = 30.8, 7.8 Hz, $^{3}J_{CF}$ = 7.8 Hz, C14), 95.30 (app. C13), 79.49 (dd, $^{1}J_{CF}$ = 171.5 Hz, $^{2}J_{CF}$ = 34.4 Hz, C16), 48.50 (ddd, $^{2}J_{CF}$ = 27.3 Hz, $^{4}J_{CF}$ = 4.2 Hz, $^{5}J_{CF}$ = 2.4 Hz, C17), 36.55 (dt, $^{2}J_{CF}$ = 22.4 Hz, $^{4}J_{CF}$ = 2.0 Hz, $^{5}J_{CF}$ = 2.0 Hz, C12), 32.07 (CH_2), 29.80 (CH_2), 29.78 (CH_2), 29.77 (CH_2), 29.75 (CH_2), 29.66 (CH_2), 29.53 (CH_2), 29.50 (CH_2), 23.30 (d, $^{3}J_{CF}$ = 3.7 Hz, C11), 22.84 (CH_2), 14.26 (C1).
^{19}F NMR (470 MHz, $CDCl_3$) δ -112.21 – -112.65 (m, 1F, F-C15), -150.37 – -150.80 (m, 1F, F-C13), -219.91 – -220.49 (m, 1F, F-C16).

^{19}F{^{1}H} NMR (377 MHz, $CDCl_3$) δ -112.43 (dd, $^{3}J_{FF}$ = 32.0 Hz, $^{4}J_{FF}$ = 12.7 Hz, 1F, F-C15), -150.59 (dd, $^{4}J_{FF}$ = 12.6 Hz, $^{5}J_{FF}$ = 2.8 Hz, 1F, F-C13), -220.19 (dd, $^{3}J_{FF}$ = 32.0 Hz, $^{5}J_{FF}$ = 2.7 Hz, 1F, F-C16).

FT-IR ($\tilde{\nu}$ = cm^{-1}): 2927 (s), 2852 (m), 1710 (w), 1463 (w), 1377 (w), 1216 (w), 1061 (m), 998 (m), 878 (m), 815 (m), 757 (s), 734 (s), 700 w).

Analysis by mass spectrometry was inconclusive.

1.5.3 Preliminary validation of enantioselective catalysis

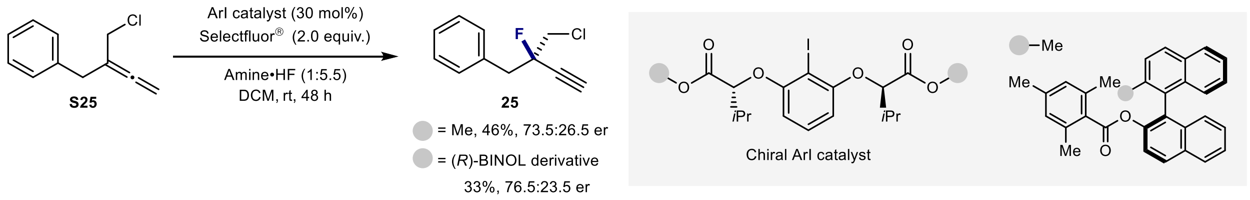

Compound **25** was prepared according to the General Procedure **B** with an amine:HF ratio of 1:5.5 using (2-(chloromethyl)buta-2,3-dien-1-yl)benzene (**S25**) (17.9 mg, 0.1 mmol, 1.0 eq.) over 48 h. The catalyst was changed to chiral dimethyl 2,2'-((2-iodo-1,3-phenylene)bis(oxy))(2*R*,2'*R*)-bis(3-methylbutanoate). The crude product was purified by column chromatography (*n*-pentane:DCM 10:1) to yield the title compound as a colorless oil (9.0 mg, 0.046 mmol, 46%) with an e.r. of 26.5:73.5. The enantiomeric ratio (e.r.) was determined by chiral HPLC (chiral OD-H-column, *n*-hexane: *i*-PrOH 99.9:0.1; flow rate: 0.5 mL/min; t-minor = 26.246 min, t-major = 27.779 min).

Compound **25** was prepared according to the General Procedure **B** with an amine:HF ratio of 1:5.5 using (2-(chloromethyl)buta-2,3-dien-1-yl)benzene (**S25**) (17.9 mg, 0.1 mmol, 1.0 eq.) over 48 h. The catalyst was changed to chiral (1'R)-2'-(((2R)-2-(2-iodo-3-(((2R)-3-methyl-1-oxo-1-((2'-((2,4,6-trimethylbenzoyl)oxy)-[1,1'-binaphthalen]-2-yl)oxy)butan-2-yl)oxy)phenoxy)-3-methylbutanoyl)oxy)-[1,1'-binaphthalen]-2-yl 2,4,6-trimethylbenzoate. The crude product was purified by column chromatography (*n*-pentane:DCM 10:1) to yield the title compound as a colorless oil (6.4 mg, 0.033 mmol, 33%) with an e.r. of 23.5:76.5. The enantiomeric ratio (e.r.) was determined by chiral HPLC (chiral OD-H-column, *n*-hexane: *i*-PrOH 99.9:0.1; flow rate: 0.5 mL/min; t-minor = 26.818 min, t-major = 28.174 min).

Racemic sample:

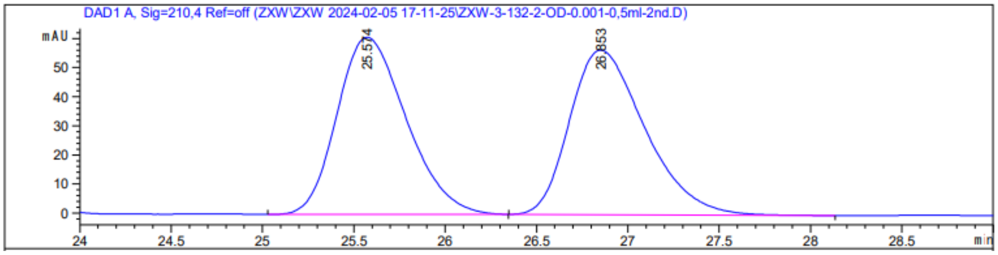

Signal 1: DAD1 A, Sig=210,4 Ref=off

Peak #	RetTime [min]	Type	Width [min]	Area [mAU*s]	Height [mAU]	Area %
1	25.574	BB	0.4094	1603.50293	60.86435	49.7620
2	26.853	BB	0.4421	1618.84180	56.53323	50.2380
Totals :				3222.34473	117.39758	

Chiral sample:

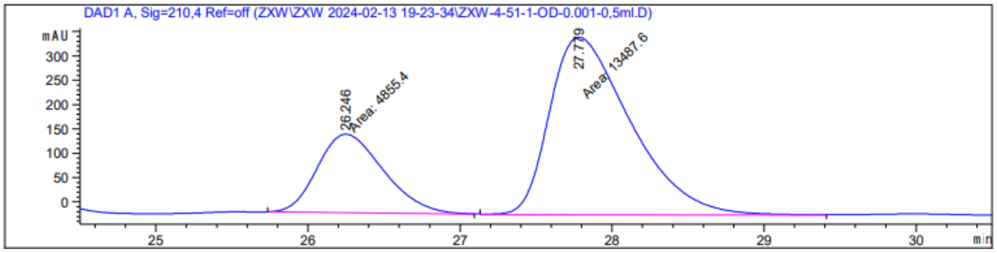

Signal 1: DAD1 A, Sig=210,4 Ref=off

Peak #	RetTime [min]	Type	Width [min]	Area [mAU*s]	Height [mAU]	Area %
1	26.246	MM	0.5027	4855.40186	160.98737	26.4700
2	27.779	MF	0.6167	1.34876e4	364.50543	73.5300
Totals :				1.83430e4	525.49280	

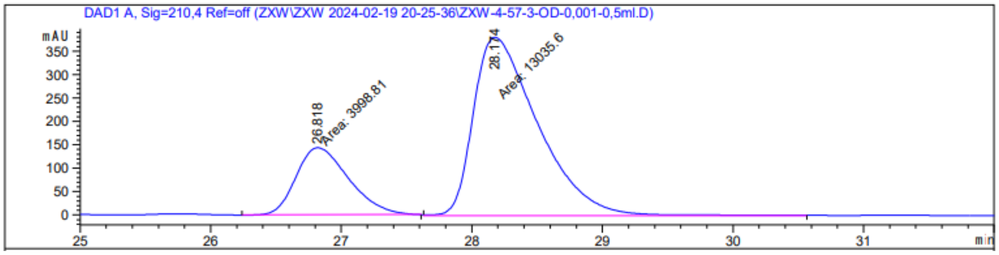

Signal 1: DAD1 A, Sig=210,4 Ref=off

Peak #	RetTime [min]	Type	Width [min]	Area [mAU*s]	Height [mAU]	Area %
1	26.818	MM	0.4654	3998.81128	143.20923	23.4749
2	28.174	FM	0.5710	1.30356e4	380.46378	76.5251
Totals :				1.70344e4	523.67300	

1.6 General catalytic functionalisation of allenes

General Procedure C for regioselective functionalisation of allenes

To a solution of allene (0.1 mmol, 1.0 eq.), *p*-iodotoluene (4.4 mg, 0.02 mmol, 20 mol%) and the nucleophile (3.0 to 45.0 eq.) in HFIP (0.5 mL), Selectfluor® (53.1 mg, 0.15 mmol, 1.5 eq.) was added in one portion under argon. After stirring (350 rpm) at 40 °C for 16 h, the reaction mixture was poured into 100 mL of a saturated solution of $NaHCO_3$. The organics were extracted with DCM (3x 30 mL), the combined organic layers were dried over Na_2SO_4, filtered and the solvent was carefully removed under reduced pressure. An internal standard (ethyl fluoroacetate) was added to the crude residue. The NMR yield and regioselectivity ratio were analysed by ^{19}F NMR spectroscopy against the internal standard. The NMR sample was recombined with the crude residue and purification by column chromatography or preparative thin layer chromatography yielded the desired product.

1-(4-(Trifluoromethyl)phenyl)prop-2-yn-1-yl acetate (40)

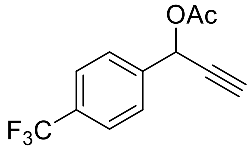

Compound **40** was prepared according to the General Procedure **C** using 1-(propa-1,2-dien-1-yl)-4-(trifluoromethyl)benzene (**S16**) (18.4 mg, 0.1 mmol, 1.0 eq.), *p*-iodotoluene (6.5 mg, 0.03 mmol, 30 mol%) and AcOH (150.1 mg, 2.5 mmol, 25.0 eq.). After workup, the crude mixture was analysed by ^{19}F NMR (63%, b:l = 8:1). Purification by column chromatography (*n*-pentane:Et_2O 15:1) yielded the title compound **40** and the linear propargylic acetate as an inseparable mixture (colorless oil, 13.1 mg, 0.054 mmol, 54%, b:l = 17:1).

$\mathbf{R_{\mathit{f}}}$ = 0.30 (*n*-pentane:Et_2O 15:1).

^{1}H NMR (400 MHz, $CDCl_3$) δ 7.68 – 7.63 (m, 4H), 6.49 (d, *J* = 2.3 Hz, 1H), 2.69 (d, *J* = 2.3 Hz, 1H), 2.13 (s, 3H).

^{19}F{^{1}H} NMR (377 MHz, $CDCl_3$) δ -62.78 (s, 3F).

GC-EI-MS: (*m/z*) requires: [($C_{12}H_9F_3O_2$)] = 242.0555, (*m/z*) found: [($C_{12}H_9F_3O_2$)] = 242.0547.

Analytical data is in agreement with literature values.[16]

(*Z*)-(3-(4-(trifluoromethyl)phenyl)prop-2-ene-1,2-diyl)dibenzene (41)

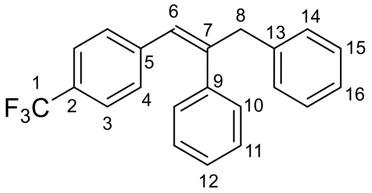

Compound **39** was prepared according to the General Procedure **C** using 1-(propa-1,2-dien-1-yl)-4-(trifluoromethyl)benzene (**S16**) (18.4 mg, 0.1 mmol, 1.0 eq.) and benzene (351.5 mg, 4.5 mmol, 45.0 eq.). After workup, the crude mixture was analysed by ^{19}F NMR (55%, *Z*:*E* = 5.5:1). Purification by column chromatography (*n*-pentane:DCM 20:1) yielded the title compound (colorless oil, 17.0 mg, 0.050 mmol, 50%, *Z*:*E* = 17:1).

$\mathbf{R_f}$ = 0.10 (*n*-pentane).

^{1}H NMR (500 MHz, $CDCl_3$) δ 7.34 (d, $^{3}J_{HH}$ = 8.3 Hz, 2H, H-C3), 7.31 – 7.28 (m, 2H, H-C15), 7.28 – 7.24 (m, 3H, H-C10, H-C12), 7.24 – 7.20 (m, 3H, H-C14, H-C16), 7.08 – 7.04 (m, 2H, H-C11), 7.02 (d, $^{3}J_{HH}$ = 8.0 Hz, 2H, H-C4), 6.45 (s, 1H, H-C6), 3.83 (s, 2H, H-C8).

^{13}C NMR (126 MHz, $CDCl_3$) δ 145.13 (C9), 140.99 (C5), 140.60 (C7), 138.76 (C13), 129.44 (C14), 129.29 (C4), 128.77 (C10), 128.58 (C11), 128.54 (C15), 128.24 (app. q, $^{2}J_{CF}$ = 32.2 Hz, C2), 127.51 (C12), 126.84 (C6), 126.55 (C16), 124.88 (q, $^{3}J_{CF}$ = 3.8 Hz, C3), 124.34 (app. q, $^{1}J_{CF}$ = 271.6 Hz, C1), 47.08 (C8).

^{19}F NMR (470 MHz, $CDCl_3$) δ -62.51 (s, 3F, F-C1).

^{19}F{^{1}H} NMR (377 MHz, $CDCl_3$) δ -62.51 (s, 3F, F-C1).

GC-EI-MS: (*m/z*) requires: [($C_{22}H_{17}F_3$)] = 338.1282, (*m/z*) found: [($C_{22}H_{17}F_3$)] = 338.1277.

FT-IR ($\tilde{\nu}$ = cm^{-1}): 2921 (m), 2852 (m), 1612 (w), 1457 (w), 1320 (s), 1222 (w), 1165 (m), 1124 (s), 1067 (s), 1015 (m), 883 (w), 837 (w), 700 (m).

(*Z*)-1-(2,3-dichloroprop-1-en-1-yl)-4-(trifluoromethyl)benzene (42)

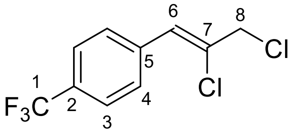

Compound **42** was prepared according to the General Procedure **C** using 1-(propa-1,2-dien-1-yl)-4-(trifluoromethyl)benzene (**S16**) (18.4 mg, 0.1 mmol, 1.0 eq.), Selectfluor® (42.5 mg, 0.12 mmol, 1.2 eq.) and CsCl (50.5 mg, 0.3 mmol, 3.0 eq.). After workup, the crude mixture was analysed by ^{19}F NMR (70%, *Z*:*E* = 2.5:1). Purification by column chromatography (*n*-pentane:DCM 20:1) yielded the title compound **40** as a colorless oil (12.7 mg, 0.050 mmol, 50%)

$\mathbf{R_f}$ = 0.25 (*n*-pentane).

^{1}H NMR (400 MHz, $CDCl_3$) δ 7.74 (d, $^{3}J_{HH}$ = 8.2 Hz, 2H, H-C4), 7.64 (d, $^{3}J_{HH}$ = 8.3 Hz, 2H, H-C3), 6.90 (s, 1H, H-C6), 4.36 (s, 2H, H-C8).

^{13}C NMR (126 MHz, $CDCl_3$) δ 137.33 (C5), 131.46 (C7), 130.48 (app. q, $^2J_{CF}$ = 32.2 Hz, C2), 129.63 (C4), 127.24 (C6), 125.43 (q, $^3J_{CF}$ = 3.8 Hz, C3), 124.07 (app. q, $^1J_{CF}$ = 272.9 Hz, C1), 49.87 (C8).

^{19}F NMR (470 MHz, $CDCl_3$) δ -62.81 (s, 3F, F-C1).

^{19}F{^{1}H} NMR (377 MHz, $CDCl_3$) δ -62.81 (s, 3F, F-C1).

GC-EI-MS: (*m/z*) requires: [($C_{10}H_7F_3Cl_2$)] = 253.9877, (*m/z*) found: [($C_{10}H_7F_3Cl_2$)] = 253.9877.

FT-IR ($\tilde{\nu}$ = cm^{-1}): 1618 (w), 1411 (w), 1320 (s), 1262 (m), 1165 (s), 1113 (s), 1067 (s), 1015 (m), 929 (w), 872 (m), 826 (m), 711 (m), 660 (m), 625 (w).

Unsuccessful result

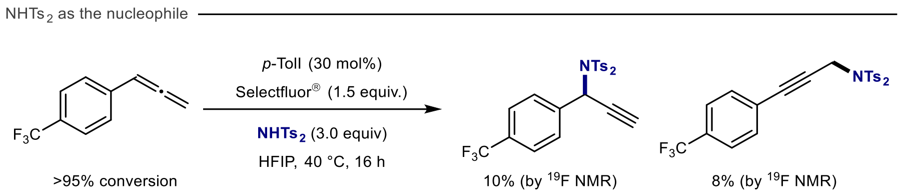

1.7 NMR-Spectra of key compounds

12-bromododeca-1,2-diene (S3)

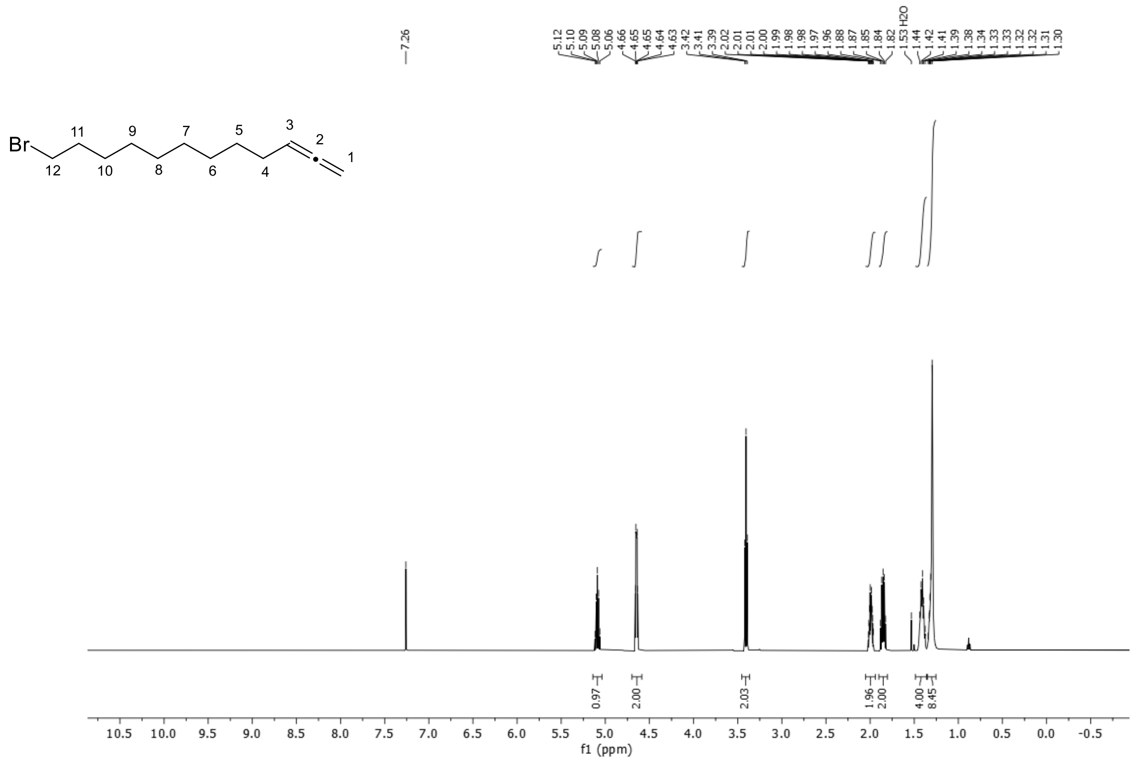

Supplementary Figure 1. ^{1}H NMR of **S3** (599 MHz, 299 K, $CDCl_3$).

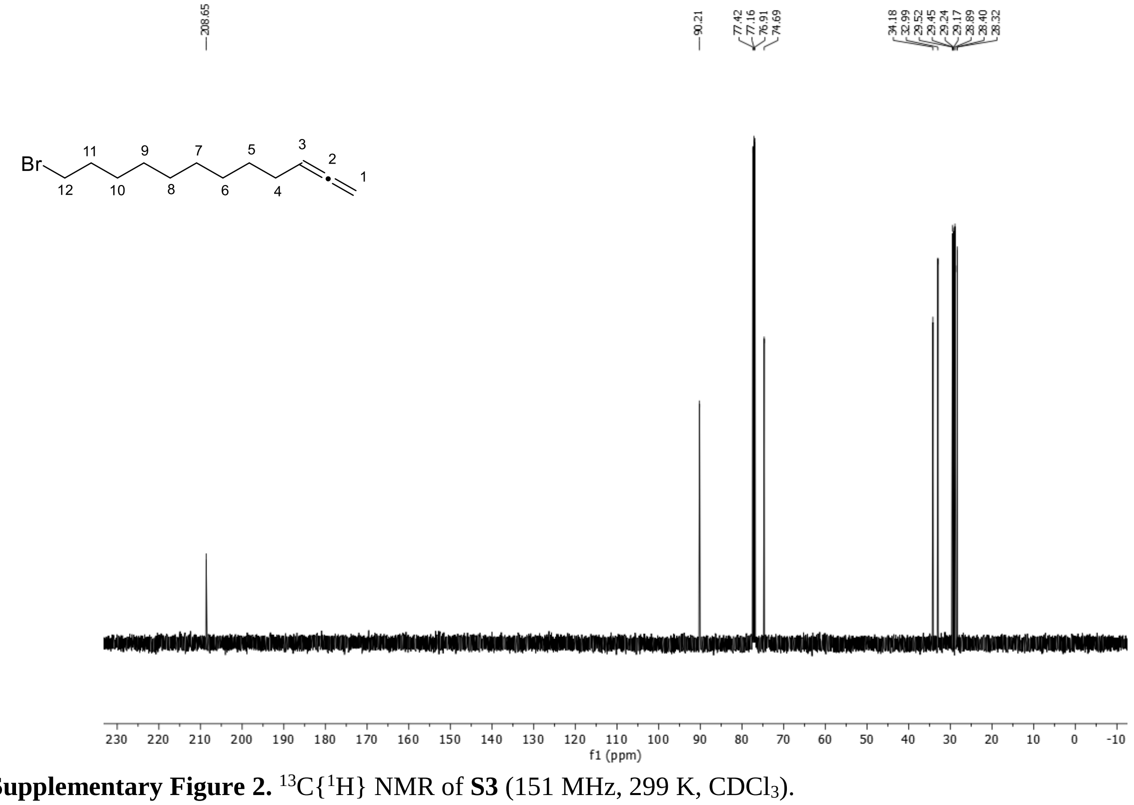

Supplementary Figure 2. $^{13}C\{^{1}H\}$ NMR of **S3** (151 MHz, 299 K, $CDCl_3$).

4-((Dodeca-10,11-dien-1-yloxy)methyl)-1,2-difluorobenzene (S4)

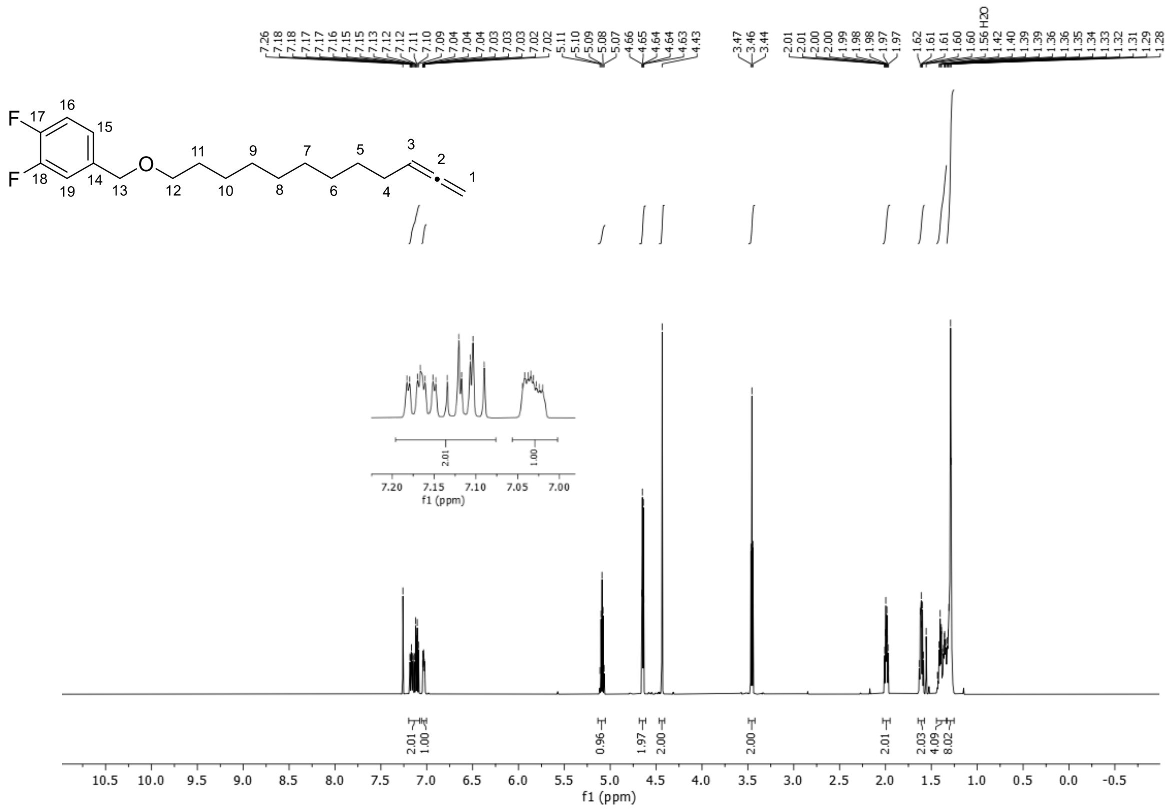

Supplementary Figure 3. ^{1}H NMR of **S4** (599 MHz, 299 K, $CDCl_3$).

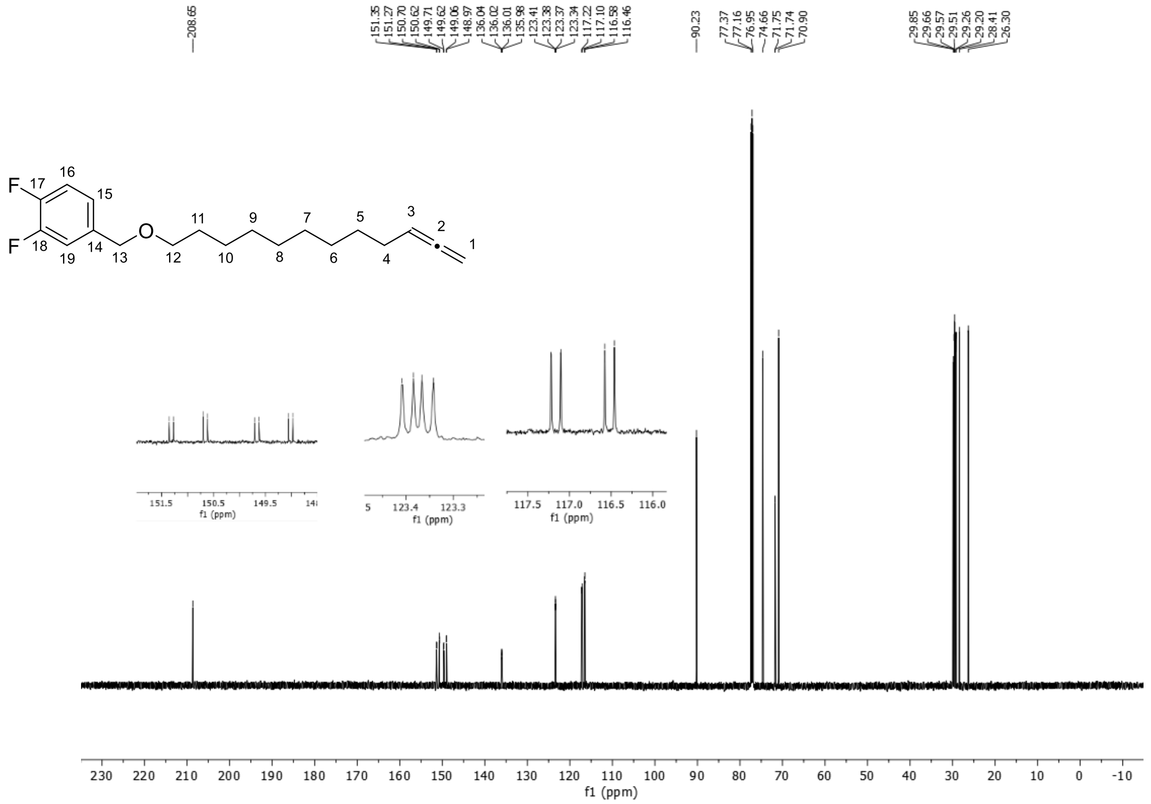

Supplementary Figure 4. $^{13}C\{^{1}H\}$ NMR of **S4** (151 MHz, 299 K, $CDCl_3$).

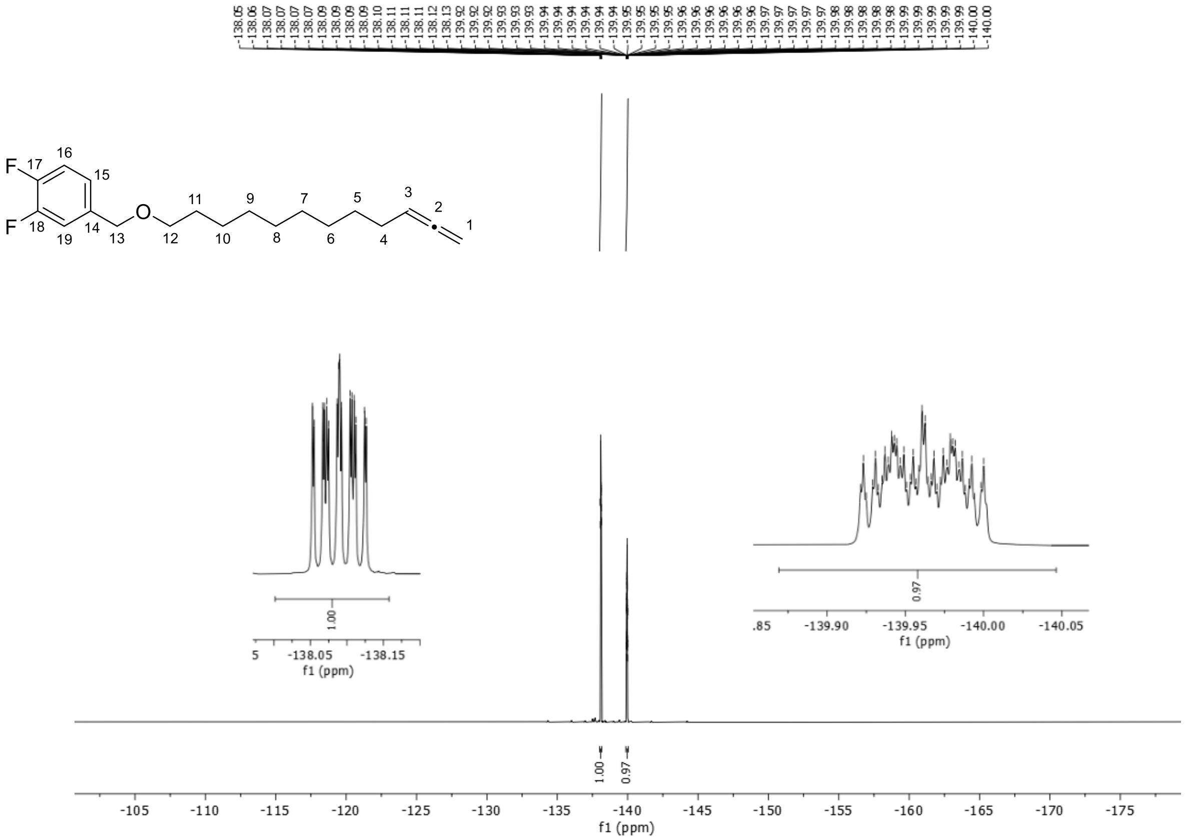

Supplementary Figure 5. ^{19}F NMR of **S4** (564 MHz, 299 K, $CDCl_3$).

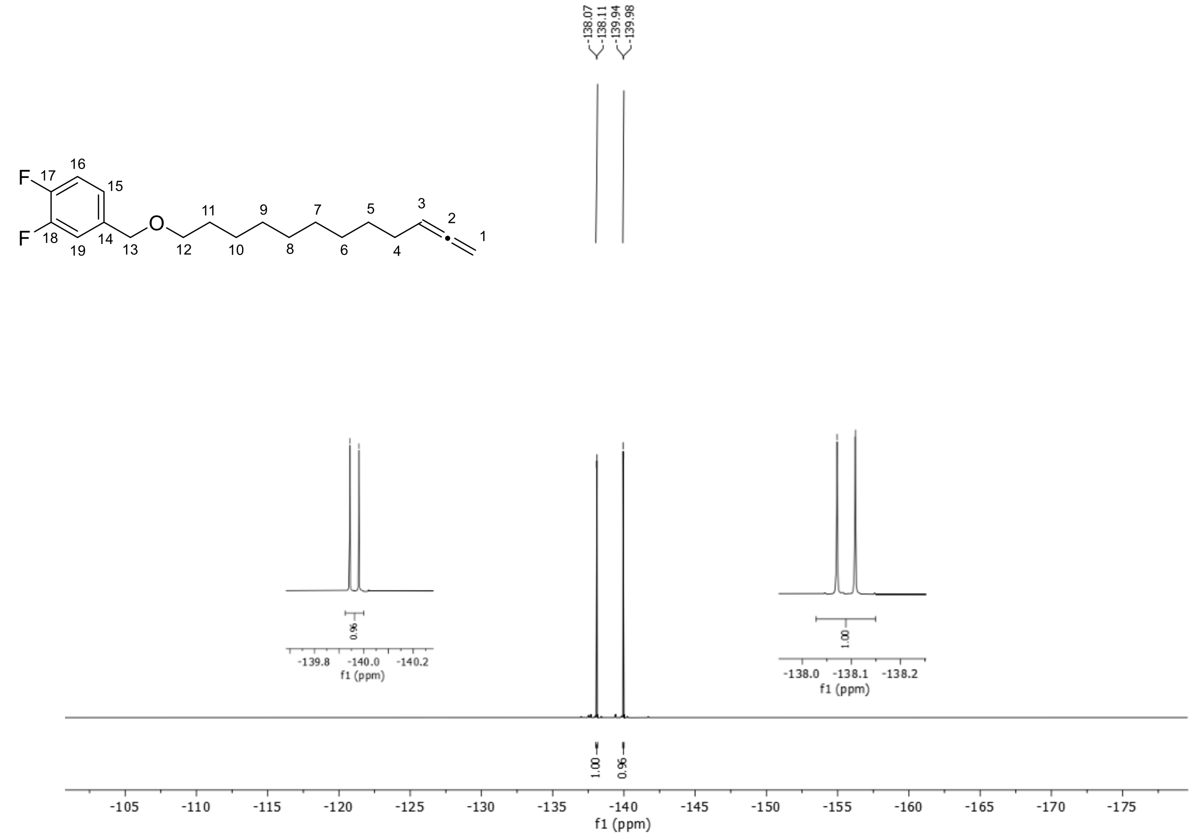

Supplementary Figure 6. $^{19}F\{^1H\}$ NMR of **S4** (564 MHz, 299 K, $CDCl_3$).

Dodeca-10,11-dien-1-yl 4-methylbenzenesulfonate (S7)

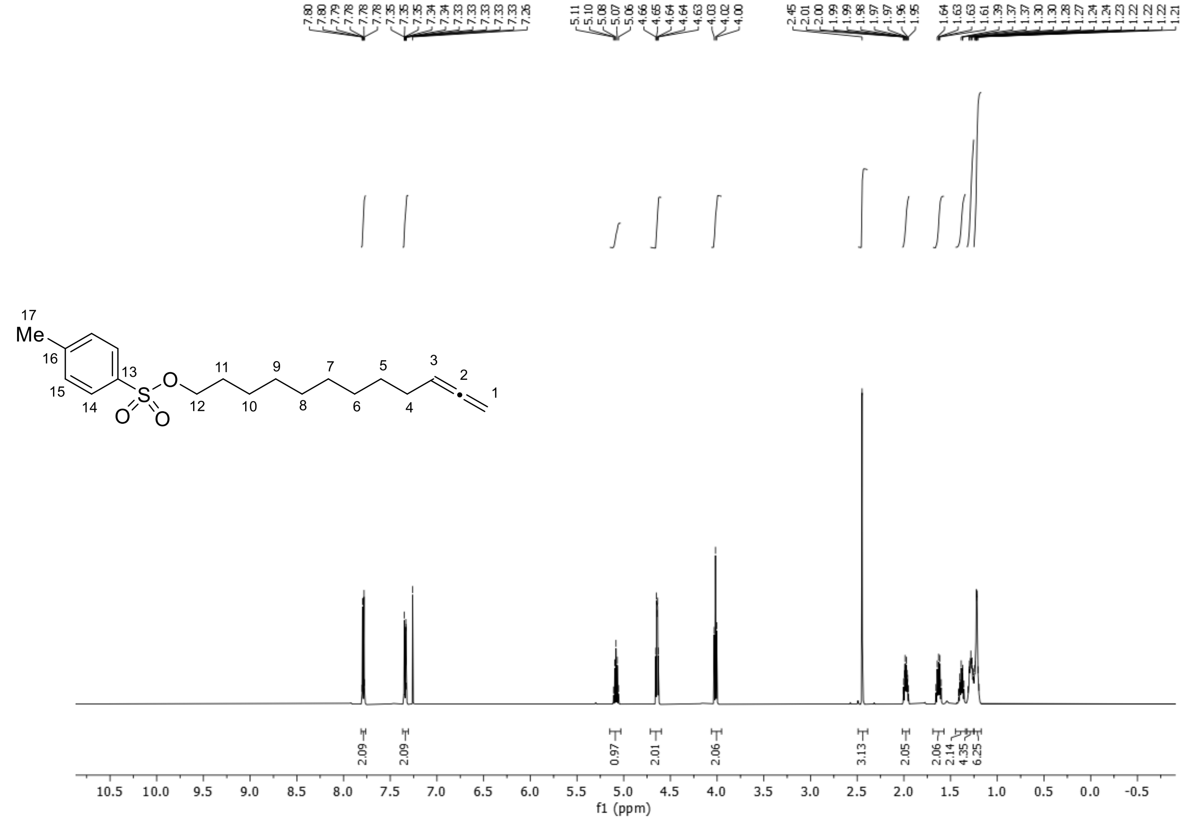

Supplementary Figure 7. ^{1}H NMR of **S7** (500 MHz, 299 K, $CDCl_3$).

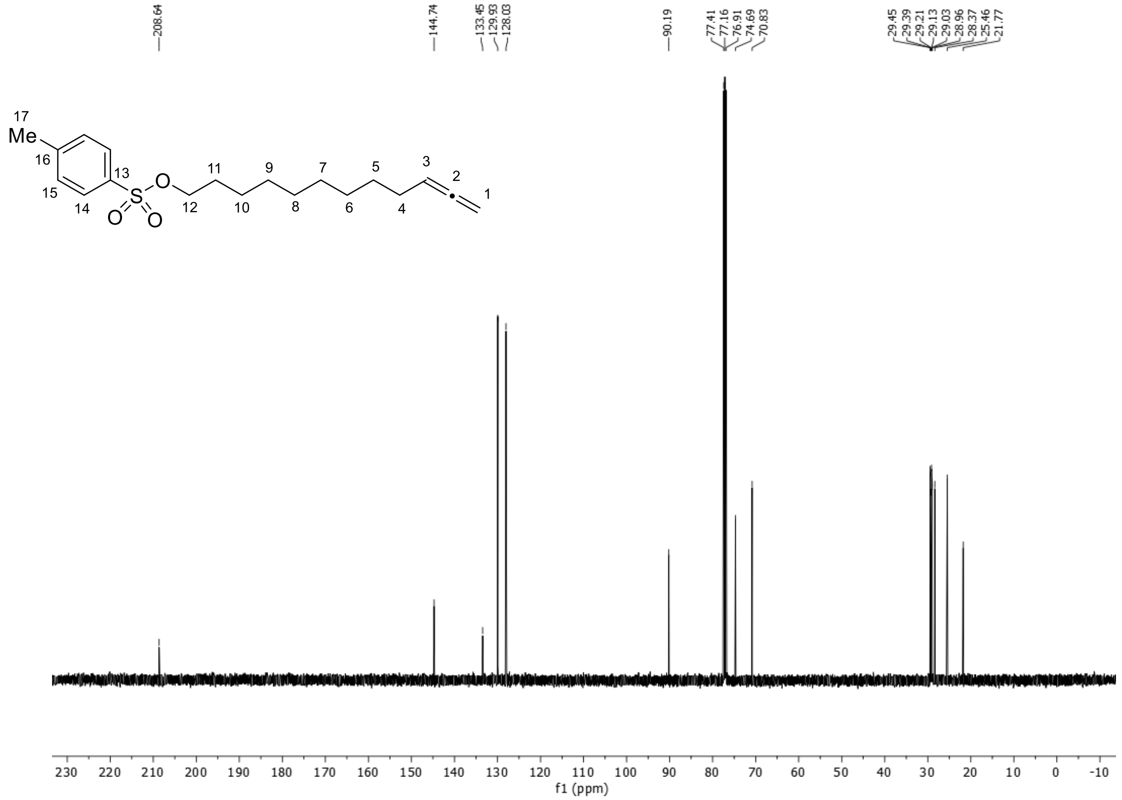

Supplementary Figure 8. $^{13}C\{^{1}H\}$ NMR of **S7** (126 MHz, 299 K, $CDCl_3$).

2-(Dodeca-10,11-dien-1-yl)isoindoline-1,3-dione (S8)

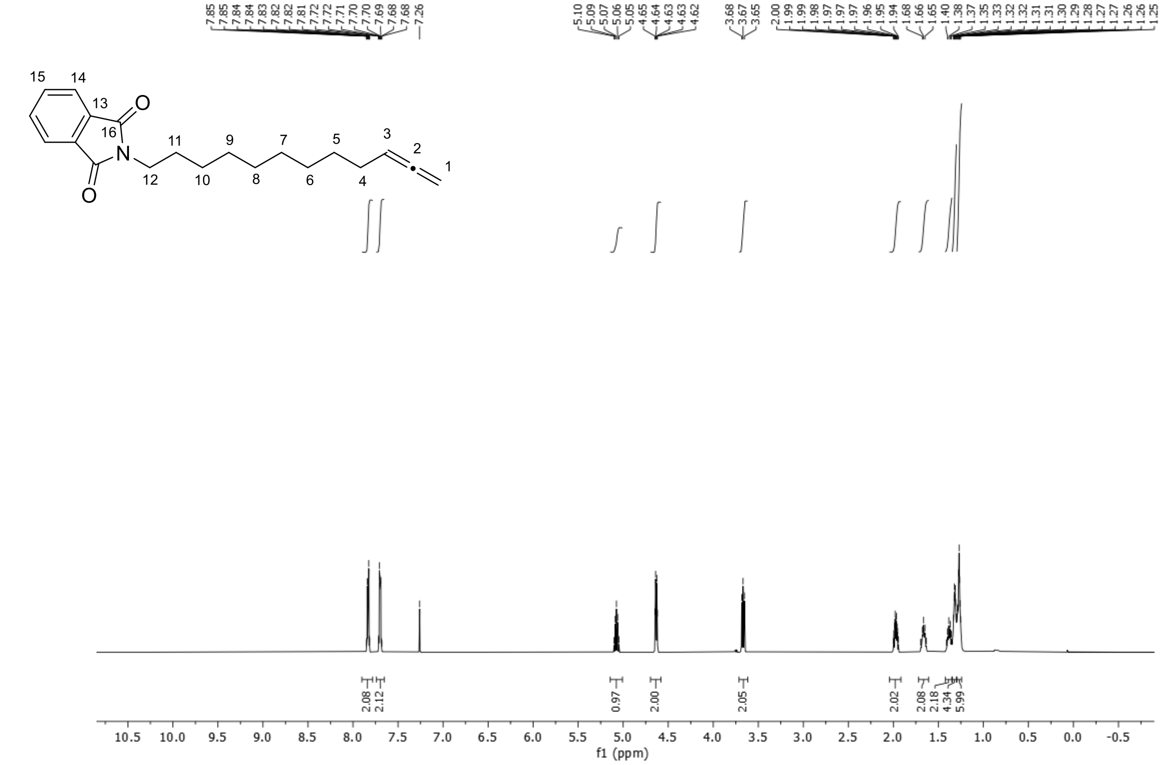

Supplementary Figure 9. ^{1}H NMR of **S8** (500 MHz, 299 K, $CDCl_3$).

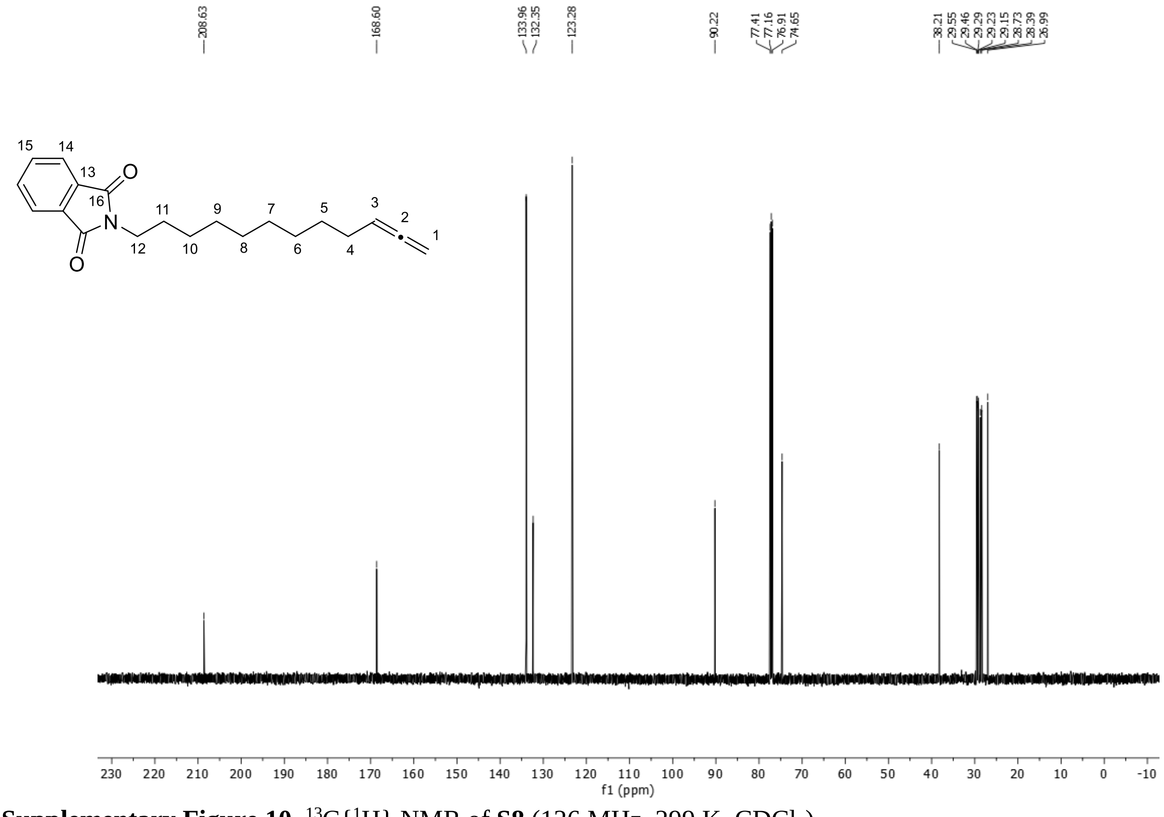

Supplementary Figure 10. $^{13}C\{^{1}H\}$ NMR of **S8** (126 MHz, 299 K, $CDCl_3$).

Dodeca-10,11-dien-1-yl nicotinate (S9)

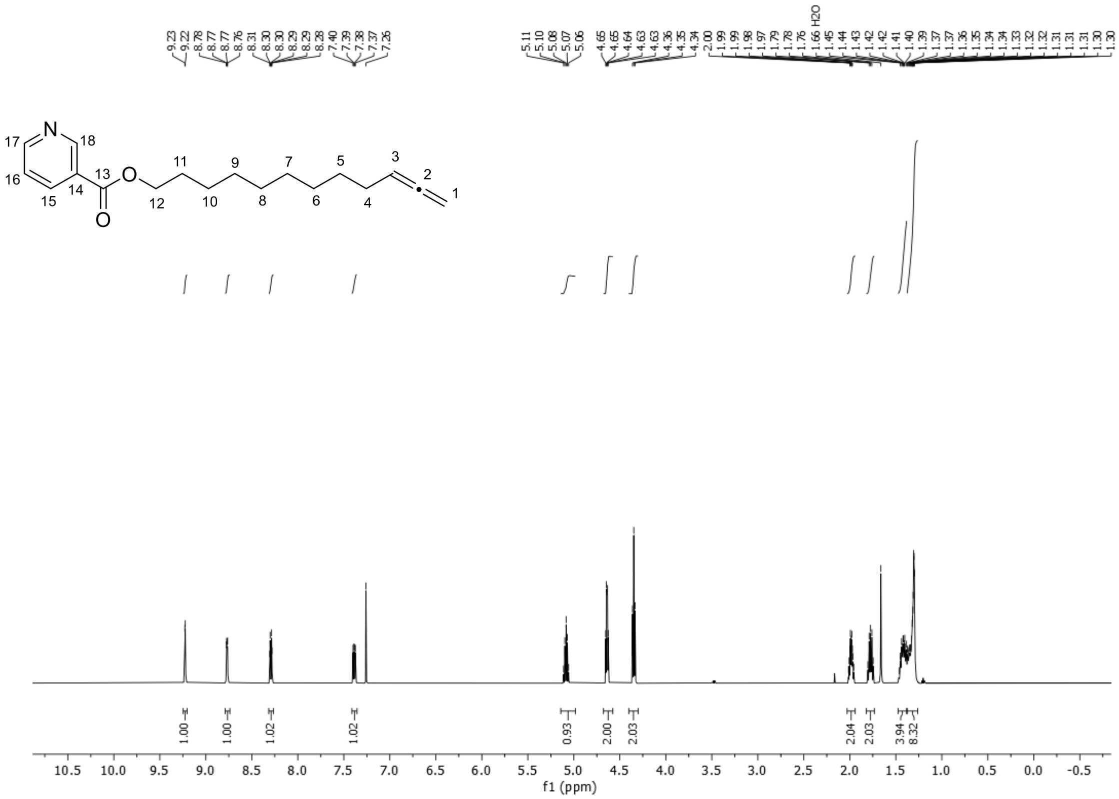

Supplementary Figure 11. ^{1}H NMR of **S9** (500 MHz, 299 K, $CDCl_3$).

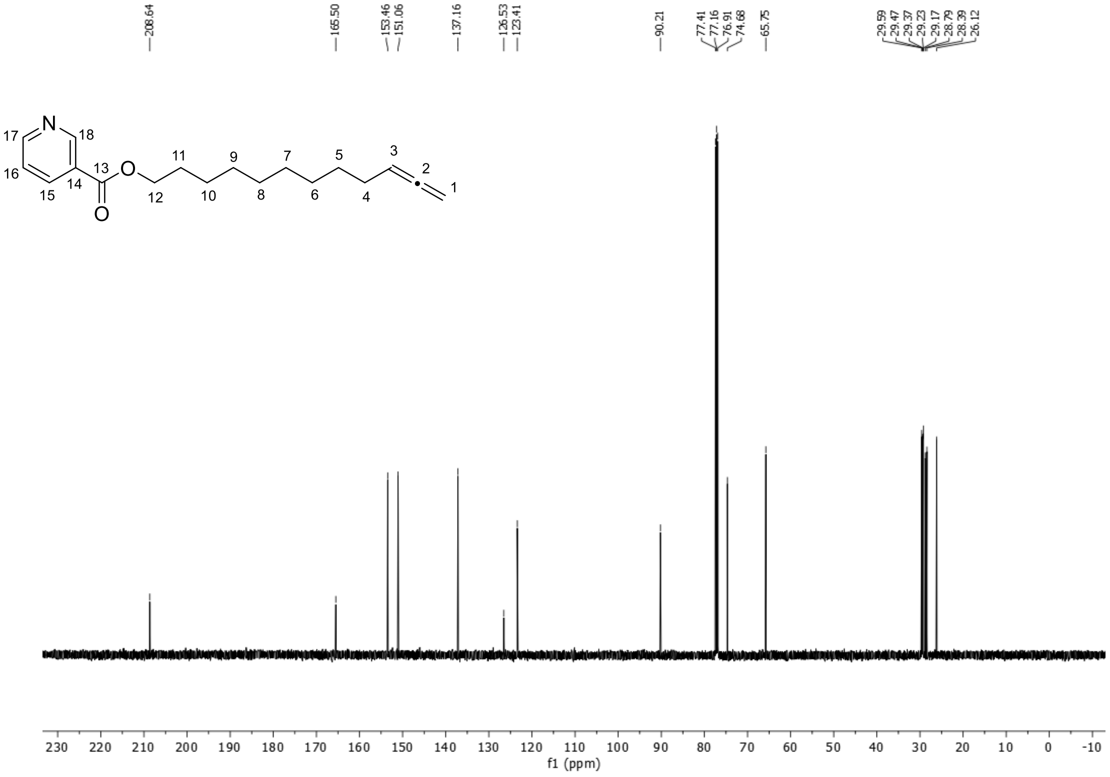

Supplementary Figure 12. ^{13}C{^{1}H} NMR of **S9** (126 MHz, 299 K, $CDCl_3$).

Dodeca-10,11-dien-1-yl 2-methylthiazole-4-carboxylate (S10)

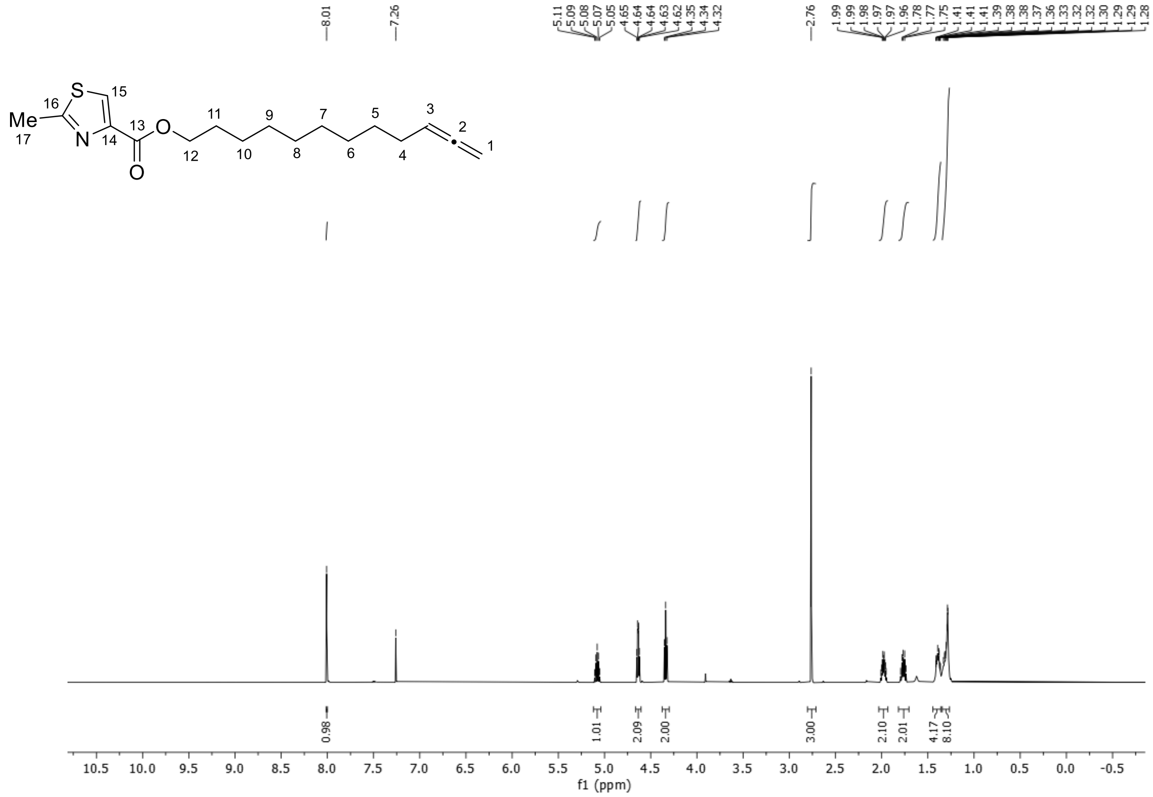

Supplementary Figure 13. ^{1}H NMR of **S10** (500 MHz, 299 K, $CDCl_3$).

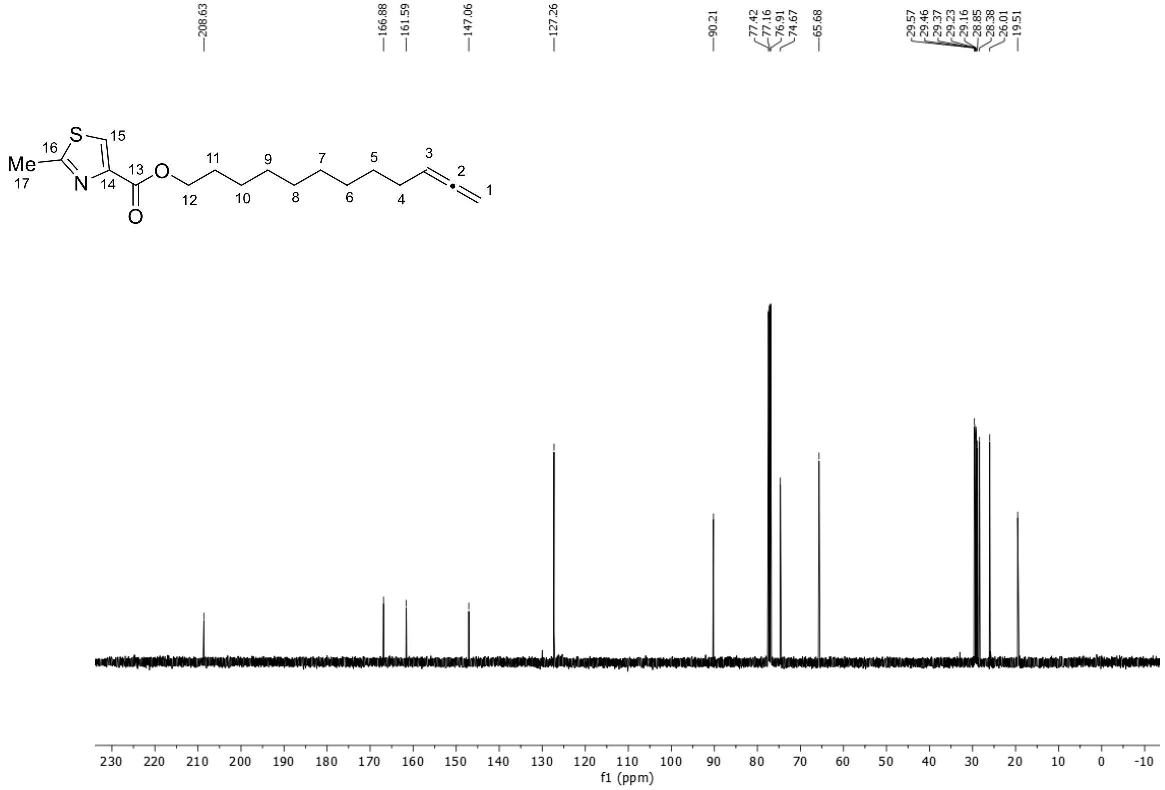

Supplementary Figure 14. $^{13}C\{^{1}H\}$ NMR of **S10** (126 MHz, 299 K, $CDCl_3$).

Dodeca-10,11-dien-1-yl cyclopropanecarboxylate (S11)

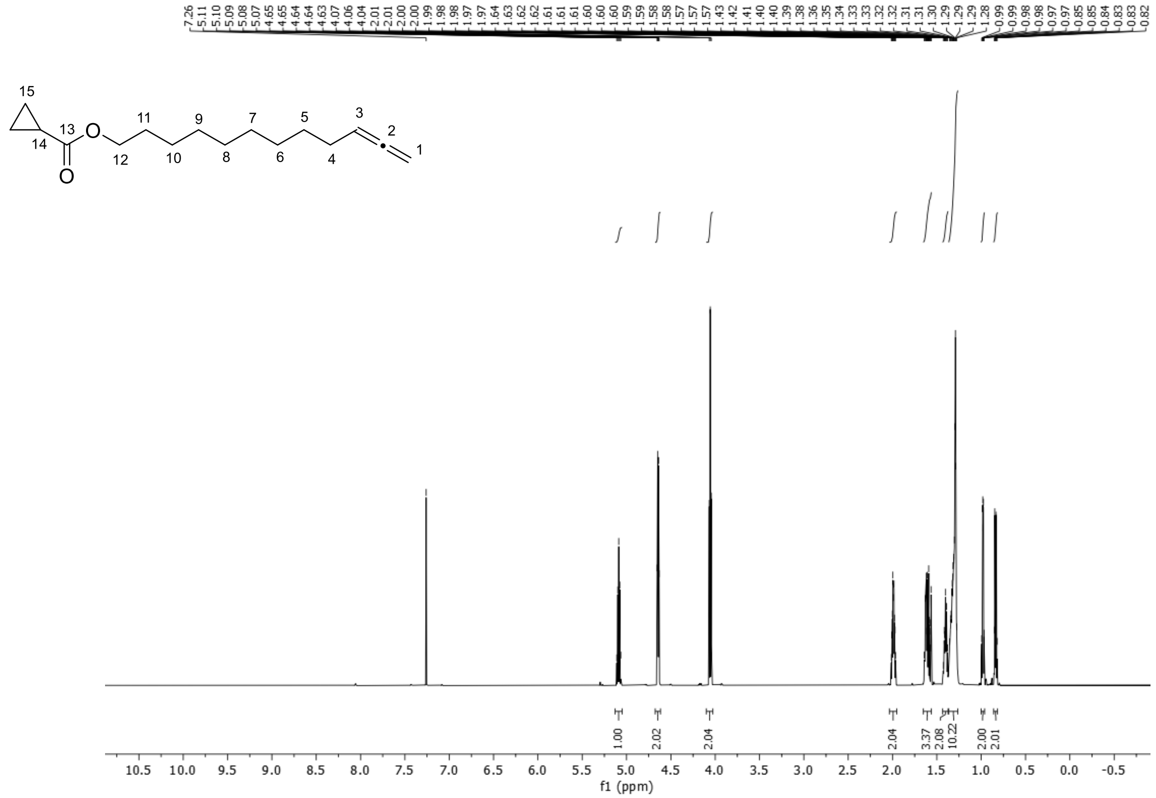

Supplementary Figure 15. ^{1}H NMR of **S11** (599 MHz, 299 K, $CDCl_3$).

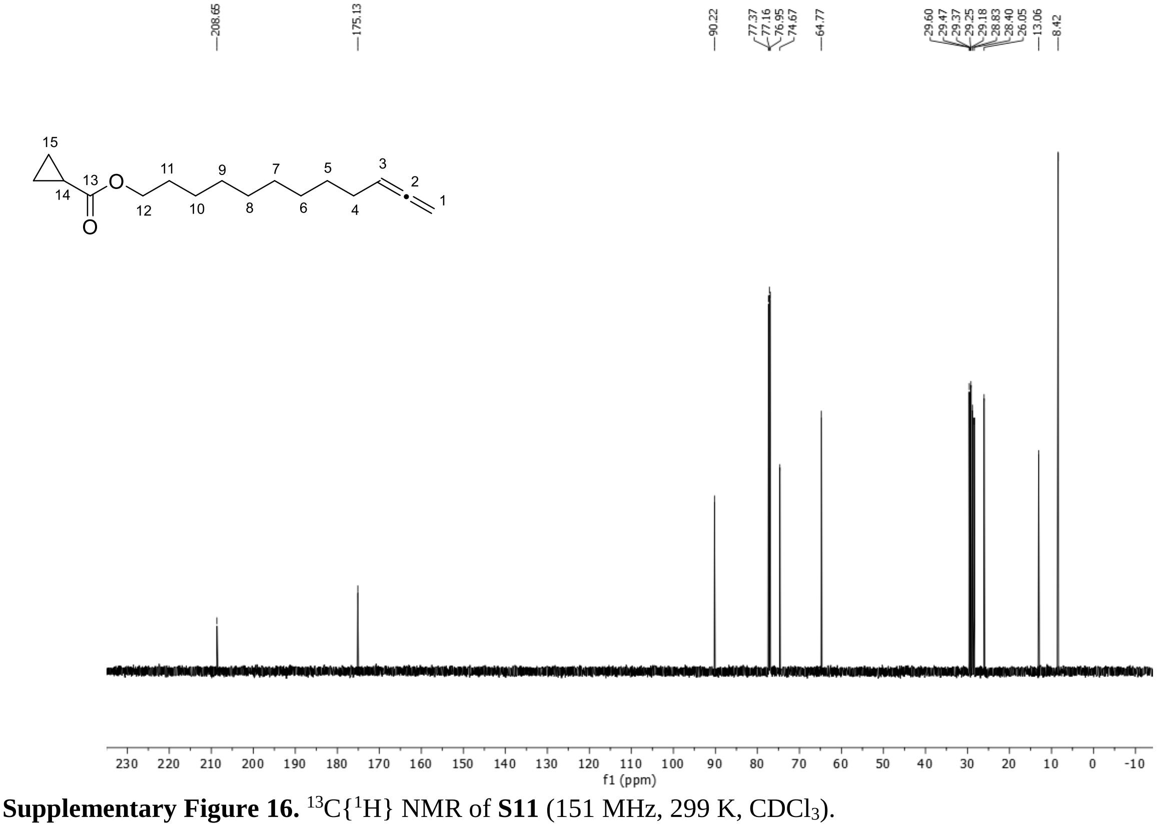

Supplementary Figure 16. $^{13}C\{^{1}H\}$ NMR of **S11** (151 MHz, 299 K, $CDCl_3$).

(8R,9S,13S,14S)-13-Methyl-17-oxo-7,8,9,11,12,13,14,15,16,17-decahydro-6H-cyclopenta[a]phenanthren-3-yl dodeca-10,11-dienoate (S13)

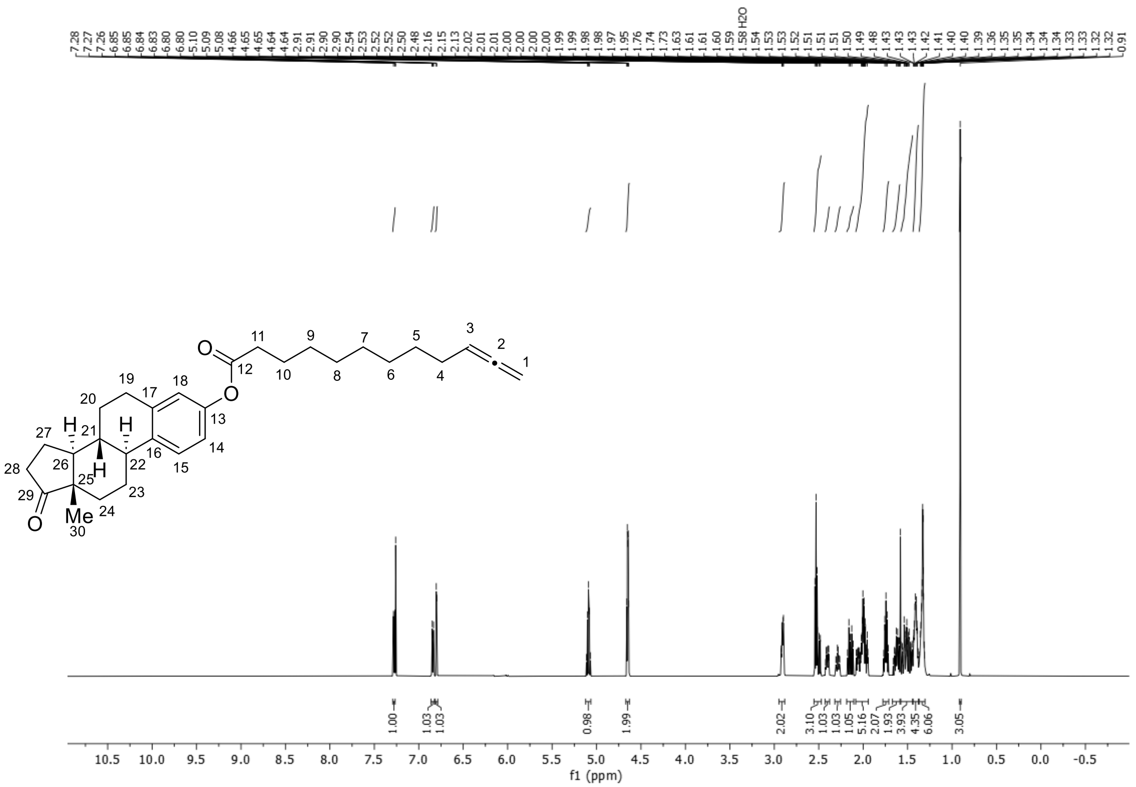

Supplementary Figure 17. ^{1}H NMR of **S13** (599 MHz, 299 K, $CDCl_3$).

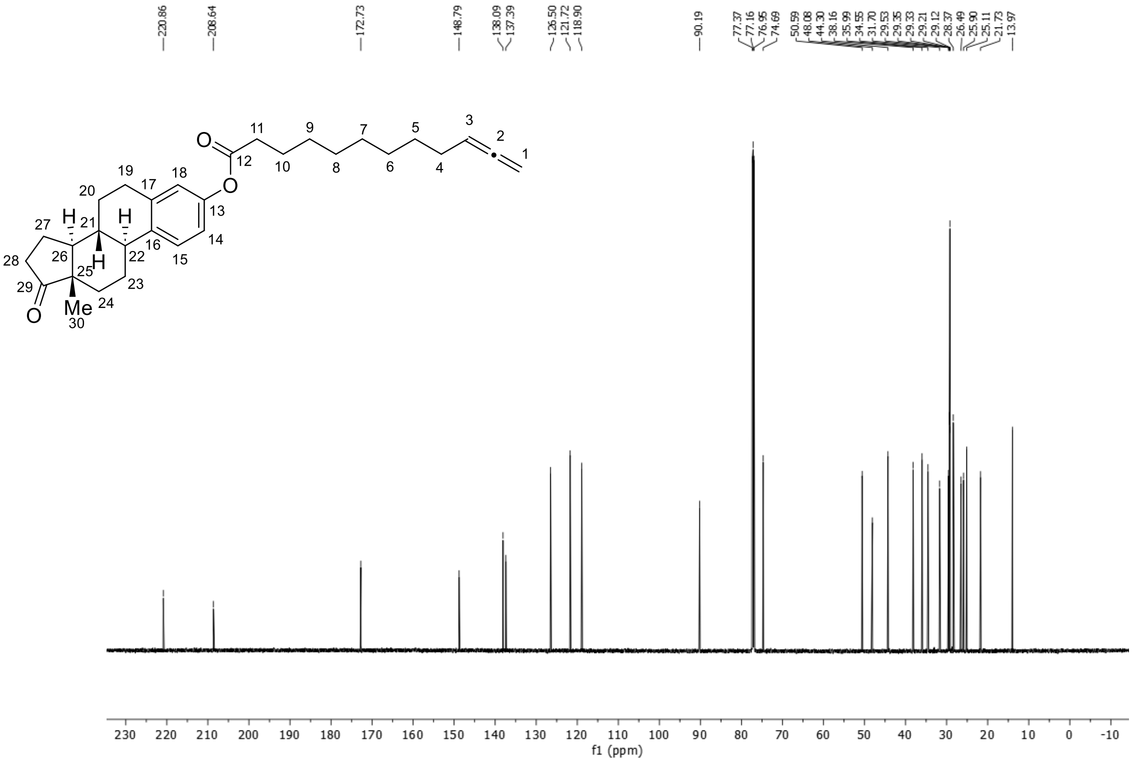

Supplementary Figure 18. $^{13}C\{^{1}H\}$ NMR of **S13** (151 MHz, 299 K, $CDCl_3$).

Dodeca-10,11-dien-1-yl 2-(4-isobutylphenyl)propanoate (S14)

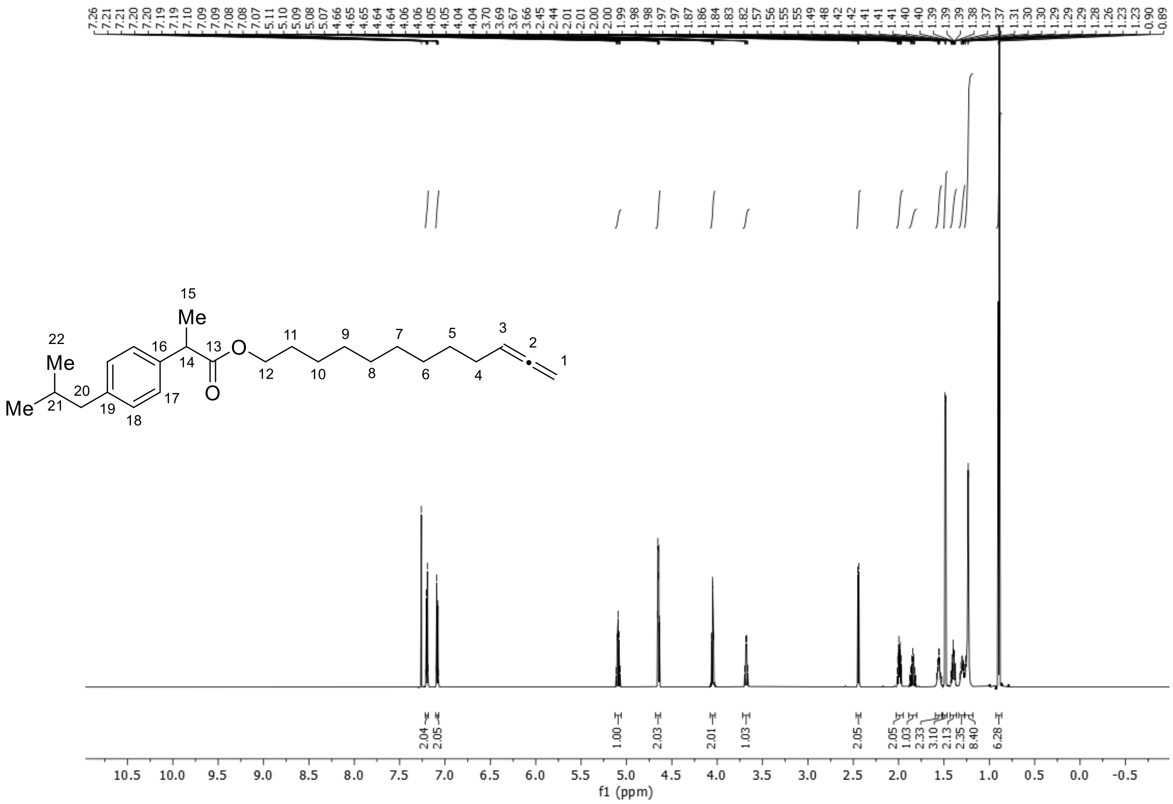

Supplementary Figure 19. ^{1}H NMR of **S14** (599 MHz, 299 K, $CDCl_3$).

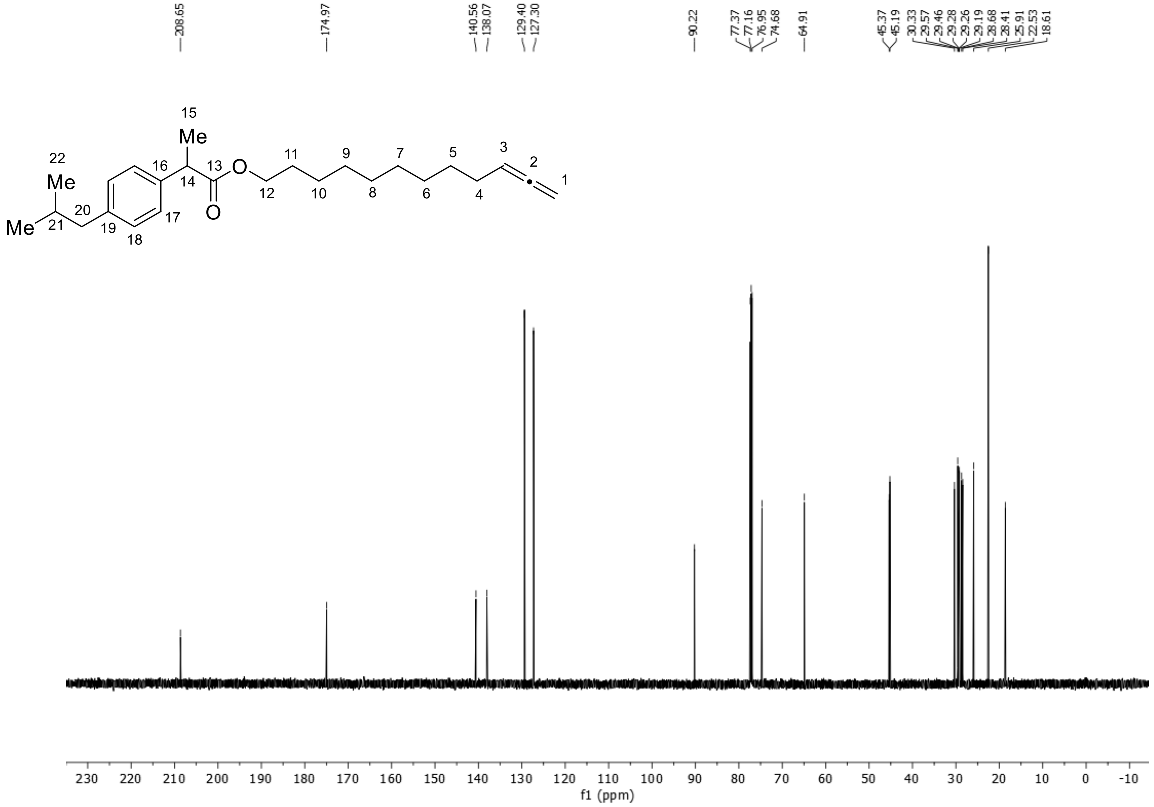

Supplementary Figure 20. $^{13}C\{^{1}H\}$ NMR of **S14**(151 MHz, 299 K, $CDCl_3$).

Dodeca-10,11-dien-1-yl (E)-3-(4-bromophenyl)acrylate (S15)

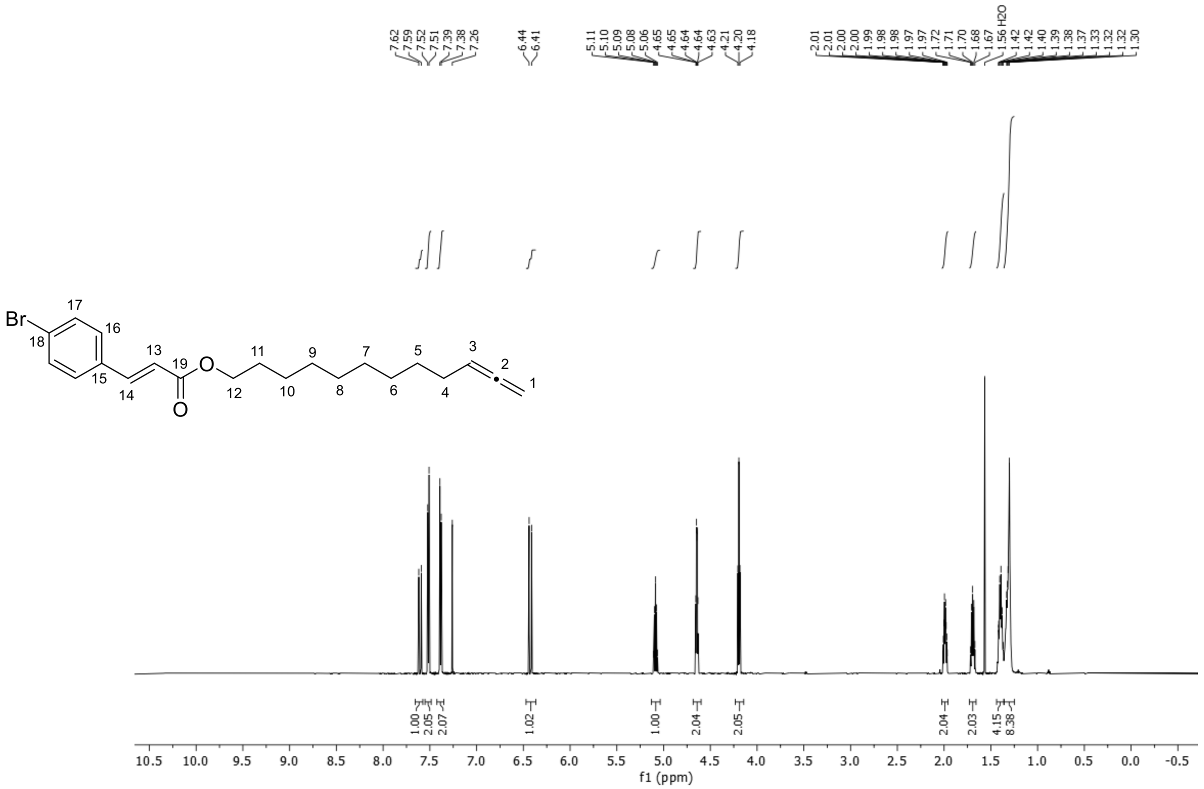

Supplementary Figure 21. ^{1}H NMR of **S15** (400 MHz, 299 K, $CDCl_3$).

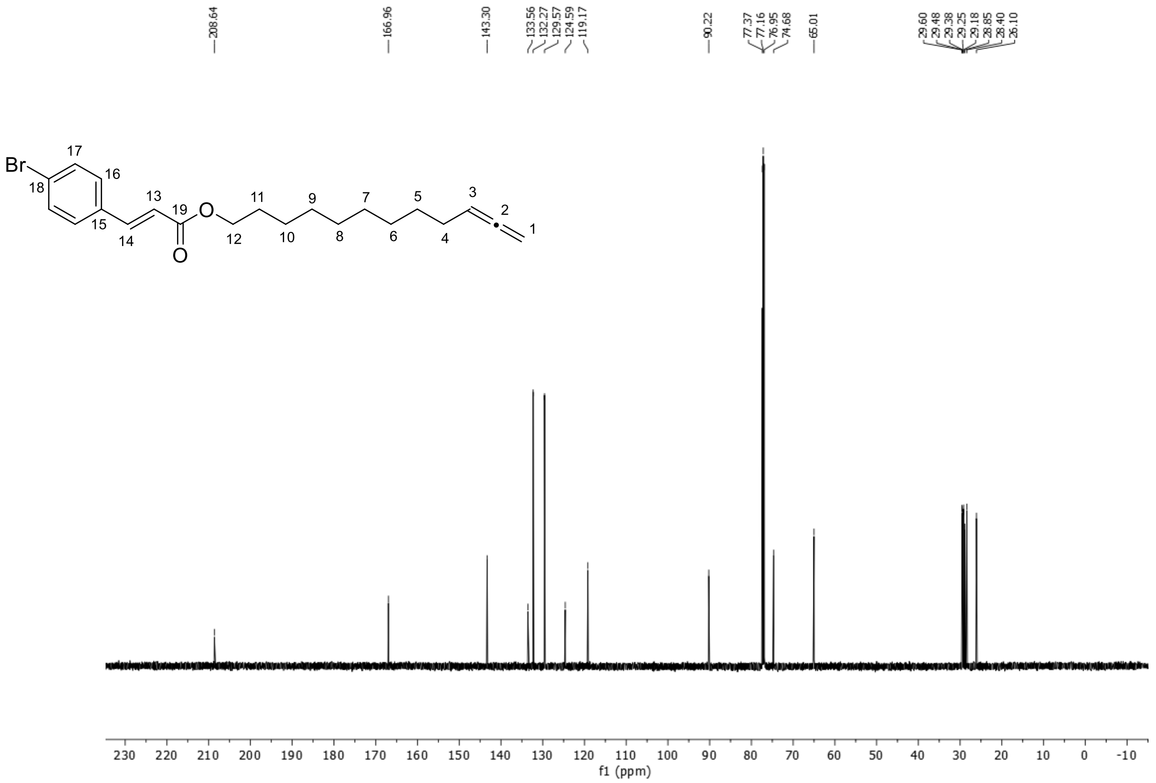

Supplementary Figure 22. $^{13}C\{^{1}H\}$ NMR of **S15** (151 MHz, 299 K, $CDCl_3$).

Morpholino(4-(propa-1,2-dien-1-yl)phenyl)methanone (S19)

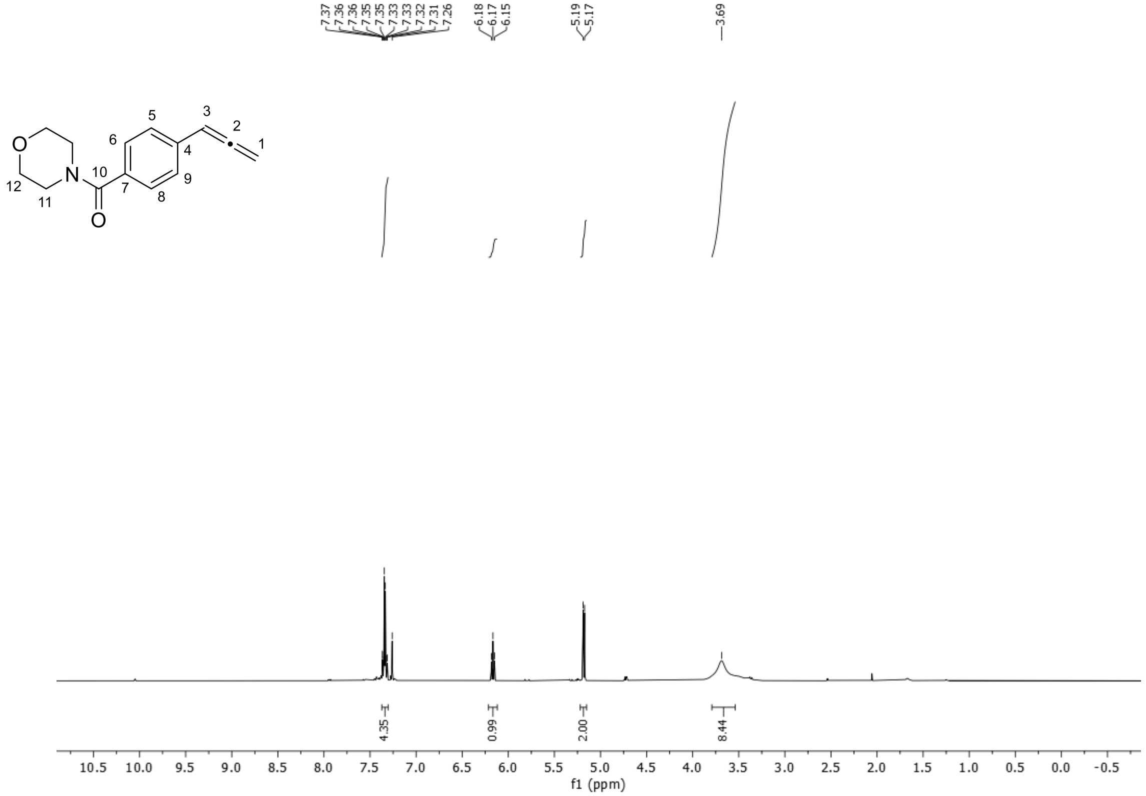

Supplementary Figure 23. ^{1}H NMR of **S19** (400 MHz, 299 K, $CDCl_3$).

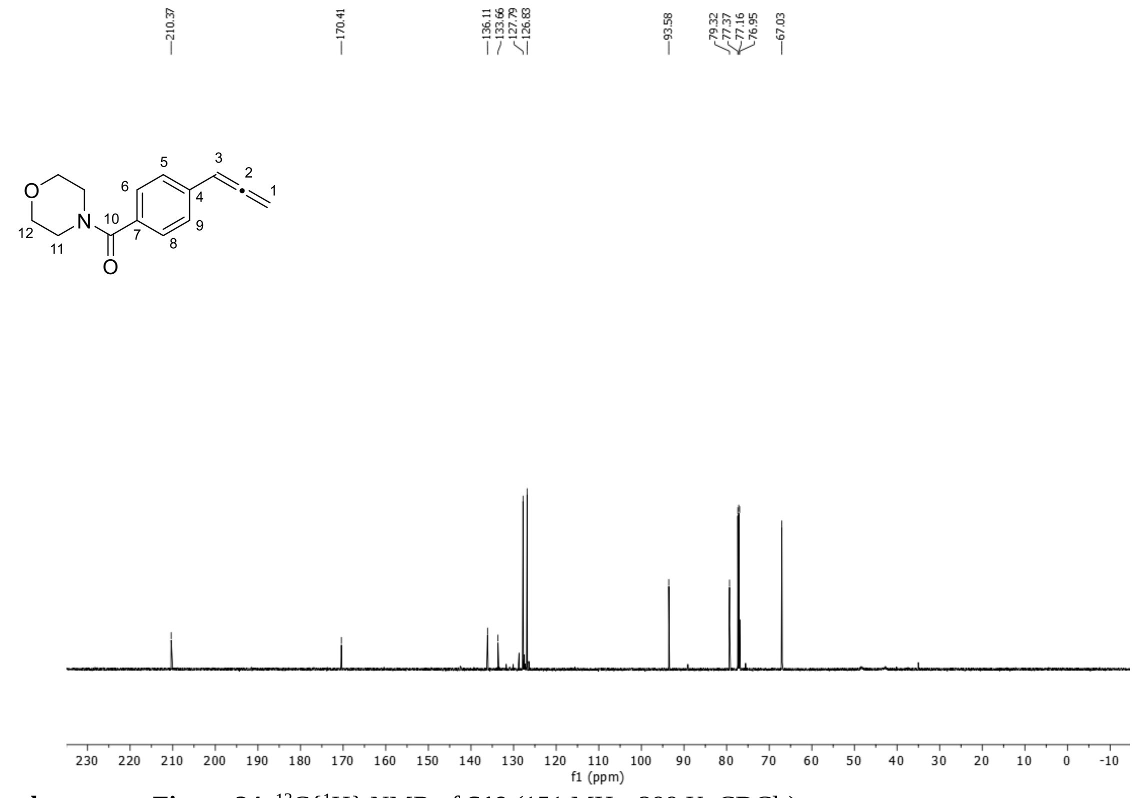

Supplementary Figure 24. $^{13}C\{^{1}H\}$ NMR of **S19** (151 MHz, 299 K, $CDCl_3$).

4-((Dodeca-10,11-dien-1-yloxy)methyl)-1,2-difluorobenzene (S20)

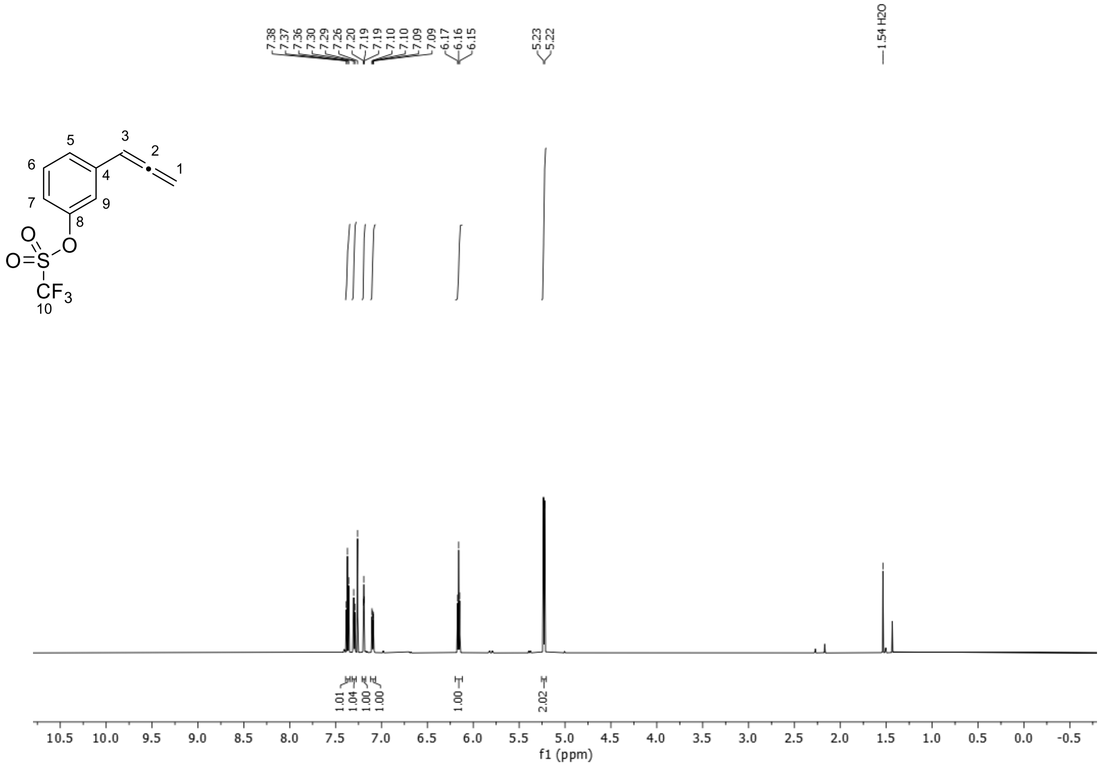

Supplementary Figure 25. ^{1}H NMR of **S20** (599 MHz, 299 K, $CDCl_3$).

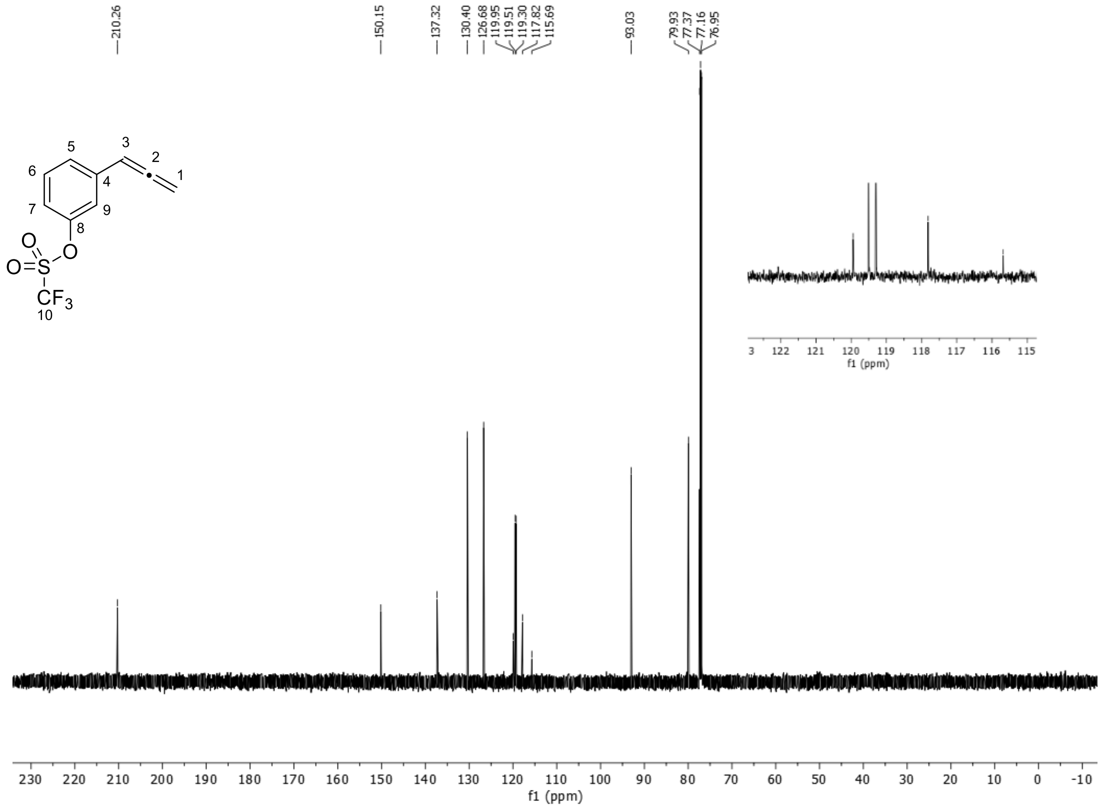

Supplementary Figure 26. $^{13}C\{^1H\}$ NMR of **S20** (151 MHz, 299 K, $CDCl_3$).

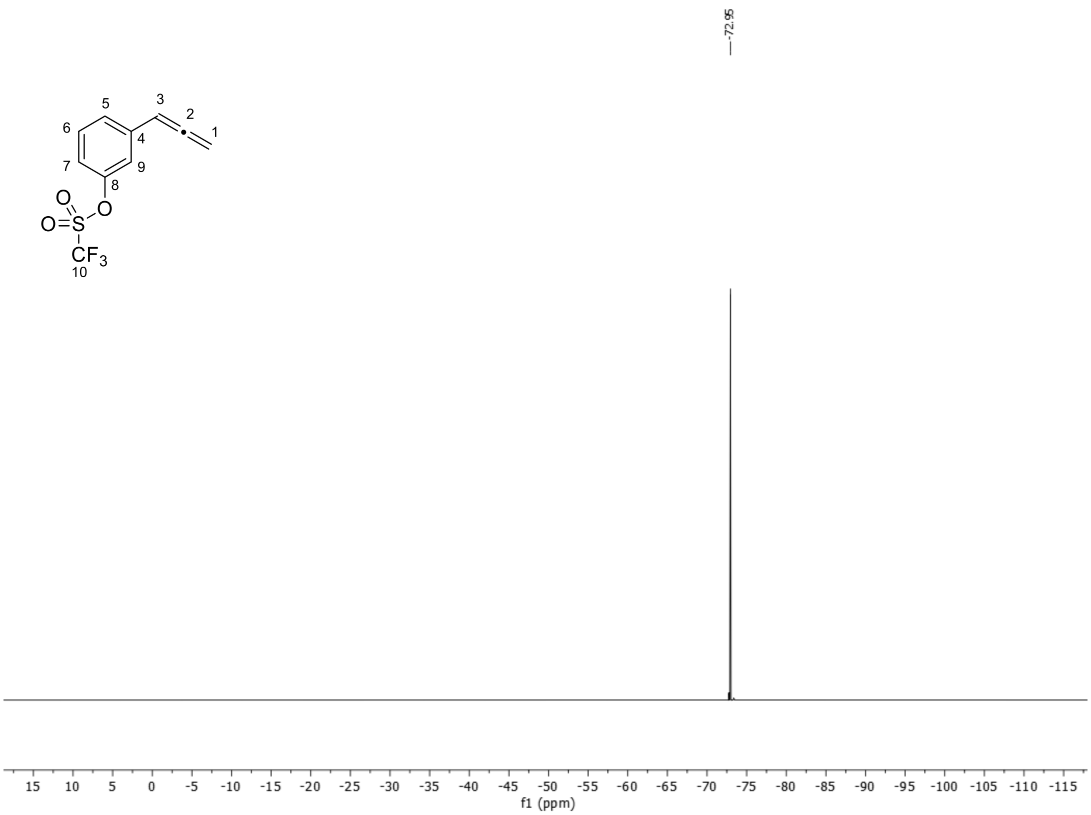

Supplementary Figure 27. ^{19}F NMR of **S20** (564 MHz, 299 K, $CDCl_3$).

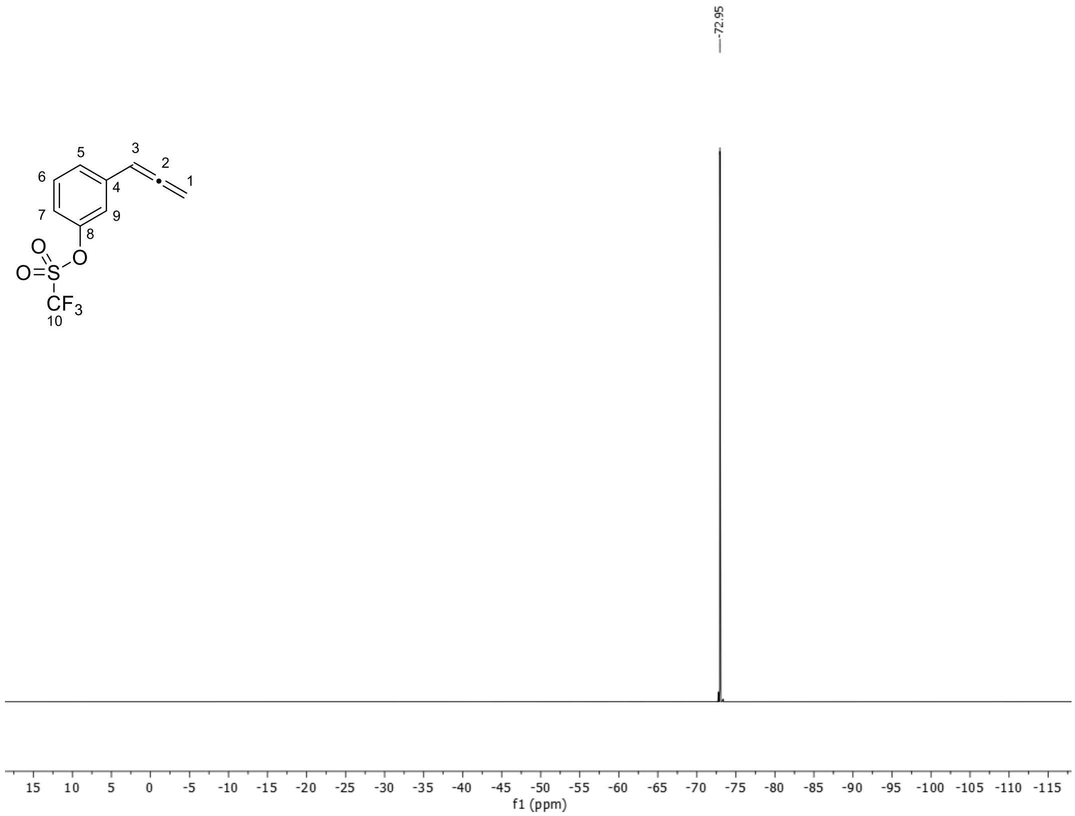

Supplementary Figure 28. ^{19}F{^{1}H} NMR of **S20** (564 MHz, 299 K, $CDCl_3$).

1-(Methylsulfonyl)-4-(propa-1,2-dien-1-yl)benzene (S21)

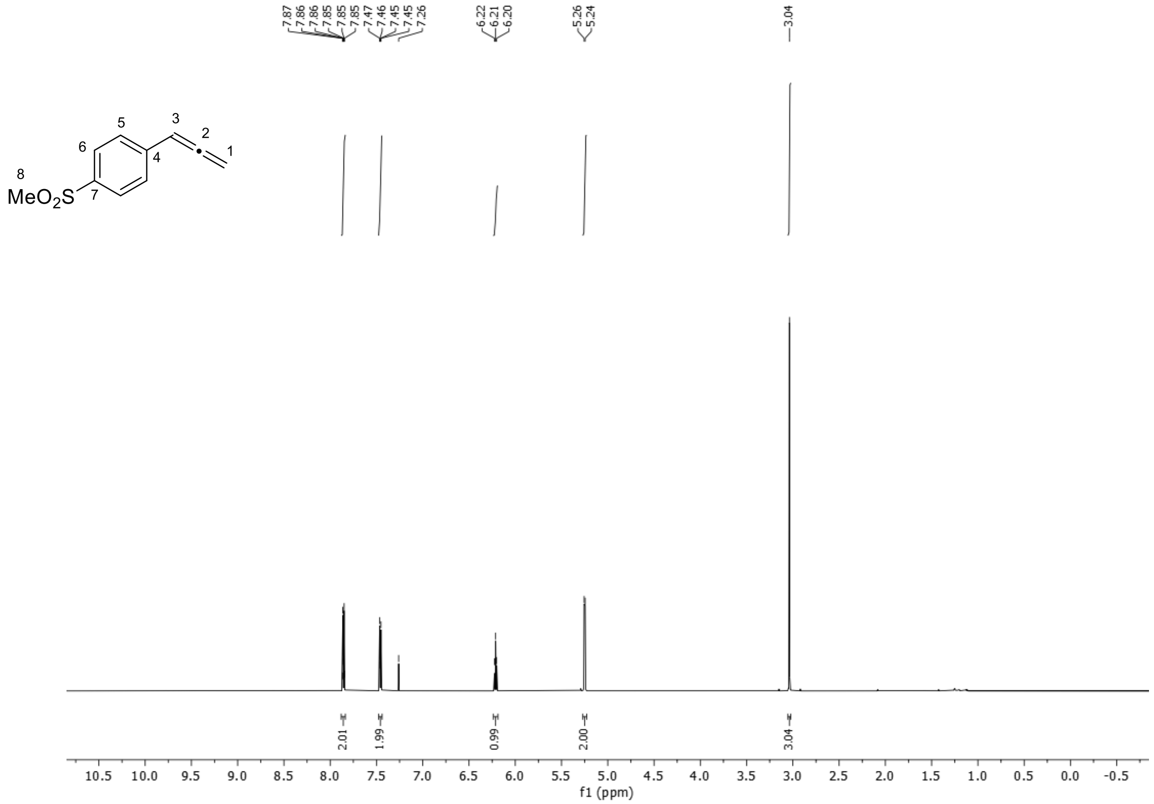

Supplementary Figure 29. ^{1}H NMR of **S21** (599 MHz, 299 K, $CDCl_3$).

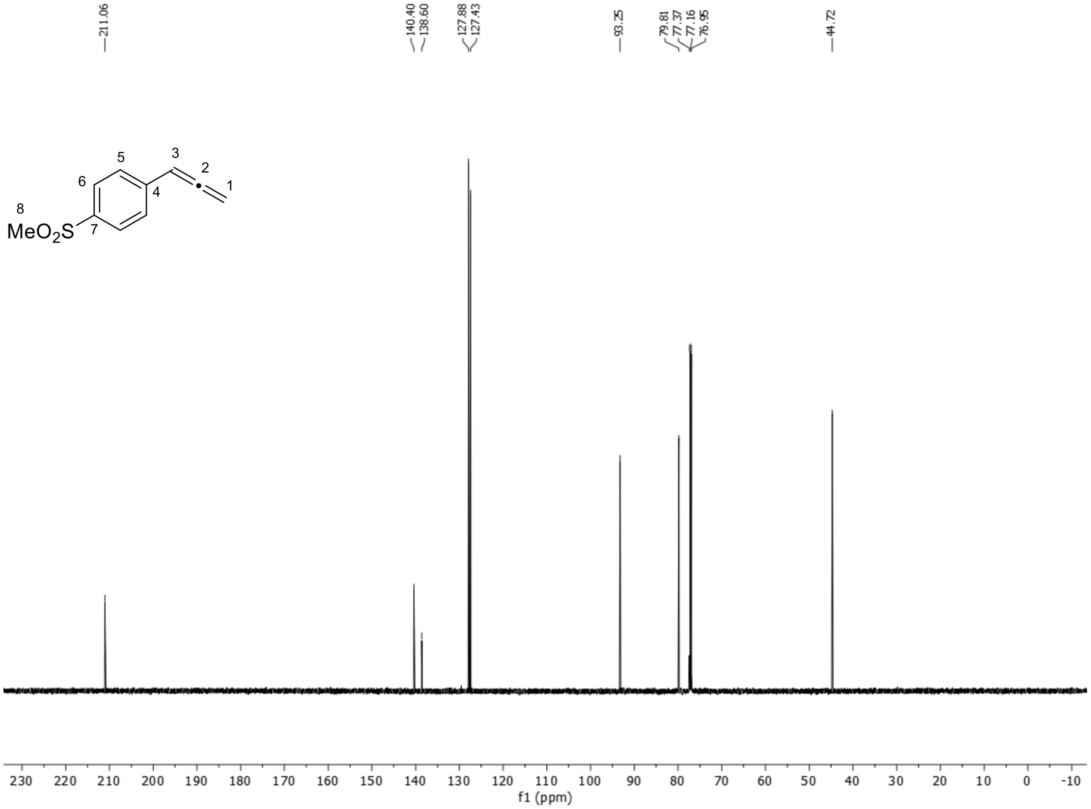

Supplementary Figure 30. $^{13}C\{^{1}H\}$ NMR of **S21** (151 MHz, 299 K, $CDCl_3$).

(1R,2S,5R)-2-Isopropyl-5-methylcyclohexyl 4-(propa-1,2-dien-1-yl)benzoate (S22)

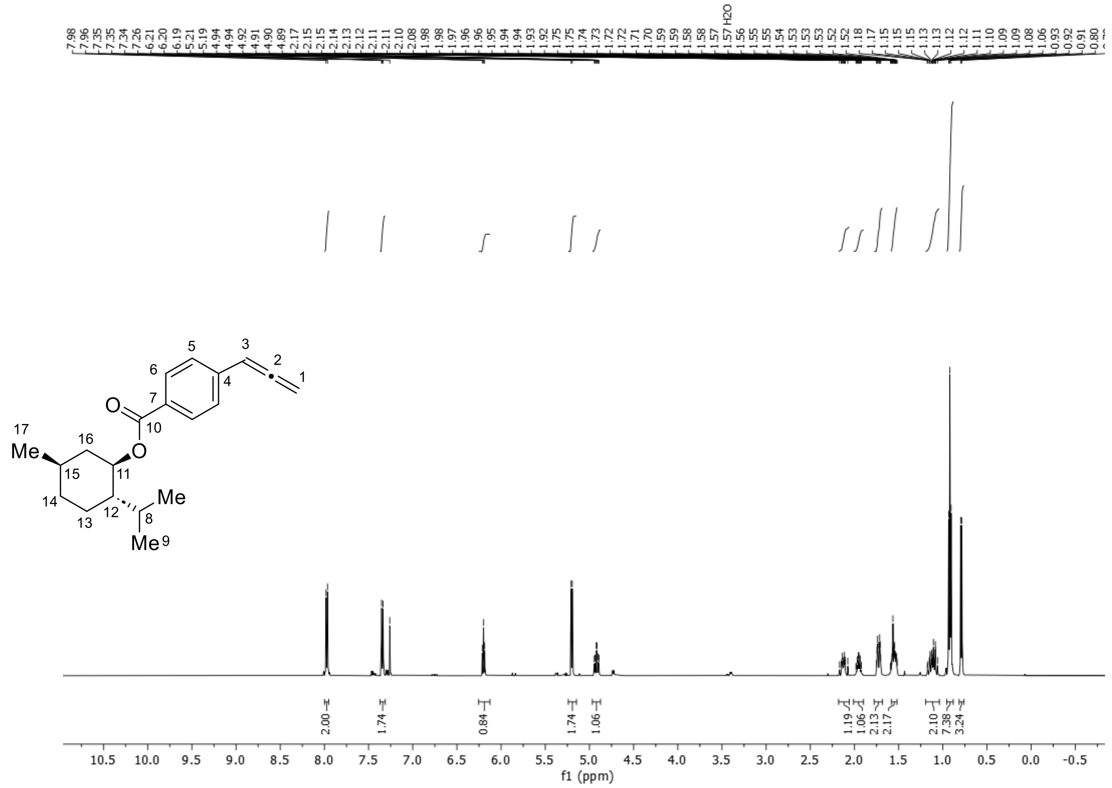

Supplementary Figure 31. ^{1}H NMR of **S22** (500 MHz, 299 K, $CDCl_3$).

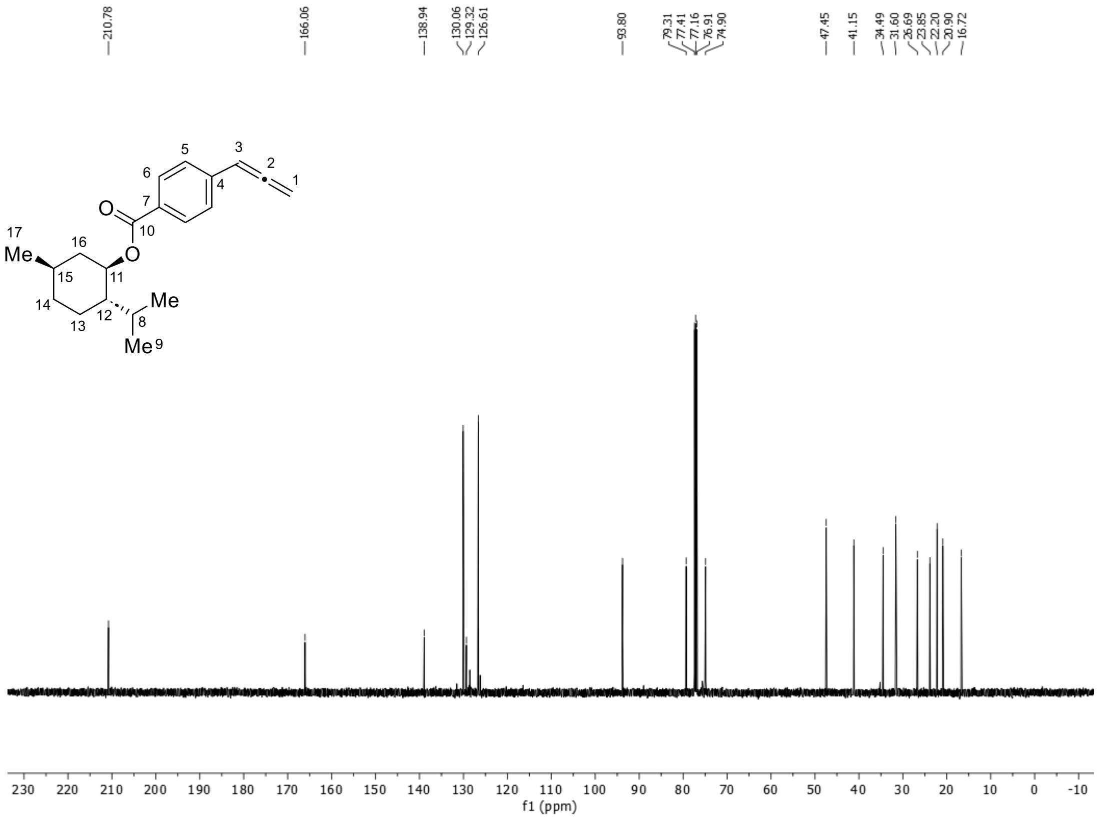

Supplementary Figure 32. $^{13}C\{^{1}H\}$ NMR of **S22** (126 MHz, 299 K, $CDCl_3$).

Methyl 4-(1-chlorobuta-2,3-dien-2-yl)benzoate (S23)

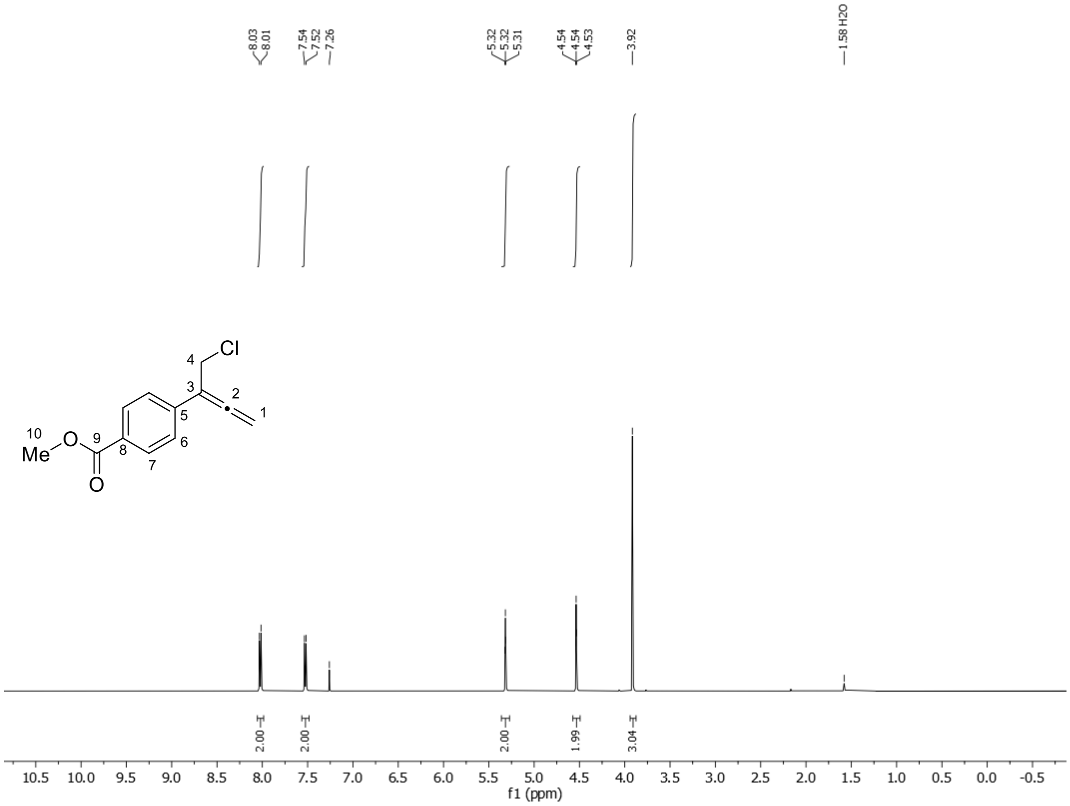

Supplementary Figure 33. ^{1}H NMR of **S23** (500 MHz, 299 K, $CDCl_3$).

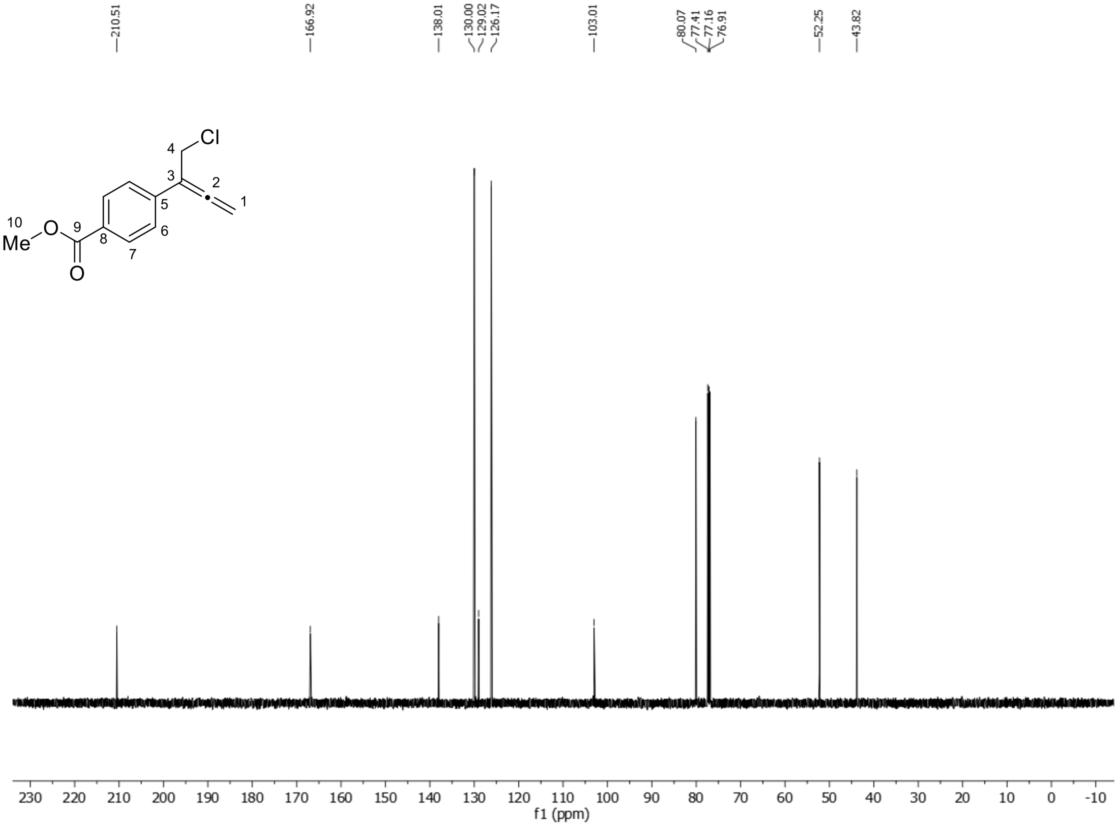

Supplementary Figure 34. ^{13}C{^{1}H} NMR of **S23** (126 MHz, 299 K, $CDCl_3$).

1-(1-Chlorobuta-2,3-dien-2-yl)-4-(trifluoromethyl)benzene (S24)

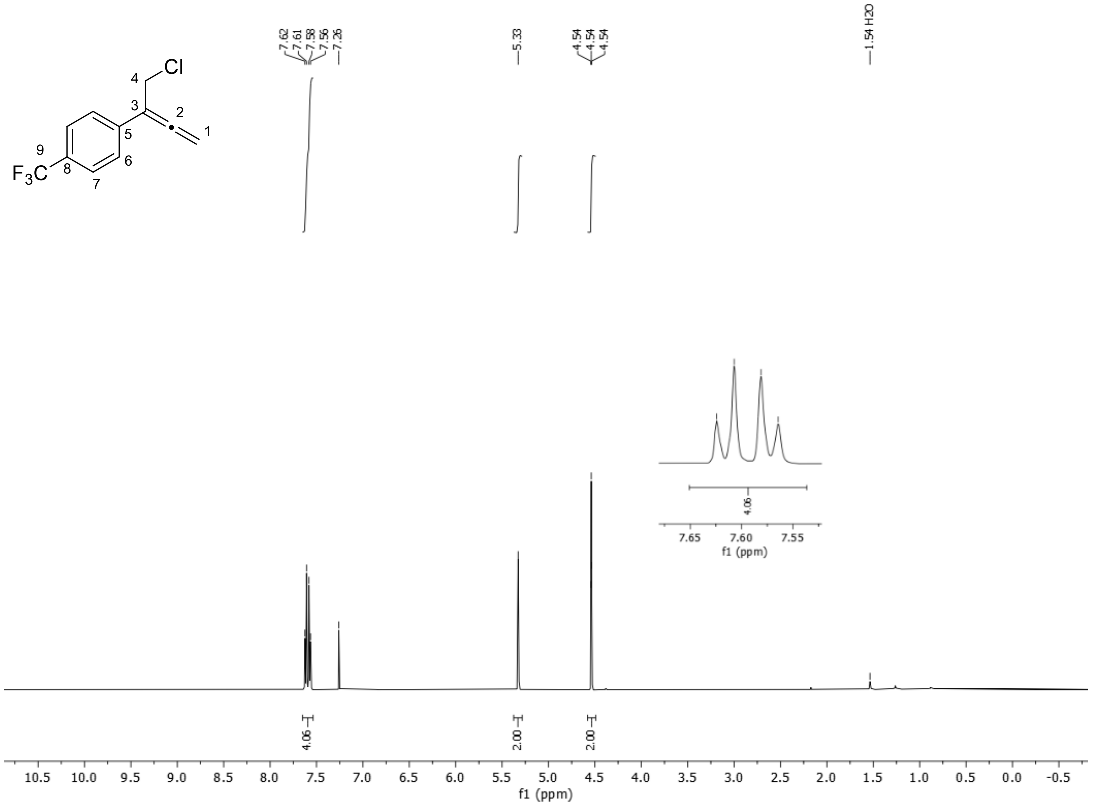

Supplementary Figure 35. ^{1}H NMR of **S24** (500 MHz, 299 K, $CDCl_3$).

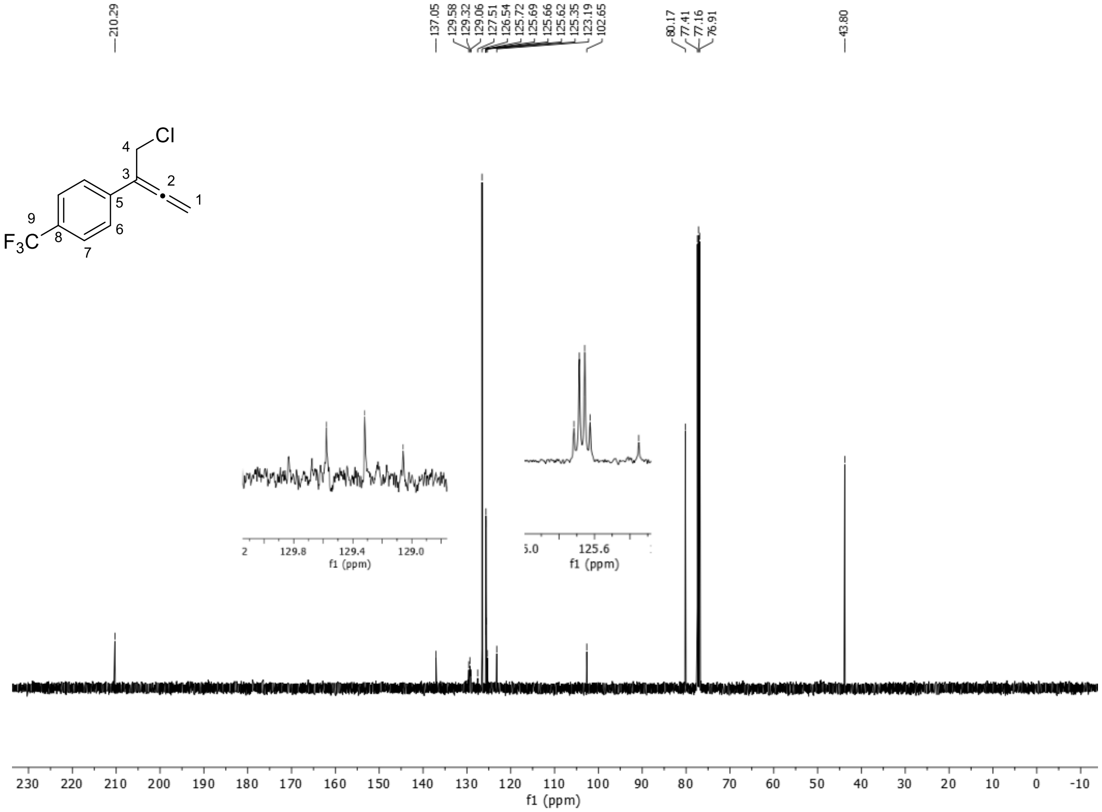

Supplementary Figure 36. $^{13}C\{^{1}H\}$ NMR of **S24** (126 MHz, 299 K, $CDCl_3$).

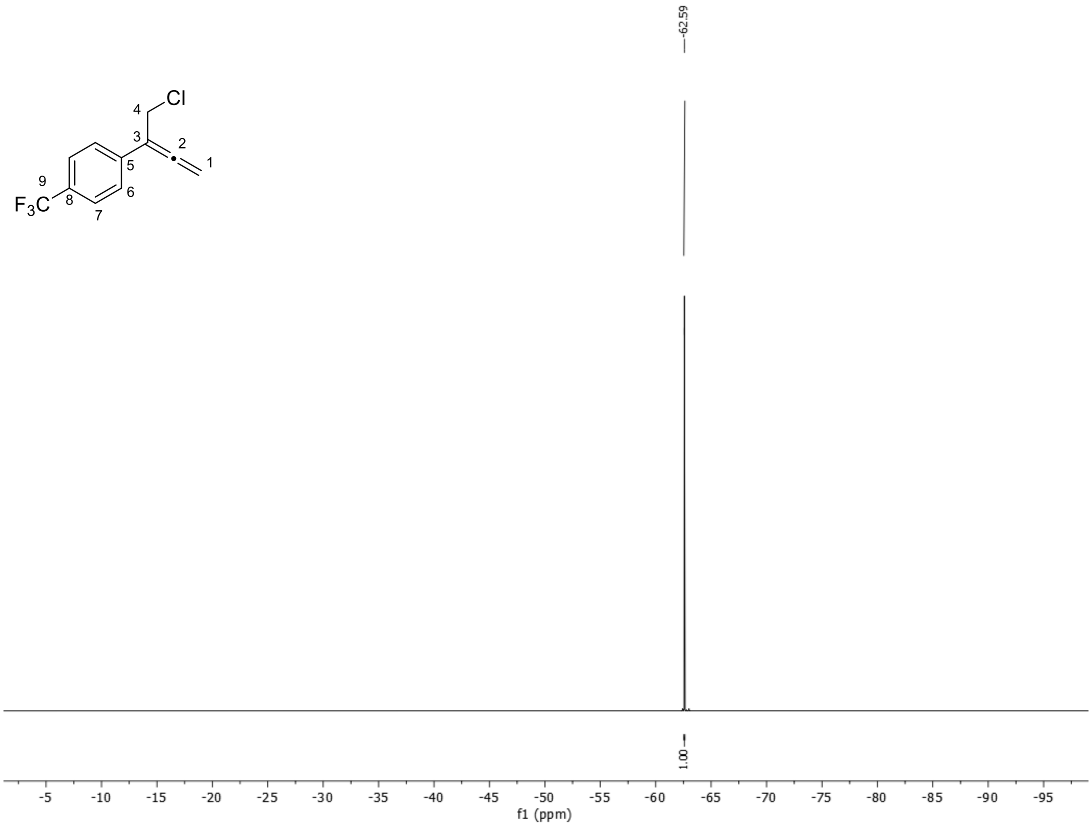

Supplementary Figure 37. ^{19}F NMR of **S24** (470 MHz, 299 K, $CDCl_3$).

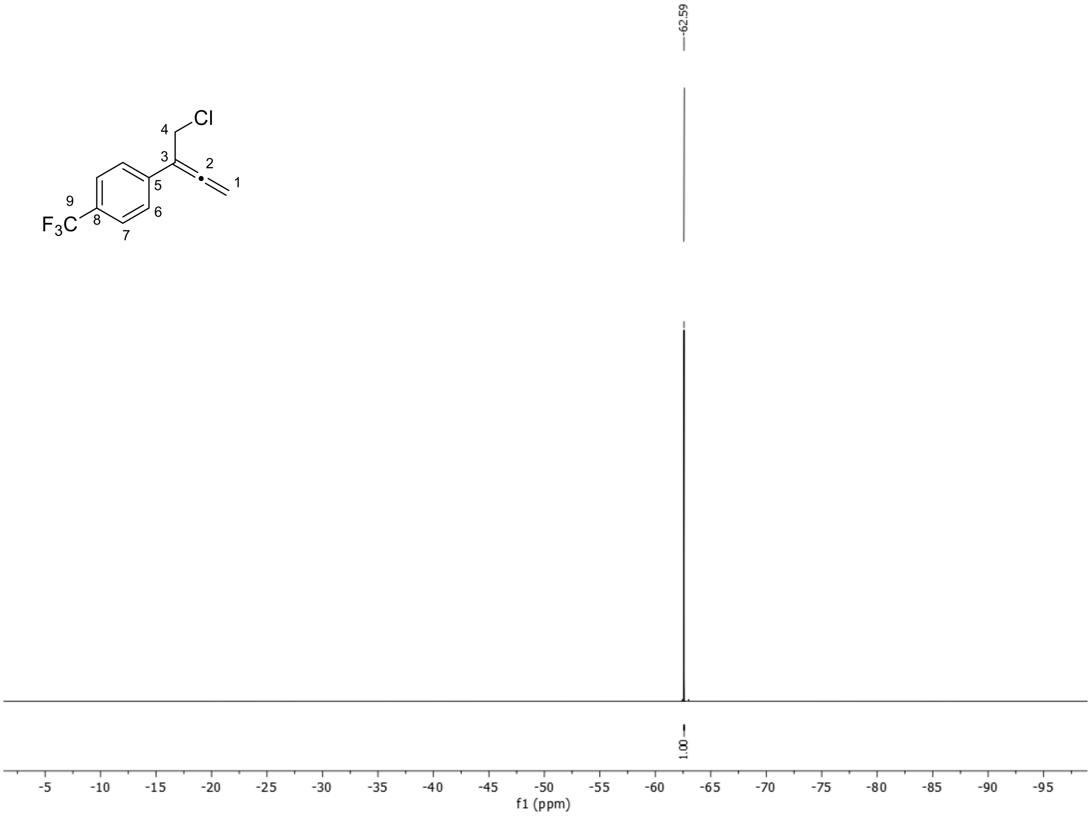

Supplementary Figure 38. ^{19}F{^{1}H} NMR of **S24** (470 MHz, 299 K, $CDCl_3$).

(2-(Chloromethyl)buta-2,3-dien-1-yl)benzene (S25)

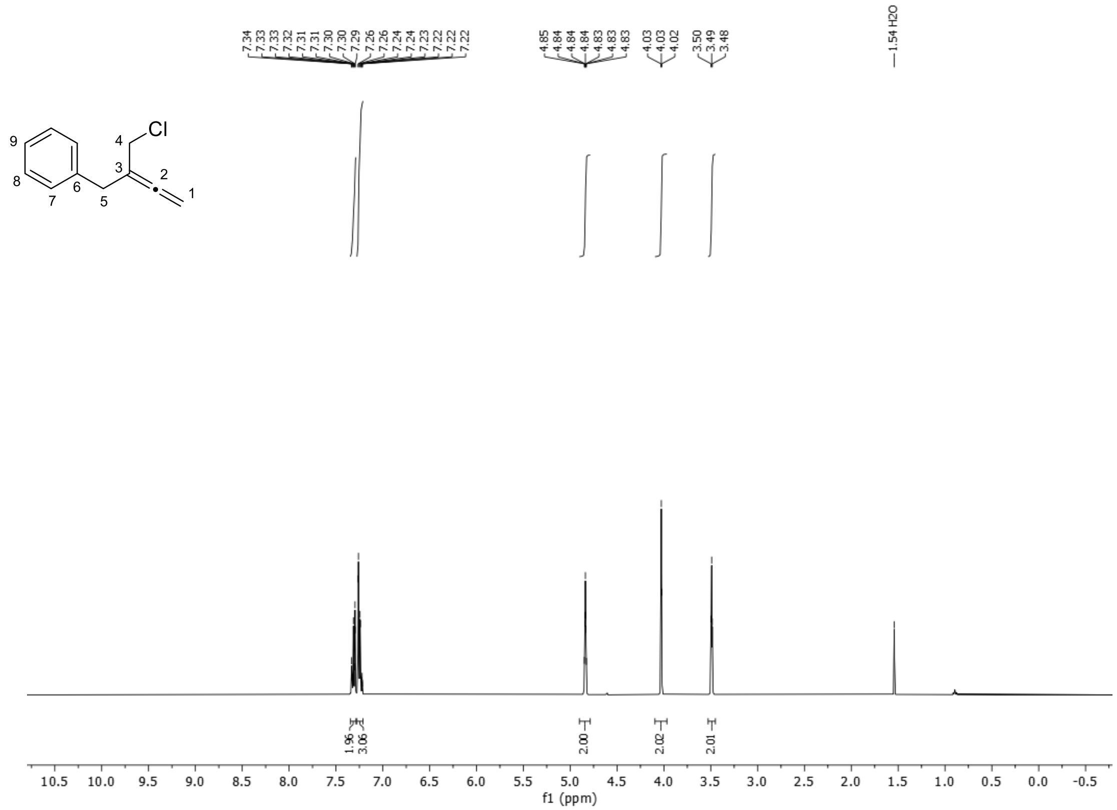

Supplementary Figure 39. ^{1}H NMR of **S25** (400 MHz, 299 K, $CDCl_3$).

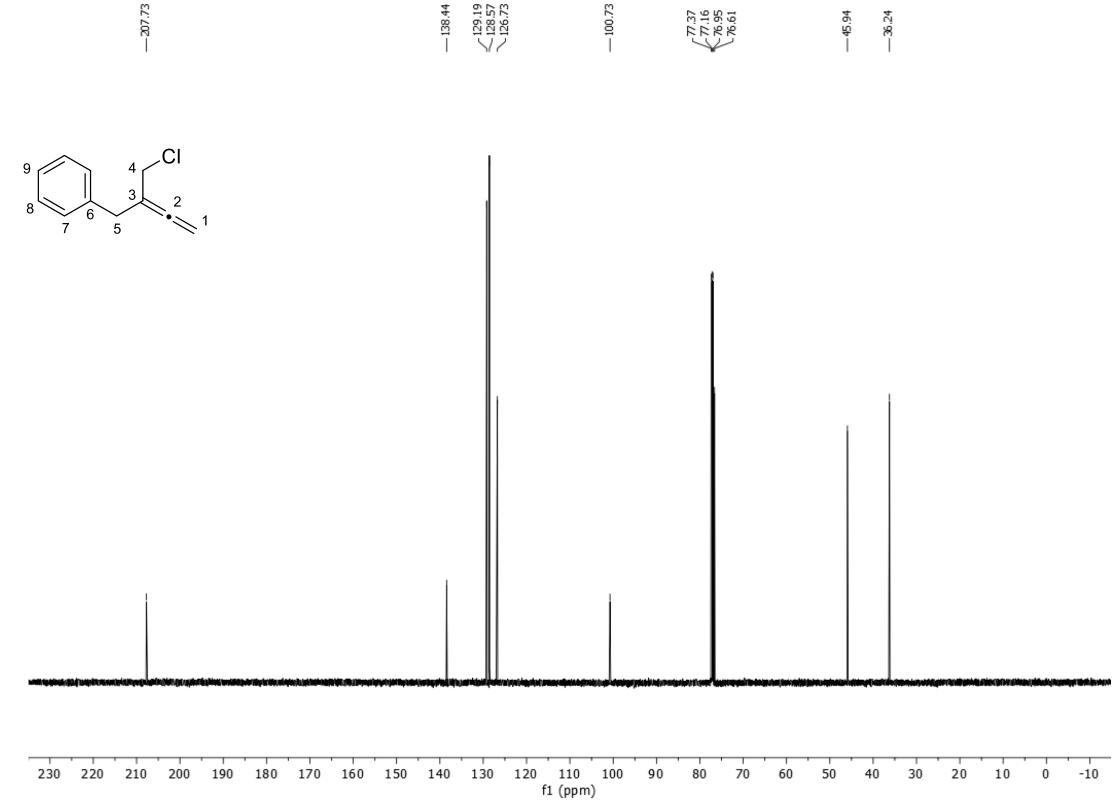

Supplementary Figure 40. $^{13}C\{^{1}H\}$ NMR of **S25** (151 MHz, 299 K, $CDCl_3$).

2-Vinylidenetetradecan-1-ol (Int 7)

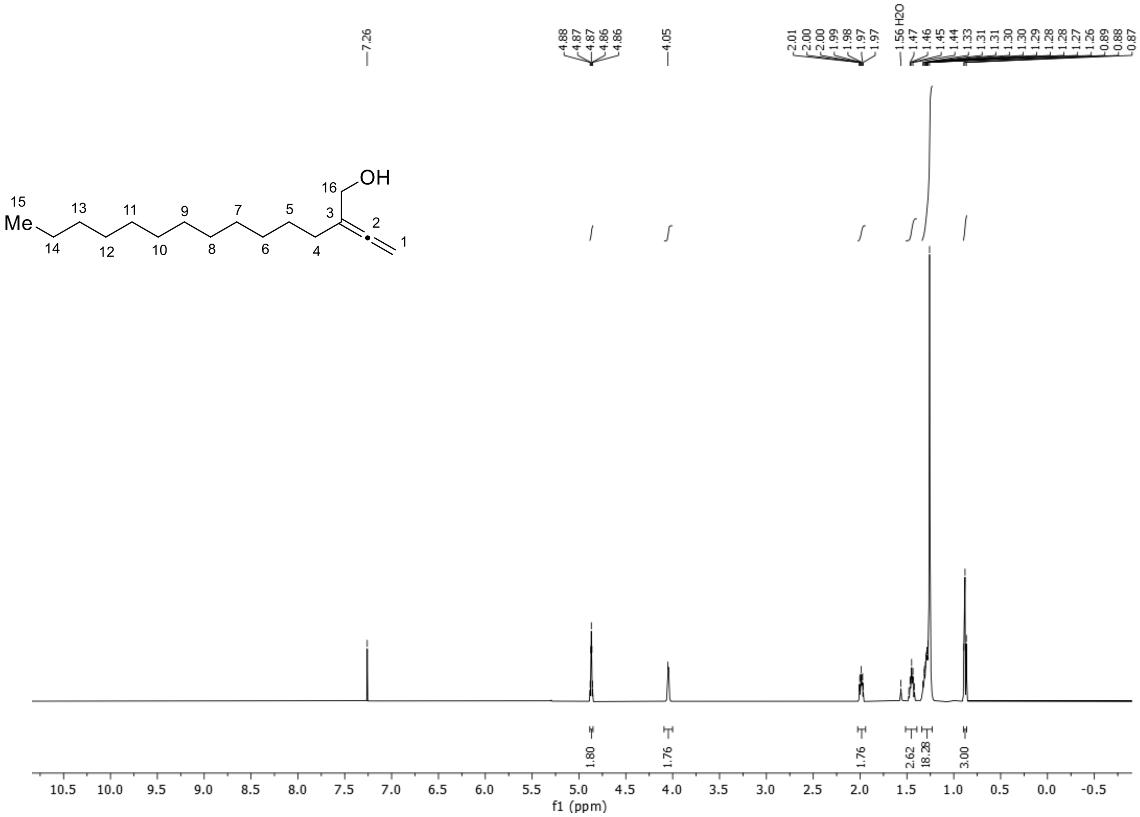

Supplementary Figure 41. ^{1}H NMR of **Int 7** (500 MHz, 299 K, $CDCl_3$).

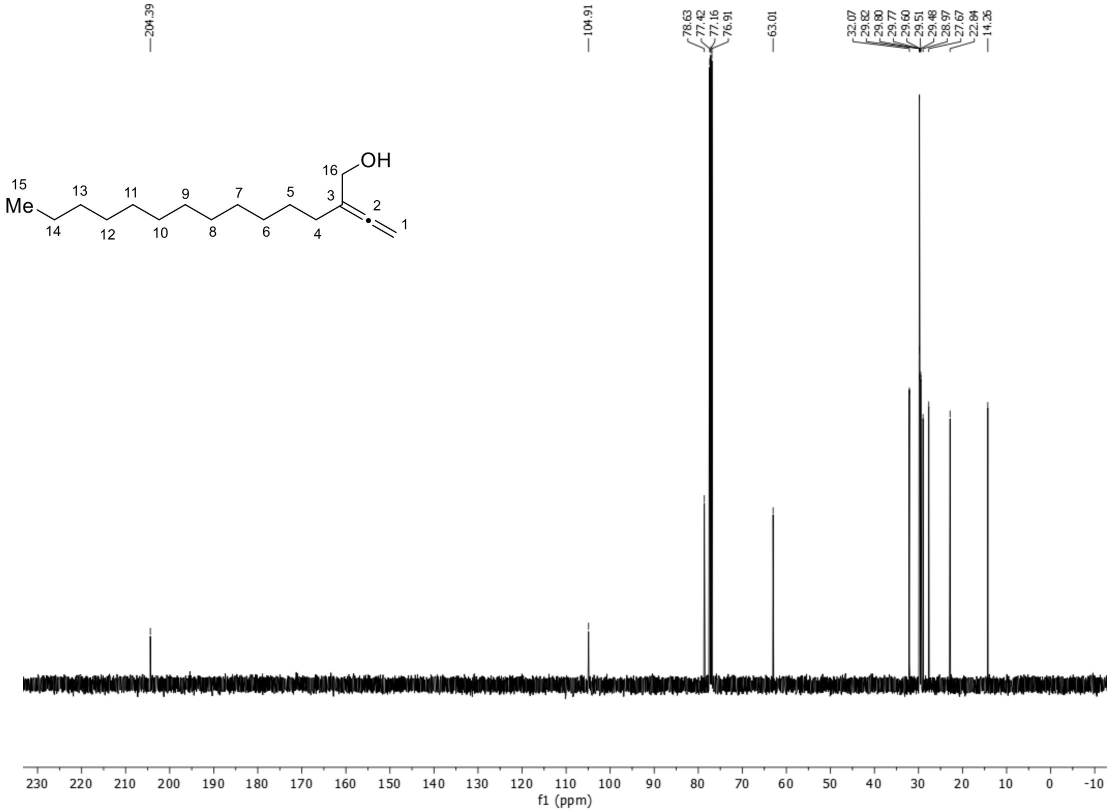

Supplementary Figure 42. $^{13}C\{^{1}H\}$ NMR of **Int 7** (126 MHz, 299 K, $CDCl_3$).

3-(Chloromethyl)pentadeca-1,2-diene (S26)

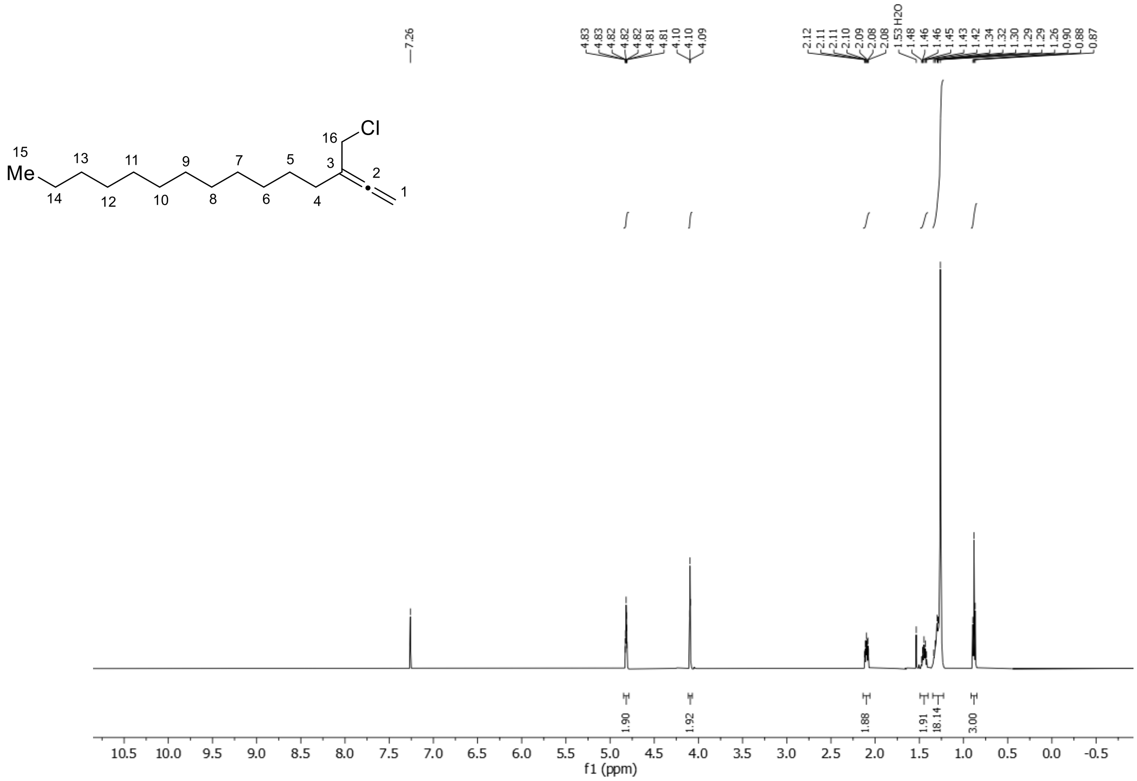

Supplementary Figure 43. ^{1}H NMR of **S26** (500 MHz, 299 K, $CDCl_3$).

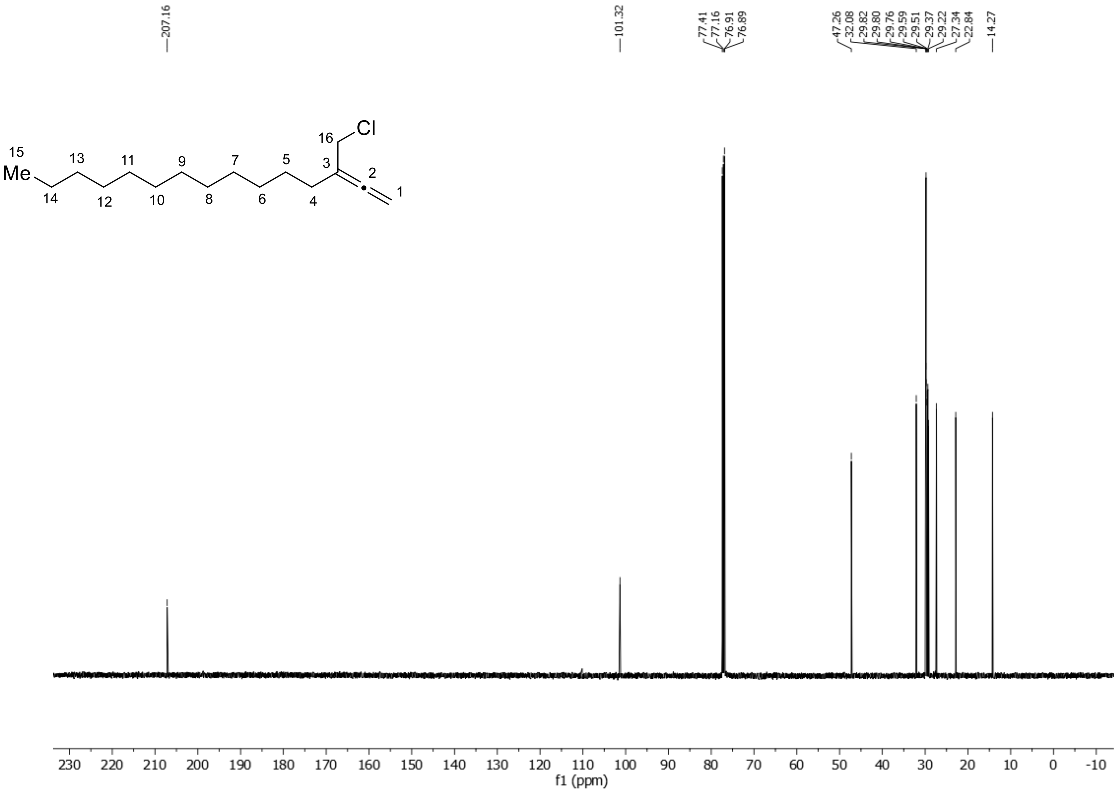

Supplementary Figure 44. $^{13}C\{^{1}H\}$ NMR of **S26** (126 MHz, 299 K, $CDCl_3$).

3-(Fluoromethyl)pentadeca-1,2-diene (S27)

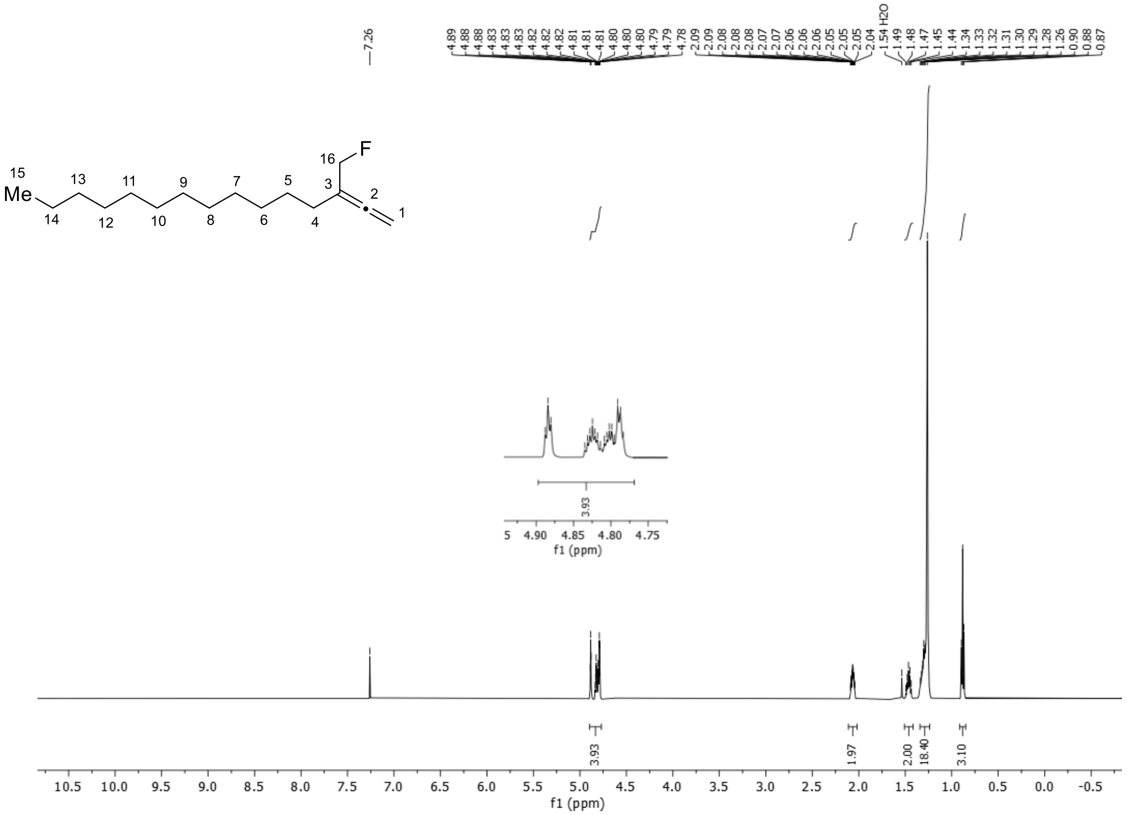

Supplementary Figure 45. ^{1}H NMR of **S27** (500 MHz, 299 K, $CDCl_3$).

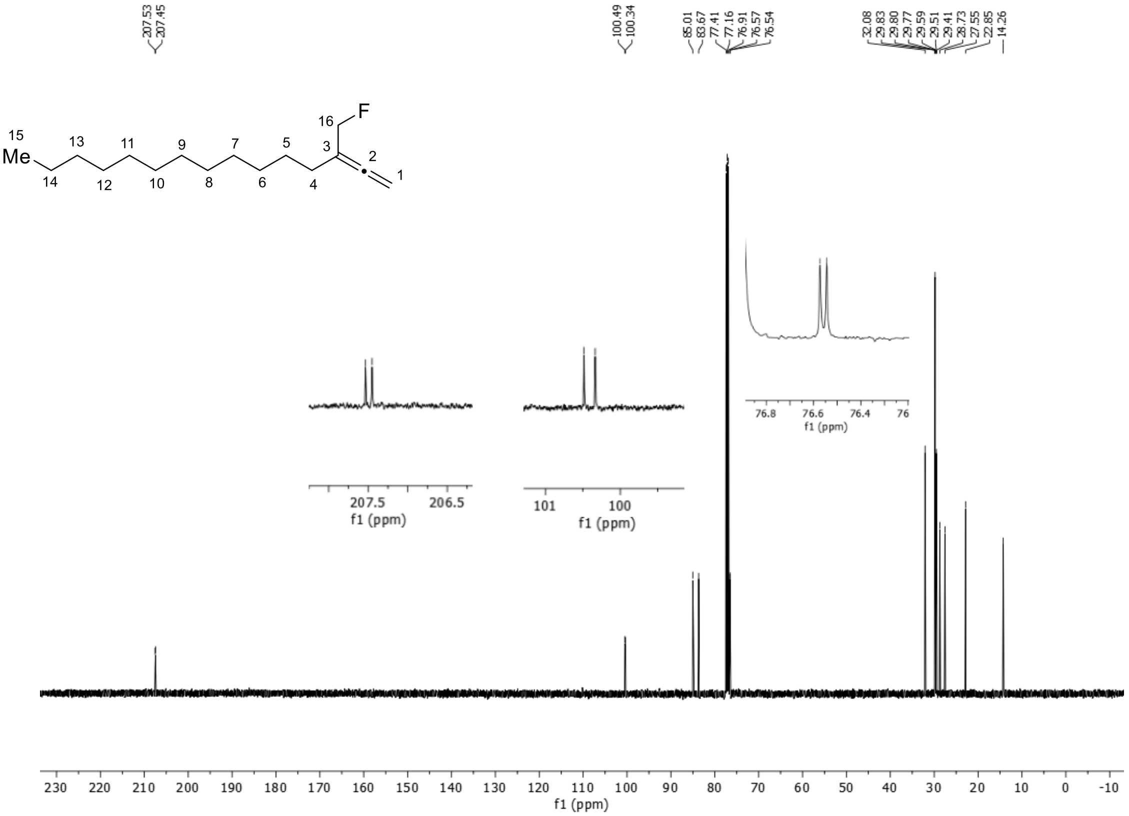

Supplementary Figure 46. $^{13}C\{^{1}H\}$ NMR of **S27** (126 MHz, 299 K, $CDCl_3$).

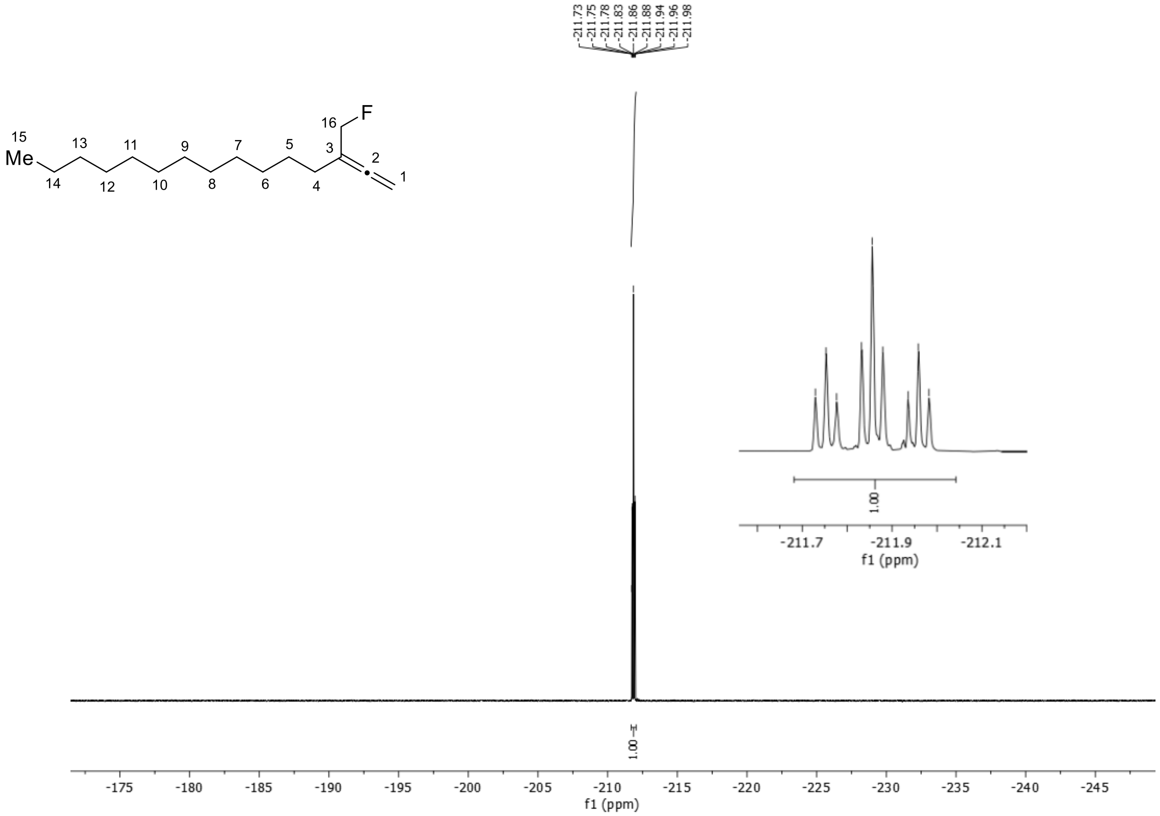

Supplementary Figure 47. ^{19}F NMR of **S27** (470 MHz, 299 K, $CDCl_3$).

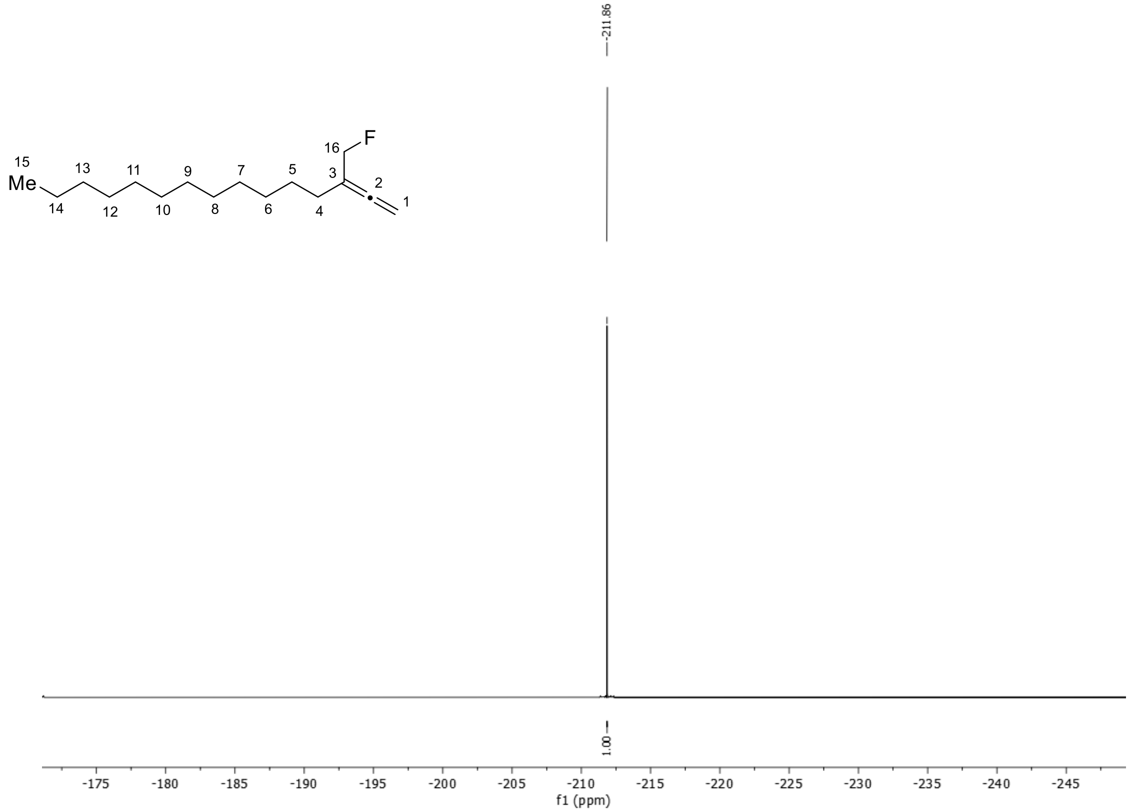

Supplementary Figure 48. ^{19}F{^{1}H} NMR of **S27** (470 MHz, 299 K, $CDCl_3$).

3-(Ethoxymethyl)pentadeca-1,2-diene (S28)

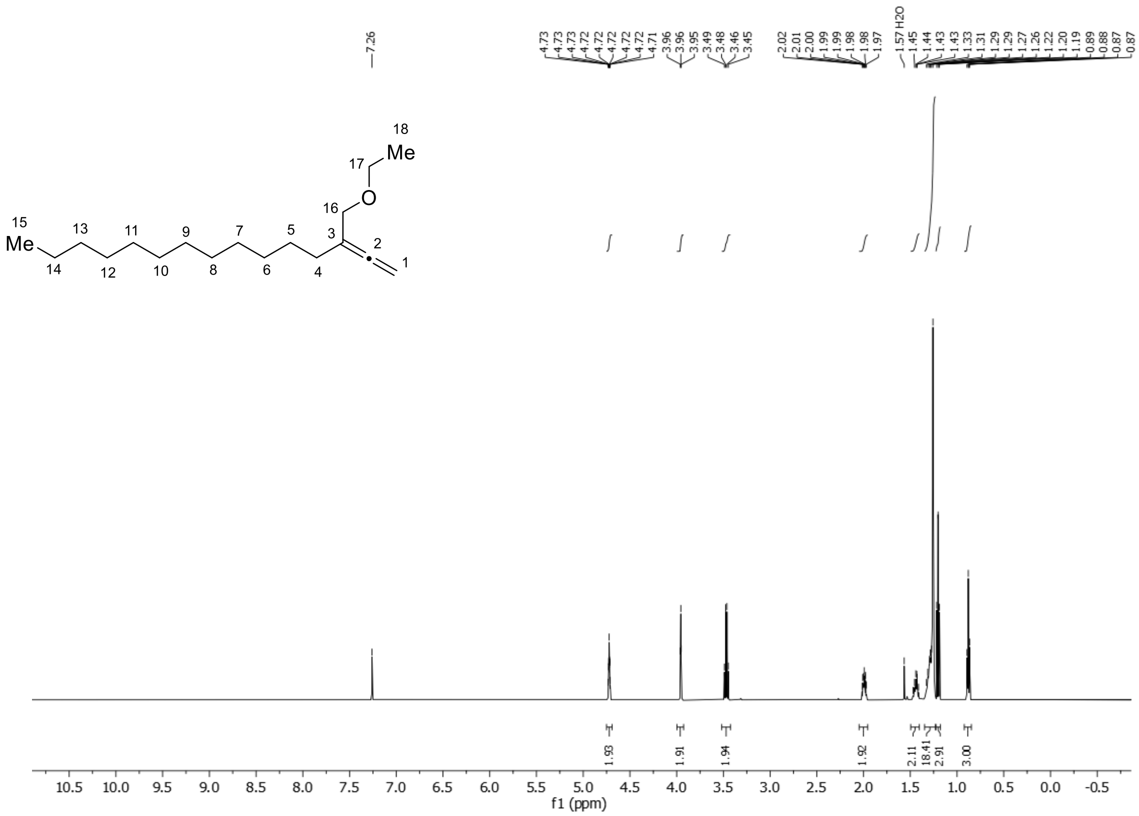

Supplementary Figure 49. ^{1}H NMR of **S28** (500 MHz, 299 K, $CDCl_3$).

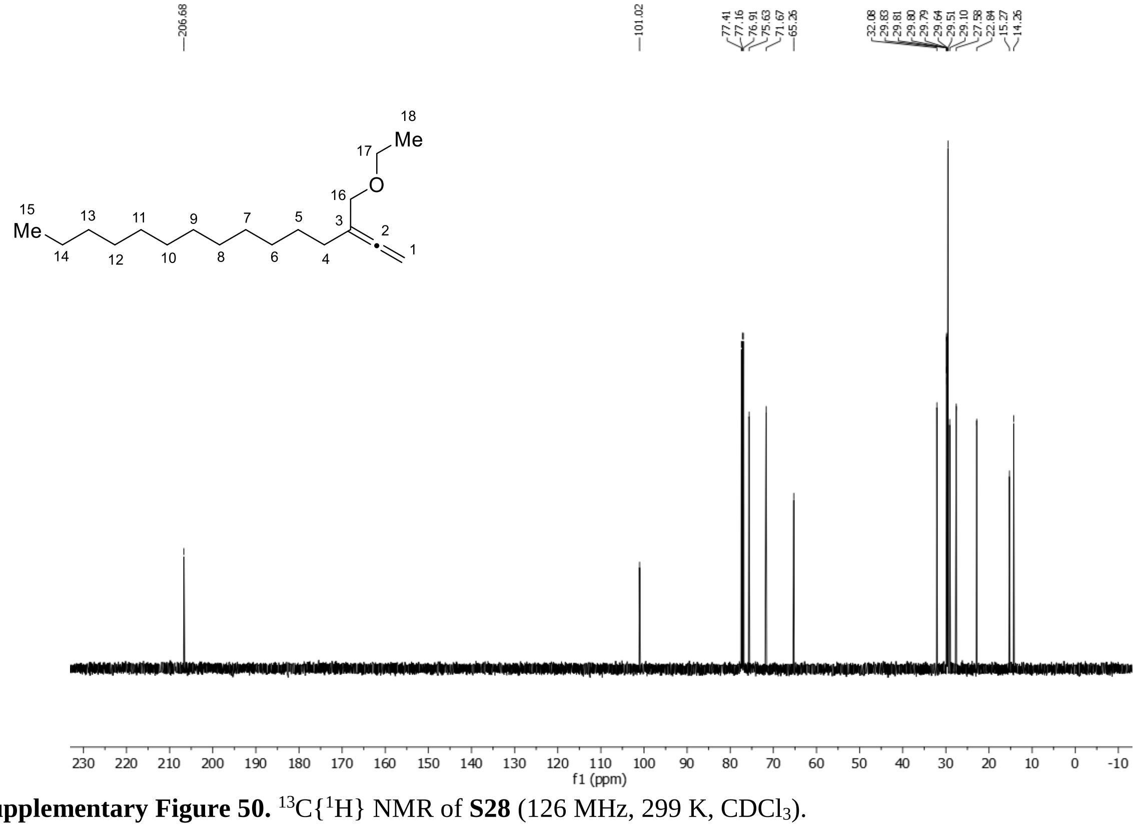

Supplementary Figure 50. $^{13}C\{^{1}H\}$ NMR of **S28** (126 MHz, 299 K, $CDCl_3$).

N,4-dimethyl-N-(2-vinylidenetetradecyl)benzenesulfonamide (S29)

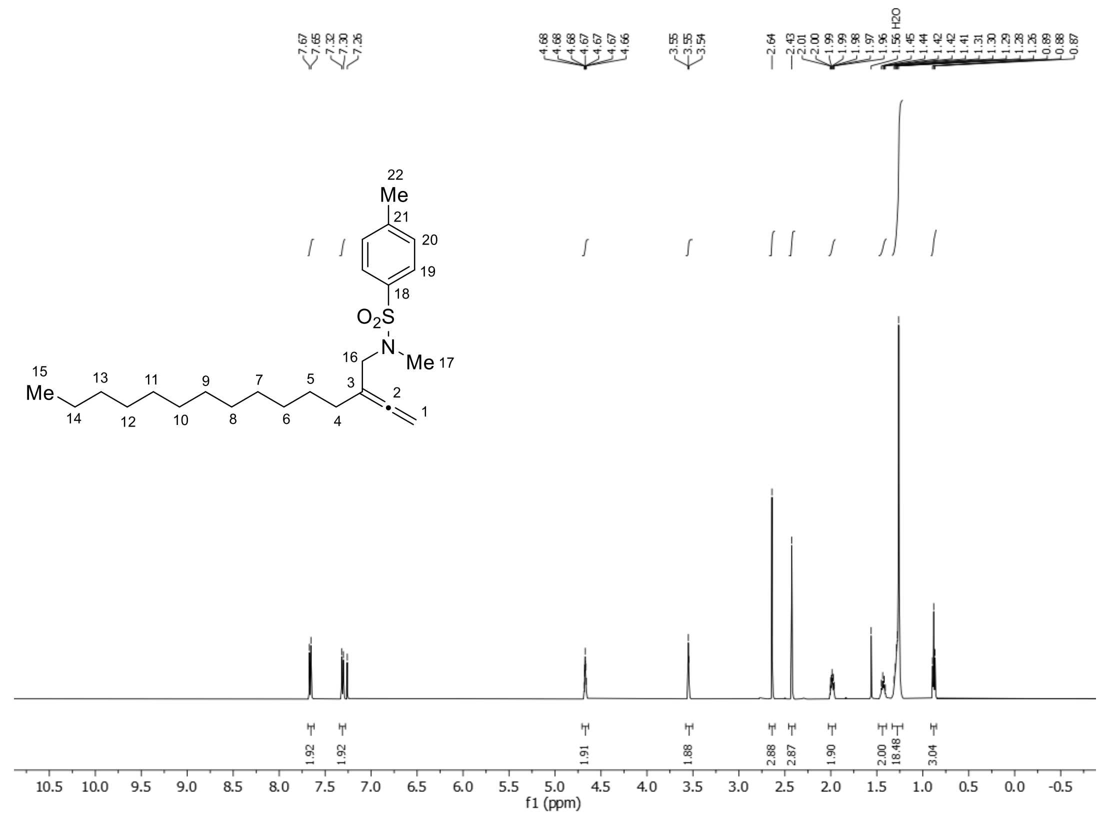

Supplementary Figure 51. ^{1}H NMR of **S29** (500 MHz, 299 K, $CDCl_3$).

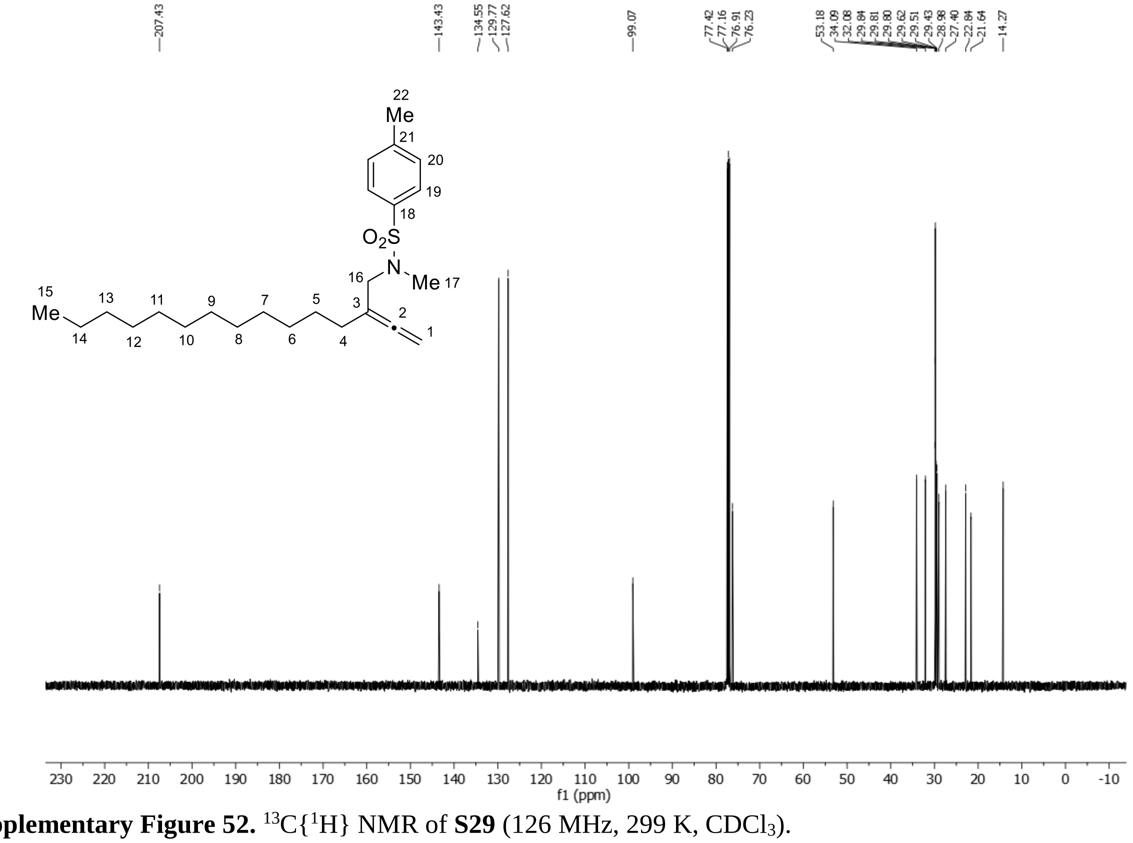

Supplementary Figure 52. $^{13}C\{^{1}H\}$ NMR of **S29** (126 MHz, 299 K, $CDCl_3$).

4-Vinylidenehexadecane (S30)

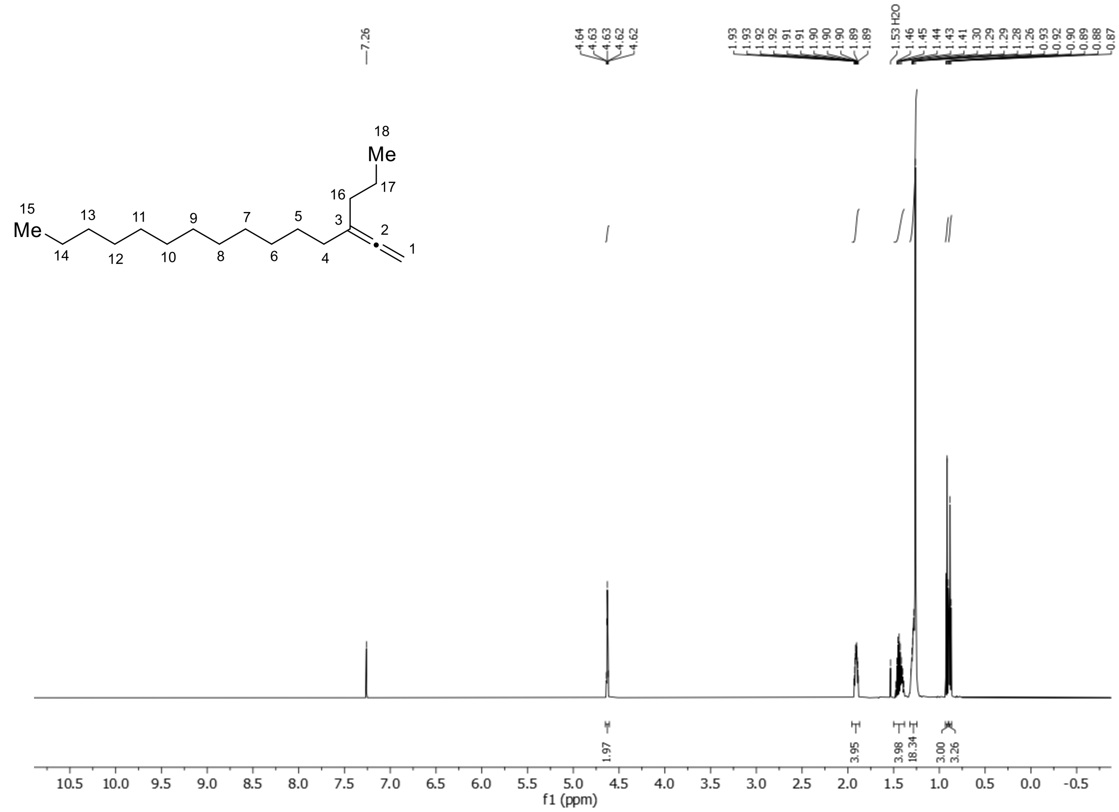

Supplementary Figure 53. ^{1}H NMR of **S30** (599 MHz, 299 K, $CDCl_3$).

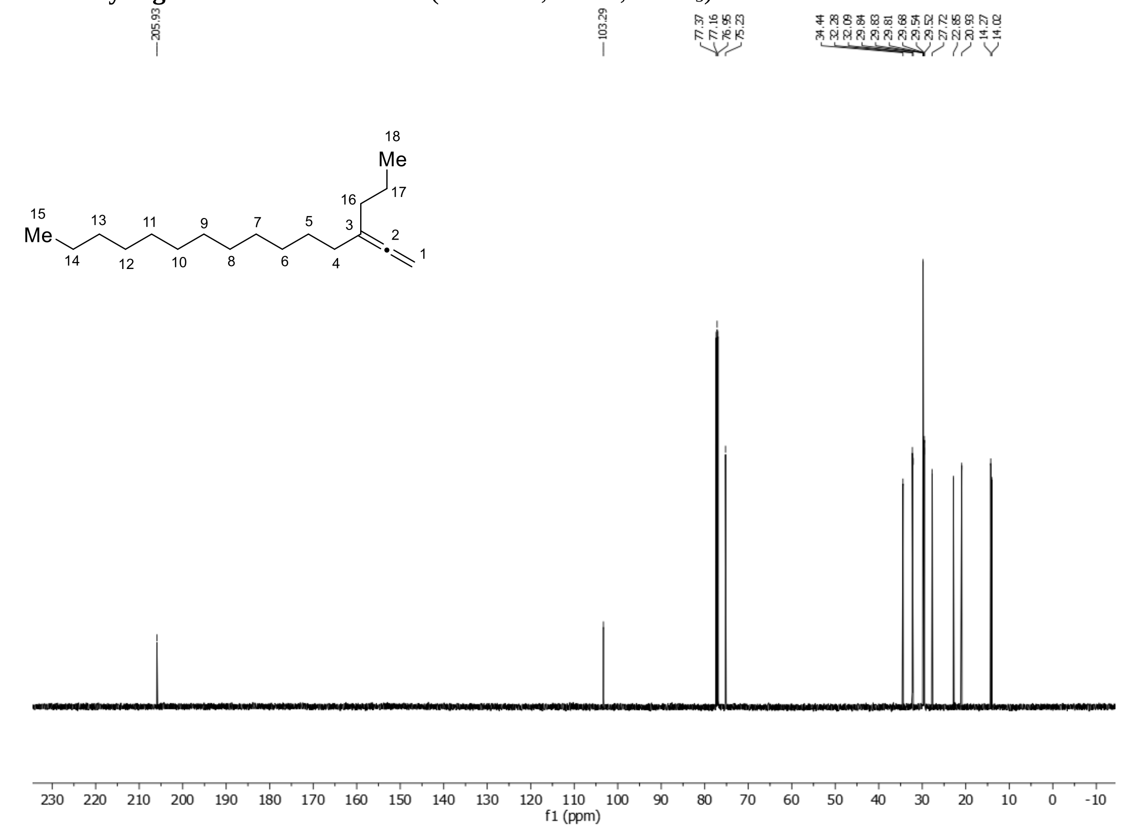

Supplementary Figure 54. $^{13}C\{^{1}H\}$ NMR of **S30** (151 MHz, 299 K, $CDCl_3$).

(1S,2R,5S)-2-Isopropyl-5-methylcyclohexyl 4-(1-chlorobuta-2,3-dien-2-yl)benzoate (S31)

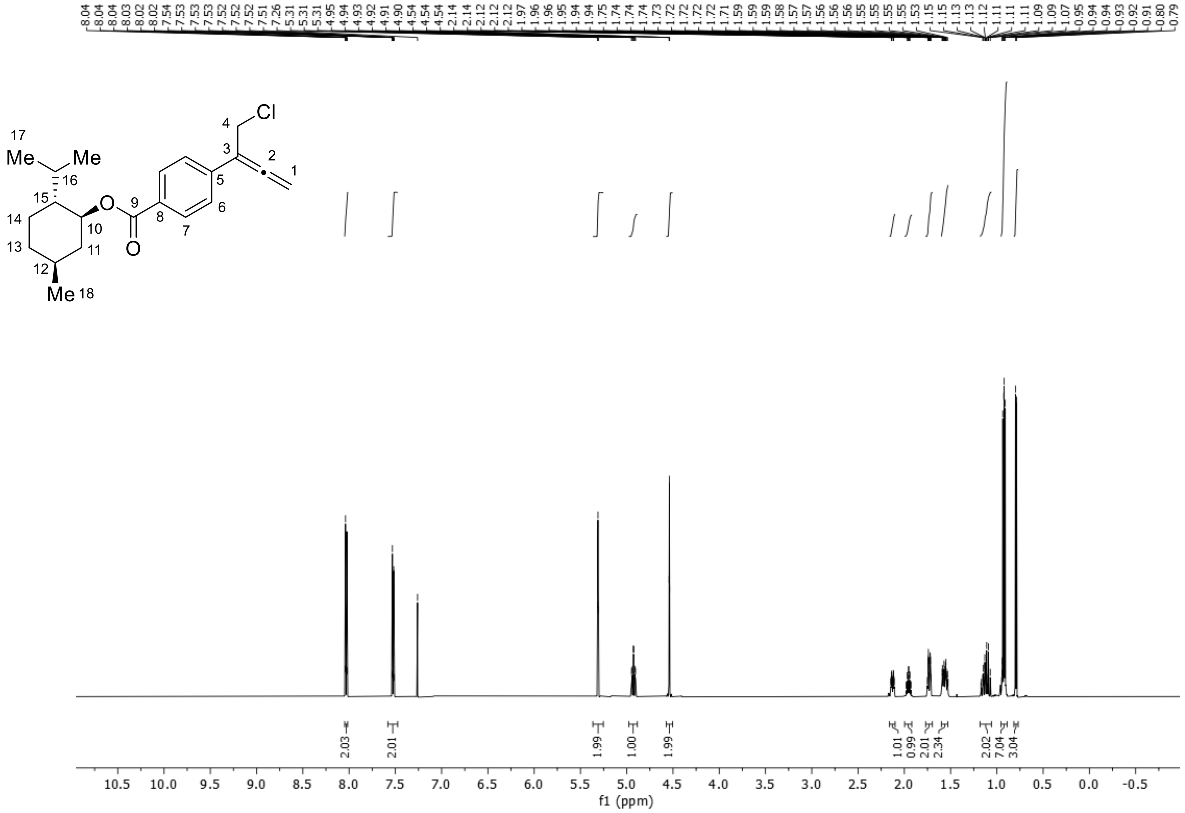

Supplementary Figure 55. ^{1}H NMR of **S31** (599 MHz, 299 K, $CDCl_3$).

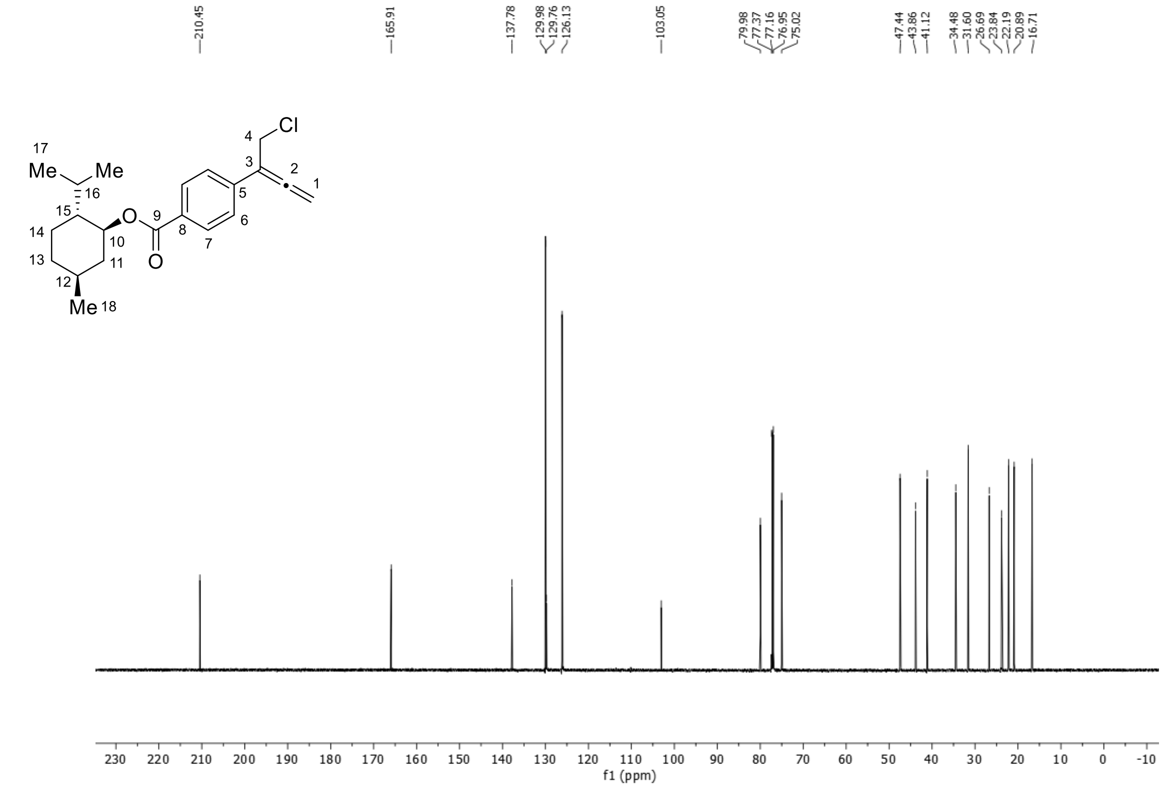

Supplementary Figure 56. $^{13}C\{^{1}H\}$ NMR of **S31** (151 MHz, 299 K, $CDCl_3$).

3-Fluoropentadec-1-yne (1)

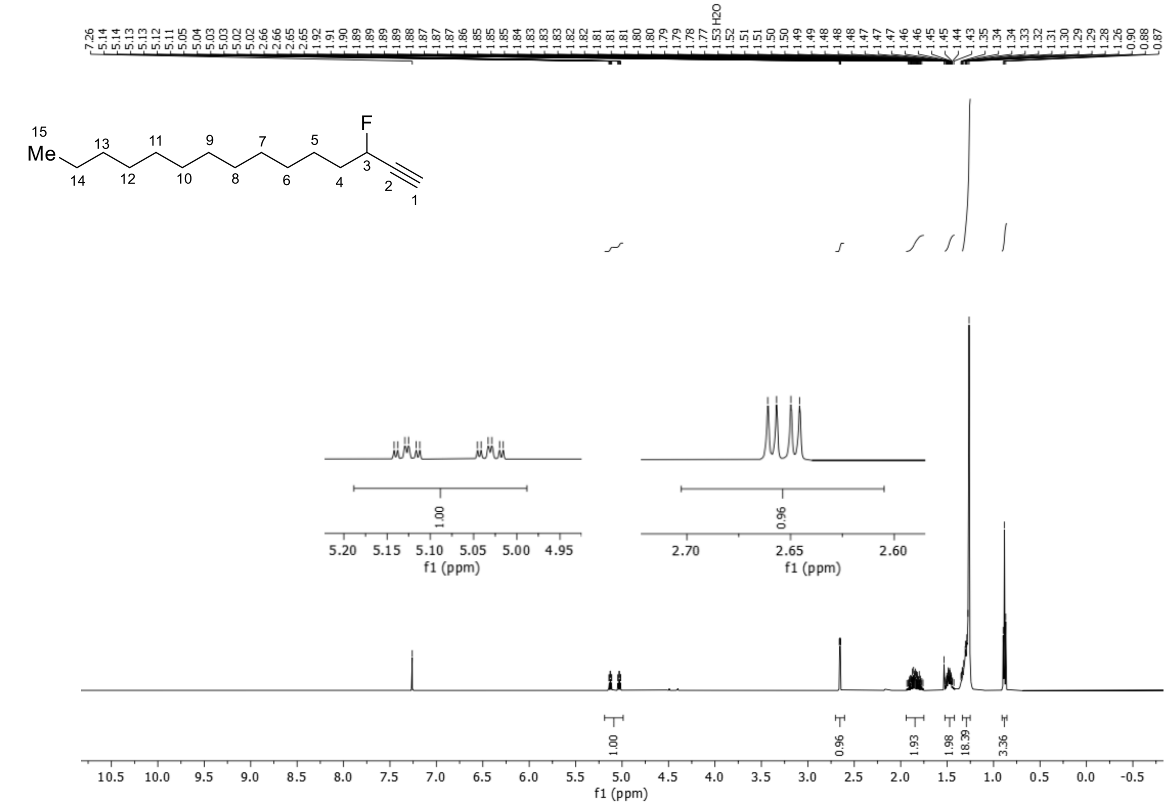

Supplementary Figure 57. ^{1}H NMR of **1** (500 MHz, 299 K, $CDCl_3$).

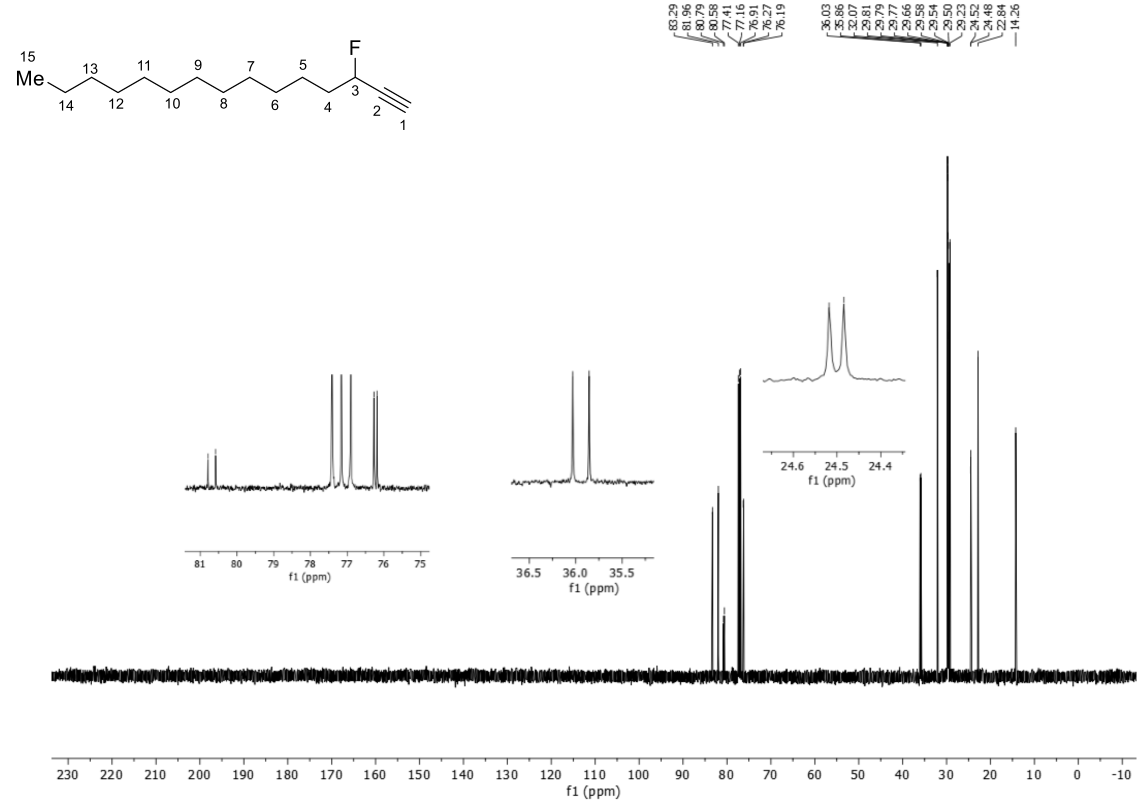

Supplementary Figure 58. $^{13}C\{^{1}H\}$ NMR of **1** (126 MHz, 299 K, $CDCl_3$).

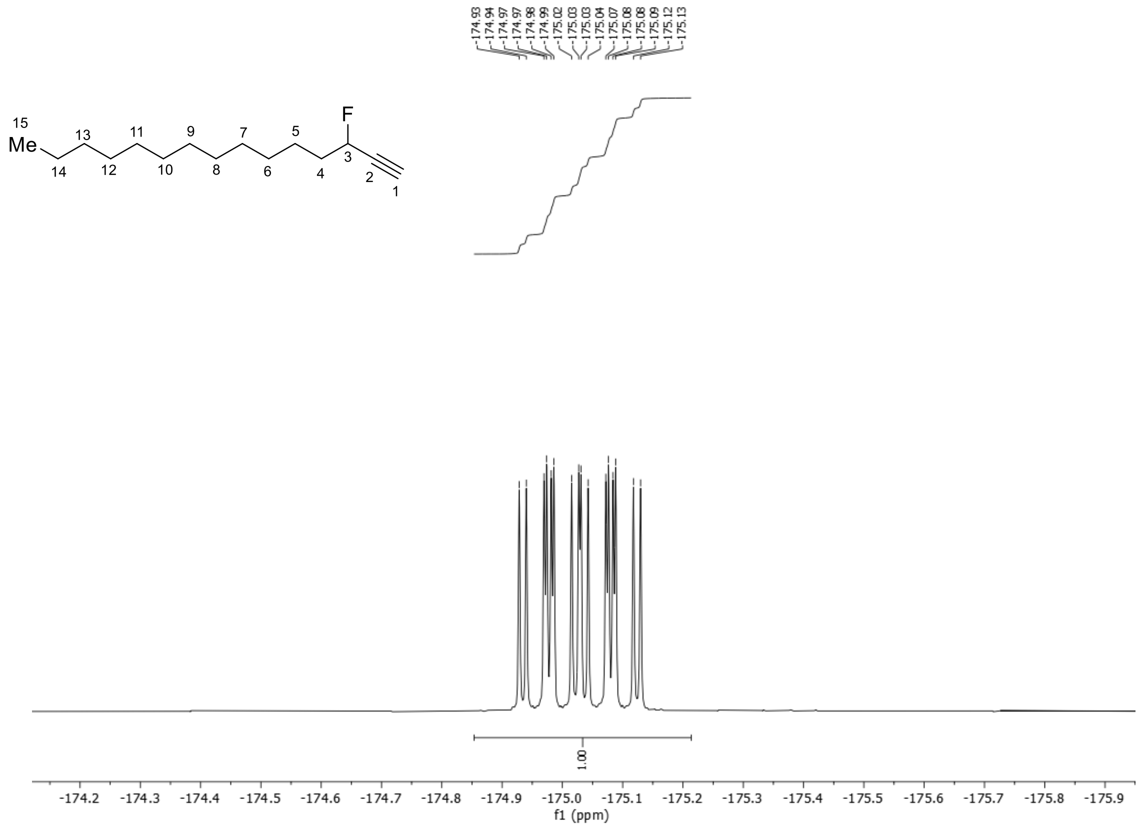

Supplementary Figure 59. ^{19}F NMR of **1** (470 MHz, 299 K, $CDCl_3$).

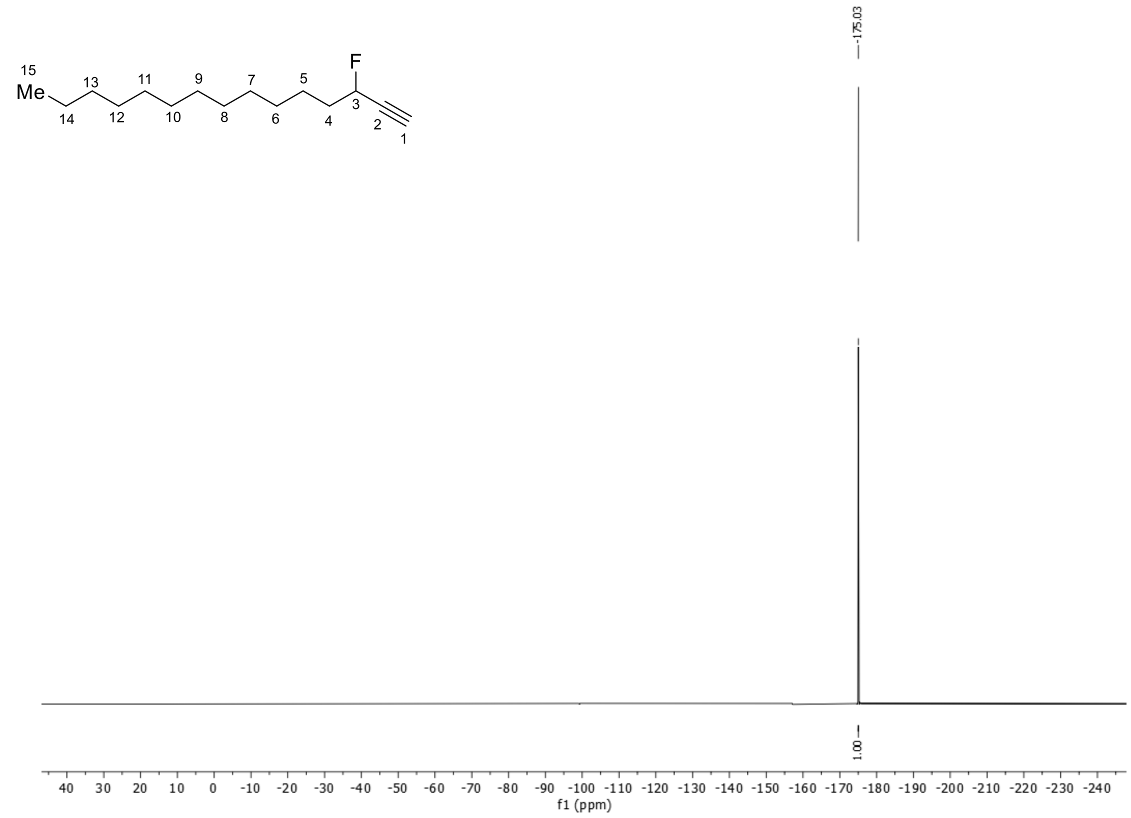

Supplementary Figure 60. $^{19}F\{^{1}H\}$ NMR of **1** (377 MHz, 299 K, $CDCl_3$).

(2-Fluorobut-3-yn-1-yl)cyclohexane (2)

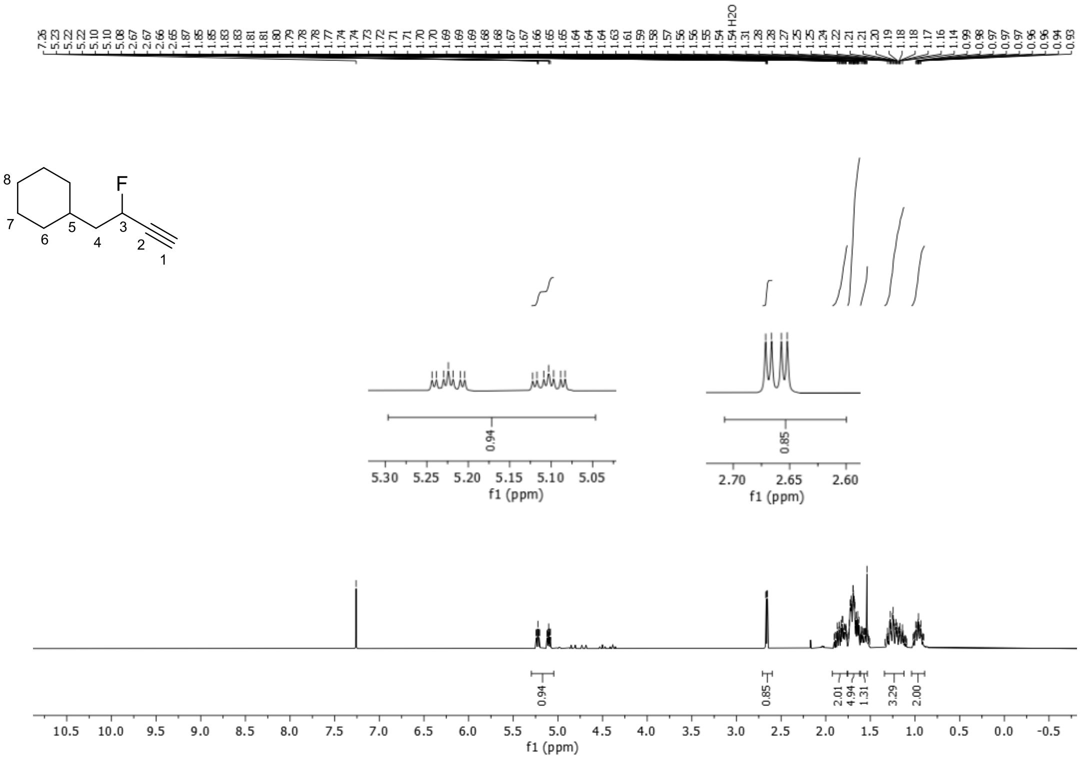

Supplementary Figure 61. ^{1}H NMR of **2** (400 MHz, 299 K, $CDCl_3$).

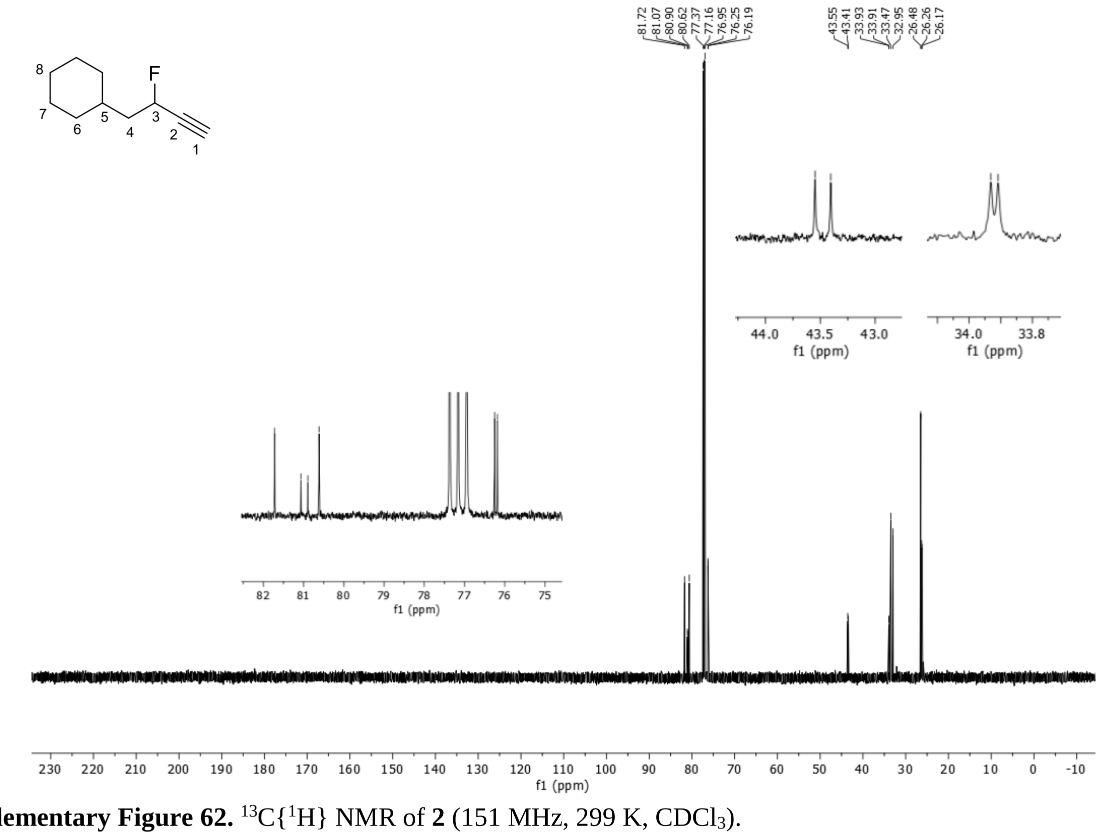

Supplementary Figure 62. $^{13}C\{^{1}H\}$ NMR of **2** (151 MHz, 299 K, $CDCl_3$).

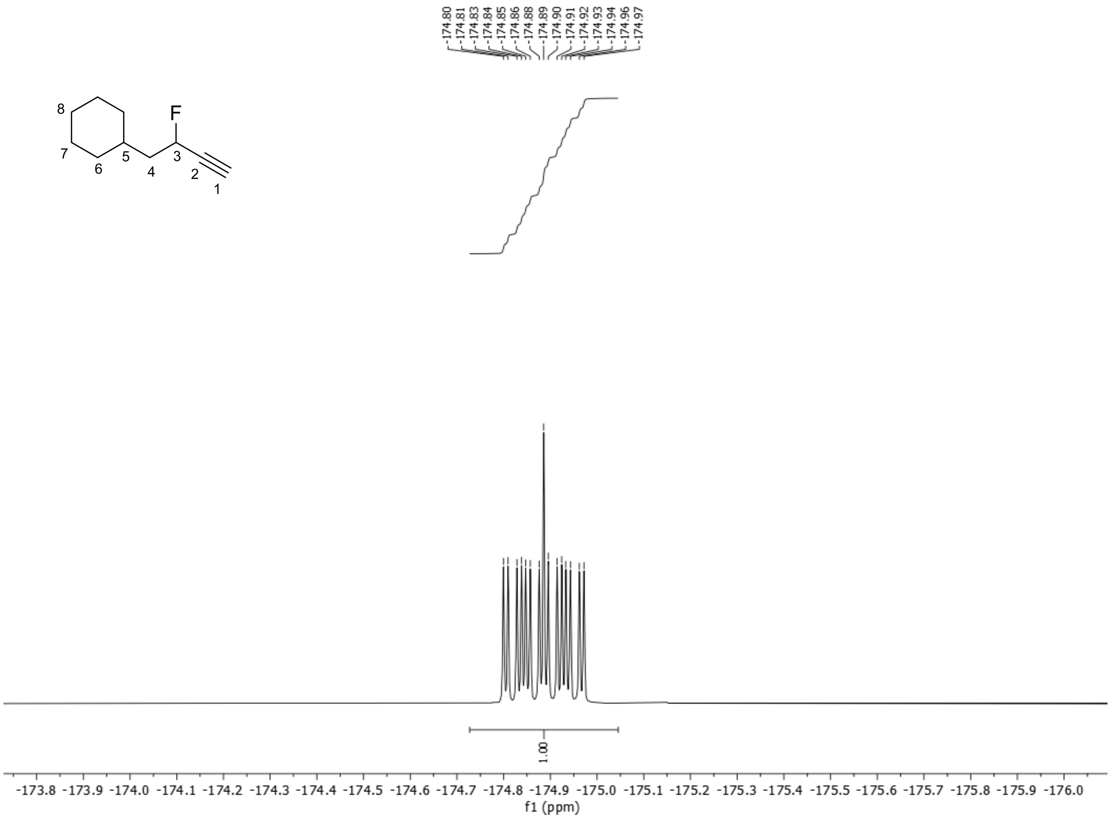

Supplementary Figure 63. ^{19}F NMR of **2** (564 MHz, 299 K, $CDCl_3$).

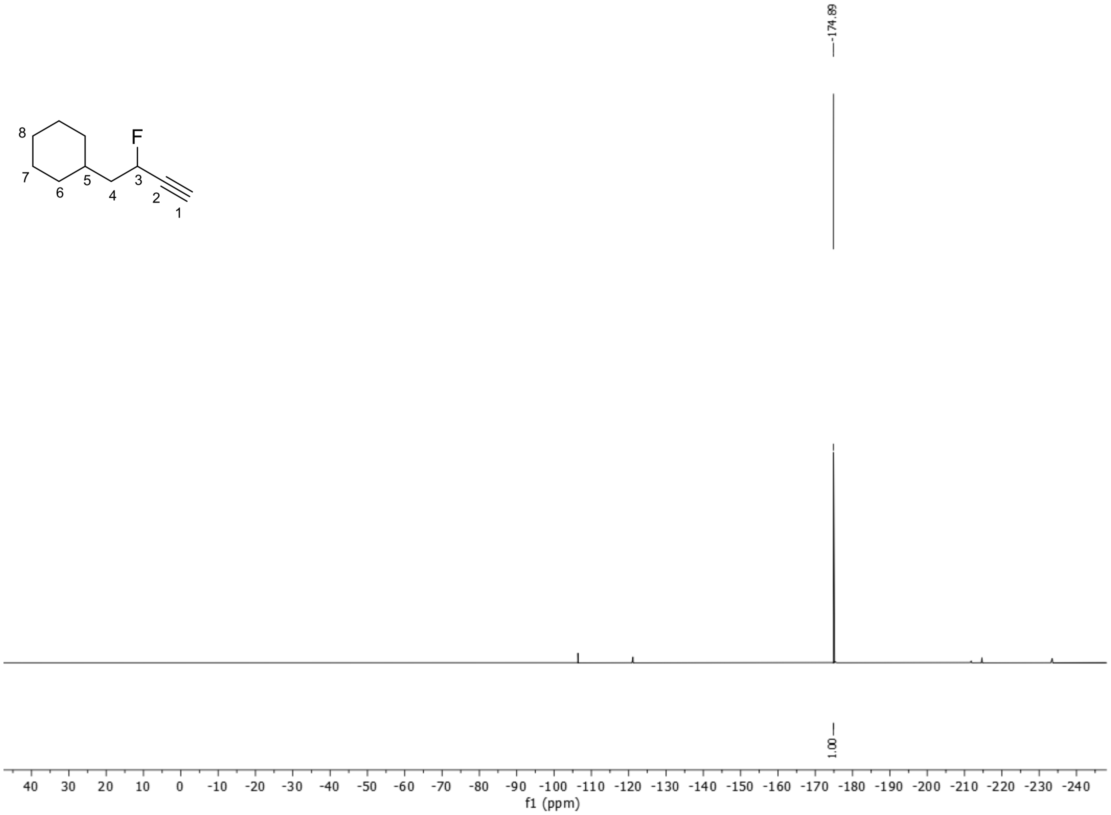

Supplementary Figure 64. ^{19}F{^{1}H} NMR of **2** (377 MHz, 299 K, $CDCl_3$).

12-Bromo-3-fluorododec-1-yne (3)

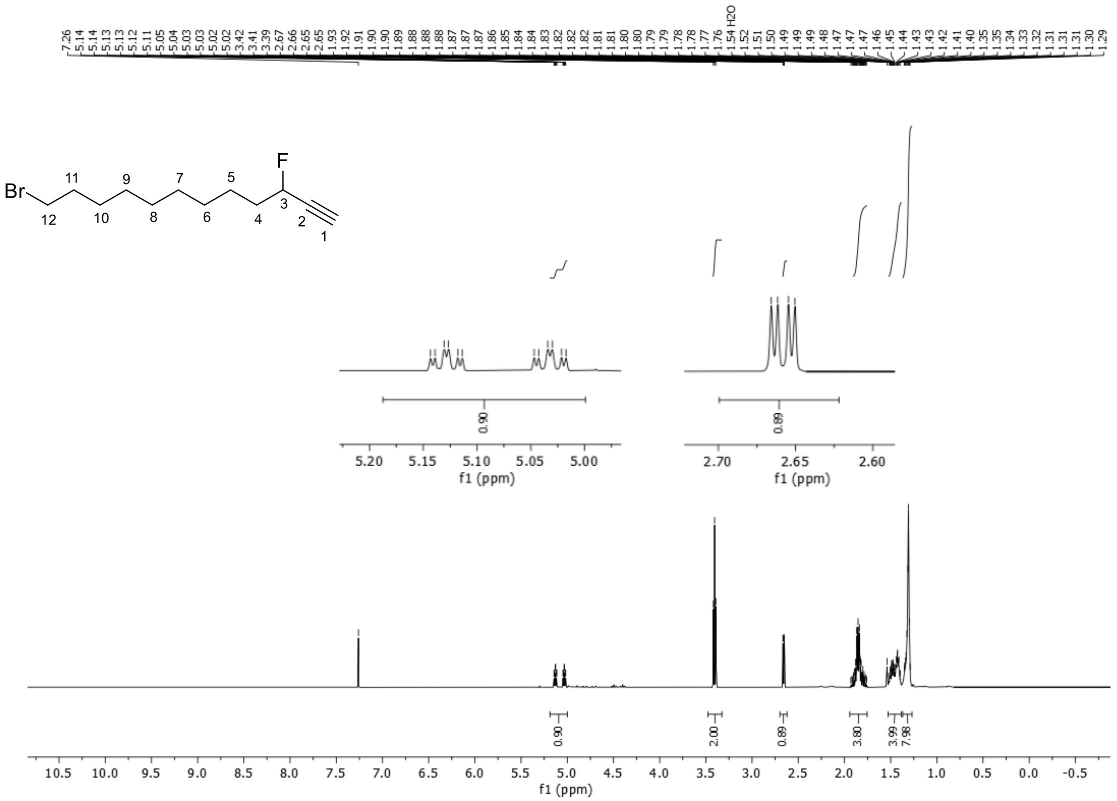

Supplementary Figure 65. ^{1}H NMR of **3** (500 MHz, 299 K, $CDCl_3$).

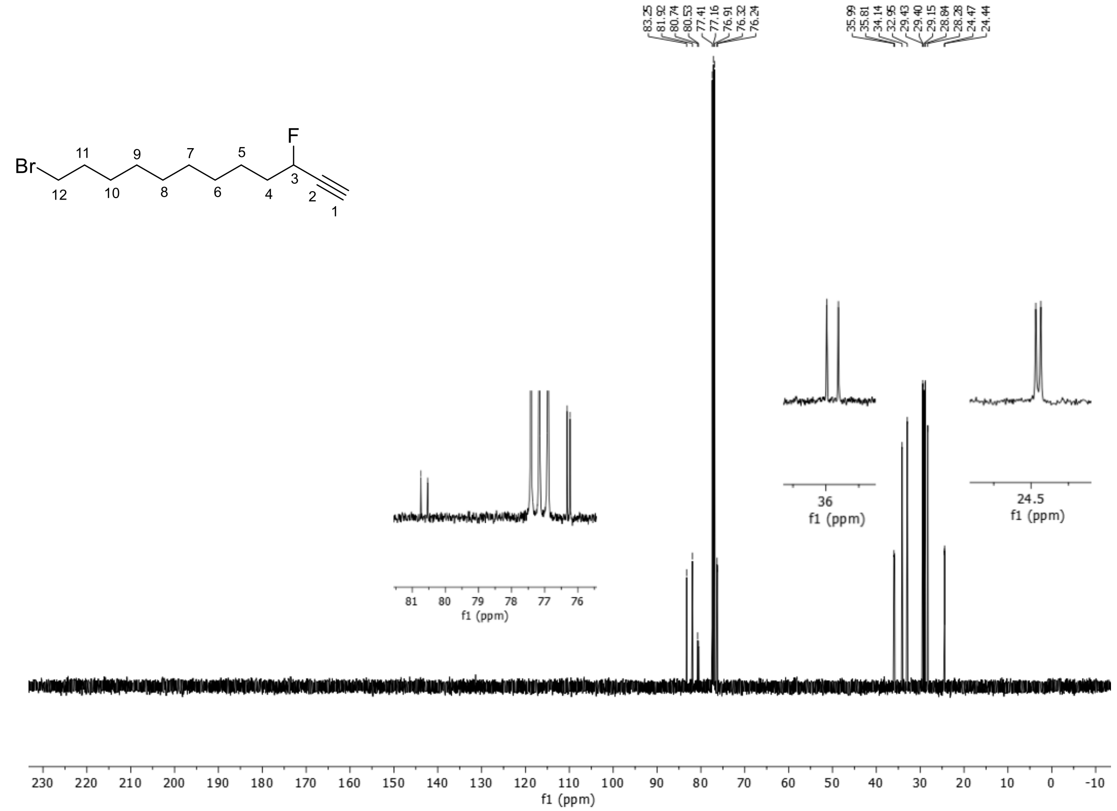

Supplementary Figure 66. $^{13}C\{^{1}H\}$ NMR of **3** (126 MHz, 299 K, $CDCl_3$).

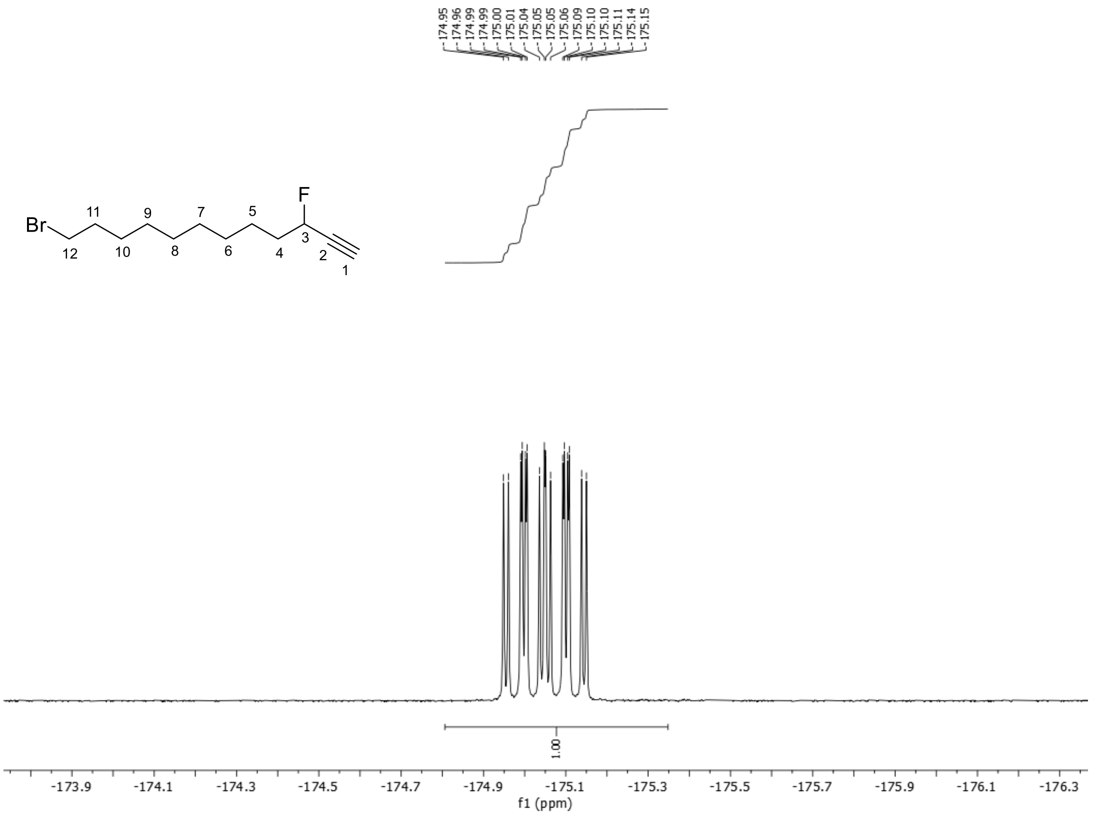

Supplementary Figure 67. ^{19}F NMR of **3** (470 MHz, 299 K, $CDCl_3$).

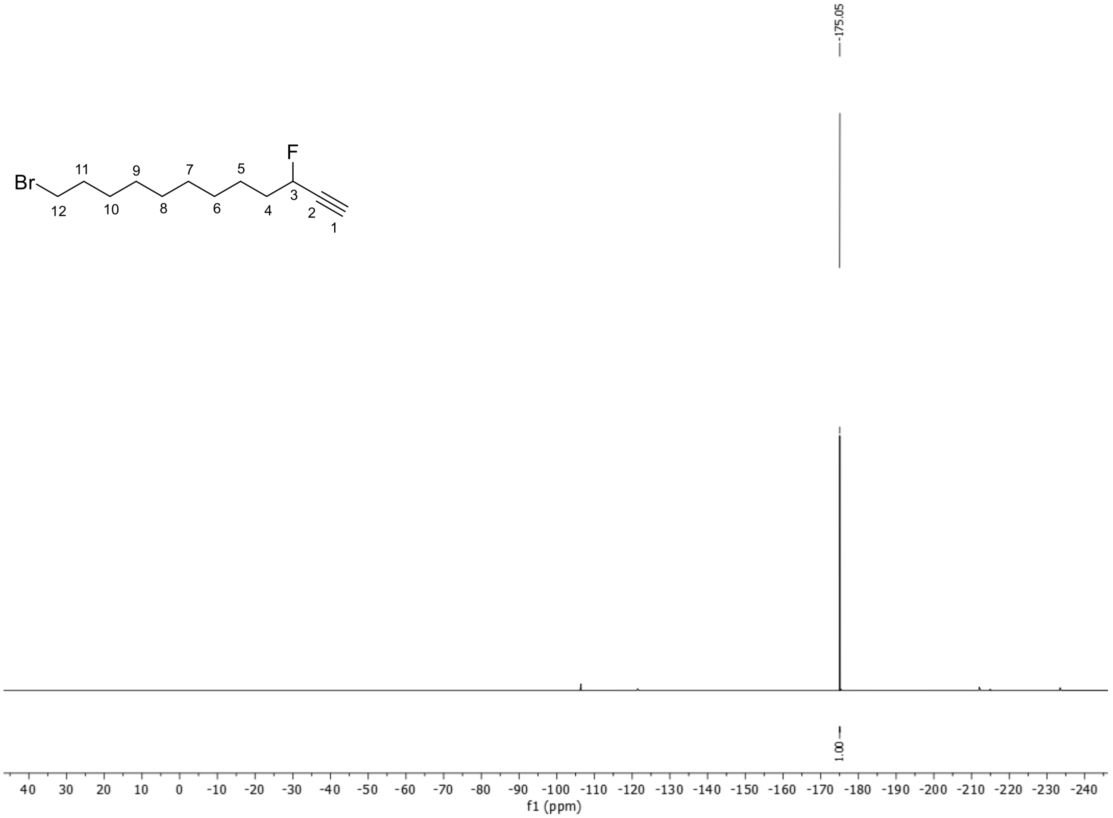

Supplementary Figure 68. ^{19}F{^{1}H} NMR of **3** (377 MHz, 299 K, $CDCl_3$).

1,2-Difluoro-4-(((10-fluorododec-11-yn-1-yl)oxy)methyl)benzene (4)

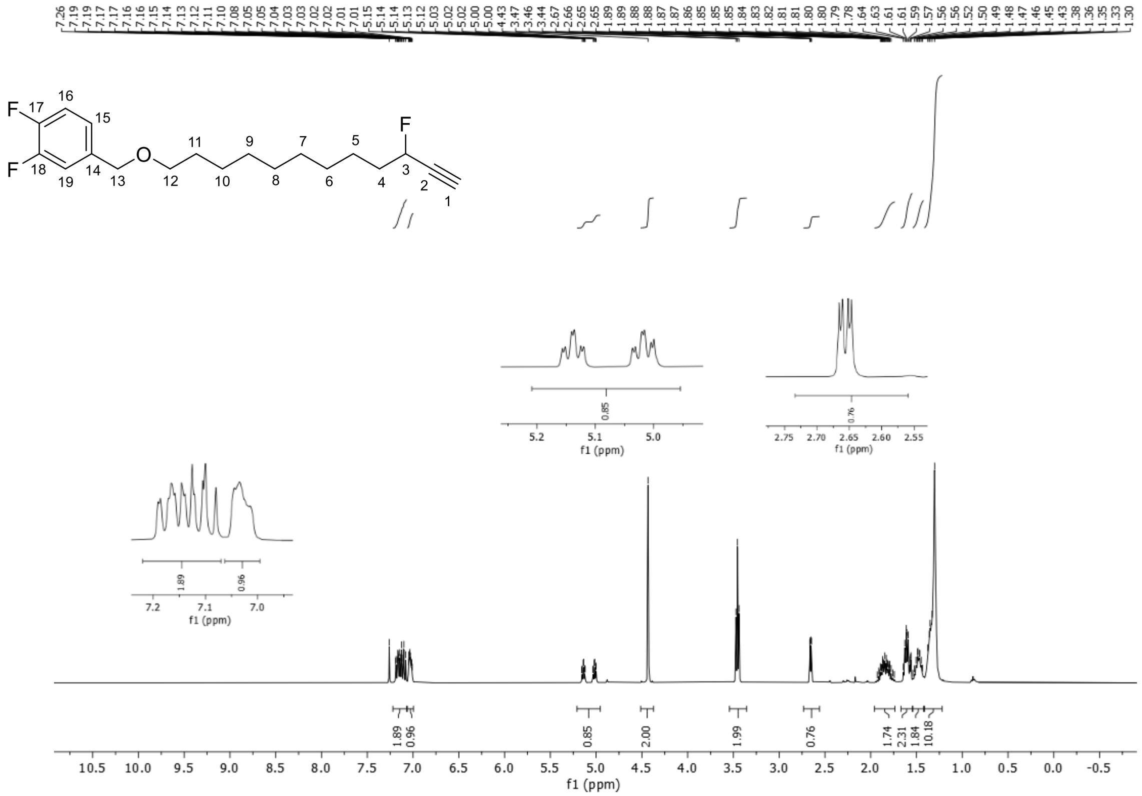

Supplementary Figure 69. ^{1}H NMR of **4** (400 MHz, 299 K, $CDCl_3$).

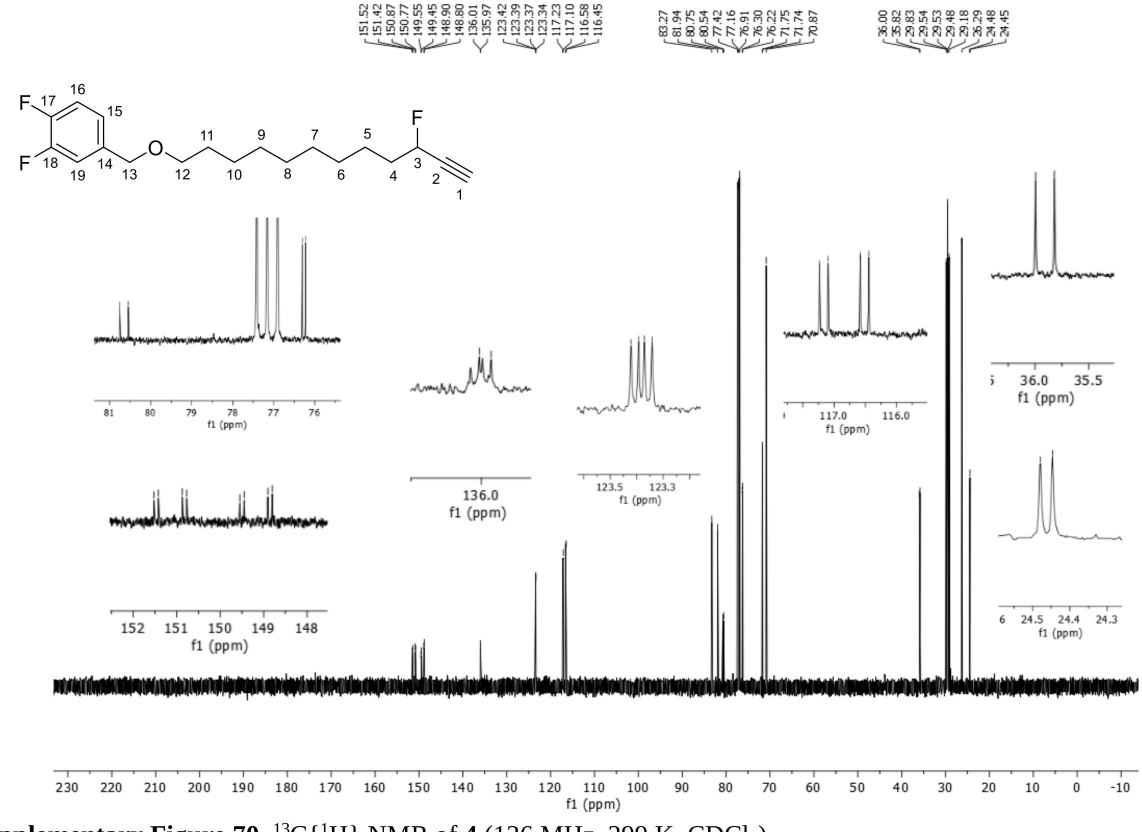

Supplementary Figure 70. $^{13}C\{^{1}H\}$ NMR of **4** (126 MHz, 299 K, $CDCl_3$).

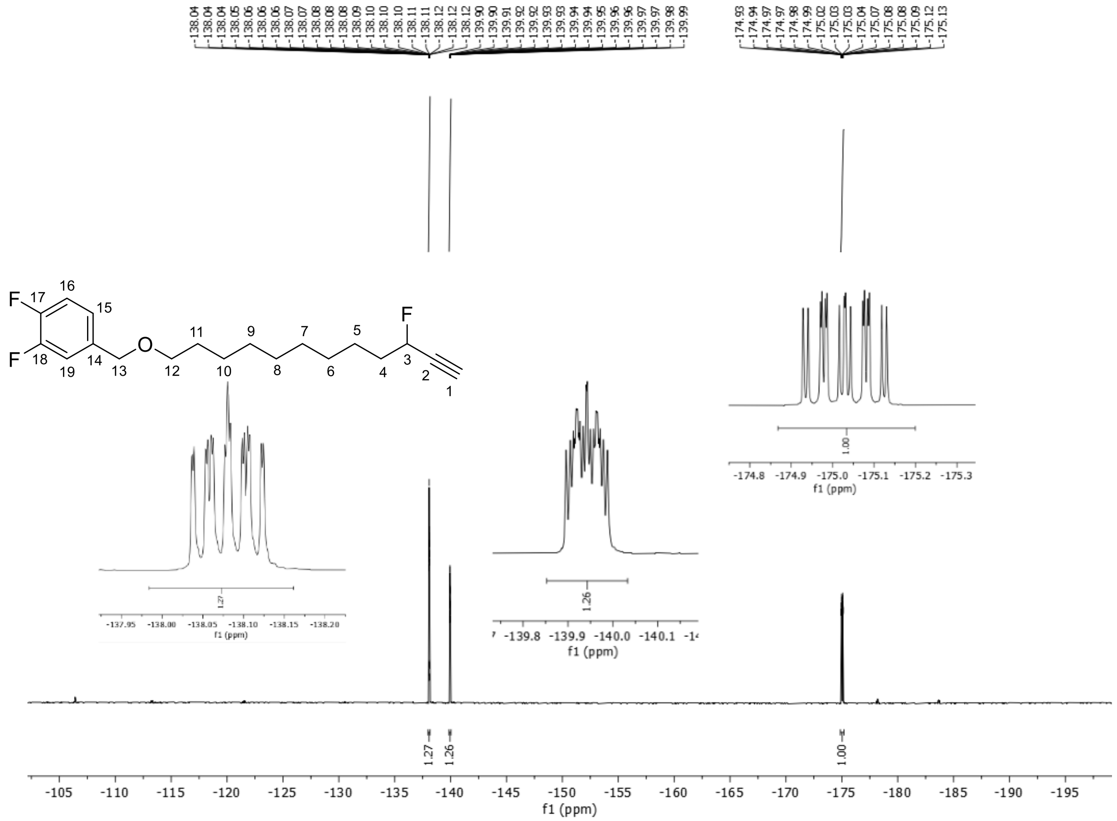

Supplementary Figure 71. ^{19}F NMR of **4** (470 MHz, 299 K, $CDCl_3$).

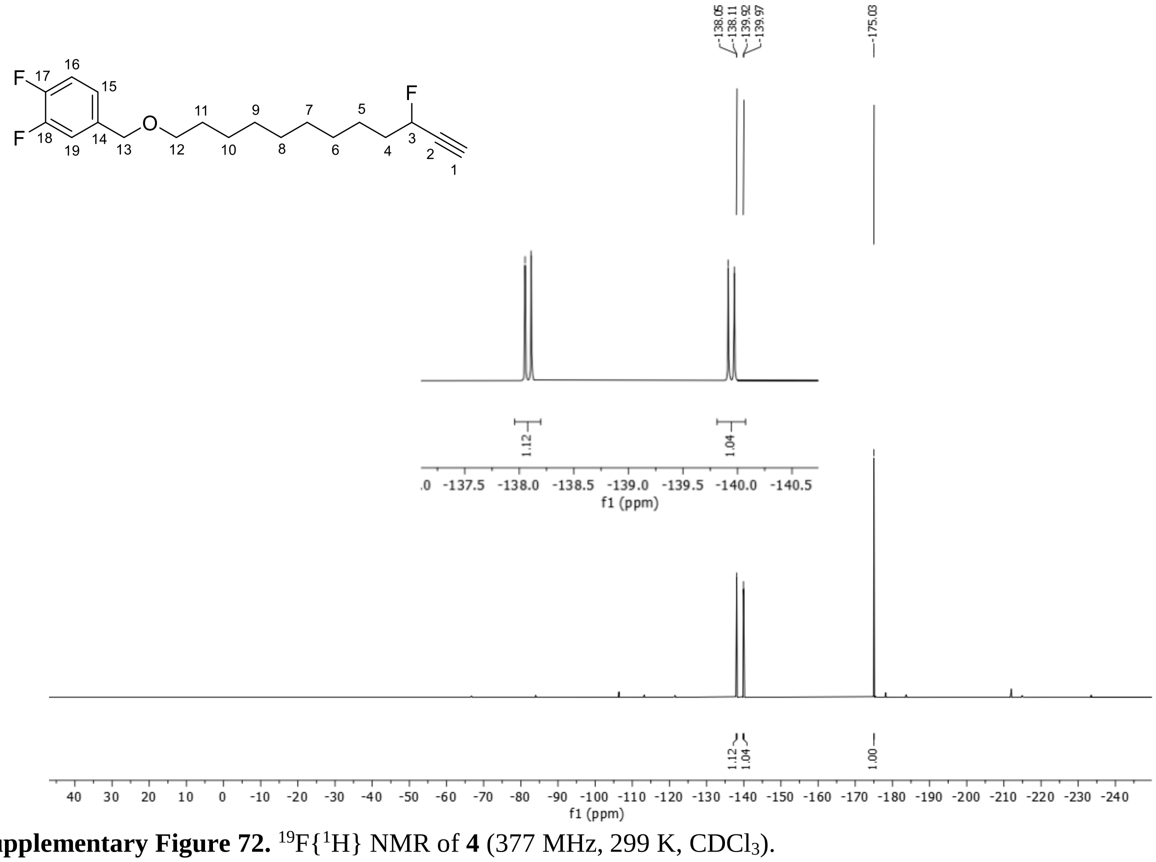

Supplementary Figure 72. $^{19}F\{^{1}H\}$ NMR of **4** (377 MHz, 299 K, $CDCl_3$).

(2-Fluorobut-3-yn-1-yl)benzene (5)

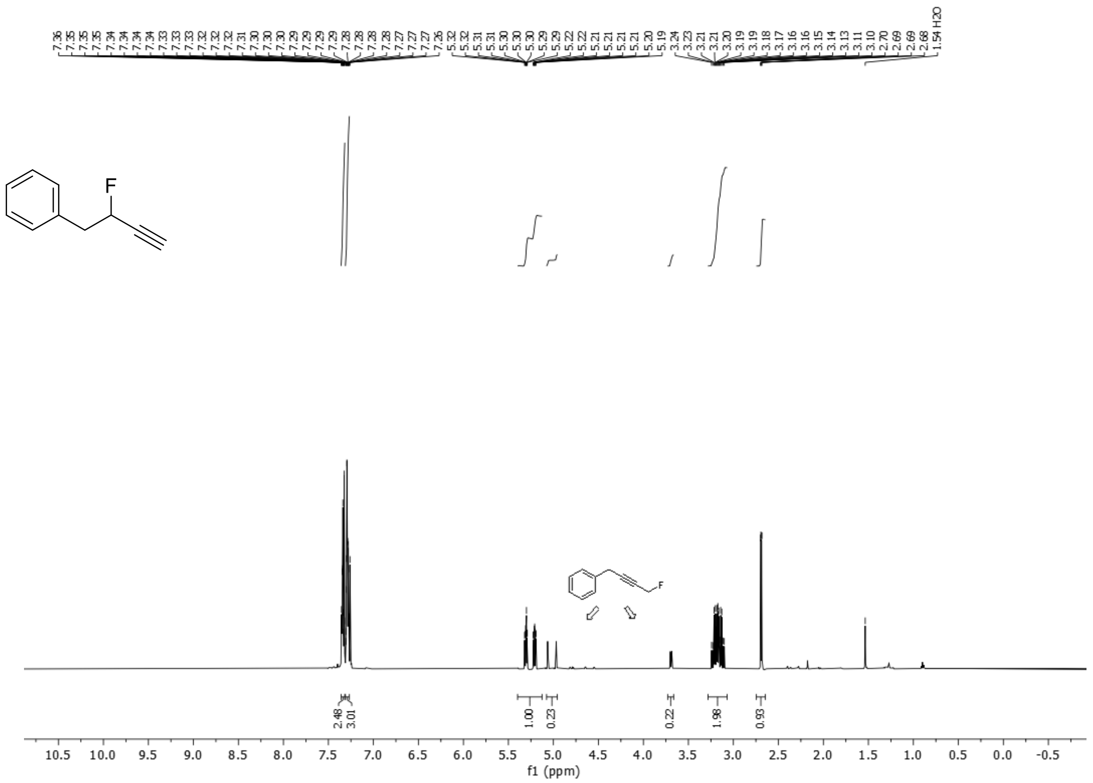

Supplementary Figure 73. ^{1}H NMR of **5** (500 MHz, 299 K, $CDCl_3$).

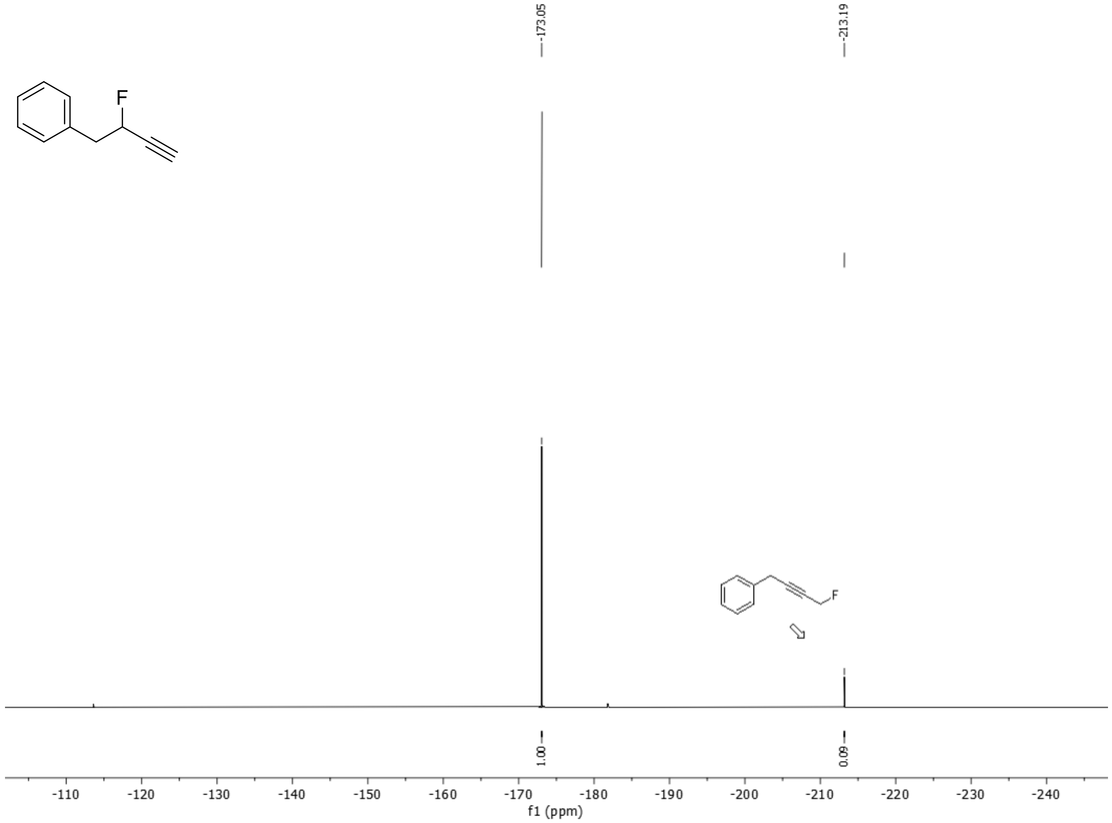

Supplementary Figure 74. ^{19}F{^{1}H} NMR of **5** (470 MHz, 299 K, $CDCl_3$).

10-Fluorododec-11-yn-1-ol (6)

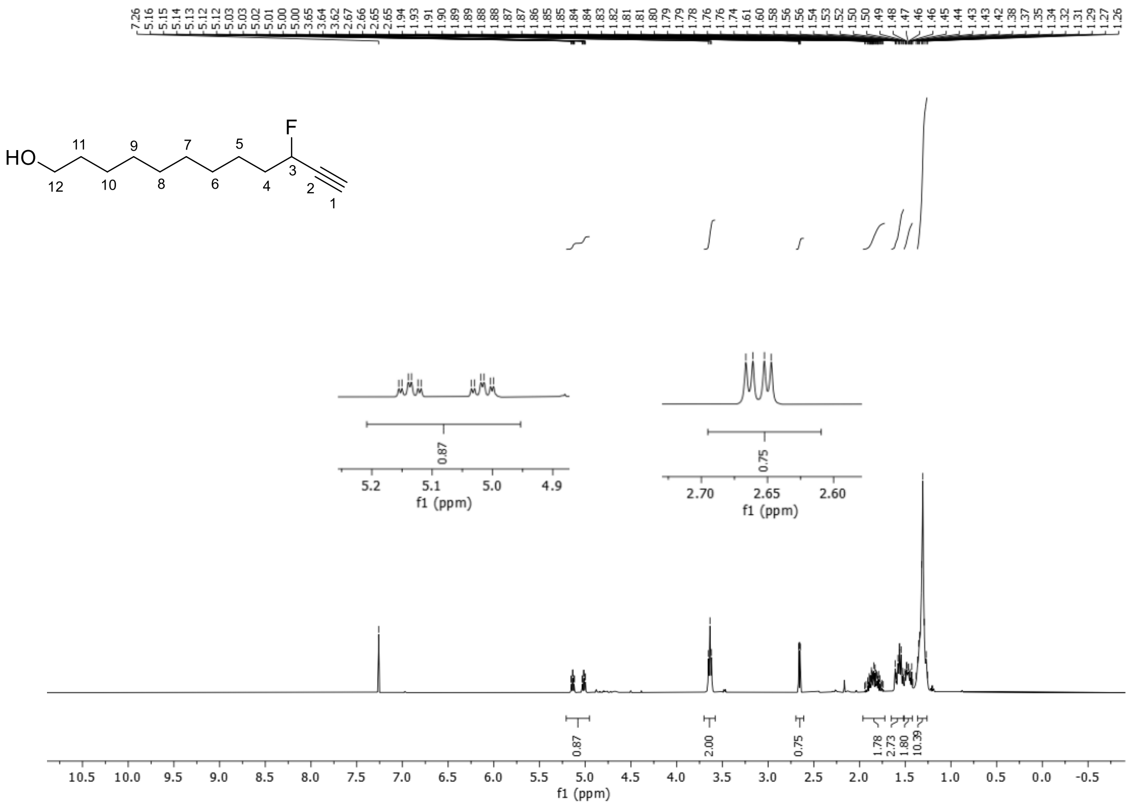

Supplementary Figure 75. ^{1}H NMR of **6** (400 MHz, 299 K, $CDCl_3$).

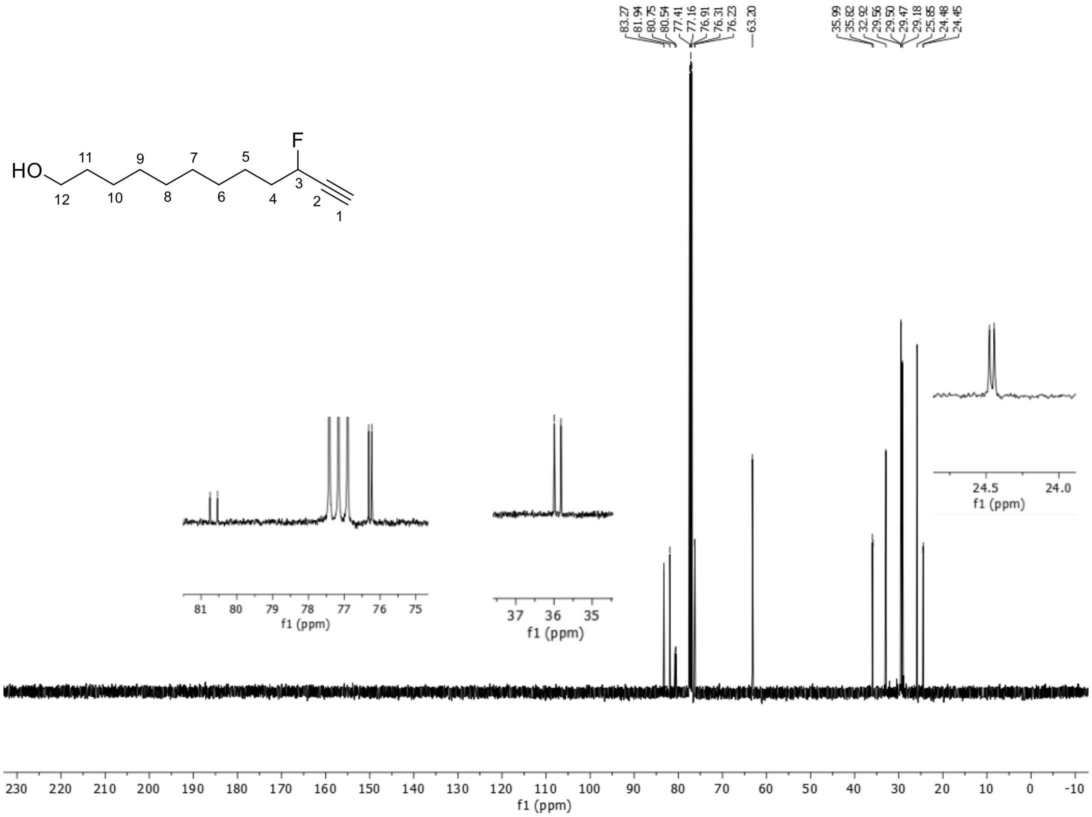

Supplementary Figure 76. $^{13}C\{^{1}H\}$ NMR of **6** (126 MHz, 299 K, $CDCl_3$).

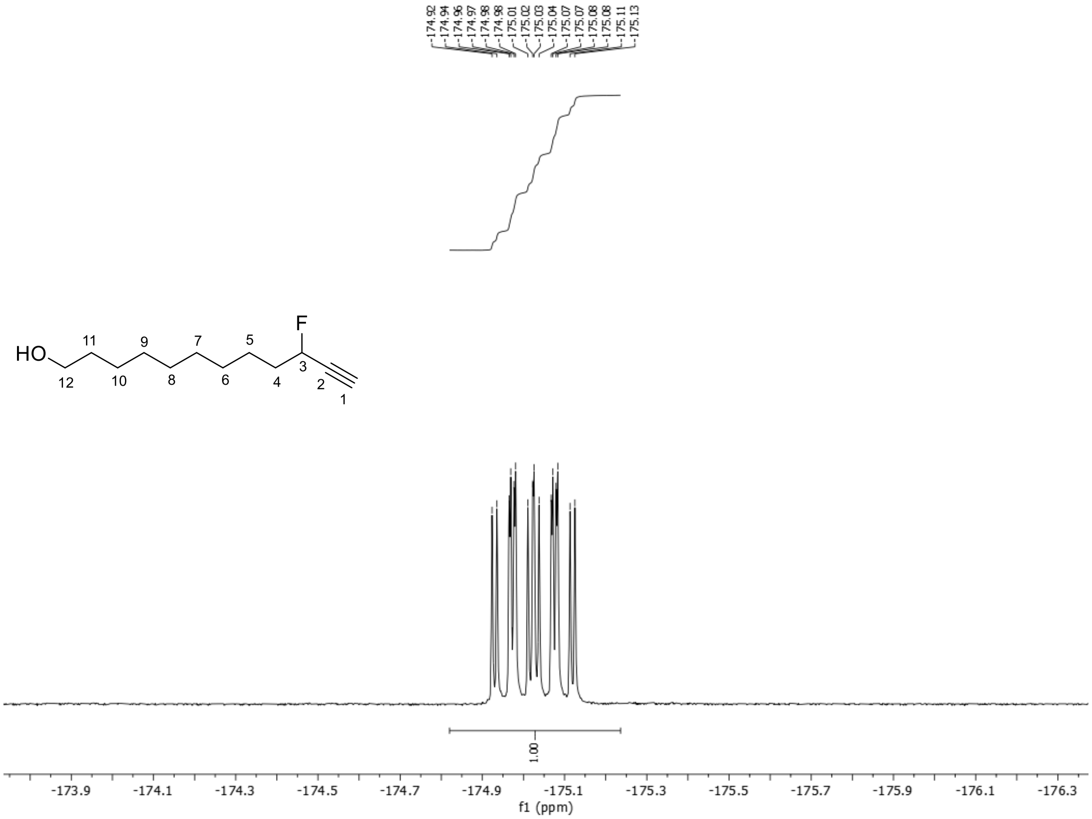

Supplementary Figure 77. ^{19}F NMR of **6** (470 MHz, 299 K, $CDCl_3$).

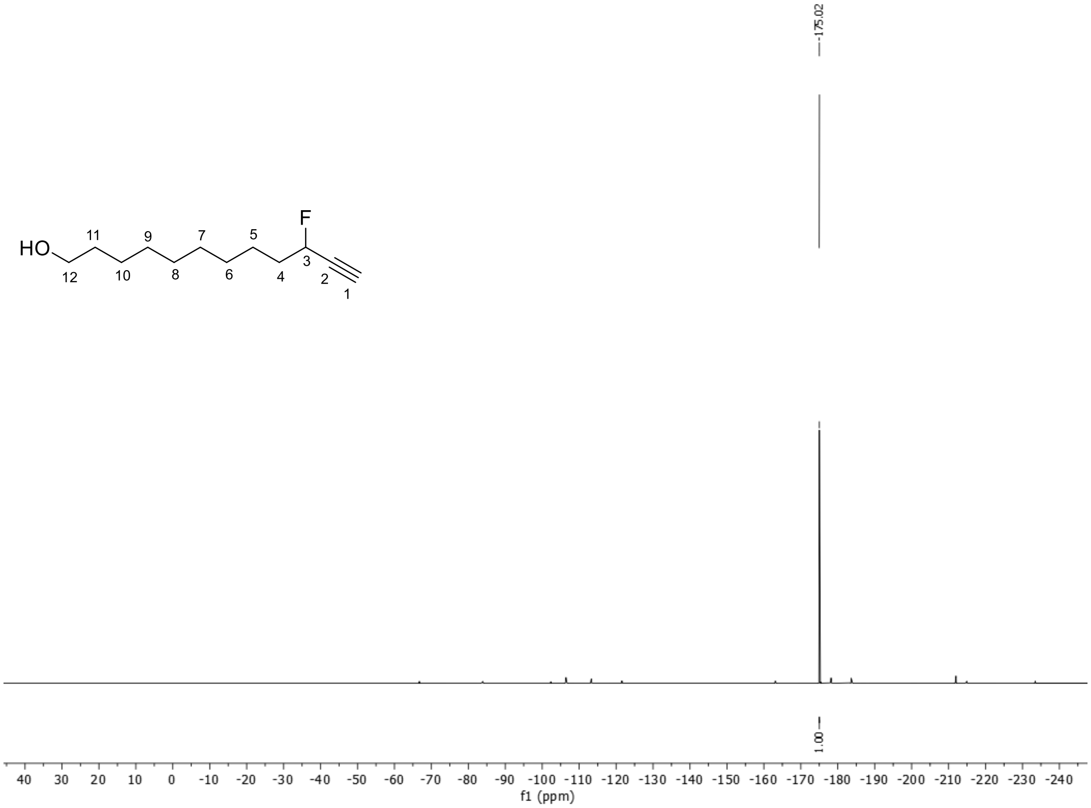

Supplementary Figure 78. $^{19}F\{^1H\}$ NMR of **6** (377 MHz, 299 K, $CDCl_3$).

10-Fluorododec-11-yn-1-yl 4-methylbenzenesulfonate (7)

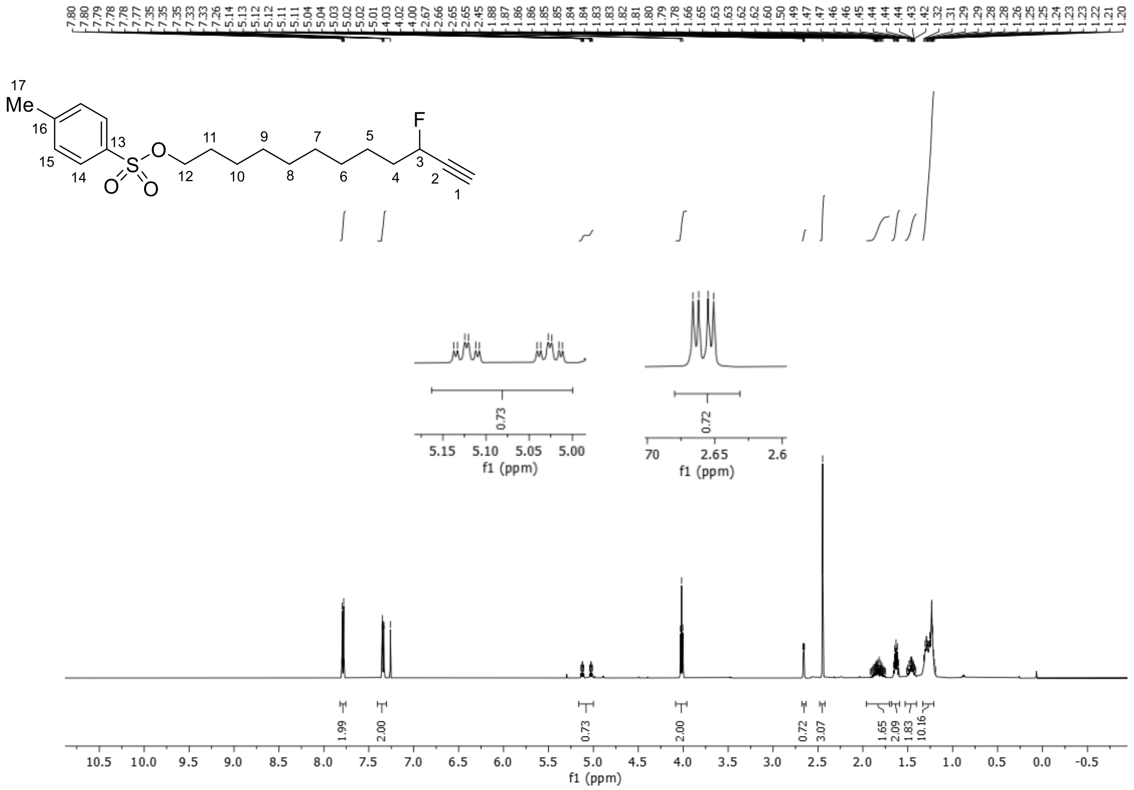

Supplementary Figure 79. ^{1}H NMR of **7** (500 MHz, 299 K, $CDCl_3$).

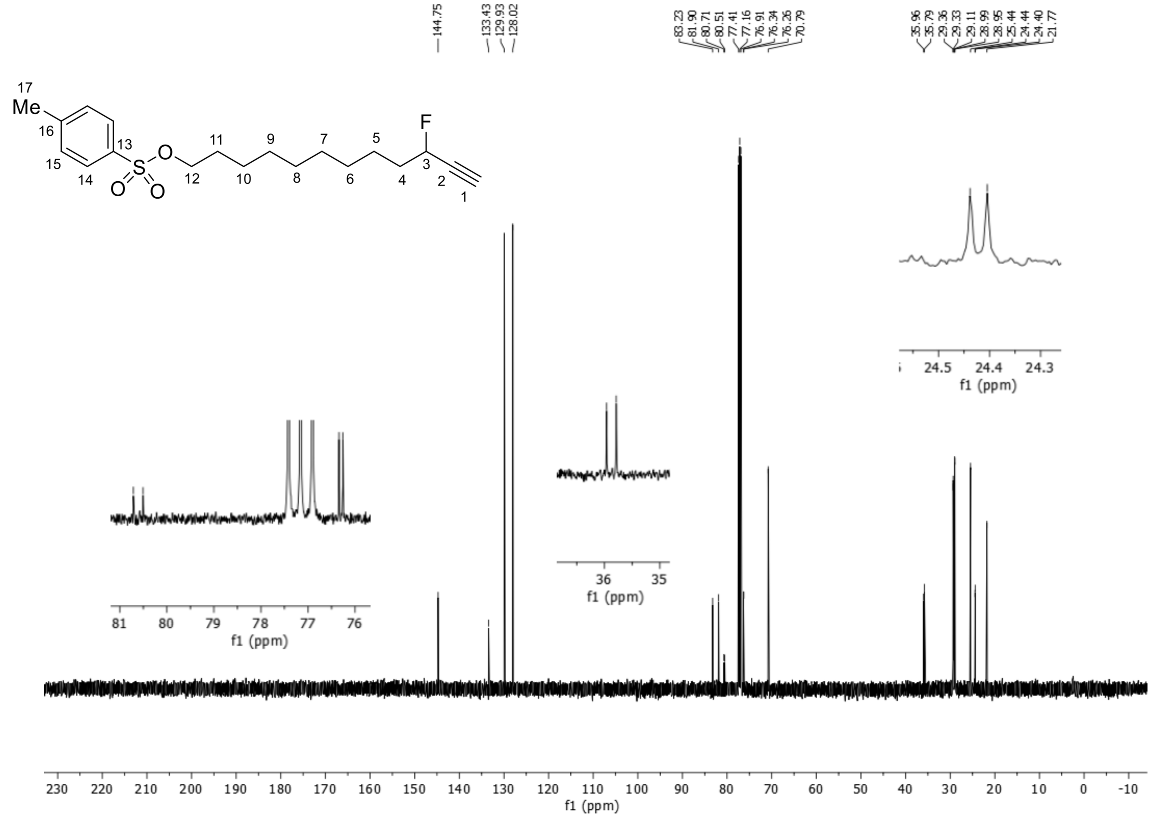

Supplementary Figure 80. $^{13}C\{^{1}H\}$ NMR of **7** (126 MHz, 299 K, $CDCl_3$).

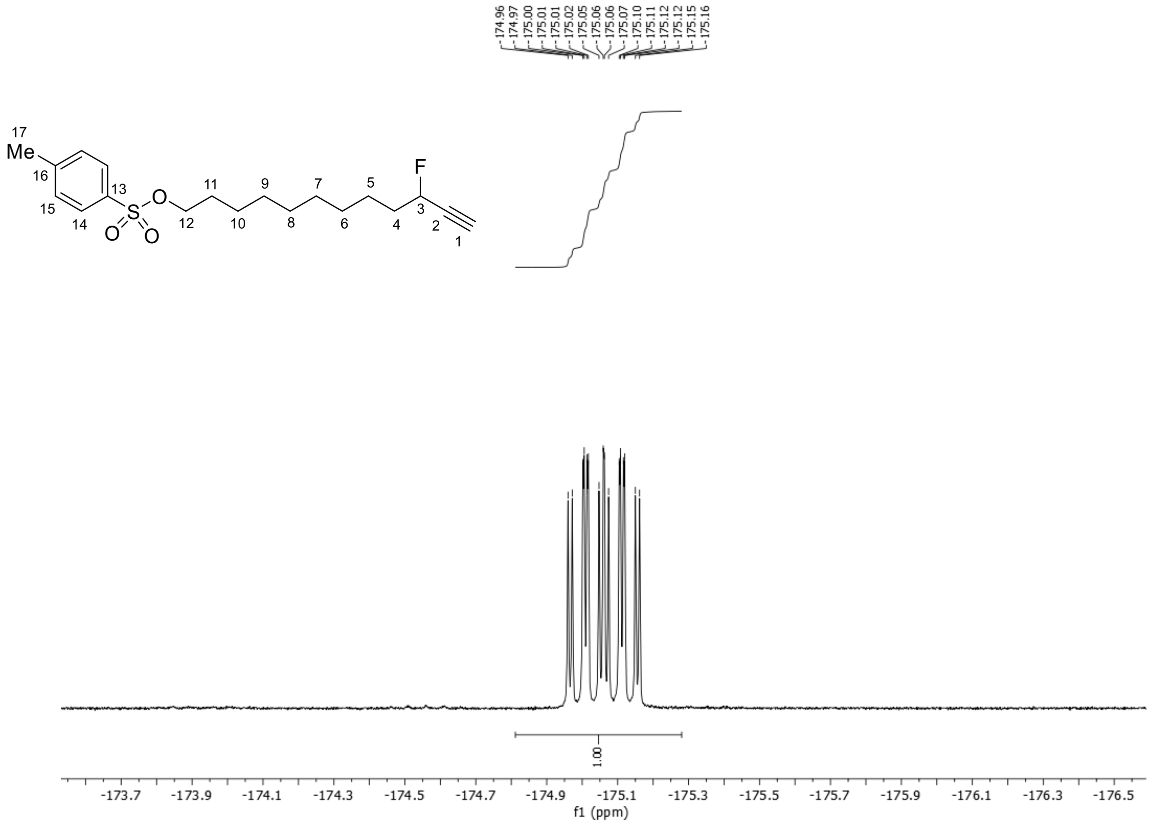

Supplementary Figure 81. ^{19}F NMR of **7** (470 MHz, 299 K, $CDCl_3$).

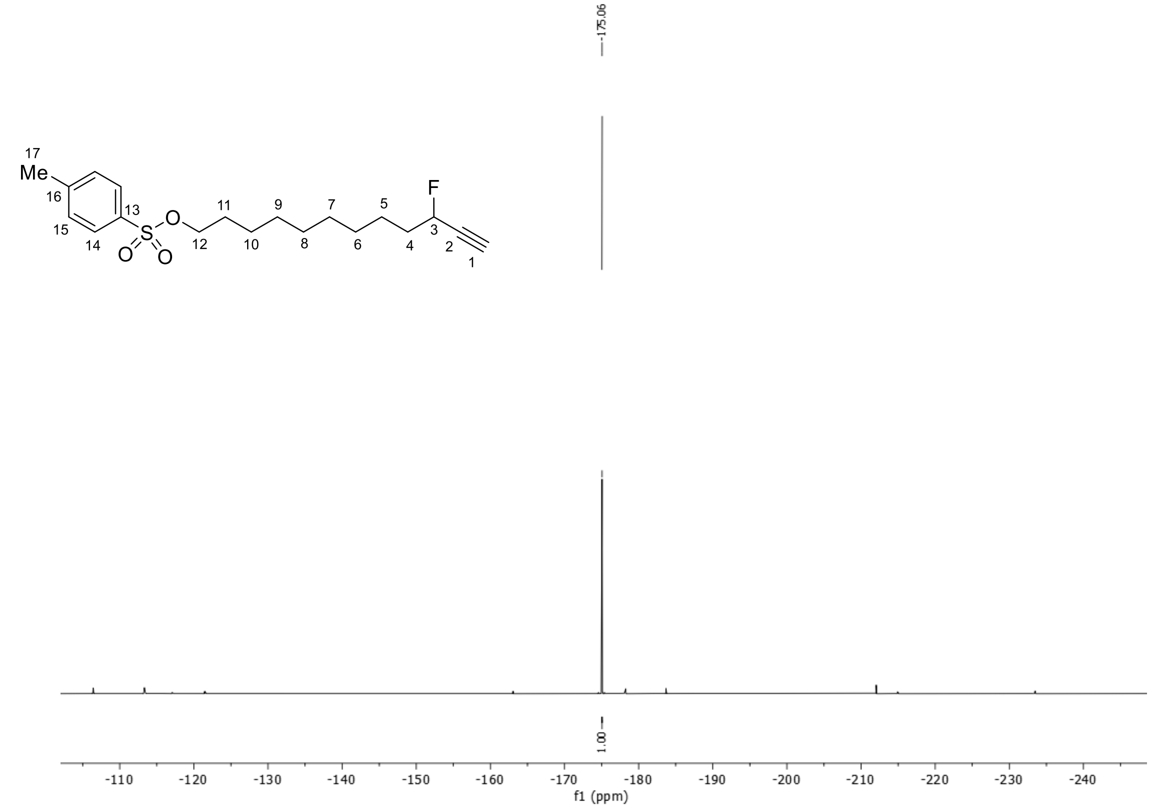

Supplementary Figure 82. $^{19}F\{^1H\}$ NMR of **7** (470 MHz, 299 K, $CDCl_3$).

2-(10-Fluorododec-11-yn-1-yl)isoindoline-1,3-dione (8)

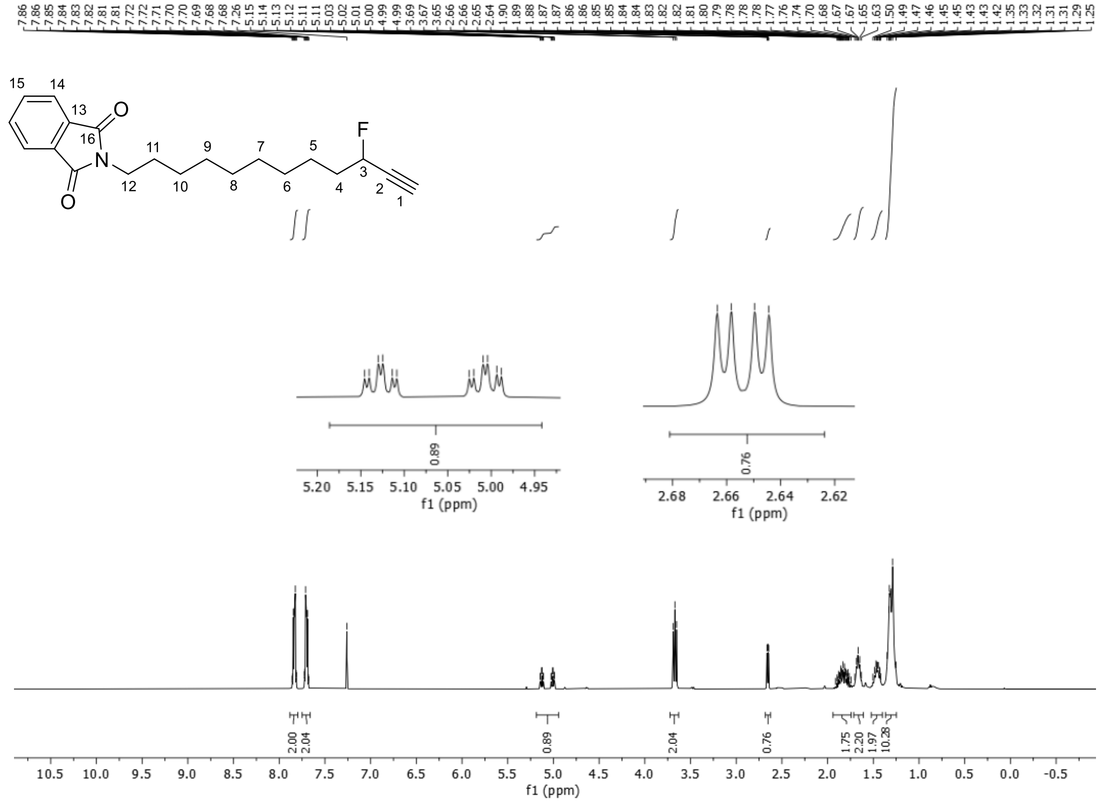

Supplementary Figure 83. ^{1}H NMR of **8** (400 MHz, 299 K, $CDCl_3$).

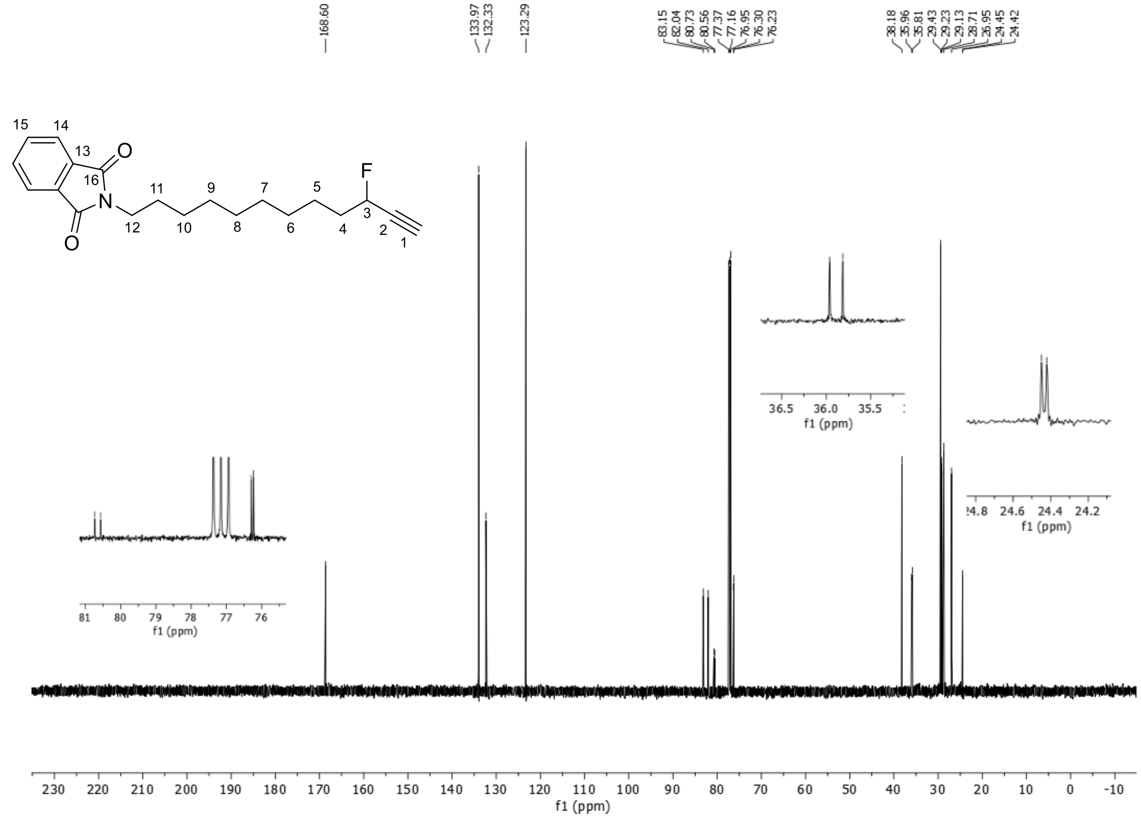

Supplementary Figure 84. $^{13}C\{^{1}H\}$ NMR of **8** (151 MHz, 299 K, $CDCl_3$).

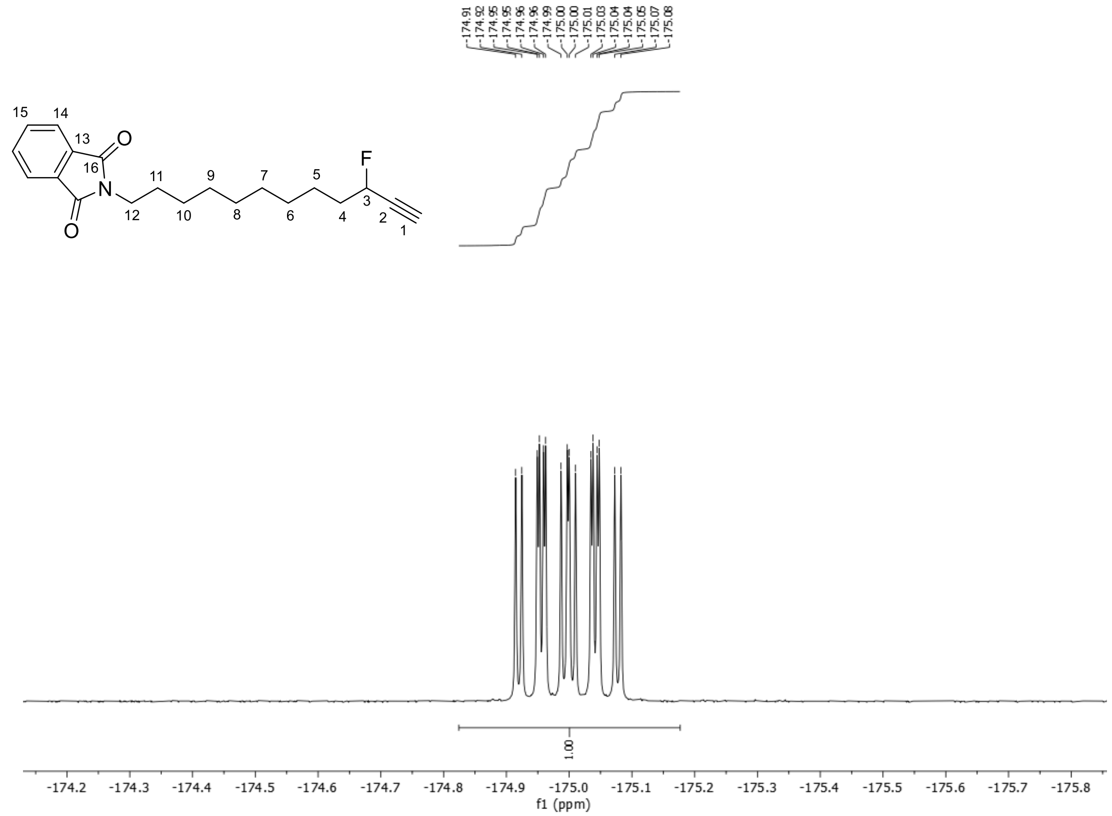

Supplementary Figure 85. ^{19}F NMR of **8** (564 MHz, 299 K, $CDCl_3$).

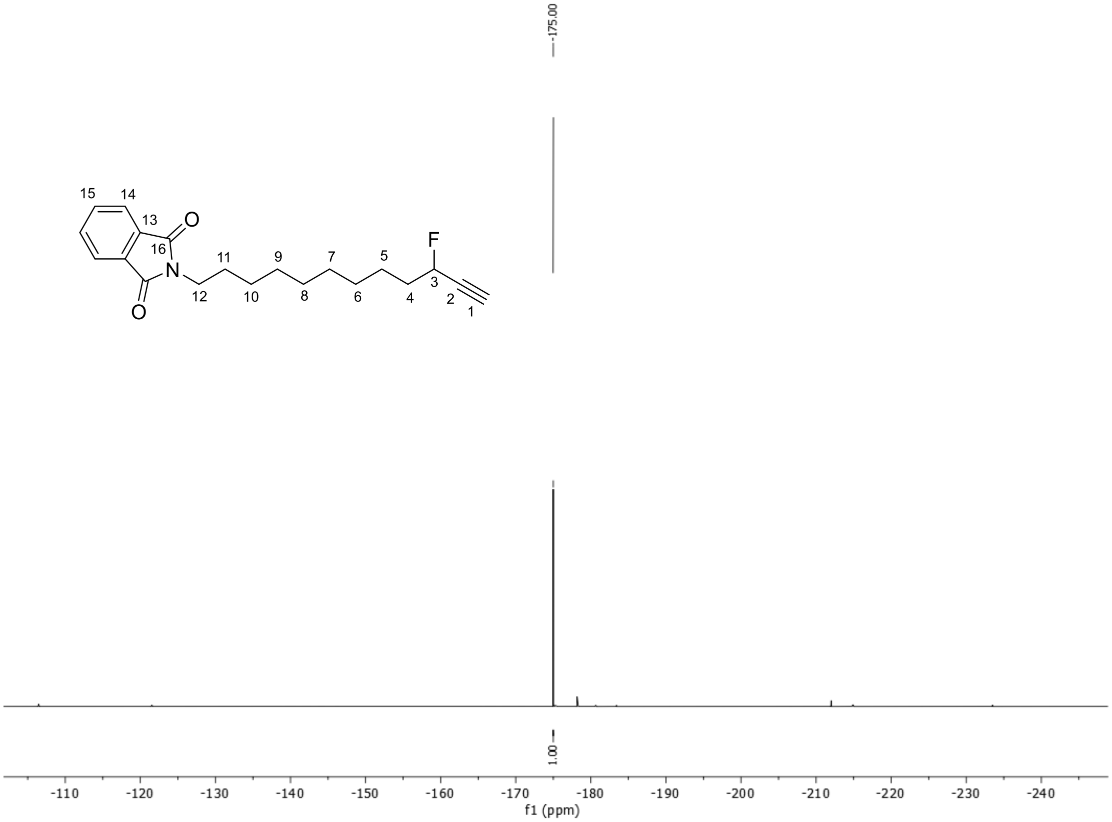

Supplementary Figure 86. $^{19}F\{^{1}H\}$ NMR of **8** (564 MHz, 299 K, $CDCl_3$).

10-Fluorododec-11-yn-1-yl nicotinate (9)

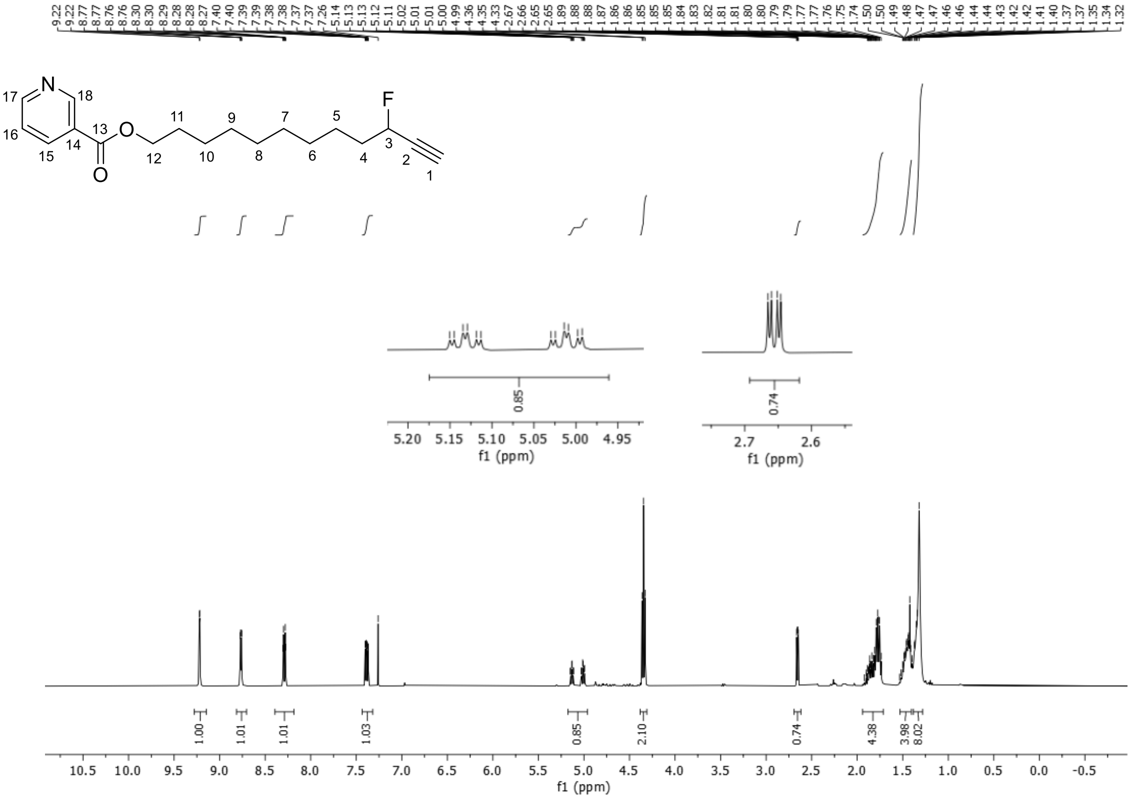

Supplementary Figure 87. ^{1}H NMR of **9** (400 MHz, 299 K, $CDCl_3$).

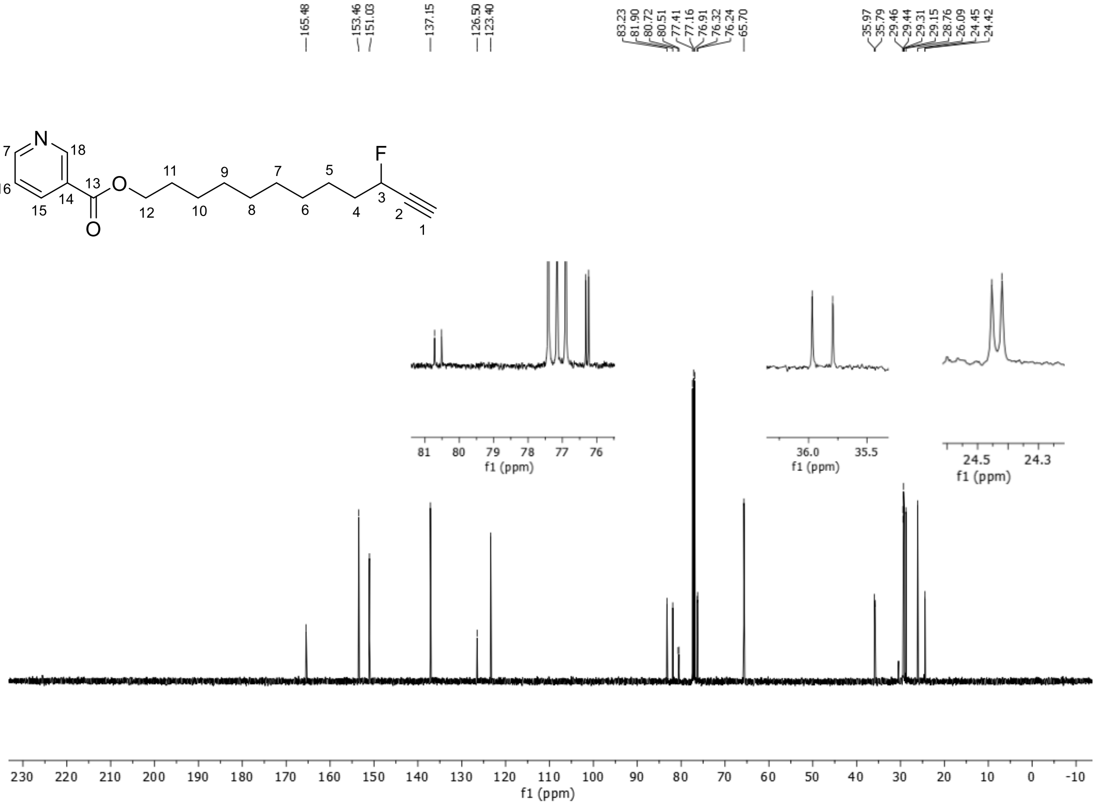

Supplementary Figure 88. $^{13}C\{^{1}H\}$ NMR of **9** (126 MHz, 299 K, $CDCl_3$).

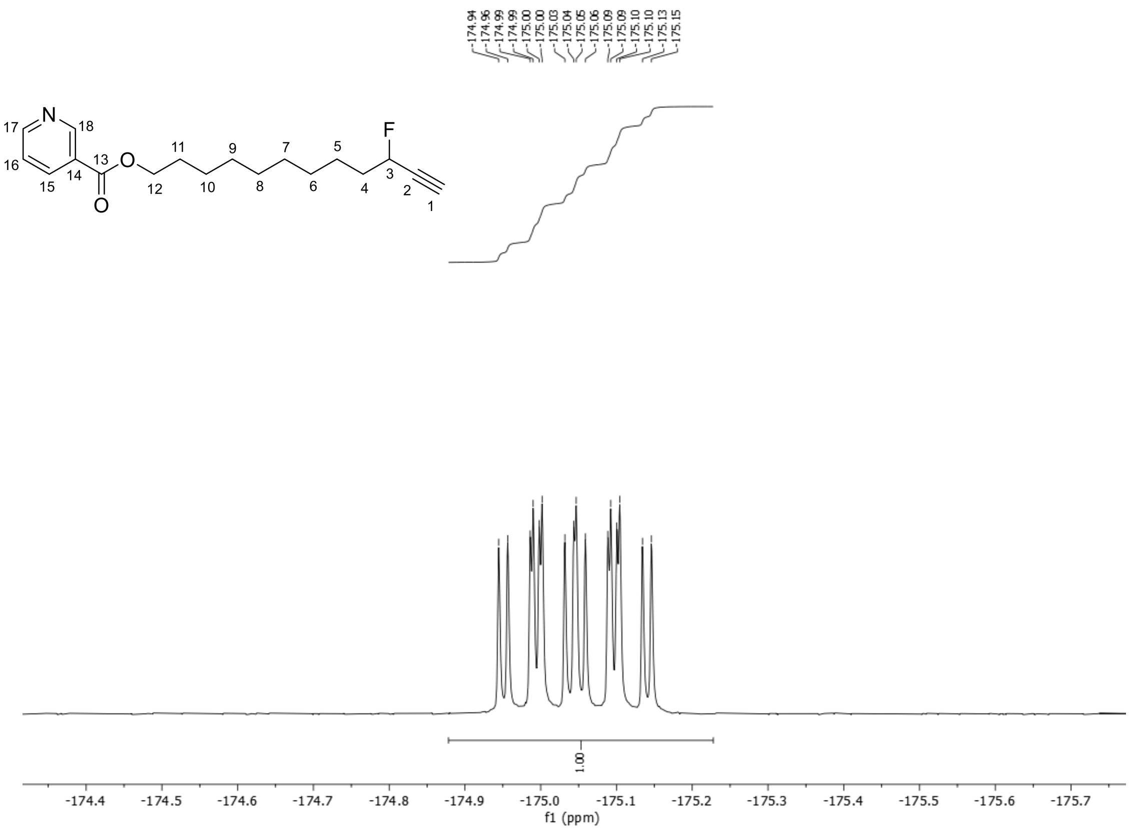

Supplementary Figure 89. ^{19}F NMR of **9** (470 MHz, 299 K, $CDCl_3$).

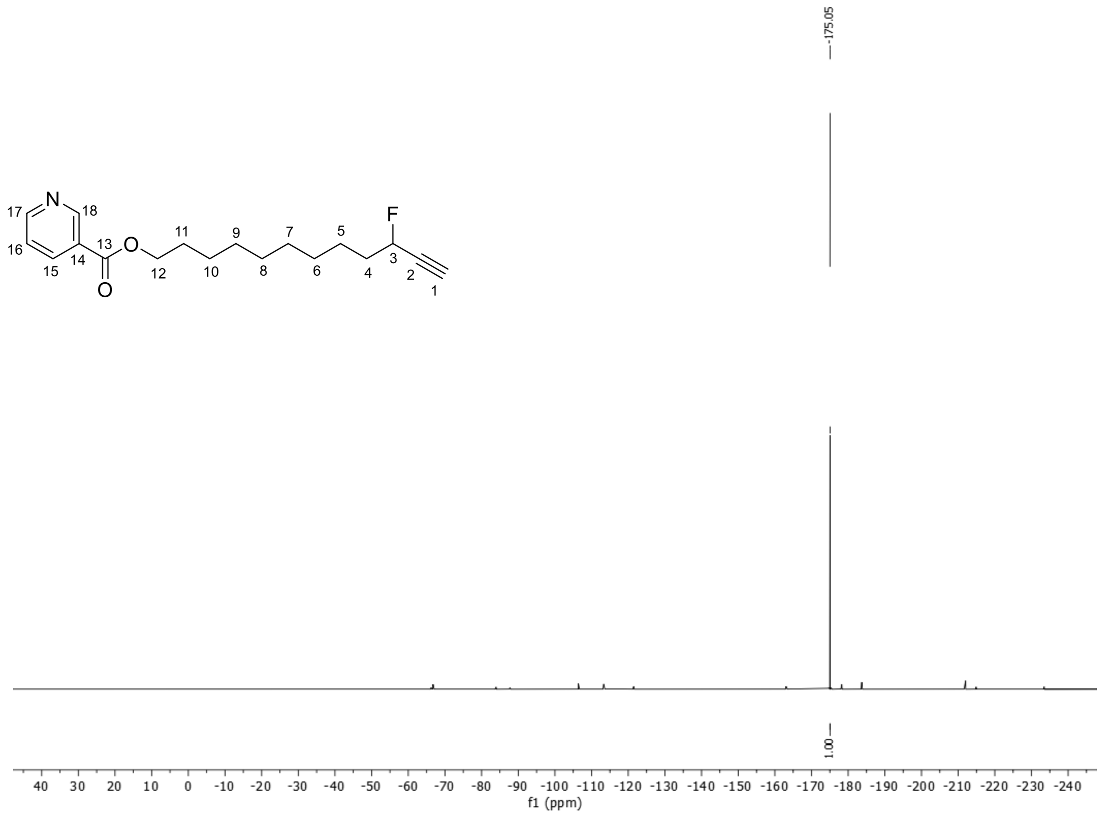

Supplementary Figure 90. $^{19}F\{^1H\}$ NMR of **9** (377 MHz, 299 K, $CDCl_3$).

10-Fluorododec-11-yn-1-yl 2-methylthiazole-4-carboxylate (10)

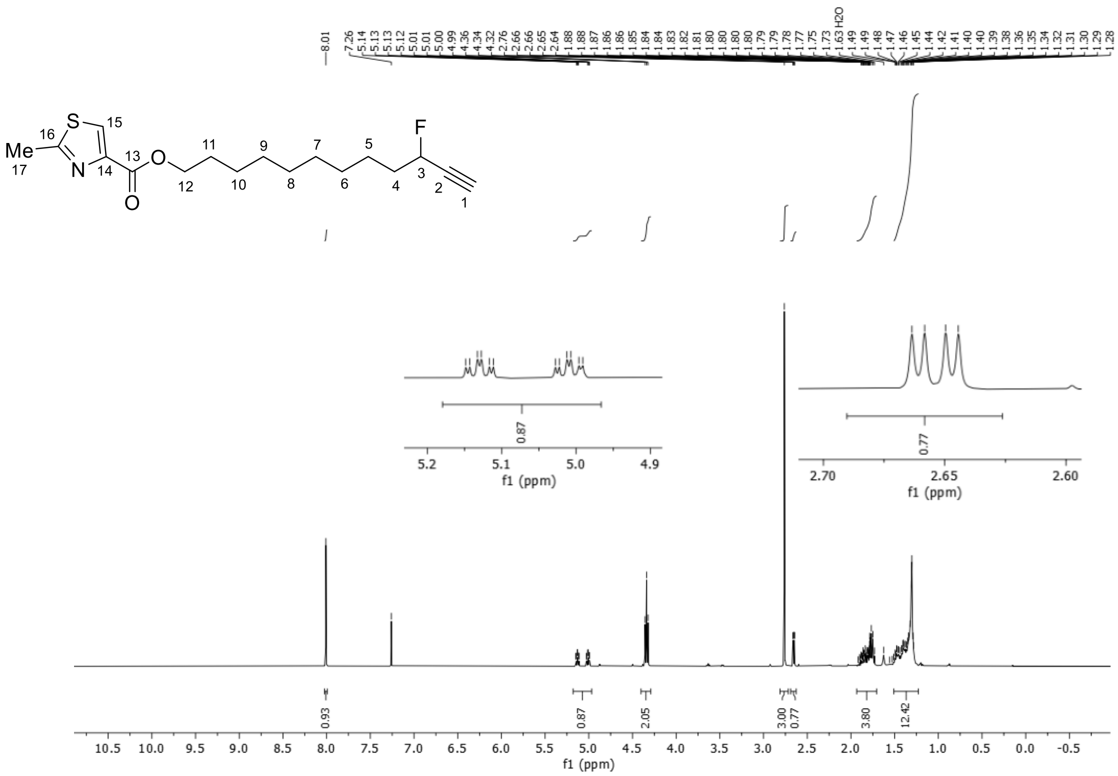

Supplementary Figure 91. ^{1}H NMR of **10** (400 MHz, 299 K, $CDCl_3$).

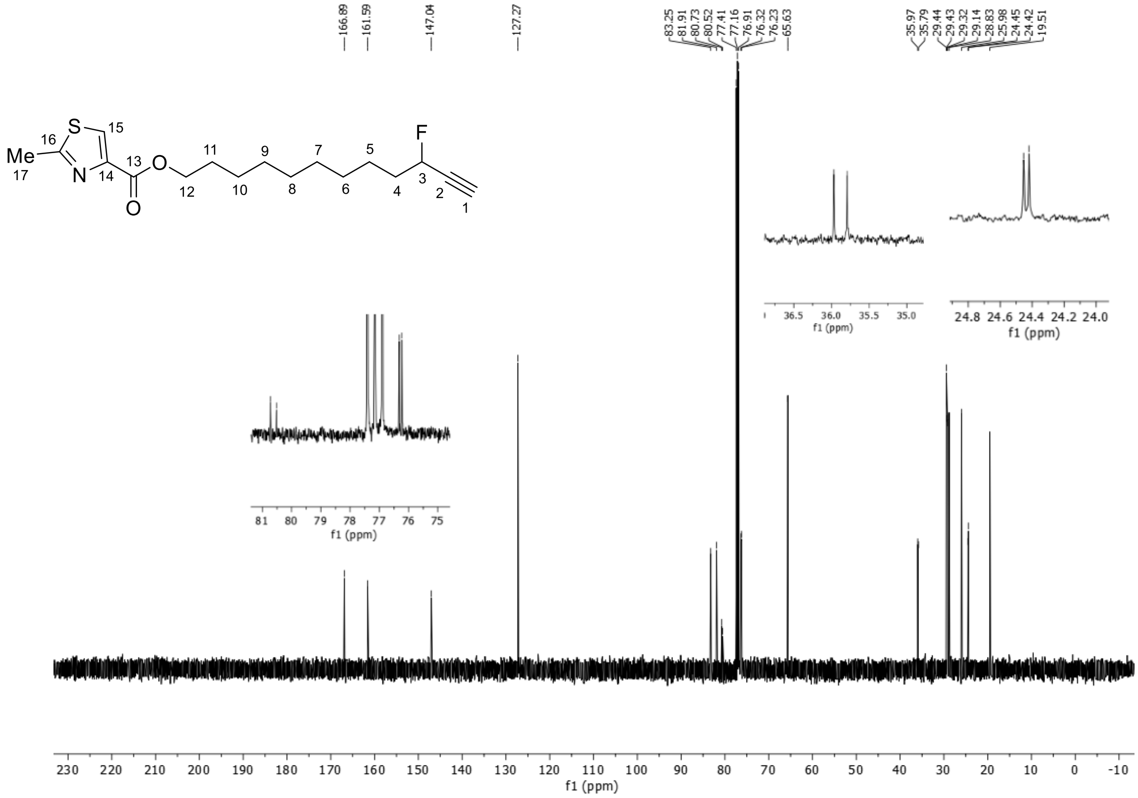

Supplementary Figure 92. $^{13}C\{^{1}H\}$ NMR of **10** (126 MHz, 299 K, $CDCl_3$).

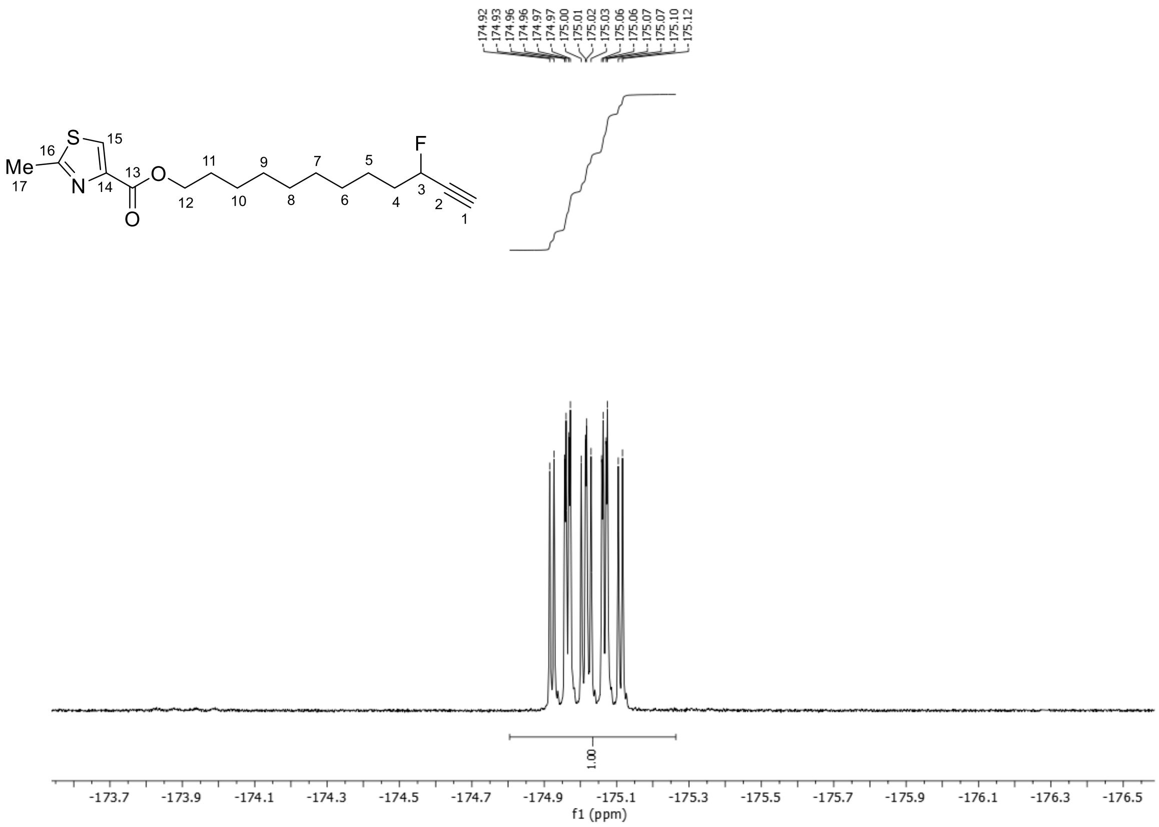

Supplementary Figure 93. ^{19}F NMR of **10** (470 MHz, 299 K, $CDCl_3$).

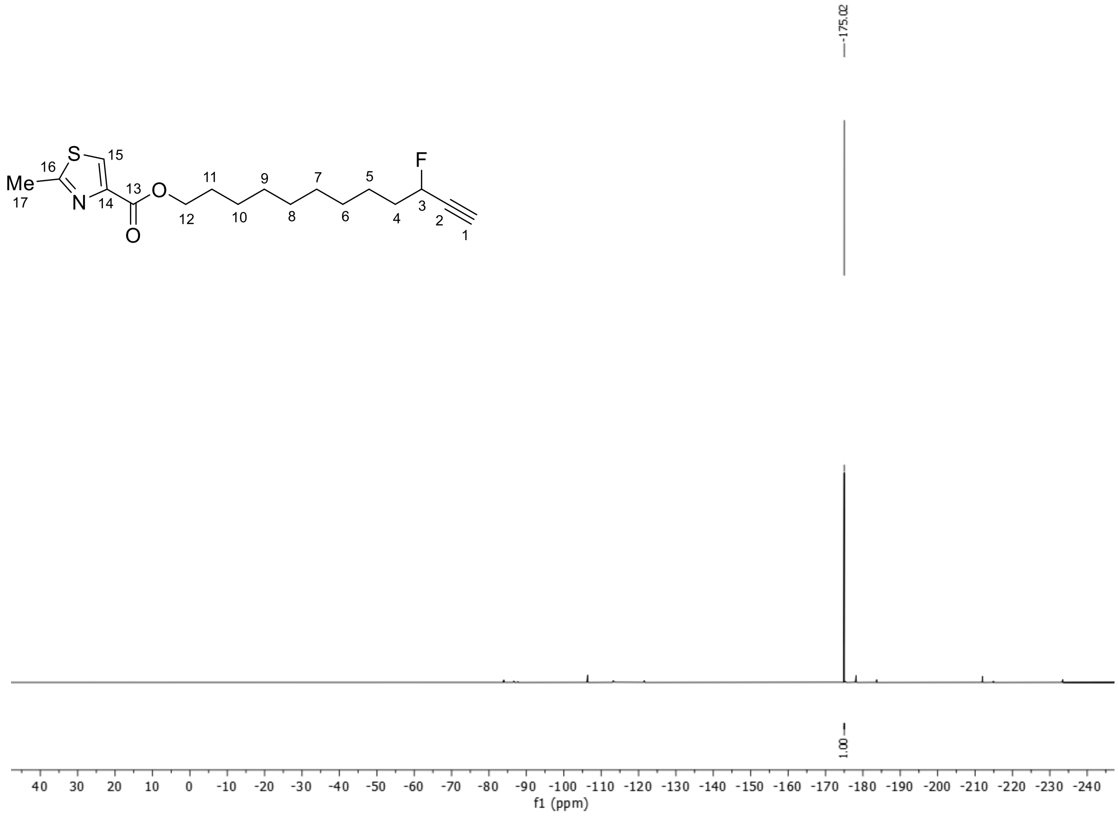

Supplementary Figure 94. $^{19}F\{^{1}H\}$ NMR of **10** (377 MHz, 299 K, $CDCl_3$).

10-Fluorododec-11-yn-1-yl cyclopropanecarboxylate (11)

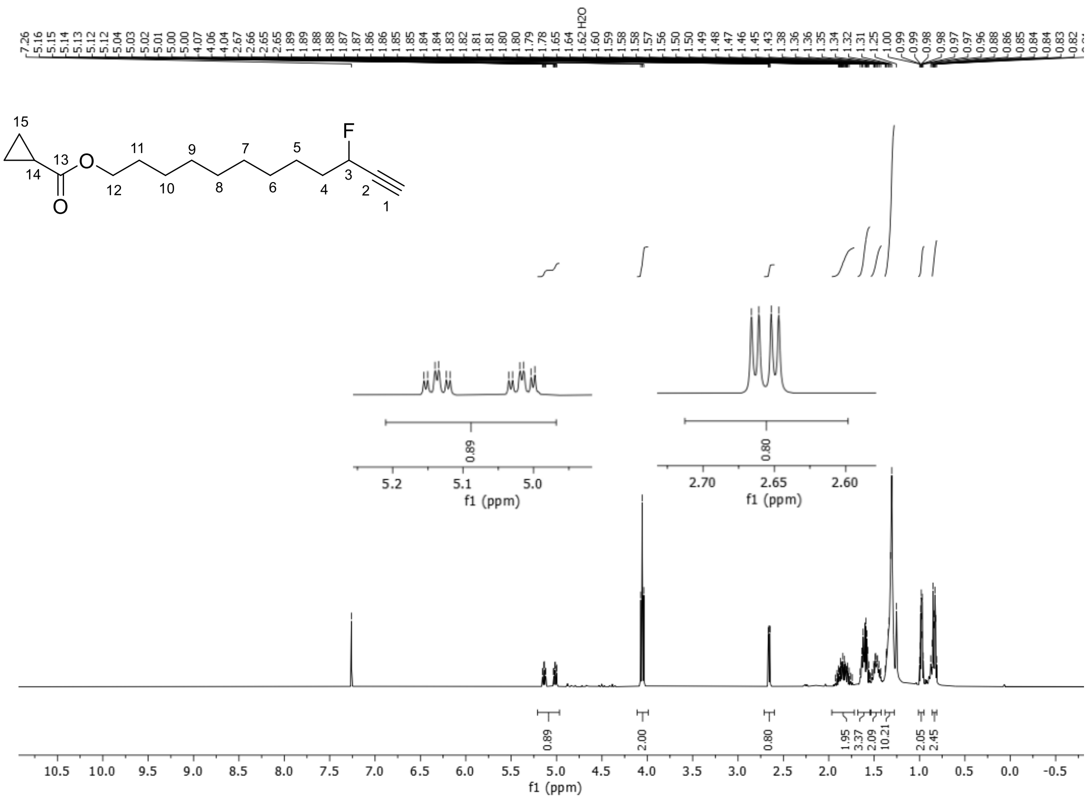

Supplementary Figure 95. ^{1}H NMR of **11** (400 MHz, 299 K, $CDCl_3$).

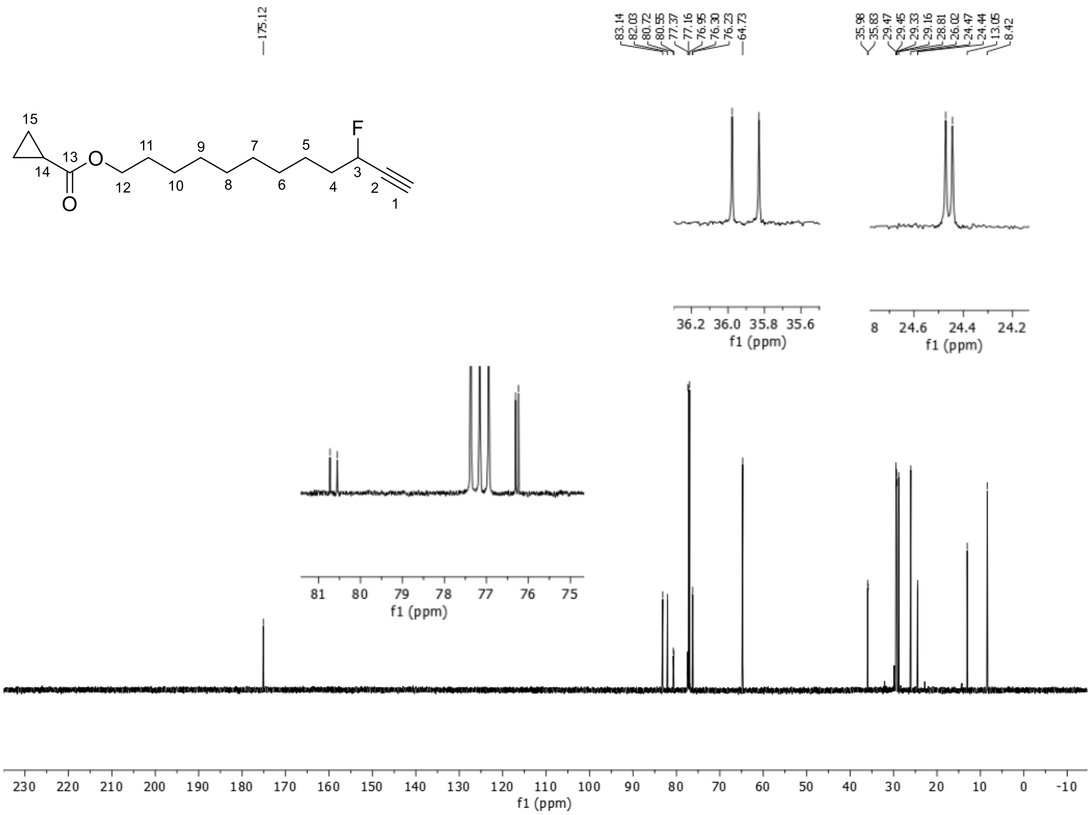

Supplementary Figure 96. $^{13}C\{^{1}H\}$ NMR of **11** (151 MHz, 299 K, $CDCl_3$).

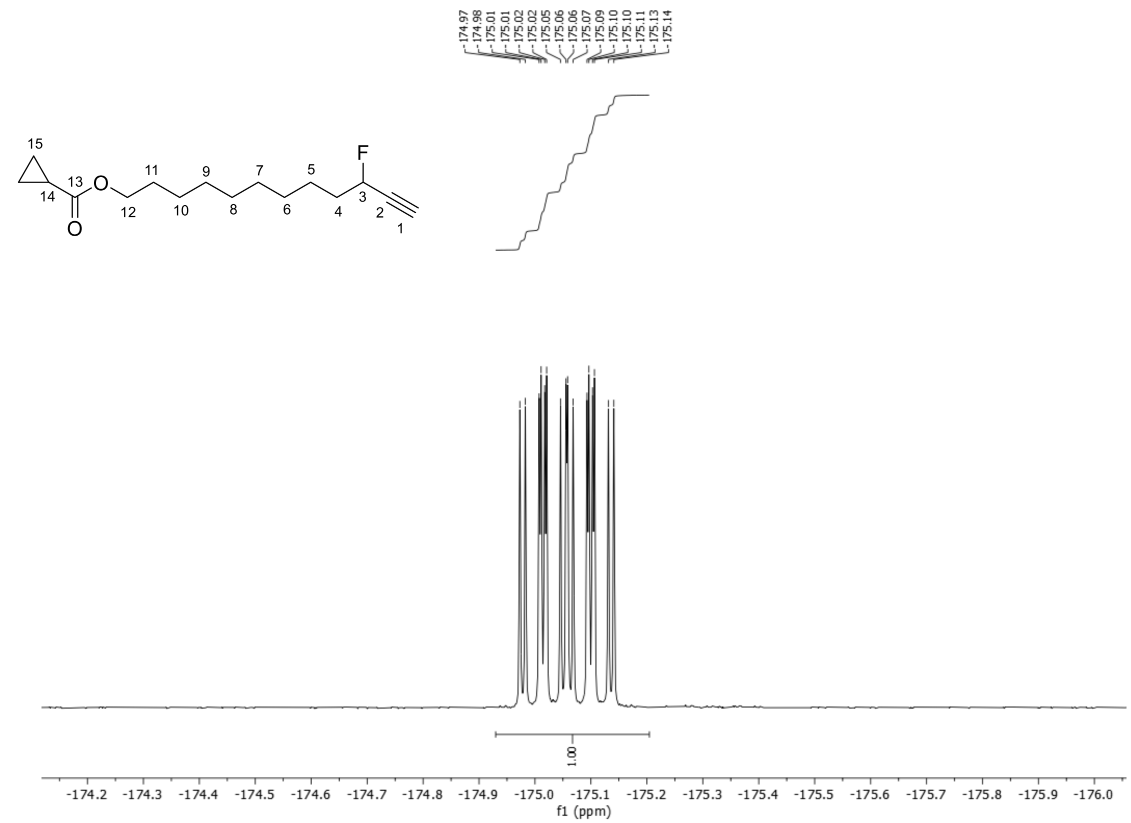

Supplementary Figure 97. ^{19}F NMR of **11** (564 MHz, 299 K, $CDCl_3$).

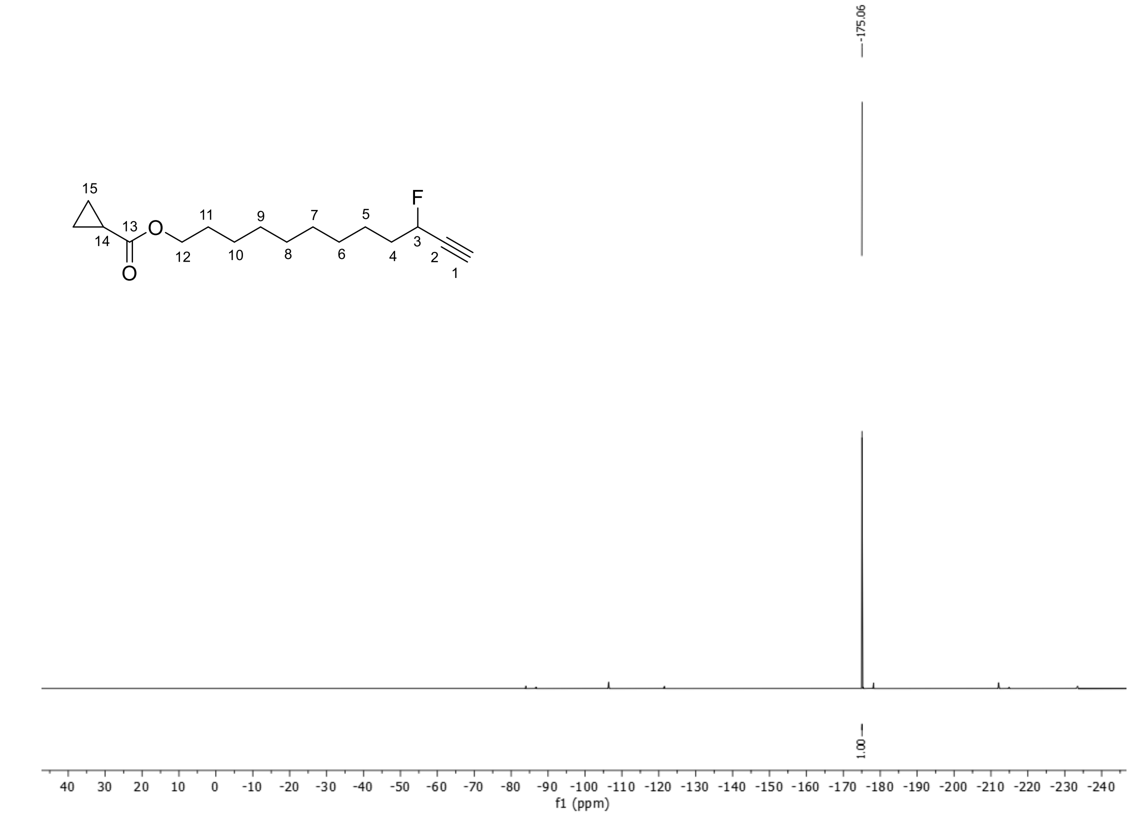

Supplementary Figure 98. ^{19}F{^{1}H} NMR of **11** (377 MHz, 299 K, $CDCl_3$).

(1-Fluoroprop-2-yn-1-yl)cyclohexane (12)

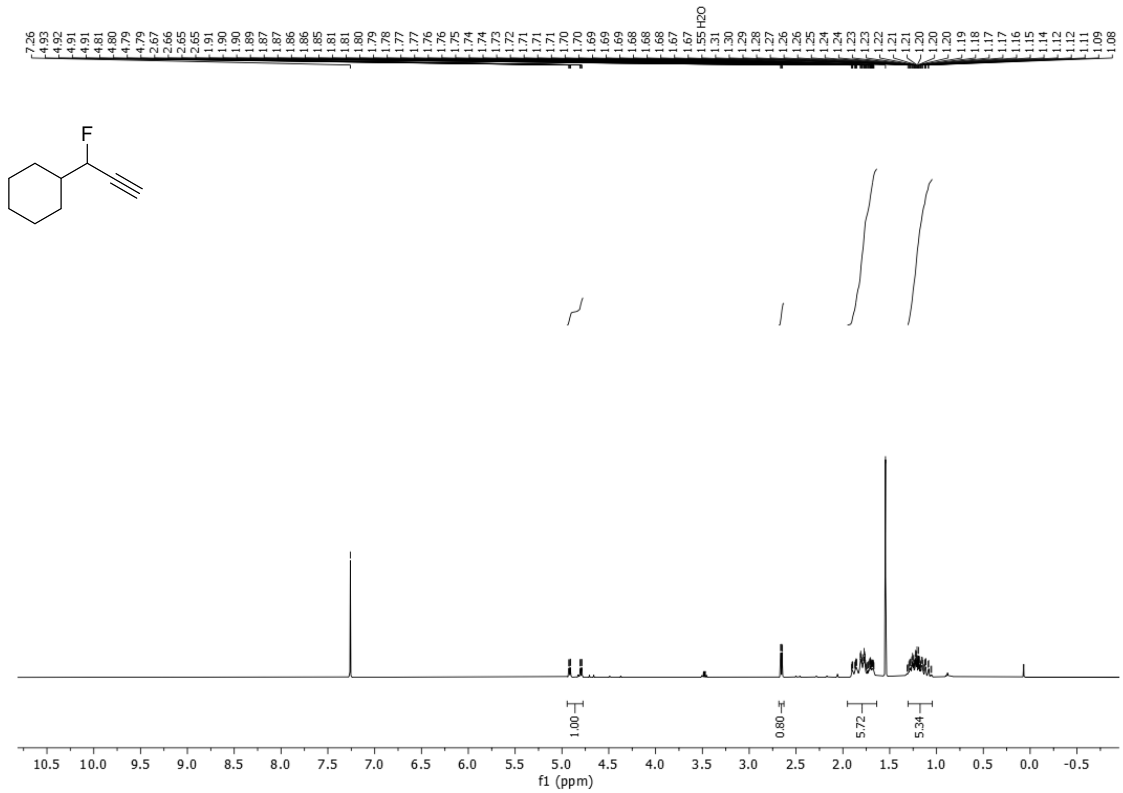

Supplementary Figure 99. ^{1}H NMR of **12** (400 MHz, 299 K, $CDCl_3$).

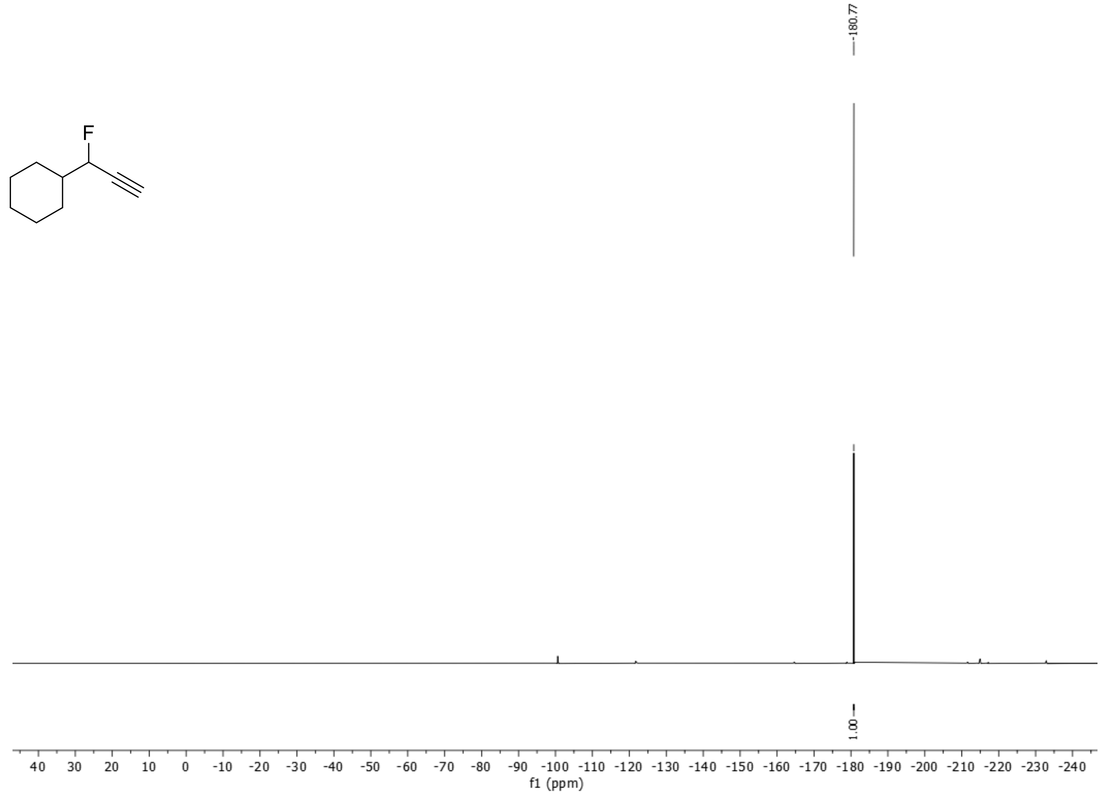

Supplementary Figure 100. $^{19}F\{^{1}H\}$ NMR of **12** (377 MHz, 299 K, $CDCl_3$).

(8R,9S,13S,14S)-13-Methyl-17-oxo-7,8,9,11,12,13,14,15,16,17-decahydro-6H-cyclopenta[a]phenanthren-3-yl 10-fluorododec-11-ynoate (13)

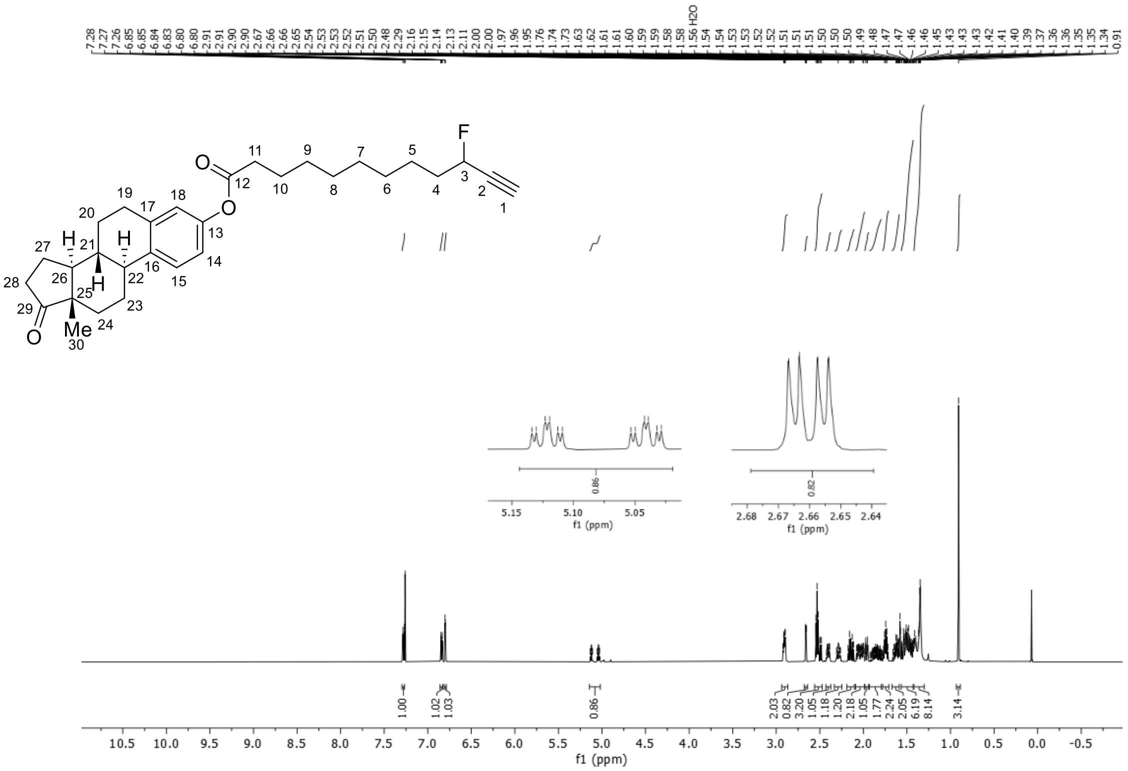

Supplementary Figure 101. ^{1}H NMR of **13** (599 MHz, 299 K, $CDCl_3$).

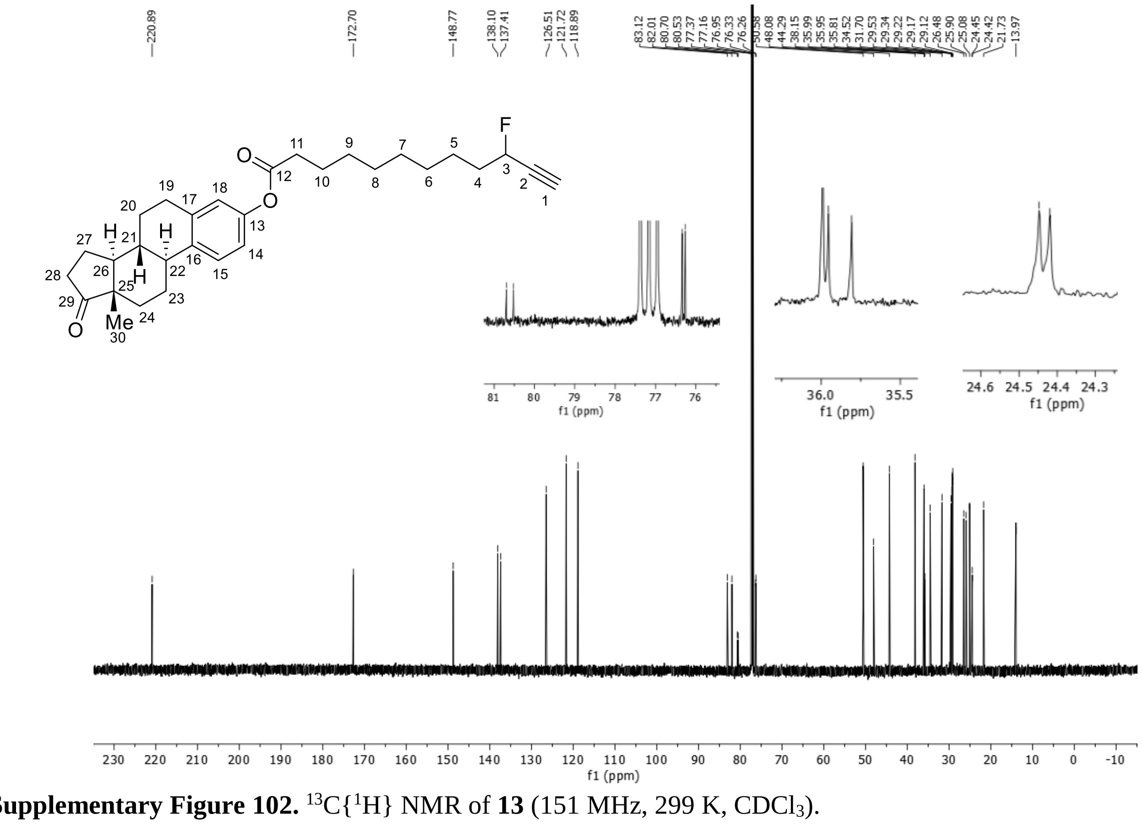

Supplementary Figure 102. $^{13}C\{^{1}H\}$ NMR of **13** (151 MHz, 299 K, $CDCl_3$).

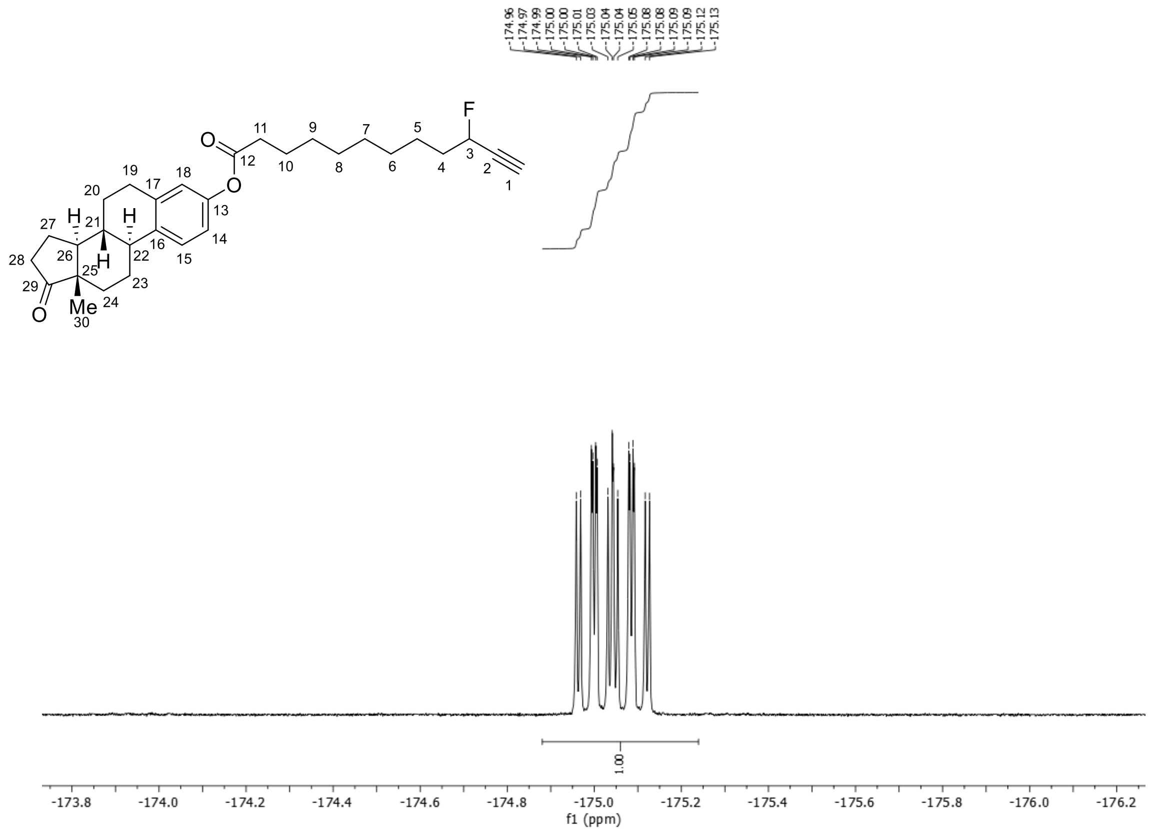

Supplementary Figure 103. ^{19}F NMR of **13** (564 MHz, 299 K, $CDCl_3$).

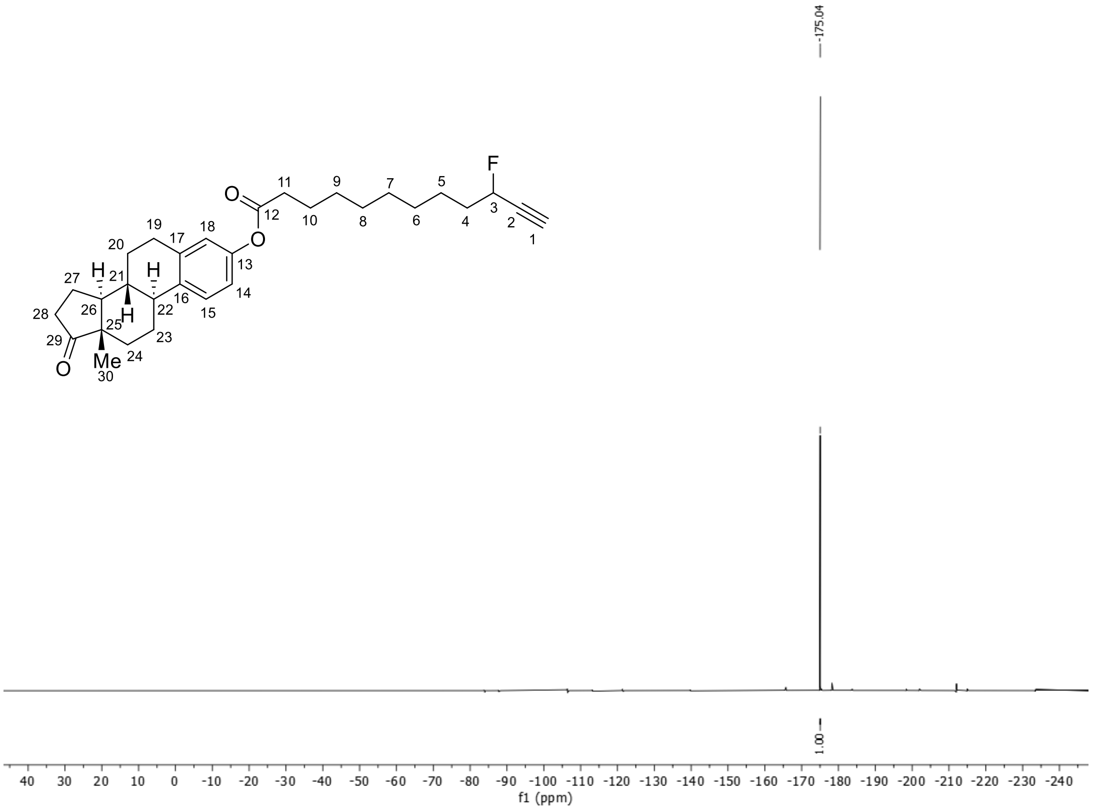

Supplementary Figure 104. $^{19}F\{^1H\}$ NMR of **13** (377 MHz, 299 K, $CDCl_3$).

10-Fluorododec-11-yn-1-yl 2-(4-isobutylphenyl)propanoate (14)

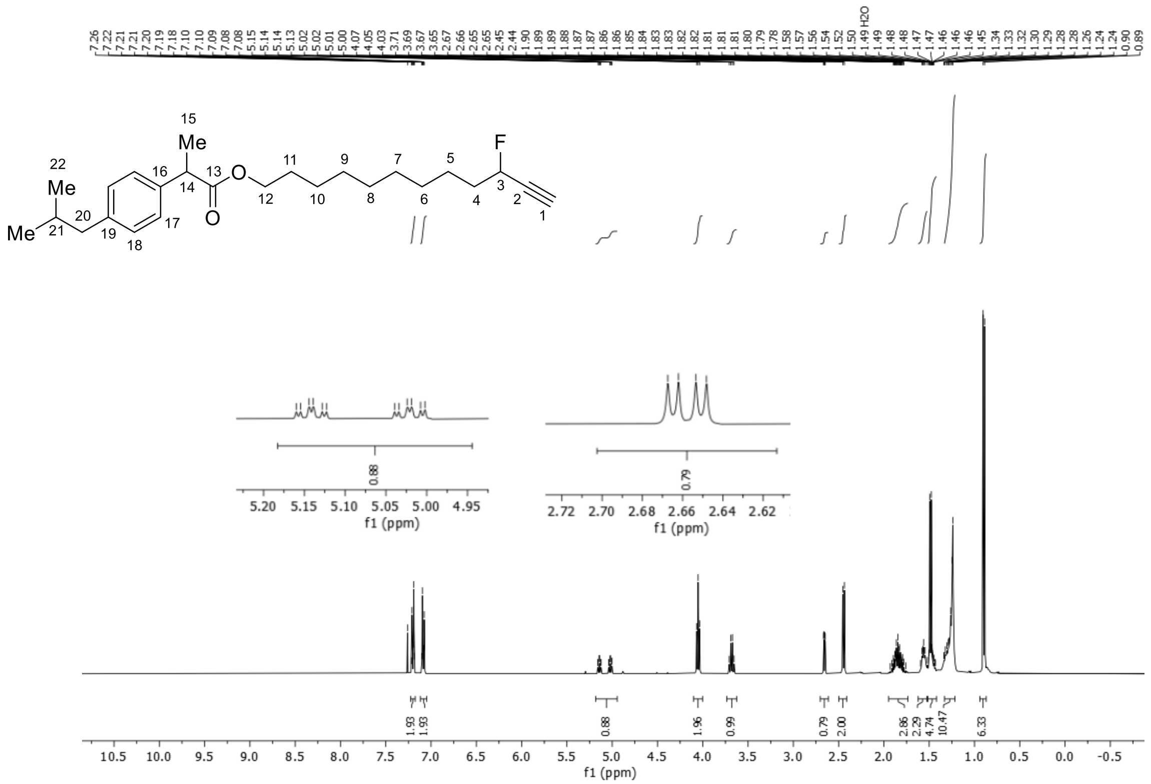

Supplementary Figure 105. ^{1}H NMR of **14** (400 MHz, 299 K, $CDCl_3$).

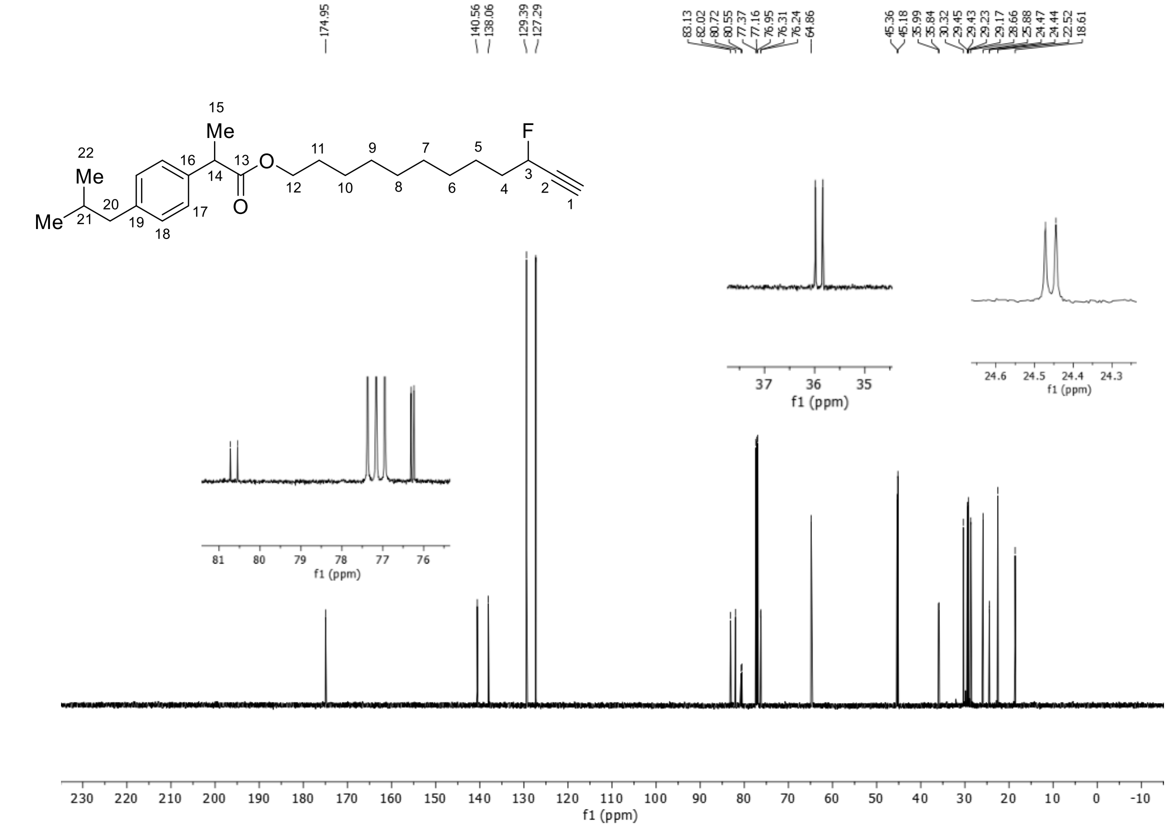

Supplementary Figure 106. $^{13}C\{^{1}H\}$ NMR of **14** (151 MHz, 299 K, $CDCl_3$).

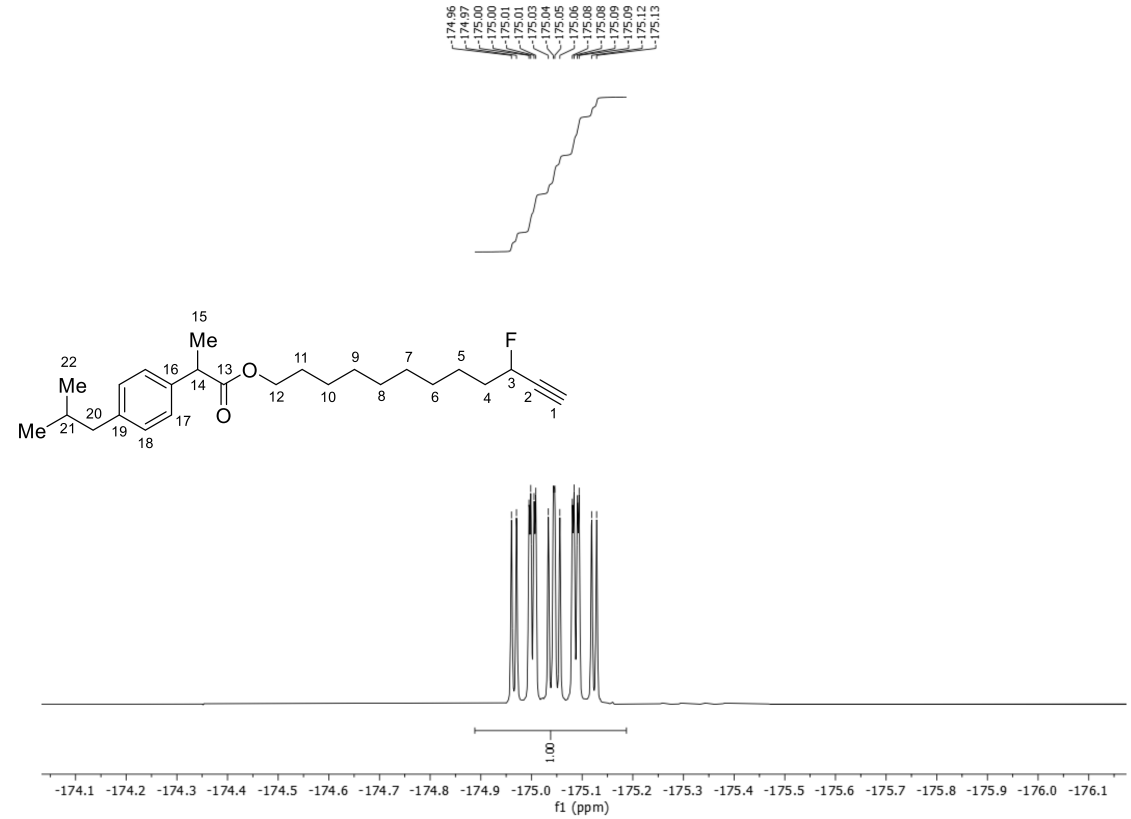

Supplementary Figure 107. ^{19}F NMR of **14** (564 MHz, 299 K, $CDCl_3$).

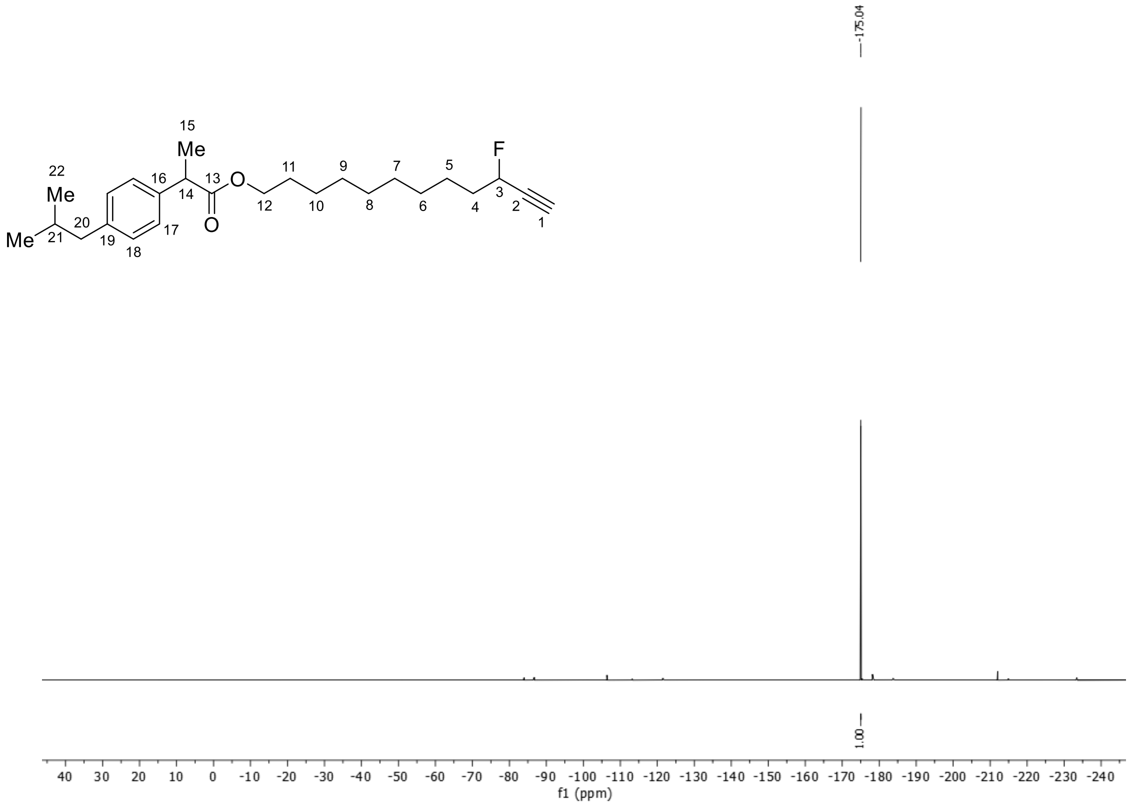

Supplementary Figure 108. ^{19}F{^{1}H} NMR of **14** (377 MHz, 299 K, $CDCl_3$).

10-Fluorododec-11-yn-1-yl (E)-3-(4-bromophenyl)acrylate (15)

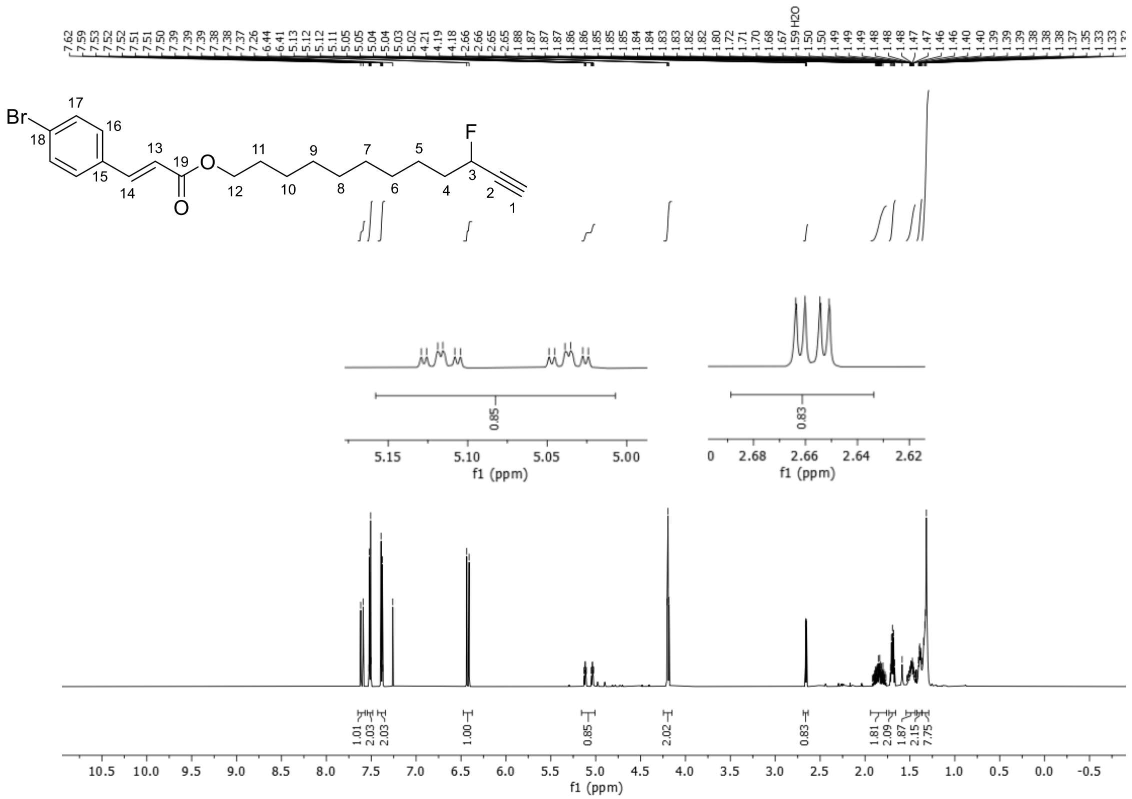

Supplementary Figure 109. ^{1}H NMR of **15** (599 MHz, 299 K, $CDCl_3$).

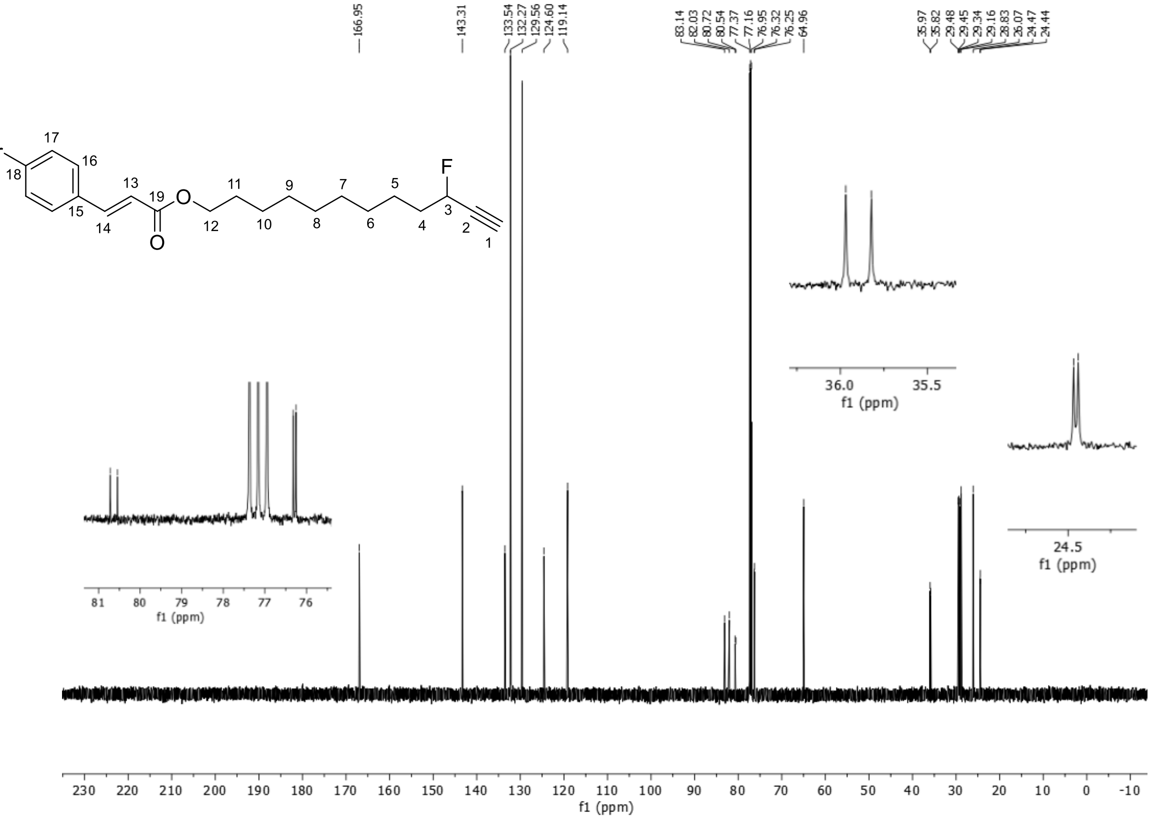

Supplementary Figure 110. $^{13}C\{^{1}H\}$ NMR of **15** (151 MHz, 299 K, $CDCl_3$).

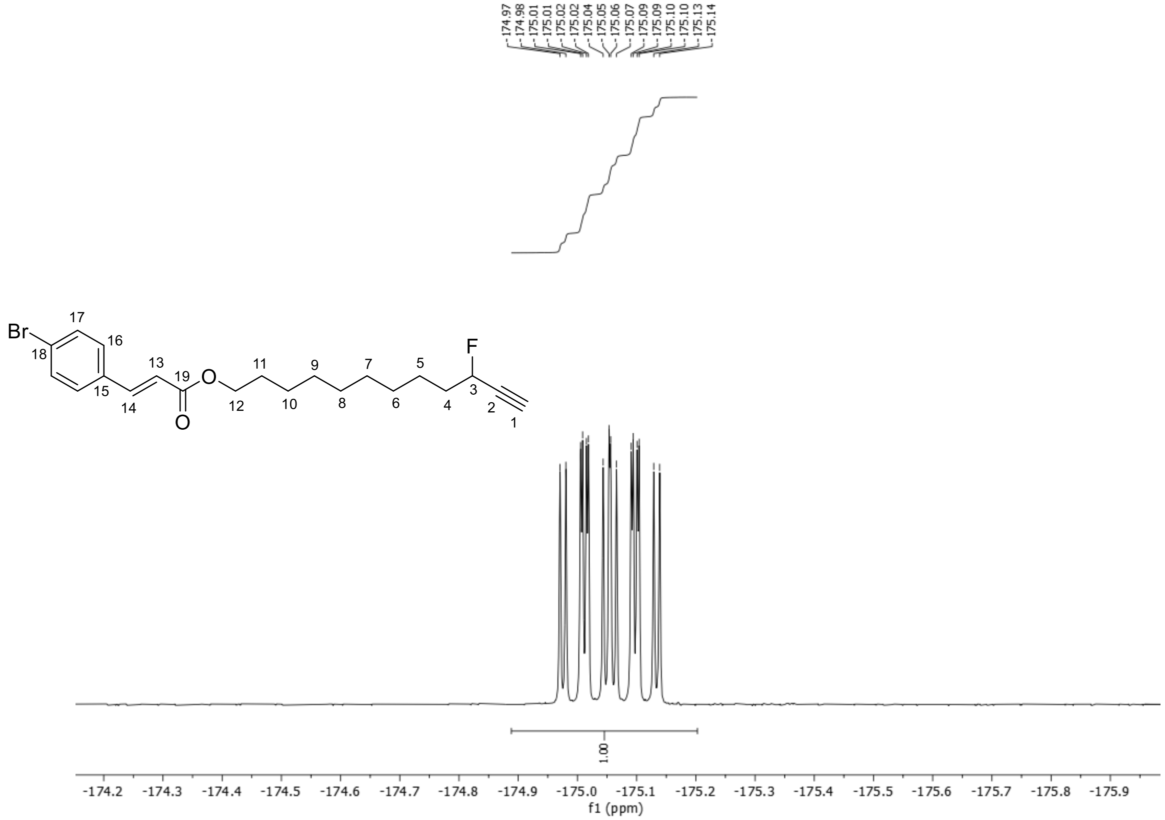

Supplementary Figure 111. ^{19}F NMR of **15** (564 MHz, 299 K, $CDCl_3$).

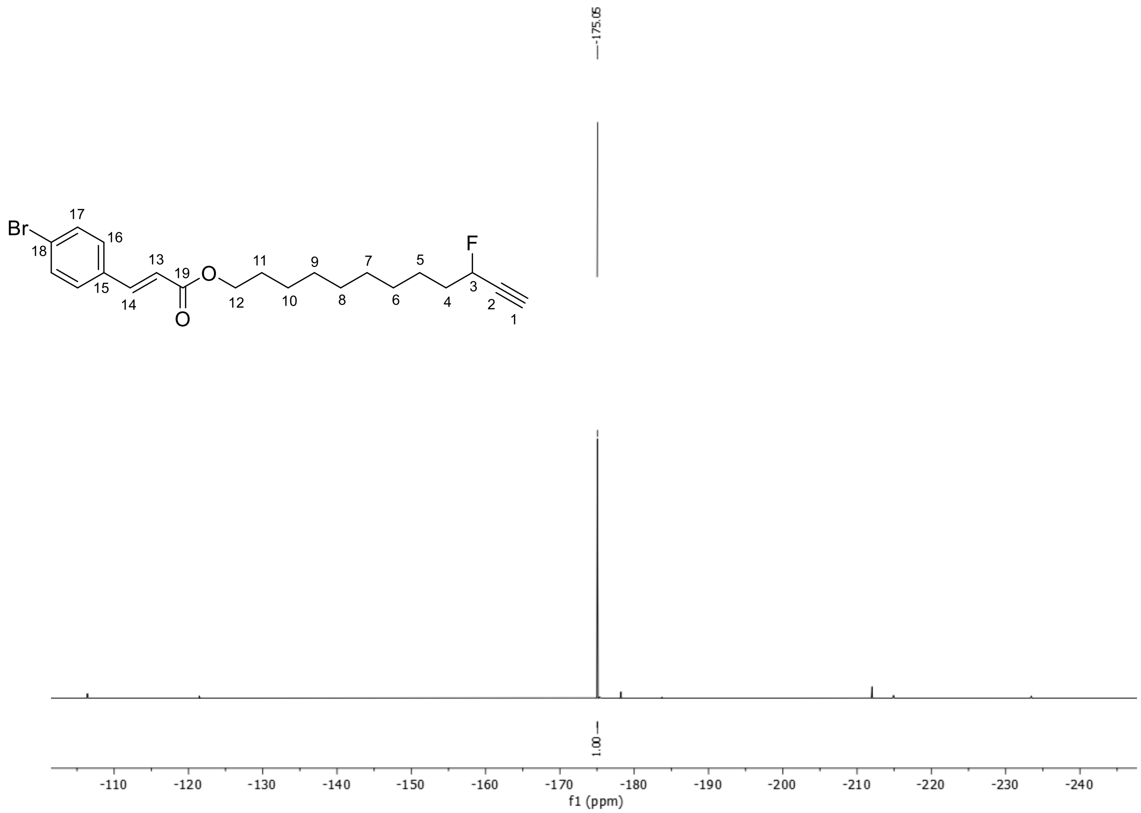

Supplementary Figure 112. $^{19}F\{^1H\}$ NMR of **15** (564 MHz, 299 K, $CDCl_3$).

1-(1-Fluoroprop-2-yn-1-yl)-4-(trifluoromethyl)benzene (16)

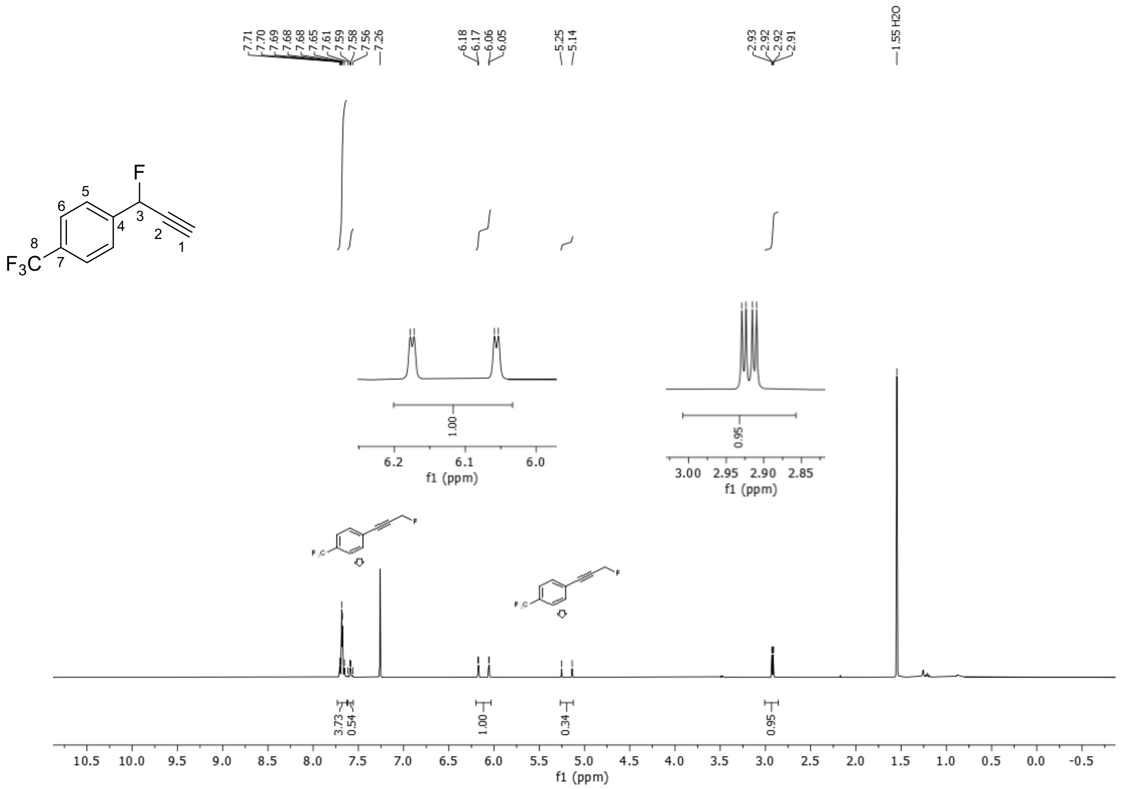

Supplementary Figure 113. ^{1}H NMR of **16** (400 MHz, 299 K, $CDCl_3$).

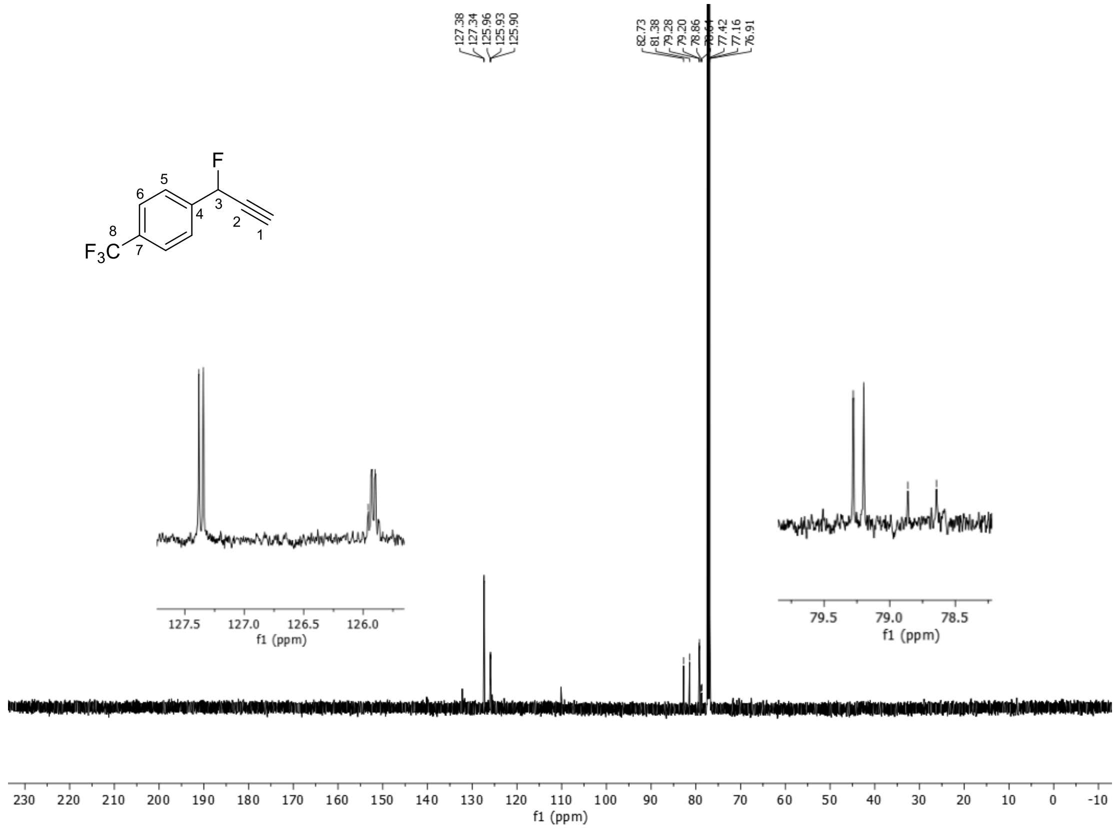

Supplementary Figure 114. $^{13}C\{^{1}H\}$ NMR of **16** (126 MHz, 299 K, $CDCl_3$).

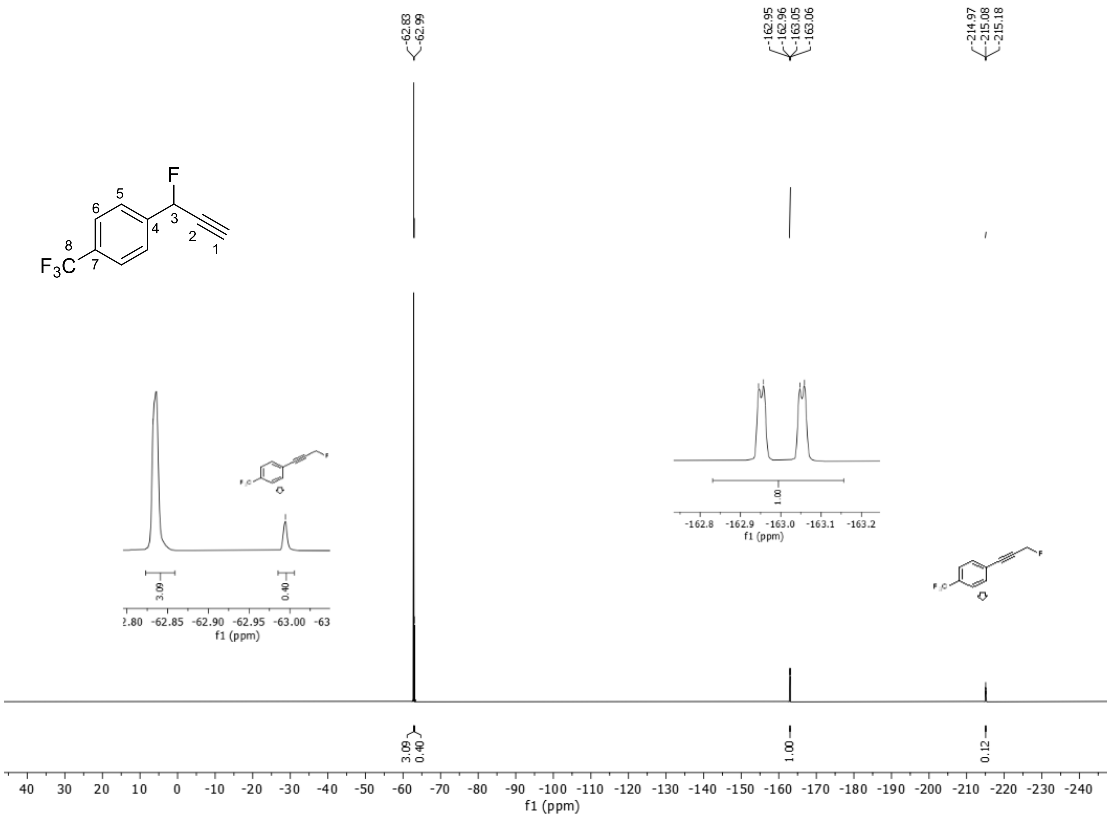

Supplementary Figure 115. ^{19}F NMR of **16** (470 MHz, 299 K, $CDCl_3$).

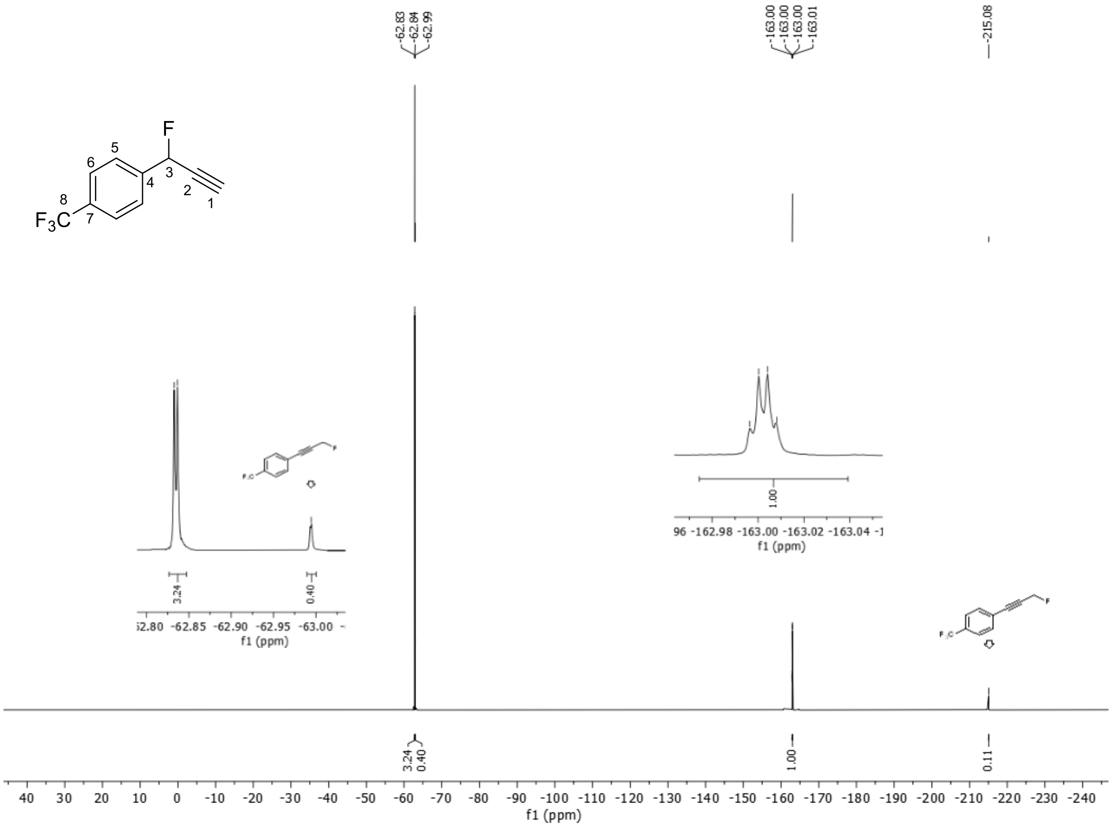

Supplementary Figure 116. $^{19}F\{^1H\}$ NMR of **16** (470 MHz, 299 K, $CDCl_3$).

1-(1-Fluoroprop-2-yn-1-yl)-3,5-bis(trifluoromethyl)benzene (17)

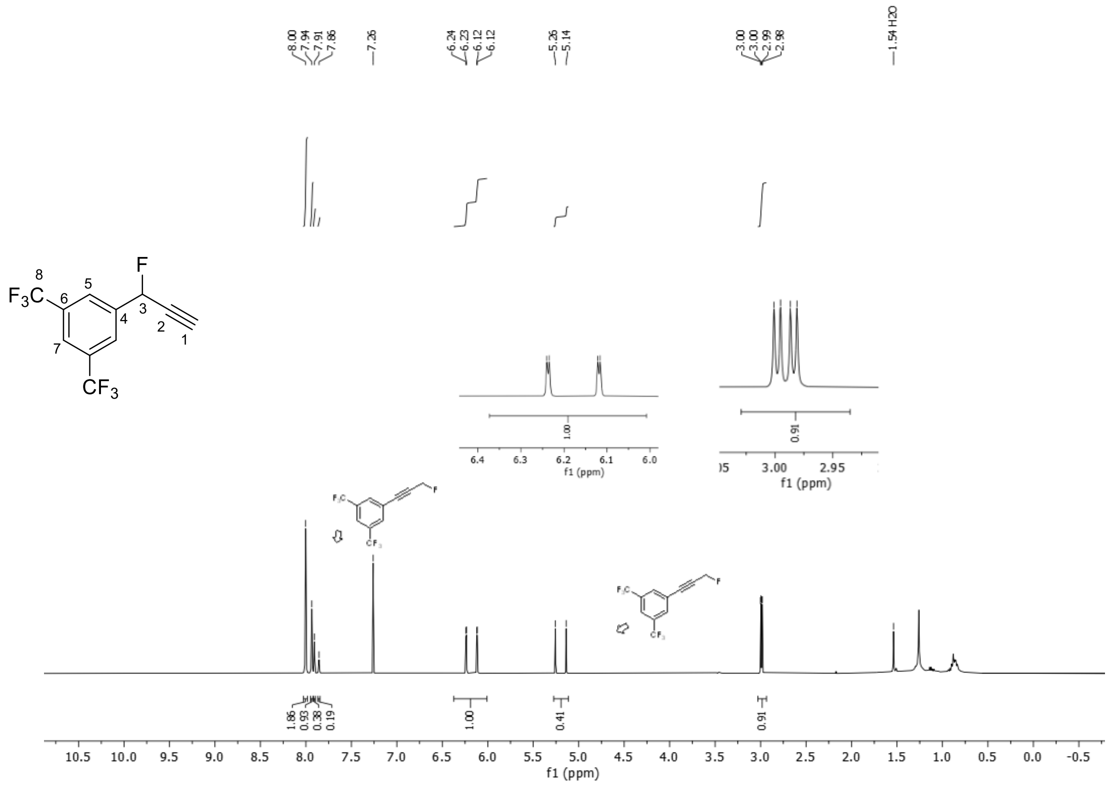

Supplementary Figure 117. ^{1}H NMR of **17** (400 MHz, 299 K, $CDCl_3$).

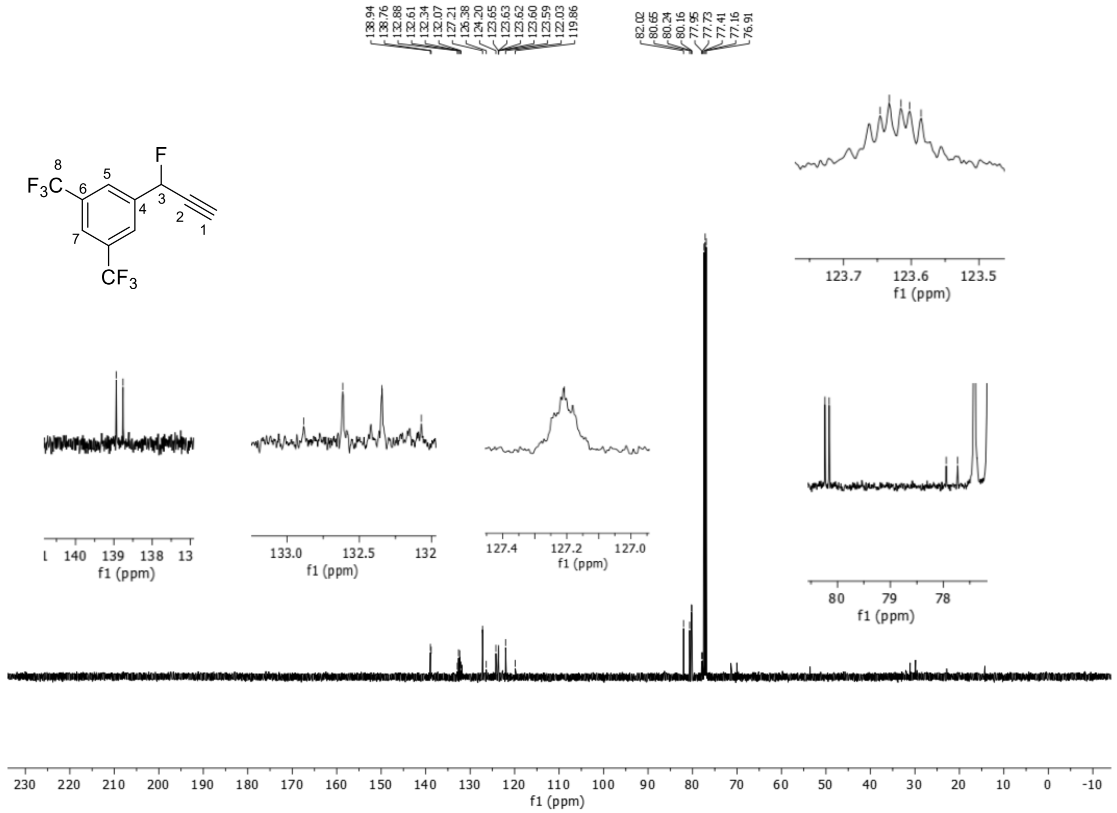

Supplementary Figure 118. $^{13}C\{^{1}H\}$ NMR of **17** (126 MHz, 299 K, $CDCl_3$).

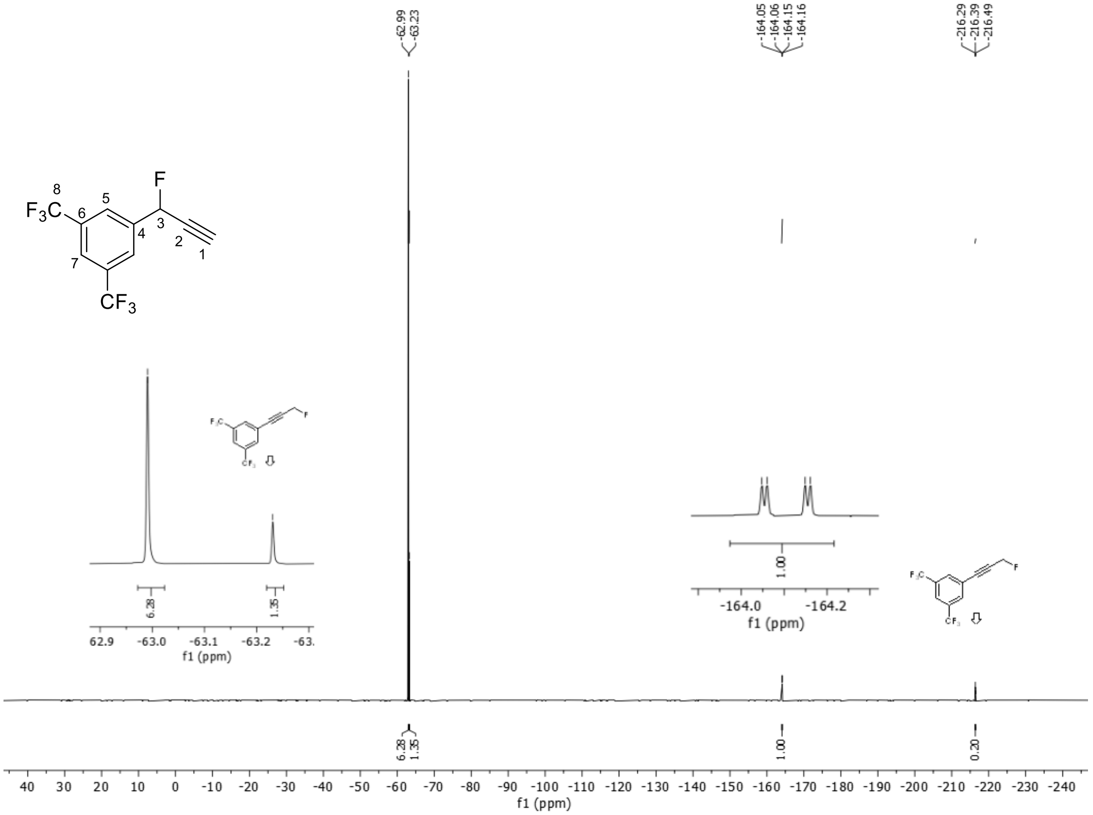

Supplementary Figure 119. ^{19}F NMR of **17** (470 MHz, 299 K, $CDCl_3$).

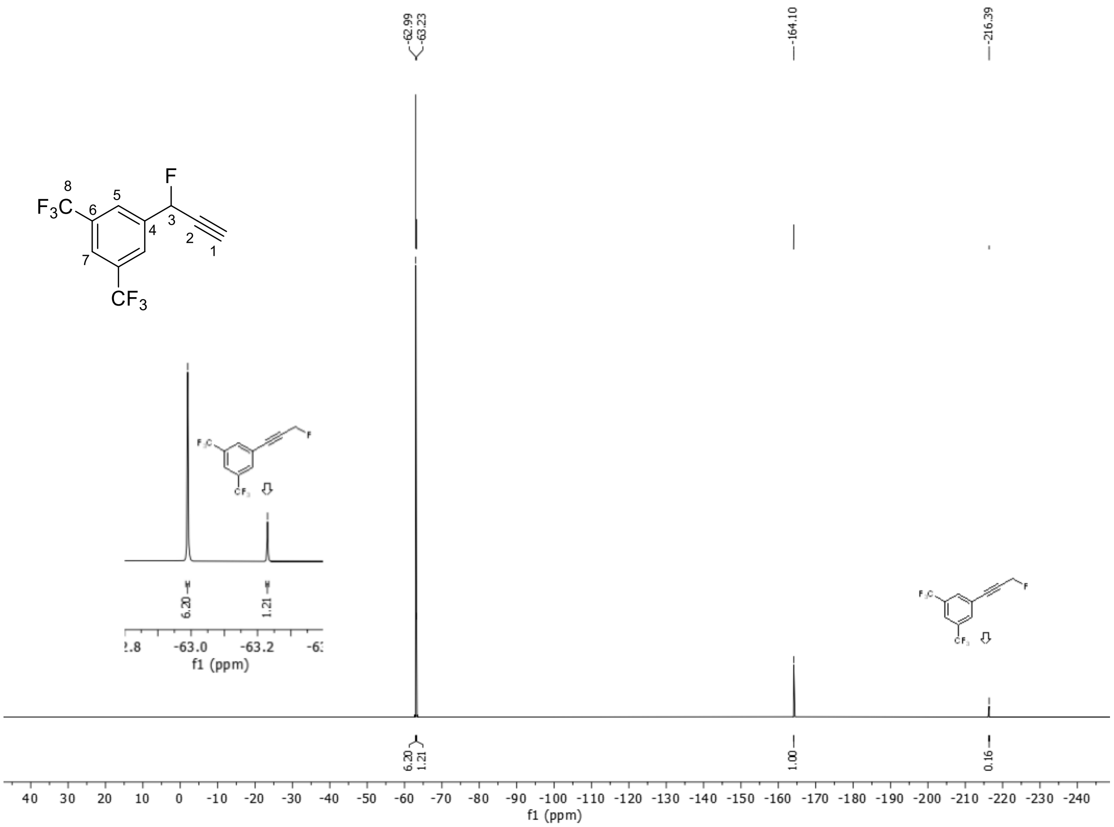

Supplementary Figure 120. $^{19}F\{^1H\}$ NMR of **17** (377 MHz, 299 K, $CDCl_3$).

Methyl 4-(1-fluoroprop-2-yn-1-yl)benzoate (18)

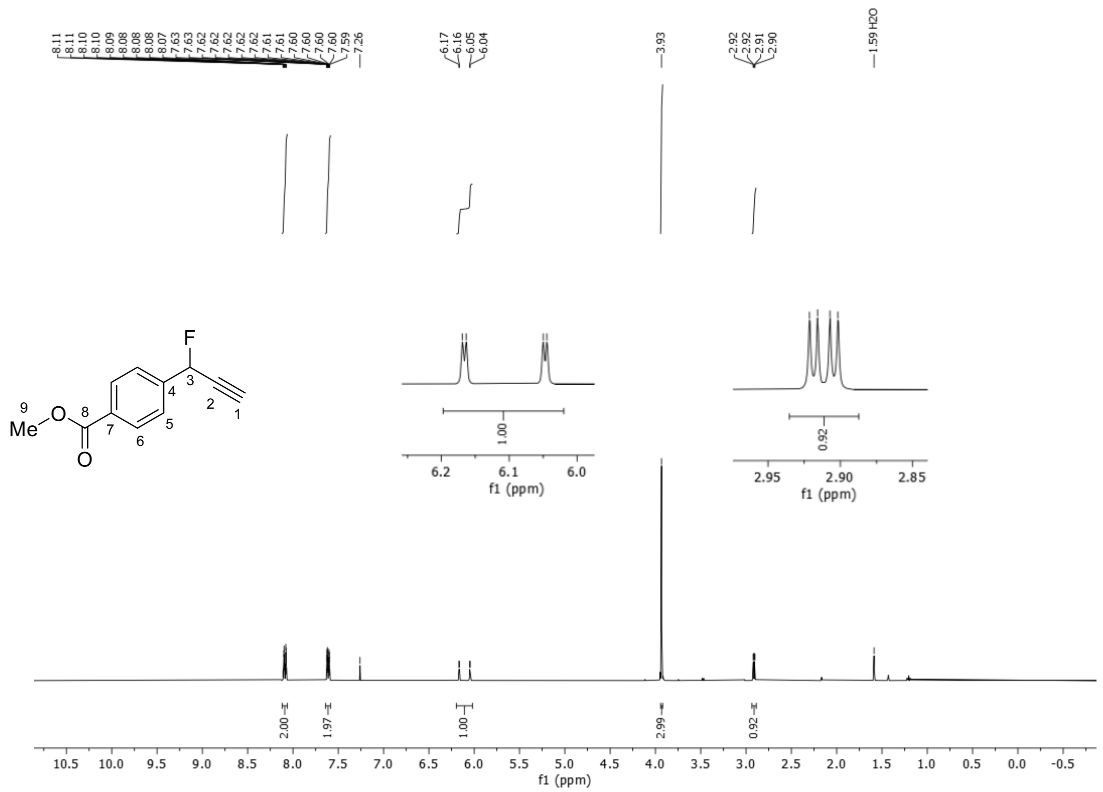

Supplementary Figure 121. ^{1}H NMR of **18** (400 MHz, 299 K, $CDCl_3$).

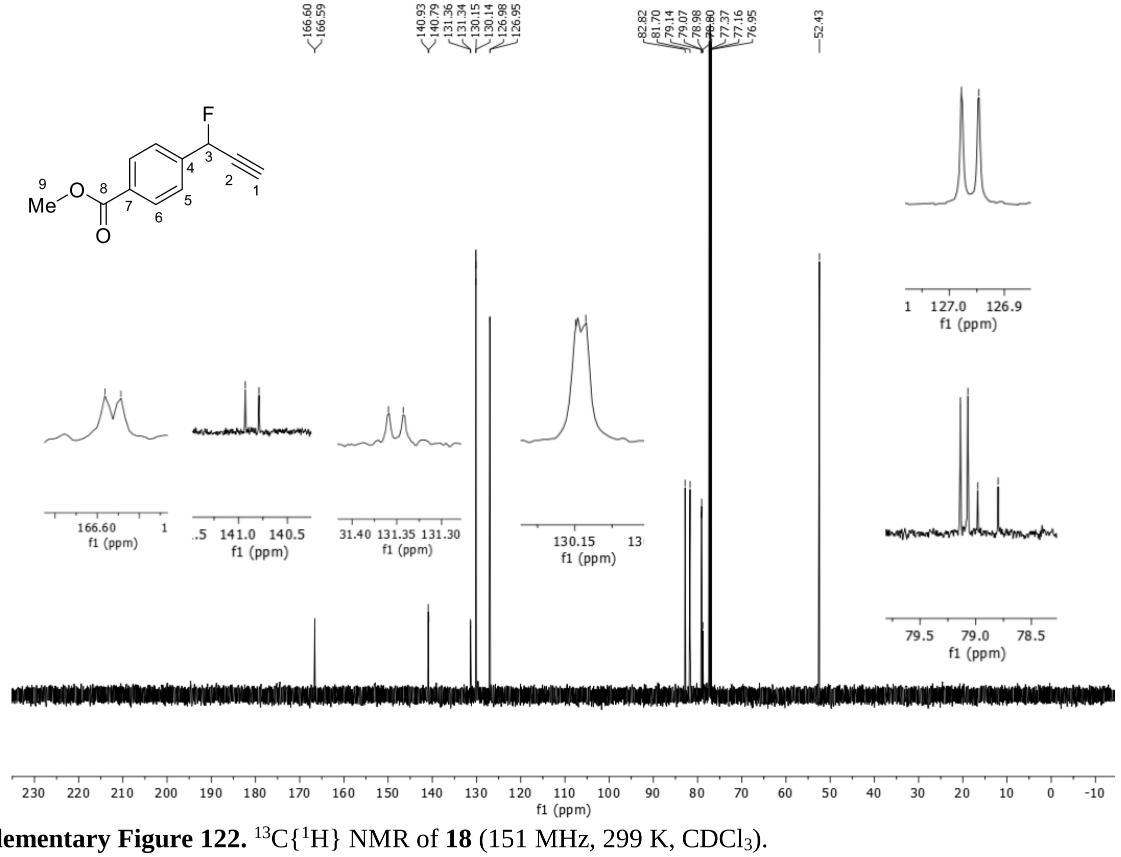

Supplementary Figure 122. $^{13}C\{^{1}H\}$ NMR of **18** (151 MHz, 299 K, $CDCl_3$).

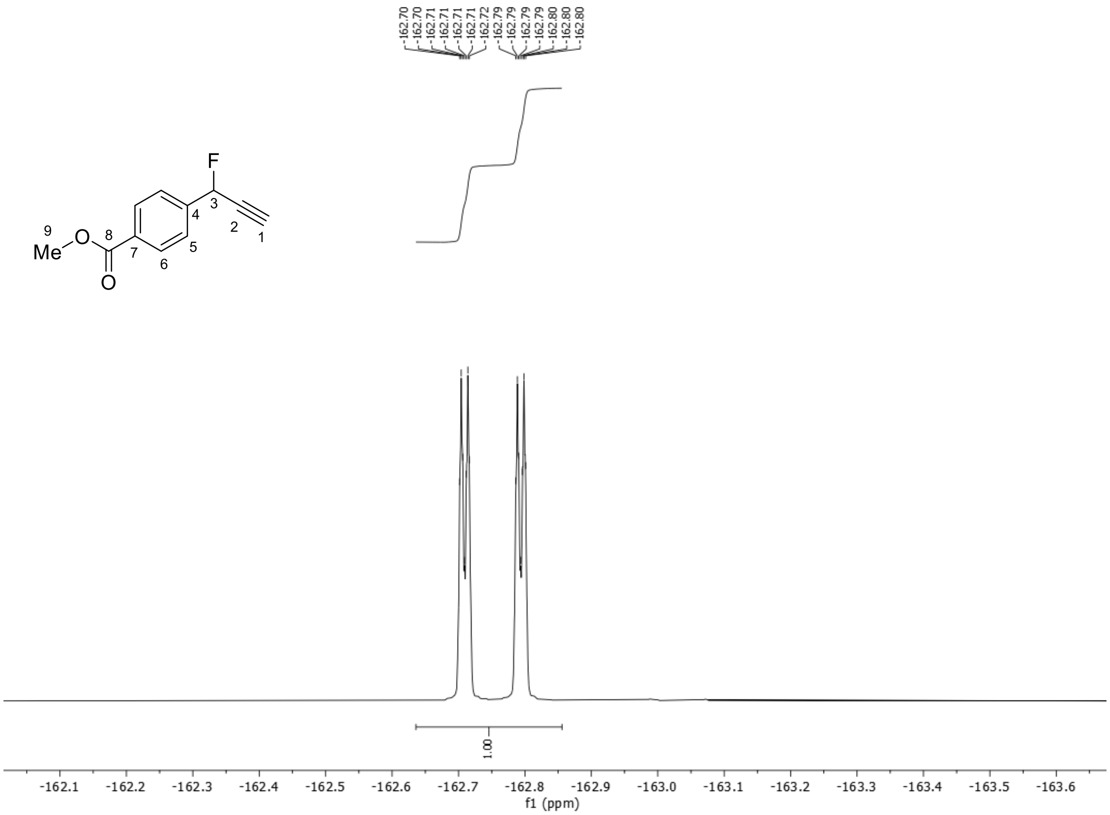

Supplementary Figure 123. ^{19}F NMR of **18** (564 MHz, 299 K, $CDCl_3$).

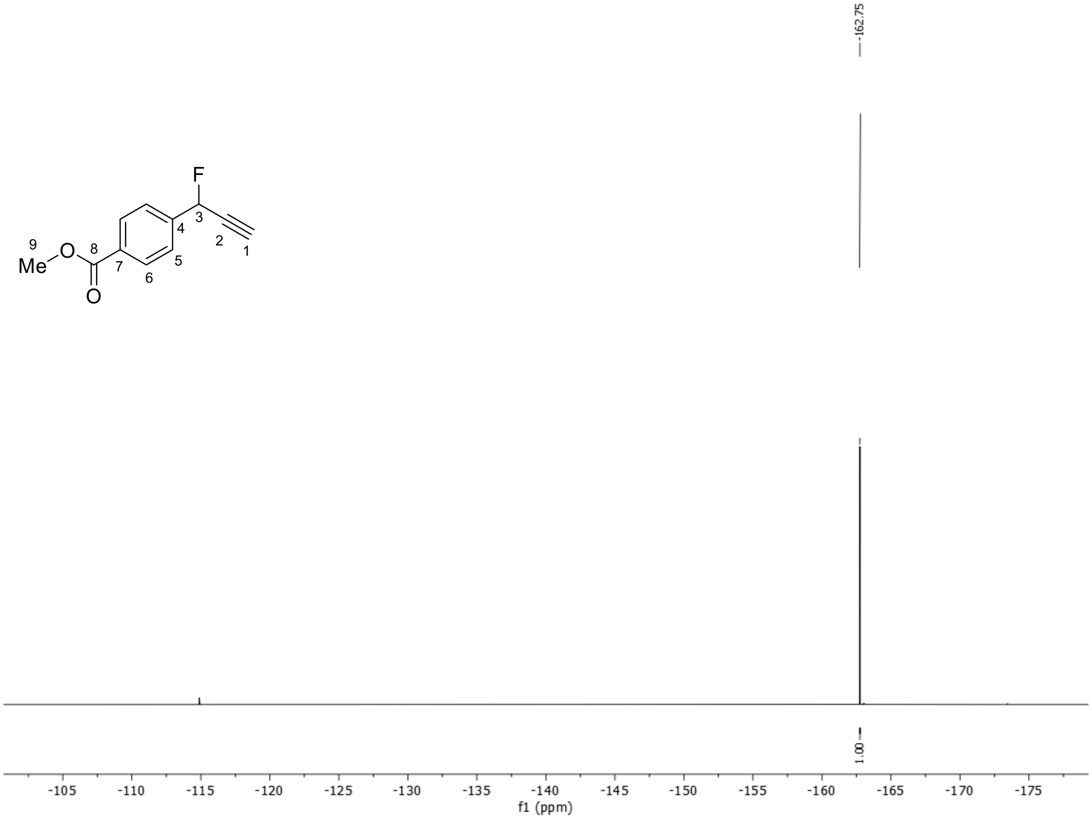

Supplementary Figure 124. ^{19}F{^{1}H} NMR of **18** (564 MHz, 299 K, $CDCl_3$).

(4-(1-Fluoroprop-2-yn-1-yl)phenyl)(morpholino)methanone (19)

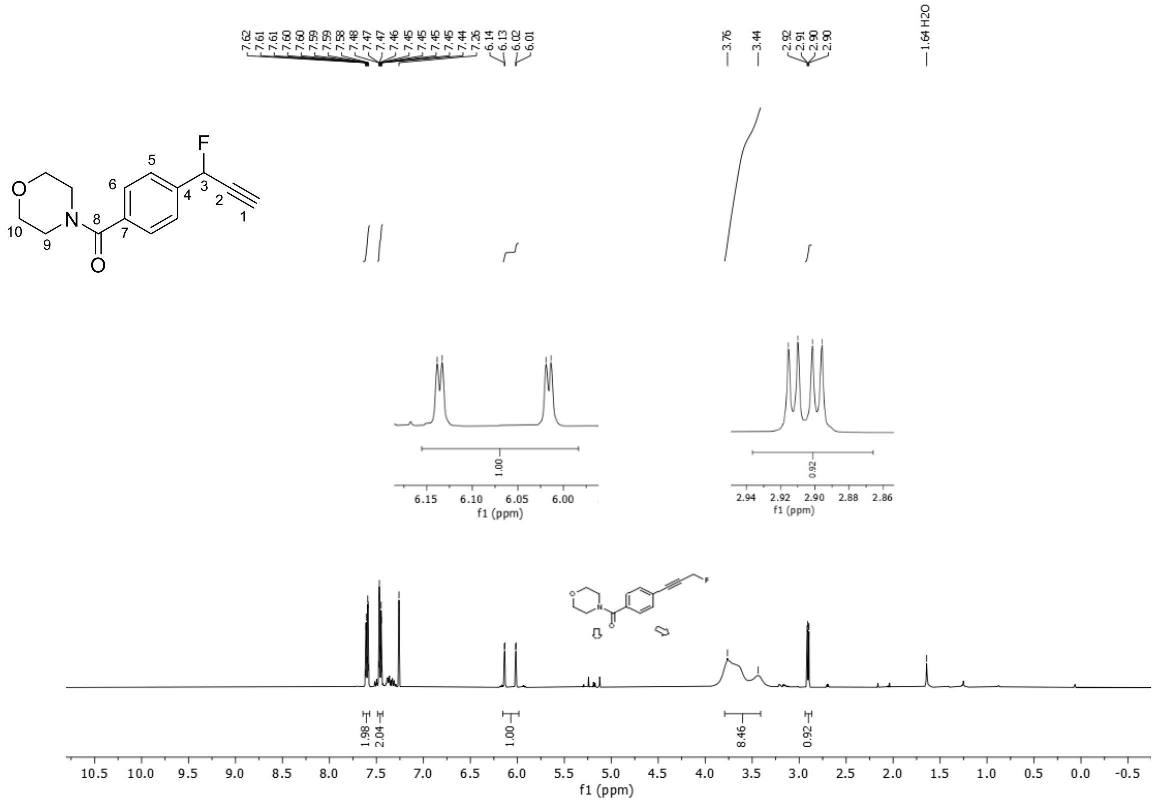

Supplementary Figure 125. ^{1}H NMR of **19** (400 MHz, 299 K, $CDCl_3$).

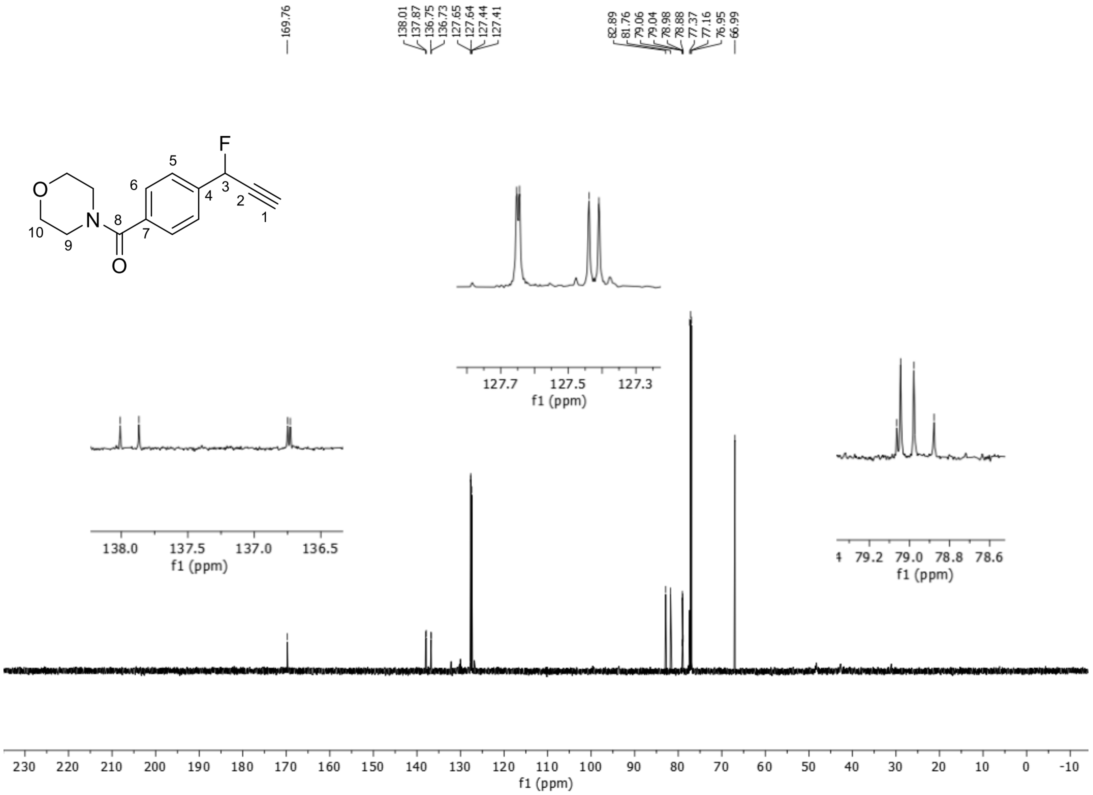

Supplementary Figure 126. $^{13}C\{^{1}H\}$ NMR of **19** (151 MHz, 299 K, $CDCl_3$).

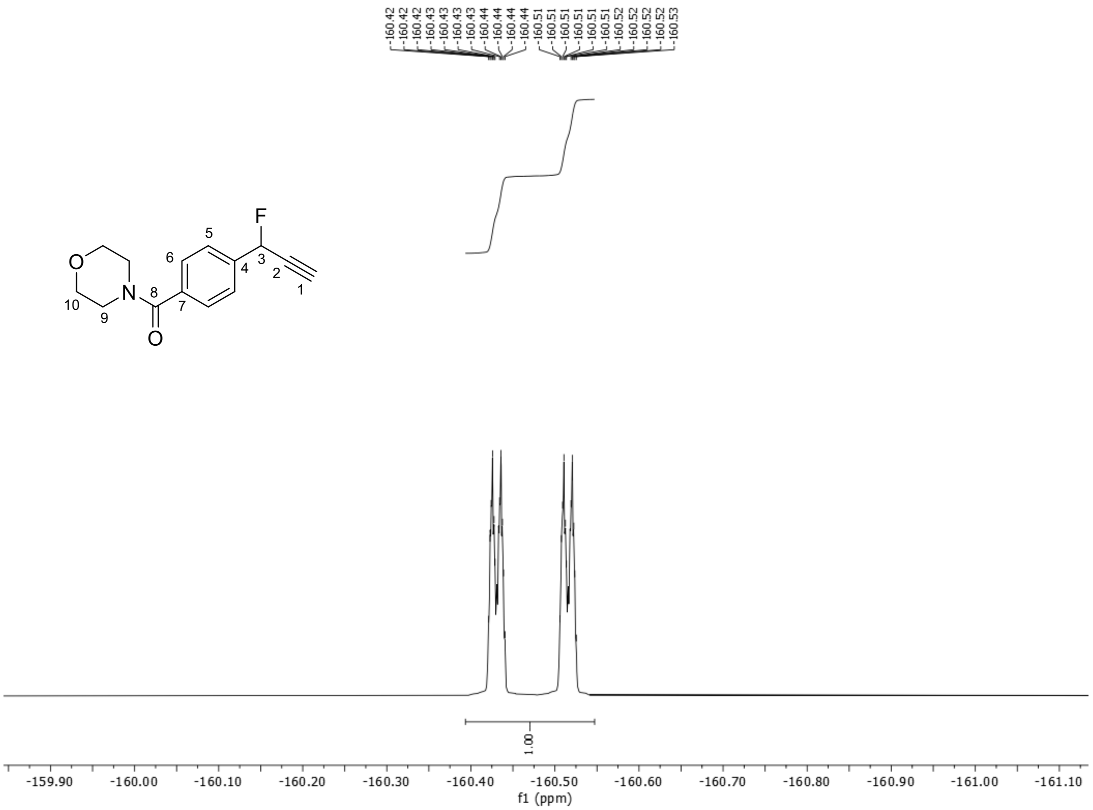

Supplementary Figure 127. ^{19}F NMR of **19** (564 MHz, 299 K, $CDCl_3$).

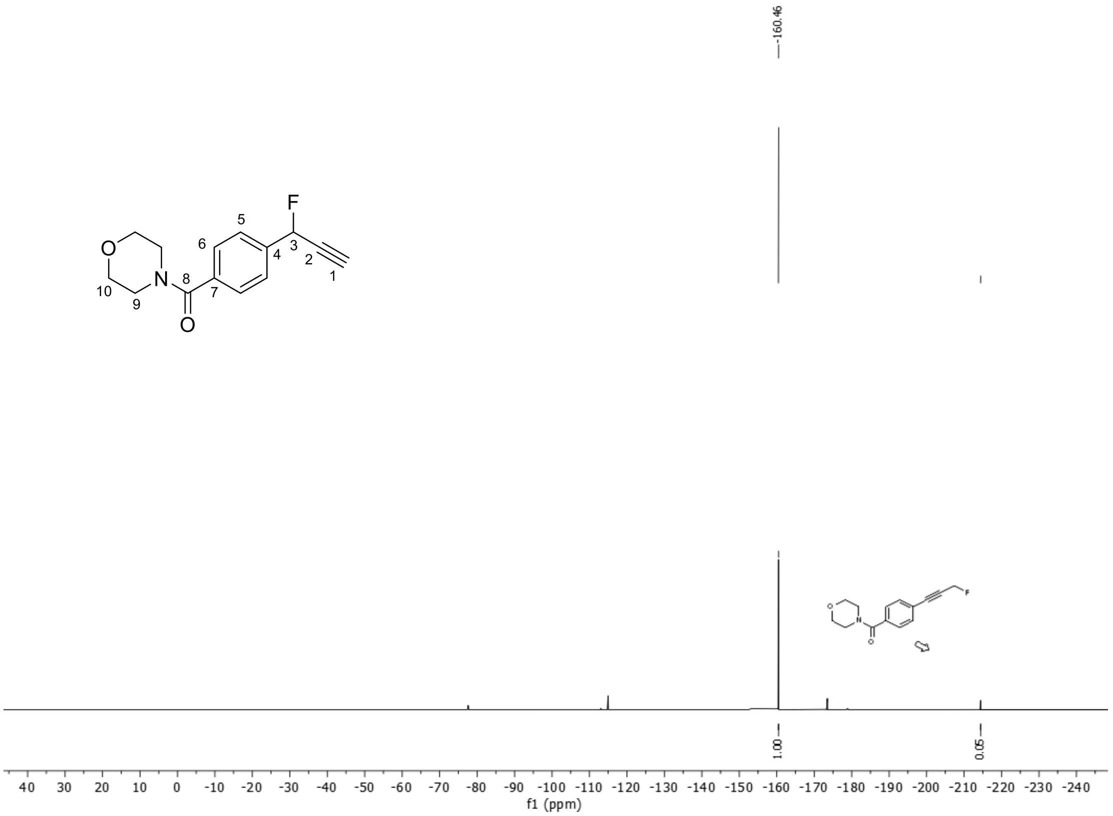

Supplementary Figure 128. $^{19}F\{^1H\}$ NMR of **19** (377 MHz, 299 K, $CDCl_3$).

3-(1-Fluoroprop-2-yn-1-yl)phenyl trifluoromethanesulfonate (20)

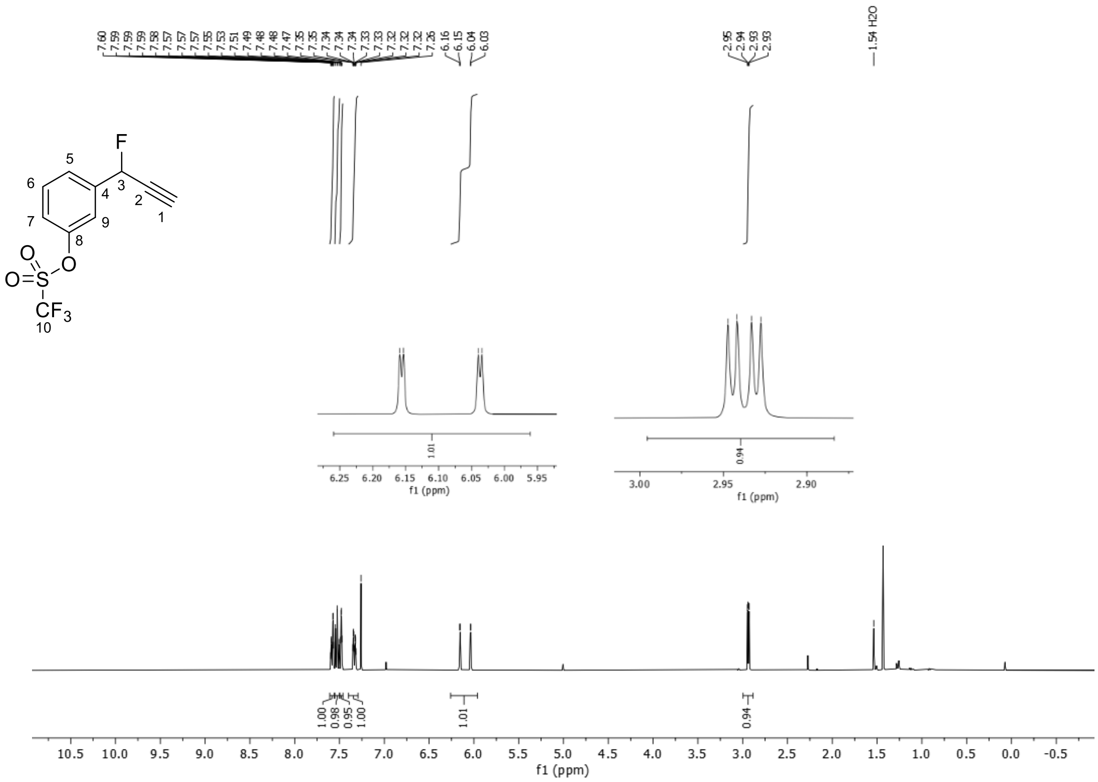

Supplementary Figure 129. ^{1}H NMR of **20** (400 MHz, 299 K, $CDCl_3$).

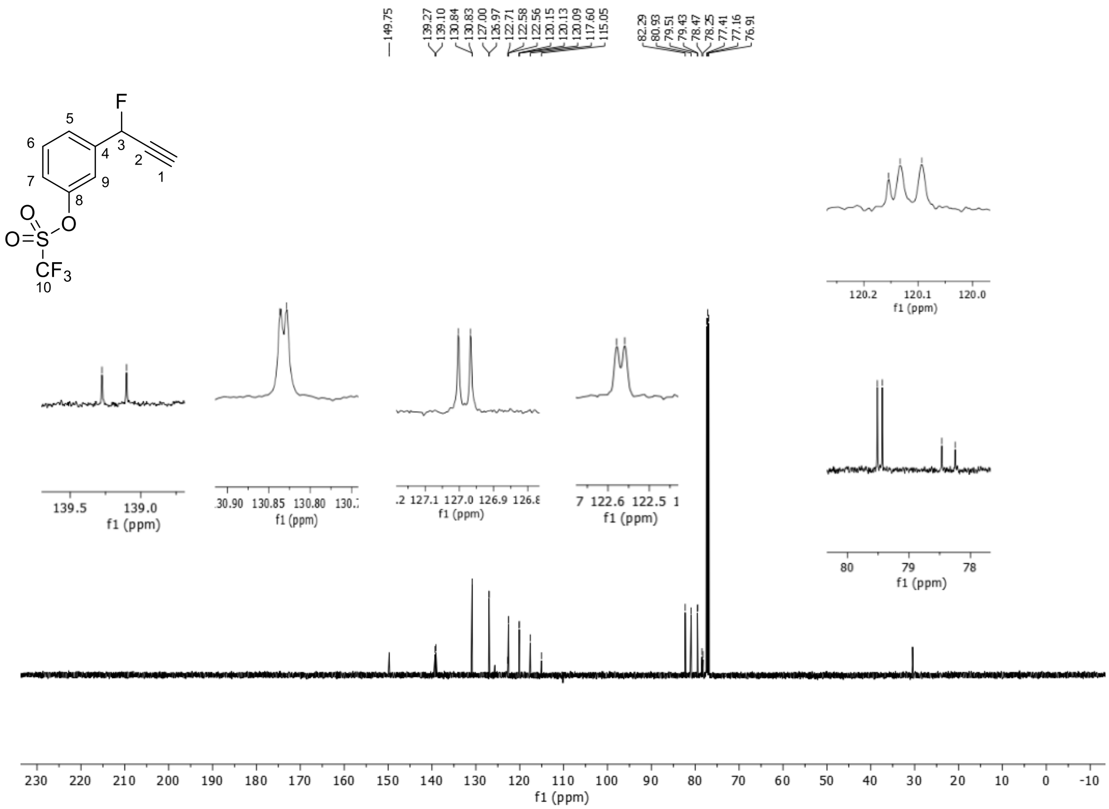

Supplementary Figure 130. $^{13}C\{^{1}H\}$ NMR of **20** (126 MHz, 299 K, $CDCl_3$).

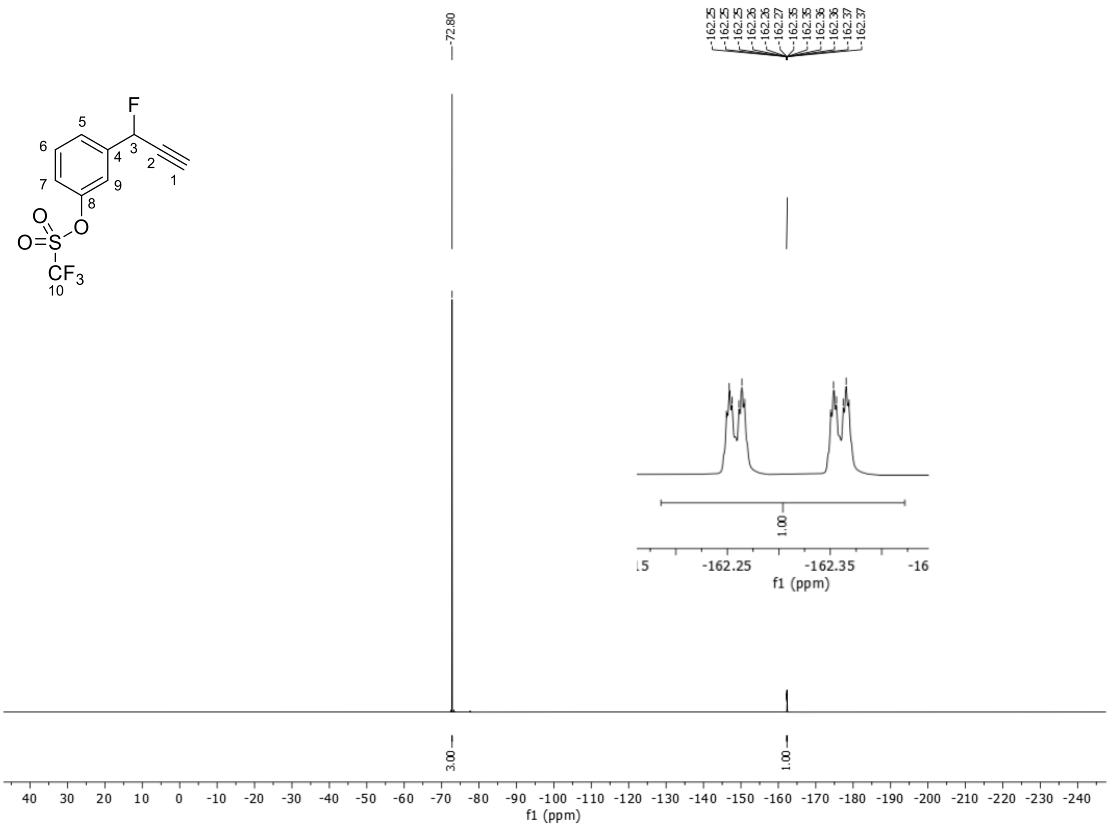

Supplementary Figure 131. ^{19}F NMR of **20** (470 MHz, 299 K, $CDCl_3$).

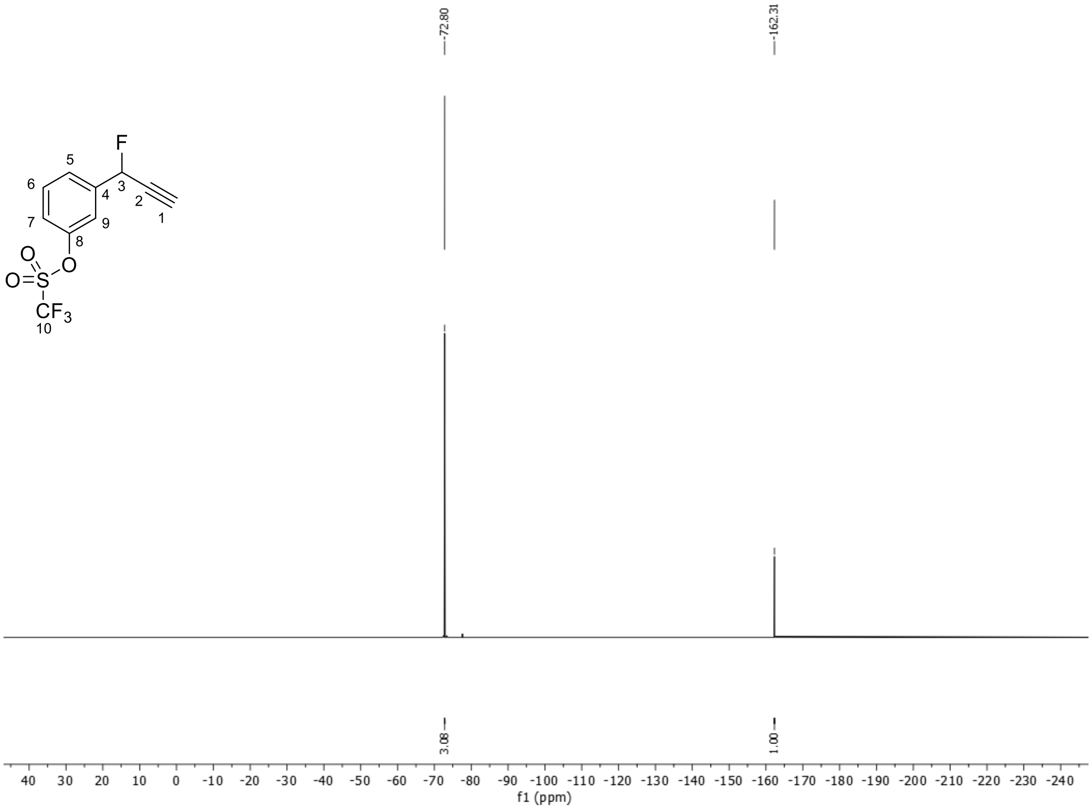

Supplementary Figure 132. $^{19}F\{^1H\}$ NMR of **20** (377 MHz, 299 K, $CDCl_3$).

1-(1-Fluoroprop-2-yn-1-yl)-4-(methylsulfonyl)benzene (21)

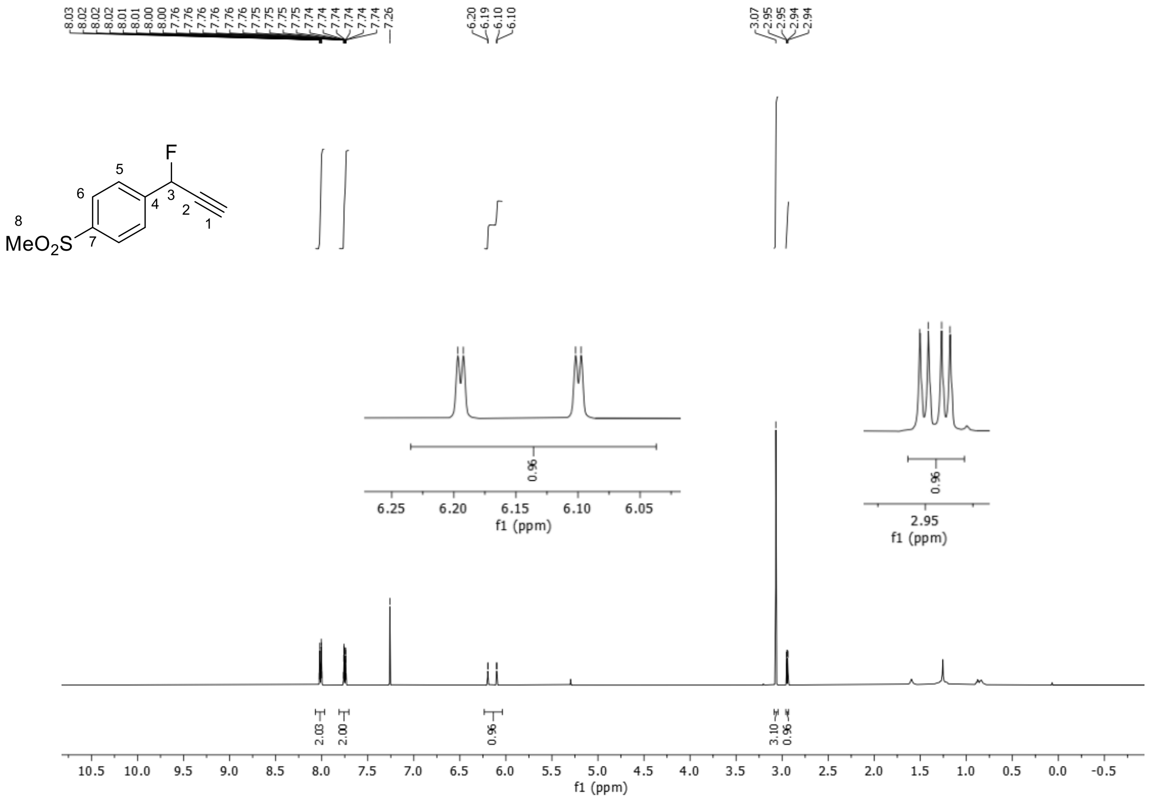

Supplementary Figure 133. ^{1}H NMR of **21** (500 MHz, 299 K, $CDCl_3$).

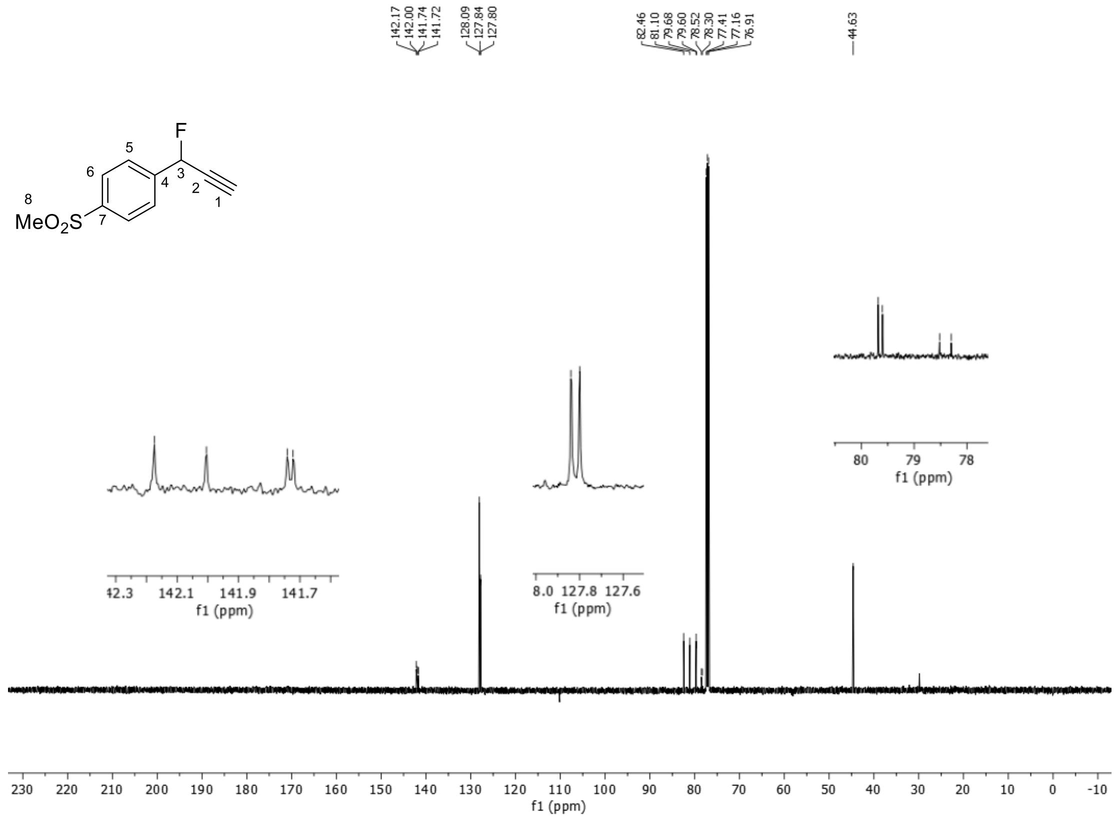

Supplementary Figure 134. $^{13}C\{^{1}H\}$ NMR of **21** (126 MHz, 299 K, $CDCl_3$).

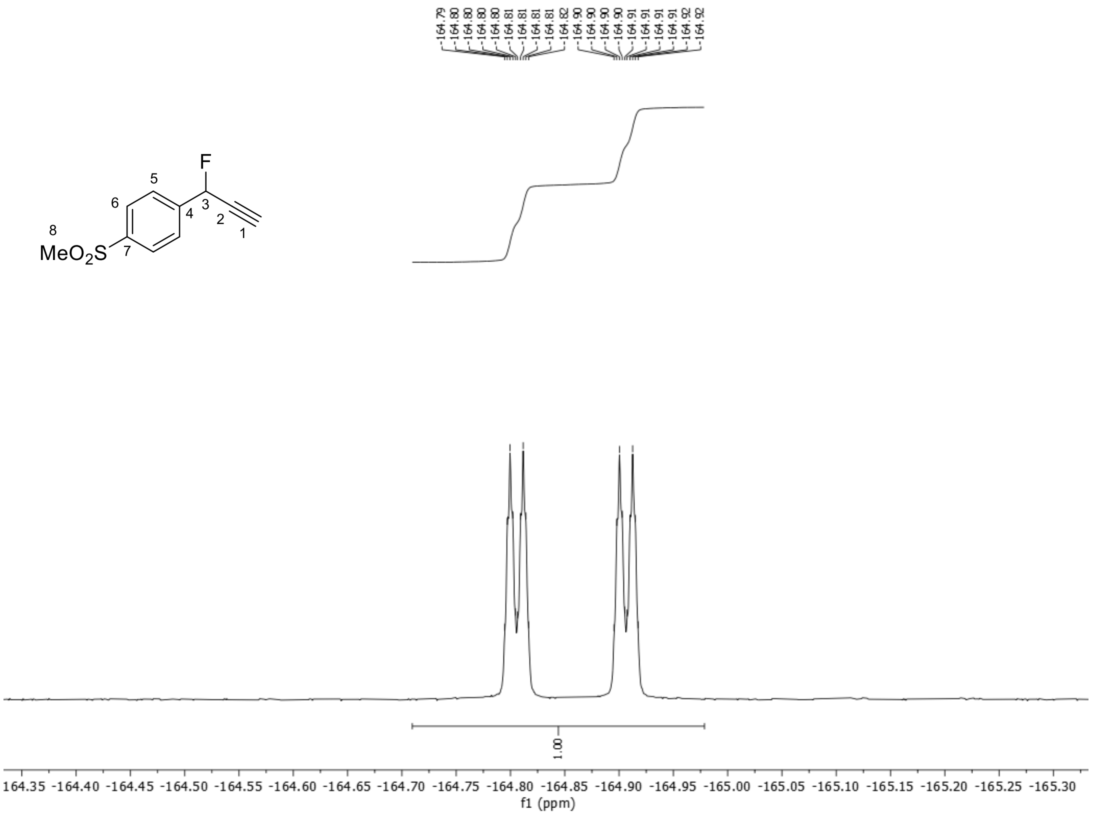

Supplementary Figure 135. ^{19}F NMR of **21** (470 MHz, 299 K, $CDCl_3$).

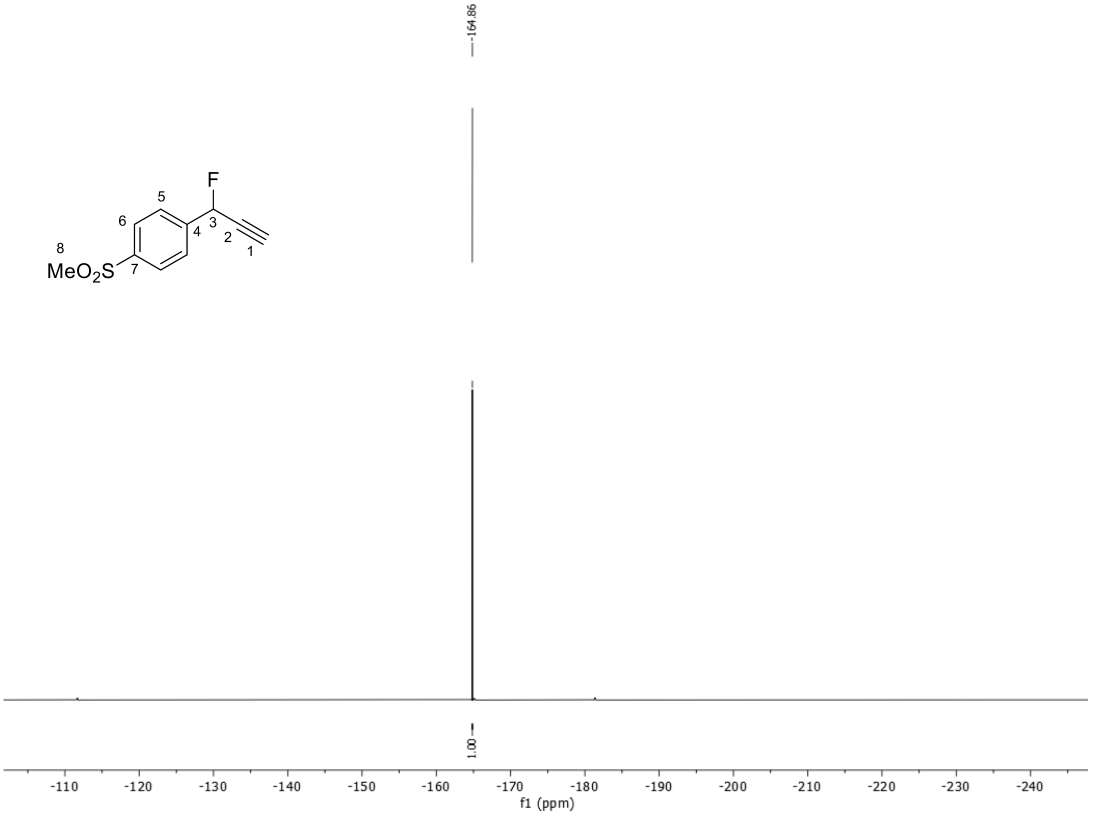

Supplementary Figure 136. ^{19}F{^{1}H} NMR of **21** (470 MHz, 299 K, $CDCl_3$).

(1R,2S,5R)-2-Isopropyl-5-methylcyclohexyl 4-(1-fluoroprop-2-yn-1-yl)benzoate (22)

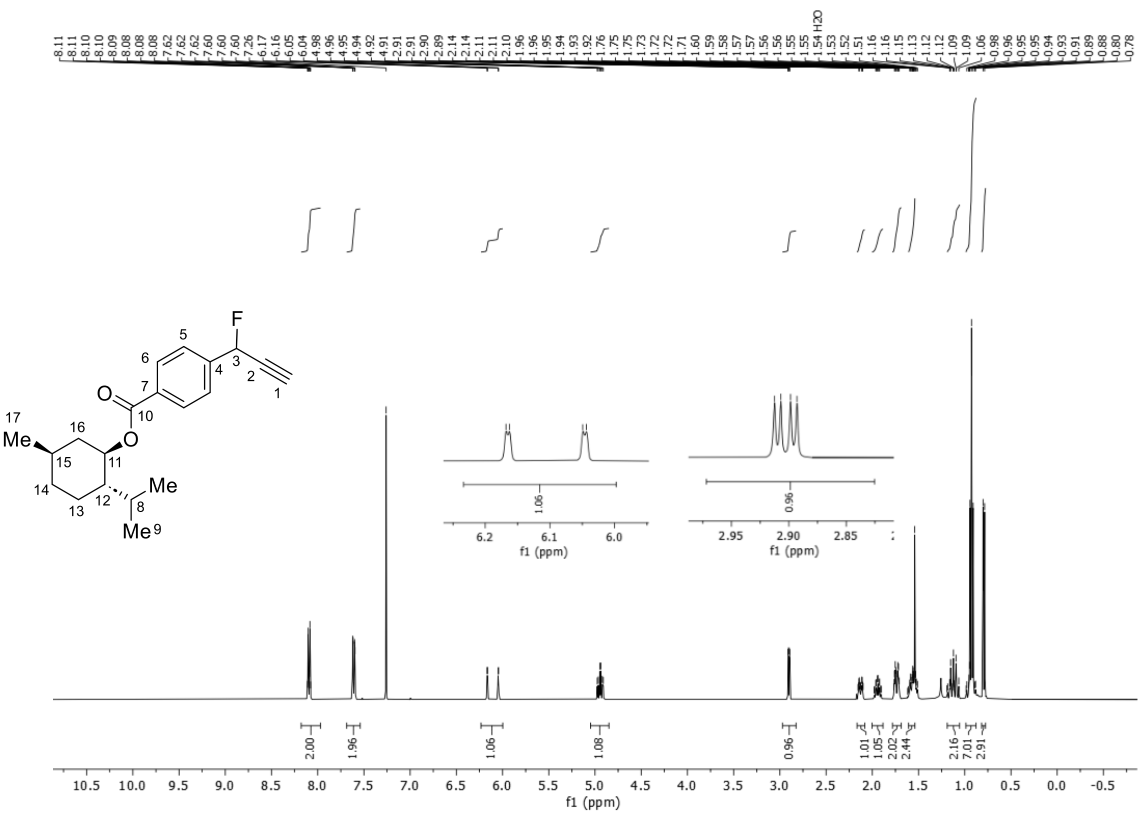

Supplementary Figure 137. ^{1}H NMR of **22** (400 MHz, 299 K, $CDCl_3$).

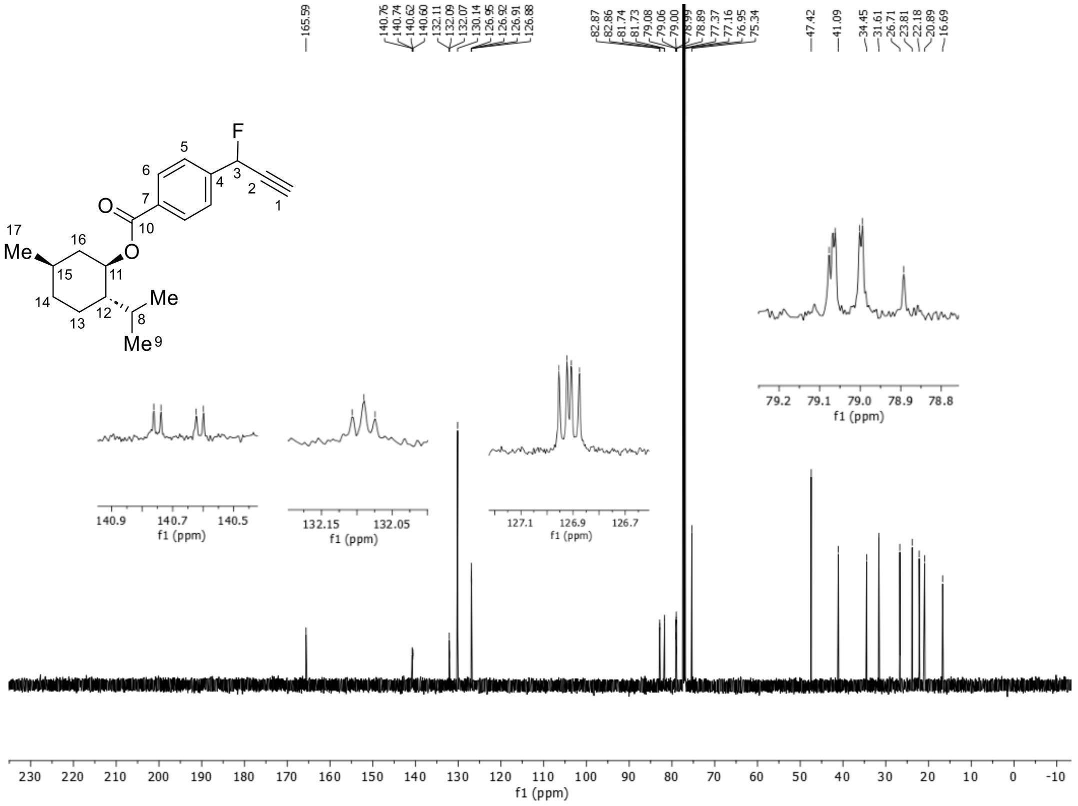

Supplementary Figure 138. $^{13}C\{^{1}H\}$ NMR of **22** (151 MHz, 299 K, $CDCl_3$).

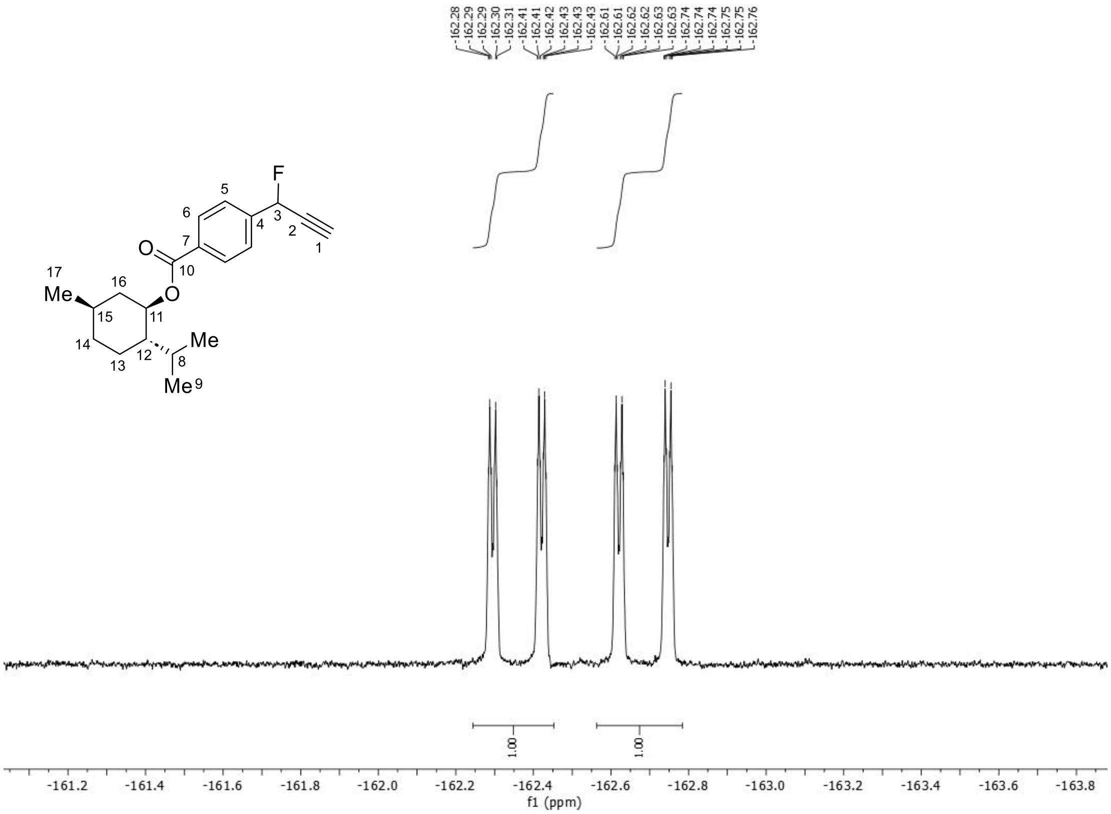

Supplementary Figure 139. ^{19}F NMR of **22** (377 MHz, 299 K, $CDCl_3$).

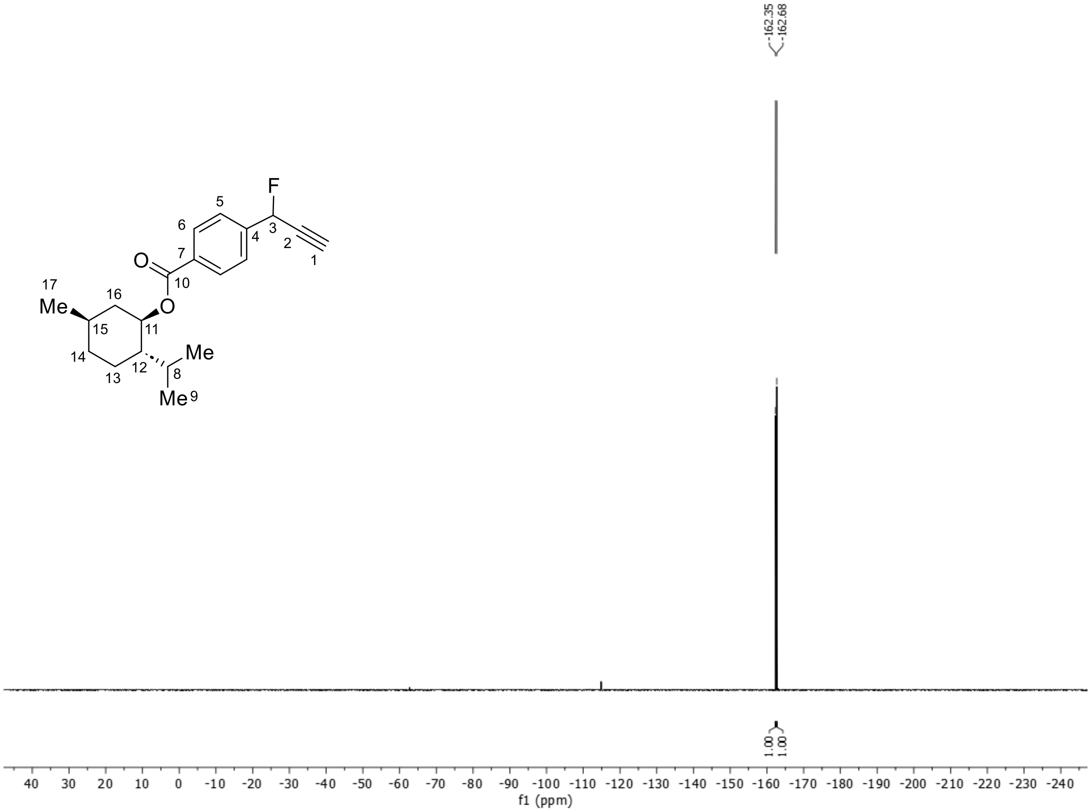

Supplementary Figure 140. ^{19}F{^{1}H} NMR of **22** (377 MHz, 299 K, $CDCl_3$).

Methyl 4-(1-chloro-2-fluorobut-3-yn-2-yl)benzoate (23)

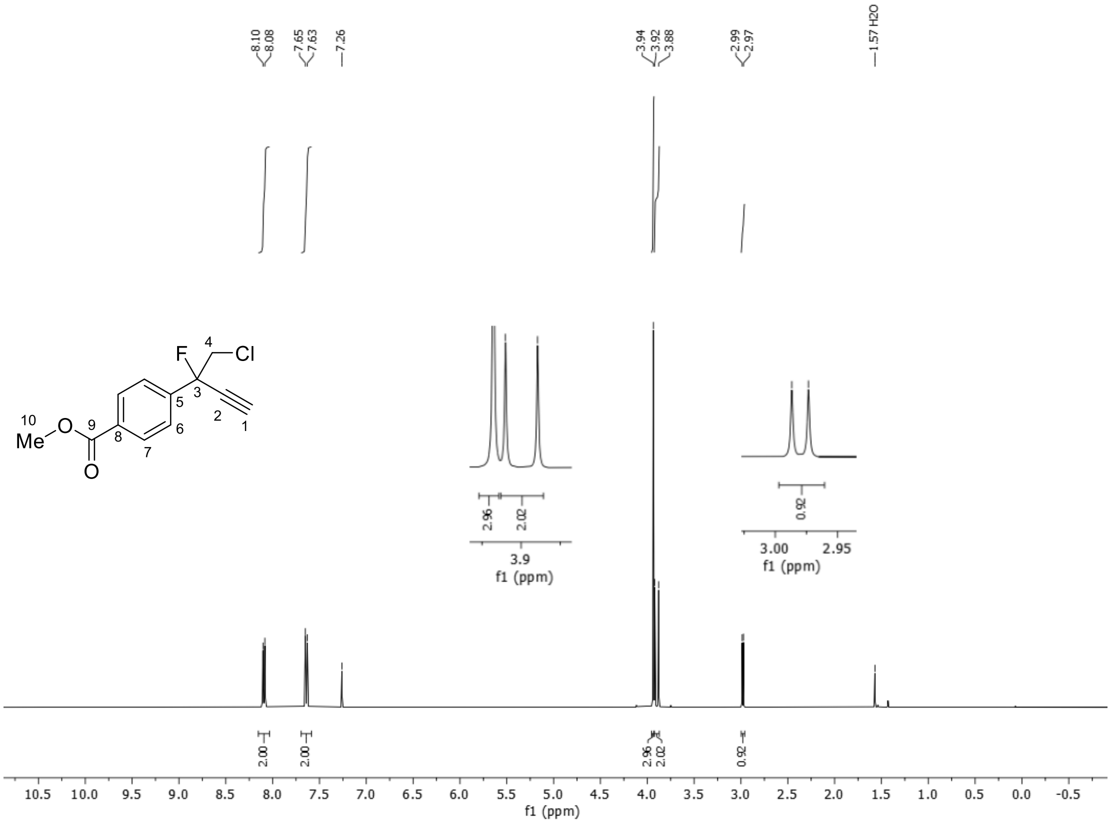

Supplementary Figure 141. ^{1}H NMR of **23** (400 MHz, 299 K, $CDCl_3$).

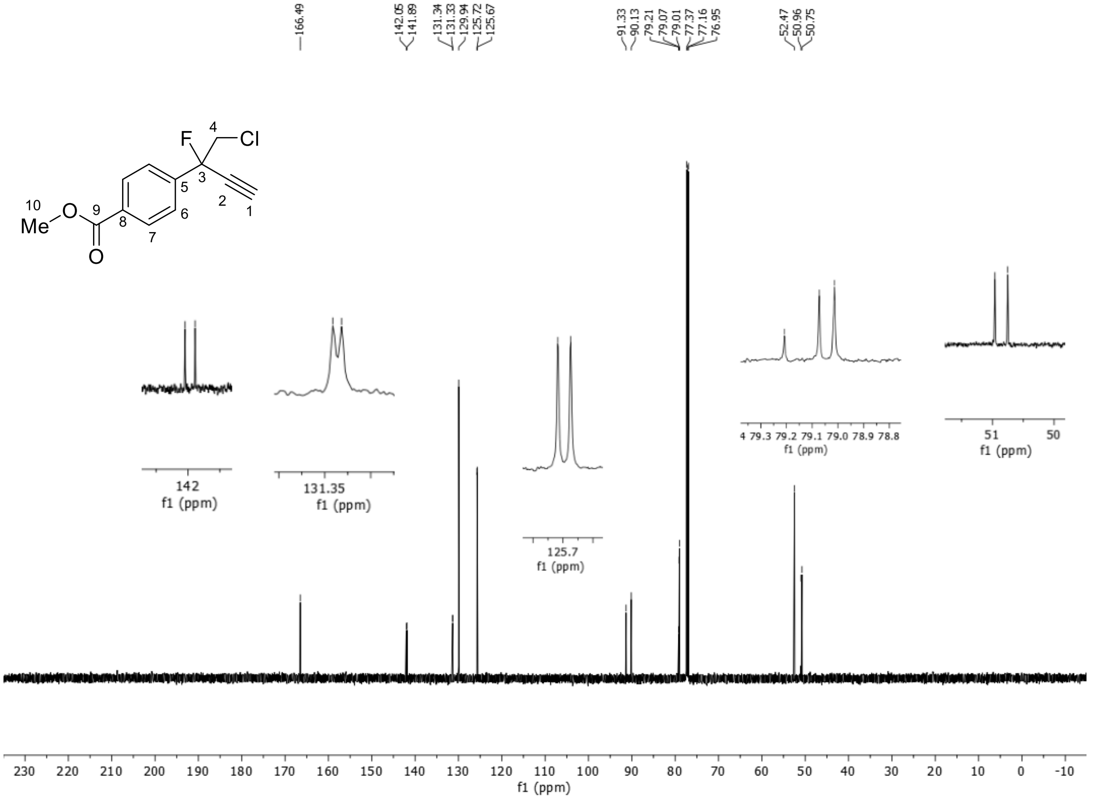

Supplementary Figure 142. $^{13}C\{^{1}H\}$ NMR of **23** (151 MHz, 299 K, $CDCl_3$).

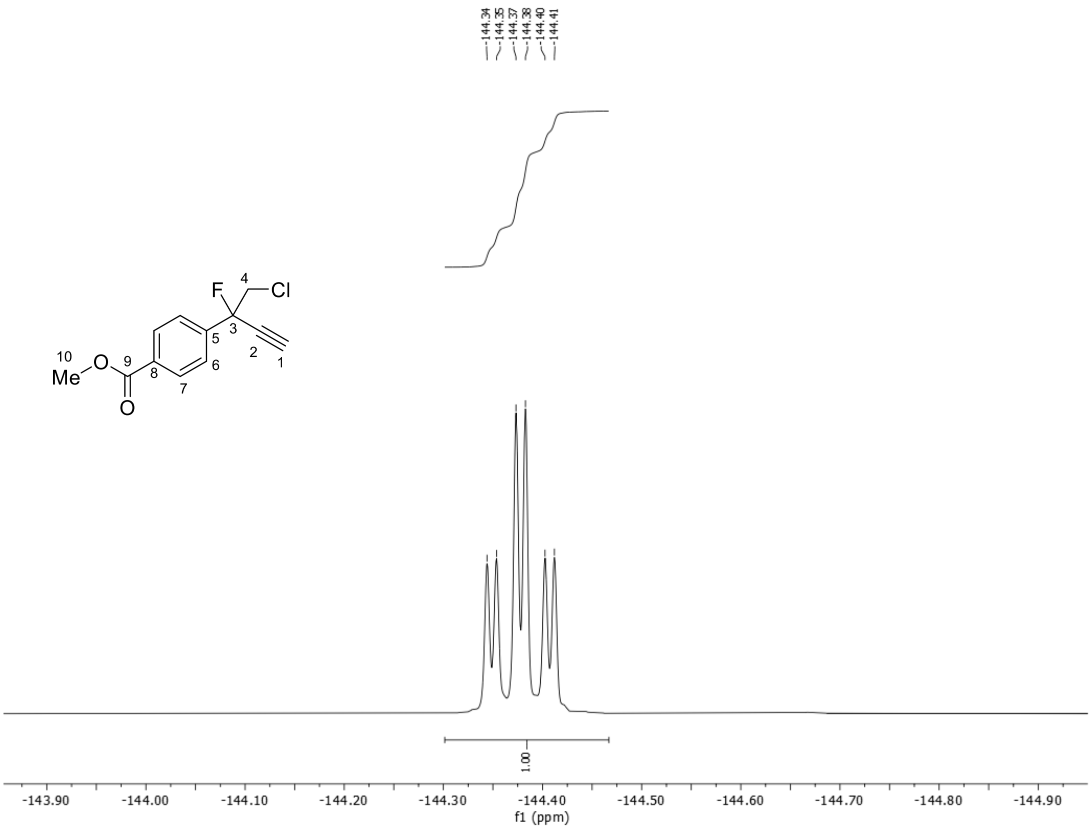

Supplementary Figure 143. ^{19}F NMR of **23** (564 MHz, 299 K, $CDCl_3$).

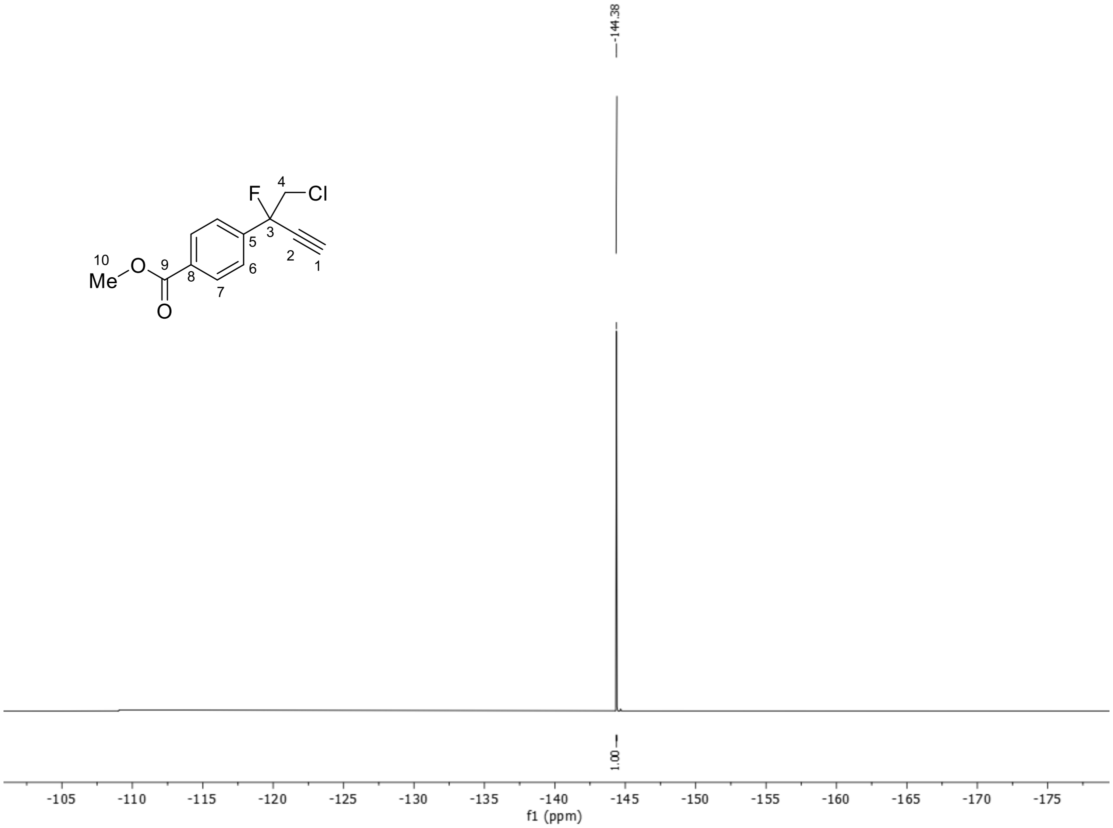

Supplementary Figure 144. ^{19}F{^{1}H} NMR of **23** (564 MHz, 299 K, $CDCl_3$).

1-(1-Chloro-2-fluorobut-3-yn-2-yl)-4-(trifluoromethyl)benzene (24)

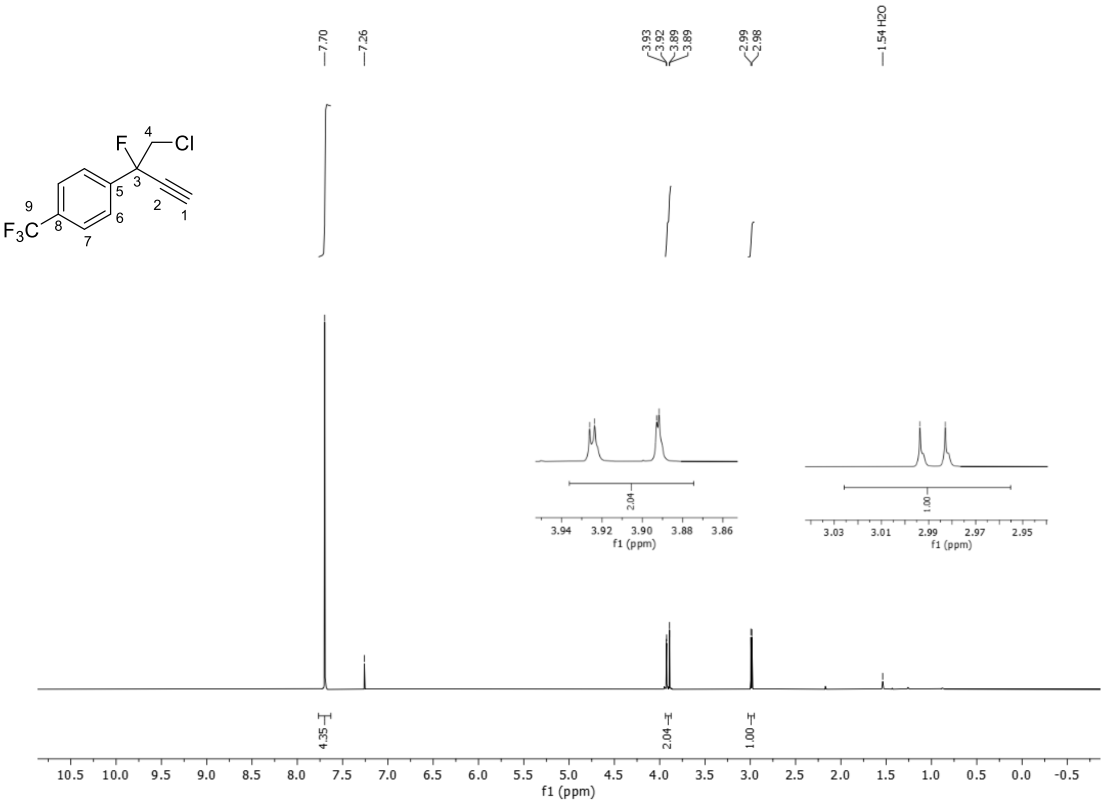

Supplementary Figure 145. ^{1}H NMR of **24** (500 MHz, 299 K, $CDCl_3$).

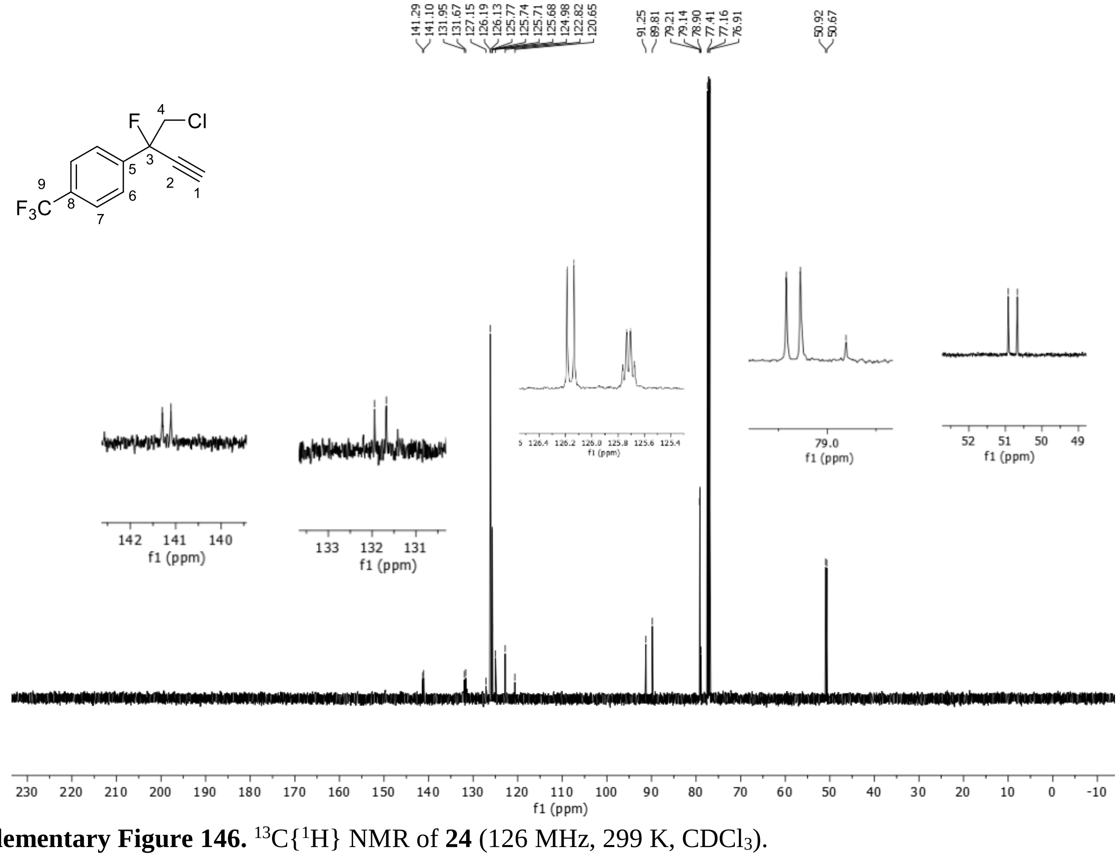

Supplementary Figure 146. $^{13}C\{^1H\}$ NMR of **24** (126 MHz, 299 K, $CDCl_3$).

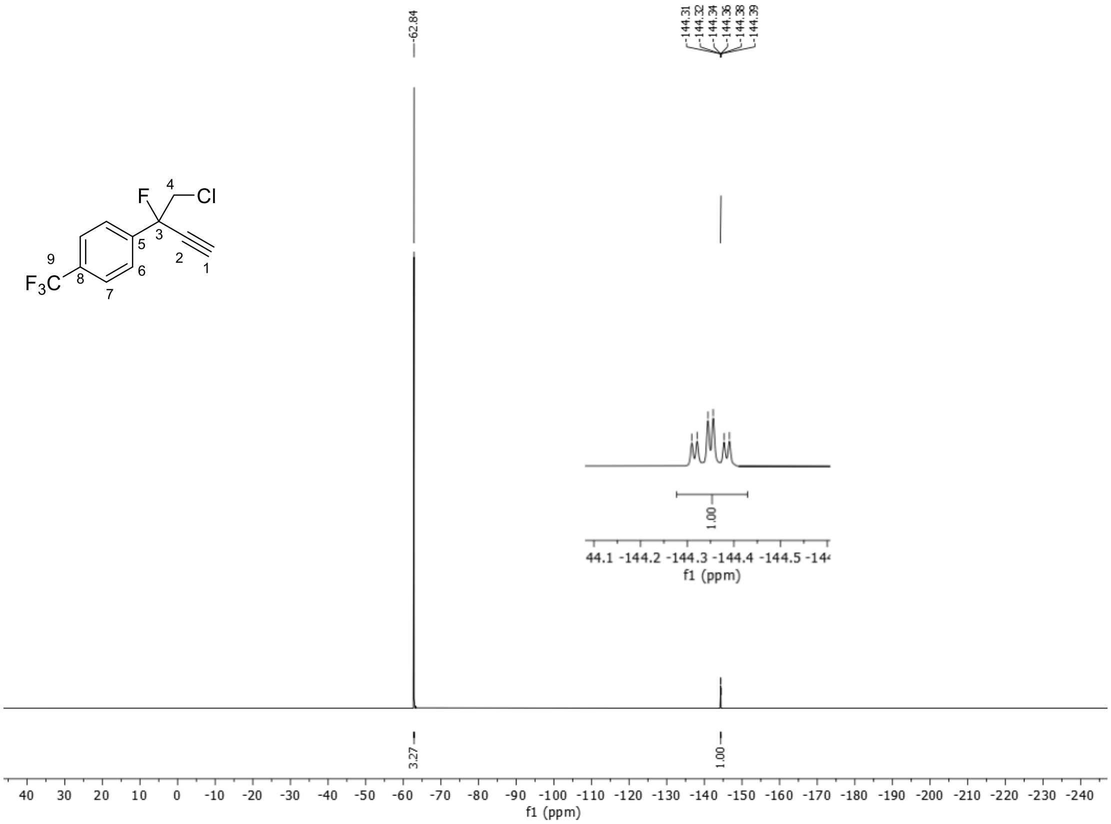

Supplementary Figure 147. ^{19}F NMR of **24** (470 MHz, 299 K, $CDCl_3$).

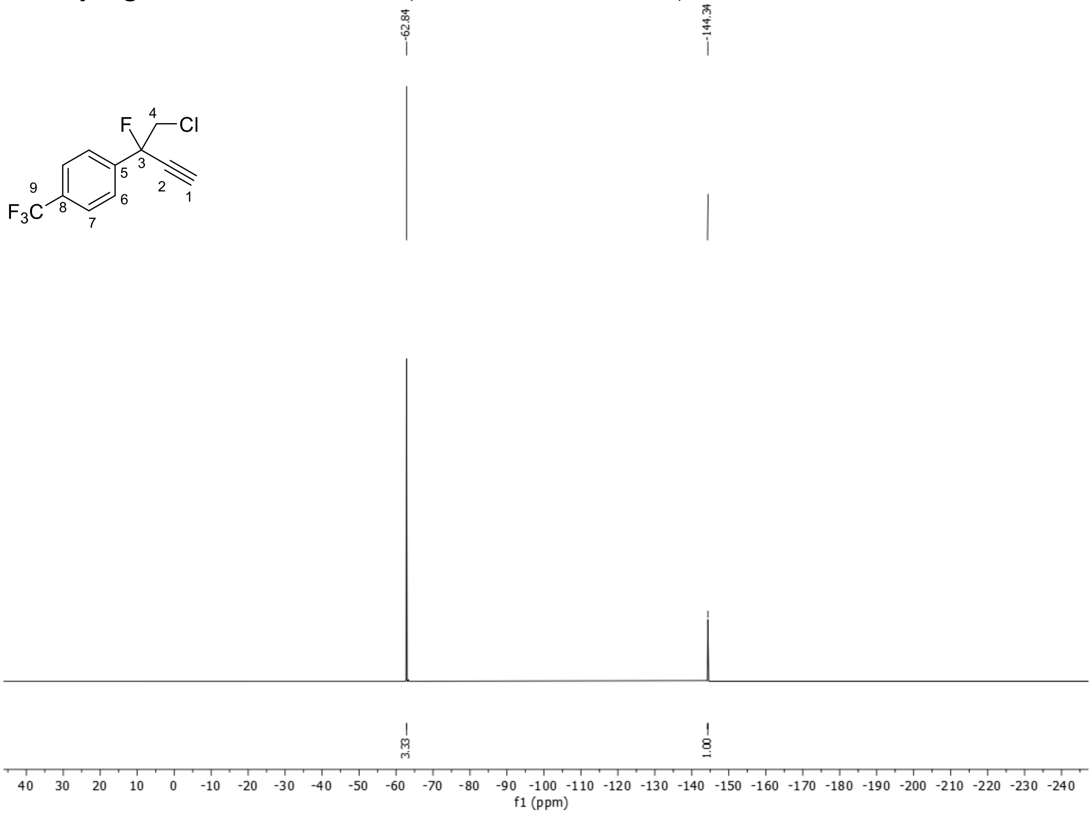

Supplementary Figure 148. ^{19}F{^{1}H} NMR of **24** (377 MHz, 299 K, $CDCl_3$).

(2-(Chloromethyl)-2-fluorobut-3-yn-1-yl)benzene (25)

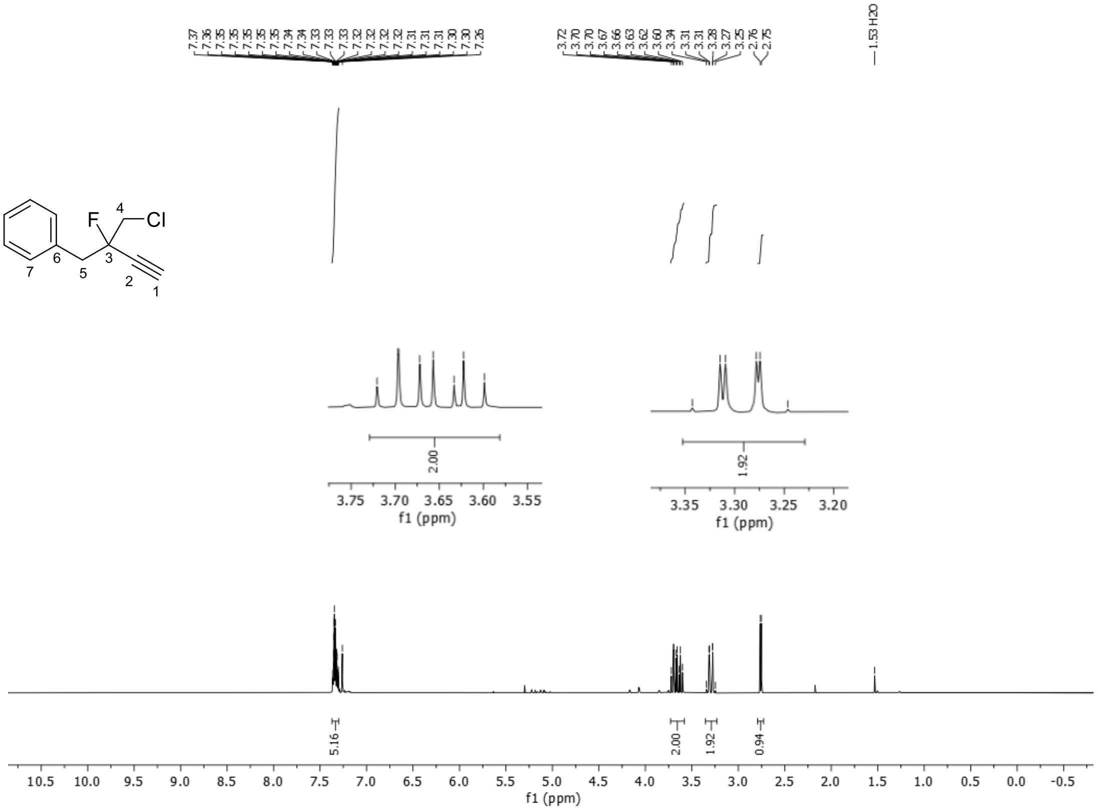

Supplementary Figure 149. ^{1}H NMR of **25** (500 MHz, 299 K, $CDCl_3$).

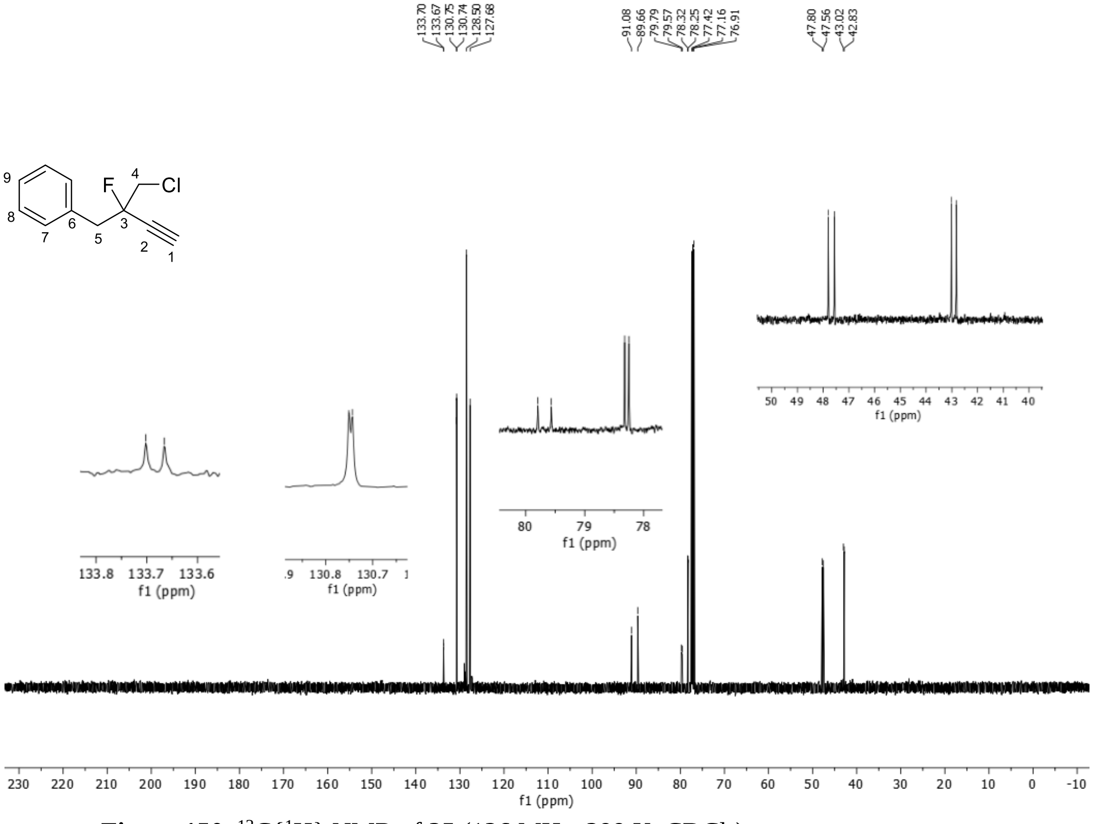

Supplementary Figure 150. $^{13}C\{^{1}H\}$ NMR of **25** (126 MHz, 299 K, $CDCl_3$).

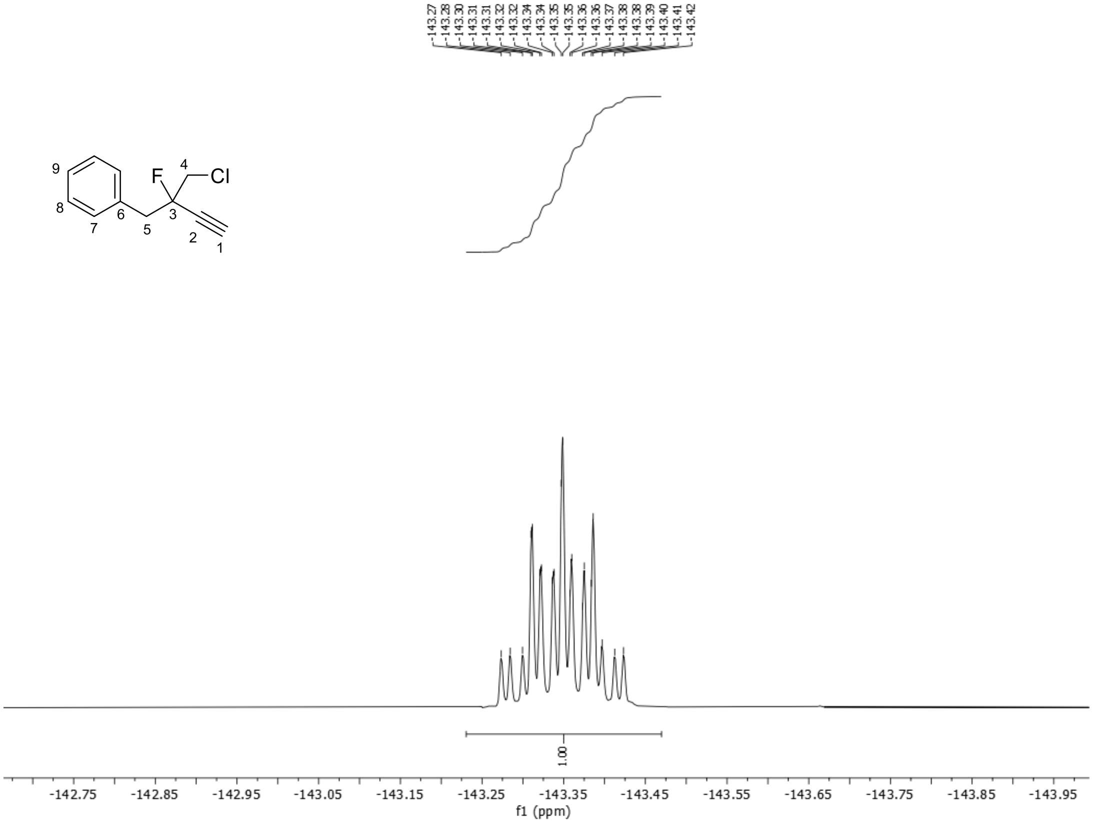

Supplementary Figure 151. ^{19}F NMR of **25** (470 MHz, 299 K, $CDCl_3$).

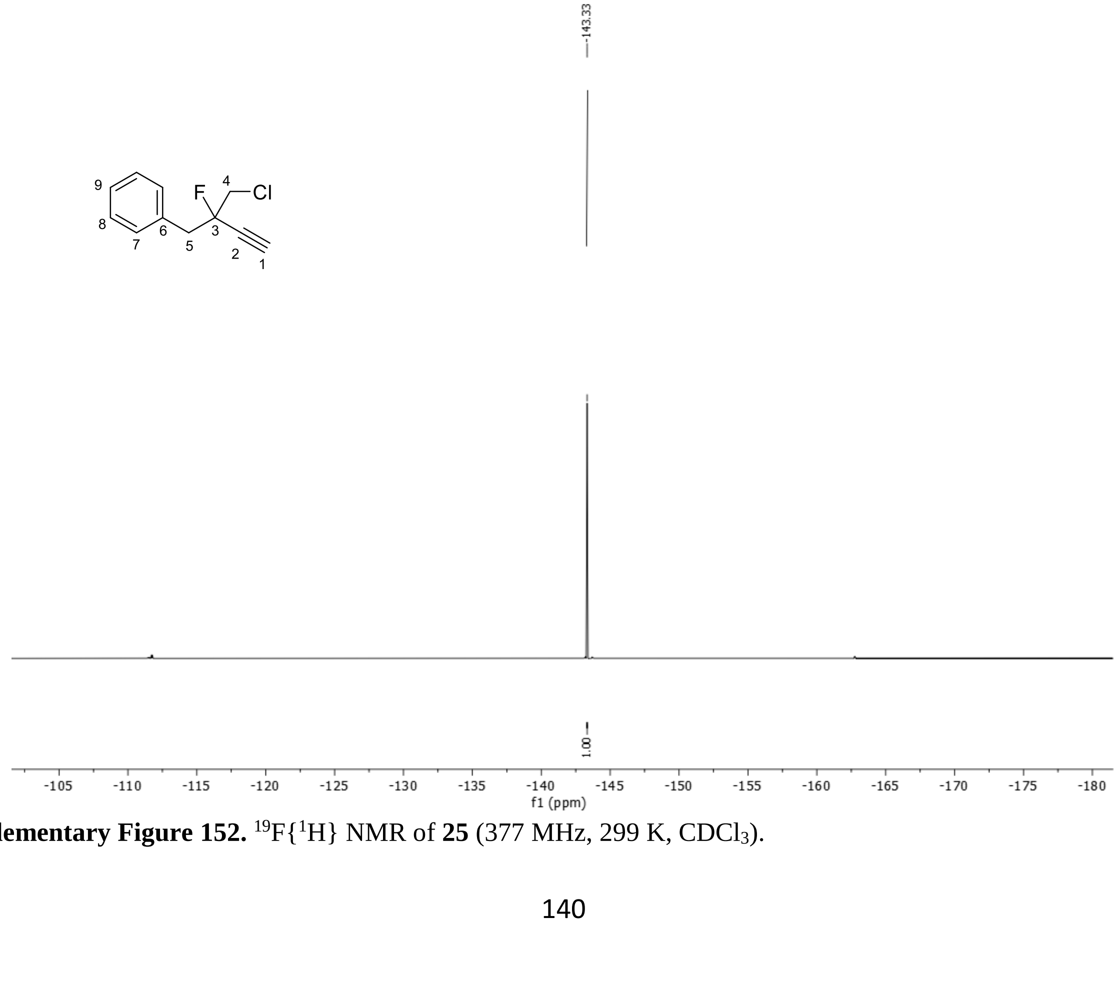

Supplementary Figure 152. $^{19}F\{^1H\}$ NMR of **25** (377 MHz, 299 K, $CDCl_3$).

3-(Chloromethyl)-3-fluoropentadec-1-yne (26)

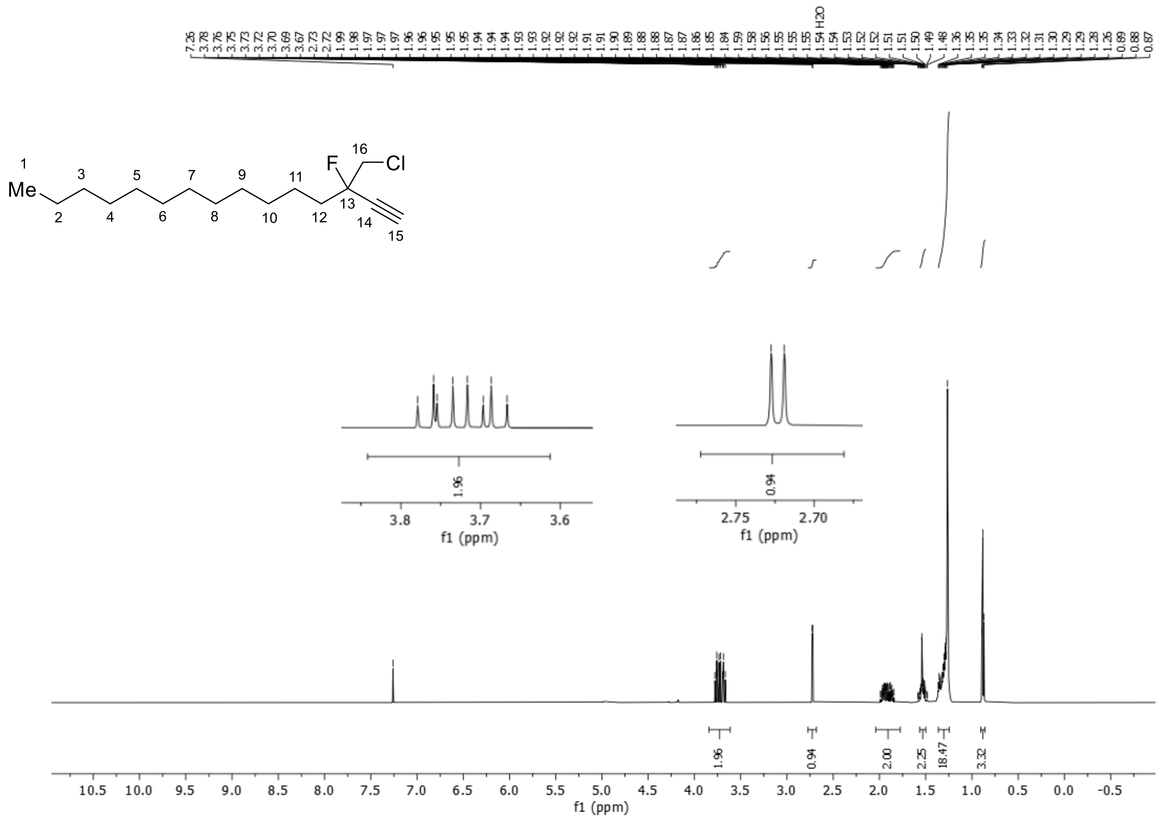

Supplementary Figure 153. ^{1}H NMR of **26** (599 MHz, 299 K, $CDCl_3$).

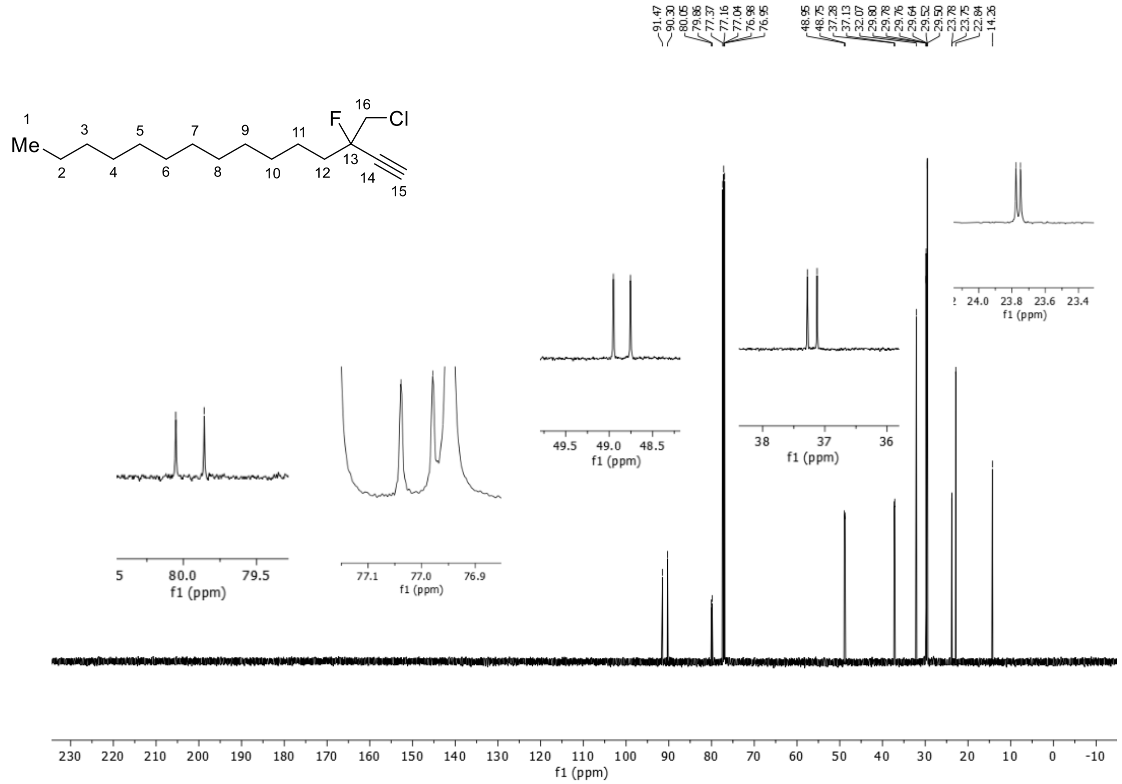

Supplementary Figure 154. $^{13}C\{^{1}H\}$ NMR of **26** (151 MHz, 299 K, $CDCl_3$).

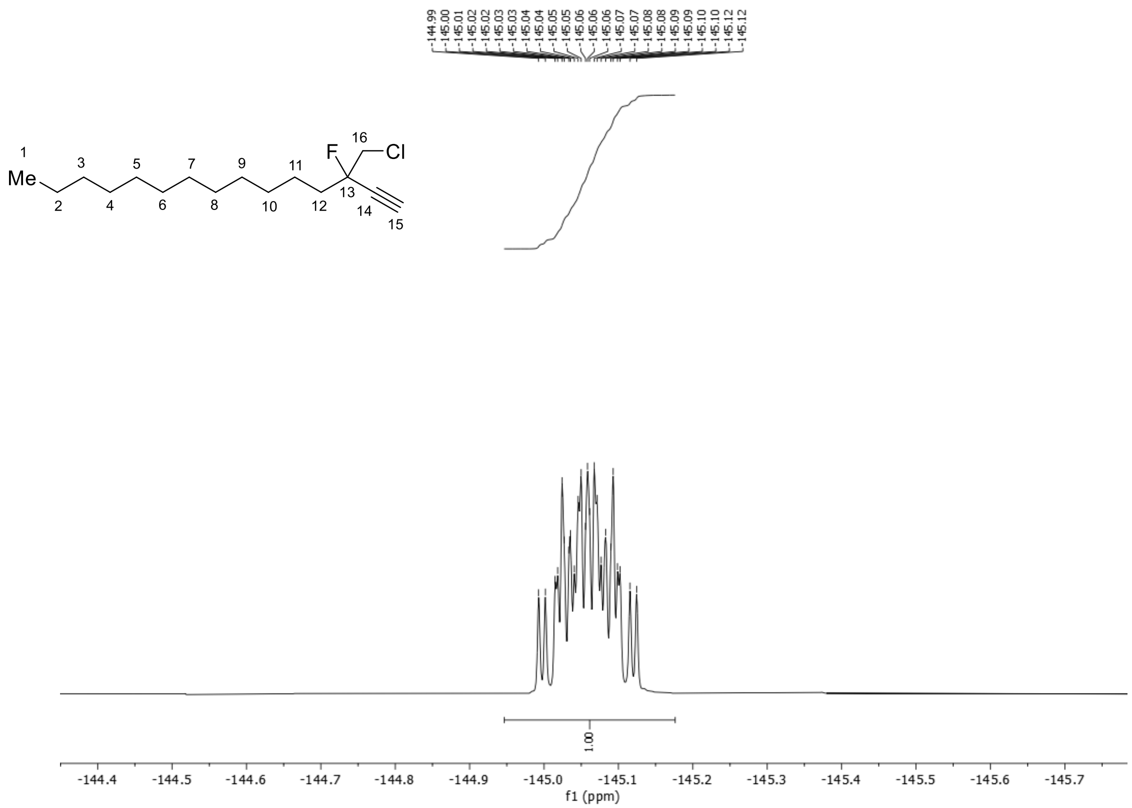

Supplementary Figure 155. ^{19}F NMR of **26** (564 MHz, 299 K, $CDCl_3$).

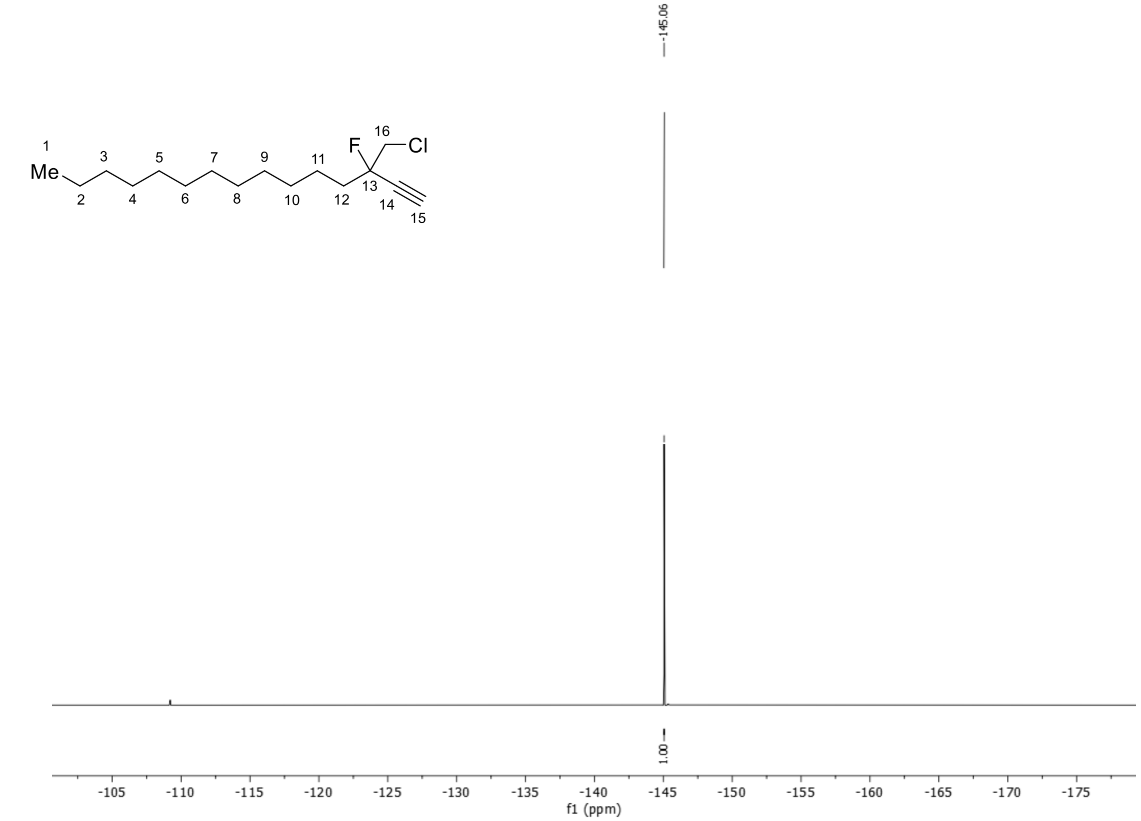

Supplementary Figure 156. $^{19}F\{^1H\}$ NMR of **26** (564 MHz, 299 K, $CDCl_3$).

3-Fluoro-3-(fluoromethyl)pentadec-1-yne (27)

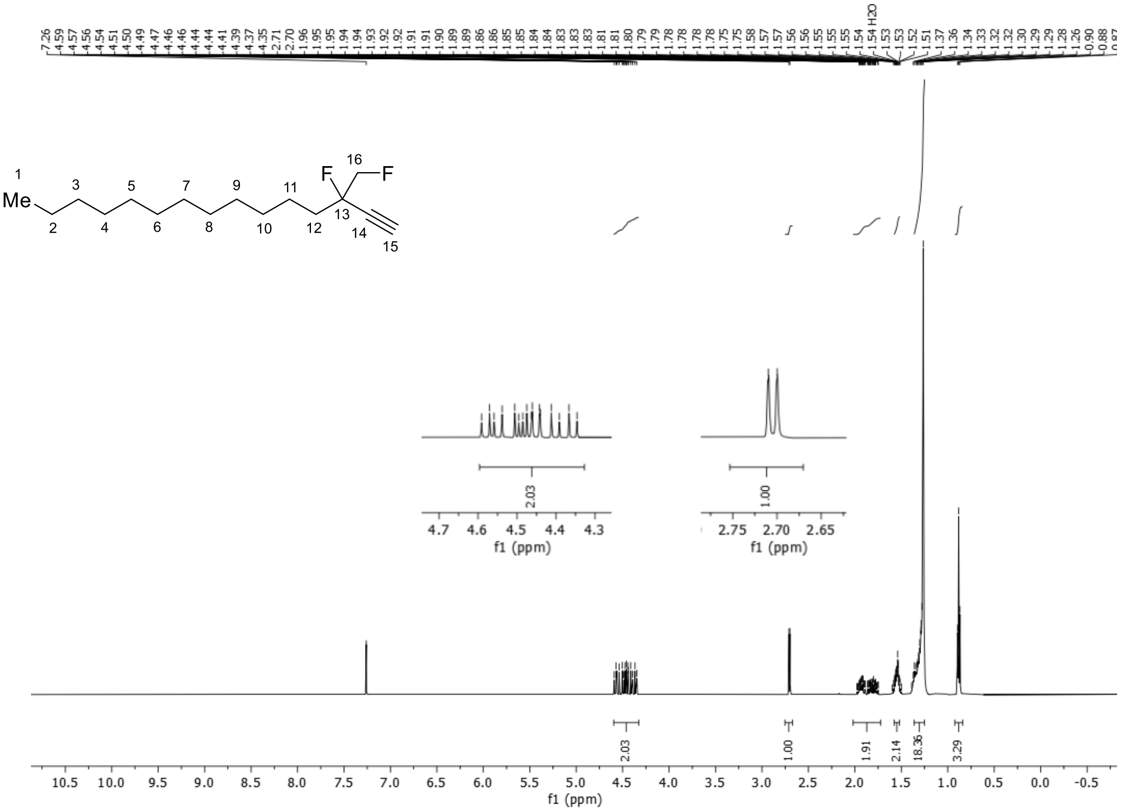

Supplementary Figure 157. ^{1}H NMR of **27** (500 MHz, 299 K, $CDCl_3$).

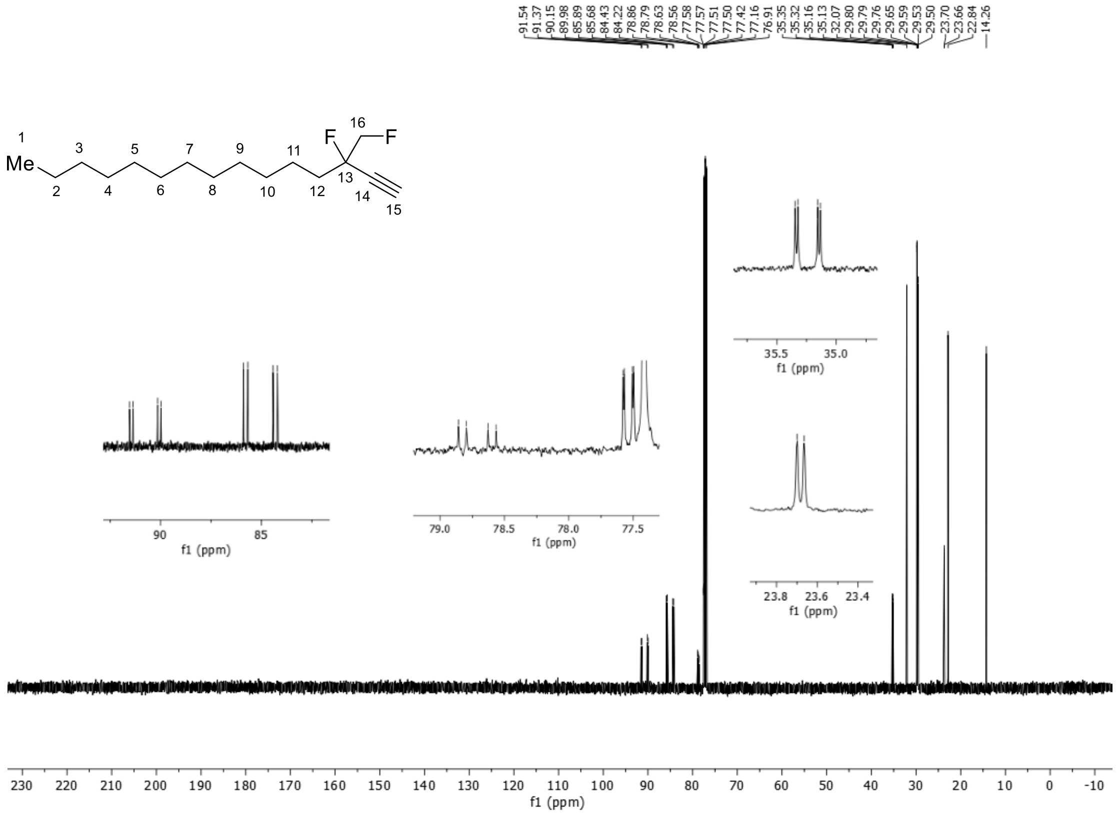

Supplementary Figure 158. $^{13}C\{^{1}H\}$ NMR of **27** (126 MHz, 299 K, $CDCl_3$).

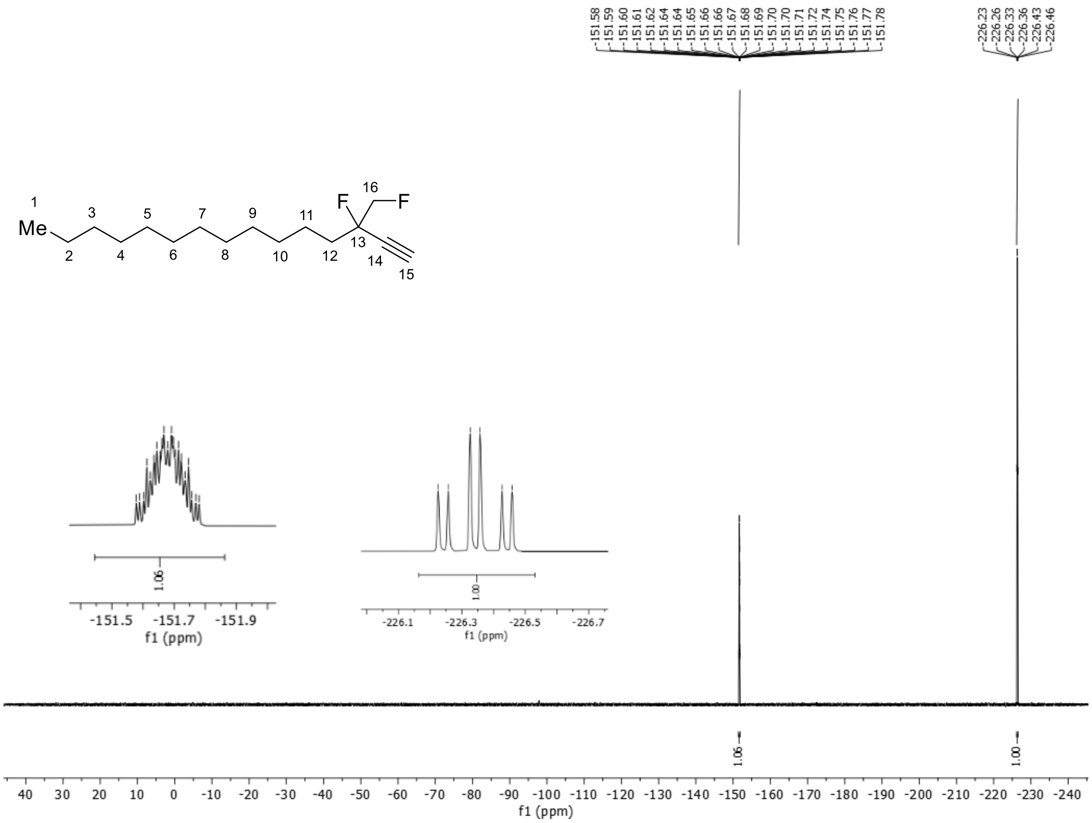

Supplementary Figure 159. ^{19}F NMR of **27** (470 MHz, 299 K, $CDCl_3$).

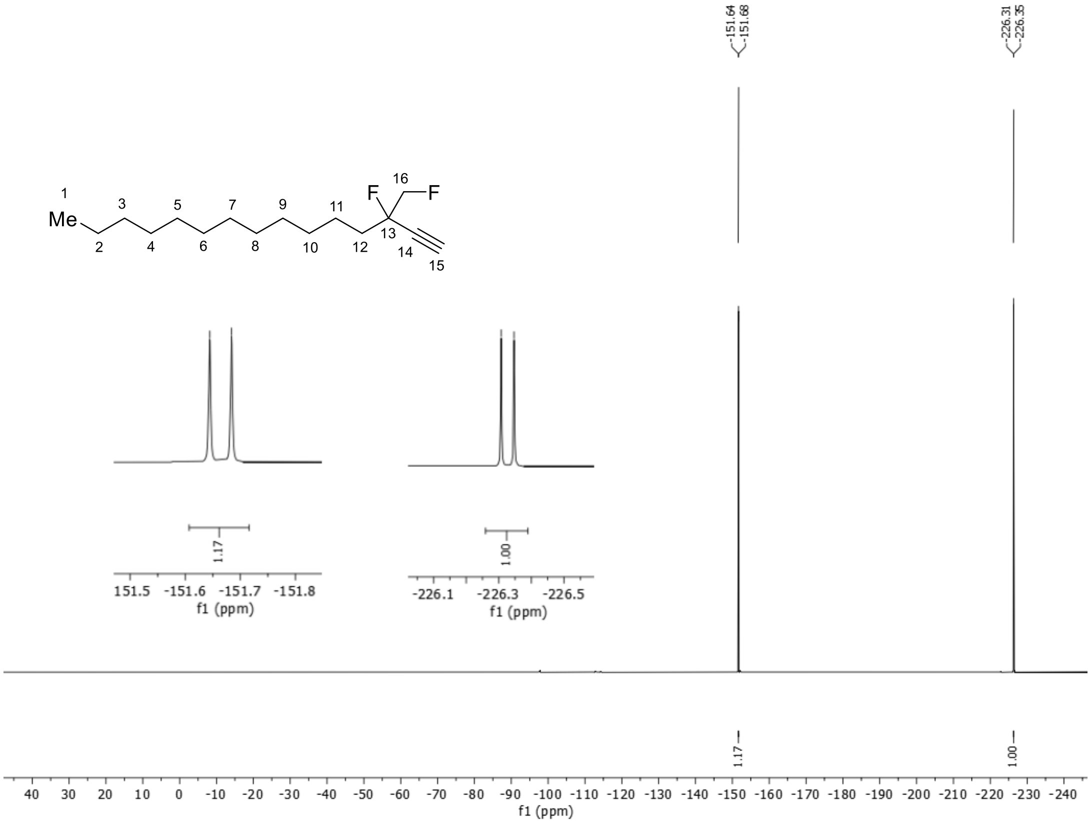

Supplementary Figure 160. $^{19}F\{^1H\}$ NMR of **27** (377 MHz, 299 K, $CDCl_3$).

3-(Chloromethyl)-3-fluoropentadec-1-yne (28)

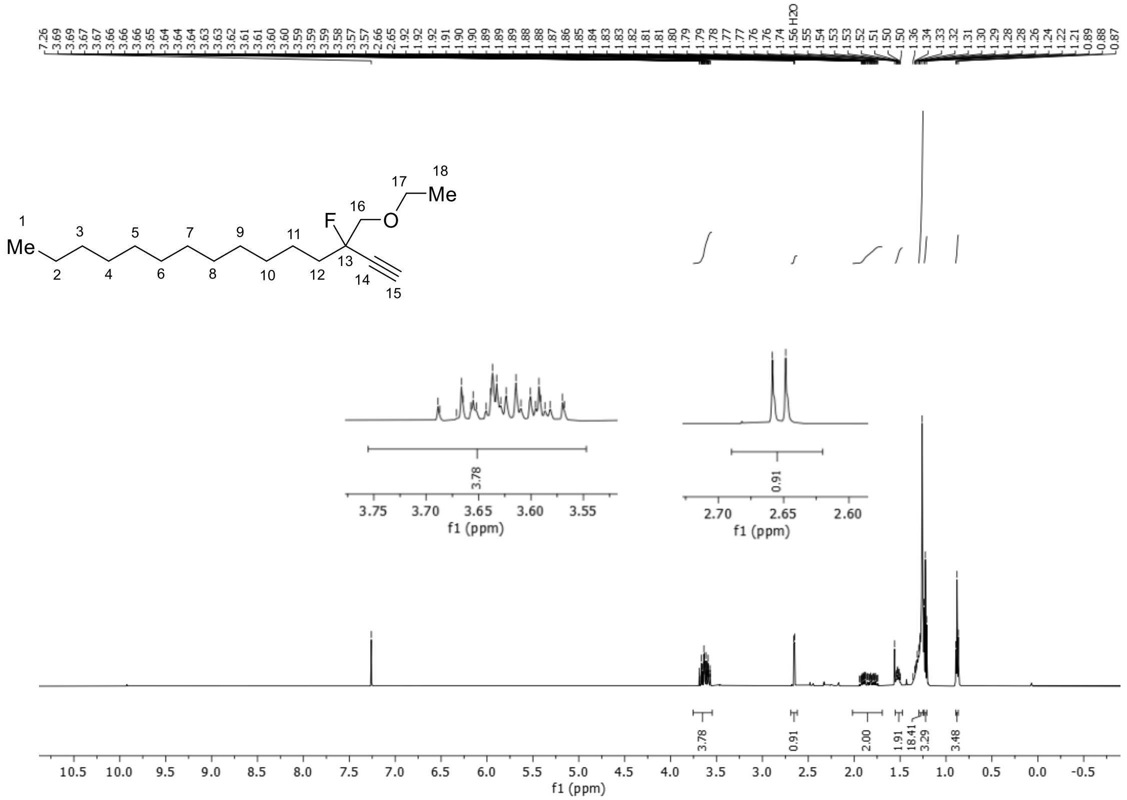

Supplementary Figure 161. ^{1}H NMR of **28** (500 MHz, 299 K, $CDCl_3$).

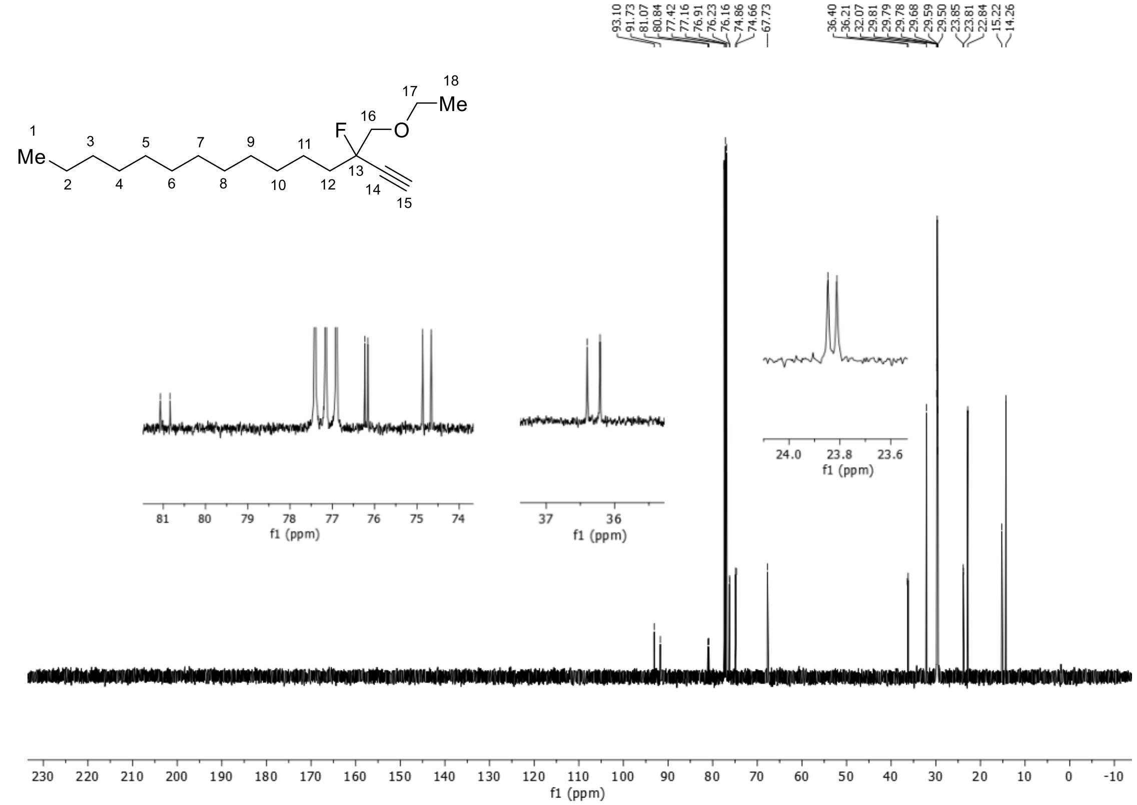

Supplementary Figure 162. $^{13}C\{^{1}H\}$ NMR of **28** (126 MHz, 299 K, $CDCl_3$).

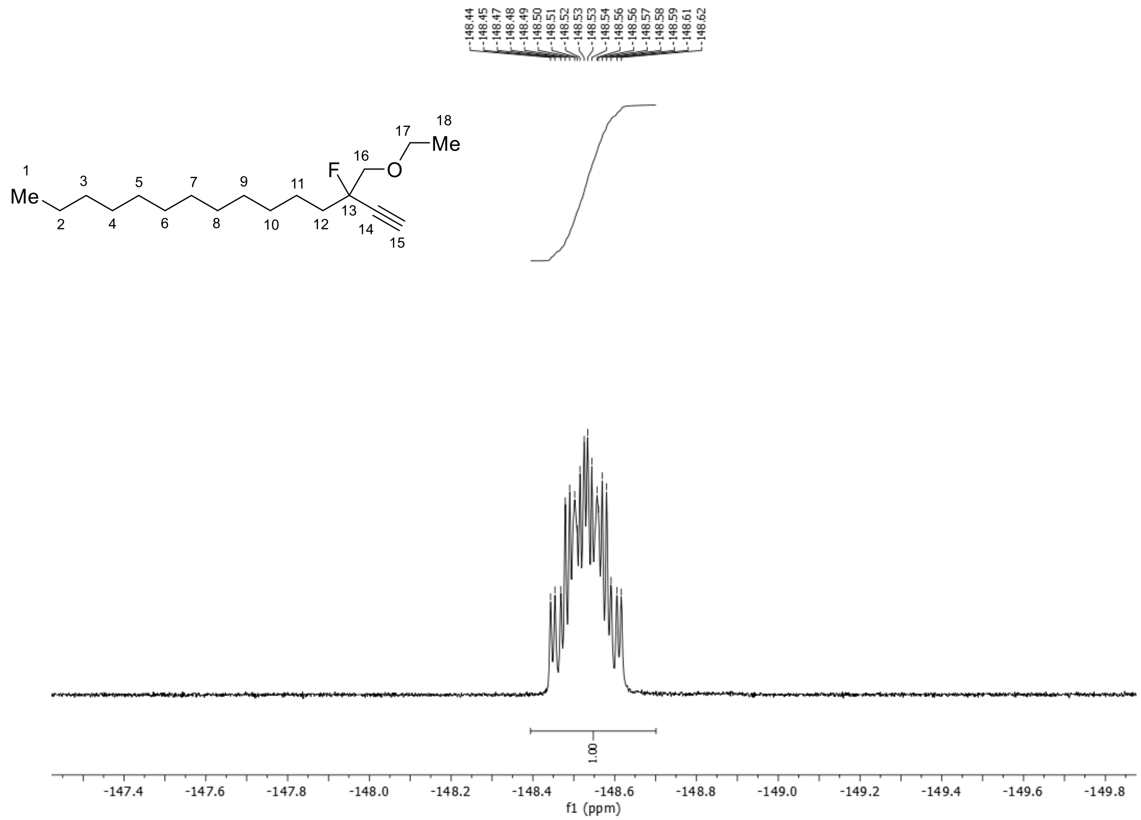

Supplementary Figure 163. ^{19}F NMR of **28** (470 MHz, 299 K, $CDCl_3$).

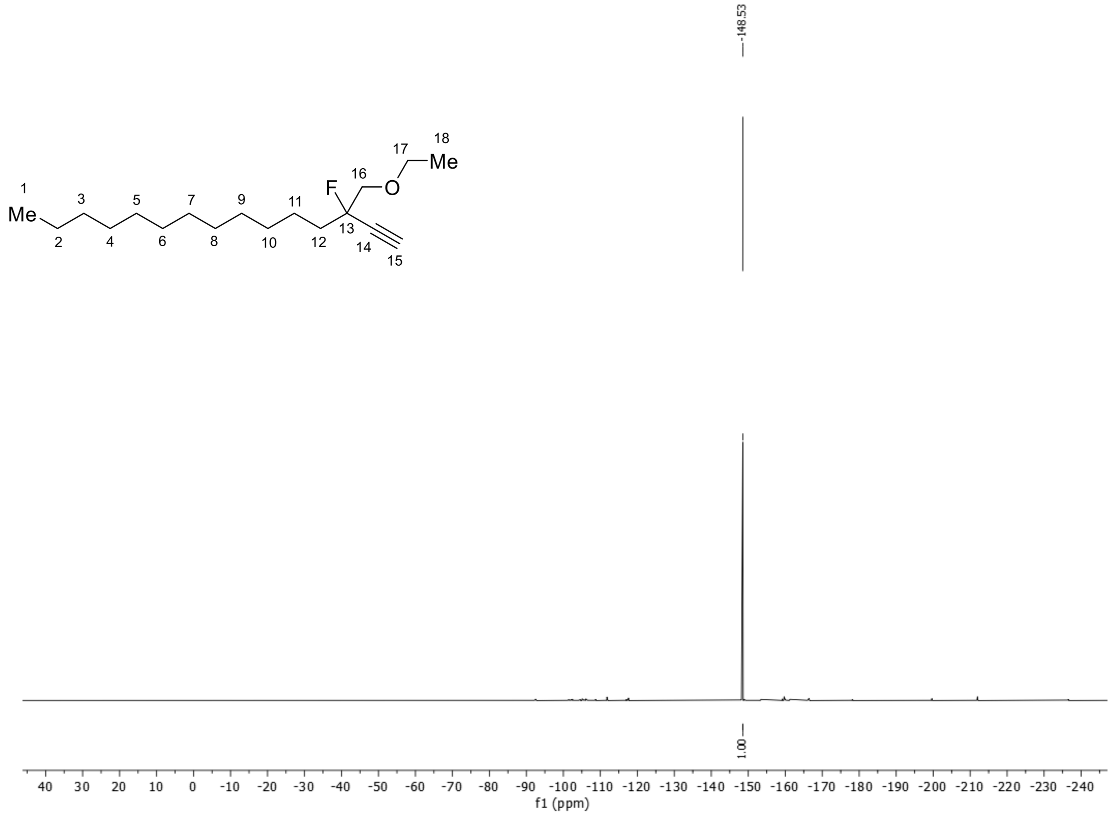

Supplementary Figure 164. $^{19}F\{^1H\}$ NMR of **28** (470 MHz, 299 K, $CDCl_3$).

N-(2-ethynyl-2-fluorotetradecyl)-N,4-dimethylbenzenesulfonamide (29)

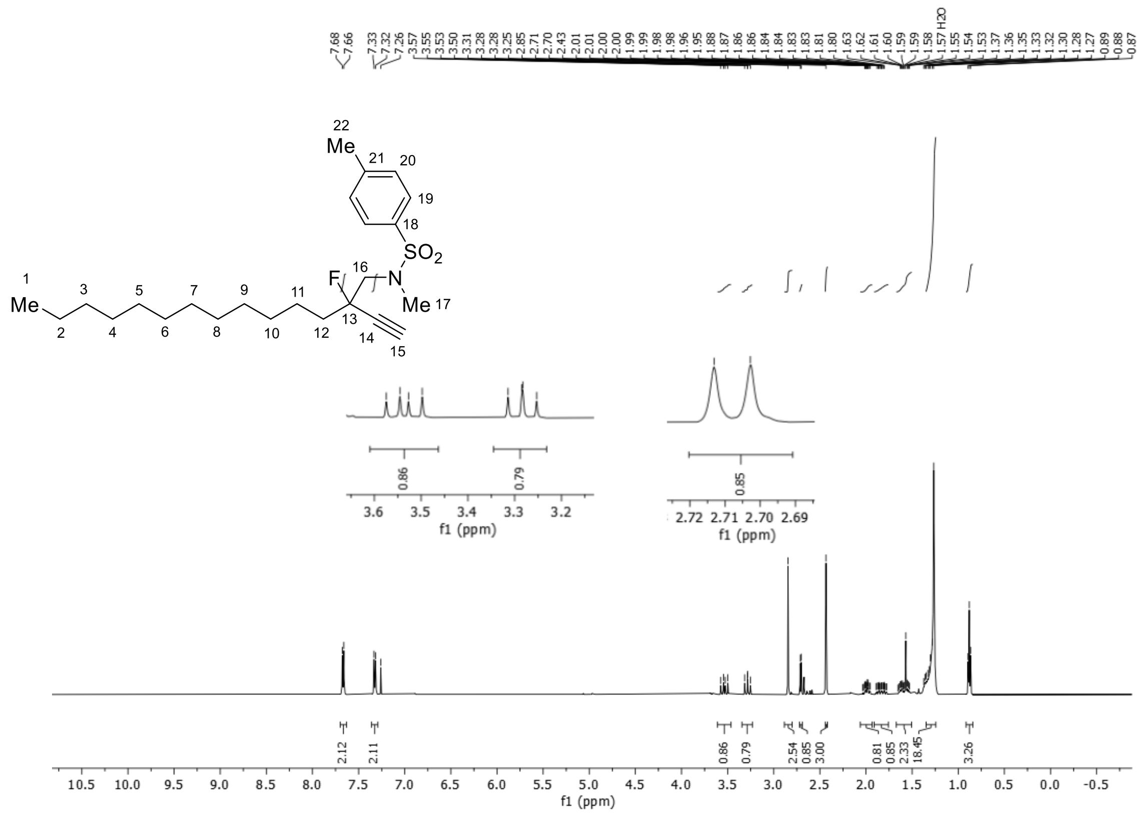

Supplementary Figure 165. ^{1}H NMR of **29** (500 MHz, 299 K, $CDCl_3$).

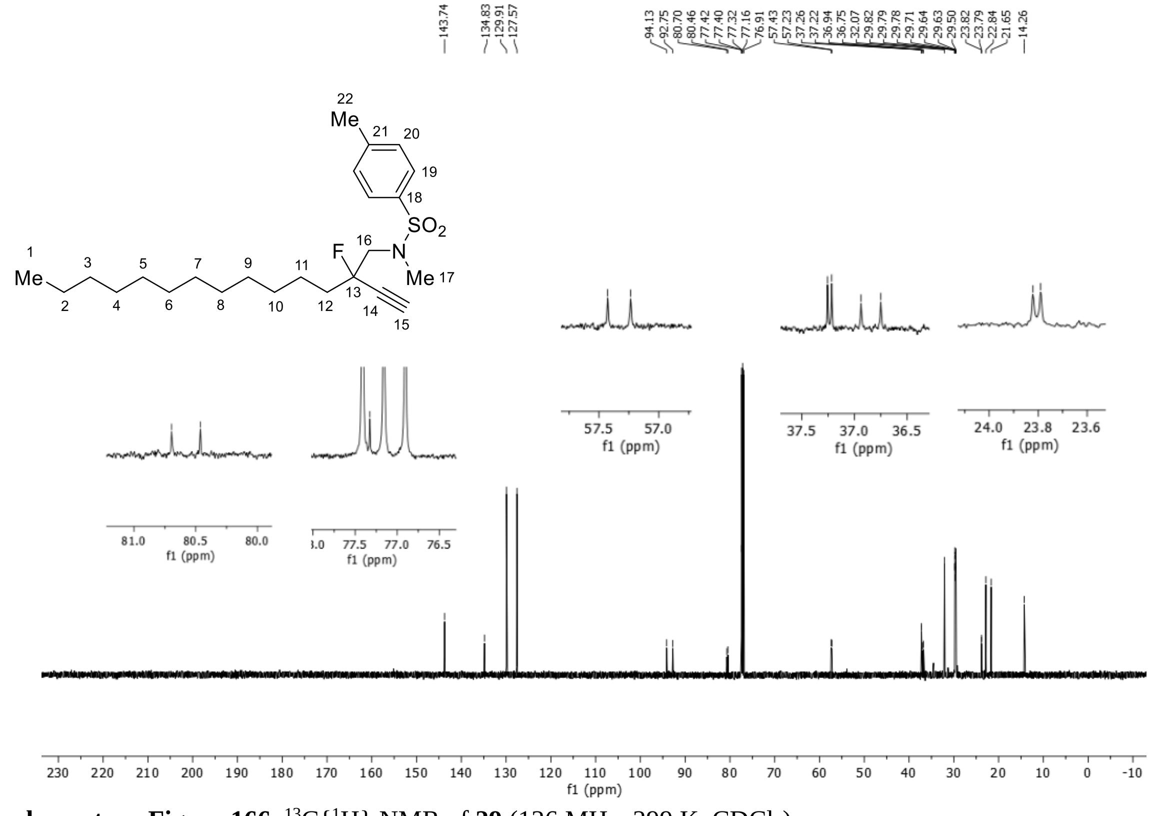

Supplementary Figure 166. $^{13}C\{^{1}H\}$ NMR of **29** (126 MHz, 299 K, $CDCl_3$).

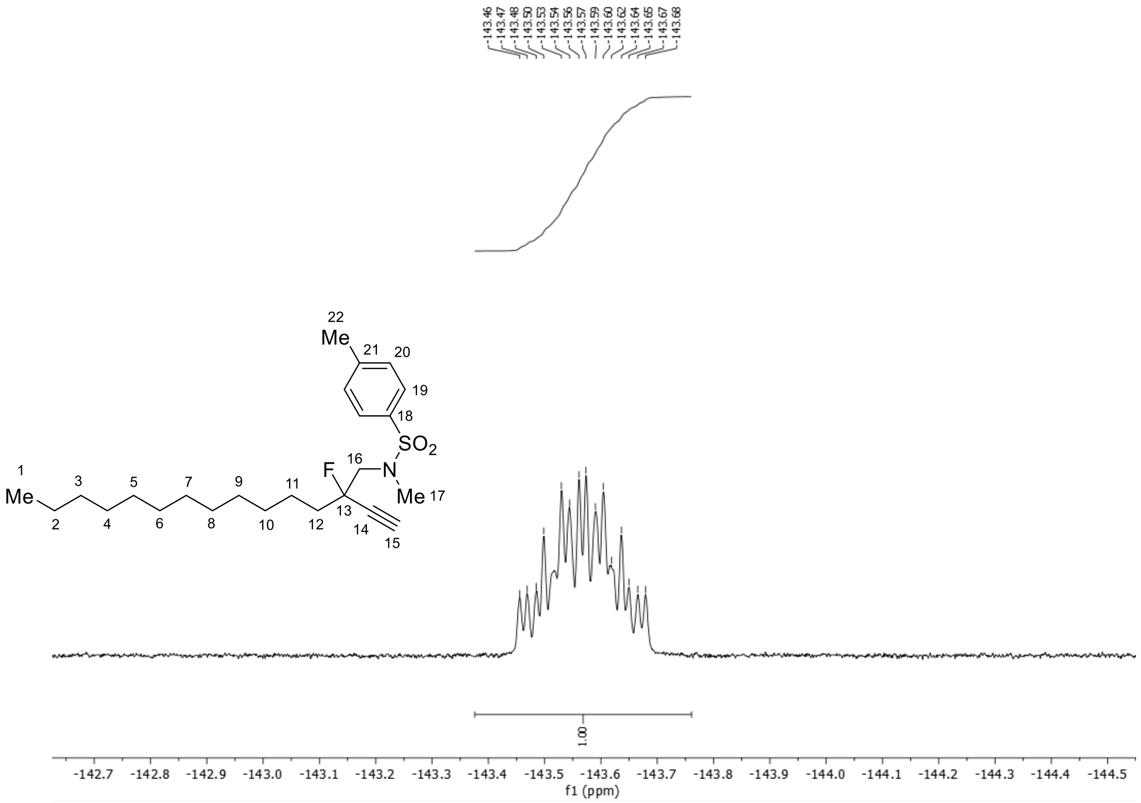

Supplementary Figure 167. ^{19}F NMR of **29** (376 MHz, 299 K, $CDCl_3$).

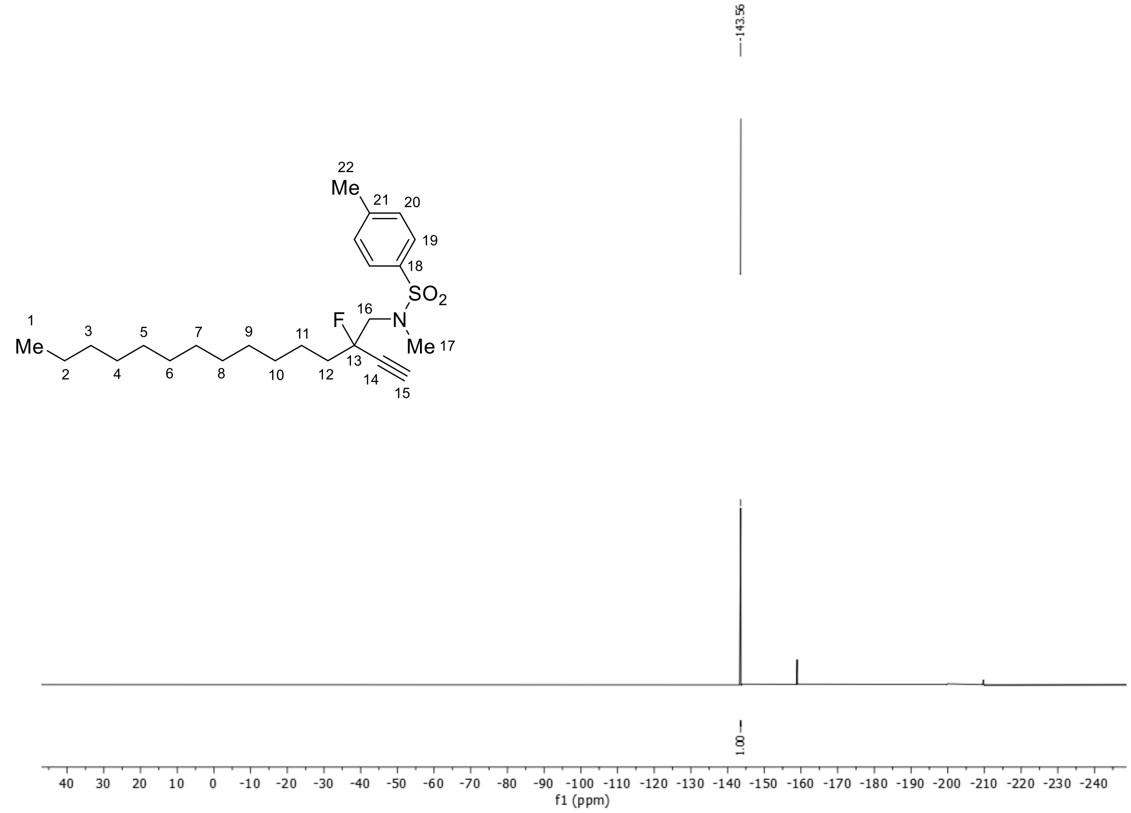

Supplementary Figure 168. ^{19}F{^{1}H} NMR of **29** (376 MHz, 299 K, $CDCl_3$).

4-Ethynyl-4-fluorohexadecane (30)

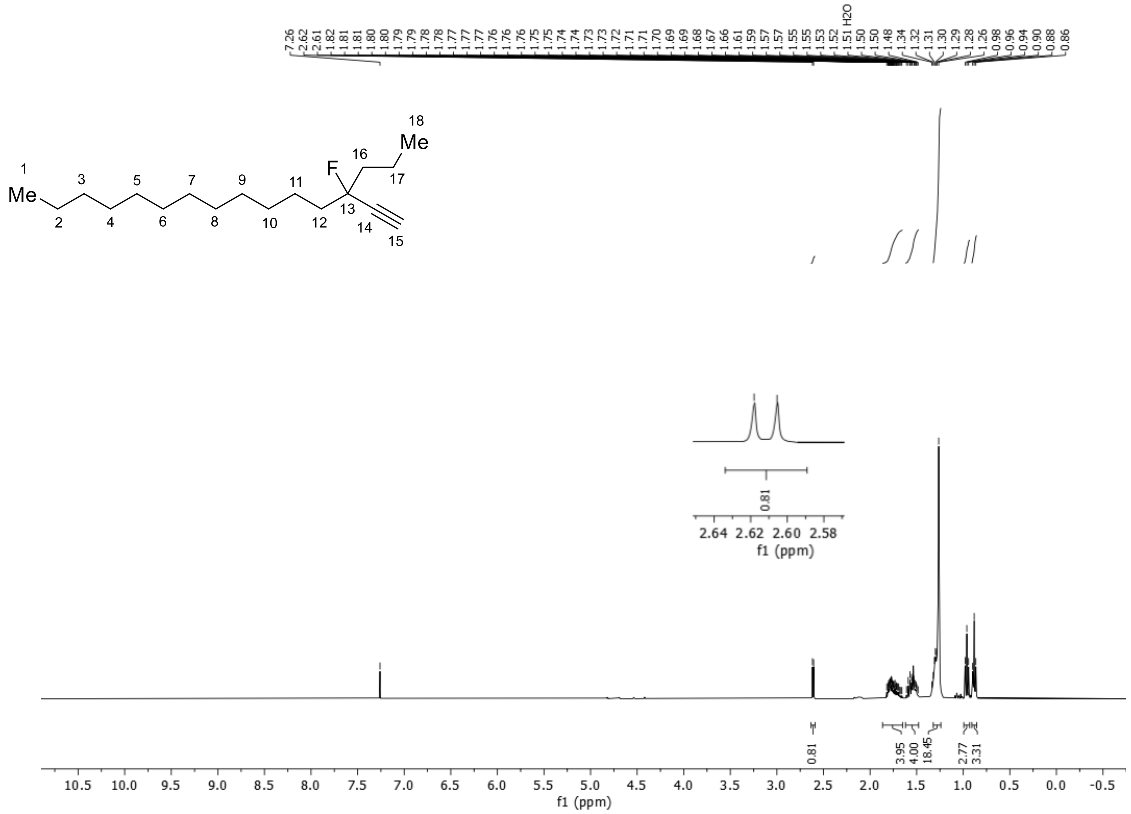

Supplementary Figure 169. ^{1}H NMR of **30** (400 MHz, 299 K, $CDCl_3$).

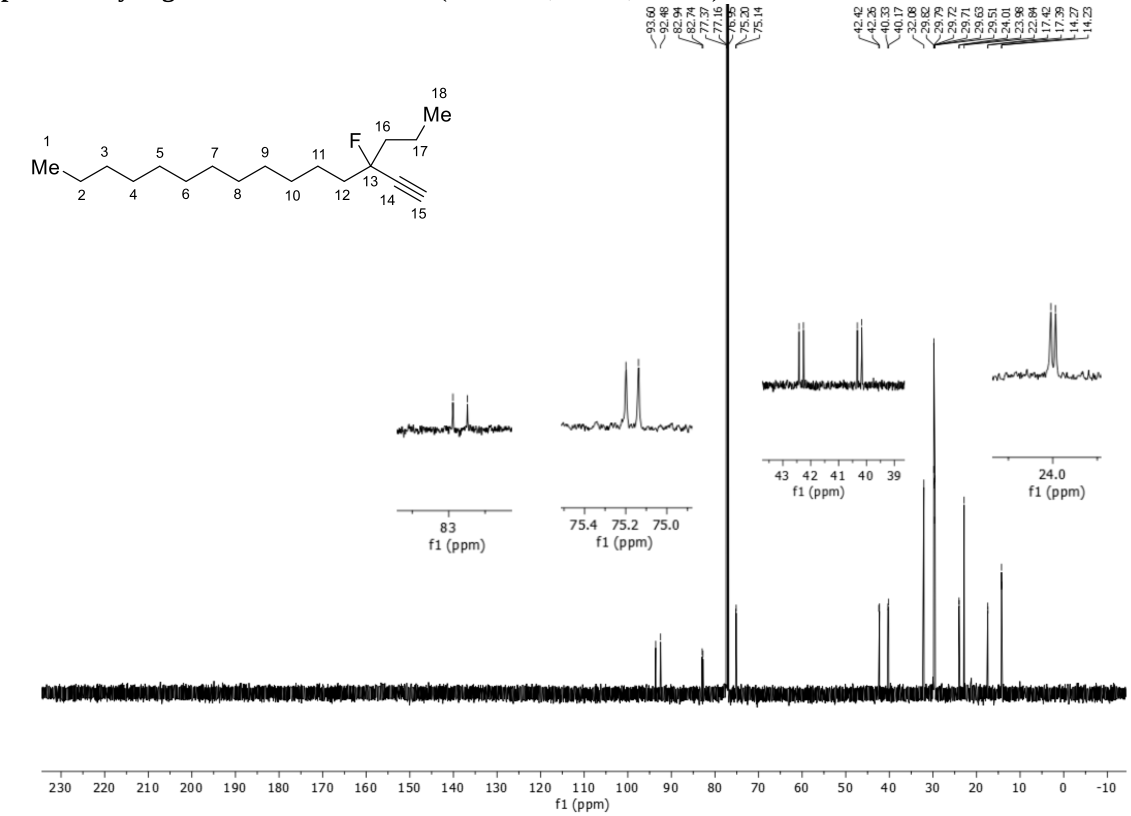

Supplementary Figure 170. $^{13}C\{^{1}H\}$ NMR of **30** (151 MHz, 299 K, $CDCl_3$).

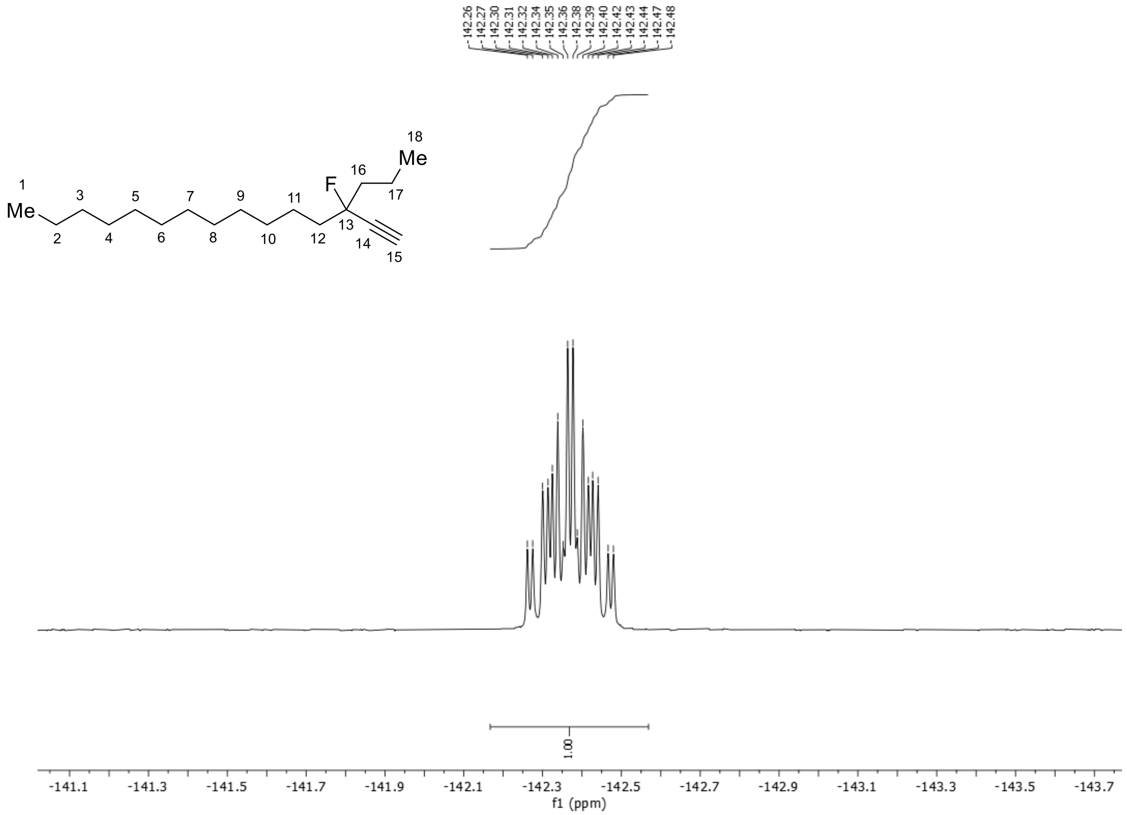

Supplementary Figure 171. ^{19}F NMR of **30** (376 MHz, 299 K, $CDCl_3$).

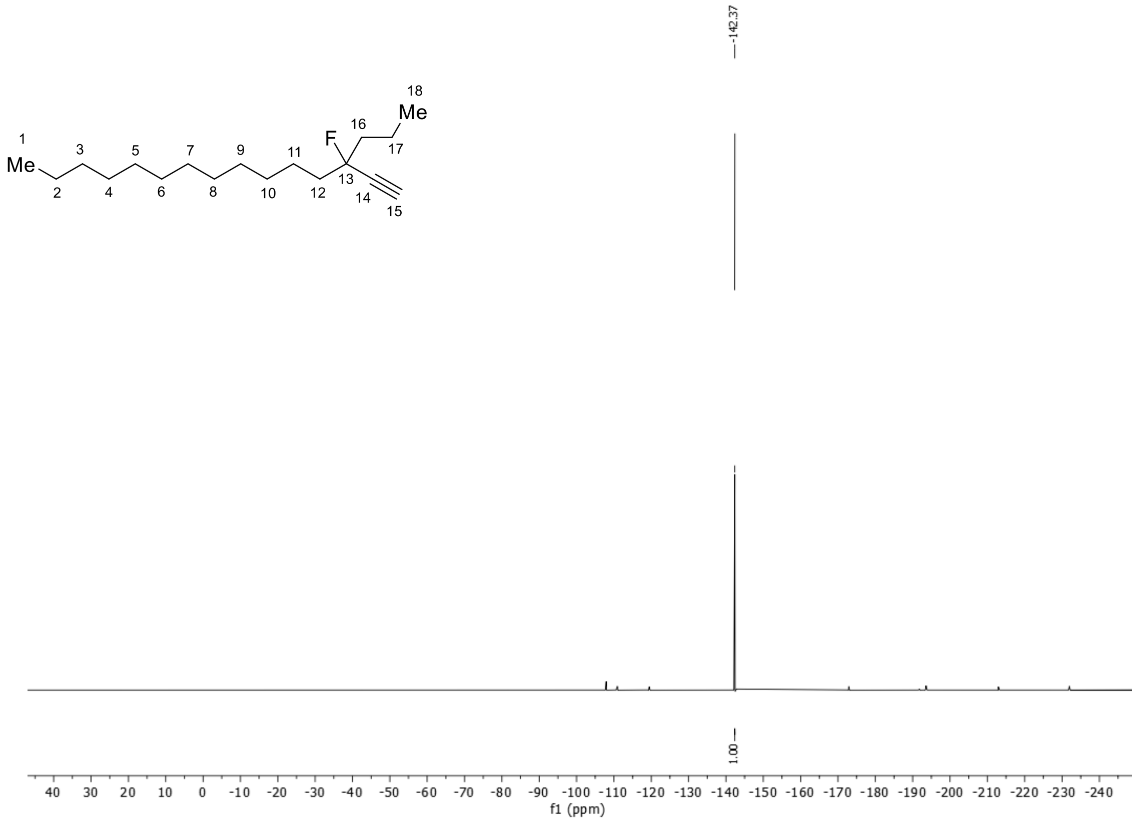

Supplementary Figure 172. $^{19}F\{^1H\}$ NMR of **30** (376 MHz, 299 K, $CDCl_3$).

(1S,2R,5S)-2-Isopropyl-5-methylcyclohexyl 4-((S)-1-chloro-2-fluorobut-3-yn-2-yl)benzoate (31)

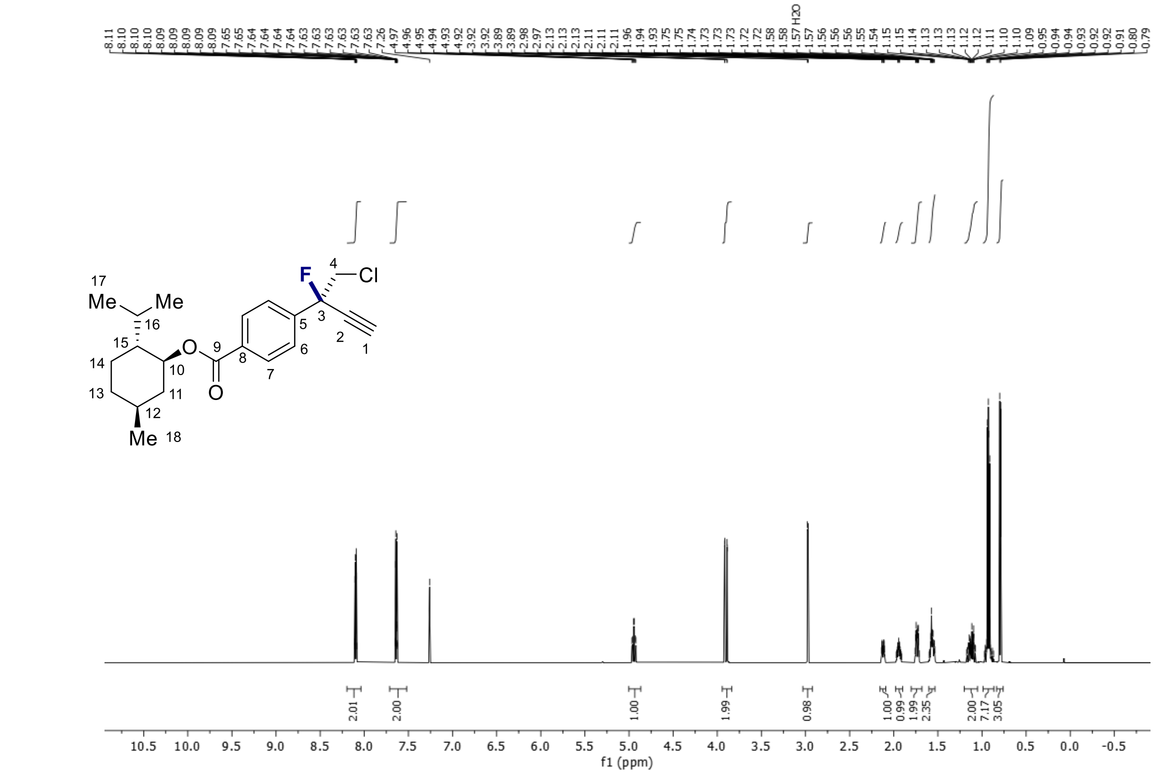

Supplementary Figure 173. ^{1}H NMR of **31** (599 MHz, 299 K, $CDCl_3$).

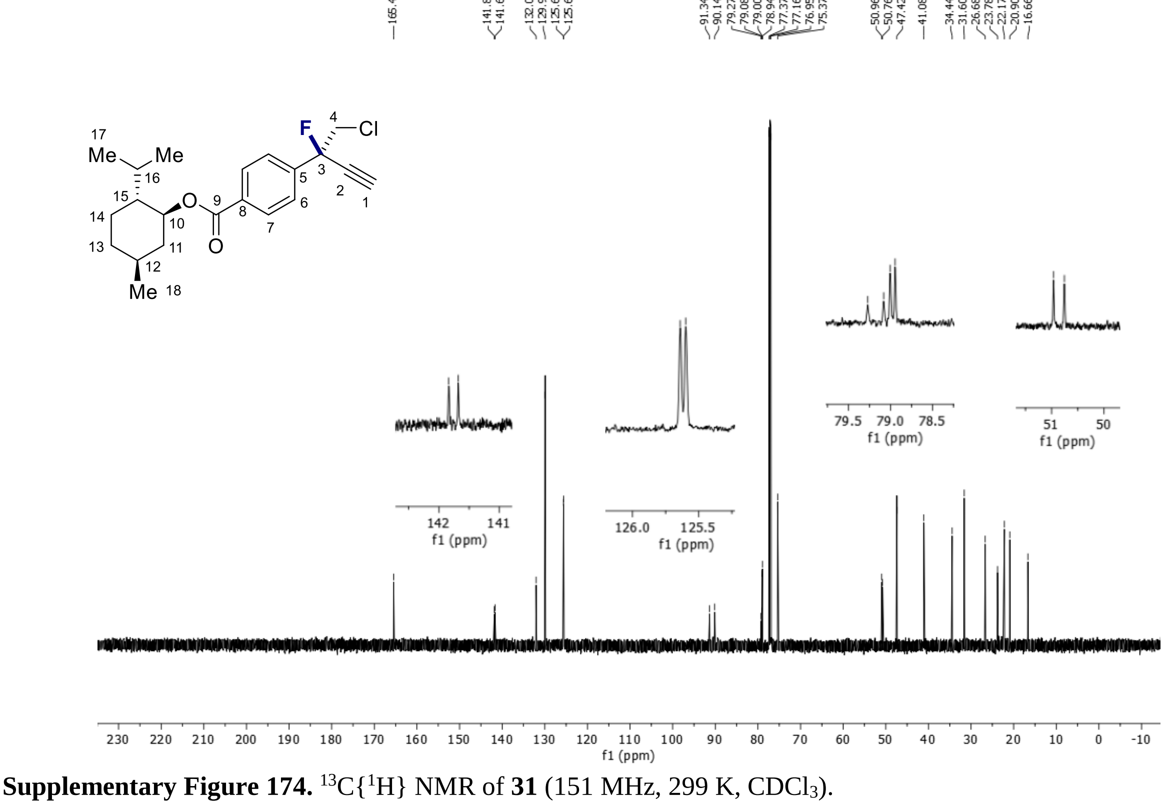

Supplementary Figure 174. ^{13}C{^{1}H} NMR of **31** (151 MHz, 299 K, $CDCl_3$).

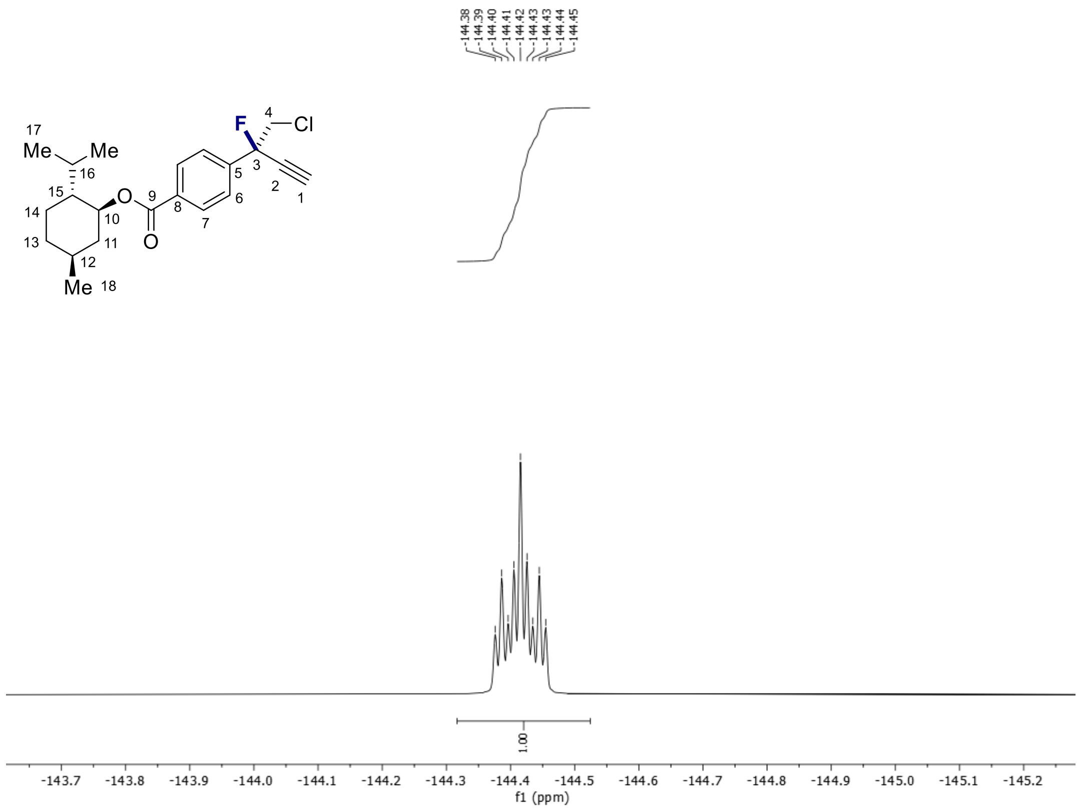

Supplementary Figure 175. ^{19}F NMR of **31** (564 MHz, 299 K, $CDCl_3$).

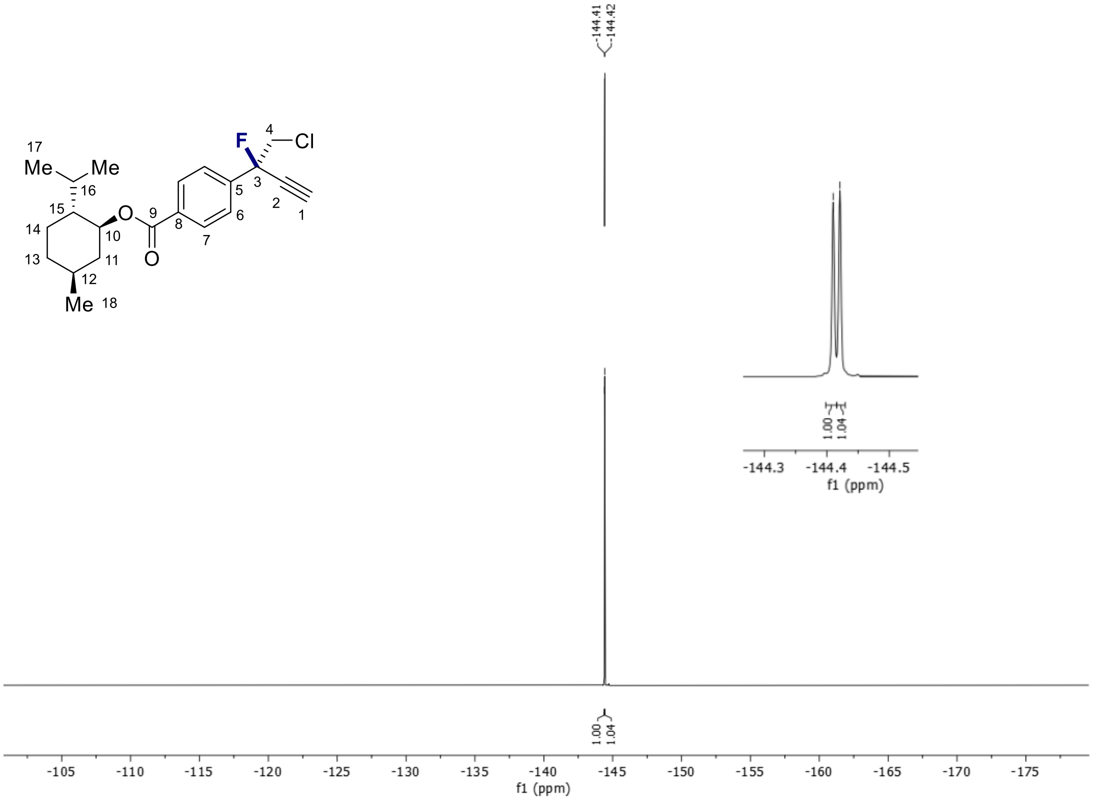

Supplementary Figure 176. ^{19}F{^{1}H} NMR of **31** (564 MHz, 299 K, $CDCl_3$).

Methyl 4-(1-chloro-2-fluoro-4-phenylbut-3-yn-2-yl)benzoate (32)

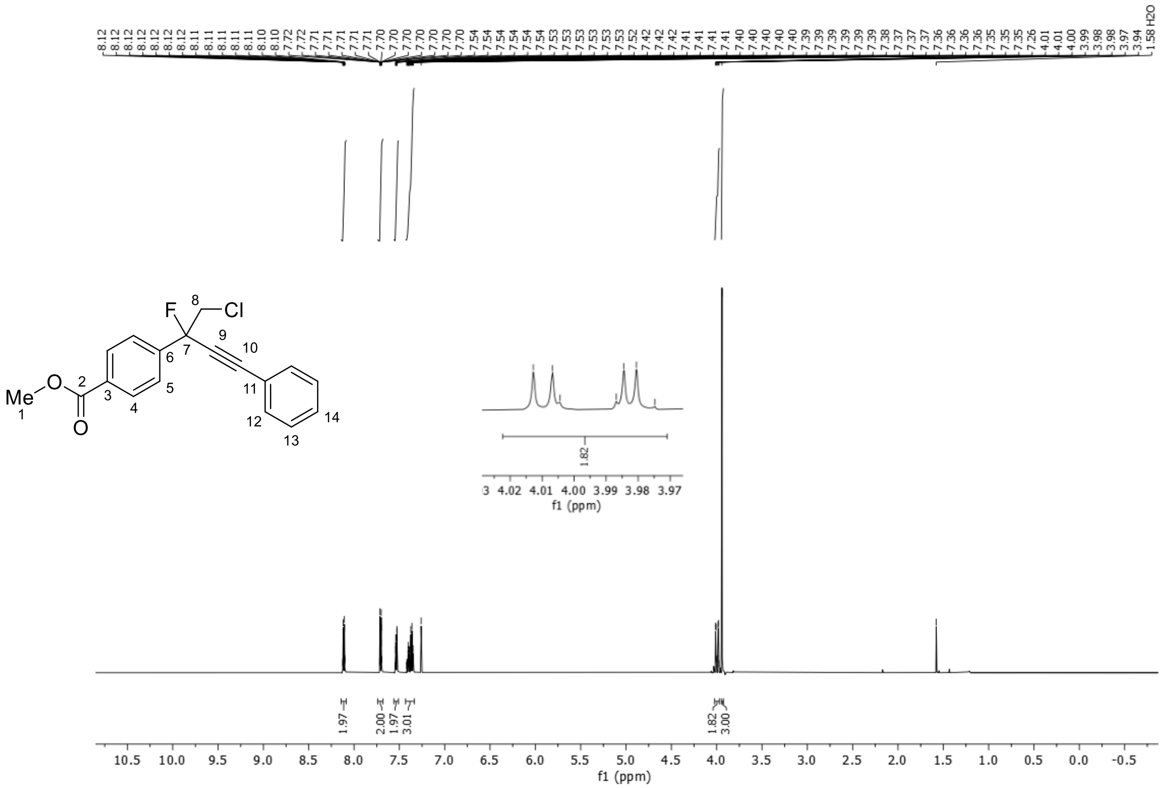

Supplementary Figure 177. ^{1}H NMR of **32** (599 MHz, 299 K, $CDCl_3$).

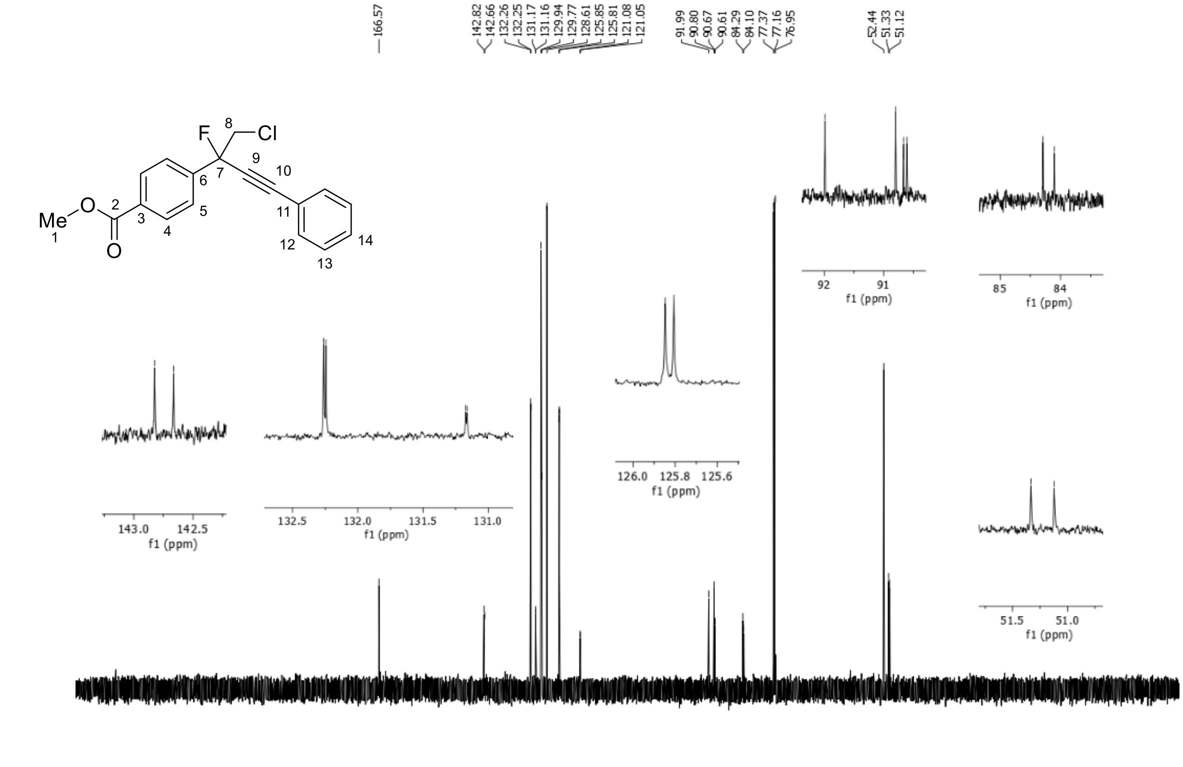

Supplementary Figure 178. $^{13}C\{^{1}H\}$ NMR of **32** (151 MHz, 299 K, $CDCl_3$).

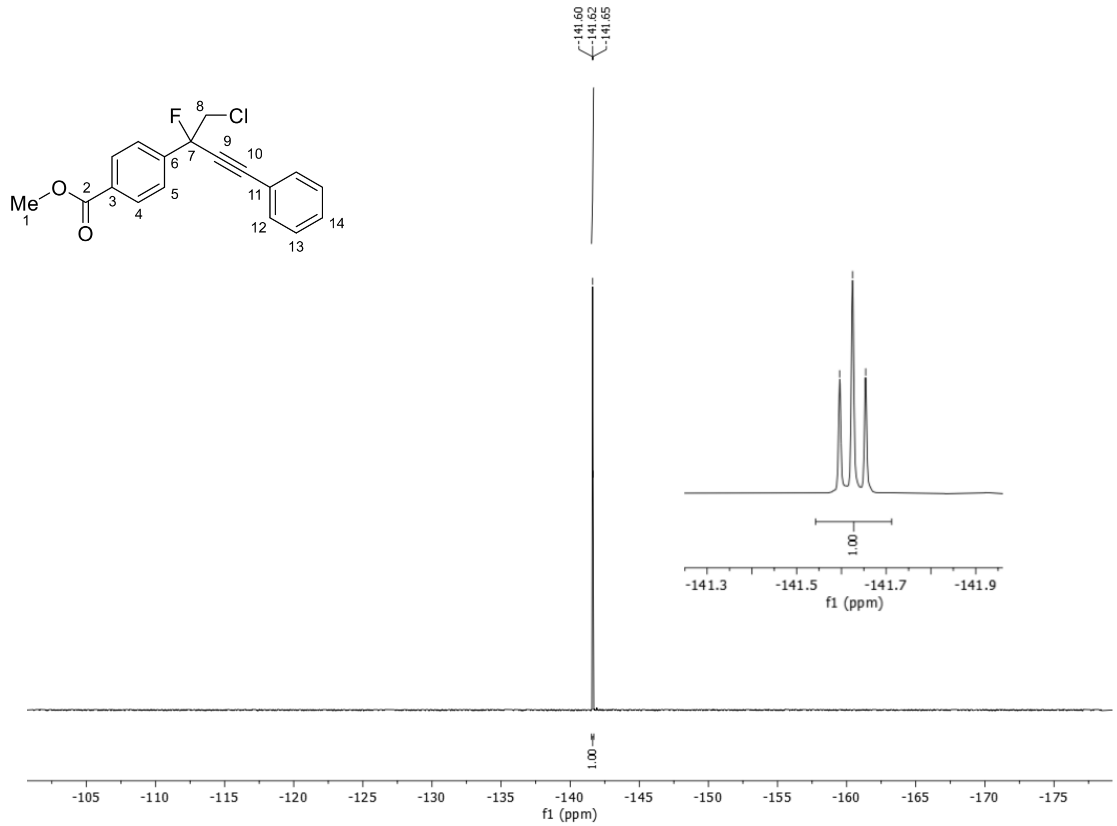

Supplementary Figure 179. ^{19}F NMR of **32** (564 MHz, 299 K, $CDCl_3$).

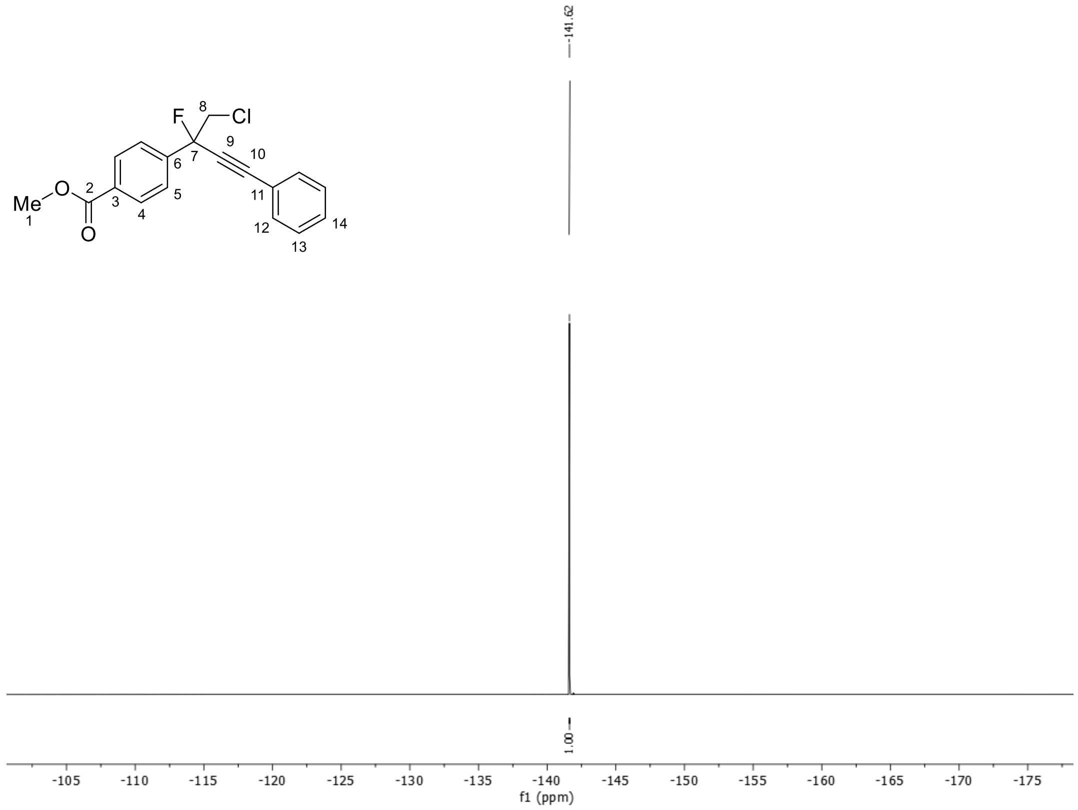

Supplementary Figure 180. $^{19}F\{^1H\}$ NMR of **32** (564 MHz, 299 K, $CDCl_3$).

Methyl 4-(1-chloro-2-fluoro-4-phenylbut-3-yn-2-yl)benzoate (33)

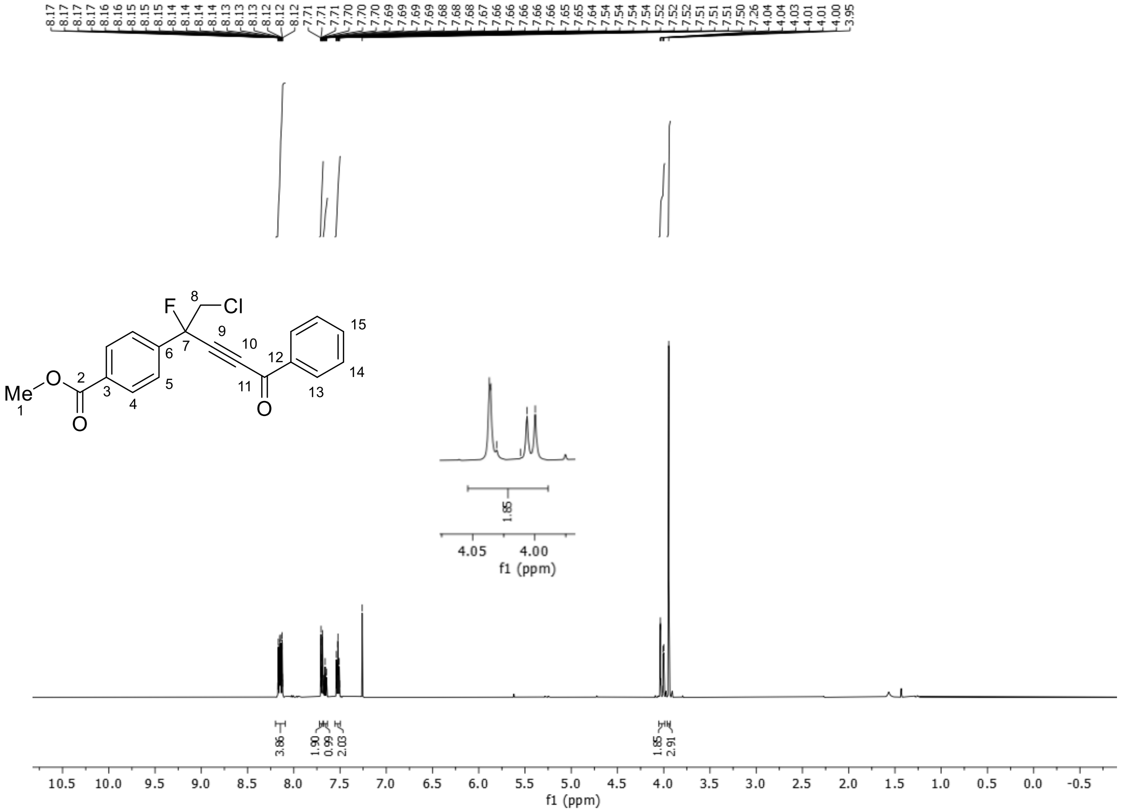

Supplementary Figure 181. ^{1}H NMR of **33** (500 MHz, 299 K, $CDCl_3$).

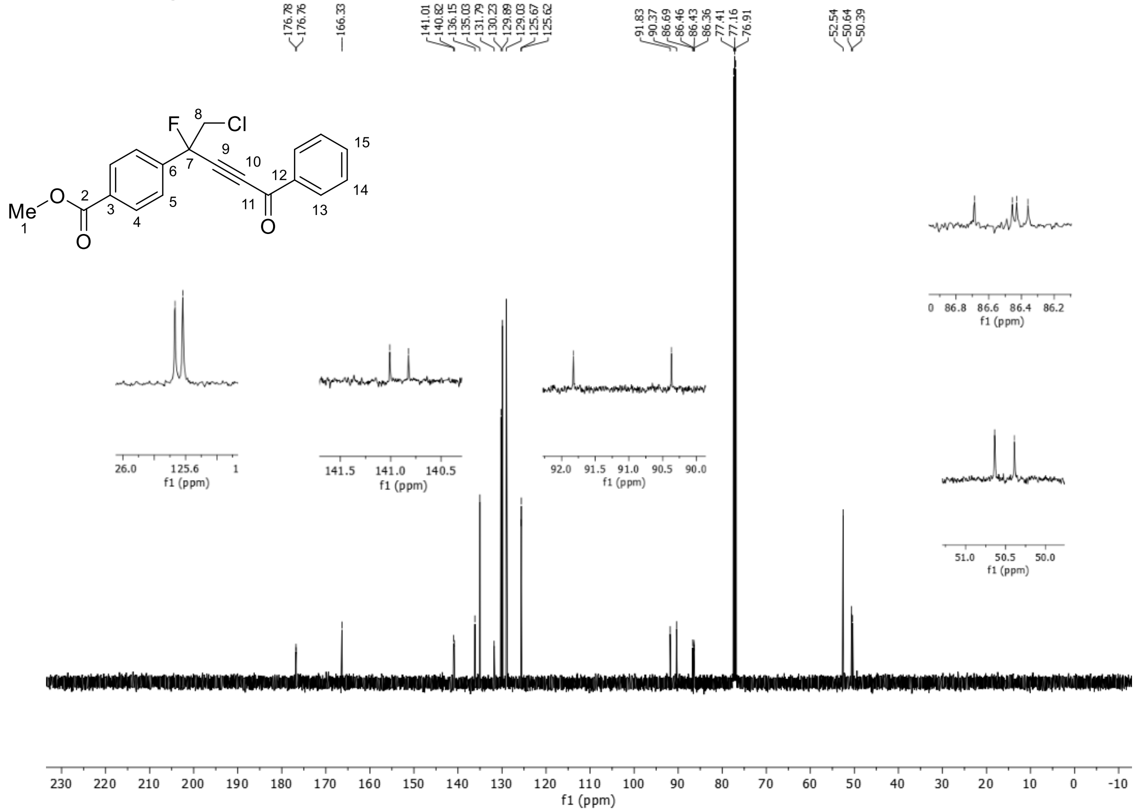

Supplementary Figure 182. $^{13}C\{^{1}H\}$ NMR of **33** (126 MHz, 299 K, $CDCl_3$).

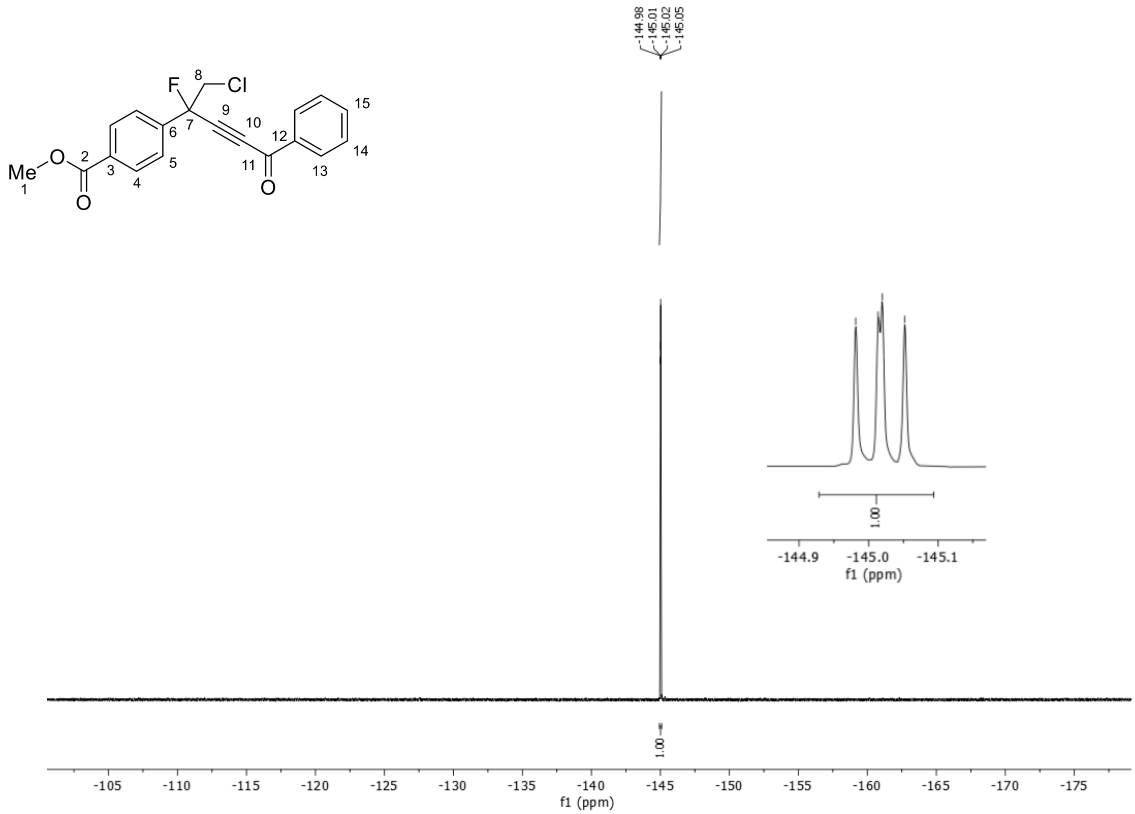

Supplementary Figure 183. ^{19}F NMR of **33** (470 MHz, 299 K, $CDCl_3$).

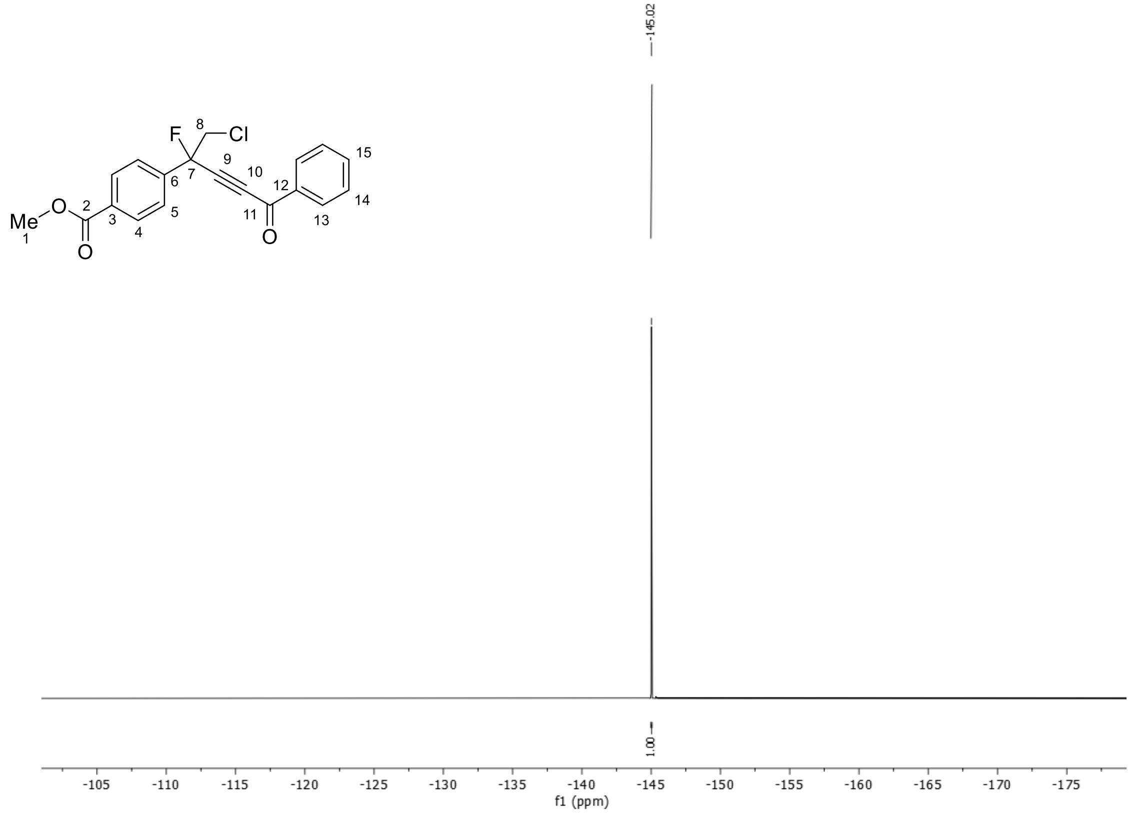

Supplementary Figure 184. ^{19}F{^{1}H} NMR of **33** (470 MHz, 299 K, $CDCl_3$).

3-Fluoropentadecane (34)

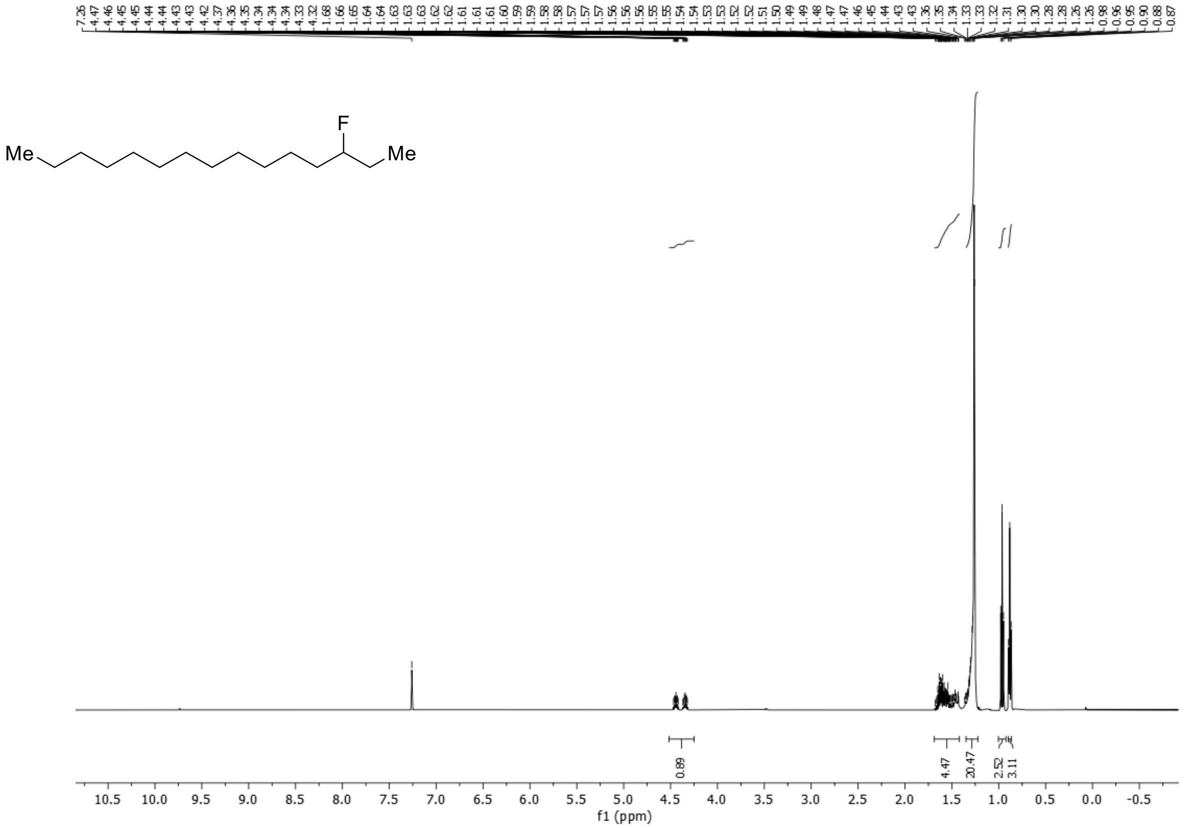

Supplementary Figure 185. ^{1}H NMR of **34** (500 MHz, 299 K, $CDCl_3$).

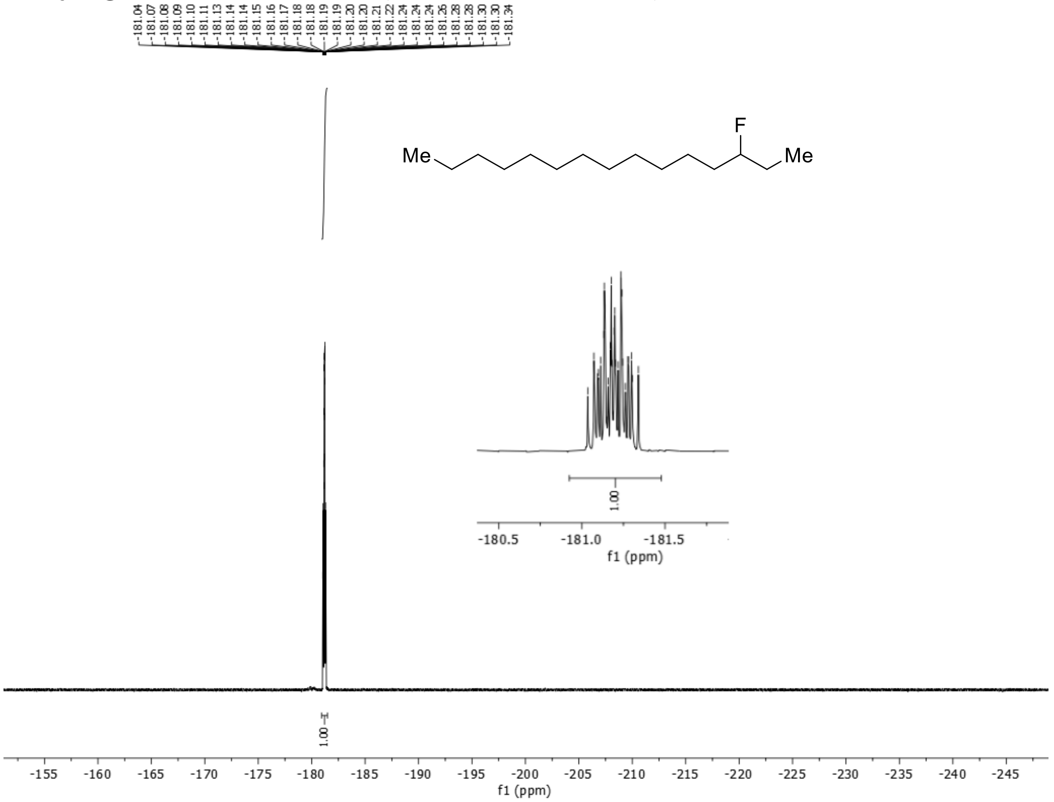

Supplementary Figure 186. ^{19}F NMR of **34** (470 MHz, 299 K, $CDCl_3$).

3-Fluoropentadec-1-ene (35)

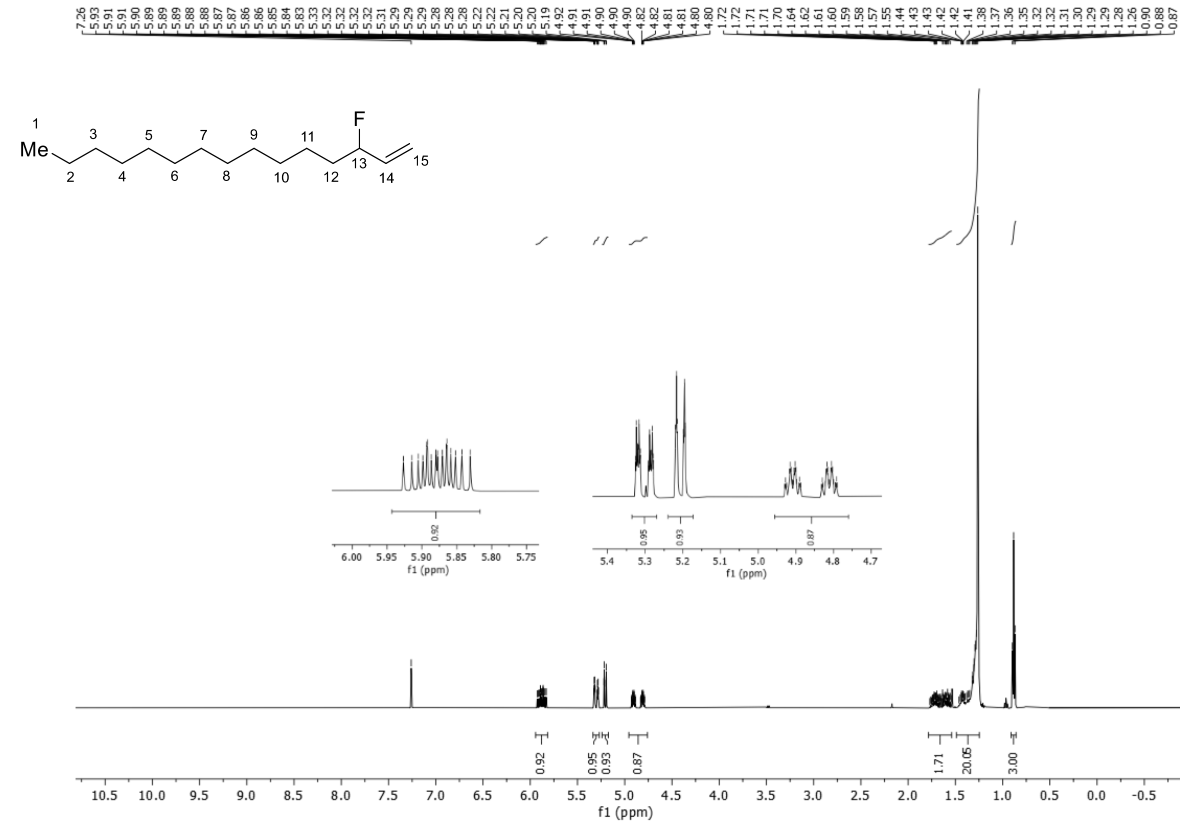

Supplementary Figure 187. ^{1}H NMR of **35** (500 MHz, 299 K, $CDCl_3$).

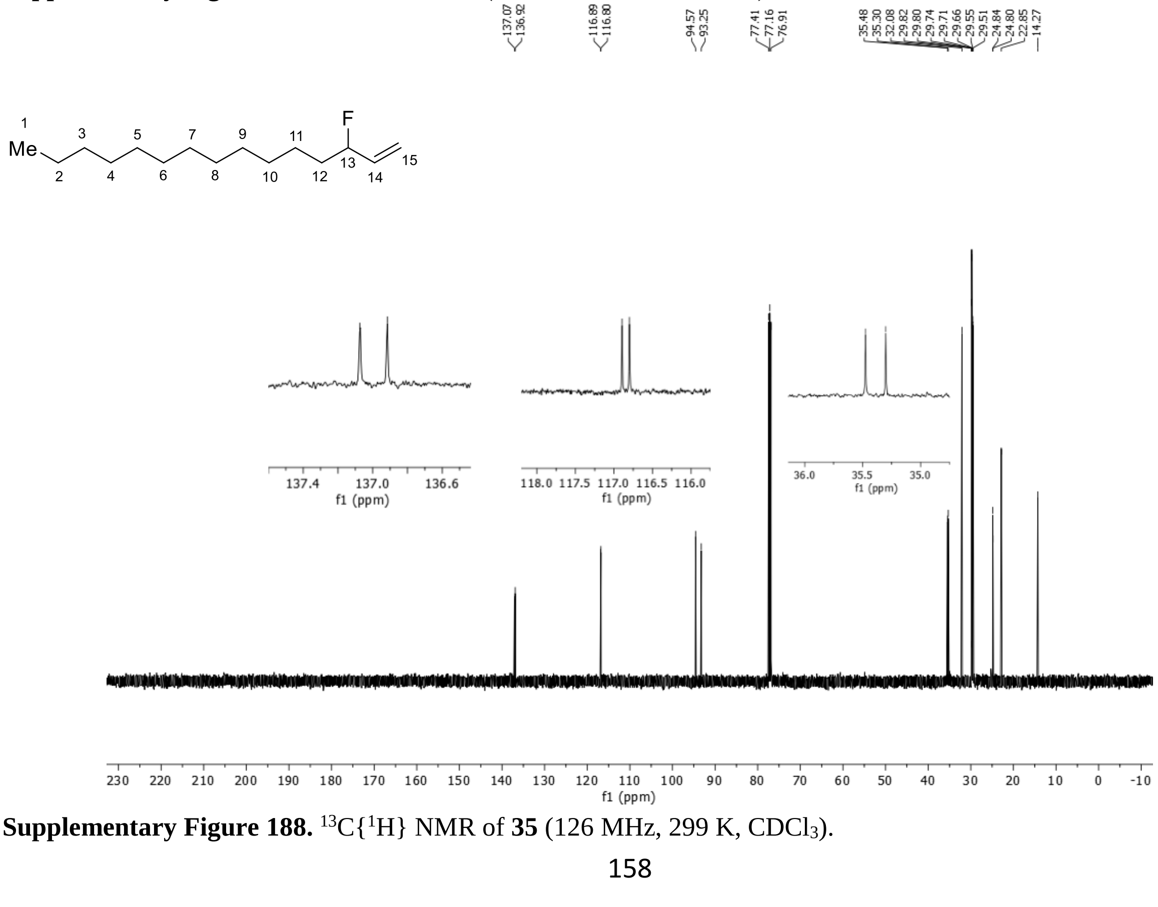

Supplementary Figure 188. $^{13}C\{^{1}H\}$ NMR of **35** (126 MHz, 299 K, $CDCl_3$).

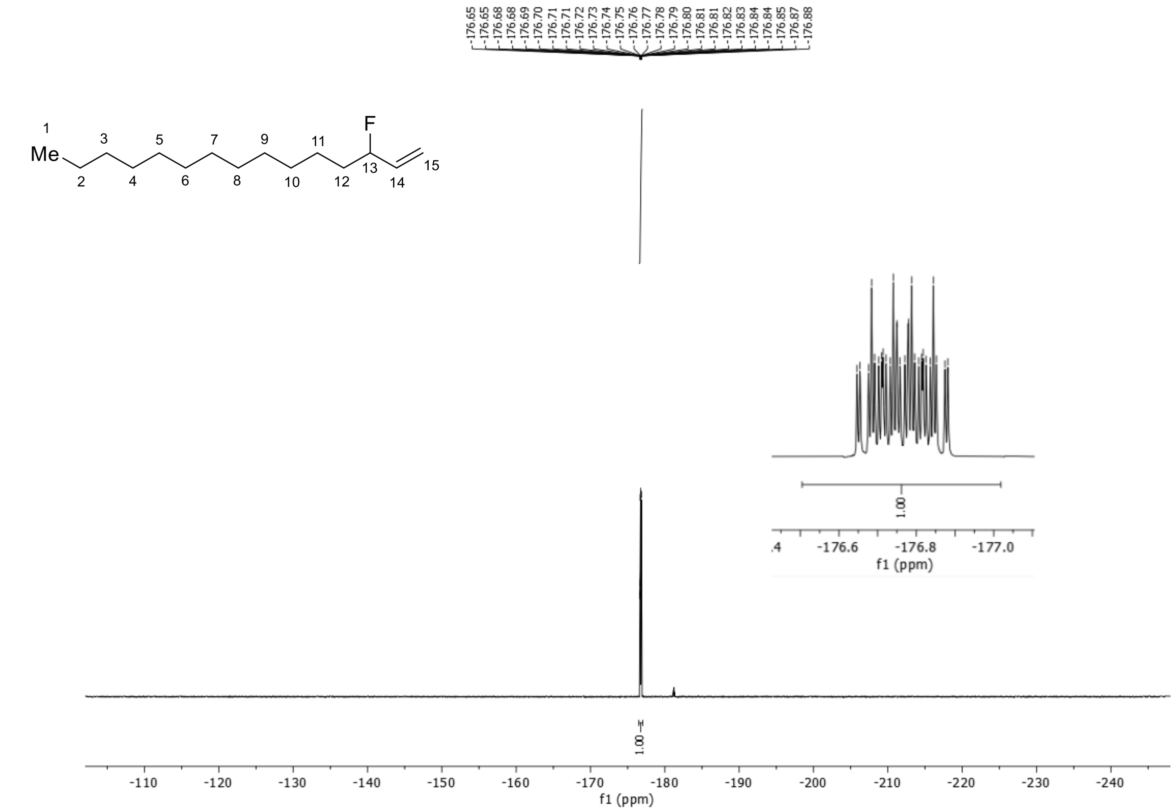

Supplementary Figure 189. ^{19}F NMR of **35** (470 MHz, 299 K, $CDCl_3$).

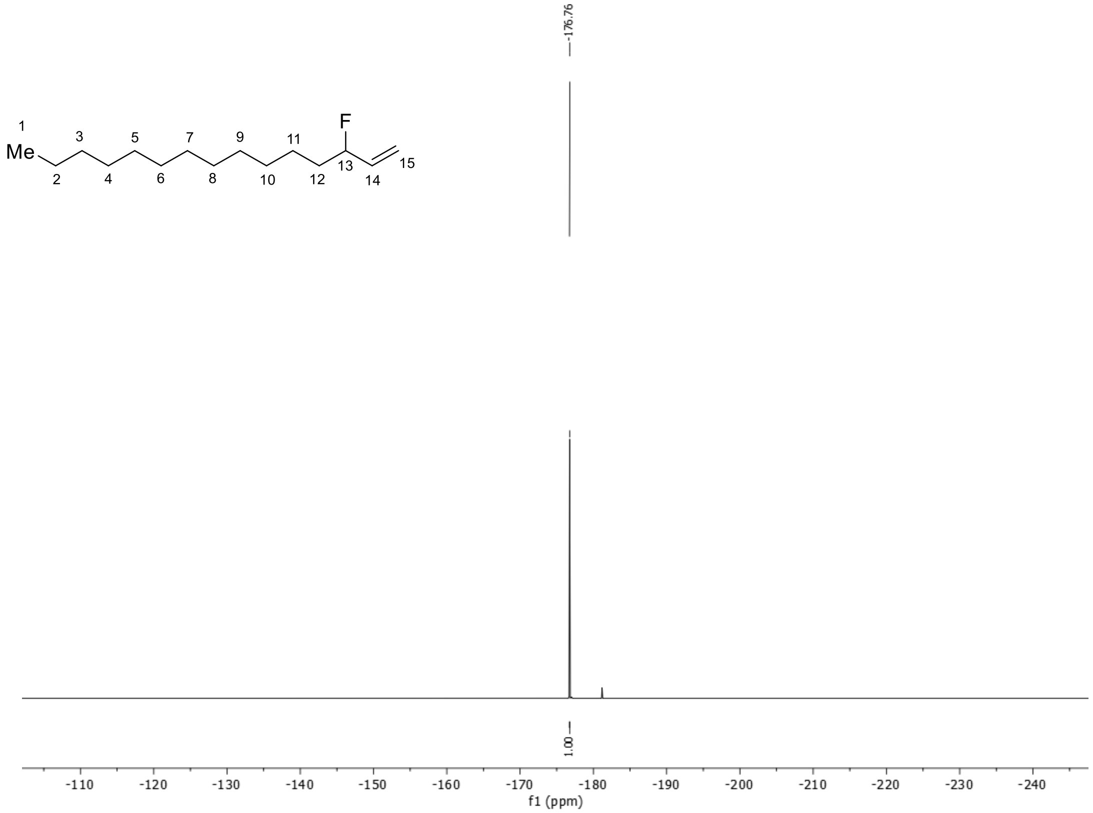

Supplementary Figure 190. $^{19}F\{^1H\}$ NMR of **35** (470 MHz, 299 K, $CDCl_3$).

3-Fluoropentadecan-2-one (36)

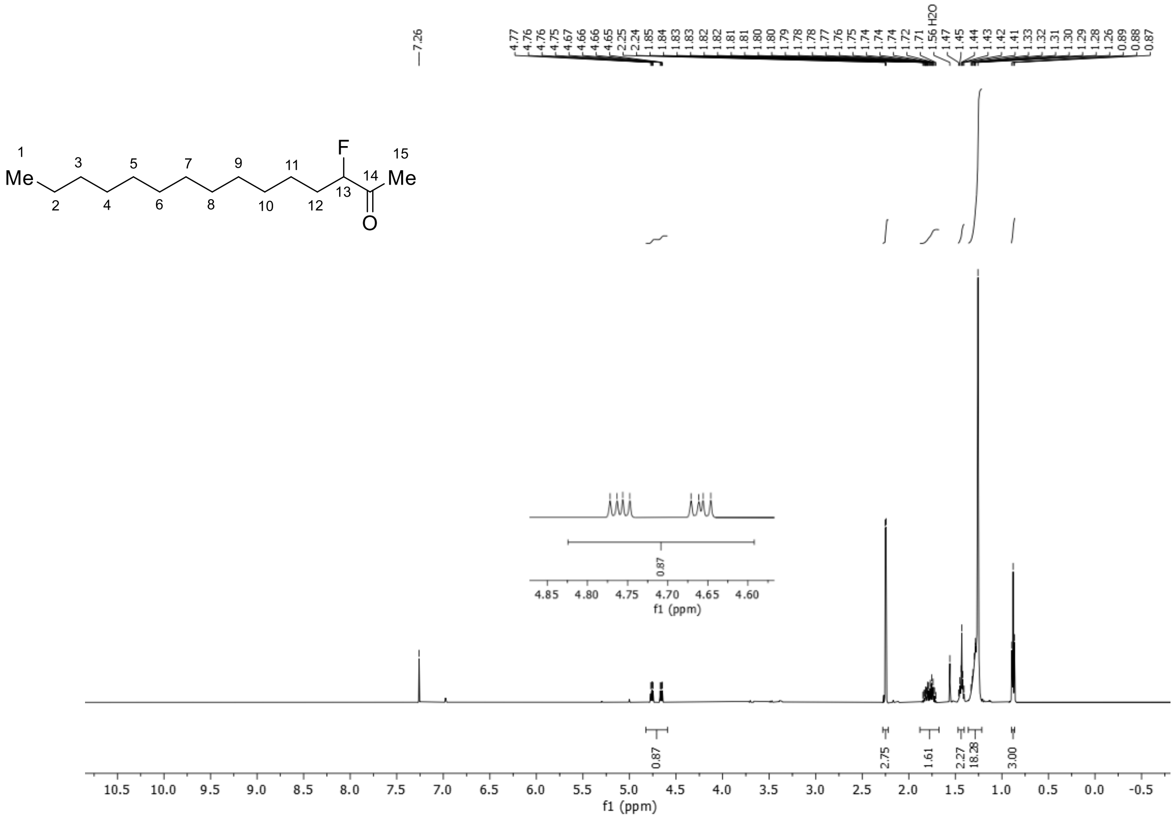

Supplementary Figure 191. ^{1}H NMR of **36** (500 MHz, 299 K, $CDCl_3$).

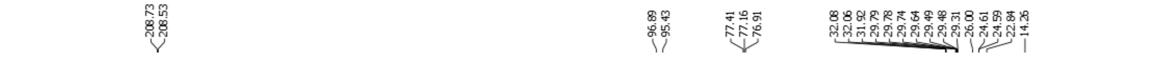

Supplementary Figure 192. $^{13}C\{^{1}H\}$ NMR of **36** (126 MHz, 299 K, $CDCl_3$).

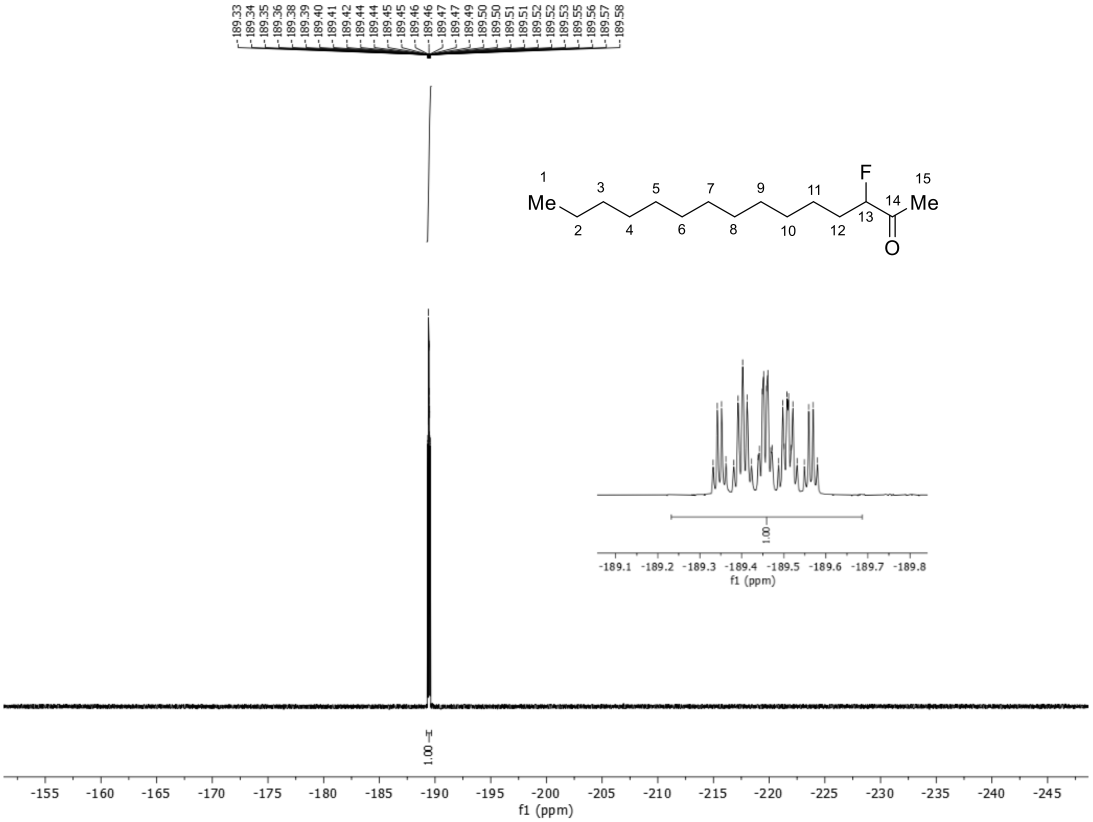

Supplementary Figure 193. ^{19}F NMR of **36** (470 MHz, 299 K, $CDCl_3$).

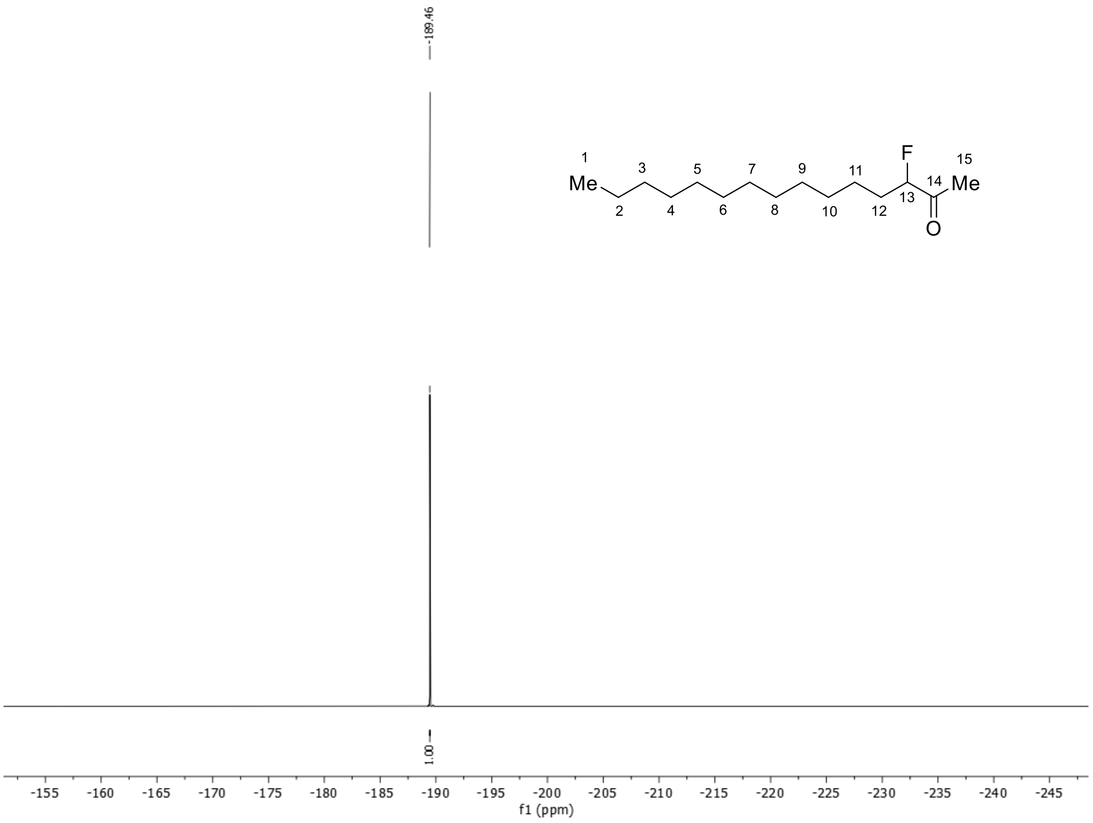

Supplementary Figure 194. ^{19}F{^{1}H} NMR of **36** (470 MHz, 299 K, $CDCl_3$).

N-(4-((4-(1-fluorotridecyl)-1H-1,2,3-triazol-1-yl)sulfonyl)phenyl)acetamide (37)

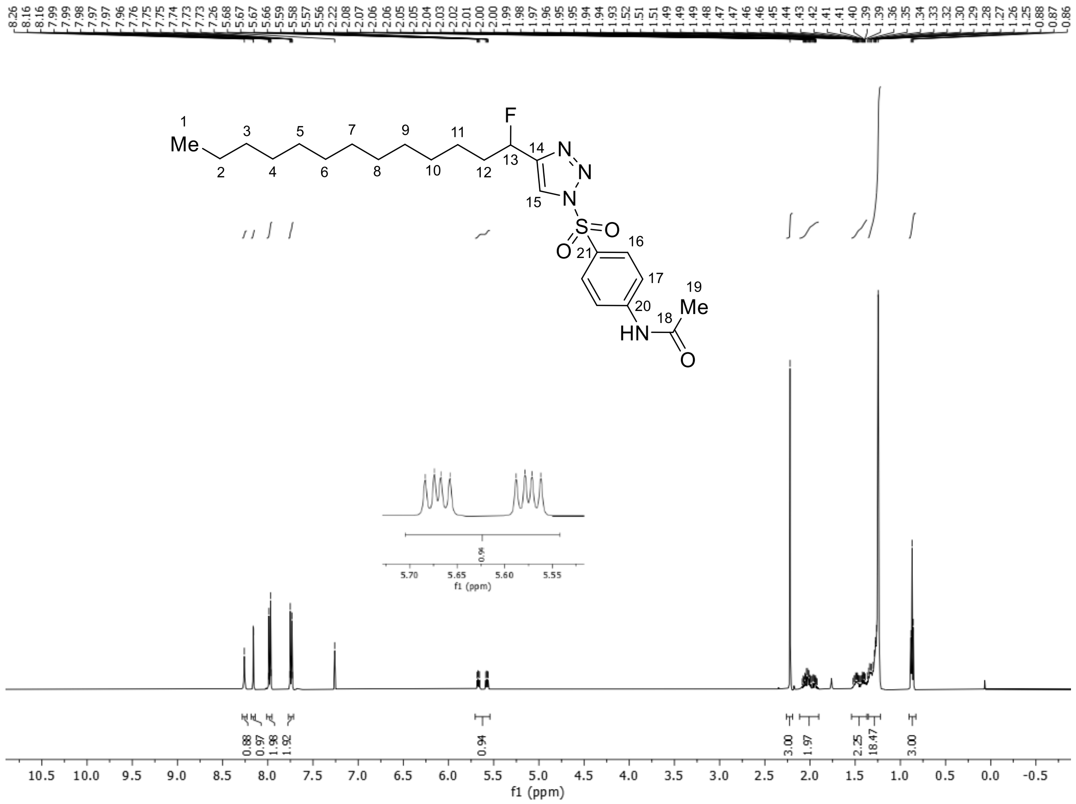

Supplementary Figure 195. ^{1}H NMR of **37** (500 MHz, 299 K, $CDCl_3$).

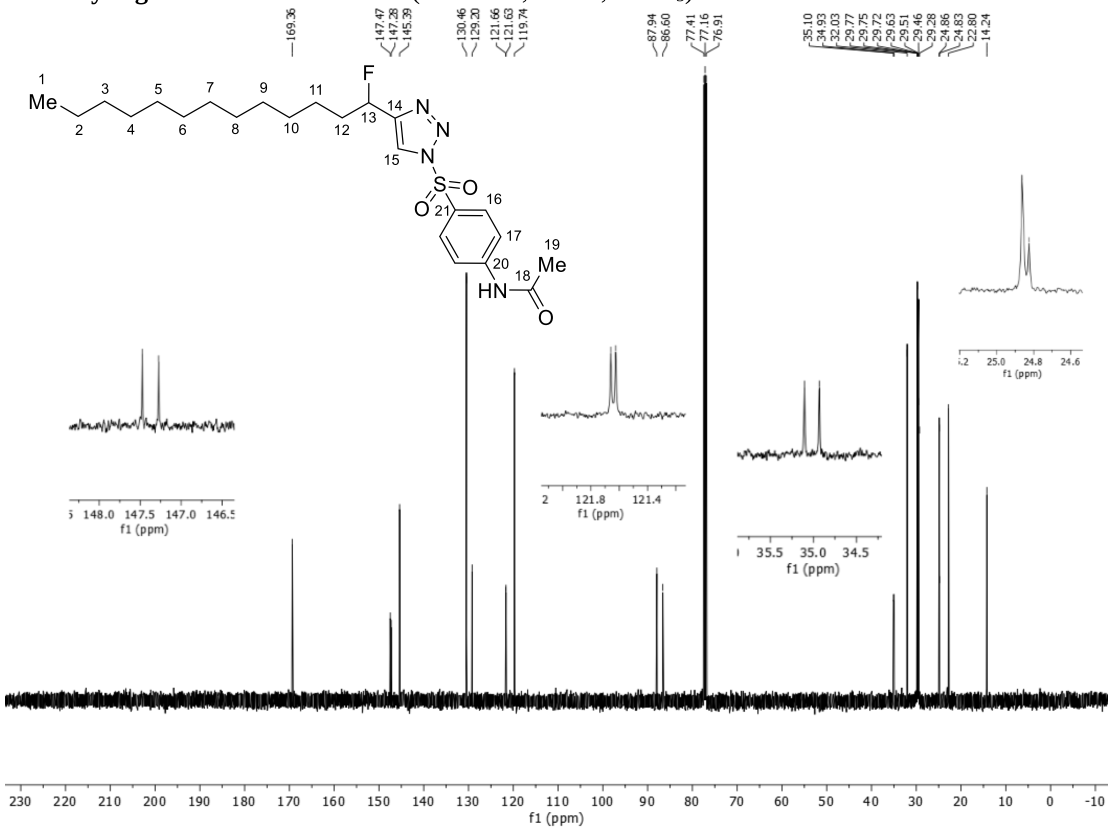

Supplementary Figure 196. $^{13}C\{^{1}H\}$ NMR of **37** (126 MHz, 299 K, $CDCl_3$).

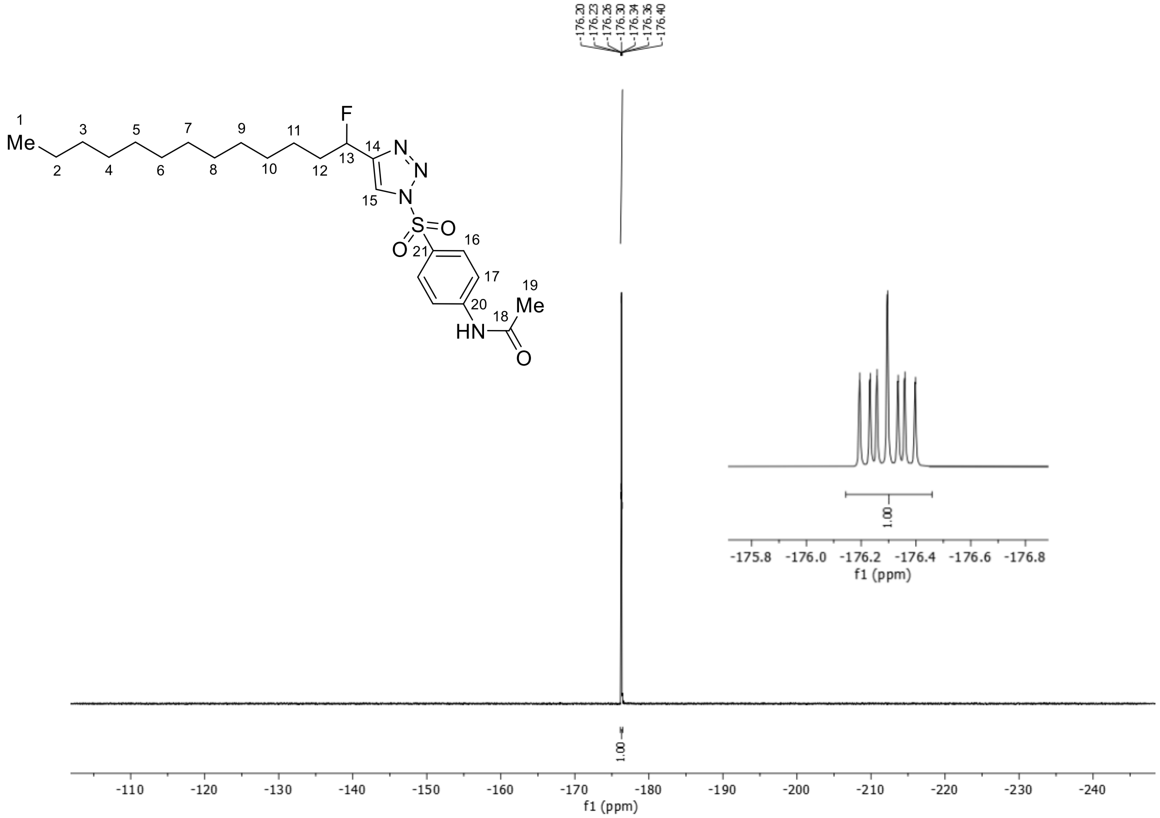

Supplementary Figure 197. ^{19}F NMR of **37** (470 MHz, 299 K, $CDCl_3$).

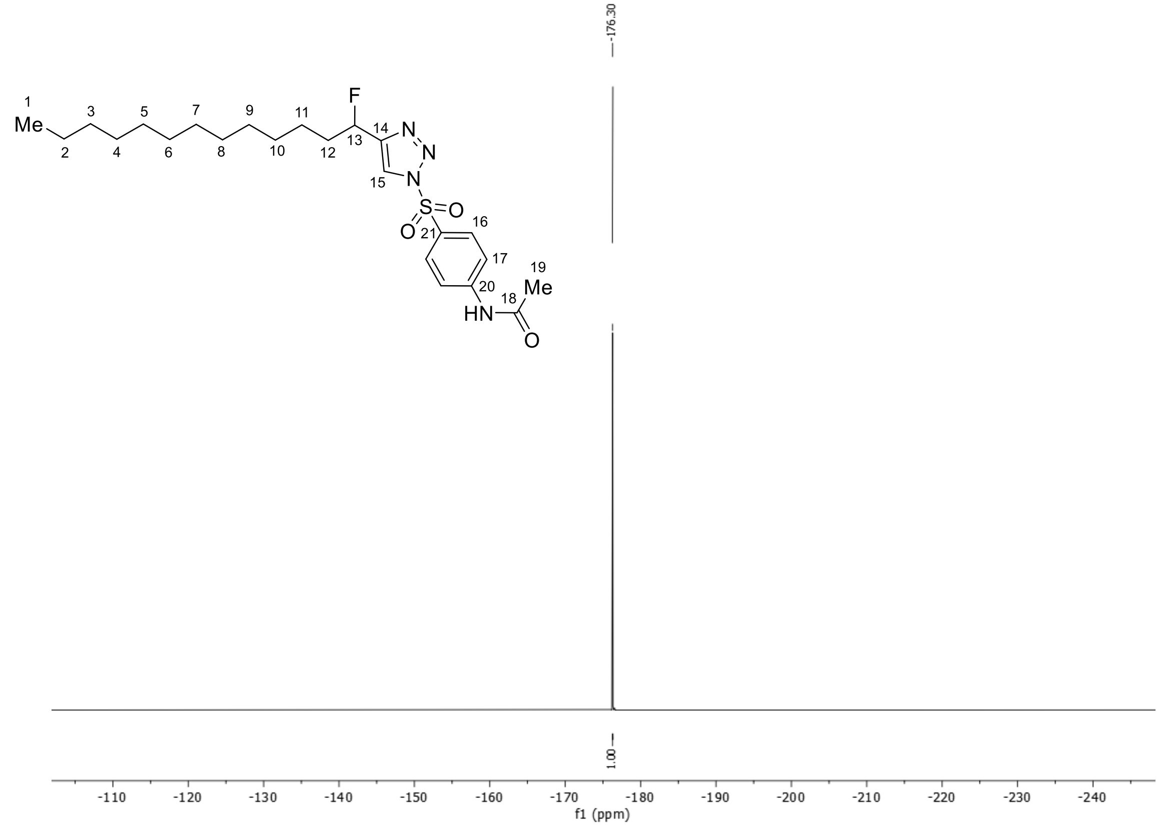

Supplementary Figure 198. $^{19}F\{^{1}H\}$ NMR of **37** (470 MHz, 299 K, $CDCl_3$).

4-(Chloromethyl)-4-fluorohexadeca-1,2-diene (38)

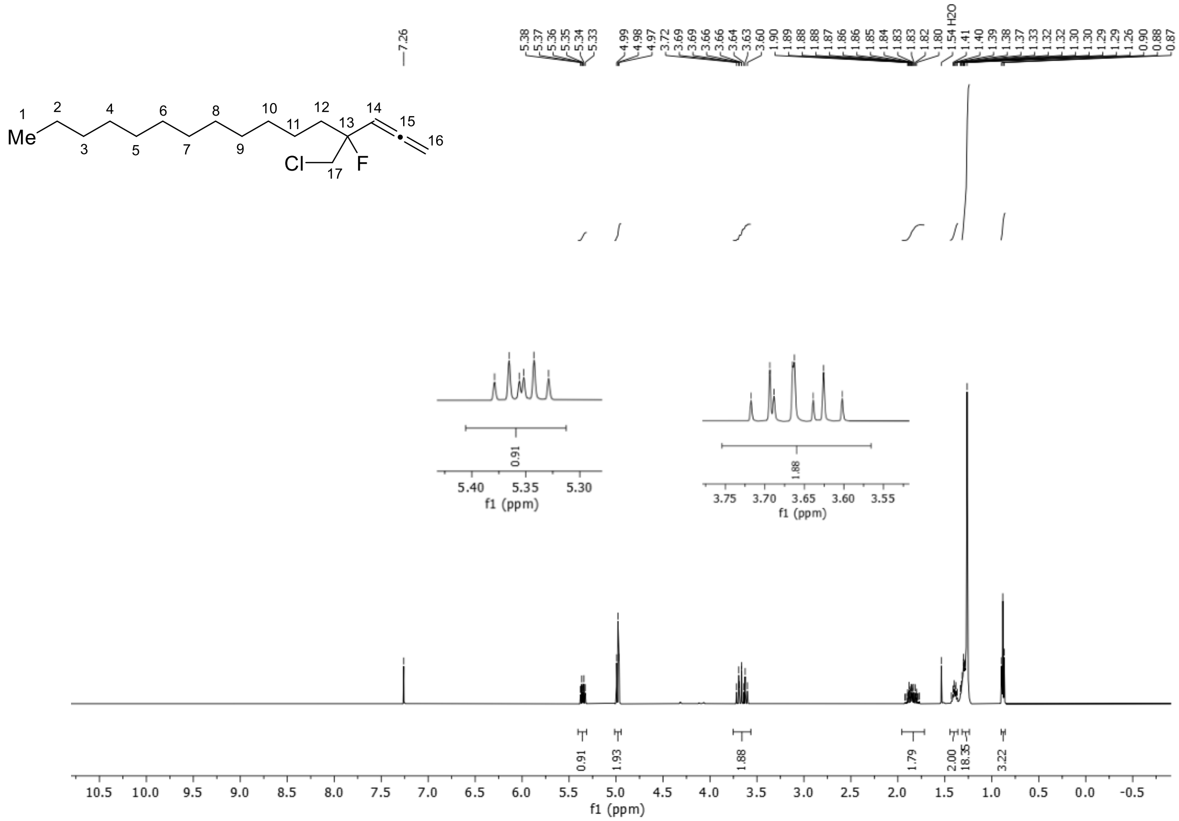

Supplementary Figure 199. ^{1}H NMR of **38** (500 MHz, 299 K, $CDCl_3$).

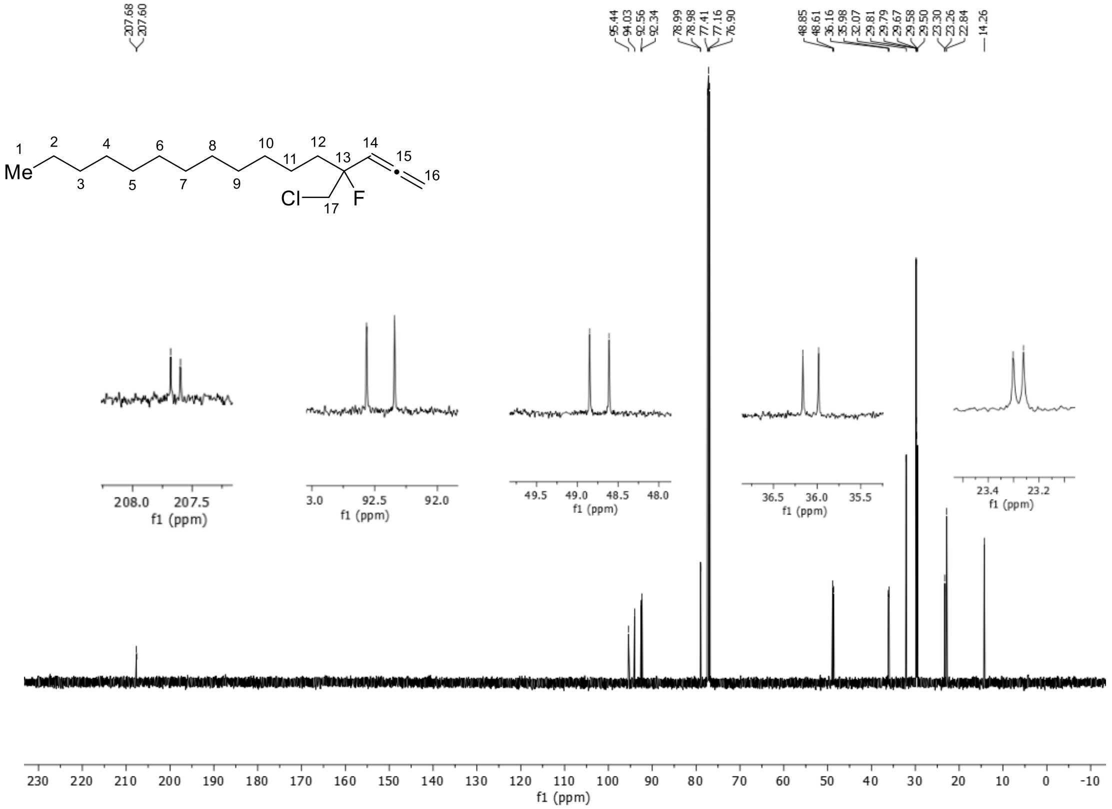

Supplementary Figure 200. ^{13}C{^{1}H} NMR of **38** (126 MHz, 299 K, $CDCl_3$).

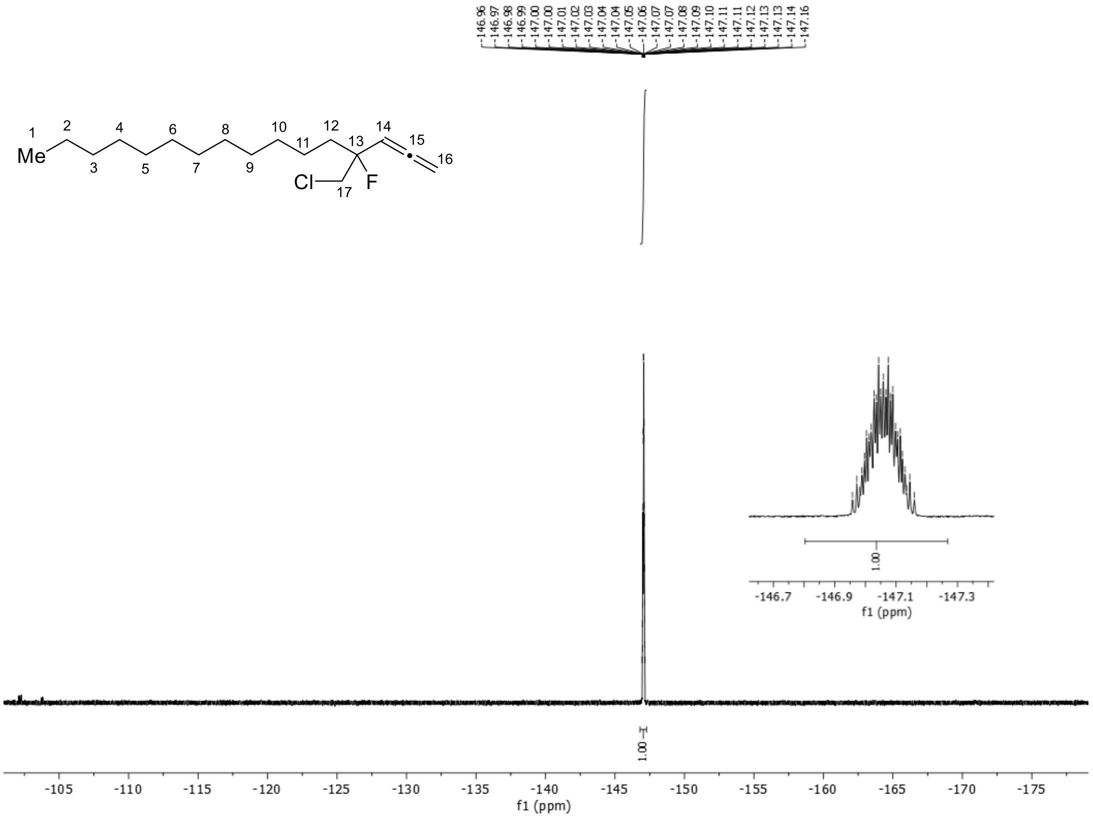

Supplementary Figure 201. ^{19}F NMR of **38** (470 MHz, 299 K, $CDCl_3$).

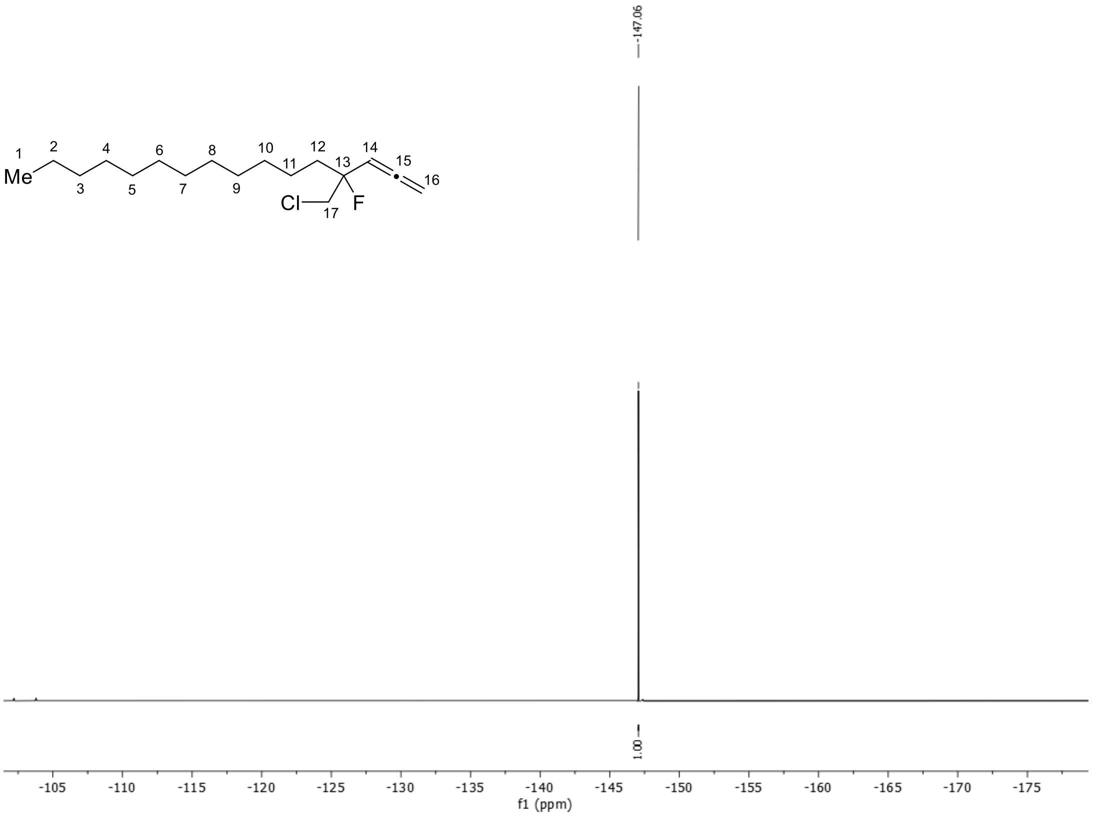

Supplementary Figure 202. $^{19}F\{^1H\}$ NMR of **38** (470 MHz, 299 K, $CDCl_3$).

(*Z*)-4-(chloromethyl)-1,2,4-trifluorohexadec-2-ene (39)

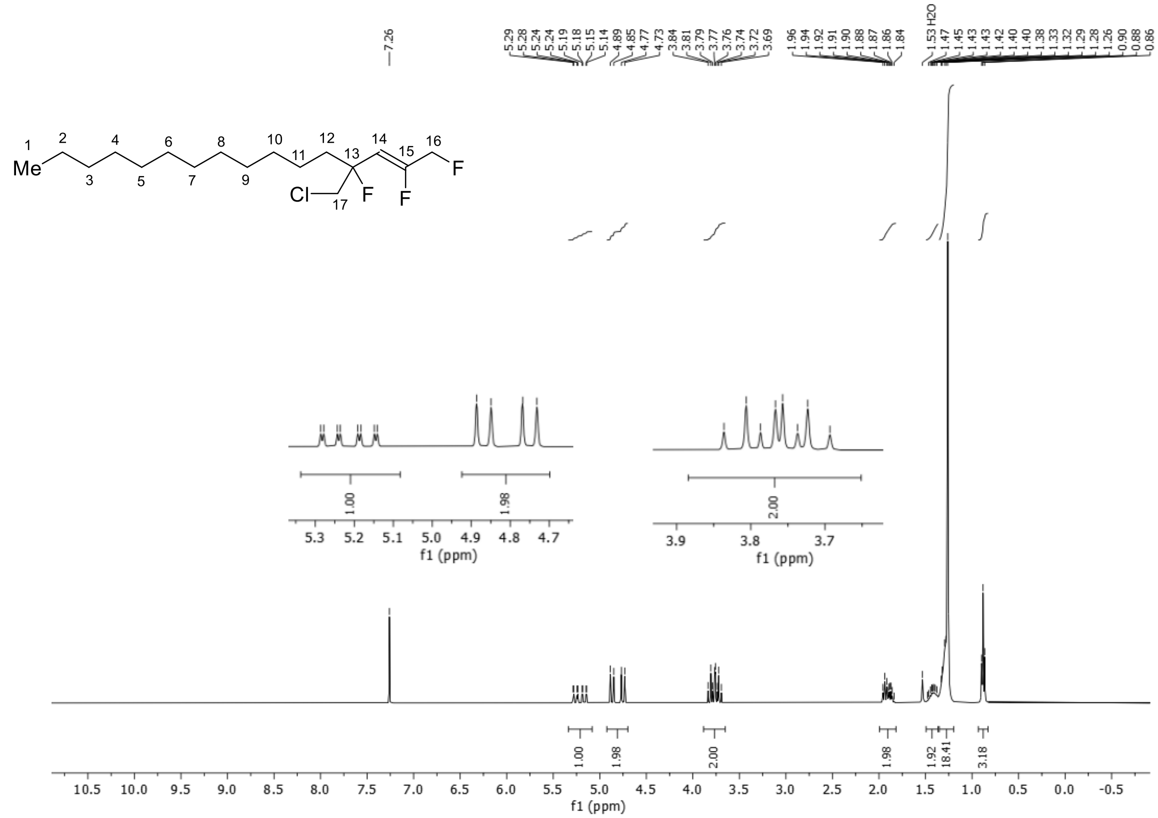

Supplementary Figure 203. ^{1}H NMR of **39** (400 MHz, 299 K, $CDCl_3$).

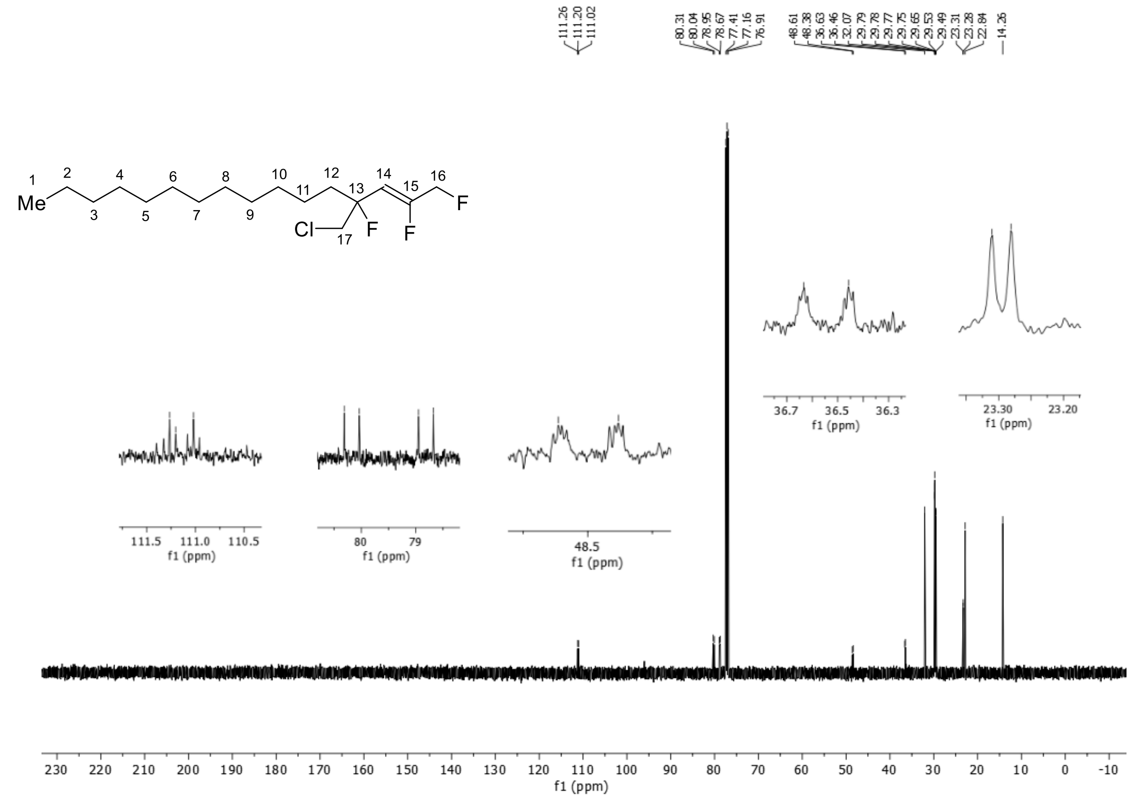

Supplementary Figure 204. $^{13}C\{^{1}H\}$ NMR of **39** (126 MHz, 299 K, $CDCl_3$).

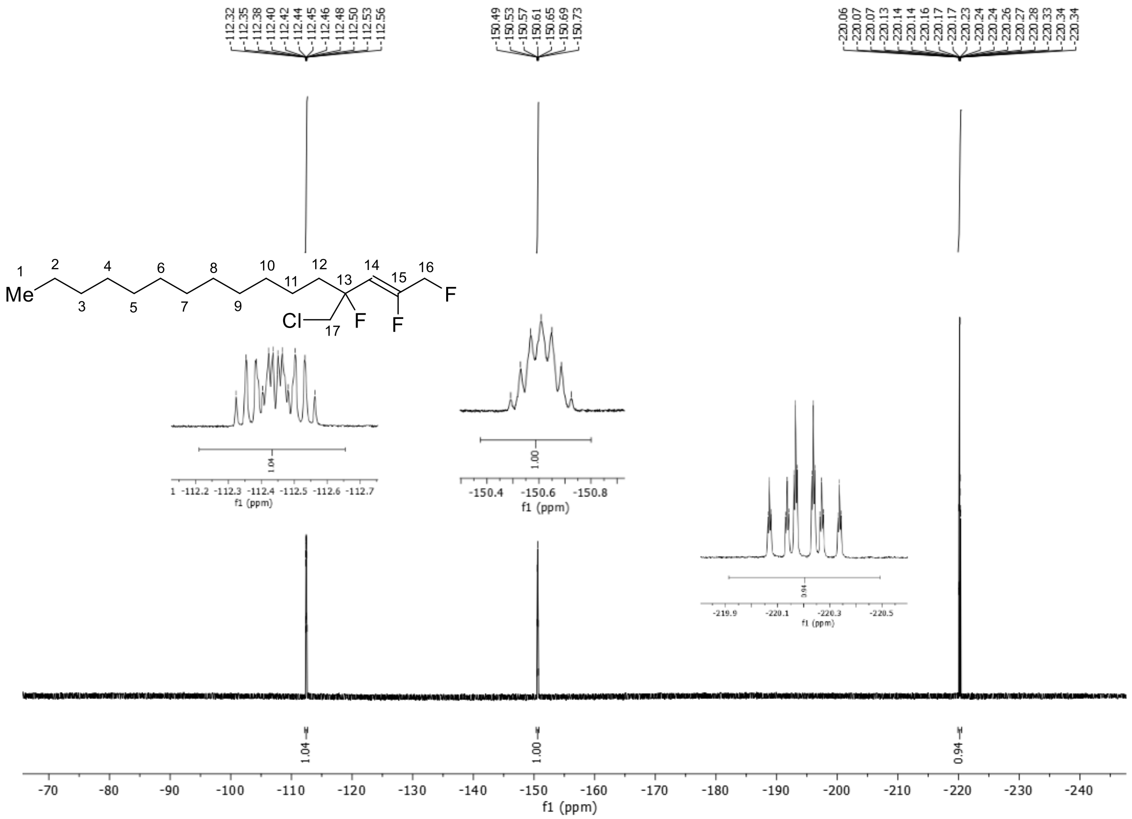

Supplementary Figure 205. ^{19}F NMR of **39** (470 MHz, 299 K, $CDCl_3$).

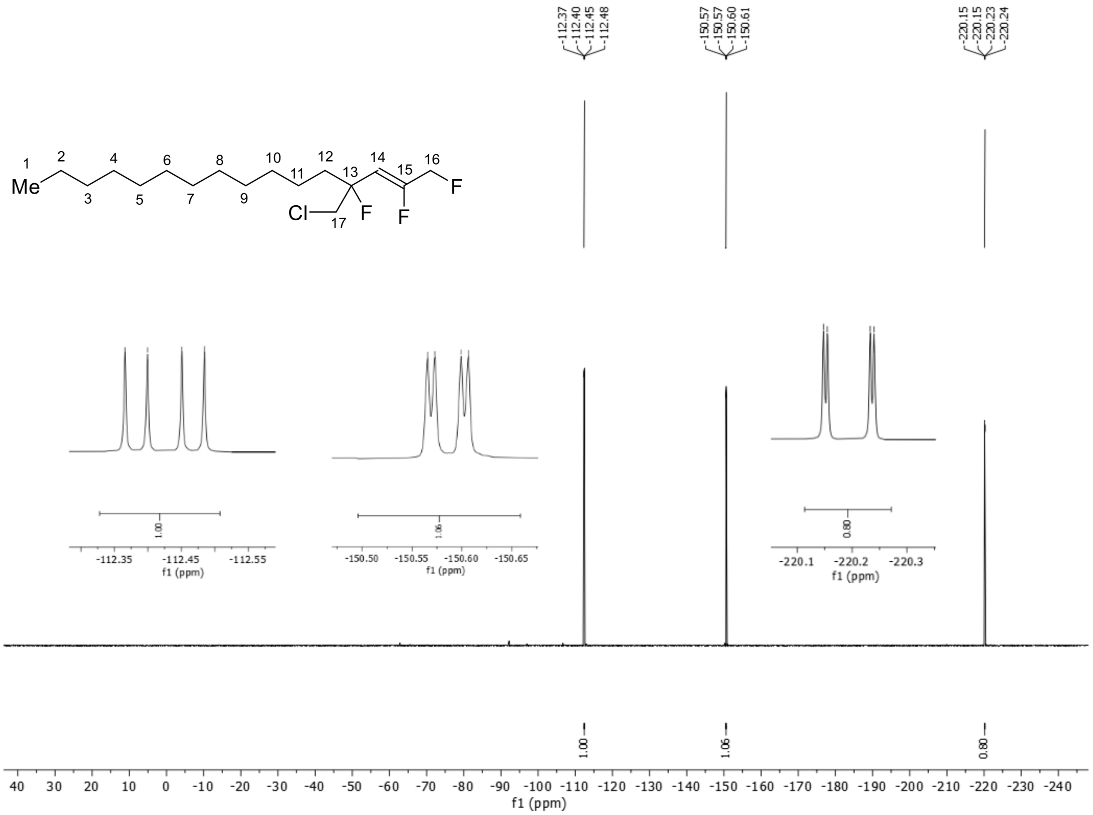

Supplementary Figure 206. $^{19}F\{^1H\}$ NMR of **39** (377 MHz, 299 K, $CDCl_3$).

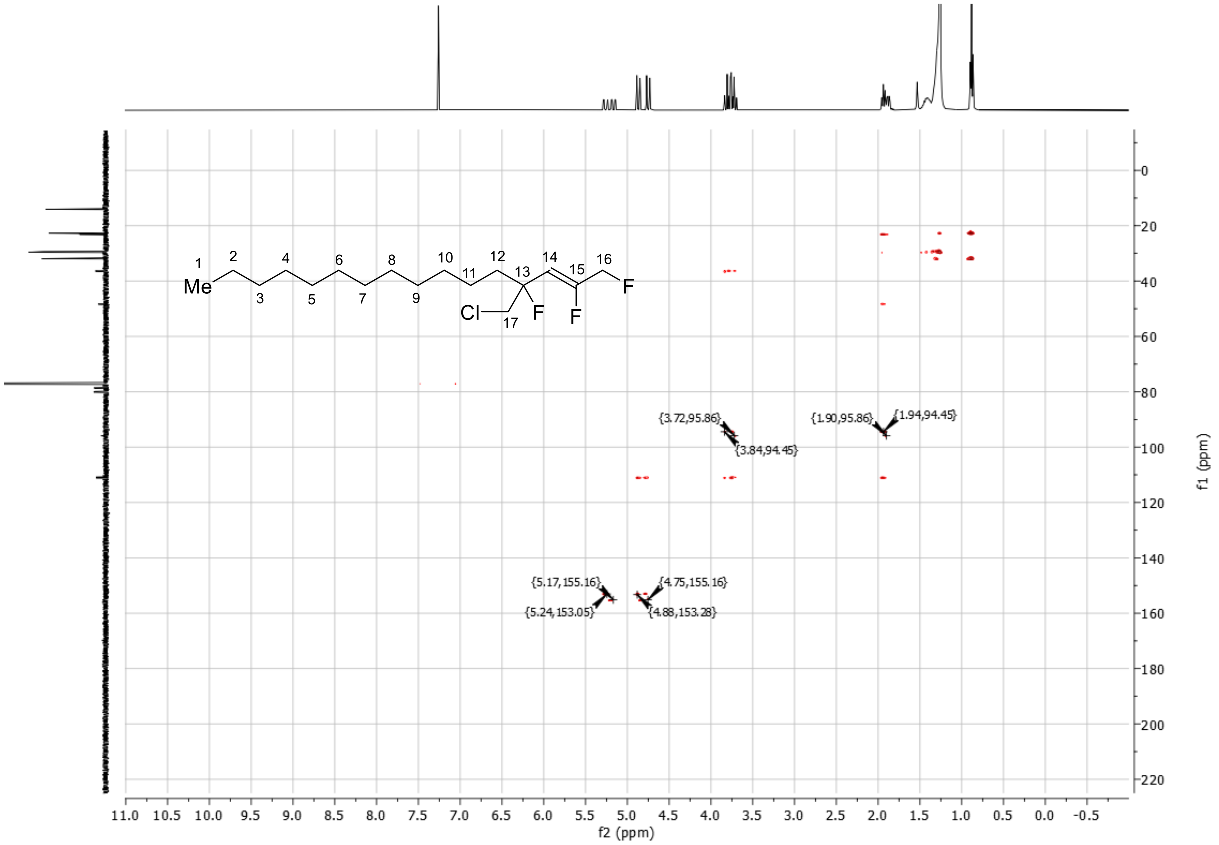

Supplementary Figure 207. HMBC NMR of **39**.

1-(4-(Trifluoromethyl)phenyl)prop-2-yn-1-yl acetate (40)

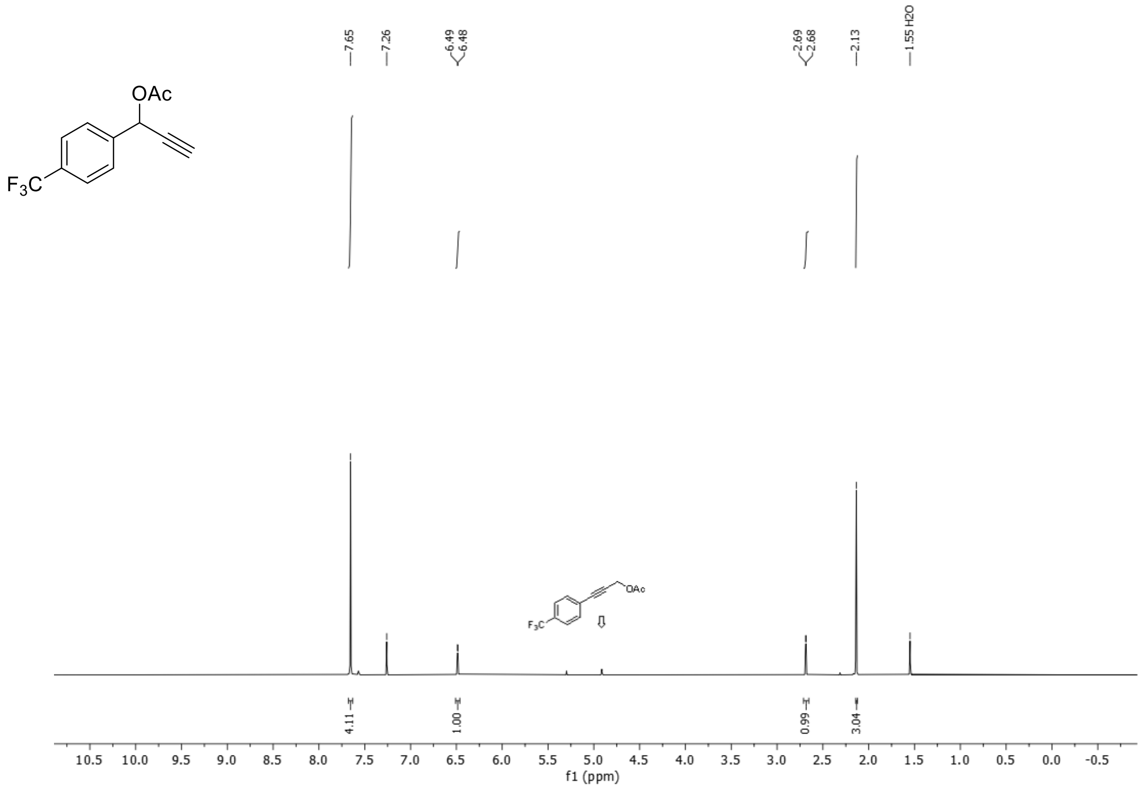

Supplementary Figure 208. ^{1}H NMR of **40** (400 MHz, 299 K, $CDCl_3$).

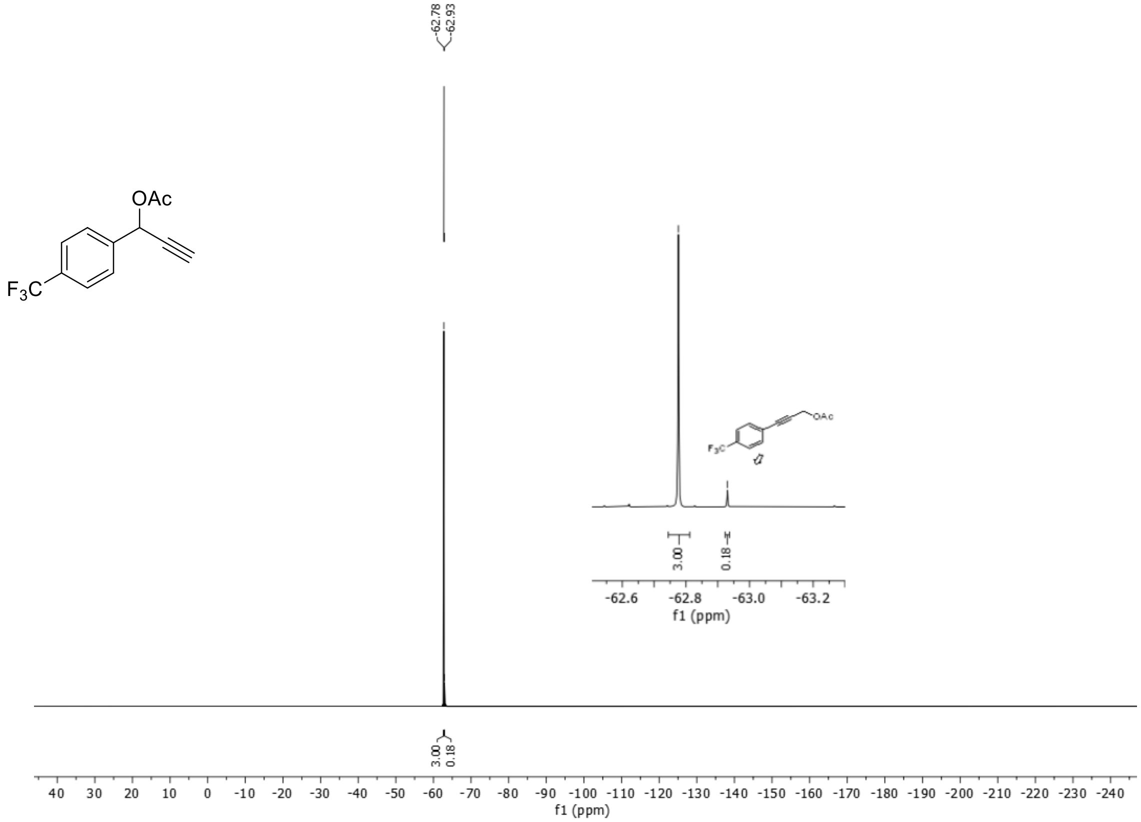

Supplementary Figure 209. $^{19}F\{^{1}H\}$ NMR of **40** (377 MHz, 299 K, $CDCl_3$).

(*Z*)-(3-(4-(trifluoromethyl)phenyl)prop-2-ene-1,2-diyl)dibenzene (41)

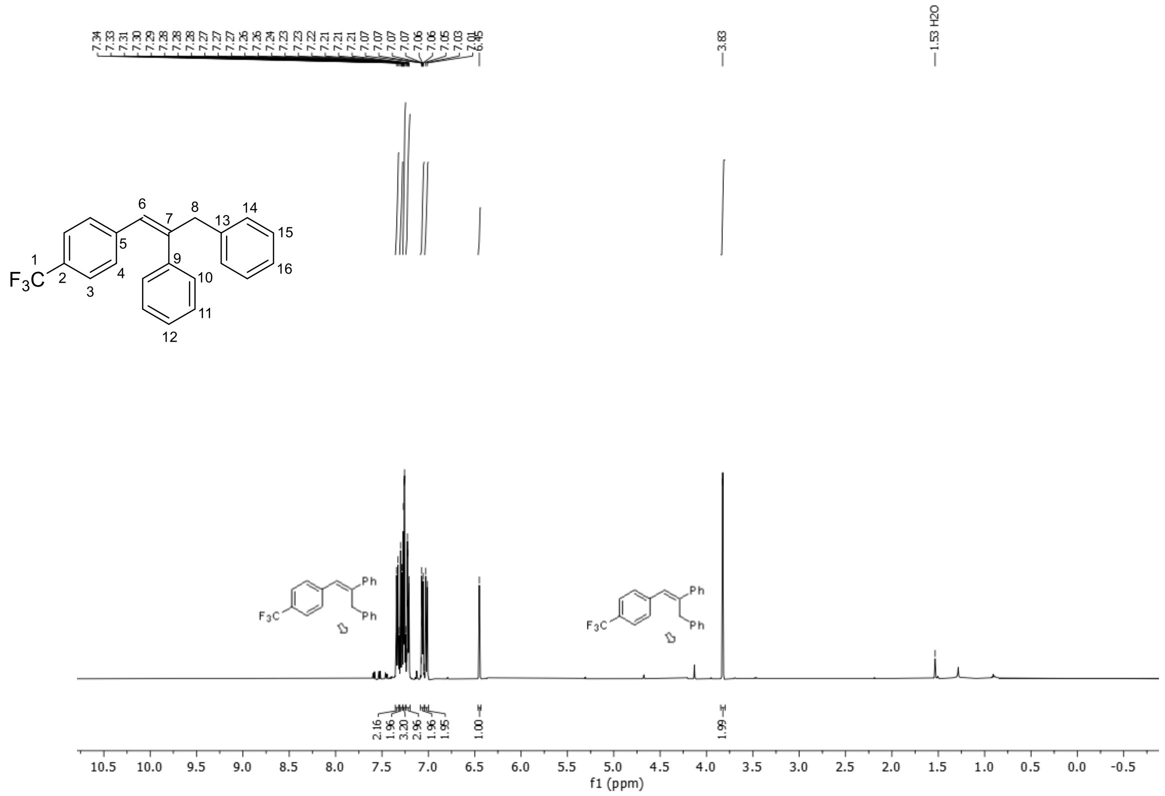

Supplementary Figure 210. ^{1}H NMR of **41** (500 MHz, 299 K, $CDCl_3$).

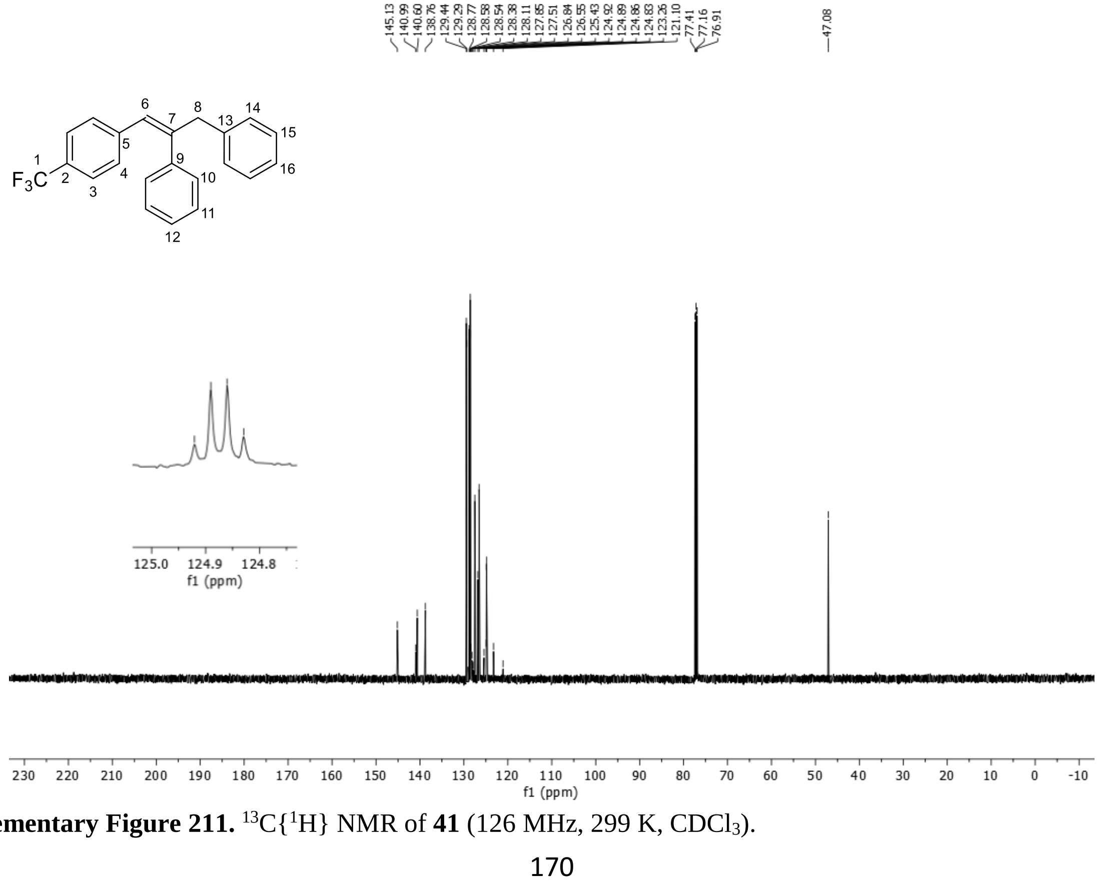

Supplementary Figure 211. $^{13}C\{^{1}H\}$ NMR of **41** (126 MHz, 299 K, $CDCl_3$).

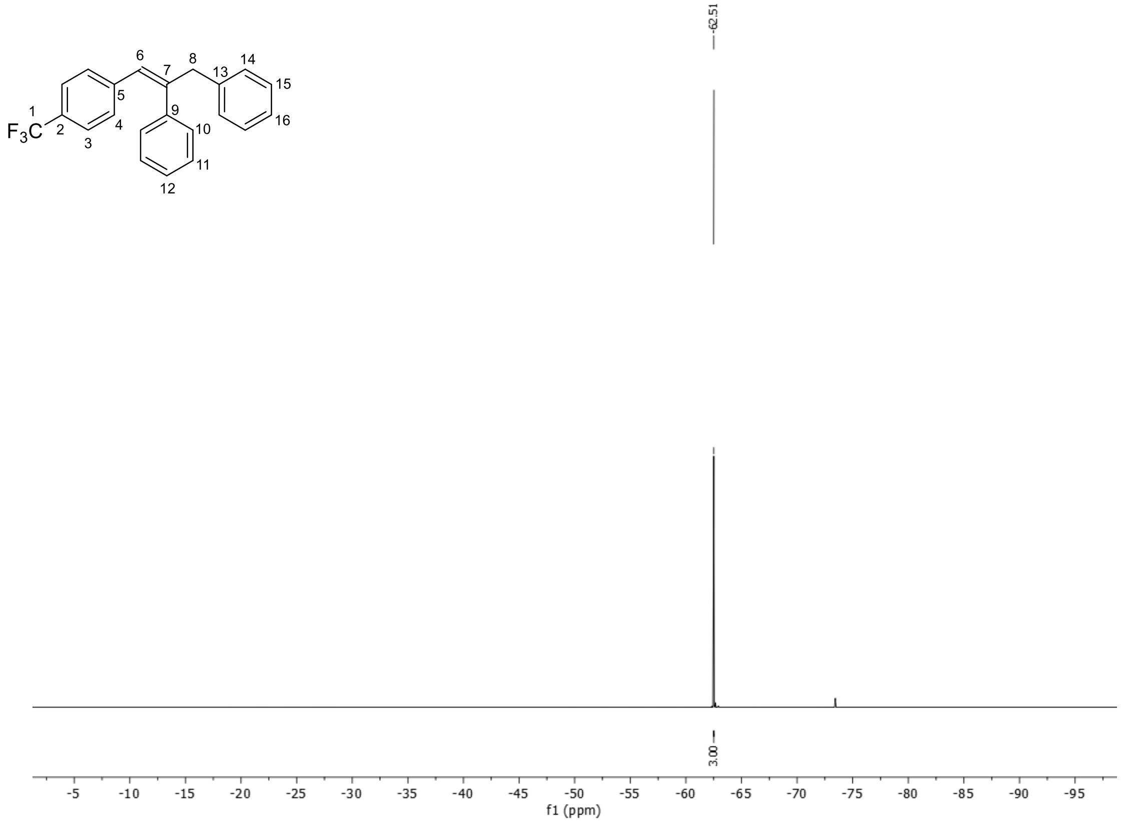

Supplementary Figure 212. ^{19}F NMR of **41** (470 MHz, 299 K, $CDCl_3$).

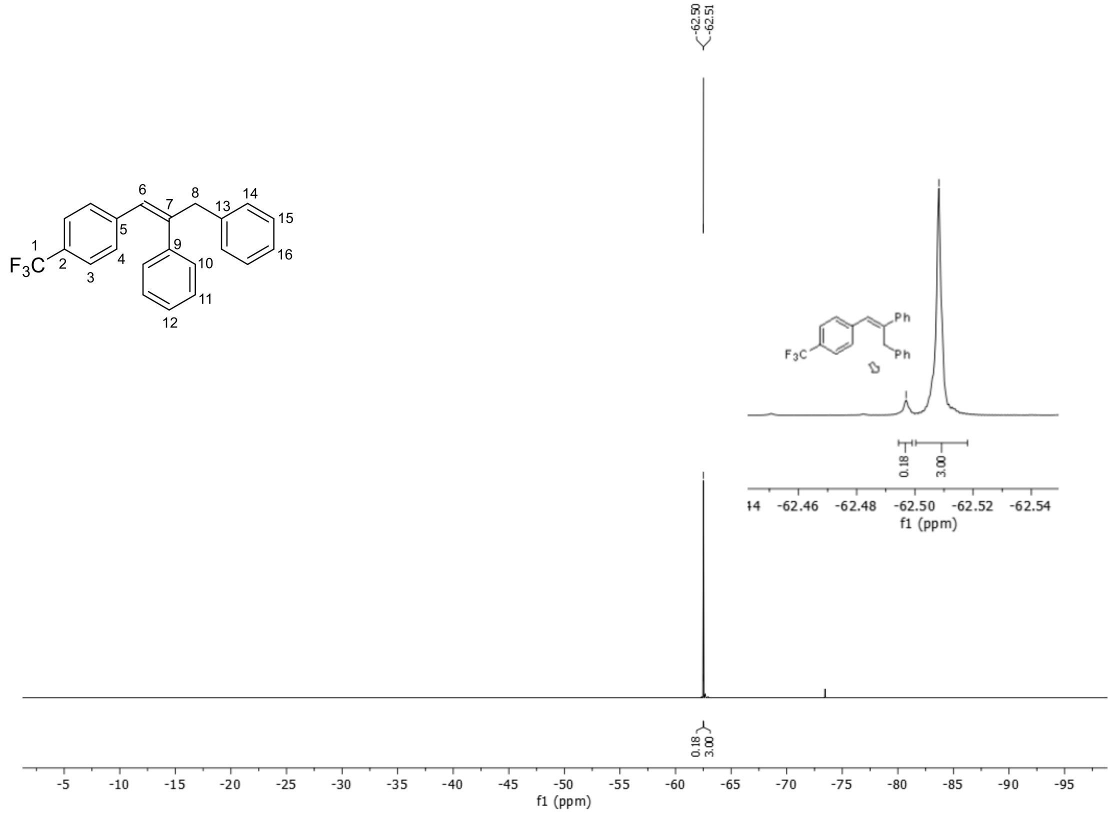

Supplementary Figure 213. $^{19}F\{^1H\}$ NMR of **41** (377 MHz, 299 K, $CDCl_3$).

(*Z*)-1-(2,3-dichloroprop-1-en-1-yl)-4-(trifluoromethyl)benzene (42)

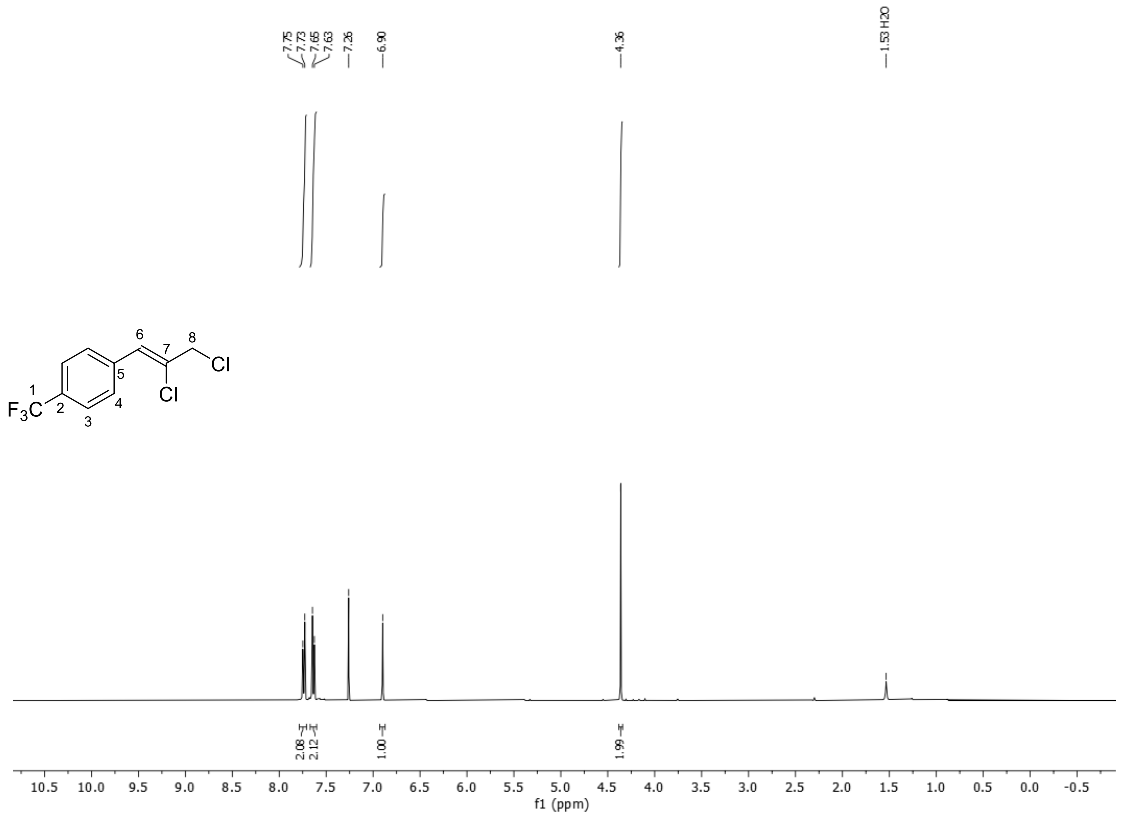

Supplementary Figure 214. ^{1}H NMR of **42** (400 MHz, 299 K, $CDCl_3$).

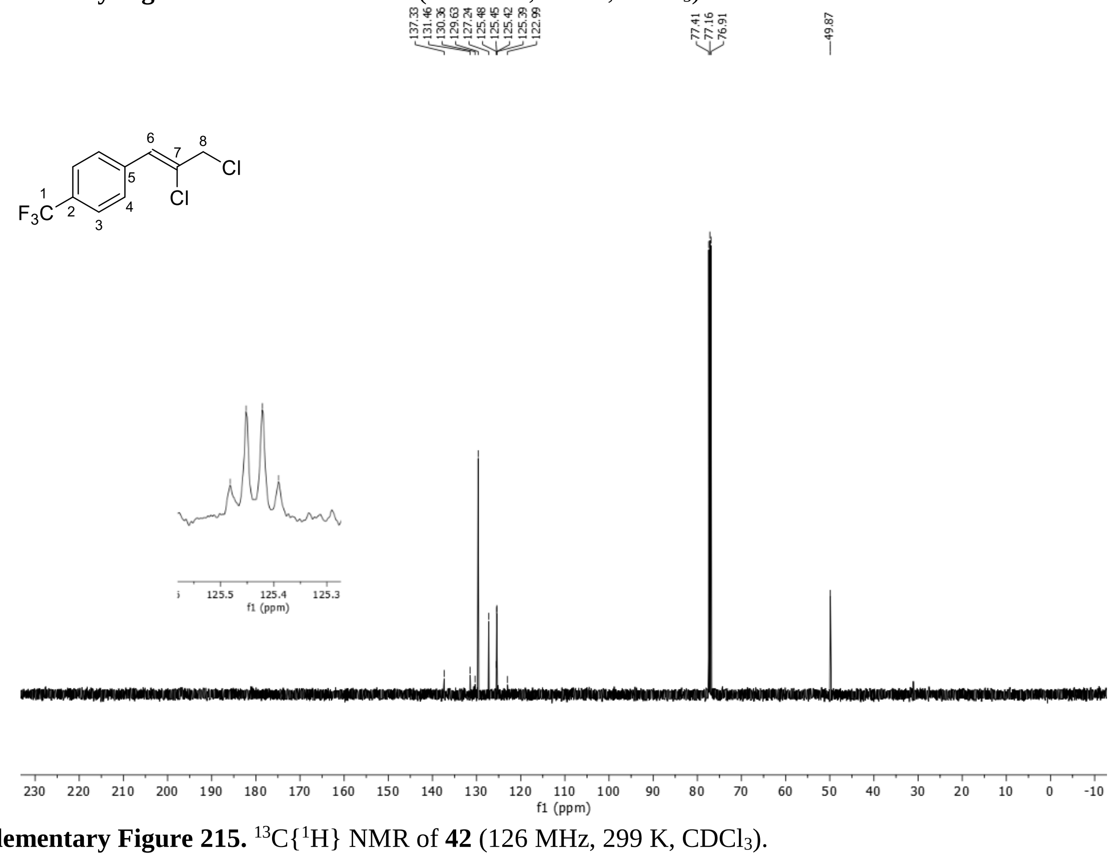

Supplementary Figure 215. $^{13}C\{^{1}H\}$ NMR of **42** (126 MHz, 299 K, $CDCl_3$).

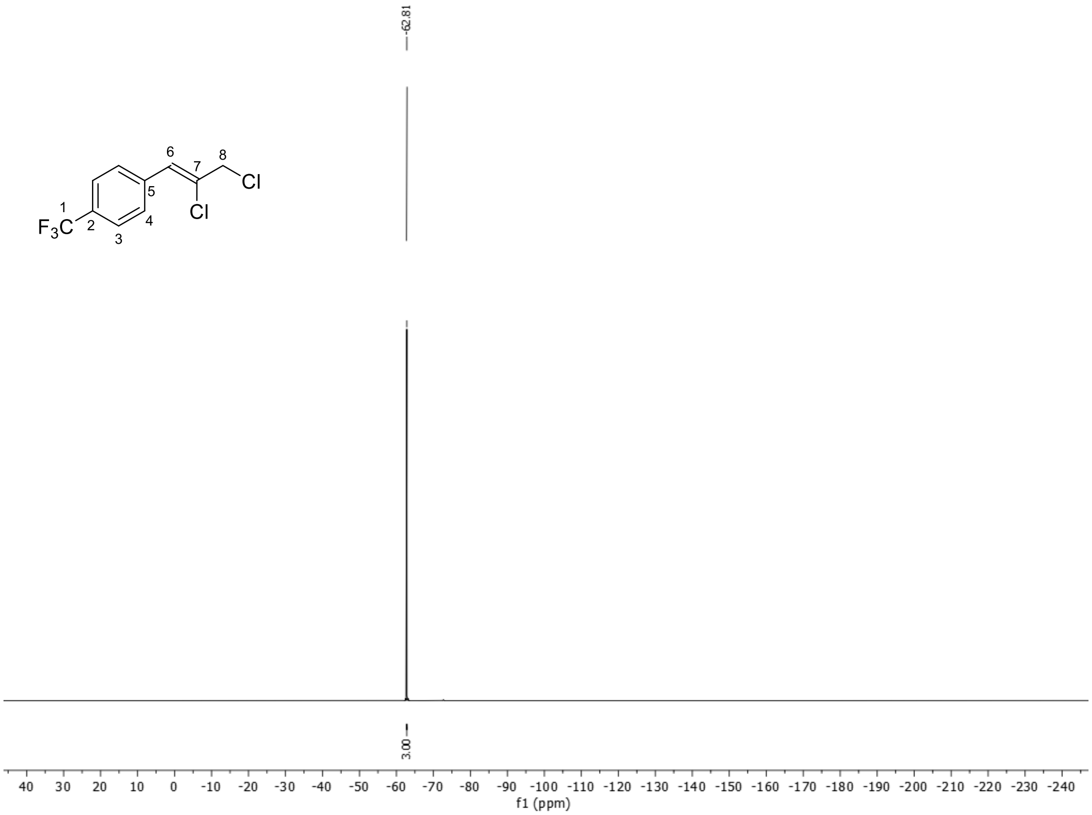

Supplementary Figure 216. ^{19}F NMR of **42** (470 MHz, 299 K, $CDCl_3$).

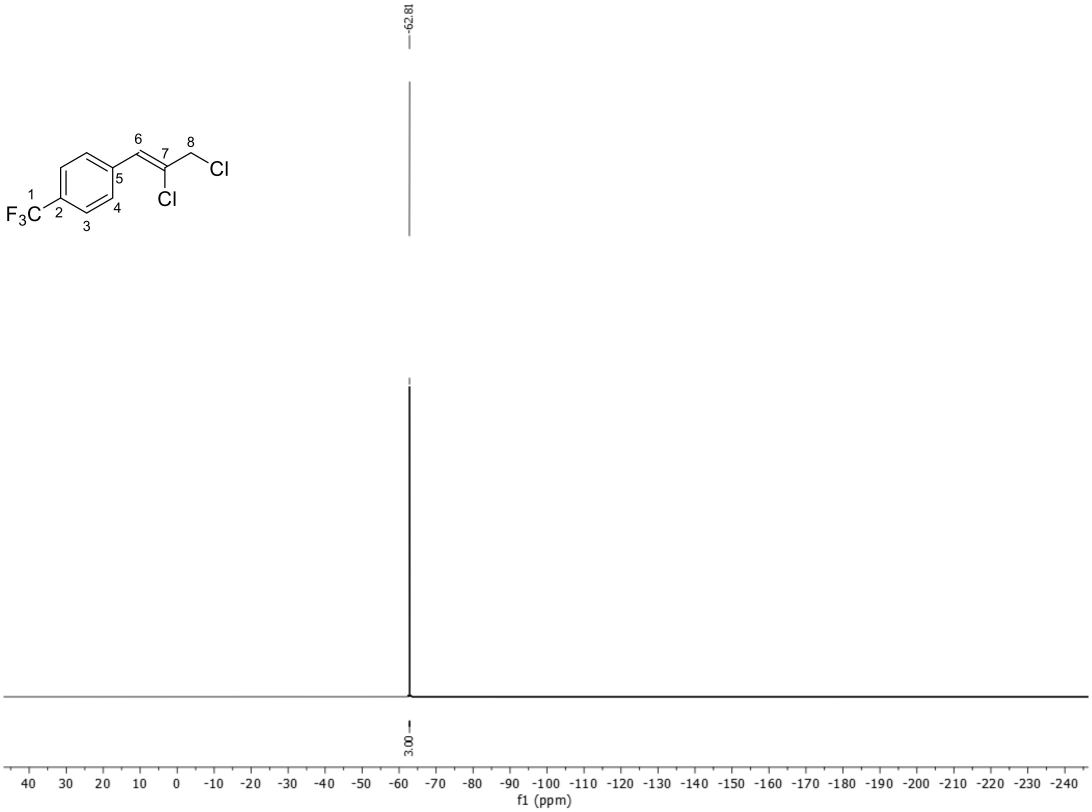

Supplementary Figure 217. $^{19}F\{^1H\}$ NMR of **42** (377 MHz, 299 K, $CDCl_3$).

2. Supplementary References

1. Häfliger, J., Livingstone, K., Daniliuc, C. G. & Gilmour, R. Difluorination of α-(bromomethyl)styrenes via I(I)/I(III) catalysis: facile access to electrophilic linchpins for drug discovery. *Chem. Sci.* **12**, 6148-6152 (2021).
2. Wang, Y. *et al.* Iron-Catalyzed Contrasteric Functionalization of Allenic C(sp^2)-H Bonds: Synthesis of α-Aminoalkyl 1,1-Disubstituted Allenes. *J. Am. Chem. Soc.* **143**, 14998-15004 (2021).
3. Chanthamath, S., Chua, H. W., Kimura, S., Shibatomi, K. & Iwasa, S. Highly Regio- and Stereoselective Synthesis of Alkylidenecyclopropanes via Ru(II)-Pheox Catalyzed Asymmetric Inter- and Intramolecular Cyclopropanation of Allenes. *Org. Lett.* **16**, 3408-3411 (2014).
4. Ganss, S. & Breit, B. Enantioselective Rhodium-Catalyzed Atom-Economical Macrolactonization. *Angew. Chem., Int. Ed.* **55**, 9738-9742 (2016).
5. Rej, S., Klare, H. F. T. & Oestreich, M. Silylium-Ion-Promoted Hydrosilylation of Aryl-Substituted Allenes: Interception by Cyclization of the Allyl-Cation Intermediate. *Org. Lett.* **24**, 1346-1350 (2022).
6. Rochat, R. *et al.* Organomagnesium-Catalyzed Isomerization of Terminal Alkynes to Allenes and Internal Alkynes. *Chem.-Eur. J.* **21**, 8112-8120 (2015).
7. Lainhart, B. C. & Alexanian, E. J. Enantioselective Synthesis of cis-Fused Cyclooctanoids via Rhodium(I)-Catalyzed [4 + 2 + 2] Cycloadditions. *Org. Lett.* **17**, 1284-1287 (2015).
8. Shimp, H. L. & Micalizio, G. C. Group 4 Metals in Polyketide Synthesis: A Convergent Strategy for the Synthesis of Polypropionate-derived (E,E)-Trisubstituted 1,3-Dienes. *Org. Lett.* **7**, 5111-5114 (2005).
9. Kim, S., Lee, Y. & Cho, E. J. Photoredox Selective Homocoupling of Propargyl Bromides. *J. Org. Chem.* **88**, 6382-6389 (2023).
10. Wang, M. *et al.* Synthesis of primary propargylic alcohols from terminal alkynes using rongalite as the C1 unit. *Org. Biomol. Chem.* **21**, 5949-5952 (2023).
11. Conrad, W. E. *et al.* A One-Pot–Three-Step Route to Triazolotriazepinoindazolones from Oxazolino-2H-indazoles. *Org. Lett.* **14**, 3870-3873 (2012).
12. Ma, S., Jiao, N., Zhao, S. & Hou, H. Control of Regioselectivity in Pd(0)-Catalyzed Coupling−Cyclization Reaction of 2-(2’,3’-Allenyl)malonates with Organic Halides. *J. Org. Chem.* **67**, 2837-2847 (2002).
13. Yu, Y.-J., Häfliger, J., Wang, Z.-X., Daniliuc, C. G. & Gilmour, R. Forging Medium Rings via I(I)/I(III)-Catalyzed Diene Carbofunctionalization. *Angew. Chem., Int. Ed.* **62**, e202309789 (2023).
14. Cheng, L.-J. & Cordier, C. J. Catalytic Nucleophilic Fluorination of Secondary and Tertiary Propargylic Electrophiles with a Copper–N-Heterocyclic Carbene Complex. *Angew. Chem., Int. Ed.* **54**, 13734-13738 (2015).
15. Yin, F., Wang, Z., Li, Z. & Li, C. Silver-Catalyzed Decarboxylative Fluorination of Aliphatic Carboxylic Acids in Aqueous Solution. *J. Am. Chem. Soc.* **134**, 10401-10404 (2012).

16. Detz, R. J., Delville, M. M. E., Hiemstra, H. & van Maarseveen, J. H. Enantioselective Copper-Catalyzed Propargylic Amination. *Angew. Chem., Int. Ed.* **47**, 3777-3780 (2008).
